# First transcriptome of the Neotropical pest *Euschistus heros* (Hemiptera: Pentatomidae) with dissection of its siRNA machinery

**DOI:** 10.1038/s41598-020-60078-3

**Published:** 2020-03-17

**Authors:** Deise Cagliari, Naymã Pinto Dias, Ericmar Ávila dos Santos, Leticia Neutzling Rickes, Frederico Schmitt Kremer, Juliano Ricardo Farias, Giuvan Lenz, Diogo Manzano Galdeano, Flávio Roberto Mello Garcia, Guy Smagghe, Moisés João Zotti

**Affiliations:** 1 Department of Crop Protection, Molecular Entomology, Federal University of Pelotas, Pelotas, Brazil; 2 Center for Technological Development, Bioinformatics and Proteomics Laboratory, Federal University of Pelotas, Pelotas, Brazil; 3 Department of Crop Protection, Universidade Regional Integrada do Alto Uruguai, Santo Ângelo, Brazil; 4 Agricultural Research and Development Center, UPL, Pereiras, Brazil; 5 Sylvio Moreira Citrus Center, Agronomic Institute of Campinas, Cordeirópolis, São Paulo Brazil; 6 Department of Crop Protection, Insect Ecology Laboratory, Federal University of Pelotas, Pelotas, Brazil; 7 Department of Plants and Crops, Ghent University, Ghent, Belgium

**Keywords:** Sequencing, RNAi

## Abstract

Over the past few years, the use of RNA interference (RNAi) for insect pest management has attracted considerable interest in academia and industry as a pest-specific and environment-friendly strategy for pest control. For the success of this technique, the presence of core RNAi genes and a functional silencing machinery is essential. Therefore, the aim of this study was to test whether the Neotropical brown stinkbug *Euschistus heros* has the main RNAi core genes and whether the supply of dsRNA could generate an efficient gene silencing response. To do this, total mRNA of all developmental stages was sequenced on an Illumina platform, followed by a *de novo* assembly, gene annotation and RNAi-related gene identification. Once RNAi-related genes were identified, nuclease activities in hemolymph were investigated through an *ex vivo* assay. To test the functionality of the siRNA machinery, *E. heros* adults were microinjected with ~28 ng per mg of insect of a dsRNA targeting the *V-ATPase-A* gene. Mortality, relative transcript levels of *V-ATPase-A*, and the expression of the genes involved in the siRNA machinery, *Dicer-2* (*DCR-2*) and *Argonaute 2* (*AGO-2*), were analyzed. Transcriptome sequencing generated more than 126 million sequenced reads, and these were annotated in approximately 80,000 contigs. The search of RNAi-related genes resulted in 47 genes involved in the three major RNAi pathways, with the absence of *sid-like* homologous. Although *ex vivo* incubation of dsRNA in *E. heros* hemolymph showed rapid degradation, there was 35% mortality at 4 days after treatment and a significant reduction in *V-ATPase-A* gene expression. These results indicated that although s*id-like* genes are lacking, the dsRNA uptake mechanism was very efficient. Also, 2-fold and 4-fold overexpression of *DCR-2* and *AGO-2*, respectively, after dsRNA supply indicated the activation of the siRNA machinery. Consequently, *E. heros* has proven to be sensitive to RNAi upon injection of dsRNA into its hemocoel. We believe that this finding together with a publically available transcriptome and the validation of a responsive RNAi machinery provide a starting point for future field applications against one of the most important soybean pests in South America.

## Introduction

The Neotropical brown stink bug (BS), *Euschistus heros* (Hemiptera: Pentatomidae), is one of the most important Pentatomidae pests in South America^1^, especially in soybean (*Glycine max*) with a reduction in seed quality and yield^2^. Stink bugs use their piercing/sucking mouthparts to inject enzymes into the plant tissues to digest plant components and remove pre-digested fluids^3^. Although rarely reported before the 70s^2,4^, since then population outbreaks^2,5^ and rapid population growth have allowed expansion of the range of *E. heros* to all the major South American soybean production regions, including Brazil^2^, Paraguay^2^, and Argentina^6^.

The current recommendations for the management of this insect rely on the use of broad-spectrum insecticides such as organophosphates and pyrethroids (AGROFIT, http://agrofit.agricultura.gov.br/agrofit_cons/principal_agrofit_cons). However, these are detrimental to the environment and some are harmful to beneficial organisms. Furthermore, the high infestation of *E. heros* has frequently been reported and the lack of a sustainable alternative for pest control has led growers frequently to spray insecticides from the same chemical group, contributing to the selection of resistant strains^7–9^. Moreover, due to favorable weather conditions found in Brazil, Argentina and Paraguay, multiple generations occur during a crop season, making the control even more difficult. Therefore, effective and environmental-friendly multiple control strategies are needed to reduce the use of highly toxic pesticides and to delay resistance development in *E. heros*.

RNA interference (RNAi), also known as Post-Transcriptional Gene Silencing (PTGS), is a natural mechanism of gene regulation and a defense system against viruses in eukaryotic cells^10,11^, and since the milestone work done by Baum *et al*.^12^, RNAi towards insect management has significantly attracted interest as an alternative control strategy to synthetic insecticides. In 2017, genetically modified maize using RNAi-based technology against *Diabrotica virgifera virgifera* (Coleoptera: Chrysomelidae), an important pest in the United States of America (USA), has been approved by the Environmental Protection Agency (EPA) in the USA^13^. Besides the use of RNAi in plants, RNA-based spray insecticides, focusing on non-transformative approaches, are expected to be introduced into the market soon^14^, with significant advances in the use of SIGS (Spray-Induced Gene Silencing)^15,16^.

RNAi triggers gene silencing through non-coding RNAs (ncRNAs), such as micro RNAs (miRNAs) and small interfering RNA (siRNA), originally generated from double-stranded RNA (dsRNA)^17^, and Piwi-interacting RNA (piRNA)^18^. The success of the RNAi relies on the ability of the insect cells to efficiently uptake the dsRNA from the environment^19^ and activate the silencing machinery. The process of dsRNA uptake can be mediated by transmembrane channel proteins such as sid-like (systemic interference defective-like)^20–22^, or endocytosis^23–27^, allowing gene silencing in cells/tissues distant from the uptake point^19,28^. Once inside cells, dsRNAs are processed into siRNA fragments, with ~20 base pairs (bp), by the ribonuclease III enzyme Dicer 2 (DCR-2)^29^. These siRNAs are incorporated into the RISC (RNA-Induced Silencing Complex), which contains the Argonaute 2 (AGO-2) protein^30^ allowing the specific breakdown of messenger RNA (mRNA) and so preventing the protein formation^19^.

Transcriptome analysis focusing on RNAi as a control strategy has been reported in insects mainly for Coleoptera^31–33^, Lepidoptera^34^ and Hemiptera^35^. According to some studies, RNAi is less efficient in Hemiptera^36,37^ when compared to Coleoptera because of the presence of double-stranded ribonucleases (*dsRNases*)^38–40^. In the pea aphid, *Acyrthosiphon pisum* (Hemiptera: Aphidoidea), the lack of RNAi response was associated with the high nuclease activity in hemolymph^41^. However, the brown marmorated stink bug, *Halyomorpha halys* (Heteroptera: Pentatomidae), has lower nuclease activities and gene silencing can reach up to 70% when compared to *Heliothis virescens* (Lepidoptera: Noctuidae)^42^. Successful use of RNAi through oral delivery has been reported in other hemipteran species such as *Diaphorina citri* (Hemiptera: Lividae)^43,44^, *Bemisia tabaci* (Hemiptera: Aleyrodidae)^45^, and *Nilaparvata lugens* (Homoptera: Delphacidae)^46^, suggesting that RNAi could be further investigated towards a control strategy in *E. heros*.

Transcriptome analysis allows researchers to understand the RNAi mechanism and its main components as well as helping in the selection of target genes, essential genes involved in biological processes and housekeeping genes. Therefore, the main goal of our work was to provide a transcriptome dataset for *E. heros*, characterize the genes involved in the RNAi pathways, and validate the RNAi machinery through a gene silencing assay. In brief, the RNAi core genes were identified, and the efficiency of the siRNA machinery was tested through injection of dsRNA followed by quantitative real-time PCR. Next, considering the importance of dsRNA degradation by nucleases, an *ex vivo* assay was performed with collected hemolymph. Finally, dsRNAs were designed to target *V-ATPase subunit A* gene, resulting in mortality after microinjection. To test the activation of the siRNA machinery, an upregulation of *DCR-2* and *AGO-2* was also investigated. Overall, these data will provide for the first time the dissection of siRNA pathway in *E. heros* and with an efficient dsRNA cellular uptake system, resulting in significant insect mortality. These data could then be further explored to develop a pest control strategy using RNAi.

## Results

### Analysis of *E. heros* transcriptome

RNA sequencing resulted in a total of 126,455,838 reads of 101 bp long, corresponding to an accumulated length of 12,772,039,638 bp. *De novo* assembling using Trinity software resulted in 147,612 transcripts, assembled into 83,114 contigs with an average length of 1,000 bp and an average GC content of 37.12%.

**Figure 1:**
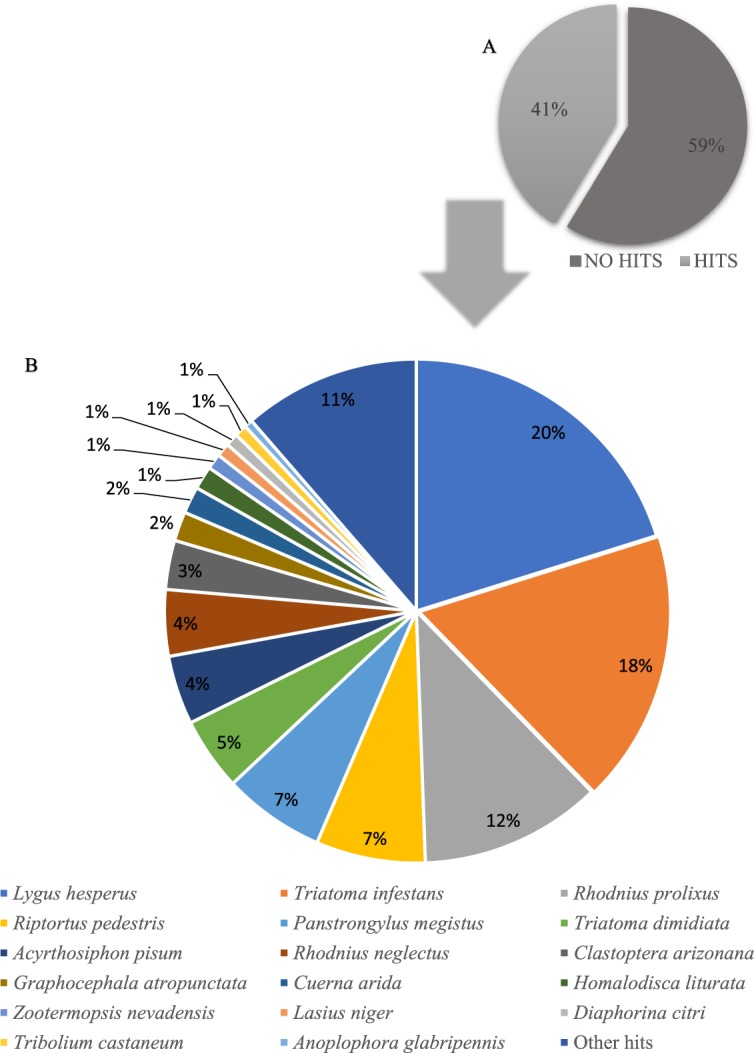
*Euschistus heros* sequence comparison to other insect species. (**A**) Total transcripts (%) with known and unknown protein sequences in *E. heros* using BLASTx search. (**B**) BLASTx comparison of *E. heros* known sequences to other insect genera (bitscore>50) against the nr protein database of the NCBI.

Based on Diamond analysis, a total of 60,956 hits was produced, representing 41.30% of the total transcripts (Fig. 1A). Out of the sequences, 60,227 hits (98.8%) were from Eukaryotes, with 84.64% of the contigs similar to sequences from Hemiptera species: 20.16% to the *Lygus hesperus* (Hemiptera: Miridae), 17.57% to *Triatoma infestans* (Hemiptera: Reduviidae), 11.69% to *Rhodnius prolixus* (Hemiptera: Reduviidae), 7.03% to *Riptortus pedestris* (Hemiptera: Alydidae), 6.55% to *Panstrongylus megistus* (Hemiptera: Reduviidae), 4.69% to *Triatoma dimidiate* (Hemiptera: Reduviidae), 4.43% to *A. pisum*, 4.26% to *Rhodnius neglectus* (Hemiptera: Reduviidae), 3.15% to *Clastoptera arizonana* (Hemiptera: Clastopteridae), 1.88% to *Graphocephala atropunctata* (Hemiptera: Cicadellidae), 1.73% to *Cuerna arida* (Hemiptera: Cicadellidae), 1.50% to *Homalodisca liturata* (Hemiptera: Cicadellidae). The reminding 15.36% belonged to *Zootermopsis nevadensis* (Isoptera: Archotermopsidae) (1.00%), *Lasius niger* (Hymenoptera: Formicidae) (0.87%), *D. citri* (0.83%), *Tribolium castaneum* (Coleoptera: Tenebrionidae) (0.78%) and *Anoplophora glabripennis* (Coleoptera: Cerambycidae) (0.54%), and other hits (11.34%) (Fig. 1B, Supplementary Table S1). The raw reads have been deposited in the sequence reads archive (SRA) at NCBI, and can be accessed using SRP159293 accession number.

**Figure 2:**
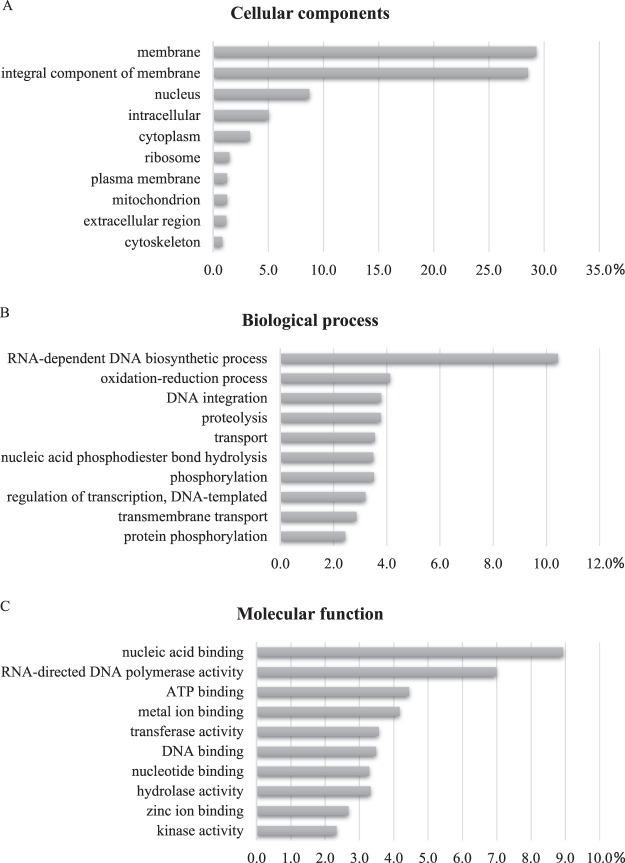
Percentage of *E. heros* contigs assigned to a gene ontology term as predicted by QuickGO from EBI. (**A**) Cellular components. (**B**) Biological process. (**C**) Molecular function.

A total of 143,806 predicted GO terms was obtained and grouped into three categories: cellular components, biological processes, and molecular functions. Membrane was the most dominant GO term within the cellular component (28,631; 29.3%), for the biological processes it was RNA-dependent DNA biosynthesis process (46,238; 10.4%), and for the molecular function was nucleic acid binding (68,937; 8.9%) (Fig. 2A–C).

### Identification of RNAi-related genes

The result of the *E. heros* transcriptome search for RNAi-related genes revealed the presence of 47 genes associated with dsRNA uptake, RNAi core machinery, auxiliary RISC factors, nucleases, antiviral RNAi, and intracellular transport. Some RNAi-related proteins presented variants, with the presence or absence of conserved domains. Overall, the sequences of *H. halys* showed the highest similarity to sequences from *E. heros*.

#### dsRNA uptake

The protein sequences involved in dsRNA uptake were searched in the *E. heros* transcriptome, and a total of six proteins related to this process was found, although there was an absence of *sid-like* genes (Table 1, Supplementary data S1). Scavenger protein was found with a CD36 domain region, Ubiquitin-protein transferase (FBX011) with an F-box conserved domain and three beta-helices, and Epsin 2 with an Epsin N-terminal homology (ENTH) domain. The Clathrin heavy chain (Chc) protein and Gap Junction Protein with an Innexin conserved domain were also found in the *E. heros* transcriptome.

**Table 1:** Overview of identified genes related to the dsRNA uptake in *E. heros*.

Gene ID	Transcripts Per Million (TPM)	First hit BLASTp	Homologue ID	Comparison	Identity (%)
Scavenger	818,716	Scavenger receptor class B member 1, partial	XP_024218066.1 (*Halyomorpha halys*)	E = 0.0; bits = 1039	96
CG4966 = orthologous to the Hermansky-Pudlak Syndrome4	310,363	Uncharacterized protein LOC106688690	XP_014288755.1 (*Halyomorpha halys*)	E = 0.0; bits = 1271	90
F-box protein 11 (FBX011)	102,285	F-box only protein 11	XP_014287303.1 (*Halyomorpha halys*)	E = 0.0; bits = 1794	99
Clathrin heavy chain (Chc)	960,642	Clathrin heavy chain	XP_014287090.1 (*Halyomorpha halys*)	E = 0.0; bits = 3477	99
Epsin 2 (Epn2)	141,317	Epsin-2 isoform X5	XP_014270392.1 (*Halyomorpha halys*)	E = 0.0; bits = 900	92
Gap Junction protein (Innexin2)	324,716	Innexin inx2	XP_014292574.1 (*Halyomorpha halys*)	E = 0.0; bits = 736	60

#### Core RNAi machinery

Proteins related to the miRNA, siRNA and piRNA pathways were identified in the *E. heros* transcriptome (Table 2, Supplementary data S2).

**Table 2:** Overview of the core RNAi-related genes in *E. heros*.

Gene ID	Transcripts Per Million (TPM)	First hit BLASTp	Homologue ID	Comparison	Identity (%)
**miRNA**					
DCR-1	0.775	Dcr-1	AVK59457.1 (*Nezara viridula*)	E = 0.0; bits = 2109	91
AGO-1 isoform 1	0.585	Argonaute-1-PC	AVK59466.1 (*Nezara viridula*)	E = 0.0; bits = 1924;	99.89
AGO-1 isoform 3	0.103	Argonaute-1-PC	AVK59466.1 (*Nezara viridula*)	E = 0.0; bits = 1923;	99.89
AGO-1 isoform 4	0.407	Argonaute-1-PC	AVK59466.1 (*Nezara viridula*)	E = 0.0; bits = 1924;	99.89
AGO-1 isoform 5	118,437	Protein argonaute-2 isoform X3	XP_014287705.1 (*Halyomorpha halys*)	E = 0.0; bits = 1877	99
Loquacious	379,404	RISC-loading complex subunit tarbp2-like isoform X1	XP_014274312.1 (*Halyomorpha halys*)	E = 0.0; bits = 521	96
Drosha	112,718	Ribonuclease 3	XP_014278529.1 (*Halyomorpha halys*)	E = 0.0; bits = 2366	91
Pasha	692,682	Microprocessor complex subunit DGCR8	XP_014282581.1 (*Halyomorpha halys*)	E = 0.0; bits = 1078	89
Exportin-5	60,305	Exportin-5	XP_014280932.1 (*Halyomorpha halys*)	E = 0.0; bits = 2420	98
**siRNA**					
DCR-2 isoform 1	646,601	Endoribonuclease Dicer isoform X1	XP_014275310.1 (*Halyomorpha halys*)	E = 0.0; bits = 2795	83
DCR-2 isoform 2	0.618	Endoribonuclease Dicer isoform X2	XP_014275311.1 (*Halyomorpha halys*)	E = 0.0; bits = 852	88
AGO-2 isoform 1	146,222	Argonaute 2	AVK59468.1 (*Nezara viridula*)	E = 0.0; bits = 565	80
AGO-2 isoform 2	0.137	Argonaute 2	AVK59468.1 (*Nezara viridula*)	E = 0.0; bits = 1516	75
R2D2	350,347	Interferon-inducible double-stranded RNA-dependent protein kinase activator A-like isoform X1	XP_014288218.1 (*Halyomorpha halys*)	E = 0.0; bits = 559	82
**piRNA**					
AGO-3	227,644	Protein argonaute-3	XP_014276831.1 (*Halyomorpha halys*)	E = 0.0; bits = 1595	85
Aubergine (AUB)	0.750	Protein Aubergine-like	XP_014270559.1 (*Halyomorpha halys*)	E = 0.0; bits = 1676	96
Piwi	579,184	Protein Aubergine-like isoform X3	XP_014275927.1 (*Halyomorpha halys*)	E = 0.0; bits = 1172;	63
Zucchini (Zuc)	0.13543	Mitochondrial cardiolipin hydrolase	XP_014288409.1 (*Halyomorpha halys*)	E = 1e-152; bits = 432	86

The DCR-1 protein was found in *E. heros* with the conserved PAZ (Piwi, Argonaute and Zwille) domain, two RNaseIII domains and a Double-stranded RNA-binding domain (DSRBD), with an absence of the helicase domains. DCR-2 was also found in *E. heros* with two isoforms as following: 1 and 2 with 646,601 and 0.618 transcripts per million (TPM), respectively. The DCR-2 isoform 1 contained all the conserved domains: one helicase domain, one PAZ domain, two RNaseIII domains, and a DSRBD, while DCR-2 isoform 2 was found with two RNaseIII domains and a Ribonuclease III C terminal domain (RIBOc). Dicer 3 protein was not found in the *E. heros* transcriptome. Drosha protein was found with two RNaseIII domains and a RIBOc, but with the absence of PAZ, and an amino-terminal DExH-box helicase domain. The dsRNA-binding proteins Pasha, Loquacious and R2D2 were also identified in *E. heros* with conserved domains (DSRBDs). Argonaute superfamily proteins were also searched in the *E. heros* transcriptome and five members of the Argonaute superfamily proteins were identified: AGO-1, AGO-2, AGO-3, Aubergine (Aub) and Piwi (Table 2, Supplementary data S2). Four variants of the AGO-1 protein were found: isoforms 1, 3, 4 and 5, presenting 0.585, 0.103, 0.4 and 118,437 TPM, respectively. All AGO-1 isoforms were found with the PAZ and PIWI conserved domains. For the AGO-2 protein, two isoforms were found, isoform 1 and 2, with 146,222 and 0.14 TPM, respectively. AGO-2 isoform 1 was found with PAZ and PIWI conserved domains, while AGO-2 isoform 2 had no PAZ domains. AGO-3, Aub and Piwi proteins presented the PAZ and PIWI conserved domains. Zucchini (Zuc), with a nuclease conserved domain, was also found in the *E. heros* transcriptome.

Phylogenetic analyses revealed distinct groups for DCR and AGO superfamily proteins (Supplementary Fig. S1, S2). The protein DCR-1 from *E. heros* was grouped in a clade with the DCR-1 proteins from *Nezara viridula* (Hemiptera: Pentatomidae) and *H. halys*, and the same results were found for *E. heros* DCR-2 (Supplementary Fig. S1). Also, *E. heros* DCR-1 was grouped in a distinct clade compared to *E. heros* DCR-2, but it showed a common ancestor. The phylogenetic analysis of the AGO superfamily resulted in two main clades, one contained the AGO subfamily proteins, AGO-1 and AGO-2, while the other had the PIWI subfamily proteins, AGO-3, Aub and Piwi (Supplementary Fig. S2). *E. heros* AGO-1 was clustered with AGO-1 from *N. viridula* and *N. lugens*, while *E. heros* AGO-2 was clustered in a second group together with other proteins of this family. *E. heros* AGO-3 was clustered in a distinct group as well as Aub and Piwi proteins.

#### Auxiliary RISC factor

The *E. heros* transcriptome was searched for RNAi auxiliary factors (Table 3, Supplementary data S3). The research resulted in 17 intracellular factors associated with the RISC. The Tudor-SN (TSN) protein sequence, with a Tudor-conserved domain, and the Translin and Translin-associated factor-X (TRAX), conserved subunits of the component 3 promoter of the RISC (C3PO), were identified in *E. heros*. The Armitage (Armi), spindle-E (Spn-E), Maelstrom, Gawky, Staufen (STAU) and CLIP-associating protein (Clp-1) were also present in the *E. heros* transcriptome with all conserved domains. HEN-1 nuclease was also present, but no conserved domain was found (DSRBD, FK506 binding protein-like domain or methyltransferase domain). Other auxiliary RISC factors identified in *E. heros* were the Elongator complex protein 1 (Elp-1), Vasa intronic gene (VIG), DEAD-box RNA helicases, PRP16 with a DExD conserved domain, Belle with the conserved DEAD-box domain, Glucose dehydrogenase (GLD-1) and Cytoplasmic aconitate hydratase (ACO-1).

**Table 3:** Overview of identified genes associated to RISC complex in *E. heros*.

Gene ID	Transcripts Per Million (TPM)	First hit BLASTp	Homologue ID	Comparison	Identity (%)
Tudor-SN	0.144	Tudor domain-containing protein 1-like isoform X2	XP_014284230.1 (*Halyomorpha halys*)	E = 0.0; bits = 2031	76
Translin	569,424	Translin	XP_014290495.1 (*Halyomorpha halys*)	E = 1e-154; bits = 434	85
Similar to translin associated factor-X (TRAX)	257,569	Translin-associated protein X isoform X1	XP_014289754.1 (*Halyomorpha halys*)	E = 3e-162; bits = 456	85
Armitage	0.284	Probable RNA helicase armi	XP_014289817.1 (*Halyomorpha halys*)	E = 0.0; bits = 1110	96
Homeless (spindle-E)	0.963535	Probable ATP-dependent RNA helicase spindle-E	XP_014286769.1 (*Halyomorpha halys*)	E = 0.0; bits = 2707	89
Maelstrom	126,192	Protein maelstrom homolog	XP_014290039.1 (*Halyomorpha halys*)	E = 0.0; bits = 694	79
HEN1	0.292	Uncharacterized protein LOC106685926	XP_014284423.1 (*Halyomorpha halys*)	E = 0.0; bits = 656	70
PRP16, mut6 homolog	351,652	Pre-mRNA-splicing factor ATP-dependent RNA helicase PRP16	XP_014279344.1 (*Halyomorpha halys*)	E = 0.0; bits = 2423	96
Clp1 homolog (kinase)	0.999	CLIP-associating protein	XP_014275582.1 (*Halyomorpha halys*)	E: 0.0; bits = 2731	94
Elp-1	221,567	Elongator complex protein 1	XP_014290480.1 (*Halyomorpha halys*)	E = 0.0; bits = 2045	82
GLD-1 homolog	0.03	Glucose dehydrogenase [FAD, quinone]-like isoform X1	XP_014290348.1 (*Halyomorpha halys*)	E = 0.0; bits = 1073	87
ACO-1 homolog	281,389	Cytoplasmic aconitate hydratase-like	XP_014275296.1 (*Halyomorpha halys*)	E: 0.0; bits = 1660	92
Vasa intronic gene (VIG)	658,838	Plasminogen activator inhibitor 1 RNA-binding protein-like isoform X2	XP_014292052.1 (*Halyomorpha halys*)	E = 0.0; bits = 644	96
Staufen	0.147	Double-stranded RNA-binding protein Staufen homolog 2 isoform X5	XP_014282526.1 (*Halyomorpha halys*)	E = 0.0; bits = 956	96
RNA helicase Belle	763,119	ATP-dependent RNA helicase bel isoform X2	XP_014279436.1 (*Halyomorpha halys*)	E = 0.0; bits = 1377	97
Protein arginine methyltransferase 7 (PRMT)	244,103	Protein arginine methyltransferase NDUFAF7, mitochondrial	XP_014292128.1 (*Halyomorpha halys*)	E = 0.0; bits = 726	84
Gawky	135,069	Protein Gawky isoform X1	XP_014288686.1 (*Halyomorpha halys*)	E = 0.0; bits = 2803	97

#### Nucleases

Exoribonuclease 1 (Eri-1) and DNA/RNA non-specific endonuclease (dsRNase) proteins were found in the *E. heros* transcriptome (Table 4, Supplementary data S4). Eri-1 was found with a 5′-3′ exonuclease N-terminus domain (XRN_N). The dsRNase protein was found with seven isoforms, 1, 3, 4, 6, 7, 9 and 10 with 0.17, 0.30, 0.46, 702,558, 719,814, 292,033 and 280,771 TMP, respectively. The isoforms presented a DNA/RNA non-specific endonuclease (Endonuclease_NS) conserved domain, except the isoform 3, which did not show any conserved domain. Small RNA degrading nuclease 1 (SDN1-like) and Nibbler were found with the 3′-5′ exonuclease conserved domain (Table 4, Supplementary data S4). The phylogenetic analyses revealed distinct clades among nuclease proteins, being the Eri-1, dsRNases, SDN1 and Nibbler grouped in clades together with these proteins from other insect species (Supplementary Fig. S3)

**Table 4:** Overview of identified genes associated with RNAi in *E. heros*.

Gene ID	Transcripts Per Million (TPM)	First hit BLASTp	Homologue ID	Comparison	Identity (%)
**Nucleases**					
Exoribonuclease 1 (Eri1)	388,358	5′-3′ exoribonuclease 1	XP_014290344.1 (*Halyomorpha halys*)	E = 0.0; bits = 2701	83
DNA/RNA non-specific endonuclease isoform 1	0.171	Uncharacterized protein LOC106684787	XP_024218583.1 (*Halyomorpha halys*)	E: 6e-172; bits = 490	83.4
DNA/RNA non-specific endonuclease isoform 3	0.294	Uncharacterized protein LOC106691872	XP_014293261.1 (*Halyomorpha halys*)	E = 2e-18; bits = 83.6	56
DNA/RNA non-specific endonuclease isoform 4	0.456	Uncharacterized protein LOC106684787	XP_024218583.1 (*Halyomorpha halys*)	E = 8e-173; bits = 486	85
DNA/RNA non-specific endonuclease isoform 6	702,558	Uncharacterized protein LOC106684787	XP_024218583.1 (*Halyomorpha halys*)	E = 4e-170; bits = 486	83.4
DNA/RNA non-specific endonuclease isoform 7	719,814	Uncharacterized protein LOC106684787	XP_024218583.1 (*Halyomorpha halys*)	E = 4e-170; bits = 486	83
DNA/RNA non-specific endonuclease isoform 9	292,033	Uncharacterized protein LOC106684787	XP_024218583.1 (*Halyomorpha halys*)	E = 5e-170; bits = 486	83
DNA/RNA non-specific endonuclease isoform 10	280,771	Uncharacterized protein LOC106684787	XP_024218583.1 (*Halyomorpha halys*)	E = 7e-172; bits = 490	83
Small RNA degrading nuclease 1 (SDN1-like)	66,023	Uncharacterized exonuclease C637.09 isoform X1	XP_014279339.1 (*Halyomorpha halys*)	E = 0.0; bits = 895	75
Nibbler	743,764	Exonuclease mut-7 homolog	XP_024216394.1 (*Halyomorpha halys*)	E = 0.0; bits = 1402	84
**Antiviral**					
Ars2	149,588	Serrate RNA effector molecule homolog isoform X1	XP_014277995.1 (*Halyomorpha halys*)	E = 0.0; bits = 1523	98
NinaC	0.352	Neither inactivation nor after potential protein C	XP_014281724.1 (*Halyomorpha halys*)	E = 0.0; bits = 525	95
Beta 1,4-mannosyltransferase (egh)	262,137	Beta-1,4-mannosyltransferase egh	XP_014283435.1 (*Halyomorpha halys*)	E = 0.0; bits = 914	97
CG4572	420,293	Venom serine carboxypeptidase-like	XP_014280828.1 (*Halyomorpha halys*)	E = 0.0; bits=857	89
**Intracellular transport**					
Vacuolar H+ ATPase sub unit A (vha68)	0.437	V-type proton ATPase catalytic subunit A	XP_014272529.1 (*Halyomorpha halys*)	E = 0.0; bits=1250	99
Vacuolar H+ ATPase sub unit C (vha16)	63,065	V-type proton ATPase 16 kDa proteolipid subunit	XP_014275063.1 (*Halyomorpha halys*)	E = 4e-100; bits = 289	99
Small Rab GTPases	206,763	Ras-related protein Rab-7a	XP_014286452.1 (*Halyomorpha halys*)	E = 3e-152; bits = 425	99

#### Antiviral RNAi

The search for proteins related to the antiviral RNAi resulted in four protein sequences: Ars2, ninaC, a seven transmembrane-domain glycosyltransferase, Egghead (egh)^47^, and the CG4572 protein (Table 4, Supplementary data S5). The phylogenetic analyses revealed distinct clusters for the four antiviral RNAi proteins (Supplementary Fig. S4).

#### Intracellular transport

Three sequences related to intracellular transport were found: *Vacuolar H*+ *ATPase subunit A* (*vha68*), *Vacuolar H*+ *ATPase subunit C* (*vha16*) and the *Small Rab GTPases* (Table 4, Supplementary data S6).

### *Ex vivo* dsRNA hemolymph degradation

The dsRNA stability in the hemolymph was assessed at 0, 1, 10, 30, 60 and 120 min of incubation. After 10 min of incubation, the dsRNA-*V-ATP-A* was partially degraded, as the gel showed a smear below the band, clearly demonstrating dsRNA degradation (Supplementary Fig. S5). At 30 and 60 min of incubation we observed increased degradation, with all dsRNA degraded after 120 min incubation.

### Mortality of *E. heros* by dsRNA microinjection

**Figure 3:**
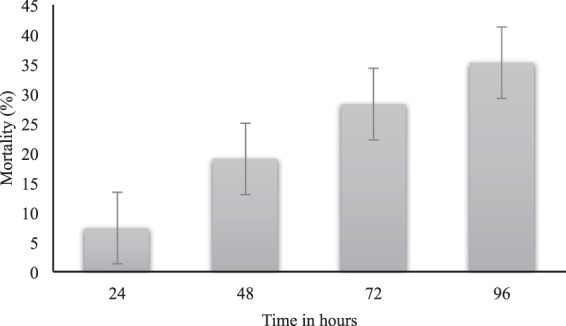
*V-ATPase subunit A* gene silencing mortality effects on *Euschistus heros*. Mortality after microinjection with dsRNA targeting *V-ATPase-A* (dsRNA-*V-ATP-A*) (24–96 h) expressed in percentage. Mortality in adults microinjected with dsRNA-*V-ATP-A* was normalized against the insects microinjected with dsRNA-*GFP*.The columns represent the mean ± SE. (N = 50).

Mortality was assessed at 24, 48, 72 and 96 h post-microinjection of dsRNA-*V-ATP-A* (Fig. 3). At 24 h, there was 7% mortality and this increased to 19% at 48 h, 28% at 72 h, and at 96 h 35% of the treated insects were killed. Alongside the mortality in dsRNA-*V-ATP-A* treated *E. heros*, reduced mobility was observed compared to the insects microinjected with dsRNA-*GFP*, which were very active. These mobility effects lasted until 72 h., with a recovery in the mobility at 96 h post microinjection.

### Gene expression of *V-ATPase-A*, and *DCR-2* and *AGO-2* in *E. heros*

**Figure 4:**
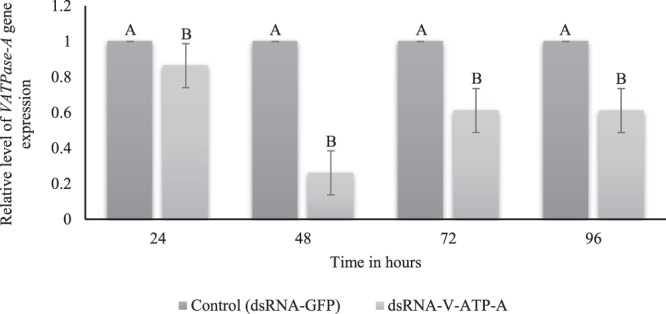
Effects of dsRNA targeting *V-ATPase subunit A* (dsRNA-*V-ATP-A*) on the relative levels of gene expression in *E. heros*. Four days old adults of *E. heros* microinjected with ~28 ng/µL per mg body weight. The adults were sampled at 24, 48, 72 and 96 h post-microinjection at both treatments. Gene expression was normalized against positive controls that were exposed to *gfp* dsRNA (dsRNA-*GFP*) (control). The bars represent the mean ± SE based on 3 biological repeats. The *p*-values were calculated by an unpaired t-test. Bars with different letters indicate that the treatments differed significantly at that time point with *p* ≤ 0.05 (N = 50).

The *V-ATPase-A* transcripts level gradually decreased following dsRNA treatment over time (14% to 74% from 24 h to 48 h, respectively) (Fig. 4). At 72 h and 96 h, there was an increase in the relative transcript levels, with ~40% reduction in gene expression, but despite this, these values were still significantly lower than the control (dsRNA-*GFP*-microinjected) insects (*p*-values <0.001 and 0.014, respectively) (Fig. 4).

**Figure 5:**
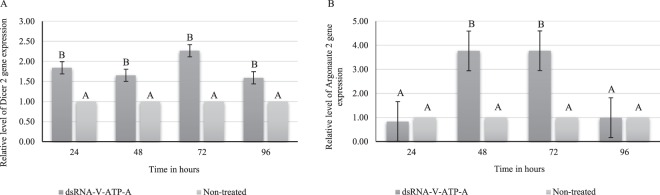
Effects of dsRNA targeting *V-ATPase subunit A* (dsRNA-*V-ATP-A*) on the relative levels of (**A**) *Dicer 2* (*DCR-2*) and (**B**) *Argonaute 2* (*AGO-2*) gene expression in *E. heros*. Four days old adults of *E. heros* were microinjected with ~28 ng/µL per mg body weight. The adults (12 in total) were sampled at 24, 48, 72 and 96 h post-microinjection. Gene expression was normalized against negative control that was not exposed to dsRNA. The bars represent the mean ± SE based on 3 biological repeats. The *p*-values were calculated by an unpaired t-test. Bars with different letters indicate that the treatments differed significantly at that time point with *p* ≤ 0.05 (N = 50).

The involvement of the siRNA machinery in the gene silencing mechanism was assessed through a qRT-PCR analysis of *DCR-2* and *AGO-2* genes (Fig. 5). The relative transcript levels of *DCR-2* were significantly higher in the insects microinjected with dsRNA-*V-ATP-A* compared to the controls (not exposed to dsRNA), with the highest *DCR-2* expression level observed at 72 h post microinjection and with an increase of ~2.0-fold (Fig. 5A). At 96 h, relative transcript levels of *DCR-2* dropped to ~1.5-fold, still higher than the controls. The expression pattern of *AGO-2* behaved similarly as we saw for *DCR-2* (Fig. 5B). At 48 and 72 h post-microinjection, the relative transcript levels of *AGO-2* were higher with almost a 4.0-fold increase compared to the control samples.

## Discussion

The Neotropical stinkbug *E. heros* is one of the most important soybean pests in Brazil, Argentina, and Paraguay, and the current lack of genetic information is among the factors limiting the prospects of RNAi as an alternative control approach.

The RNAi pathway works primarily through dsRNA uptake, intracellular dsRNA transport, dsRNA processing to sRNA, RISC complex formation and binding, and digestion/repression of the target mRNA^48^. Based on our currently reported *E. heros* transcriptome database, most of the genes involved in these processes above and related to RNAi pathways, are also present in the *E. heros* transcriptome (Tables 1–4). However, it is important to note that although these genes are involved in the RNAi process in other organisms, it does not mean that they play the same role in the RNAi mechanism in *E. heros*, and the real involvement of these genes needs to be further confirmed in future functional assays.

To achieve gene silencing through RNAi, dsRNA is taken up by the tissue/cell. In eukaryote organisms, this process occurs through sid-like transmembrane proteins^25,49^ or endocytosis-mediated uptake^24,25^. Before Sid-like homologous proteins have been found in Coleoptera, Hymenoptera, Lepidoptera, and Hemiptera, but not in Diptera^49^. Also in the *E. heros* transcriptome, *sid-like* homolog genes were not found. In *Drosophila* (Diptera: Drosophilidae), with the lack of *sid-like* homolog genes, the dsRNA uptake occurs via endocytosis-involving scavenger receptors^23,24^. Indeed previous work demonstrated that the Scavenger protein is involved in endocytic dsRNA uptake in insects^23,24,50^ and other organisms, such as mites^19,51,52^. The Chc protein, which is related to an alternative mechanism for endocytic dsRNA uptake in insects^23–26^, was found in the *E. heros* transcriptome. Consequently, with the absence of *sid-like* genes in the *E. heros* transcriptome, we believe that the Chc protein may be involved in cellular uptake in *E. heros*; however the involvement of this protein in dsRNA uptake needs to be proven in future functional assays. In addition, future experiments need to investigate the importance of endocytosis in *E. heros*.

Core RNAi machinery genes were also searched for in the *E. heros* transcriptome with focus on the miRNA, siRNA and piRNA pathways (Table 2), and most of these were present with the absence of a *RNA-dependent RNA polymerase* (*RdRP*) gene. The lack of *RdRP* was generally expected because, so far, it has been reported only in ticks, plants and in the nematode *Caenorhabditis elegans* (Rhabditida: Rhabditidae)^53^. The main core domains of Dicer are well known due to their involvement in dsRNA cleavage into small RNA molecules (sRNAs), including miRNAs and siRNAs. In the current work, the DCR-1 protein, which is related to the miRNA pathway, contains a PAZ domain, two RNaseIII domains, and a DSRBD, however no conserved domains for helicase were identified. For DCR-2, two isoforms with distinct structures and abundances were identified. The DCR-2 isoform 1 was the most abundant and showed a helicase domain, a PAZ domain, two RNaseIII domains, and a DSRBD^54,55^. The PAZ domain holds a binding pocket for the 3′ overhang of dsRNA substrate and a phosphate-binding pocket that recognizes the phosphorylated 5′ end of small RNAs^56,57^. The two RNaseIII domains are the catalytic core components of Dicer and responsible for the cleavage of the dsRNA substrate^58^. The function of the helicase domain remains unclear, but so far it is known that this domain is required to process siRNA but not miRNA^56^. In flies, the loss in the functionality of DEAD/Helicase domain is related to a particular function in the miRNA-based gene regulation^56^. We hypothesize here that the loss of the helicase domain in the DCR-1 protein in *E. heros* may be a functional adaptation, related to the miRNA pathway, but this needs to be further investigated. The canonical conserved domains of DCR-2 isoform were not identified in *E. heros*. Similarly, DCR-2 isoforms with the lack of conserved domains were also identified in mammals^59,60^ as well as in *Arabidopsis thaliana*^61^. Due to the lack of important functional domains it is expected that these DCR-2 variants may not be involved in the siRNA pathway, however the function of these isoforms in insects still remains unclear. To our knowledge, this is the first report of DCR-2 variants in insects. It would be interesting in the future to investigate the role of DCR variants in cellular processes.

In other insects such as *Cylas puncticollis* (Coleoptera: Curculionidae)*, N. lugens*, *D. v. virgifera*, *L. decemlineata*, *Drosophila* and *Tribolium*, *DCR-1* and *DCR-2* are also present^31,48,62,63^. In *Drosophila* the involvement of *DCR-1* and *DCR-2* is well established in the miRNA and siRNA pathways^62^. In the piRNA pathway, there is no evidence of a dsRNA precursor and the need of DCR endonucleases^64–66^. Drosha protein was identified with two RNaseIII domains plus a RIBOc^54^ and with some similar features to Dicer, although it processes miRNA precursors in the nucleus^17^. The dsRNA-binding proteins Pasha, Loquacious and R2D2, which mediate dsRNA binding to the RISC complex, are among the other proteins from the DCR superfamily identified in *E. heros*. These proteins are cofactors required to interact with the RNaseIII genes *Drosha*,* DCR-1*, and *DCR-2*, respectively^31,62^ (Table 2).

Five members belonging to the Argonaute superfamily were identified in the *E. heros* transcriptome as follows: AGO-1 and AGO-2 which belong to Argonaute subfamily, and AGO-3, Aubergine (Aub) and Piwi, which belong to the PIWI subfamily^67,68^. AGO-1 is an essential protein related to the miRNA pathway, and AGO-2 is related to the siRNA pathway^68,69^. More recently, two new functions have been attributed to AGO-1 and AGO-2 in early *Drosophila melanogaster* embryos: the generation of polarity within cells and tissues by modulating an important cell-cell signaling pathway^70^. These proteins are characterized by the presence of PAZ and PIWI domains, which guide sRNA recognition and binding, supporting endonucleolytic cleavage^71^. The PAZ domain forms a pocket for siRNAs binding and, specifically, the characteristic two nucleotides (nt) 3′ overhangs, trimmed by Dicer proteins, while the PIWI domain shares structural similarities with ribonucleases and degrades the corresponding RNAs^72–74^. The lack of a PAZ functional domain in the AGO-2 isoform 2 raises the hypothesis that this isoform may be related in another biological process, as mentioned above for the DCR-2 isoforms. In the shrimp *Marsupenaeus japonicus* (Decapoda: Penaeidae), three AGO-1 isoforms have been identified, and interestingly, two isoforms were more expressed in the lymphoid organ, suggesting a role in immunity^75^. The presence of multiple isoforms of AGO-1 and AGO-2 may indicate a role of AGO in many biological processes, including cell proliferation/differentiation, immune defense, among others^75^. AGO-3, Aub, and Piwi are proteins related to the piRNAs pathway^65,68^ and they were also found in *E. heros* (Table 2). Zucchini (Zuc), responsible for piRNA maturation^76^ and related to the germline RNAi processes^77^, was also identified in the *E. heros* transcriptome.

The identification of both DCR-1 and DCR-2 was confirmed through a phylogenetic analysis using sequences from other insects and revealed distinct groups inside DCR proteins (Supplementary Fig. S1). The *E. heros* protein DCR-1 was grouped in a clade with DCR-1 proteins from *N. viridula* and *H. halys*, showing a common ancestor. The same results were found for *E. heros* DCR-2. Also, *E. heros* DCR-1 was grouped in a distinct clade compared to *E. heros* DCR-2, but with a common ancestor. The phylogenetic analysis for the AGO superfamily resulted in two main clades; one containing the AGO subfamily proteins AGO-1 and AGO-2, and another with the PIWI subfamily proteins AGO-3, Aub and Piwi (Supplementary Fig. S2). *E. heros* AGO-1 clustered with AGO-1 from *N. viridula* and *N. lugens* with the same ancestor. *E. heros* AGO-2 was assembled in a second group together with other proteins of this family. These two clusters showed a common ancestor. The AGO-3 was clustered in a distinct group, as well as Aub and Piwi proteins. *E. heros* AGO-3 was grouped with the AGO-3 proteins from *H. halys* and other insects. Thereby, the phylogenetic analyses were useful to confirm the identification of the core RNAi genes present in the *E. heros* transcriptome.

AGO protein is the core component of the RISC, and guided by the siRNA it promotes mRNA cleavage^72,73^. Next to *AGO*, other important genes related to RISC were identified in the *E. heros* transcriptome (Table 3). Tudor-SN (TSN) protein is known to interact with Argonaute proteins in the silkworm *Bombyx mori* (Lepidoptera: Bombycidae)^78^, while Translin and TRAX, that are conserved subunits of the C3PO, are involved in RISC activation, supporting RNAi activity^79^. The *Armi*, *Spn-E*, *Maelstrom* and *Hen-1* nucleases are involved in piRNA biogenesis^31^. *Maelstrom* mutations in *Drosophila* ovaries resulted in a depletion of Dicer and AGO-2 proteins, the latter two being related to the RNAi pathways^80^. Elp-1, that is also present in *E. heros*, is a component of the polymerase II elongator complex, and although the absence of this protein in *Drosophila* S2 cell lines did not affect the miRNA pathway, it can cause an inhibition of the siRNA pathway^81^. The *Vasa intronic gene* (*VIG*), that encodes a putative RNA-binding protein through association with RISC^82^, and related to the production of piRNAs^83^, was also identified in the *E. heros* transcriptome. The Gawky protein, a cytoplasmic mRNA component necessary in early embryonic development^84^, Staufen (STAU), a DSRBP, and CLIP-associating protein (Clp-1), that is responsible for the phosphorylation of the 5′ end of siRNAs^85^ and related to the splicing process of transfer RNAs^86^, were all also found in the *E. heros* transcriptome of this study. The PRP16 protein plays a role in the pre-mRNA processing^87^, while Belle has a function in the endo-siRNA pathway^88^. The proteins GLD-1 and ACO-1, known to inhibit translation of mRNA into protein^81^, were also identified in *E. heros*.

Nucleases (RNases together with other RNA enzymes) function in DNA/RNA digestion in the midgut^89^ and offer an additional defense and regulatory control layer. The activity of nucleases in dsRNA degradation (dsRNases) is well known, taken an important role in RNAi efficiency across insect groups such as Hemiptera^39,41,90^, Lepidoptera^91,92^, and Diptera^38^. Four nucleases were identified in the *E. heros* transcriptome: *Eri-1*, *Nibbler*, *SDN1*, and *dsRNase* (Table 4; Supplementary Fig. S3). The Eri-1 nuclease is suggested to play a role in the intracellular siRNA and miRNA pathways^93^. In *C. elegans*, Eri-1 forms a complex with Dicer, generating specific classes of siRNAs, while in mouse, Eri-1 negatively regulates the global abundance of miRNA^93^. Nibbler, an exonuclease known to be involved in shaping the 3′end of the miRNAs, and its depletion leading to developmental defects in *Drosophila*^94^, was found with conserved domains in *E. heros*. Another intracellular nuclease found in *E. heros* was SDN1. In *Arabidopsis*, this protein is involved in the degradation of mature miRNA, and the knockdown resulted in developmental defects^94^. However, the involvement of the Eri-1, Nibbler, and SDN1 in RNAi efficiency in insects remains unclear. In *E. heros* we also identified a *dsRNase* gene with seven isoforms and with a conserved Endonuclease_NS domain associated with the degradation of foreign dsRNA molecules^38^. In *B. mori* three dsRNases isoforms were identified and expressed in different tissues, such as epidermis, fat body, and gut; these dsRNases are related to the innate immune response against invasive nucleic acids^92^. The presence of a *dsRNase* nuclease with seven isoforms may indicate that *E. heros* has a strong nuclease activity, so this may result in a lower potential to suppress the expression of target genes and so in turn a lower RNAi response.

In the current work, we identified some genes related to antiviral RNAi as follows: *Ars2*, a gene related to RISC regulation, *ninaC*, a gene associated with vesicle transport, and a seven transmembrane-domain glycosyltransferase, *egh*^47^ (Table 4; Supplementary Fig. S4). These genes are known to be involved in antiviral defense in *Drosophila*^47,95^. The *CG4572* gene was also identified in *E. heros*; it is a carboxypeptidase with unknown function, but related to RNAi in *D. melanogaster*^47^. Three genes involved in intracellular transport were also identified. Two *vacuolar H*+ *adenosine triphosphatases* (*V-ATPases*) genes were identified in the *E. heros* transcriptome: *V-ATPase subunit A* (*vha68*) and *V-ATPase subunit C* (*vha16*). These genes are located at different functional V-ATPase domains, the peripheral domain (V1) and the integral domain (V0)^96^, respectively, and they are related to dsRNA release by the endocytic vesicles^23^. The Small Rab GTPases and vha68 are essential signaling components linked to the extracellular part with the cytoplasm in *L. decemlineata*^25,48^.

The presence of some genes in *E. heros* suggests that it has an active and functional RNAi machinery. However, the lack of *sid-like* gene and the presence of nuclease raise the concern about the RNAi efficiency. So, we first checked the stability of a dsRNA molecule in the hemolymph of adults in which it was rapidly degraded. After 10 min, the dsRNA-*V-ATP-A* was partially degraded, with increasing dsRNA degradation over time up to 120 min (Supplementary Fig. S5B). In a similar experiment with the pea aphid *A. pisum*, dsRNA was completely degraded after 3 h incubation and this was associated with the lack of RNAi responses in this species^41^. In *E. heros*, dsRNA was completely degraded after 2 h of incubation with watery saliva^97^. Indeed, high nuclease activity in the hemolymph and saliva of *E. heros* may reduce RNAi efficiency and so some form of dsRNA protection may be needed for future field applications.

To confirm the effectivity of the *E. heros* RNAi machinery, a dsRNA targeting the *V-ATPase-A* gene was microinjected into adults. Previously, targeting the *V-ATPase-A* gene led to mortality in *Pectinophora gossypiella* (Lepidoptera: Gelechiidae)^98^, *E. heros* nymphs^97^, *A. pisum*^99^, *H. halys* nymphs^42^, among others. The main V-ATPase function is the pumping of protons across the membrane^100,101^, generating an energy gradient. *E. heros* adults were microinjected with ~28 ng of dsRNA-*V-ATPase-A* per mg of insect fresh weight, and the mortality was evaluated at 24, 48, 72 and 96 h after microinjection. At 24 h post-microinjection, there was 7% mortality and this increased to 35% at 96 h (Fig. 3). The same dsRNA concentration previously demonstrated to cause up to 50% mortality in *E. heros* 2^nd^ instar nymphs 7 days post-microinjection^97^. Based on these results, we believe that this species is sensitive to RNAi when we compare to other insects also considered sensitive to RNAi. Fishilevich and collaborators^102^ used the same dsRNA concentration through microinjection in *E. heros* adults targeting chromatin remodeling genes and this significantly reduced fecundity and egg viability. Coleoptera insects are considered to be more sensitive to RNAi, presenting a robust RNAi mechanism, while Lepidoptera and Hemiptera appear to be more recalcitrant^103^. Second-instar larvae of the African sweet potato weevil *C. puncticollis* were microinjected with 200 ng/mg of body weight targeting different genes, and mortality reached up to ~50% after six days^104^; this concentration is ~9 times higher than that one used in *E. heros*. One of the main reasons associated with the lack of RNAi response in the *C. puncticollis* weevil was the high nuclease activity^104,105^. In the pink bollworm, *P. gossypiella*, microinjection of 20 ng/mg of body weight of a dsRNA targeting *V-ATPase-A* induced mortality up 26% at 96 h post-microinjection in 3^rd^-instar larvae^98^. One strategy to increase RNAi efficiency is an adequate formulation of the dsRNA molecules. In *E. heros* nymphs, liposome-encapsulated dsRNA targeting *V-ATPase-A* led to 45% mortality after 14 days as compared to 30% with naked dsRNA^97^. Similar results were found for dsRNA α-tubulin and lipoplexes in the German cockroach, *Blattella germanica* (Blattodea: Blattellidae)^106^. Therefore, the formulation of dsRNA may provide an affordable non-transformative easy-to-use strategy to deliver gene silencing for pest control in the field. However, for successful pest control, it is very important to know the dsRNA concentration, expressed as per mg of insect body weight, to permit a rationalized pest control strategy based on dsRNA concentration and the delivery approach.

Alongside the mortality, other effects were also observed. The treated insects exhibited reduced mobility in contrast to the insects microinjected with dsRNA-*GFP* which were very active. This effect lasted until 72 h post-microinjection. Retardation in larval development was reported in *P. gossypiella*^98^ and *Helicoverpa armigera* (Lepidoptera: Noctuidae)^107^ treated with dsRNA targeting the *V-ATPase*.

To confirm that the observed mortality in *E. heros* injected with dsRNA-*V-ATPase-A* is a true phenotype of gene silencing, a qRT-PCR assay was performed. Indeed we confirmed the *V-ATPase-A* gene silencing with a reduction of 74% in the relative level of transcripts. At 72 and 96 h post-microinjection, there is an increase in gene expression but still 40% lower compared to the insects treated with *GFP* (Fig. 4). An increase in *DCR-2* and *AGO-2* gene expression was observed with the highest gene expression observed at 48 and 72 h, with a respective increase of ~2.0 and ~4.0-fold, so confirming the activation of the siRNA machinery upon exogenous dsRNA delivery (Fig. 5A,B). This data has shown the activity of the siRNA machinery in *E. heros* through the supply of dsRNA. As expected, due to the high nuclease activity, the RNAi effects were temporary, and at 72 and 96 h, there was a recovery in the relative transcript levels from the target genes. Also, at 96 h post-microinjection, there was a reduction in the expression of the siRNA-related genes. In *Manduca sexta* (Lepidoptera: Sphingidae), an upregulation of *DCR-2* and *AGO-2* expression in response to injection with dsRNA with also only transient effects in the gene upregulation was reported^108^. As discussed above*, DCR-2* and *AGO-2* are core components of the siRNA pathway, and the overexpression of *DCR-2* and *AGO-2* after dsRNA microinjection confirmed the upregulation of the siRNA machinery.

Over the past year, scientists have made enormous progress towards the use of RNAi as a pest control strategy taking advantage of genetic sequences available in public databases, and used this information to understand the RNAi mechanism in insects. To our knowledge, this is the first study of *E. heros* transcriptome, including the identification of RNAi-related genes and dissecting the siRNA pathway. The analyses of the *E. heros* transcriptome have identified the main components of the three RNAi pathways with the surprising lack of *sid-like* genes. Identification of the core RNAi genes, efficient mortality rates and activation of the siRNA machinery, these data provide a novel and important dataset on RNAi machinery and its efficiency, underpinning future strategies to enhance RNAi in *E. heros* and potentially other piercing-sucking insects as models or species important in agriculture.

## Material and Methods

### Brown stink bug insects

The colony of *E. heros* was originally started with insects collected in Pelotas, Brazil (27°48′1.7352″ S; 52°54′3.834″W) in 2013, and kept for about 73 generations under laboratory conditions before experiments. New insects collected in soybean fields in Rondinha, Brazil (27°48′1.7352″ S; 52°54′3.834″ W) were introduced in the laboratory colony in 2015. All stages were maintained in plastic cages under laboratory conditions with a photoperiod of 14:10 (Light: Dark), temperature of 25 ± 1 °C and 75 ± 10% relative humidity. Green beans, peanut and water were supplied *ad libitum* and replaced twice in a week. Eggs were collected twice a week to obtain the insects necessary for microinjection and colony maintenance^109^. Insects were collected every day and insects of four days old were used in the microinjection assays.

### cDNA libraries, Illumina sequencing and *de novo* assembly

Eggs, all nymphal stages and adults of *E. heros* were used for total RNA extraction using the Trizol reagent (Invitrogen, Carlsbad, CA, USA), according to the manufacturer instructions. The RNA pool was prepared with an equally RNA amount from all stages, and the cDNA library preparation and Illumina sequencing were conducted at the Laboratory of Functional Genomics Applied to Agriculture and Agri-Energy, at the University of São Paulo, Brazil. The TruSeq RNA Sample Prep kit (Illumina) protocol was used to construct the cDNA library, following manufacturer instructions. A high-throughput Illumina sequencing platform (HiSeq. 2000) was used for the final library sequencing, in one lane of a 100 bp paired-end run.

The raw reads originating from the Illumina sequencing were check for quality using the FastQC software (http://www.bioinformatics.babraham.ac.uk/projects/fastqc). After that, reads were trimmed using Trimmomatic^110^, and only high-quality reads, showing a Phred score superior to 30, were used for the *de novo* assembly to generate a set of contigs using Trinity software (http://trinityrnaseq.sourceforge.net)^111^. De Bruijn graph algorithm and a k-mer length of 25 were used as parameters.

### Homology search and gene ontology annotation

The generated contigs were analyzed using the UniProt-TrEMBL database^112^ via Diamond algorithm^113^, with an E-value<10^−5^ as a cut-off parameter. The contigs with insect hits were submitted to a second homology search using QuickGO to identify gene ontology (GO) terms. For this annotation, a similarity search was performed against the UniProt database using Diamond, with an E-value<10^−5^ as a cut-off parameter. The QuickGo from EBI (https://www.ebi.ac.uk/QuickGO/annotations) was used to calculate the GO terms, using the generated gene identifiers as inputs.

### RNAi-related genes

We searched for the genes related to RNAi efficacy and these included genes on dsRNA uptake (Table 1), RNAi core machinery (Table 2), auxiliary factors (Table 3), nucleases, antiviral RNAi and intracellular transport (Table 4)^31,33,48^. Homologous sequences for these proteins were searched in *UniProt* or Protein database from NCBI, and we used as a query to search the *E. heros* transcriptome using the tBLASTn tool from NCBI. Generated contigs with a bitscore>150 and E-value <1e-5 were further used to confirm the identity. To detect the open reading frames (ORFs) in the contigs sequence, the ORF Finder from NCBI was used (https://www.ncbi.nlm.nih.gov/orffinder/), and the protein domains predicted by the NCBI Conserved Domains Database (https://www.ncbi.nlm.nih.gov/Structure/cdd/wrpsb.cgi). Protein Basic Local Alignment Tool (Protein BLAST) was used for protein homology search against insect non-redundant protein database at NCBI.

To provide additional confirmation on identity and function prediction of the core RNAi proteins, nucleases and antiviral RNAi, members of these groups of proteins were subject to a phylogenetic analysis using the neighbor-joining (MEGA 7.0.26) algorithm with 1,000 bootstrap replicates. A total of 39 Argonaute superfamily protein sequences, 30 endoribonuclease III protein sequences, 25 nuclease protein sequences, and 27 antiviral RNAi sequences were aligned using the MUSCLE program from MEGA 7.0.26 software. ORF Finder from NCBI was used to predict the proteins.

### dsRNA synthesis and purification

Specific primers were used to amplify the fragments of the target genes (Table 5). The cDNA was synthesized using the SuperScript First-Strand Synthesis System Kit (Invitrogen) following the manufacturer’s instructions. The T7 primer sequence (TAATACGACTCACTATAGGGAGA) was placed in the front of the forward and reverse primers. These primers were used for dsRNA synthesis with cDNA as a template. The PCR reaction was performed with 2 µl of cDNA template, 2 µl of a 10 µM solution of each primer (Integrated DNA Technologies, Coralville, IA, USA), 0.125 µl of Taq DNA polymerase, 2.5 µl of Buffer 10×X, 0.5 µl of 10 µM dNTPs, 0.75 µl of MgCl_2_ (Invitrogen) and 15.125 µl of nuclease-free water (GE Healthcare, Little Chalfont, UK) in a total volume of 25 µl. The PCR conditions used were 5 min at 94 °C for initial denaturation, followed by 30 s at 94 °C, 45 s at 59.5 °C, 55 s at 72 °C for 30 cycles and final extension for 10 min at 72 °C. The amplified products were purified using a PCR purification kit (Qiagen, Valencia, CA, USA) and analyzed on 1% agarose gels. The PCR product was quantified using a Nanovue spectrophotometer (GE Healthcare) and then samples were stored at −20 °C.

**Table 5:** Primers used in qRT-PCR and dsRNA synthesis.

	Gene name	Primer	Sequence (5′-3′)	Product size (bp)	Amplification factor	R²
qRT-PCR	*18 s ribosomal RN*A	rp*18Sr*RNA-F	TACAACAAGACAACGCTCGC	150	2.07	0.997
		rp*18Sr*RNA-R	TTGCGCTCAGTGACATCTCT			
	*Ribosomal protein L32e*	rp*rpl32*-F	TCAGTTCTGAGGCGTGCAT	175	2.15	0.992
		rpr*pl32*-R	TCCGCAAAGTCCTCGTTCA			
	*V-ATPase subunit A*	rpdsRNA-*V-ATP-A*-F	GATTATGGTCGTGCGATTTC	102	1.93	0.998
		rpdsRNA-*V-ATP-A*-F	GAACACCAGCTCTCACTAA			
	*Dicer 2*	rpDCR2-F	GAAGCAGGATAACCTCCTAA	156	1.94	1
		rp*DCR2*-R	GGATGCAATTGTTCTACTGGA			
	*Argonaute 2*	rp*AGO2*-F	GACCATCTCCACAACAAATG	113	1.97	0.994
		rp*AGO2*-R	GTCAGAGGATTGAGGTCTAATA			
dsRNA synthesis	*V-ATPase subunit A*	dsRNA-*V-ATP-A*-F	TAATACGACTCACTATAGGGAGACAGGTTTCGACCAATGCCAA	623	—	—
		dsRNA*-V-ATP-A-*R	TAATACGACTCACTATAGGGAGAACCTCAGAACACCAGCTCTC			
	*Green Fluorescent Protein*	dsRNA-*GFP*-F	TAATACGACTCACTATAGGGAGATCGTGACCACCCTGACCTAC	560	—	—
		dsRNA-*GFP*-R	TAATACGACTCACTATAGGGAGATCGTCCATGCCGAGAGTGAT			

The *V-ATPase-A* dsRNA was synthesized using the MEGAscript T7 RNAi kit (Ambion, Austin, TX, USA) following the manufacturer’s instructions. The control group consisted of a dsRNA of the *green fluorescent protein* (dsRNA-*GFP*) synthesized from a DNA plasmid (pIG1783f) and cloned in *Escherichia coli* (DH5α). Plasmid DNA was extracted and sequenced to confirm the identity of PCR products. The identity of the sequence was confirmed by Sanger sequencing. The dsRNA was analyzed for integrity on 1% agarose gels, its concentration quantified in a Nanovue spectrophotometer (GE Healthcare) and then stored at −20 °C.

### *Ex vivo* dsRNA hemolymph degradation assay

Insects were anesthetized with CO_2_ during ~30 s and then taped with the abdomen upwards on a glass plate. Legs and rostrum were cut, and hemolymph collected by a needle, prepared with glass capillary tubes, coupled to an insulin syringe (8 ×X 0.30 mm) and placed in chilled 1.5 ml tubes containing phenylthiourea (PTU) to prevent melanisation. After that, 30 µl of dsRNAs-*V-ATPase-A* solution at 200 ng/µl was incubated in 3 µl of RNase-free water or 3 µl of hemolymph at 25 °C. Aliquots of 5 µl were collected after 0, 1, 10, 30, 60, and 120 min, and the same volume of EDTA (10 mM) was added to the solution to stop the enzymatic reaction^41^. The integrity of the samples was analyzed on 1% agarose gel.

### Adult microinjection

To silence the *V-ATPase-A* gene in *E. heros*, dsRNA-*V-ATP-A* with 623 bp was microinjected in adults (~60 mg) at the concentration of ~28 ng per mg of body weight (0.50 µl of a 3350 ng/µl dsRNA solution)^102^. The control group consisted of insects microinjected with a 560 bp dsRNA molecule targeting *GFP*^31,104^. The dsRNA-*V-ATP-A* was designed to have a length similar to the one used in previous RNAi assays in the hemipterans *D. citri*^43^ and *N. lugens*^114^.

To perform the microinjection, insects were anesthetized with CO_2_ and immobilized in a glass plate with double-sided tape (3 M, São Paulo, Brazil). The microinjection was performed using an insulin syringe (8 ×x 0.30 mm) with a needle (30 g) (Solidor) coupled to a micro-applicator (Burkard, Rickmansworth, UK). In total, 62 adults were injected per treatment, of which 12 individuals were used for qRT-PCR and 50 individuals for mortality assay, at 24, 48, 72 and 96 h post-microinjection. Alongside mortality analysis, visual observations were carried out to analyze other effects related to the dsRNA in the insects. After microinjection, the insects were placed in plastic cages containing green beans, peanut and water *ad libitum*, and kept at 25 °C, photoperiod of 14:10 (L:D) and 75 ± 10% RH, as with the colony maintenance. The insect mortality was normalized against the control (dsRNA-*GFP*).

### Quantitative reverse-transcription polymerase chain reaction (qRT-PCR)

Total RNA was extracted from whole insect body at 24, 48, 72 and 96 h after microinjection, and each time point had three biological samples containing one insect. RNA extraction was performed using RNAzol RT (MCR, Cincinnati, OH, USA), following the manufacturer’s instructions. The samples were quantified using a Nanovue spectrophotometer (GE Healthcare), verified in a 1% agarose gel electrophoresis, and kept at −80 °C. First-strand cDNA synthesis proceeded as described in the dsRNA synthesis and purification section.

The qRT-PCR was performed on a Roche LightCycler 480 (LC480) (Roche Diagnostics, Basel, Switzerland) real-time PCR platform. To validate the primers used in the analysis (Table 5), a melting curve analysis with temperatures from 60 to 95 °C and a standard curve based on a serial dilution of cDNA were used to determine the primer annealing efficiency and specificity. The reaction included 6 µl of EvaGreen 2X qPCR MasterMix (ABM, Milton, ON, Canada), 1.25 µl of 10 µM forward primer (Integrated DNA Technologies), 1.25 µl of 10 µM reverse primer (Integrated DNA Technologies), 2.5 µl of nuclease-free water and 2 µl of cDNA, in a total volume of 13 µl. The amplification conditions were 3 min at 95 °C followed by 45 cycles of 30 s at 95 °C, 45 s at 59 °C and 30 s at 77 °C. The reactions were set-up in 96-well microtiter plates (Roche Life Science, Indianapolis, IN, USA), using the cDNA dilution of 1:25 and three technical replicates. The normalization of the data was performed using two endogenous genes, *ribosomal protein L32e* (*rpl32*) and *18 s ribosomal RNA* (*18S rRNA*) (Table 5); also an appropriate no template control (NTC) was included. The equation ratio 2^−∆∆Ct^ was used for normalization of the relative gene expression levels^115^. Data were analyzed using analysis of variance (one-way ANOVA) and unpaired *t*-test (*p*-value ≤ 0.05).

### Ethical approval

This article does not contain any studies with human participants or vertebrate performed by any of the authors

## Supplementary information

Supplementary material.

## References

[1] Medeiros L, Amegier GA (2009). Ocorrência e Desempenho de *Euschistus heros* (F.) (Heteroptera: Pentatomidae) em Plantas Hospedeiras Alternativas no Rio Grande do Sul. Neotrop. Entomol..

[2] Panizzi ARSB (2015). Growing Problems with (Hemiptera: Heteroptera: Pentatomidae): Species Invasive to the U.S. and Potential. Am. Entomol..

[3] Panizzi, A. R., Bueno, A. F. & Silva, F. A. C. Insetos que atacam vagens e grãos. Hoffmann-Campo, C. B., Corrêa-Ferreira, B. S. & Moscardi, F. (ed.) *Soja: manejo integrado de insetos e outros artrópodes-praga*. **5**, 335–420. (Embrapa, 2012).

[4] Panizzi, A. R. *et al*. Insetos da Soja no Brasil. *Bol. Técnico n° 1* https://ainfo.cnptia.embrapa.br/digital/bitstream/item/77369/1/CNPSO-BOL.-TEC.-1-77.pdf (1977).

[5] Sosa-Gómez DR (2009). Insecticide susceptibility of *Euschistus heros* (Heteroptera: Pentatomidae) in Brazil. J. Econ. Entomol..

[6] Saluso A, Xavier L, Silva FAC, Panizzi AR (2011). An invasive pentatomid pest in Argentina: Neotropical brown stink bug, *Euschistus heros (*F.) (Hemiptera: Pentatomidae). Neotrop. Entomol..

[7] Sosa-Gómez DR, Silva JJda (2010). Neotropical brown stink bug (*Euschistus heros*) resistance to methamidophos in Paraná, Brazil. Pesqui. Agropecuária Bras..

[8] Guedes RNC (2017). Insecticide resistance, control failure likelihood and the First Law of Geography. Pest Manag. Sci..

[9] Tuelher ES (2018). Area-wide spatial survey of the likelihood of insecticide control failure in the neotropical brown stink bug *Euschistus heros*. J. Pest Sci..

[10] Sagan SM, Sarnow P (2014). RNAi, antiviral after all. Science..

[11] Gammon DB, Mello CC (2015). RNA interference-mediated antiviral defense in insects. Curr. Opin. Insect Sci..

[12] Baum JA (2007). Control of coleopteran insect pests through RNA interference. Nat. Biotechnol..

[13] Head GP (2017). Evaluation of SmartStax and SmartStax PRO maize against western corn rootworm and northern corn rootworm: Efficacy and resistance management evaluation. Pest Manag. Sci..

[14] Cagliari, D., Santos, E. A. dos, Dias, N., Smagghe, G. & Zotti, M. Nontransformative strategies for RNAi in crop protection. Singh, A. (ed.). *Modulating Gene Expression - Abridging the RNAi and CRISPR-Cas9 Technologies* **2**, 1–17 (2018).

[15] Palli SR (2014). RNA interference in Colorado potato beetle: steps toward development of dsRNA as a commercial insecticide. Curr. Opin. Insect Sci..

[16] San Miguel K, Scott JG (2016). The next generation of insecticides: DsRNA is stable as a foliar-applied insecticide. Pest Manag. Sci..

[17] Carthew RWSJE (2009). Origins and Mechanisms of miRNAs and siRNAs. Natl. Institutes Heal..

[18] Weick E-M, Miska E (2014). A. piRNAs: from biogenesis to function. Development.

[19] Huvenne H, Smagghe G (2010). Mechanisms of dsRNA uptake in insects and potential of RNAi for pest control: A review. J. Insect Physiol..

[20] Feinberg EH, Hunter CP (2003). Transport of dsRNA into cells by the transmembrane protein SID-1. Science.

[21] Aronstein K, Pankiw T, Saldivar E (2006). SID-I is implicated in systemic gene silencing in the honey bee. J. Apic. Res..

[22] Kobayashi I (2012). SID-1 protein of *Caenorhabditis elegans* mediates uptake of dsRNA into *Bombyx* cells. Insect Biochem. Mol. Biol..

[23] Saleh M (2006). The endocytic pathway mediates cell entry of dsRNA to induce RNAi silencing. Nat. Cell Biol..

[24] Ulvila J (2006). Double-stranded RNA is internalized by scavenger receptor-mediated endocytosis in *Drosophila* S2 cells. J. Biol. Chem..

[25] Cappelle K, De Oliveira CFR, Van Eynde B, Christiaens O, Smagghe G (2016). The involvement of clathrin-mediated endocytosis and two Sid-1-like transmembrane proteins in double-stranded RNA uptake in the Colorado potato beetle midgut. Insect Mol. Biol..

[26] Pinheiro DH (2018). Clathrin-dependent endocytosis is associated with RNAi response in the western corn rootworm, *Diabrotica virgifera virgifera* LeConte. PLoS One.

[27] Vélez AM, Fishilevich E (2018). The mysteries of insect RNAi: A focus on dsRNA uptake and transport. Pestic. Biochem. Physiol..

[28] Whangbo JS, Hunter CP (2008). Environmental RNA interference. Trends Genet..

[29] Zotti MJ, Smagghe G (2015). RNAi technology for insect management and protection of beneficial insects from diseases: Lessons, challenges and risk assessments. Neotrop. Entomol..

[30] Ketting RF (2011). The Many Faces of RNAi. Developmental Cell.

[31] Prentice K (2015). Transcriptome analysis and systemic RNAi response in the African sweetpotato weevil (*Cylas puncticollis*, Coleoptera, Brentidae). PLoS One.

[32] Firmino AAP (2013). Transcriptome analysis in cotton boll weevil (*Anthonomus grandis*) and RNA interference in insect pests. PLoS One.

[33] Swevers L (2013). Colorado potato beetle (Coleoptera) gut transcriptome analysis: Expression of RNA interference-related genes. Insect Mol. Biol..

[34] Camargo R (2015). *De novo* transcriptome assembly and analysis to identify potential gene targets for RNAi-mediated control of the tomato leafminer (*Tuta absoluta*). BMC Genomics.

[35] Sparks ME, Shelby KS, Kuhar D, Gundersen-Rindal DE (2014). Transcriptome of the invasive brown marmorated stink bug, *Halyomorpha halys* (Stål) (Heteroptera: Pentatomidae). PLoS One.

[36] Terenius O (2011). RNA interference in Lepidoptera: An overview of successful and unsuccessful studies and implications for experimental design. J. Insect Physiol..

[37] Baum, J. A. & Roberts, J. K. Progress towards RNAi-mediated insect pest management. Dhadialla, T. S. & Gill, S. S. (ed.) *Advances in Insect Physiology* **47**, 249-295 (2014).

[38] Singh IK, Singh S, Mogilicherla K, Shukla JN, Palli SR (2017). Comparative analysis of double-stranded RNA degradation and processing in insects. Sci. Rep..

[39] Wang K (2016). Variation in RNAi efficacy among insect species is attributable to dsRNA degradation *in vivo*. Insect Biochem. Mol. Biol..

[40] Katoch R, Thakur N (2012). Insect gut nucleases: a challenge for RNA interference mediated insect control strategies. Int. J. Biochem. Biotechnol..

[41] Christiaens O, Swevers L, Smagghe G (2014). DsRNA degradation in the pea aphid (*Acyrthosiphon pisum*) associated with lack of response in RNAi feeding and injection assay. Peptides.

[42] Mogilicherla K, Howell JL, Palli SR (2018). Improving RNAi in the brown marmorated stink bug: Identification of target genes and reference genes for RT-qPCR. Sci. Rep..

[43] Galdeano DM, Breton MC, Lopes JRS, Falk BW, Machado MA (2017). Oral delivery of double-stranded RNAs induces mortality in nymphs and adults of the Asian citrus psyllid, *Diaphorina citri*. PLoS One.

[44] Taning CNT, Andrade EC, Hunter WB, Christiaens O, Smagghe G (2016). Asian citrus psyllid RNAi pathway-RNAi evidence. Sci. Rep..

[45] Upadhyay SK (2011). RNA interference for the control of whiteflies (*Bemisia tabaci*) by oral route. J. Biosci..

[46] Chen J (2010). Feeding-based RNA interference of a trehalose phosphate synthase gene in the brown planthopper, *Nilaparvata lugens*. Insect Mol. Biol..

[47] Saleh MC (2009). Antiviral immunity in *Drosophila* requires systemic RNA interference spread. Nature.

[48] Yoon J-S, Shukla JN, Gong ZJ, Mogilicherla K, Palli SR (2016). RNA interference in the Colorado potato beetle, *Leptinotarsa decemlineata:* Identification of key contributors. Insect Biochem. Mol. Biol..

[49] Jose AM, Hunter CP (2007). Transport of sequence-specific RNA interference information between cells. Annu. Rev. Genet..

[50] Rocha JJE, Korolchuk VI, Robinson IM, O’Kane CJ (2011). A phagocytic route for uptake of double-stranded RNA in RNAi. PLoS One.

[51] Wynant N, Santos D, Van Wielendaele P, Van den Broeck J (2014). Scavenger receptor-mediated endocytosis facilitates RNA interference in the desert locust, *Schistocerca gregaria*. Insect Mol. Biol..

[52] Aung Kyaw Min, Boldbaatar Damdinsuren, Umemiya-Shirafuji Rika, Liao Min, Xuenan Xuan, Suzuki Hiroshi, Linggatong Galay Remil, Tanaka Tetsuya, Fujisaki Kozo (2011). Scavenger Receptor Mediates Systemic RNA Interference in Ticks. PLoS ONE.

[53] Gordon KHJ, Waterhouse PM (2007). RNAi for insect-proof plants. Nat. Biotechnol..

[54] Carmell MA, Hannon GJ (2004). RNase III enzymes and the initiation of gene silencing. Nat. Struct. Mol. Biol..

[55] Song M-S, Rossi JJ (2017). Molecular mechanisms of Dicer: endonuclease and enzymatic activity. Biochem. J..

[56] Welker NC (2011). Dicer’s helicase domain discriminates dsRNA termini to promote an altered reaction mode. Mol. Cell.

[57] MacRae IJ (2006). Structural basis for double-stranded RNA processing by Dicer. Science.

[58] Zhang H, Kolb FA, Jaskiewicz L, Westhof E, Filipowicz W (2004). Single processing center models for human Dicer and bacterial RNase III. Cell.

[59] Irvin-Wilson CV, Chaudhuri G (2005). Alternative initiation and splicing in dicer gene expression in human breast cells. Breast Cancer Res..

[60] Potenza N (2010). A novel splice variant of the human dicer gene is expressed in neuroblastoma cells. FEBS Lett..

[61] Yan F (2009). Identification of novel splice variants of the *Arabidopsis* DCL2 gene. Plant Cell Rep..

[62] Xu HJ (2013). Genome-wide screening for components of small interfering RNA (siRNA) and micro-RNA (miRNA) pathways in the brown planthopper, *Nilaparvata lugens* (Hemiptera: Delphacidae). Insect Mol. Biol..

[63] Miyata Keita, Ramaseshadri Parthasarathy, Zhang Yuanji, Segers Gerrit, Bolognesi Renata, Tomoyasu Yoshinori (2014). Establishing an In Vivo Assay System to Identify Components Involved in Environmental RNA Interference in the Western Corn Rootworm. PLoS ONE.

[64] Vagin VV (2006). A distinct small RNA pathway silences selfish genetic elements in the germline. Science.

[65] Houwing S (2007). A role for Piwi and piRNAs in germ cell maintenance and transposon silencing in Zebrafish. Cell.

[66] Das PP (2008). Piwi and piRNAs Act Upstream of an endogenous siRNA pathway to suppress Tc3 transposon mobility in the *Caenorhabditis elegans* germline. Mol. Cell.

[67] Carmell MA, Xuan Z, Zhang MQ, Hannon GJ (2002). The Argonaute family: tentacles that reach into RNAi. Genes Dev..

[68] Yigit E (2006). Analysis of the *C. elegans* Argonaute family reveals that distinct Argonautes act sequentially during RNAi. Cell.

[69] Hammond SM, Boettcher S, Caudy AA, Kobayashi R, Hannon GJ (2001). Argonaute2, a link between genetic and biochemical analyses of RNAi. Science.

[70] Meyer WJ (2006). Overlapping functions of argonaute proteins in patterning and morphogenesis of *Drosophila* embryos. PLoS Genet..

[71] Parker JS, Roe SM, Barford D (2005). Structural insights into mRNA recognition from a PIWI domain-siRNA guide complex. Nature.

[72] Hutvagner G, Simard MJ (2008). Argonaute proteins: Key players in RNA silencing. Nat. Rev. Mol. Cell Biol..

[73] Peters L, Meister G (2007). Argonaute Proteins: Mediators of RNA Silencing. Mol. Cell.

[74] Ender M, Meister G (2010). Argonaute proteins at a glance. J. Cell Sci..

[75] Huang T, Zhang X (2012). Contribution of the Argonaute-1 Isoforms to Invertebrate Antiviral Defense. PLoS One.

[76] Hartig JV, Tomari Y (2007). piRNAs—the ancient hunters of genome invaders. Genes Dev..

[77] Pane A, Wehr K, Schüpbach T (2007). Zucchini and squash encode two putative nucleases required for rasiRNA production in the *Drosophila* germline. Dev. Cell.

[78] Zhu L (2012). Molecular cloning of BmTUDOR-SN and analysis of its role in the RNAi pathway in the silkworm, *Bombyx mori* (Lepidoptera: Bombycidae). Appl. Entomol. Zool..

[79] Liu Y, Ye X, Jiang F, Liang C, Chen D (2009). C3PO, an endoribonuclease that promotes RNAi by facilitating RISC activation. Science..

[80] Findley S, Maelstrom DA (2003). *Drosophila* spindle-class gene, encodes a protein that colocalizes with Vasa and RDE1/AGO1 homolog, Aubergine, in nuage. Development.

[81] Lipardi C, Paterson BM (2010). Identification of an RNA-dependent RNA polymerase in *Drosophila* establishes a common theme in RNA silencing. Fly (Austin).

[82] Caudy AA, Myers M, Hannon GJ, Hammond SM (2002). Fragile X-related protein and VIG associate with the RNA interference machinery. Genes Dev..

[83] Lasko P (2013). The DEAD-box helicase Vasa: Evidence for a multiplicity of functions in 3 RNA processes and developmental biology. Biochim. Biophys. Acta J..

[84] Schneider MD (2006). Gawky is a component of cytoplasmic mRNA processing bodies required for early *Drosophila* development. J. Cell Biol..

[85] Weitzer S, Martinez J (2007). The human RNA kinase hClp1 is active on 3′ transfer RNA exons and short interfering RNAs. Nature.

[86] Siomi H, Siomi MC (2009). On the road to reading the RNA-interference code. Nature.

[87] Liu Y, Cheng S (2015). Functional roles of DExD/H-box RNA helicases in pre-mRNA splicing. J. Biomed. Sci..

[88] Wang YAN, Guthrie C (1998). PRP16, a DEAH-box RNA helicase, is recruited to the spliceosome primarily via its nonconserved N-terminal domain. RNA.

[89] Arimatsu Y, Kotani E, Sugimura Y, Furusawa T (2007). Molecular characterization of a cDNA encoding extracellular dsRNase and its expression in the silkworm, *Bombyx mori*. Insect Biochem. Mol. Biol..

[90] Christiaens O, Smagghe G (2014). The challenge of RNAi-mediated control of hemipterans. Curr. Opin. Insect Sci..

[91] Guan R-B (2018). A nuclease specific to lepidopteran insects suppresses RNAi. J. Biol. Chem..

[92] Liu J, Swevers L, Iatrou K, Huvenne H, Smagghe G (2012). *Bombyx mori* DNA/RNA non-specific nuclease: Expression of isoforms in insect culture cells, subcellular localization and functional assays. J. Insect Physiol..

[93] Thomas MF, L’Etoile ND, Ansel KM (2014). Eri1: A conserved enzyme at the crossroads of multiple RNA processing pathways. Trends Genet..

[94] Han BW, Hung JH, Weng Z, Zamore PD, Ameres SL (2011). The 3′-to-5′ exoribonuclease Nibbler shapes the 3′ ends of microRNAs bound to *Drosophila* Argonaute1. Curr. Biol..

[95] Sabin LR (2009). Ars2 regulates both miRNA- and siRNA-dependent silencing and suppresses RNA virus infection in *Drosophila*. Cell.

[96] Beyenbach KW, Wieczorek H (2006). The V-type H+ ATPase: molecular structure and function, physiological roles and regulation. J. Exp. Biol..

[97] Castellanos NL, Smagghe G, Sharma R, Oliveira EE, Christiaens O (2018). Liposome encapsulation and EDTA formulation of dsRNA targeting essential genes increase oral RNAi-caused mortality in the Neotropical stink bug *Euschistus heros*. Pest Manag. Sci..

[98] Mohammed AMA (2016). RNAi-based silencing of genes encoding the vacuolar-ATPase subunits a and c in pink bollworm (*Pectinophora gossypiella*). Afr. J. Biotechnol..

[99] Whyard S, Singh AD, Wong S (2009). Ingested double-stranded RNAs can act as species-specific insecticides. Insect Biochem. Mol. Biol..

[100] Finbow ME, Harrison MA (1997). The vacuolar H^+^-ATPase: a universal proton pump of eukaryotes. Biochem. J..

[101] Nelson N (2000). The cellular biology of proton-motive force generation by V-ATPases. J. Exp. Biol..

[102] Fishilevich E (2016). Use of chromatin remodeling ATPases as RNAi targets for parental control of western corn rootworm (*Diabrotica virgifera virgifera*) and Neotropical brown stink bug (*Euschistus heros*). Insect Biochem. Mol. Biol..

[103] Zotti M (2017). RNAi technology in crop protection against arthropod pests, pathogens and nematodes. Pest Manag. Sci..

[104] Prentice K (2016). RNAi-based gene silencing through dsRNA injection or ingestion against the African sweet potato weevil *Cylas puncticollis* (Coleoptera: Brentidae). Pest Manag. Sci..

[105] Prentice K, Smagghe G, Gheysen G, Christiaens O (2019). Nuclease activity decreases the RNAi response in the sweetpotato weevil *Cylas puncticollis*. Insect Biochem. Mol. Biol..

[106] Lin YH, Huang JH, Liu Y, Belles X, Lee HJ (2017). Oral delivery of dsRNA lipoplexes to German cockroach protects dsRNA from degradation and induces RNAi response. Pest Manag. Sci..

[107] Jin S, Singh ND, Li L, Zhang X, Daniell H (2015). Engineered chloroplast dsRNA silences *cytochrome p450 monooxygenase*, *v-ATPase* and *chitin synthase* genes in the insect gut and disrupts *Helicoverpa armigera* larval development and pupation. Plant Biotechnol. J..

[108] Garbutt JS, Reynolds SE (2012). Induction of RNA interference genes by double-stranded RNA; implications for susceptibility to RNA interference. Insect Biochem. Mol. Biol..

[109] Borges, M. *et al*. Metodologias de criação e manejo de colônias de percevejos da soja (Hemíptera - Pentatomidae) para estudos de comportamento e ecologia química. https://ainfo.cnptia.embrapa.br/digital/bitstream/CENARGEN/27988/1/doc182.pdf (Embrapa, 2006).

[110] Bolger AM, Lohse M, Usadel B (2014). Trimmomatic: A flexible trimmer for Illumina sequence data. Bioinformatics.

[111] Haas BJ (2013). *De novo* transcript sequence reconstruction from RNA-seq using the Trinity platform for reference generation and analysis. Nat. Protoc..

[112] Apweiler R (2004). UniProt: the universal protein knowledgebase. Nucleic Acids Res..

[113] Buchfink B, Xie C, Huson DH (2014). Fast and sensitive protein alignment using DIAMOND. Nat. Methods.

[114] Liu S, Ding Z, Zhang C, Yang B, Liu Z (2010). Gene knockdown by intro-thoracic injection of double-stranded RNA in the brown planthopper, *Nilaparvata lugens*. Insect Biochem. Mol. Biol..

[115] Livak KJ, Schmittgen TD (2001). Analysis of relative gene expression data using real-time quantitative PCR and the 2−ΔΔCT method. Methods.

