# Supplementary material for: First transcriptome of the Neotropical pest Euschistus heros (Hemiptera: Pentatomidae) with dissection of its siRNA machinery
Source: Sci Rep. 2020 Mar 17;10:4856. doi: 10.1038/s41598-020-60078-3 (PMC7078254; doi:10.1038/s41598-020-60078-3)
Supplement: Supplementary file 1 — Supplementary material. [file 41598_2020_60078_MOESM1_ESM.docx]

**First transcriptome of the Neotropical pest** *Euschistus heros* **(Hemiptera: Pentatomidae) with dissection of its siRNA machinery**

Deise Cagliari^1*^; Naymã Pinto Dias^1^; Ericmar Ávila dos Santos^1^; Leticia Neutzling Rickes^1^; Frederico Schmitt Kremer^2^; Juliano Ricardo Farias^3^; Giuvan Lenz^4^; Diogo Manzano Galdeano^5^; Flávio Roberto Mello Garcia^6^; Guy Smagghe^7*^; Moisés João Zotti^1*^

^1^ Department of Crop Protection, Molecular Entomology, Federal University of Pelotas, Pelotas, Brazil.

^2^ Center for Technological Development, Bioinformatics and Proteomics Laboratory, Federal University of Pelotas, Pelotas, Brazil.

^3^ Department of Crop Protection, Universidade Regional Integrada do Alto Uruguai, Santo Ângelo, Brazil.

^4^ Agricultural Research and Development Center, UPL, Pereiras, Brazil

^5^ Sylvio Moreira Citrus Center, Agronomic Institute of Campinas, Cordeirópolis, São Paulo, Brazil.

^6^ Department of Crop Protection, Insect Ecology Laboratory, Federal University of Pelotas, Pelotas, Brazil.

^7^ Department of Plants and Crops, Ghent University, Ghent, Belgium.

*Corresponding authors:

Deise Cagliari, Moisés João Zotti, Guy Smagghe

deisycagliari@yahoo.com.br; moises.zotti@ufpel.edu.br; guy.smagghe@ugent.be

+55 55 9 9162-2651; +55 55 9 9671-2207; +32 9 2646150

**Table S1:** *Euschistus heros* sequence comparison to other insect species. BLASTx comparison of *E. heros* known sequences to other insect genera (bitscore>50) against the nr protein database of the NCBI with hits <0.54%, grouped in other hits.

| **Species** | **Hits** | **Percentage (%)** |
| --- | --- | --- |
| *Stegodyphus mimosarum* | 269 | 0.52 |
| *Nasonia vitripennis* | 253 | 0.48 |
| *Bombyx mori* | 192 | 0.37 |
| *Pediculus humanus corporis* | 176 | 0.34 |
| *Cerapachys biroi* | 153 | 0.29 |
| *Dendroctonus ponderosae* | 147 | 0.28 |
| *Camponotus floridanus* | 135 | 0.26 |
| *Blattella germanica* | 129 | 0.25 |
| *Apis mellifera* | 123 | 0.24 |
| *Cyphomyrmex costatus* | 118 | 0.23 |
| *Daphnia magna* | 114 | 0.22 |
| *Dufourea novaeangliae* | 114 | 0.22 |
| *Ixodes ricinus* | 108 | 0.21 |
| *Bactrocera latifrons* | 108 | 0.21 |
| *Tabanus bromius* | 106 | 0.20 |
| *Plautia stali* | 106 | 0.20 |
| *Aedes albopictus* | 99 | 0.19 |
| *Papilio machaon* | 98 | 0.19 |
| *Bactrocera dorsalis* | 96 | 0.18 |
| *Phlebotomus papatasi* | 96 | 0.18 |
| *Nilaparvata lugens* | 95 | 0.18 |
| *Halyomorpha halys* | 95 | 0.18 |
| *Trachymyrmex cornetzi* | 94 | 0.18 |
| *Harpegnathos saltator* | 92 | 0.18 |
| *Habropoda laboriosa* | 87 | 0.17 |
| *Operophtera brumata* | 84 | 0.16 |
| *Melipona quadrifasciata* | 71 | 0.14 |
| *Fopius arisanus* | 71 | 0.14 |
| *Danaus plexippus* | 62 | 0.12 |
| *Lutzomyia longipalpis* | 61 | 0.12 |
| *Apolygus lucorum* | 59 | 0.11 |
| *Bactrocera tryoni* | 59 | 0.11 |
| *Oncopeltus fasciatus* | 57 | 0.11 |
| *Anasa tristis* | 57 | 0.11 |
| *Ceratitis capitata* | 54 | 0.10 |
| *Zeugodacus cucurbitae* | 53 | 0.10 |
| *Trachymyrmex septentrionalis* | 48 | 0.09 |
| *Papilio xuthus* | 46 | 0.09 |
| *Acromyrmex echinatior* | 46 | 0.09 |
| *Pyrrhocoris apterus* | 46 | 0.09 |
| *Corethrella appendiculata* | 44 | 0.08 |
| *Aedes aegypti* | 44 | 0.08 |
| *Clunio marinus* | 43 | 0.08 |
| *Oryctes borbonicus* | 43 | 0.08 |
| *Trachymyrmex zeteki* | 42 | 0.08 |
| *Lepeophtheirus salmonis* | 42 | 0.08 |
| *Pectinophora gossypiella* | 41 | 0.08 |
| *Scylla olivacea* | 37 | 0.07 |
| *Rhipicephalus pulchellus* | 37 | 0.07 |
| *Pararge aegeria* | 36 | 0.07 |
| *Culex tarsalis* | 36 | 0.07 |
| *Dysdercus peruvianus* | 35 | 0.07 |
| *Drosophila ananassae* | 34 | 0.07 |
| *Lucilia cuprina* | 33 | 0.06 |
| *Orchesella cincta* | 32 | 0.06 |
| *Atta cephalotes* | 31 | 0.06 |
| *Strigamia maritima* | 29 | 0.06 |
| *Culex quinquefasciatus* | 29 | 0.06 |
| *Daphnia pulex* | 29 | 0.06 |
| *Psorophora albipes* | 27 | 0.05 |
| *Stomoxys calcitrans* | 26 | 0.05 |
| *Atta colombica* | 25 | 0.05 |
| *Nyssomyia neivai* | 25 | 0.05 |
| *Solenopsis invicta* | 23 | 0.04 |
| *Anopheles albimanus* | 21 | 0.04 |
| *Riptortus clavatus* | 21 | 0.04 |
| *Anacanthocoris striicornis* | 21 | 0.04 |
| *Pristhesancus plagipennis* | 20 | 0.04 |
| *Euschistus heros* | 20 | 0.04 |
| *Helicoverpa zea* | 19 | 0.04 |
| *Eurygaster integriceps* | 18 | 0.03 |
| *Anopheles stephensi* | 18 | 0.03 |
| *Anopheles gambiae* | 17 | 0.03 |
| *Neoseiulus cucumeris* | 17 | 0.03 |
| *Anopheles atroparvus* | 16 | 0.03 |
| *Drosophila busckii* | 16 | 0.03 |
| *Drosophila melanogaster* | 15 | 0.03 |
| *Drosophila virilis* | 15 | 0.03 |
| *Cotesia congregata* | 14 | 0.03 |
| *Lygus lineolaris* | 14 | 0.03 |
| *Drosophila willistoni* | 14 | 0.03 |
| *Anopheles culicifacies* | 14 | 0.03 |
| *Glossina pallidipes* | 13 | 0.02 |
| *Eurygaster maura* | 13 | 0.02 |
| *Musca domestica* | 13 | 0.02 |
| *Ixodes scapularis* | 13 | 0.02 |
| *Cimex lectularius* | 13 | 0.02 |
| *Caligus clemensi* | 13 | 0.02 |
| *Aphis gossypii* | 12 | 0.02 |
| *Tetranychus urticae* | 12 | 0.02 |
| *Laodelphax striatella* | 11 | 0.02 |
| *Amblyomma aureolatum* | 11 | 0.02 |
| *Anopheles quadriannulatus* | 11 | 0.02 |
| *Anopheles funestus* | 11 | 0.02 |
| *Nezara viridula* | 11 | 0.02 |
| *Glossina morsitans morsitans* | 10 | 0.02 |
| *Anopheles sinensis* | 10 | 0.02 |
| *Anopheles dirus* | 10 | 0.02 |
| *Heliothis virescens* | 10 | 0.02 |
| *Haematobia irritans* | 10 | 0.02 |
| *Triatoma matogrossensis* | 10 | 0.02 |
| *Artemia parthenogenetica* | 9 | 0.02 |
| *Drosophila simulans* | 9 | 0.02 |
| *Locusta migratoria* | 9 | 0.02 |
| *Coptotermes formosanus* | 9 | 0.02 |
| *Anopheles epiroticus* | 9 | 0.02 |
| *Rhipicephalus appendiculatus* | 9 | 0.02 |
| *Anopheles minimus* | 8 | 0.02 |
| *Triatoma brasiliensis* | 8 | 0.02 |
| *Hyalomma excavatum* | 8 | 0.02 |
| *Glossina fuscipes fuscipes* | 8 | 0.02 |
| *Cercopis vulnerata* | 8 | 0.02 |
| *Apis cerana* | 8 | 0.02 |
| *Maconellicoccus hirsutus* | 8 | 0.02 |
| *Chorthippus parallelus* | 7 | 0.01 |
| *Anopheles coluzzii* | 7 | 0.01 |
| *Drosophila persimilis* | 7 | 0.01 |
| *Glossina austeni* | 7 | 0.01 |
| *Amblyomma cajennense* | 6 | 0.01 |
| *Glyptapanteles flavicoxis* | 6 | 0.01 |
| *Papilio polytes* | 6 | 0.01 |
| *Graphosoma rubrolineatum* | 6 | 0.01 |
| *Anopheles melas* | 6 | 0.01 |
| *Drosophila pseudoobscura pseudoobscura* | 6 | 0.01 |
| *Nabis sp.* HMR-1997a | 6 | 0.01 |
| *Anopheles merus* | 5 | 0.01 |
| *Anopheles farauti* | 5 | 0.01 |
| *Eurydema gebleri* | 5 | 0.01 |
| *Drosophila mojavensis* | 5 | 0.01 |
| *Drosophila erecta* | 5 | 0.01 |
| *Drosophila yakuba* | 5 | 0.01 |
| *Drosophila grimshawi* | 5 | 0.01 |
| *Chinavia ubica* | 4 | 0.01 |
| *Ctenocephalides felis* | 4 | 0.01 |
| *Glossina brevipalpis* | 4 | 0.01 |
| *Amblyomma triste* | 4 | 0.01 |
| *Phenacoccus solenopsis* | 4 | 0.01 |
| *Agrilus planipennis* | 4 | 0.01 |
| *Liposcelis entomophila* | 4 | 0.01 |
| *Colaphellus bowringi* | 4 | 0.01 |
| *Sarcoptes scabiei* | 4 | 0.01 |
| *Sogatella furcifera* | 4 | 0.01 |
| *Bagrada hilaris* | 4 | 0.01 |
| *Cotesia chilonis* | 4 | 0.01 |
| *Misgolas hubbardi* | 4 | 0.01 |
| *Spodoptera exigua* | 4 | 0.01 |
| *Mayetiola destructor* | 4 | 0.01 |
| *Anastrepha fraterculus* | 3 | 0.01 |
| *Drosophila hydei* | 3 | 0.01 |
| *Lethocerus deyrollei* | 3 | 0.01 |
| *Galendromus occidentalis* | 3 | 0.01 |
| *Anastrepha suspensa* | 3 | 0.01 |
| *Ranatra unicolor* | 3 | 0.01 |
| *Tapinoma nigerrimum* | 3 | 0.01 |
| *Dermanyssus gallinae* | 3 | 0.01 |
| *Drosophila heteroneura* | 3 | 0.01 |
| *Spodoptera frugiperda* | 3 | 0.01 |
| *Dolycoris baccarum* | 3 | 0.01 |
| *Anopheles aquasalis* | 3 | 0.01 |
| *Drosophila sechellia* | 3 | 0.01 |
| *Chilo suppressalis* | 3 | 0.01 |
| *Bemisia tabaci* | 3 | 0.01 |
| *Anopheles darlingi* | 3 | 0.01 |
| *Chironomus tentans* | 3 | 0.01 |
| *Anopheles christyi* | 3 | 0.01 |
| *Helicoverpa armigera* | 2 | <0.01 |
| *Campsomeris sp.* Cas_Ok01 | 2 | <0.01 |
| *Nephotettix cincticeps* | 2 | <0.01 |
| *Gryllus firmus* | 2 | <0.01 |
| *Amegilla dulcifera* | 2 | <0.01 |
| *Rubiconia intermedia* | 2 | <0.01 |
| *Glossina palpalis* | 2 | <0.01 |
| *Acrosternum hilare* | 2 | <0.01 |
| *Chironomus thummi* | 2 | <0.01 |
| *Portunus pelagicus* | 2 | <0.01 |
| *Graminella nigrifrons* | 2 | <0.01 |
| *Tenebrio molitor* | 2 | <0.01 |
| *Chymomyza amoena* | 2 | <0.01 |
| *Nysius plebeius* | 2 | <0.01 |
| *Leptinotarsa decemlineata* | 2 | <0.01 |
| *Antheraea yamamai* | 2 | <0.01 |
| *Planococcus kraunhiae* | 2 | <0.01 |
| *Forficula auricularia* | 2 | <0.01 |
| *Diploptera punctata* | 2 | <0.01 |
| *Tityus obscurus* | 2 | <0.01 |
| *Ornithodoros parkeri* | 2 | <0.01 |
| *Laccotrephes japonensis* | 2 | <0.01 |
| *Marsupenaeus japonicus* | 2 | <0.01 |
| *Bactrocera umbrosa* | 2 | <0.01 |
| *Amblyomma maculatum* | 2 | <0.01 |
| *Cyrtorhinus lividipennis* | 2 | <0.01 |
| *Locusta migratoria manilensis* | 2 | <0.01 |
| *Ctenoplusia agnata* | 2 | <0.01 |
| *Oxya chinensis* | 2 | <0.01 |
| *Amblyomma parvum* | 2 | <0.01 |
| *Anopheles maculatus* | 2 | <0.01 |
| *Anopheles arabiensis* | 2 | <0.01 |
| *Gryllus pennsylvanicus* | 2 | <0.01 |
| *Ctenolepisma lineata* | 2 | <0.01 |
| *Glaucias subpunctatus* | 2 | <0.01 |
| *Bombus lapidarius* | 2 | <0.01 |
| *Dastarcus helophoroides* | 2 | <0.01 |
| *Lethocerus indicus* | 2 | <0.01 |
| *Haemaphysalis longicornis* | 2 | <0.01 |
| *Heliconius erato* | 2 | <0.01 |
| *Amblyomma sculptum* | 2 | <0.01 |
| *Scylla paramamosain* | 2 | <0.01 |
| *Heteropoda venatoria* | 2 | <0.01 |
| *Latrodectus hesperus* | 2 | <0.01 |
| *Paracyclopina nana* | 1 | <0.01 |
| *Thenus orientalis* | 1 | <0.01 |
| *Thermobia domestica* | 1 | <0.01 |
| *Glyptapanteles indiensis* | 1 | <0.01 |
| *Misgolas dereki* | 1 | <0.01 |
| *Adelphocoris suturalis* | 1 | <0.01 |
| *Rhipicephalus geigyi* | 1 | <0.01 |
| *Acanthosoma crassicaudum* | 1 | <0.01 |
| *Melanoplus sanguinipes* | 1 | <0.01 |
| *Drosicha corpulenta* | 1 | <0.01 |
| *Leptopilina clavipes* | 1 | <0.01 |
| *Thitarodes armoricanus* | 1 | <0.01 |
| *Neoseiulus californicus* | 1 | <0.01 |
| *Varroa destructor* | 1 | <0.01 |
| *Hister sp. APV-2005* | 1 | <0.01 |
| *Heliothis subflexa* | 1 | <0.01 |
| *Tribolium madens* | 1 | <0.01 |
| *Phytoseiulus persimilis* | 1 | <0.01 |
| *Dacus demmerezi* | 1 | <0.01 |
| *Periplaneta americana* | 1 | <0.01 |
| *Parochlus steinenii* | 1 | <0.01 |
| *Phaenacantha marcida* | 1 | <0.01 |
| *Ericerus pela* | 1 | <0.01 |
| *Ornithodoros moubata* | 1 | <0.01 |
| *Cnaphalocrocis medinalis* | 1 | <0.01 |
| *Euschistus quadrator* | 1 | <0.01 |
| *Myrmecia midas* | 1 | <0.01 |
| *Calliphora vicina* | 1 | <0.01 |
| *Parides eurimedes* | 1 | <0.01 |
| *Pterostichus campbelli* | 1 | <0.01 |
| *Tityus serrulatus* | 1 | <0.01 |
| *Nephilengys cruentata* | 1 | <0.01 |
| *Carios mimon* | 1 | <0.01 |
| *Chironomus plumosus* | 1 | <0.01 |
| *Reticulitermes speratus* | 1 | <0.01 |
| *Paratlanticus ussuriensis* | 1 | <0.01 |
| *Fenneropenaeus chinensis* | 1 | <0.01 |
| *Hyalomma rufipes* | 1 | <0.01 |
| *Samia ricini* | 1 | <0.01 |
| *Stenus nitens* | 1 | <0.01 |
| *Megaselia scalaris* | 1 | <0.01 |
| *Hadronyche infensa* | 1 | <0.01 |
| *Megacopta punctatissima* | 1 | <0.01 |
| *Cryptolestes ferrugineus* | 1 | <0.01 |
| *Ixodes persulcatus* | 1 | <0.01 |
| *Hylaeus confusus* | 1 | <0.01 |
| *Delia antiqua* | 1 | <0.01 |
| *Aphonopelma sp.* WDB-1998 | 1 | <0.01 |
| *Amblyomma americanum* | 1 | <0.01 |
| *Sclerodermus sp.* MQW-2015 | 1 | <0.01 |
| *Microplitis mediator* | 1 | <0.01 |
| *Homarus americanus* | 1 | <0.01 |
| *Aethus indicus* | 1 | <0.01 |
| *Caligus rogercresseyi* | 1 | <0.01 |
| *Oxycarenus pallens* | 1 | <0.01 |
| *Cotesia rubecula* | 1 | <0.01 |
| *Parasitid 'Pas'* | 1 | <0.01 |
| *Pachliopta aristolochiae* | 1 | <0.01 |
| *Telenomus podisi* | 1 | <0.01 |
| *Lymantria dispar* | 1 | <0.01 |
| *Cupiennius salei* | 1 | <0.01 |
| *Carpelimus sp.* HMR-1993 | 1 | <0.01 |
| *Arma custos* | 1 | <0.01 |
| *Rhipicephalus sanguineus* | 1 | <0.01 |
| *Amblyomma hebraeum* | 1 | <0.01 |
| *Rhagophthalmus ohbai* | 1 | <0.01 |
| *Lysiphlebus testaceipes* | 1 | <0.01 |
| *Mesobuthus martensii* | 1 | <0.01 |
| *Hadrurus arizonensis* | 1 | <0.01 |
| *Antricola delacruzi* | 1 | <0.01 |
| *Polyrhachis vicina* | 1 | <0.01 |
| *Adelphocoris lineolatus* | 1 | <0.01 |
| *Cryptocellus centralis* | 1 | <0.01 |
| *Boisea trivittata* | 1 | <0.01 |
| *Antheraea pernyi* | 1 | <0.01 |
| *Oxytate striatipes* | 1 | <0.01 |
| *Delphinia picta* | 1 | <0.01 |
| *Alopecosa kochi* | 1 | <0.01 |
| *Bacillus rossius* | 1 | <0.01 |
| *Rhynchosciara americana* | 1 | <0.01 |
| *Conogethes punctiferalis* | 1 | <0.01 |
| *Mastotermes darwiniensis* | 1 | <0.01 |
| *Gampsocleis gratiosa* | 1 | <0.01 |
| *Bombyx mandarina* | 1 | <0.01 |
| *Lycosa singoriensis* | 1 | <0.01 |
| *Cypridopsis vidua* | 1 | <0.01 |
| *Anastrepha sororcula* | 1 | <0.01 |
| *Lonomia obliqua* | 1 | <0.01 |
| *Trigonotylus caelestialium* | 1 | <0.01 |
| *Apodemia mormo* | 1 | <0.01 |
| *Procambarus clarkii* | 1 | <0.01 |

**Data S1:** Sequences of *E. heros* dsRNA uptake.

**Scavenger receptor**

>TRINITY_DN27164_c0_g2_i2 length= 2955 nt

TTATAATTATCTTTATTTAATCACAATAAATGGAATGTCTGCAAAAATACATCGTTTTTTCTCTGAATATAAAATATTTTTCACATTTAGTTGTATTCGCTCTTAGCTCTGTATTCGCTAAGTAATGAGTTTTAACCATTTCTTCCAATGCTTATTTACAAGACACCAGAACCATCGAATGCATTTTGCTGGTCCGATTGCATTTATATTGCAAACTTTGTATTGCAGTGCGAATTGATCAAAAAGAAAATTAGAGAAAAAACATCCTCTCATAGAGAGCTCAACACTAACACTAATTAGTGATTAGTACTCGCATGATGGCACTGGTGTGTAGTAAATAATTACAGGCAGCAAATACCATTTACTAAATATCACACATAATATACTTCACATGATTTTATACAGTTTATACATTAAAATAAGTAATAAATATTTAACCAATACATTTCTCCTTTAATGTGAGGGATTTTTTAATAAATGCCTGATTTCCACATCCACACTATAATACATATAAGTATATATTTATTTAAAAAAACAAAAATAAAAAACAAACAAAGAACAAATAATAAAAATAAACAAAATATTTCTGAAAAACTAACAATAATAAATTCTCGATAAATTGGTAAAATAATGCACCTCATCACAAATTTTAACCATTTTCATACAAAAATCTCAATTAATATCTAATACATAACATCAGGCACAAGGCTTCAAATATTCCTACAACTAAATCCTCATAAACACTCATCAAACGCAGAAATATAATGACCACTATACAATGTATATACACAATTTATGTATAAAAATACTAAGTACTCCTTGATTGGACGGATATGTTACAAATGTTTATAATAAATGTATGTATAAACATACACTTACAAACATAAGAAAAAAATATTTGAAAAGCTTACCTTAGCTAATGGACACACAAAATGAAATGTACGTATATCACAAAAAAGGTGATTGAATTAAAAAAAAAATATTTTTTTTTTCGATGTTCAACAATTAAAACAACAATAAAAAATAAAAAGAAATTGAGCAATGCTTTAAACAGCTAGTCACAAATATTCATTTCTCTCAACTGATGTTCACTACTTAATTTATCAGCTTCTAAAATGATCATTACCGCTTTTTAGTTTAAATTTGTACTGCGGTAAAGTCTGTCCTCCAAAAAATAAAATAAAAATATTTGGAAACTTATGTTATGATTGATCACCAACAAAAGCTGGATTTGTCTTGGTATCGGGGTATTTGTGTCCGTTGGTCTGGCCGTTGGCTTGTTGCTTAGCTTTCTTGTTCGCTTCGTCGTTGGCTGCATAGTGAGCTGTTCCTTCGAGGCTGAGAGTCTCTTGTCTTTGAGAGTTCCTAACGAGGCAGAATGCGGAAAGGATGAAGAAAAAGAAACCACAAATAAAGAAAATATAAAGAAGGACCATCTTTAAAACTGGCGGAACTGTTGTAGCGAGGTTCATAAGTTTGGTTACATTCTCAGGCAAACCGCTAATTCCATCTTCAAACCACATGATGGGGAAAACTATGTCTGGGAATGTTGCCACTTGTTTGATGTCCACAACTTGACTTACGGCCAAATTTACTTGAATTCTAGCTTGTGCTCTTAAACCTGCTCCCATGGTTGGCTGAACGTCAAGCCAAAAGCCGTGTTTTTCAGGATCAGGCTCAGAAATTCCTTCAACAGCAGTCCTAAGTTTTGGATCAGCAAGGTAGAAGTGAGGGAATGAAAGCATGATGGGGGAGTCATATTGGCAAAGGGAAACATTGAACAATCCATGTGGTGCACAAGGAGGTCCTGATGGGCAGAAACATTCGTTATCTGGATTCTTTTCAATTTCTGCAAAAACGTCATCAGGTGGTGTGAATCTGTACCCGAGAACATTTCCTGGGGTGTCGACTTCTTCCTTGTATTTCAGAGGGAGCAGCCTGCAGAGATCCTTGTCGAAGACATAGAGTGTAGTATTTTTAGTAATGTGGGGTGGGAAAATTGATCCGTCACTGCCTGCCAGTCTGTTACATTCTTCGCTCTTCCAATGCGGCAGATGAGACATTCCGTTGAACCGATCGACGAGACCGTACCTTGTTATGTCATTAGCACCAGTGAATACGGTCACAATATCTTTGGAAGTTCCATTTTTCCCATACAAAAGACCAAATTCTTCATAAGGAAGCTTCTGCTCTTTTGGTACGACGTCTTTAGCTAATTTGAGAAGAGGATCCTCATATCCCCAGAGCAATTGGCCGACAGAGACCTCCACGAATGGTTTTATCTTTAAAATATCCATGATGGAAGCCATTGCCAGCCTGAGGAATCGTGCAGCGTGTTTACTCTGAGAAGTGGCGCTCAACATTGGAATATTAGGAACAACGACCATATCATTCTCATCGCCTACTGACTGATCAGGATCGAATTTGAAAATTTTCTGTTGTTGAAATGTAACAGTTCCATTGTCATTAAATGTCAAATTTACTTTTTCCCAAGTTTCAATGTAGACGAATGGACCCAGCTCATCAAGAATAGGTTTGTCTCCCCGATTGAGGAAGTCATCAGCGTTGGTGACGTTGTAGACGTATACCCTGAAGACTGGTTCCACAGGGGGCTTCCTCCACATGTCGAACACTTGGCCGCCTTCTCGGAGGGTAATTTCCTTATCTATGACTAGGTTGACAATACTGCTAAAGAATATAGCTATGAAGATCCCGACGAATATAAGTATCAGCGACACAACGATCACTATCCACCACTTCCTAAGAAAGCTGCTGGTGAGCTTGTCGCAAAGCTTCCTCTTGTTGTGCATGGCGGTGGGTTCGATGCCCGAAAGCAATGAGGAAGAAACGAAGTATAAAGTTGTAGAAAAGAAGTTGGAGAAGCGATCACAGAACACACCCGGACCACTGTTGCACCGAGAAGTCGAGAGATCACCAGCTCACGGCCGACTAACT

Protein: RF -2: -2810 -> -1203 (535 aa)

Comparison with *Halyomorpha halys*, PREDICTED: scavenger receptor class B member 1 isoform X1 - Sequence ID: XP_014285135.1

E= 0.0; bits= 1085

Query 1 MHNKRKLCDKLTSSFLRKWWIVIVVSLILIFVGIFIAIFFSSIVNLVIDKEITLREGGQV 60

MHNKRKLCDKLTSSFLRKWW+VI+VSLILIFVGIFIAIFFSSIVNLVIDKEITLREGGQV

Sbjct 1 MHNKRKLCDKLTSSFLRKWWVVIIVSLILIFVGIFIAIFFSSIVNLVIDKEITLREGGQV 60

Query 61 FDMWRKPPVEPVFRVYVYNVTNADDFLNRGDKPILDELGPFVYIETWEKVNLTFNDNGTV 120

FDMWRKPPVEP+FRVYVYNVTNADDFLNRGDKPILDELGPFVY+ETWEKVNLTFNDNGTV

Sbjct 61 FDMWRKPPVEPIFRVYVYNVTNADDFLNRGDKPILDELGPFVYVETWEKVNLTFNDNGTV 120

Query 121 TFQQQKIFKFDPDQSVGDENDMVVVPNIPMLSATSQSKHAARFLRLAMASIMDILKIKPF 180

TFQQQKI+KFDPDQSVGDE+DMVVVPNIPMLSATSQSKHAARFLRLAMASIMDILKIKPF

Sbjct 121 TFQQQKIYKFDPDQSVGDESDMVVVPNIPMLSATSQSKHAARFLRLAMASIMDILKIKPF 180

Query 181 VEVSVGQLLWGYEDPLLKLAKDVVPKEQKLPYEEFGLLYGKNGTSKDIVTVFTGANDITR 240

VEVSVGQLLWGYEDPLLKLAKDVVPKEQKLPYEEFGLLYGKNGTSKDI+TVFTGANDIT+

Sbjct 181 VEVSVGQLLWGYEDPLLKLAKDVVPKEQKLPYEEFGLLYGKNGTSKDIMTVFTGANDITK 240

Query 241 YGLVDRFNGMSHLPHWKSEECNRLAGSDGSIFPPHITKNTTLYVFDKDLCRLLPLKYKEE 300

YGLVDRFNGMSHLPHWKSEECNRLAGSDGSIFPPHITKNTTLYVFDKDLCRLLPL++K+E

Sbjct 241 YGLVDRFNGMSHLPHWKSEECNRLAGSDGSIFPPHITKNTTLYVFDKDLCRLLPLQFKKE 300

Query 301 VDTPGNVLGYRFTPPDDVFAEIEKNPDNECFCPSGPPCAPHGLFNVSLCQYDSPIMLSFP 360

V+TPG+VLGYRF+PP++VFAEIEKNPDNECFCPSGPPCAPHGLFNVSLCQYDSP+MLSFP

Sbjct 301 VNTPGDVLGYRFSPPENVFAEIEKNPDNECFCPSGPPCAPHGLFNVSLCQYDSPVMLSFP 360

Query 361 HFYLADPKLRTAVEGISEPDPEKHGFWLDVQPTMGAGLRAQARIQVNLAVSQVVDIKQVA 420

HFYLADPKLRTAVEGISEPDPEKHGFWLDVQPTMGAGLRAQARIQ+NLAVSQVVDIKQVA

Sbjct 361 HFYLADPKLRTAVEGISEPDPEKHGFWLDVQPTMGAGLRAQARIQINLAVSQVVDIKQVA 420

Query 421 TFPDIVFPIMWFEDGISGLPENVTKLMNLATTVPPVLKMVLLYIFFICGFFFFILSAFCL 480

TFPDIVFPIMWFEDGISGLPE+VTKLMNLATTVPPVLKMVLLYIFFICGFFFFILSAFCL

Sbjct 421 TFPDIVFPIMWFEDGISGLPESVTKLMNLATTVPPVLKMVLLYIFFICGFFFFILSAFCL 480

Query 481 VRNSQRQETLSLEGTAHYAANDEANKKAKQQANGQTNGHKYPDTKTNPAFVGDQS 535

VRNSQRQETLSLEGTAHYAANDEANKKAKQQANGQTNGHKYPDTKTNPAFVGDQS

Sbjct 481 VRNSQRQETLSLEGTAHYAANDEANKKAKQQANGQTNGHKYPDTKTNPAFVGDQS 535

Graphical representation


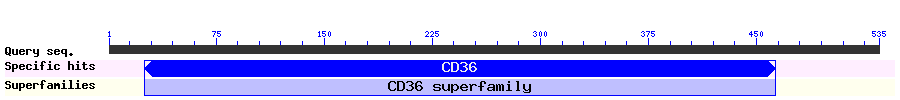


**CG4966 = orthologous to the Hermansky-Pudlak Syndrome 4**

>TRINITY_DN24861_c0_g1_i2 length= 3098 nt

AAGTATTATTATATTTTTTCAATCATCTAGTAATATTAAAAGATGAATAGAGTGGTACCAATTCAGTAACCACCGGAAAAGACTTTTGCAACCTCTTCCAAACAAACCATCAACATGAACCATTACCTGTTATGTAATGCGGTCGCTTTGACTTTAATGGGTTCTAGTAACTAGTACTGAACAACCTTGTGATGGCCAAGGAACTGTTGATAGTGTTTATATACGATTGTGAATCCTGTAAGAAGGAAGAAGATGATCCTCAAGATGCAATAATTTATTTTTACCCTACTTGGGTCAACAACGAGCAGAGACATGCTCTGTGTAGTCAGTTAATGGGTGTAACTCAATTTTGTTCTGCTTCCTTTTCTTTGCCGAGCATTATTTCTCTACAAAGTGGAAAATTCGCTCTGAAAAAGTTAGGCAGATATGCTTTGTGTGTGGGAACCGATAGGAACATTCCAGACCTAGTTTTGGAAACACGTGCAAGCATTTTATATAAGTTGCTCAGGCTGTTTCATTACAGCATCGAAAATTTACAAGAATCCTCATCAGATGAAGATATTTCAGATAAATTGGCTGAACTGCTCCAAGTTTACTTACCCATTTTACAATATGCCACCAACACGTTCGGAAACATACCAGTATTAAAAATTCCTAAGAGCACAAGCACCCTTTACTTGGAAGCGAGACAAACTTTGCAAGGTTTTCAAGAAATTCCTGGCGTTCTTGGCGGTGTGCTACTGTATCAGAACAAAGTTGTCGCCACTCAGTTAGATAAAGCATTTACAAAGCAGTTGGTGGTGACAGACCCCTATCGAATTAAGCTTCCAGCAGAATCAGTTGCCACTTCTTTCCATCTTCCATTTGGAGTTCAACTTATTAAGATATATGTTCCATCAGATGAAATTAAGAGGTTGCAAAAAGAAAACAACGAACTACTGGATGCTTTAAAAGATGTTGGAAATTGGAGGAAGGAACTGCAAAATTCTCAAAGCCAGAACCTAAATAAGAATAGTAGTGTGAAGCCTCCTGAATCTTTTGTATCGATGTGTGGCATGAAAAGAGATGTTTCAAGGATTTTCACCGTACTCGAGGAAGAAGGAAATGAAAATATTGAAACAAGAGATCAAACAGACATTCCTGATGTCGTCCGTGACGCTGTTAAAGCACGACATCTGGCCAGAATTGAGGCTGTAGTTCCTAATAACTTTATTATACCTGAATCCTCTGTGAAAGTTCGTTCAGCAATAACACCAGAAGATATTTCTACCAGAAAAATTCCTATTAGATATTATAGCATCGGTCTTCCACAGGTTAATTCTGATTGGTGTGATTCTCCGCCTAAGTCTAAGTCTCGGTATCCTAGACCATATTATAACACCATATGTGATCCCAAATATCCTCTATTTAAATATGATGGACTTCCTGCTTCAATTTCCCTTTCAGAATCACGAATTATTAAACAAGTCGAAGCACTGAAAGATGAAAAACACTCTATCAAAAGCTTAAGTATAGGAAAGCCTCTGAATGGGAATATCAAGATGGAAACAGCTGATAAACTGCCGGATGTAAAGCCACAACAAAAAATTAAATCTTCATTTACTTTACCTCTAAAATCTACTTCAGGTTCAAAAGGATCTCATGGAATTCCACTAACACCACTCCTGGCAAGGCTGTCCCTTTTGGCTGAAGAAGGGAGCACGATAGAAACCCCTTTAAAAACTCCATCAACTTTCGTGAACAAAATAAAGGATACCAAAAGTCAGTTTTCAGAAATCAATGATGTGCCCTCTACGAAAGAAAGACGTAACATTCCAATTAGCAATGATAATAATGAAGATCTTATTCCTTCAGTCCTTTACGTATTTGGTATCGGAAACACATCTGTGCTCCTTCTGATTGAGCCTGATGCGGCGGTTCAACCGGAAGTGATACACACATTGTGGGAAAGAAGCATGACTTCCTTGAAACATATGGATGTCCGTTTGAGCACCTGCTTAAATGCTACTGAAAGCAGCGGGAGTACTGACTACAGTTATTCACTGGTAGACTCAGCGTGGGGGCCGATAGAGAGGGGTGGCGCCTATCATCCCCATCAGCTGGAGCTCCTCACTATCCTACACCACACCCTCTCCAAGCGCCCGTTTATTACTGACATTCTTGTGAGATCCGAAGATACAATTACTTACGGATACCAATGTGCAGATACACAAGTGTTTTATCAAGAAAACAATTGTACAAATTCTATTGGTCTTCCAACTCCTTCTGATCTCATGGGAGTGATCCCTCTTAAAGCGAGGCGTATATTAGAACGTGACCATGGGATCATACTTCTCTAAAATAGCTTTGTGGTTCATCGGGTGTGCCATTGTAAAATCTTAATCTAGTCATATTGTCATCGCAAGTGTGATAGTAGTATTATCGCGATATTTATTTATTTTTATTTAGTCTTCCTATCTTTATATTAAGCTAGTTTTTATGGGATGCAAGTATATTATTTTATGAGACATTTCTTCGTTTGTTTCAATCTATATTGTTTAACTGTTTTGTTTTAGTTACCATGGTAAGTTGCACACCACTTACACACACATTAGTTATATTCCATTCCTAACTAATATGTGGTTATTGTTATTAACCAGATTATGATTTTGATAGAAGTGGTGAATTTTTGTCTAAGATTTAGTGATAGTTAAGTAGAATTCAAGAGGGATCCAGTCAATGATAGTAAAAAATTTTTTGATCCTACAAAACTCAATTTTGATACCATCAGCACTACAAAACAAATATGCCGAATGAATTGAAAAAAGAAGATAAATAATAAAGATCTGAGTTATCAGCTGTTAAAGTAACAAAGGACTAGCAGCGTTATCTGATGGTATACCAACTTACTAACCTTCTCCATGGTAACTAAAATTAAACTATATAGGAAGTATGTAGATAGTATCAGTTGATTCTCTTTCTTCCCACAGTGGTGCTTTTAAACATTTGGTAACCTCTCTTAAATAATTTTTTTTTTTTATATTTACAAAGATTTTAATTATCTTCTTAAATATAAAATTTTAGGAATAATACGTGCTTTCAATTACTACAAAATAAAG

Protein: RF -3: -192 -> -2339 (715 aa)

Comparison with *Halyomorpha halys*, PREDICTED: uncharacterized protein LOC106688690 - Sequence ID: XP_014288755.1

E= 0.0; bits= 1271

Query 1 MAKELLIVFIYDCESCKKEEDDPQDAIIYFYPTWVNNEQRHALCSQLMGVTQFCSASFSL 60

MAKELLIVFIYDCE CKKEEDDPQDAIIYFYPTWVNNEQRHALCSQLMGVTQFC++SFSL

Sbjct 1 MAKELLIVFIYDCECCKKEEDDPQDAIIYFYPTWVNNEQRHALCSQLMGVTQFCASSFSL 60

Query 61 PSIISLQSGKFALKKLGRYALCVGTDRNIPDLVLETRASILYKLLRLFHYSIENLQESSS 120

PSIISLQSGKFALKKLGRYALCVGTDRNIPDLVLETRASILYKLLRLFHYSIENLQESS

Sbjct 61 PSIISLQSGKFALKKLGRYALCVGTDRNIPDLVLETRASILYKLLRLFHYSIENLQESSI 120

Query 121 DEDISDKLAELLQVYLPILQYATNTFGNIPVLKIPKSTSTLYLEARQTLQGFQEIPGVLG 180

DEDISDKLAELLQVYLPILQYATNTFGNIPVLKIPKSTSTLYLEARQTLQGFQEI GVLG

Sbjct 121 DEDISDKLAELLQVYLPILQYATNTFGNIPVLKIPKSTSTLYLEARQTLQGFQEIHGVLG 180

Query 181 GVLLYQNKVVATQLDKAFTKQLVVTDPYRIKLPAESVATSFHLPFGVQLIKIYVPSDEIK 240

GVLLYQNKVVATQL+KAFTKQLVVTDPYRIKLPAE+V TSFHLPFGVQLIK+YVPS EIK

Sbjct 181 GVLLYQNKVVATQLNKAFTKQLVVTDPYRIKLPAETVVTSFHLPFGVQLIKVYVPSAEIK 240

Query 241 RLQKENNELLDALKDVGNWRKELQNSQSQNLNKNSSVKPPESFVSMCGMKRDVSRIFTVL 300

RL++EN+ELLDALKDV NWRKELQNSQSQN+NKNS VKPPESFVSMCGMKRDVSRIFTVL

Sbjct 241 RLREENHELLDALKDVSNWRKELQNSQSQNVNKNSCVKPPESFVSMCGMKRDVSRIFTVL 300

Query 301 EEEGNENIETRDQTDIPDVVRDAVKARHLARIEAVVPNNFIIPESSVKVRSAITPEDIST 360

EEEGNEN+ETRDQTDIPDVVRDAVKARHLARIEA+VPNNFIIPESS+K+R A+TP+DIST

Sbjct 301 EEEGNENLETRDQTDIPDVVRDAVKARHLARIEAIVPNNFIIPESSIKIRPALTPDDIST 360

Query 361 RKIPIRYYSIGLPQVNSDWCDSPPKSKSRYPRPYYNTICDPKYPLFKYDGLPASISLSES 420

RKIPIRYYSIGLPQVNS+WCDS K R PRPYYNTICDPKYP+FK+DGLPAS+SLSES

Sbjct 361 RKIPIRYYSIGLPQVNSEWCDS--PPKPRSPRPYYNTICDPKYPIFKHDGLPASVSLSES 418

Query 421 RIIKQVEALKDEKHSIKSLSIGKP----LNGNIKMETADKLPDVKPQQKIKSSFTLPLKS 476

RI+KQ E LK+EKH KSL+IGK +NGN K+ETADK P+VKPQQKIKSSFTLPLKS

Sbjct 419 RIMKQFEVLKEEKHLPKSLNIGKTQNGNINGNNKIETADKPPEVKPQQKIKSSFTLPLKS 478

Query 477 TSGSKGSHGIPLTPLLARLSLLAEEGSTIETPLKTPSTFVNKIKDTKSQFSEINDVPSTK 536

TSGSKG HGIPLTPLLARLSLLAEEGSTIETPLKTPS F NKIK+ K Q E NDV STK

Sbjct 479 TSGSKGPHGIPLTPLLARLSLLAEEGSTIETPLKTPSAFGNKIKEIKIQSGETNDVHSTK 538

Query 537 ERRNIPISNDNNE-DLIPSVLYVFGIGNTSVLLLIEPDAAVQPEVIHTLWERSMTSLKHM 595

E+ NIP +ND +E DLIPSVLYVFGI NTSVLLLIEP AVQPEVIHTLWERSMTSLKHM

Sbjct 539 EKMNIPGNNDKDEDDLIPSVLYVFGISNTSVLLLIEPTVAVQPEVIHTLWERSMTSLKHM 598

Query 596 DVRLSTCLNATESSGSTDYSYSLVDSAWGPIERGGAYHPHQLELLTILHHTLSKRPFITD 655

DVRLSTCLNATE SGSTDYSYSLVD+AWGPIERGGAYHPHQLELLTILHHTLSKRPFITD

Sbjct 599 DVRLSTCLNATECSGSTDYSYSLVDAAWGPIERGGAYHPHQLELLTILHHTLSKRPFITD 658

Query 656 ILVRSEDTITYGYQCADTQVFYQENNCTNSIGLPTPSDLMGVIPLKARRILERDHGIILL 715

ILVRSEDTITYGYQCADTQVFYQENNCTNSIGLPTPSDLMGVIPLKARRILERDHGIILL

Sbjct 659 ILVRSEDTITYGYQCADTQVFYQENNCTNSIGLPTPSDLMGVIPLKARRILERDHGIILL 718

Graphical representation

**No putative conserved domains have been detected**

**F-box protein 11 (FBX011)**

>TRINITY_DN17021_c0_g1_i1 length= 2886 nt

TTTTGCTTCCGCCTTTAGTAATGGCTGCTCGGTGATTGAGTTTGTGCTTCTCGATATAGTTTCTGATCTTAGTGCGTTTTAGTGTTAAAAATGCCGAGTGCTTCGTACGGCTCTTCTAGATATTATCCGAGAAGATCTAGACGTAAAGGAAACAGCAGAGTATCGGTTTCAAACCGAAATAATACTGCTGAAACTTGTGCTTCTGGAAGCAGTTCCAATGGGATTCCTGCTACCGTTATGAATTCGCAACCACCTCATAACCCTCCATATGATCTTCGCAGAAAATCACCACCTTATCCTGTTCTAGATACTGGATTCCCTAATCTAAGAAAAAGACCTCGCAGGTCATGTTCTGTGAATGCAGACATATTTTCACCACCGGCTGCTCATTATCTTCAGTATGAACTTCCTGATGAGGTGCTGCTGACAATATTTAGTTATCTTCTAGAAAAAGATTTGTGTCGTCTTTCACAAGTTTGTAAACGCTTTCAAACCATAGCCAATGATAATGGATTATGGAAAAGACTTTATCATAATATCTTTGAATATGATATCCCACTTTTTAATCCAGAGCCATGTGAATTTGAGTTCATACCTCCGAACGATTGTGAATATGCTAATCCTTGGAAAGAAAGTTTCCGTCAATTATATAGAGGGATTCACGTTCGACCAGGACAGGGTTCACGATATAAAGCAAGAGGTAGATCTCTAGCTCACTTCGATACAGTTCAGACGGCGTTAGATCATGCTGATGAAAATCCTACAGCTGGTGTTCCCTCAATTGTTTTTCTTCATGCTGGTACTTATAGAGGCGAATTTTTAGTCATAGATACTGATGTTGCACTTATAGGAGCAGCTCCAGGGAATGTAGCTGAATCAGTTATTTTAGAAAGGCAATCAGGTTCTACTATGATGTTCGGTGATGGCGCTAAGTCAGCTTATGCTGGCCATCTTACTTTGAAGTTTACTCCTGATGTTGCCTCTACAGTTCCGCACCATAAACATTATTGTTTGGAAGTAGGAGAAAATTGTAGCCCTACTATTGACCATTGTATTATTAGAAGCACTTCAGTCGTTGGAGCTGCAGTTTGTGTTAGTGGTCAAGGGGCTGCACCTATTATAAAGCACTGTGACATTAGCGATTGCGAAAATGTTGGACTTTATGTTACTGATTATGCTCATGGTACCTATGAAGATAATGAAATTTCTAGGAATGCACTTGCAGGCATTTGGGTTAAAAATTATGCCAACCCTATTATGAGAAGAAATCATATACATCATGGCAGAGATGTTGGAATCTTTACTTTTGACAATGGCTTAGGATTTTTTGAGGCCAATGATATTCACAATAATAGAATAGCTGGATTTGAAGTTAAAGCTGGAGCTAATCCAACTGTTGTCCAATGTGAAATTCATCATGGTCAGACAGGTGGTATTTATGTTCATGAAAATGGCCTAGGTCAATTTATTGACAACAGAATCCACTCAAATAATTTTGCTGGTGTTTGGATTACGTCGAATAGCAACCCTACAATCAGGAGGAATGAAATATATAATGGACAACAAGGTGGTGTATATATATTTGGAGAAGGGCGGGGTCTCATAGAACACAACAATATTTATGGTAATGCTTTGGCTGGGATTCAAATTCGTACCAACAGTGACCCAATTGTTAGGCATAATAAGATACATCACGGTCAACATGGAGGAATTTATGTCCATGAAAAAGGTGAAGGACTCATAGAAGAAAATGAAGTTTATGCCAACACTTTAGCTGGAGTTTGGATTACTACTGGGTCAACACCTGTTCTACGTAGAAATCGAATACATTCTGGAAAACAAGTTGGTGTATATTTTTATGATAATGGACATGGAAAGTTGGAGGATAATGATATATTCAATCACTTATACTCAGGAGTACAAATCAGGACTGGAAGTAATCCTGTTATTCGTGGTAATAAGATCTGGGGAGGTCAAAATGGTGGTGTTTTGGTTTATAATGGTGGTCTCGGCTTATTAGAACAAAATGAGATTTTCGACAATGCTATGGCTGGGGTGTGGATTAAAACTGACTCAAATCCAACCTTGAAACGCAACAAAATTTTTGATGGCCGAGATGGAGGAATATGTATTTTTAATGGTGGGAAAGGTATATTGGAGGAAAATGATATCTTCAGGAATGCTCAAGCAGGAGTTCTAATTTCTACTCAAAGTCATCCAGTCTTGAGACATAATAGAATATTTGATGGATTAGCTGCAGGCATTGAAATAACTTTAAATGCCACAGCAACACTGGAAAATAACCAAATATTTAACAATAGGTTTGGGGGACTTTGTCTTGCTACTGGTGTTAATCCTATTGTGCGATGTAATAAAATCTTTAGTAACCAAGATGCTGTAGAAAAAGCTGTTAAAAATGGACAATGCCTATATAAAATTTCTAGTTACACTTCGTTCCCTATGCATGATTTTTATCGTTGCCAAACCTGTAACACCACTGGCCGAAATGCAATTTGTGTGAATTGTATCAAAACATGCCATGCCGGCCATGAAGTAGAATTTGTAAGACATGATAGGTTTTTCTGTGATTGTGGTGCTGGTACGTTGTGCAATCAGTGTCAGCTGCAAGGTGAACCAGCTCAAGATACAGATACTTTATATGATTCTGCTGAACCAATGGAATCACACACACTAATGGTTAATTAAATTGAACTCTTTTCGTTACCAATATTTATTGTAATTTATCTCTTTCTATTGTTCCGAACTATCTTTTCATAGAAATAATTGAAATGTATGTTGATGAGTTGGTGATAATGTTTGGAGGATAAATATAGAATGTATACAAACACATCACATACATACATTATTTTTATTTTTACAATTAGG

Protein: RF -1: -91 -> -2706 (871 aa)

Comparison with *Halyomorpha halys,* PREDICTED: F-box only protein 11 - Sequence ID: XP_014287303.1

E= 0.0; bits= 1794

Query 1 MPSASYGSSRYYPRRSRRKGNSRVSVSNRNNTAETCASGSSSNGIPATVMNSQPPHNPPY 60

MPSASYGSSRYYPRRSRRKGN+RVSVSNRNNTAETCASGSSSNGIP TVMNSQPPHNPPY

Sbjct 1 MPSASYGSSRYYPRRSRRKGNNRVSVSNRNNTAETCASGSSSNGIPTTVMNSQPPHNPPY 60

Query 61 DLRRKSPPYPVLDTGFPNLRKRPRRSCSVNADIFSPPAAHYLQYELPDEVLLTIFSYLLE 120

DLRRKSPPYP+L+ GFPNLRKRPRRSCSVN DIFSPPAAHYLQYELPDEVLLTIFSYLLE

Sbjct 61 DLRRKSPPYPILENGFPNLRKRPRRSCSVNTDIFSPPAAHYLQYELPDEVLLTIFSYLLE 120

Query 121 KDLCRLSQVCKRFQTIANDNGLWKRLYHNIFEYDIPLFNPEPCEFEFIPPNDCEYANPWK 180

KDLCRLSQVCKRFQTIANDNGLWKRLYHNIFEYDIPLFNPEPC+FEFIPPNDCEYANPWK

Sbjct 121 KDLCRLSQVCKRFQTIANDNGLWKRLYHNIFEYDIPLFNPEPCKFEFIPPNDCEYANPWK 180

Query 181 ESFRQLYRGIHVRPGQGSRYKARGRSLAHFDTVQTALDHADENPTAGVPSIVFLHAGTYR 240

ESFRQLYRGIHVRPGQGSRYKARGRSLAHFDTVQTALDHADENPT+GVPSIVFLHAGTYR

Sbjct 181 ESFRQLYRGIHVRPGQGSRYKARGRSLAHFDTVQTALDHADENPTSGVPSIVFLHAGTYR 240

Query 241 GEFLVIDTDVALIGAAPGNVAESVILERQSGSTMMFGDGAKSAYAGHLTLKFTPDVASTV 300

GEFLVIDTDVALIGAA GNVAESV+LERQSGSTMMFGDGAKSAYAGHLTLKFTPDVASTV

Sbjct 241 GEFLVIDTDVALIGAAAGNVAESVVLERQSGSTMMFGDGAKSAYAGHLTLKFTPDVASTV 300

Query 301 PHHKHYCLEVGENCSPTIDHCIIRSTSVVGAAVCVSGQGAAPIIKHCDISDCENVGLYVT 360

PHHKHYCLEVGENCSPTIDHCIIRSTSVVGAAVCVSGQGAAP+IKHCDISDCENVGLYVT

Sbjct 301 PHHKHYCLEVGENCSPTIDHCIIRSTSVVGAAVCVSGQGAAPVIKHCDISDCENVGLYVT 360

Query 361 DYAHGTYEDNEISRNALAGIWVKNYANPIMRRNHIHHGRDVGIFTFDNGLGFFEANDIHN 420

DYAHGTYEDNEISRNALAGIWVKNYANPIMRRNHIHHGRDVGIFTFDNGLGFFEANDIHN

Sbjct 361 DYAHGTYEDNEISRNALAGIWVKNYANPIMRRNHIHHGRDVGIFTFDNGLGFFEANDIHN 420

Query 421 NRIAGFEVKAGANPTVVQCEIHHGQTGGIYVHENGLGQFIDNRIHSNNFAGVWITSNSNP 480

NRIAGFEVKAGANPTVVQCEIHHGQTGGIYVHENGLGQFIDNRIHSNNFAGVWITSNSNP

Sbjct 421 NRIAGFEVKAGANPTVVQCEIHHGQTGGIYVHENGLGQFIDNRIHSNNFAGVWITSNSNP 480

Query 481 TIRRNEIYNGQQGGVYIFGEGRGLIEHNNIYGNALAGIQIRTNSDPIVRHNKIHHGQHGG 540

TIRRNEIYNGQQGGVYIFGEGRGLIEHNNIYGNALAGIQIRTNSDPIVRHNKIHHGQHGG

Sbjct 481 TIRRNEIYNGQQGGVYIFGEGRGLIEHNNIYGNALAGIQIRTNSDPIVRHNKIHHGQHGG 540

Query 541 IYVHEKGEGLIEENEVYANTLAGVWITTGSTPVLRRNRIHSGKQVGVYFYDNGHGKLEDN 600

IYVHEKGEGLIEENEVYANTLAGVWITTGSTPVLRRNRIHSGKQVGVYFYDNGHGKLEDN

Sbjct 541 IYVHEKGEGLIEENEVYANTLAGVWITTGSTPVLRRNRIHSGKQVGVYFYDNGHGKLEDN 600

Query 601 DIFNHLYSGVQIRTGSNPVIRGNKIWGGQNGGVLVYNGGLGLLEQNEIFDNAMAGVWIKT 660

DIFNHLYSGVQIRTGSNPVIRGNKIWGGQNGGVLVYNGGLGLLEQNEIFDNAMAGVWIKT

Sbjct 601 DIFNHLYSGVQIRTGSNPVIRGNKIWGGQNGGVLVYNGGLGLLEQNEIFDNAMAGVWIKT 660

Query 661 DSNPTLKRNKIFDGRDGGICIFNGGKGILEENDIFRNAQAGVLISTQSHPVLRHNRIFDG 720

DSNPTLKRNKIFDGRDGGICIFNGGKGILEENDIFRNAQAGVLISTQSHPVLRHNRIFDG

Sbjct 661 DSNPTLKRNKIFDGRDGGICIFNGGKGILEENDIFRNAQAGVLISTQSHPVLRHNRIFDG 720

Query 721 LAAGIEITLNATATLENNQIFNNRFGGLCLATGVNPIVRCNKIFSNQDAVEKAVKNGQCL 780

LAAGIEITLNATATLENNQIFNNRFGGLCLATGVNPIVRCNKIFSNQDAVEKAV+NGQCL

Sbjct 721 LAAGIEITLNATATLENNQIFNNRFGGLCLATGVNPIVRCNKIFSNQDAVEKAVRNGQCL 780

Query 781 YKISSYTSFPMHDFYRCQTCNTTGRNAICVNCIKTCHAGHEVEFVRHDRFFCDCGAGTLC 840

YKISSYTSFPMHDFYRCQTCNTTGRNAICVNCIKTCHAGHEVEFVRHDRFFCDCGAGTLC

Sbjct 781 YKISSYTSFPMHDFYRCQTCNTTGRNAICVNCIKTCHAGHEVEFVRHDRFFCDCGAGTLC 840

Query 841 NQCQLQGEPAQDTDTLYDSAEPMESHTLMVN 871

NQCQLQGEPAQDTDTLYDSAEPMESHTLMVN

Sbjct 841 NQCQLQGEPAQDTDTLYDSAEPMESHTLMVN 871

Graphical representation


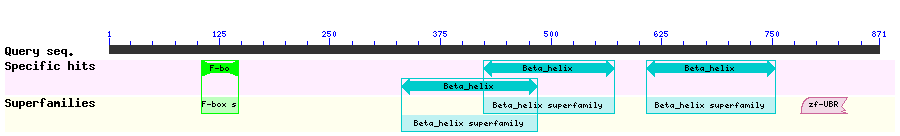


**Clathrin heavy chain**

>TRINITY_DN23162_c0_g1_i1 length= 7925 nt

CGTGATTAGACGAATTACCATAAGCATATGGGACATAATGTGGAGATGAATATACTATGAATGTACTTAAACTTTTGTACATGATAAAGAATCTAGCCAAGAAAATAAAATAAAAAATGGTTCAAAAAAGATCATATAATTAGAATTATTTTAAATATTTGAAATAGTTTTAAAGGTAGTTAATCTGATTATAAGTAATAAGTAGGGAGGATCAATACATTTTCTAAATGTAACCAACATATGTTGAAGTTGATTAAGAGTAAACCATAGTCAATCAGATGTTCTATTGACTGGATGAGATGTTAAAAAGCAAAAAAAAAGCAGTTCATTGATATATTAACCGATTTTTTTTGCAGATAATTAAAACCTAATAACTCTGAAACTAAAATTTTAGGTCCACATTTAGTCAGTTAGACTTTATATCCACATCTTAAGCCATGAAATAAAAATAAATAAATAATAGTAAAAATTATTAACAATACAGTTGTTCACTAAAATAAGGCCATTGACAAATGAACACTCAGCAGACTTTTGTTTCCTATAATCAAGAGAAATGTACTAATACCATATTAGACTAATATAACAATTACGAATGTAAAAGCCATAACTCTTTCAAAAAAAGTTATACAAATATAATAAACAGCAATTAATAATATATAAACAAGTGACCATTCAAAAACAAAAGACCTTTCATATCGTATTCACAAAAGGATATATATACTATGTATTTTATTTGGAAAATTAGAAATTTTAGTGATGGCAAGACCAGATAATTGTCTAAATAACTAAAAGCTATCATTTATAATCATTTTCCGAAAATGTTCCATCAAAATGCCCTTTAAAAAAATTGTGAGGGGATGGACAAACAAGAGATGATTTACCTCACCACATTGAATTGAAAGAAACAGGCTAGGGAAAGGATTTCTATGAATGCTCCACACCTTAAAATAACTATACTTAGAAATGTAAGATATTGCATATTTTAAAATGTAATATATTGAAAGCCATATTTACATATGGTGTTTAATATTTTGGCACATTGCTACATCCTCTGTATTCATATCAATTCCATAAGGTGATGTAATCATTTTTTAAATTGAGAAAAGTTTAGTTTAAGTCATATGGGATGACAAAGATAATGGATTTTAACTGAAACAAATAATCAATTAAAAATTTAATGAATGAAAATAAATTATTTTGTATCTTTTTTAATGAATTGTATTATTATAATTTTAAAATAATATGAGAGTAGTTACTTCCTGATGTTAATAATAAACATTATGCAGGTTTTATGATCTTCACTTCCATTAAAAGATGGAATAAATGTTCATTTAAATAAGGCACATGCCAATAAATCAAGTTACTCCGCCCACTGATAAACAAAATATAATGTGTATTTATTCTTTTTTTTTTTAATTAAATTATAAAATTAAAATAATTATTTAATTCAACTTTTGACTGAACCTCCATATCCAGCAACATTTATATACTATAAAAGAAAACAAAAACATAGAGAAAGCAATGTAACTGATATGATACAAAAACATATCTGGATATGAAGCCAGAGTGTTGCTCTACATAACTGATCTTTTTCTCTTAGGACTCATTACATCCACAAAATACAATAACCATTAACAATTACTTATAATTTTTAATACAATTTGATTCTAATAATAAAGAGTATATTTTTCAATCTTTACATATTTCCATAAGGTCTATGTATATGTTATACTATAGTGCATACTTTGCATGTTGGTTCTAATAATAATACACCTTAATAACATAAAGTTATTCAAGAGATAACTAATTAAAAAAAAGTAACAACATTGAAACACAAAATATAGATAAATTCATAATTTAAATATATAAACAATTTTTCCAAATGTAGCAGTAAATAATTTTACTAATAAAACAAATATTCACACAAGCTAACAAGATAATACCACAATAAATAGTTAGTGTTTCTATACTCTAGAACACATAATTACACACATACACATTATGTGTCAATGTGAGTCTATGTATGTGTATATGTGTGAAGAAATGGAATGCATAACATAAACTTAAATAGTTAACAAATTCATTAAATACTATAAAAAATATATGTAGTAACGGAATAGGACTATCCGTTAACTAATTATAGGTTAATGCTAACTTTACCATCCAGAGCTTAATAAGATACATGAATATCAACAGAAATATCATAACATACATATAGATTTTTACAAAGTTTATATGCGCATGATATTCATTAAAAGCACACTTATGAAGGCAGTATTGAACTTTATACAGTTTATTTTTAATGAAGACAGAACCACAGTATAAGAGGCCTTGGATTAGATGAATATGTTGAGTAAACAAAACCAACCTCTACATCAACTATAACTTGTATAGATTGCTTCTTAATATACATTTCAGTTTCAATTAGGCTGGATAGAGTTTAAACCATTCAGTTCAGTAGCTTAATACGGTGGTTTTGATATTACTGGTGATCACATTTGATATTGCCTACATACCATACCCTTGATAGGGCATGCCAGTGGTAAAAGTGTTAGGATAAGCTGGTGAAAAACCAGGTCCTATCATTCCTGGGCCAGCAGTTAACATTAGCTGGGGCTCAGGCATCATCATAGGTTTATGATCTTGTTCAGCTCTTTCTTCTAACCTTCTTGCTTCAGACTCTTCCAACTTATCAACTTTGGAAATATATTCTCTTGTCACTTGAATAATATATGGCATAGCGAAGTCCATGATATTATGTCGCCATGCAAGTTCAAGAATAACATCAGGATGAAGAAGATCATAACATTGGAACAAACAAGCAGCAAAACAGTCATGATTTCCTTTTTCTAAAAACCATGCCAAAAGTTCTTCAGCAACTTCTGAATTCTTTGATTCTGCAGCATATTCCATTGCATCCTTAAACAGCCTATCTTTCTTGCATAATTGAACACTTTGCTTCCATCGATTGTTTCCCTTATATAGGTAAGCAGCTATCCTCCTAAATTCAATTAATTCATGCTTTTCTAGCTTTTGGGCTAATACAATATTATCAAAATTATCAAACGCATCAATTGATGTTCTAACACCCTGAAAGTCTTCCTCTTCAATAAGAAGGTTATTCAGAGCTTCATTTATTGCTTTATTATTTAATGATTGTACTGAGCGAAGATAAGGTTTAACCAATTGCAAATGGTTAACCTTGGCGAAGAAATTAACTGCTCTAGTATGGTCCATTCTTGGTGCTAACACCAACAAAATATCATTTAAAAGGAGAGGTTTGTAATCCAGATAAAATTGTATAGCTTTGTAATATAATTCAATGTTGGCAACCTTGGTAATTATATCTTTAAAATGACCCTCCCGCCAAGCCTCAGTTGGGTGGTTCATCATCGCTGATACAGCATTATCATATTCTTCATATTTATCATACAGAAAAACTAGTTCTGCCCATAAATGAGCTTGTTCAGCAGCTCTTAAGACCTTAGGAATATTAACACGAGACCAAAATAATTCTAAATGTTCTCTCATTTTTGCTGGTTTGTATTTAGAATACAGAATAGCCAATTCTGTGAACATTCCCATATGAGCACGTTCTAATCCAAGGGCTGCCTCCAATAAATTTATAAGCTCTTCAAAATATCCTCGGTCCTGATAATAATTTATAAGGTCTTCTAGTTCATCTGCATGCACAACAATGTGTAAACCACACATCTGGGCTAGTCTGAATTCTTCACTATCCACGCAAGCGAAACAGACTTCTTTCCAGGTTCTTGTACTATTAGCTTTCCTAGCACTGTCCACAGCTCCTTGAAATTCTTTTAAATGAACCAGAGTTATAGCCAGTCTGGCAAAATTTGAAACATTGTTATAGAGCAGTTTAGCAGGTTCATACATCTTGTCATCAAAACATCTATCACCAATCTTTTGAATATCAGCATGATTTGGACCTGATATAAATTCCTCAAGATCAGCTAATCTGTTAGTGCGAGCATAAGCATAAATAAGCTCACTTTCAATATAAGATTCTCTAGCCTTTTTCCTGGCCATCTGTAAATAACGGACTAAATCCTCCCAGCTTTCTGTTTTATGAGCTGTTTCAACAACATCAACATAAGCAGAGGGGTCATCAGCTTTTATAAATGAATCAATTGCTTCTTTTACAAGACCATGTTGCAGTTGAGCTTTGGCTAATTGGCTCCACACAGCTGGCTCATTACAACGTTCCGCGAACTCATAGGCTCTATCAAGATTGTTTACATTTTCAATCAAAACCTGGATAGCAGAAGTGTTTACATCAAACTTTTTGAATATCGCAAATGCCTCTTCATACAACTGATAATTTATAGCAATATTTGCTATATCAGGTGCGTCGTAATTATCTAAACGGTTGATATATTCCATGACTCTTGATCGATCTGCTTTTATAGCAGTGAGAATGAGGAGATTTTGAAGATTCCGATGATCACTAAATATAGAATTATCAAGAACGATTTTCTCTAGAAGTTCAATAAGTTCATTAGGTAAATCTGCAGTCATGAAAGCCTTGACAGTCACACTAATGTCTTCAGGATCCTGAGTTTCTGAAAGAGCTGTCTGAACCACCTGATCGATTAATGGTCTCTTGTAAGGGTTGTTTTCGTTAAGAACTTCAGCCCATAATTCAGGATCCTTACGTCTAACCAGGTACCTGGCTTCTGATTTGAACAGAGAATTCTCATTGCAAACATTGATAAGCTCTCTGTCACATTGACCTCTTTCATAGGCTACACATGCCAAATGTGGATCCCGTTTTTCACAATACTTTCCAACTACACAACTATCATAATACTGATTTTCCCTTAAGAATCGTTCTGGGTTATTATTACTATCAATATATATCTTTGCTAGAGCATTATGAGTAGCAGGCTCAACGCAGCCTTCATGAACACGTGTTTCCAACCAAGGAAGTAAAAGTTTCAAACGATTCCGCTTCTCTACTTCTGCAACAAGCTCCTCTGTTGAAAACTGACCTCTAACAACCATCATCAAATTTTTAATAATATCTTCAGAGCAATCTACATCTAATAATCCTCCTATTACAACTGGCAACCGAGATGGATTGACCTTTTGTACATAAATTTCAATATATTTTTGCAAATTATTTCTATATAAATATAAAACCAAATCATGACAAAAATCAAACCGATCACAAACAATAATTAGCGGTAATTGATCTGTCAACTTTGCTTCCTTCAGAAAATTTTTCACTCTCTCTGGATTATAACAATTAGATTCACGGCAAATTCGTTCAACTTCTTTTATTTGTCCTGTTTTACAGGCTGCCTGAATGTATTTAAAATGAACTTCCTGATCTTGACTGAAATTAACTATGGAACCAAGGAAATAAAACAATCCTTCATAACTTTTGAATGATTCAAAAAGATCAATCAAAGCCTTTGTTGTCAACTGTTCATGGTATTTAGTAGCTATCTGAACACATATGTTCAAATTCTGTCGAATATTTGCAGTTAGCATAGCTTTAAGACATTCAAGAGAATCTTCAACGGAAAGAGTACCAAAGTATCCCACAAGCCAATCAGGCCCAAGGAGGTGAGTGTGAACTACAGCTCGTTTTATATCATAGAGATCTGTGTAATGTTCTAATGCTCTCTGAAGTAGACCAGCCTTCTCACAAAGTTGAGCAATGTGTGCTCTGTCATAATGTGTAAACATTTGGTTTCCAAGTATAGCATCAGCAACCTGAGGAGCAGAAATCAAATTCATTTCTAGAAGCCTAGTTTGTAATGGACCTTCAGAGGGCCGATTATTTTTTAGAGCATCCAAAAGAAAAGCTGTGCACTGCTGTACCATGTTCTGCTCCATCAGTATATCAACAATCTGATTTATATCAGCCAGTGGTTCTTCATCTTGAACAAGCATTTGAGCAAAGCTAACGCCTTGATCAGGATTGACACGCATTACATTACGGAGCAGAAAAATGTAGTCAGGTGAATAACCTACTTTTTTTGCATATAATACAATCTTTTGAAACTGTCCAGTTTCAGCAAAGGATTGTATAACCTTATTTGGAACATTAGCTCTTAAATAAATTGAGAGTGCAAGAGTAGGGTCAGTTGGTTTCACCAAATCGCCAAGTTCTTCAGAGGGTTCAAGTTTATCTTCTTTCATCCATTTTTCCAATAATTGCTTTCTTCCTTGAGCCAGAACAGGTCTGCATAATTCCAAGGATTCATATTTATTTAATTTACCCTGATCAAGTAATATTCCAAAATACTGAAGGAGAGGTGACGATTGGCCCTGAGGATTTGGTATCTGTTGAAACTGCTGAATGGTTTGTGGTGTTCTGAGAATTCCCTTAGGTGCATTTGCAGCCACTTTAGCAGCTTCAGCATACTGTCCATTTTGGAAAAGCAAATTGAATTTTTTAACAAACAAATCTTCAGCTCCAGCTAGATTATTTCTAACAGCCATACGTAAGGCAAGGTCAGGATTTTGTAATATATTGTTAATATATGGAATTATATGATCTTCTTCTACACTCACTGATAATACCTGTCCTTTTCTATTCACACCAATGATACCCCCAGTTGATTCATGAGGTGCAGTGACAAATATAGTATCAATACTAATACGGTTCATGTAGATACATGTAGCTGTTTCCAAATCATACAAATGAATGTACCCGTACTTTGTTATAAGATAGATTACATCGTATTTGGAGCTAACCTGCATAGCAACTGGAAAATCATTTTGTGCTTCTACTGGAAAAAATACATCAACCGCTTTTTTGGAGAATGGCTGGTTTCCTGTAGGAGGTTGTCCAACCTCAATAATATGAAGCTTTCCTCCTTGAAGAGTGCGAACAGCAAAGCAAAACAGGGTGGATACATCAGGGTTTCCTTCCATTTTAAACTGAGCAAATGATGCAGCATGACCTTCAATAGGCTGTGAGCATTTACGTTCAACTGAATATAATTGCATAGCCCCTACTACACGGTTTAGCTGAGCAGAGATTCCAACAAGTAAGAGCCAGGTTTGCTTAGGGTCTGTTCGGTAATTAATTATTTGACAACCATTCAGACTAGAATGTCTATCAAACATTTTCACTGGTTGGGAATCACCTTCCATGCTCCAATGATACACGGATGTTTCAGTAACTAAAGCAAGTGTATTAAGGGAAATCCATTTCCAAAAAACAACTTCGTCTGTCATGGTATGAGCTTTCATCTTACTCCTCATTTCTATATTAAAAATCTGGAGTGTTTTCTGAGTAGTGCTTCCATCAGAACCTGCTTTTCCCTTCAGTGCAATTACTTTACTAGCTGGATTCATAATAGCTGAATCTGCTGAAATAGGTCTTCTTATTAGATTTGCAGTATCATTTAAATCTATTATCACAACTTGAGAAGTTTCTCCAACTTTTTCTCTAACACAAATGAACTTGTCAGATTCCATTGTTAATGTATTAAAGCCTACACTAGTTGGGTTTATTCCTACGCTTGAAAGCTGTAAATGTTCTTGGAAACGTATCGGTAGTATTTGAGACATCTTCGTAATTATAAATTAGCTTCAACTAAATGTTTAATCCTCTTTAAATTATCCTATTGACCCCTCAGGAAAACTAAACAATCACTTCCACCTCCAACACCATGGATATTATCAATCTTTAATTCAACATGGCTACACCAGTTATACAAAAAAATAACCTTACGCCCACATTTATTAAATACTTTCAAATAGAGAGTAAGTTTAAAAACTTCCTATGAACAGATAACAACCGCCTATTCAATTCAACAAGAATTAACGGGATGGAATATGTCCAAGCAAGTAGAGCAGTATTCTGTGGAACTACTTTAGATAAGGGAAAGAGGTATCATGCTCATCCATTGCCACATAATCAAGAAGTTACACTATGAACAGCG

Protein: RF -3: -7551 -> -2509 (1680 aa)

Comparison with *Halyomorpha halys*, PREDICTED: clathrin heavy chain - Sequence ID: XP_014287090.1

E= 0.0; bits= 3477

Query 1 MSQILPIRFQEHLQLSSVGINPTSVGFNTLTMESDKFICVREKVGETSQVVIIDLNDTAN 60

MSQILPIRFQEHLQLSSVGINPTSVGFNTLTMESDKFICVREKVGETSQVVIIDLNDTAN

Sbjct 1 MSQILPIRFQEHLQLSSVGINPTSVGFNTLTMESDKFICVREKVGETSQVVIIDLNDTAN 60

Query 61 LIRRPISADSAIMNPASKVIALKGKAGSDGSTTQKTLQIFNIEMRSKMKAHTMTDEVVFW 120

IRRPISADSAIMNPASKVIALKGKAG DG+TTQKTLQIFNIEMRSKMKAHTMTDEVVFW

Sbjct 61 PIRRPISADSAIMNPASKVIALKGKAGPDGTTTQKTLQIFNIEMRSKMKAHTMTDEVVFW 120

Query 121 KWISLNTLALVTETSVYHWSMEGDSQPVKMFDRHSSLNGCQIINYRTDPKQTWLLLVGIS 180

KWISLNTLALVTETSVYHWSMEGDSQPVKMFDRHSSLNGCQIINYRTDPKQTWLLL+GIS

Sbjct 121 KWISLNTLALVTETSVYHWSMEGDSQPVKMFDRHSSLNGCQIINYRTDPKQTWLLLIGIS 180

Query 181 AQLNRVVGAMQLYSVERKCSQPIEGHAASFAQFKMEGNPDVSTLFCFAVRTLQGGKLHII 240

AQLNRVVGAMQLYSVERKCSQPIEGHAASFAQFKMEGNPDVSTLFCFAVRTLQGGKLHII

Sbjct 181 AQLNRVVGAMQLYSVERKCSQPIEGHAASFAQFKMEGNPDVSTLFCFAVRTLQGGKLHII 240

Query 241 EVGQPPTGNQPFSKKAVDVFFPVEAQNDFPVAMQVSSKYDVIYLITKYGYIHLYDLETAT 300

EVGQPPTGNQPFSKKAVDVFFPVEAQNDFPVAMQVSSKYDVIYLITKYGYIHLYDLETAT

Sbjct 241 EVGQPPTGNQPFSKKAVDVFFPVEAQNDFPVAMQVSSKYDVIYLITKYGYIHLYDLETAT 300

Query 301 CIYMNRISIDTIFVTAPHESTGGIIGVNRKGQVLSVSVEEDHIIPYINNILQNPDLALRM 360

CIYMNRISIDTIFVTAPHESTGGIIGVNRKGQVLSVSVEEDHIIPYINNILQNPDLALRM

Sbjct 301 CIYMNRISIDTIFVTAPHESTGGIIGVNRKGQVLSVSVEEDHIIPYINNILQNPDLALRM 360

Query 361 AVRNNLAGAEDLFVKKFNLLFQNGQYAEAAKVAANAPKGILRTPQTIQQFQQIPNPQGQS 420

AVRNNLAGAEDLFVKKFN+LFQNGQYAEAAKVAANAPKGILRTPQTIQQFQQIPNPQGQ+

Sbjct 361 AVRNNLAGAEDLFVKKFNMLFQNGQYAEAAKVAANAPKGILRTPQTIQQFQQIPNPQGQT 420

Query 421 SPLLQYFGILLDQGKLNKYESLELCRPVLAQGRKQLLEKWMKEDKLEPSEELGDLVKPTD 480

SPLLQYFGILLDQG+LNKYESLELCRPVLAQGRKQLLEKW+KEDKLE SEELGDLVK TD

Sbjct 421 SPLLQYFGILLDQGQLNKYESLELCRPVLAQGRKQLLEKWLKEDKLECSEELGDLVKQTD 480

Query 481 PTLALSIYLRANVPNKVIQSFAETGQFQKIVLYAKKVGYSPDYIFLLRNVMRVNPDQGVS 540

PTLALS+YLRANVPNKVIQ FAETGQFQKIVLYAKKVGYSPDYIFLLRNVMRVNPDQGVS

Sbjct 481 PTLALSVYLRANVPNKVIQCFAETGQFQKIVLYAKKVGYSPDYIFLLRNVMRVNPDQGVS 540

Query 541 FAQMLVQDEEPLADINQIVDILMEQNMVQQCTAFLLDALKNNRPSEGPLQTRLLEMNLIS 600

FAQMLVQDEEPLADINQIVDILMEQNMVQQCTAFLLDALKNNRPSEGPLQTRLLEMNLIS

Sbjct 541 FAQMLVQDEEPLADINQIVDILMEQNMVQQCTAFLLDALKNNRPSEGPLQTRLLEMNLIS 600

Query 601 APQVADAILGNQMFTHYDRAHIAQLCEKAGLLQRALEHYTDLYDIKRAVVHTHLLGPDWL 660

APQVADAILGNQMFTHYDRAHIAQLCEKAGLLQRALEHYTDLYDIKRAVVHTHLLGPDWL

Sbjct 601 APQVADAILGNQMFTHYDRAHIAQLCEKAGLLQRALEHYTDLYDIKRAVVHTHLLGPDWL 660

Query 661 VGYFGTLSVEDSLECLKAMLTANIRQNLNICVQIATKYHEQLTTKALIDLFESFKSYEGL 720

VGYFGTLSVEDSLECLKAMLTANIRQNLNICVQIATKYHEQLTTKALIDLFESFKSYEGL

Sbjct 661 VGYFGTLSVEDSLECLKAMLTANIRQNLNICVQIATKYHEQLTTKALIDLFESFKSYEGL 720

Query 721 FYFLGSIVNFSQDQEVHFKYIQAACKTGQIKEVERICRESNCYNPERVKNFLKEAKLTDQ 780

FYFLGSIVNFSQDQEVHFKYIQAACKTGQIKEVERICRESNCYNPERVKNFLKEAKLTDQ

Sbjct 721 FYFLGSIVNFSQDQEVHFKYIQAACKTGQIKEVERICRESNCYNPERVKNFLKEAKLTDQ 780

Query 781 LPLIIVCDRFDFCHDLVLYLYRNNLQKYIEIYVQKVNPSRLPVVIGGLLDVDCSEDIIKN 840

LPLIIVCDRFDFCHDLVLYLYRNNLQKYIEIYVQKVNPSRLPVVIGGLLDVDCSEDIIKN

Sbjct 781 LPLIIVCDRFDFCHDLVLYLYRNNLQKYIEIYVQKVNPSRLPVVIGGLLDVDCSEDIIKN 840

Query 841 LMMVVRGQFSTEELVAEVEKRNRLKLLLPWLETRVHEGCVEPATHNALAKIYIDSNNNPE 900

LMMVVRGQFSTEELVAEVEKRNRLKLLLPWLETRVHEGCVEPATHNALAKIYIDSNNNPE

Sbjct 841 LMMVVRGQFSTEELVAEVEKRNRLKLLLPWLETRVHEGCVEPATHNALAKIYIDSNNNPE 900

Query 901 RFLRENQYYDSCVVGKYCEKRDPHLACVAYERGQCDRELINVCNENSLFKSEARYLVRRK 960

RFLRENQYYDSCVVGKYCEKRDPHLACVAYERGQCDRELINVCNENSLFKSEARYLVRRK

Sbjct 901 RFLRENQYYDSCVVGKYCEKRDPHLACVAYERGQCDRELINVCNENSLFKSEARYLVRRK 960

Query 961 DPELWAEVLNENNPYKRPLIDQVVQTALSETQDPEDISVTVKAFMTADLPNELIELLEKI 1020

DPELWAEVLNENNPYKRPLIDQVVQTALSETQDPEDISVTVKAFMTADLPNELIELLEKI

Sbjct 961 DPELWAEVLNENNPYKRPLIDQVVQTALSETQDPEDISVTVKAFMTADLPNELIELLEKI 1020

Query 1021 VLDNSIFSDHRNLQNLLILTAIKADRSRVMEYINRLDNYDAPDIANIAINYQLYEEAFAI 1080

VLDNSIFSDHRNLQNLLILTAIKADRSRVMEYINRLDNYDAPDIANIAINYQLYEEAFAI

Sbjct 1021 VLDNSIFSDHRNLQNLLILTAIKADRSRVMEYINRLDNYDAPDIANIAINYQLYEEAFAI 1080

Query 1081 FKKFDVNTSAIQVLIENVNNLDRAYEFAERCNEPAVWSQLAKAQLQHGLVKEAIDSFIKA 1140

FKKFDVNTSAIQVLIENVNNLDRAYEFAERCNEPAVWSQLAKAQLQHGLVKEAIDSFIKA

Sbjct 1081 FKKFDVNTSAIQVLIENVNNLDRAYEFAERCNEPAVWSQLAKAQLQHGLVKEAIDSFIKA 1140

Query 1141 DDPSAYVDVVETAHKTESWEDLVRYLQMARKKARESYIESELIYAYARTNRLADLEEFIS 1200

DDPSAYVDVVETAHKTESWEDLVRYLQMARKKARESYIESELIYAYARTNRLADLEEFIS

Sbjct 1141 DDPSAYVDVVETAHKTESWEDLVRYLQMARKKARESYIESELIYAYARTNRLADLEEFIS 1200

Query 1201 GPNHADIQKIGDRCFDDKMYEPAKLLYNNVSNFARLAITLVHLKEFQGAVDSARKANSTR 1260

GPNHADIQKIGDRCFDDKMYEPAKLLYNNVSNFARLAITLVHLKEFQGAVDSARKANSTR

Sbjct 1201 GPNHADIQKIGDRCFDDKMYEPAKLLYNNVSNFARLAITLVHLKEFQGAVDSARKANSTR 1260

Query 1261 TWKEVCFACVDSEEFRLAQMCGLHIVVHADELEDLINYYQDRGYFEELINLLEAALGLER 1320

TWKEVCFACVDSEEFRLAQMCGLHIVVHADELEDLINYYQDRGYFEELINLLEAALGLER

Sbjct 1261 TWKEVCFACVDSEEFRLAQMCGLHIVVHADELEDLINYYQDRGYFEELINLLEAALGLER 1320

Query 1321 AHMGMFTELAILYSKYKPAKMREHLELFWSRVNIPKVLRAAEQAHLWAELVFLYDKYEEY 1380

AHMGMFTELAILYSKYKP KMREHLELFWSRVNIPKVLRAAEQAHLWAELVFLYDKYEEY

Sbjct 1321 AHMGMFTELAILYSKYKPGKMREHLELFWSRVNIPKVLRAAEQAHLWAELVFLYDKYEEY 1380

Query 1381 DNAVSAMMNHPTEAWREGHFKDIITKVANIELYYKAIQFYLDYKPLLLNDILLVLAPRMD 1440

DNAVSAMMNHPTEAWREGHFKDIITKVANIELYYKAIQFYLDYKPLLLND+LLVLAPRMD

Sbjct 1381 DNAVSAMMNHPTEAWREGHFKDIITKVANIELYYKAIQFYLDYKPLLLNDMLLVLAPRMD 1440

Query 1441 HTRAVNFFAKVNHLQLVKPYLRSVQSLNNKAINEALNNLLIEEEDFQGVRTSIDAFDNFD 1500

HTRAVNFFAKVNHLQLVKPYLRSVQSLNNKAINEALNNLLIEEEDFQGVRTSIDAFDNFD

Sbjct 1441 HTRAVNFFAKVNHLQLVKPYLRSVQSLNNKAINEALNNLLIEEEDFQGVRTSIDAFDNFD 1500

Query 1501 NIVLAQKLEKHELIEFRRIAAYLYKGNNRWKQSVQLCKKDRLFKDAMEYAAESKNSEVAE 1560

NIVLAQKLEKHELIEFRRIAAYLYKGNNRWKQSVQLCKKDRLFKDAMEYAAESKNSEVAE

Sbjct 1501 NIVLAQKLEKHELIEFRRIAAYLYKGNNRWKQSVQLCKKDRLFKDAMEYAAESKNSEVAE 1560

Query 1561 ELLAWFLEKGNHDCFAACLFQCYDLLHPDVILELAWRHNIMDFAMPYIIQVTREYISKVD 1620

ELLAWFLEKGNHDCFAACLFQCYDLLHPDVILELAWRHNIMDFAMPYIIQVTREYISKVD

Sbjct 1561 ELLAWFLEKGNHDCFAACLFQCYDLLHPDVILELAWRHNIMDFAMPYIIQVTREYISKVD 1620

Query 1621 KLEESEARRLEERAEQDHKPMMMPEPQLMLTAGPGMIGPGFSPAYPNTFTTGMPYQGYGM 1680

KLEESEARRLEERAEQDHKPMMMPEPQLMLTAGPGMIGPGFSPAYPNTFTTGMPYQGYGM

Sbjct 1621 KLEESEARRLEERAEQDHKPMMMPEPQLMLTAGPGMIGPGFSPAYPNTFTTGMPYQGYGM 1680

Graphical representation


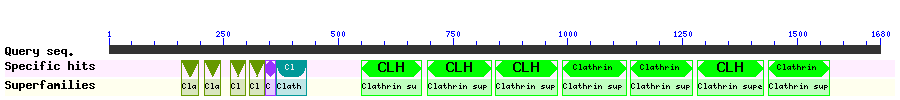


**Epsin 2 (Epn2)**

>TRINITY_DN26893_c1_g1_i1 length= 3139 nt

GTCCATGATATGGTATAAATTTTTTTCAAAATATATAGTTTTTAAAAATTTAAGTTTGCACATTGAATATTTAGATATCTCACTGAAATTTCAGGTTAAATACGTTTCTTAATCAGGAGTCAACTGAATAAAGTAAGAAGGGTTCCACTATCACATATAAATTTAAGGAAAAAGTTTCGGATCAGCGTTAAATTAATAGTACAAATAACAATAGATTGATCCAATATTTAAAGCGAAAACAAACTTGGGACATGAGAATACAATTAGTCAACAACAAATCATGATTAAAAGCACCTTTACATTTAAACAAAAAAAATTATCAATAATTCTTTCGAAAGATGTTGAAAAGAATCCAAGAAACATTATAACGGTAAATTGATATTAGCTGTCGACAAGATTATTCTCAAGTCTCAAGGACAATTATCAACAATTGATTCAACAAAAGGTATCTTCCTGTTGTTAACTAATATTATCATATAAAAACGAGGTTACAAGTCCCGACTGGCCAGTAAGTAGCATTCGTTTAATAACTAGAGCCAATATAGCATCAAGCTGGCATAAGATTTTTTTTAACTGGCATTCGCTGAAGGTGGTGAGATATCTTTCAGAGGTTATAAAACACTGCATCGCGGCATCATTCACAGCTAAGGTCTTTAGCTGTTACATGATATGCTCAGATTTTATTGCCTATTTTATGCAAATATTACATTTTTGCAAAAAATTCAAGTATTACTTATATTTTTTTAAGATTAAATTCATAAAAAAGTTATATAAAAACTAAATTTCTTAACTTCATAGCAATTATGAAATCACTATCGAGGCATGGGAGAAATATCACGTTTCGTCGAATAGAAACTGAACGTTATTCAAATGAAAGTAGTATAGAATATCTTGCTAACTCACAGTATCAGATTTATTATTCAGCCGTAAGCTGCAGAAGGCTATGTACATAAAATTTCTCTATCCCTATCTATATAAATATATAAGCTCAAGTGGACGAAAATATATCTAAATATAAATATATATTATGTATTTATAATTTGGTGTTTTGAATATGATATTCTCGTATAATTTAAAATAAACACTTTCAAAAACGAAAAAATTAGGAGCTGGTGAGCATATATTCCATCGGAAAAGATAAATTAGTCATAATATAATTCATAGCTCTTGACCTTTGGTTCTATTAAAAAACAAAACAATAAATCTAACATGTACACCATGAATAAATAAAAATGTAAAAATAATACTGCACCATTCTAAAAGAGCTAATTAATACAACTCAAGTATAAACGGGCGACAAACATTAGATTTAATATTTAAATTACGAGGCACAGAGATTGATAATACGTGTTGCGAGTAACCAAAGAAAACAGATCGTTTCCCTGACTTTGCTTGTTTTTTATTGCTTTCACTCAAGTGGATAGTAGCCTATTCTTTCATGTTTTAAAAAAAGGGGACAATTGCTGATTGTAAAGAGTTTTTTTTTTTTGTTTTTTTTAGACAGTTGACCGTTTAACGCAATCAGAAACAATCCTAAGACAAGAAAGGATTAGGCTGAGGTACAAGAGGTTGAGATATTGCCTGAGGTGCAAAAGCGTGAGTGTTCCGAACTGGCTGCAATGGAGCACCCACCGGTGAGAAAGGGTTAGCTGCAACTGGTCGTATTGGTTGTTGCTGACTTGGTTGAGGCTGAGTCGGTTTAGTGACAAGATTGTCGAGATTTACCAATCCAGAATTCTCACCTAAGAACGCATGAGGACTTCTTCTTTTGGCAGATGCCAACCCATTCCTATCGTTTAAGCTATCTCCAAGGCCTCTCATGTCAAAAGGATTAGGACTAATGCAAGTTTTATTATTATTGTTGATATTGTTCCTATTTGTAATAATGTCAAAATCATCAAGGTCGTTTGATGGAGATGATAACGCAGGACTGGGCTCTGTAGCGGGAGACCATGGATCGTTATGAGGTTTTACTGCTTTCCATGGGTCCACTGAGGCAGCCTCGTTTGGCTTTGGAGGTGCAGCGTATACGGGGGCGGGGGACCAAGGGTCGGCAACAGAAGGGTTAGTTGCTGTCGAAGATGAAACACTCCAAGGATCAACCTGGGGTCGGGACGGAGGGATAGGCATCCCCCATGGGTCCTGGAGCGGGACTGGGGCAGACGTGGTTGGGCCAGTATCAATAGGTCCGAGGTTCACATCGAGAAGGTCTAGCATGTGACTGCTACTCGGTTCTTTAGGGACCTCTTGCTGATGTTGCTTGAACTCATTCTGGCTCTGTGAAATAGCCAATTGGAGTCGAACATCATCACTTCTCCTTTTCTGTTCTTCCTGTTCTGCCTCCTCTCTTGACATTGCAAGGGCCAATTGCAATTGGAGTTCTTCTTCCCCTGCTGTTGTTGGCCTGGCGCACATTATGTCAACTTTACTAGGAGTTTCAACATCACTGGAGCTCCAAGCTGATCTCGATTGAAATGTAGGGCTCGAAGGAGATGCACCATCTAAAGTCGTATCACTGCCGAAACCCGAAGCTGTCTGAGCAAACCGTTCTTTAGCTTTAAGCGCTCGTGCACGTTCGTTACGTAACCTTTCCTCATCTTTTAAAAGTGATACAAGCTGCTTTGCTTTTTCTCGTACATTGAGTCCTTGGTCTTTACCCTCATCAGAATACTGAAAATCCCGAAGAGTTTGGATTGCAAAGATATTTTCTTTACACTGCTGACCAACCTTTTCAGAACCAGTTTTAATAAGATACTCCAAGAGTAATAAAGCTTTATACACGTGCCTCCAATTTCTACCATGATCATTTAAGCGTTTCCATATCATTTGCATAATTTCTGTAAATGCAACAACATTATAAGTTAAGTCAGCGATTTCAGACATCAGTGTACTACTGGGACCCCATGGATCATTGCTGGTCGCTTCCCGTACTTTTATCTGAGCATCAGAATAATTATGAGCTAGATTTTTAATGTTTCGCCGAAGCCCTGCCACATTCACCTGCATGTCTTCACGCCGCCGCATCTTGGATAATAAGATTTTAACCTTTTCTAAGTTTTTAAAAACAATTTACACACATAAAGATAAGAATGACAGTAGTCATTCAATTTCGACAAATCAGCAAAGCCAGGCGATATGG

Protein: RF -2: -3024 -> -1534 (496 aa)

Comparison with *Halyomorpha halys*, PREDICTED: epsin-2 isoform X5 - Sequence ID: XP_014270392.1

E= 0.0; bits= 900

Query 1 MRRREDMQVNVAGLRRNIKNLAHNYSDAQIKVREATSNDPWGPSSTLMSEIADLTYNVVA 60

MRRREDMQVNVAGLRRNIKNLAHNYSDAQIKVREATSNDPWGPSSTLMSEIADLTYNVVA

Sbjct 14 MRRREDMQVNVAGLRRNIKNLAHNYSDAQIKVREATSNDPWGPSSTLMSEIADLTYNVVA 73

Query 61 FTEIMQMIWKRLNDHGRNWRHVYKALLLLEYLIKTGSEKVGQQCKENIFAIQTLRDFQYS 120

FTEIMQMIWKRLNDHGRNWRHVYKALLLLEYLIKTGSEKVGQQCKENIFAIQTLRDFQYS

Sbjct 74 FTEIMQMIWKRLNDHGRNWRHVYKALLLLEYLIKTGSEKVGQQCKENIFAIQTLRDFQYS 133

Query 121 DEGKDQGLNVREKAKQLVSLLKDEERLRNERARALKAKERFAQTASGFGSDTTLDGASPS 180

DEGK+QGLNVREKAKQLVSLLKDEE+LRNERARALKAKERFAQTASGFGSDTTLDGASPS

Sbjct 134 DEGKNQGLNVREKAKQLVSLLKDEEKLRNERARALKAKERFAQTASGFGSDTTLDGASPS 193

Query 181 SPTFQSRSAWSSSDVETPSKVDIMCARPTTAGEEELQLQLALAMSREEAEQEEQKRRSDD 240

SPTFQSRSAWSSSDVETPSKVDIMCARPTTAGEEELQLQLALAMSREEAEQEEQKRRSDD

Sbjct 194 SPTFQSRSAWSSSDVETPSKVDIMCARPTTAGEEELQLQLALAMSREEAEQEEQKRRSDD 253

Query 241 VRLQLAISQSQNEFKQHQQEVPKEPSSSHMLDLLDVNLGPIDTGPTTSAPVPLQDPWGMP 300

VRLQLAISQSQNEFKQ EV K+ SHMLDLLDVNLGP+D GPTTSAPVPLQDPWGMP

Sbjct 254 VRLQLAISQSQNEFKQQHHEVQKDQGGSHMLDLLDVNLGPLDGGPTTSAPVPLQDPWGMP 313

Query 301 IPPSRPQ-----------VDPWSVSSSTATNPSVADPWSPAPVYAAPPKPNEAASVDPWK 349

IPPSRPQ VDPWSVSSSTATNPS ADPWSPAP +APPKPNEAASVDPWK

Sbjct 314 IPPSRPQTLDLRLNNWSSVDPWSVSSSTATNPSAADPWSPAPTASAPPKPNEAASVDPWK 373

Query 350 AVKPHNDPWSPATEPSPALSSPSNDLDDFDIITNRNNINNNNKTCISPNPFDMRGLGDSL 409

VKP NDPWSPATEPSPALSSP NDLD+FDIITNRNNINNNNKTCISPNPFDMRGLG+SL

Sbjct 374 TVKPQNDPWSPATEPSPALSSPLNDLDEFDIITNRNNINNNNKTCISPNPFDMRGLGESL 433

Query 410 NDRNGLASAKRRSPHAFLGENSGLVNLDNLVTKPTQPQPSQQQPIRPVAANPFSPVGAPL 469

NDRNGL S+KRRSPHAFLGENSGLVNLDNLVTKPTQPQ +QQQP+RPVAANPFSPVGAPL

Sbjct 434 NDRNGLGSSKRRSPHAFLGENSGLVNLDNLVTKPTQPQQNQQQPLRPVAANPFSPVGAPL 493

Query 470 QPVRNTHAFAPQAISQPLVPQPNPFLS 496

QPVR+T AFAPQAISQPLVPQPNPFLS

Sbjct 494 QPVRSTPAFAPQAISQPLVPQPNPFLS 520

Graphical representation


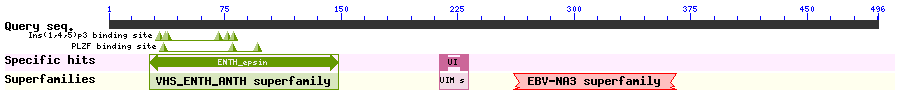


**Gap Junction protein (Innexin2)**

>TRINITY_DN17230_c0_g1_i1 length= 1670 nt

GGTACTTACAGACCATCAGTGCAGGTTTGGCTGAGAGAGTGTTTGTGTGTGTTCTAGTGGCTCCAGCATGTTTGACGTGTTCGGCTCCCTCAAGGGGCTTCTCCGGATAGACACAGTATGCATCGACAACAACGTTTTCCGGCTGCATTACAAAGCTACCGTCATCATCCTGGTGGCCTTCTCCCTGTTGGTCACCTCCAGGCAATACATCGGGGACCCTATCGACTGCATCGTCGATGACATCCCCCTCAGCGTCATGGACACCTACTGCTGGATCTATTCGACTTTTACGATTCCGAATCGACTCAGAGGTACTGTGGGAAAAGATGTCGTTCAGCCGGGAGTAGCAGGCCACGTAGAAGGTGAAGATGAAGTTAAGTACCATAAATATTATCAGTGGGTTTGTTTCGTTTTGTTTTTCCAAGCGATTCTGTTTTATGTGCCTCGTTACCTTTGGAAAACCTGGGAAGGAGGTAGGATCAAGATGTTGGTACTGGATCTGAACTGCCCCGTCATTAATGAAGAATGCAAAGCCGACAGAAAGAAACTGCTGGTCGATTATTTCACCTCTAATCTTCATACTCAAAATTTCTACGCTATAAGATTCTTTATTTGCGAAGCACTGAATTTCATCAATGTCTTGGTACAGATTTACTTCATGGATTTCTTCCTTGATGGAGAGTTCACGACTTATGGGTCAGATGTCGTCAGGTTCACTGAAATGGAACCGGAAGAAAGAGAAGATCCCATGTCGAGAGTTTTTCCAAAAGTTACCAAATGTACCTTCCACAAATACGGTCCATCAGGATCGGTGCAGAAATTCGACGGCTTGTGCGTCCTCCCATTGAACATCGTCAACGAAAAGATATACGTCTTCCTATGGTTCTGGTTCATCATCCTGACCATCCTATCTGCATTGGCACTGGTATACCGCGTTGCAGTGGTTTGTGGACCCCAGCTTCGCCTCTACCTGCTCCGCGCCAGGTCCAGGTTGTCGCCCCAGGGCCAAATAGAGACAATCGCCAAAAACTGCCACCTTGGTGACTGGTTTGTTCTATACCAGTTGGGTAAGAATATTGACCCTCTCGTATTTAAAGAACTGATAGCAGATCTGGCGAAAAAGTTTGAAGGTAAAGAGTCAGTTTAAGATTTTTGTATTTTTGTAATAATAGTGCCTTAACTATGCCCTCTGGATTAGGGCTCTGTCCAGACTTTCAGCTCTTGAAATTATGTGTTCAAAAAGTGCATTAGTTTCAATTTTGTTATTTGACAACATCCTTCTAATGGGGGCTTCAGGCACTTATGTGATTTTGCTTATTATTATATTTTGTTTATTATTATCAAATGAAGTAATACTCCTCCATAATTGCACTGCCAAAGCTAAATGTCTTTCATTCATAGCTAGGAAGTAGCACATTATGAGAGCAAACGCACACATTTTATTTACATTGTTTGAAGACAATAATATACATTCCAAATTGCAACAATTGGCAATATTTAGGTGTTCAAATCGTAAAATAATGCTCATGTTACTTTTAAAAGACTGTATTAAACTTTAACTGCTTCCTTATTGTTTATAAAATTAATGTTTTTATTTACATGTAAAGATTTTAAGATATAGTAGAAGTTTATGTAATGTATCACTTCTATATTTAAATTAAGGTGCCGAG

Protein: RF -2: -68 -> -1147 (359 aa)

Comparison with *Halyomorpha halys*, PREDICTED: innexin inx2 - Sequence ID: XP_014292574.1

E= 0.0; bits= 736

Query 1 MFDVFGSLKGLLRIDTVCIDNNVFRLHYKATVIILVAFSLLVTSRQYIGDPIDCIVDDIP 60

MFDVFGSLKGLLRIDTVCIDNNVFRLHYKATVIILVAFSLLVTSRQYIGDPIDCIVDDIP

Sbjct 1 MFDVFGSLKGLLRIDTVCIDNNVFRLHYKATVIILVAFSLLVTSRQYIGDPIDCIVDDIP 60

Query 61 LSVMDTYCWIYSTFTIPNRLRGTVGKDVVQPGVAGHVEGEDEVKYHKYYQWVCFVLFFQA 120

LSVMDTYCWIYSTFTIPNRL GTVGKDVVQPGVAGHVEGEDEVKYHKYYQWVCFVLFFQA

Sbjct 61 LSVMDTYCWIYSTFTIPNRLSGTVGKDVVQPGVAGHVEGEDEVKYHKYYQWVCFVLFFQA 120

Query 121 ILFYVPRYLWKTWEGGRIKMLVLDLNCPVINEECKADRKKLLVDYFTSNLHTQNFYAIRF 180

ILFYVPRYLWKTWEGGRIKMLVLDLNCPVINEECKADRKKLLVDYFTSNLHTQNFYAIRF

Sbjct 121 ILFYVPRYLWKTWEGGRIKMLVLDLNCPVINEECKADRKKLLVDYFTSNLHTQNFYAIRF 180

Query 181 FICEALNFINVLVQIYFMDFFLDGEFTTYGSDVVRFTEMEPEEREDPMSRVFPKVTKCTF 240

FICEALNFINVLVQIYFMDFFLDGEFTTYGSDVVRFTEMEPEEREDPMSRVFPKVTKCTF

Sbjct 181 FICEALNFINVLVQIYFMDFFLDGEFTTYGSDVVRFTEMEPEEREDPMSRVFPKVTKCTF 240

Query 241 HKYGPSGSVQKFDGLCVLPLNIVNEKIYVFLWFWFIILTILSALALVYRVAVVCGPQLRL 300

HKYGPSGSVQKFDGLCVLPLNIVNEKIYVFLWFWFIILTILSALALVYRVAVVCGPQLRL

Sbjct 241 HKYGPSGSVQKFDGLCVLPLNIVNEKIYVFLWFWFIILTILSALALVYRVAVVCGPQLRL 300

Query 301 YLLRARSRLSPQGQIETIAKNCHLGDWFVLYQLGKNIDPLVFKELIADLAKKFEGKESV 359

YLLRARSRLSPQGQIETIAKNCHLGDWFVLYQLGKNIDPLVFKELIADLAKKFEGKESV

Sbjct 301 YLLRARSRLSPQGQIETIAKNCHLGDWFVLYQLGKNIDPLVFKELIADLAKKFEGKESV 359

Graphical representation


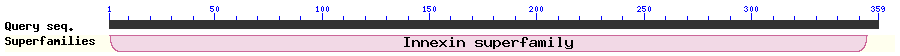


**Data S2:** Sequences of *E. heros* core machinery proteins.

**Dicer-1**

>TRINITY_DN22802_c0_g1_i1 length= 3568 nt

GTGACAATTACTGTCATATAAAATCCTCAGATTAGTGAAAATCACAAATATAAATGACTTCTTTGTTATAAATAATTTAATGAAGTTCTAGTGCTTGTTTTTCATATCATGACAAACCCTGAGGACTTTTAACAATGACTGACTAGGATGTTCAGTTAACTTTCTAGCTAATAGTCCAATCTTCAATTGTTTTAAAGCACATTTGGCTGCGGTACATTTAGCTATTCTATAGTTTCGCCCTATTCCTTTGAATGTTCCTTTGCCGAATACTTCAACCGTAACTCTGACCCTTCTTCCATCTGCTAGTTTTTCAGGTTTACTGAATTTAGCAGTCTCTGGTTCCAATTCTAATAATTCTCTGATGGGAGATTTCGGAACTTTTGTACTAAATTGTTCTATTTCTTTTTTCATCATTCTGTAGTACACTTTCCAAACAGTATCCAAAGACATTCCGCTGTCGAGAAAAATTGCTCCAGCAACTGATTCAAAAACGTCACCAAGCGCTTTTGGTACTTCAACATCTTCGGCCTCTTCTCCAACTAAATAGTACTCTTCGCTAATGAGATGACTATTTTCTTCTTGAATAGTAACAAATCTTTGAATAACTTCCGCTAGGCCTGGTGAGAGATGGCGGAAGTATTTGTGGAGTCCATATTTAACAGCTAGAGAAGCAAATATAGTGTTGTTAACTAATGCAGACCTTAAATCTGTTAAAGCTCCAGGACTGTGCGCACGCTTGTCCTCAAAAAGATGCCGTGTTATAAGATAATCTAAAACTGCATCACCCAAAAACTCAAGCCGTTGATAACAATCTGTGACTCTATTAGGAGAATATGAAGCATGGCTCATAGCTTGTAGAAGATATGACTTGTCCCTGAATGAATAATGCAGAGATGCTTCCAAAGCTTCAAAGCCAGCCATGAGAAATGCTAGCTCCCCTTCTGGGTCATCGACGTGTCGAAGCAAAGGTGAAATAGGTTCCGAAAGACTAGTAAAGTTTACCACTGTATTCTCATCAGTTTTAATACTTCCGACACGCTGAACTAAACTGGATTCAATAGTCGTATTTATTTCCTCTCGGGGTAAAACTTTTATTCCAAGCCATGACATAAACAACAATGCTCCCCTCGGACCACATTCTATCAAGTAAGCACCAATCAAGGCCTCTACACAATCAGCAATGCTTTTGTCAGGAATGGAGTGCTGGGTAACTAGATTATACGGGAGAGTAGCTTGAGGCTCGGAATCAAGTAACCTTTGCTCAGCGTCACGAGGGACTAAATAACATGGCGGTAACCAGTTATCATGTGGCTCAAATTTACTAGCTATCATACTTTCGCCAAATACCTTCTTTTGTCCCAGCCTATACAGCTTTAAATTACTGACCTGTTTGGAACGAAGATGACTAAGTTTTCCTTCGTGTATATTTTCATGAGTACAATATAAGTAAGTCGTTATAGCATATTTAAGAAAGGAATCGCCAATAGTTTCCAAACGCTCTAGATTGATGCCATCATTGGCGTTGGACATAGTCAAAGCTTGGAGAAGAATGGAAGGACTGGGACCTGGATGACCGTCTAATTCTGGCTGCTGATCAAAACTGAATGCCATATTCTCTTCTGTTTCCTCATTATTAATAGGTTCAGCAGAATACTGGTATGGTAGAGACTTTTTCTTTGAAGATGTGGGTAATATACCTGAAGGCATACCATAGCTGTTGACTTTGAAATCAAATTCCTTCGTCTCTCCTGGGGCAGTTTCTTGTTTTTTACTATCCTCCTTATTAATTATGAACTGTTCCTCTTTTGTAATATATTTTTTTTGTGTTTTTATTATTTCCATTTGTTCCTCAGTCTGTATCTGAAATTCTTCAGTCTCCTTATCGATAGCTTCCTTGACATCGGGAATTAAGTCTTCAGTATCCCATGTCCATAACTCAAGTTCTAATTTCTTGCTATCATCTGTTTCTTTCTTTGGTTCGGGTTCATTTTCTTCAATCGCTTCTGCTAAGTAATCTCCCCTGAATGTGATTTTCAATTTTCCAGCACCTCCATCGGAAGAATCAGAGGTTTCAGATTCTAAATATATTTCACTAGAATCATCAGATTCACAATCGGAGCTGTAGTAATTAAATGATTGGTTTTCACCAACATTCCAGCTGGTTGGAGATCCGTAGCGCACCATTGAGAGCTCTGGAGGGAGGTCCTTTTGAAACTCTTCACCAAGTTGAGCCATGTCATTGGACCATGTTCCAATTTCCATCCAATCACCCTTATCTTTATCTTCTTCTTCATTGCCAGAATCCTTATTTGTTTCTTCATCAGAATCTTCAGCATCAGACTCTTCTTTTTTCTTTGTGGCATCAGACTCTTCTTTTTTCTCTAAATCATCAGAATCATCCTTTTTTTTTGTATCTGATTCACTTTTATCATCTTCTGGCAAATCATCTTTGGCCTCTAAACCTTCATTTTTAACAGCAATTTCTTTTCCATTTGTTCCCTTGTTTTCAATCTCTTGTTTTGTTTTTTTCAAGACATCAGCCAAACTCCAGCCAAAATCCAATGGCGCCCACTCGAAATCTTTAGTAAGAGTATTTAGACCAAGCCCAATTTCCTCAGCGACTTTTGTCCGAATTTCATCCGCAAGAAGAAGAGCATTAAGCCTGTATAGAATACAAGGAAGACAGACAGCCTGCCTCCAAAGAGAAGCCGGAACAACATGCACTTCACAAAGTTCACCAACAAGAATTTGCTTTTGTTCTAAGTTTTCCCTTTTAGCTCTCTTTGTTTCCTCACTGGATGTAGGTAAAGCAACACCTTTACGGTTTACATACCTGGGTGTAAGGAAATTCAATCGCGCAGAAGTATGGTCGACATCAAGGAGAGGTTGTGATGTGTTTTGAATCTGTATACCGTACTTTTTAAGGTAATAATCCTCAAATGTTTTATAAAAACTTCCAGGAAAACCAGATTTTGGGTTGAGATGATAACATATTTCAGCAACATAGAAATACTGTGGTTGGTCTTGATTTCGATACCATGGCATCACGACAGCATCATTGTACTTCTCTGGATCAAATGAGAAATCTTTTCTTTCTTCATCGGAAATTAAATGAGGACATTCGTCTTTCCTTTTGTAAATTACCTCAAGGAAATCCCAGTCCACTTGACTTACCCCATTTTCATCTTTTTTTGTAGGAACAATGAAAAATGAATTTTCTTTAGCTTTTGGATCGAAACTCATCAAATATTTCTGTAGGCGAAGGACTGAAGTGAATATAAAATTTAAAAAGGTAGCTATTGTTTCAACATGCCTCTCAGATAAATTTATTGTGCTGGAACATAGTTTTAAACTGACTTGAACTTCACCTGATCTAGTGAATATTGGAAAAGGACAAACCTTGGGAATTTCTTTAAGCGTAAGAATCCCAAAAGCTTGTGGAGCAAGTTCAGGAGGATGAAGTTTTCTACCTCTTGTATTTTGTTCTTCCGGAAGCGGGCAGGTGAGAACCATATCTATGTTATATAAATAACAATCGACTCCTGCAACTGGGCGGCAATTATTGAGG

Protein: RF -2: -3513 -> -88 (1141 aa)

Comparison with Nezara viridula, PREDICTED: Dicer-1-PA- Sequence ID: AVK59457.1

E=0.0; bits= 2109;

Query 1 MVLTCPLPEEQNTRGRKLHPPELAPQAFGILTLKEIPKVCPFPIFTRSGEVQVSLKLCSS 60

MVLTCPLPEEQNTRGRKLHPPELAPQAFGILTLKEIPKVCPFPIFTRSGEVQVSLKLCSS

Sbjct 948 MVLTCPLPEEQNTRGRKLHPPELAPQAFGILTLKEIPKVCPFPIFTRSGEVQVSLKLCSS 1007

Query 61 TINLSERHVETIATFLNFIFTSVLRLQKYLMSFDPKAKENSFFIVPTKKDENGVSQVDWD 120

+INLSERHVETIATFLNFIFTSVLRLQKYLMSFDP+AKENSFFIVPTKKD+NG++QVDWD

Sbjct 1008 SINLSERHVETIATFLNFIFTSVLRLQKYLMSFDPQAKENSFFIVPTKKDDNGINQVDWD 1067

Query 121 FLEVIYKRKDECPHLISDEERKDFSFDPEKYNDAVVMPWYRNQDQPQYFYVAEICYHLNP 180

FLEVIYKRKDECPHLI+DE+RKDF+FDPEKYNDAVVMPWYRNQDQPQYFYVAEICYHLNP

Sbjct 1068 FLEVIYKRKDECPHLITDEDRKDFTFDPEKYNDAVVMPWYRNQDQPQYFYVAEICYHLNP 1127

Query 181 KSGFPGSFYKTFEDYYLKKYGIQIQNTSQPLLDVDHTSARLNFLTPRYVNRKGVALPTSS 240

KSGFPGSFYKTFEDYYLKKYGIQIQNT+QPLLDVDHTSARLNFLTPRYVNRKGVALPTSS

Sbjct 1128 KSGFPGSFYKTFEDYYLKKYGIQIQNTTQPLLDVDHTSARLNFLTPRYVNRKGVALPTSS 1187

Query 241 EETKRAKRENLEQKQILVGELCEVHVVPASLWRQAVCLPCILYRLNALLLADEIRTKVAE 300

EETKRAKRENLEQKQILVGELCEVHVVPASLWRQAVCLPCILYRLNALLLADEIRTKVAE

Sbjct 1188 EETKRAKRENLEQKQILVGELCEVHVVPASLWRQAVCLPCILYRLNALLLADEIRTKVAE 1247

Query 301 EIGLGLNTLTKDFEWAPLDFGWSLADVLKKTKQEIENKGT-NGKEIAVKNEGLEAKDDLP 359

EIGLGL TLTK+FEWAPLDFGWSLADVLKK KQEIENK T N KEI K+EGLEAK+ L

Sbjct 1248 EIGLGLITLTKEFEWAPLDFGWSLADVLKKNKQEIENKETSNIKEITDKDEGLEAKELL- 1306

Query 360 EDDKSESDTKKKDDSDDLEKKEESDATKKKEESDAEDSDEETNKDSGNEEEDKDKGDWME 419

DDS E TKKKEESDA+D+D+ETNKDS NEE+DKDKGDWME

Sbjct 1307 ------------DDS------ENKIVTKKKEESDADDTDDETNKDSANEEDDKDKGDWME 1348

Query 420 IGTWSNDMAQLGEEFQKDLPPELSMVRYGSPTSWNVGENQSFNYYSSDCESDDSSEIYLE 479

IGTWSNDMAQLG+EFQKDLPPELSMVRYGSPTSWNVGENQ FNYYSSDCESDDSSEIYLE

Sbjct 1349 IGTWSNDMAQLGDEFQKDLPPELSMVRYGSPTSWNVGENQPFNYYSSDCESDDSSEIYLE 1408

Query 480 SETSDSSDGGAGKLKITFRGDYLAEAIEENEPEPKKETDDSKKLELELWTWDTEDLIPDV 539

SETSDSSDGGAGKLKITFRGDYLAEAIE+NEPE KKE ++S KL+LELWTW+T+DLI DV

Sbjct 1409 SETSDSSDGGAGKLKITFRGDYLAEAIEDNEPEAKKENNNSMKLDLELWTWETDDLITDV 1468

Query 540 KEAIDKETEEFQIQTEEQMEIIKTQKKYITKEEQFIINKEDSKKQETAPGETKEFDFKVN 599

KEAI ETEEFQ QTE+QMEI+K+QKK I KEEQF++NKED KKQE GE K FDFKV

Sbjct 1469 KEAILTETEEFQKQTEKQMEILKSQKKLIKKEEQFLVNKEDDKKQEIVSGEMKNFDFKVR 1528

Query 600 SYGMPSGILPTSSKKKSLPYQYSAEPINNEETEENMAFSFDQQPELDGHPGPSPSILLQA 659

SY MPS I+P SSKKKSL Y Y AE INN+E EE++AFSFD QPEL+GHPGPSPSILLQA

Sbjct 1529 SYNMPSDIIPKSSKKKSLSYHYPAETINNQEIEEDIAFSFDHQPELEGHPGPSPSILLQA 1588

Query 660 LTMSNANDGINLERLETIGDSFLKYAITTYLYCTHENIHEGKLSHLRSKQVSNLKLYRLG 719

LTMSNANDGINLERLETIGDSFLKYAITTYLYCTHENIHEGKLSHLRSKQVSNLKLYRLG

Sbjct 1589 LTMSNANDGINLERLETIGDSFLKYAITTYLYCTHENIHEGKLSHLRSKQVSNLKLYRLG 1648

Query 720 QKKVFGESMIASKFEPHDNWLPPCYLVPRDAEQRLLDSEPQATLPYNLVTQHSIPDKSIA 779

QKKVFGESMIASKFEPHDNWLPPCYLVPRDAEQRLLDSEPQATLPYNLVTQHSIPDKSIA

Sbjct 1649 QKKVFGESMIASKFEPHDNWLPPCYLVPRDAEQRLLDSEPQATLPYNLVTQHSIPDKSIA 1708

Query 780 DCVEALIGAYLIECGPRGALLFMSWLGIKVLPREEINTTIESSLVQRVGSIKTDENTVVN 839

DCVEALIGAYLIECGPRGALLFMSWLGIKVLP+EEI+TT + SLVQRVGSIKTDEN VVN

Sbjct 1709 DCVEALIGAYLIECGPRGALLFMSWLGIKVLPKEEIDTTTDPSLVQRVGSIKTDENIVVN 1768

Query 840 FTSLSEPISPLLRHVDDPEGELAFLMAGFEALEASLHYSFRDKSYLLQAMSHASYSPNRV 899

FTSLSEPISPLLRHVDDPEGELAFL+AGFEALE+SLHYSFRDKSYLLQAMSHASYSPNRV

Sbjct 1769 FTSLSEPISPLLRHVDDPEGELAFLLAGFEALESSLHYSFRDKSYLLQAMSHASYSPNRV 1828

Query 900 TDCYQRLEFLGDAVLDYLITRHLFEDKRAHSPGALTDLRSALVNNTIFASLAVKYGLHKY 959

TDCYQRLEFLGDAVLDYLITRHLFEDKRAHSPGALTDLRSALVNNTIFASLAVKYGLHKY

Sbjct 1829 TDCYQRLEFLGDAVLDYLITRHLFEDKRAHSPGALTDLRSALVNNTIFASLAVKYGLHKY 1888

Query 960 FRHLSPGLAEVIQRFVTIQEENSHLISEEYYLVGEEAEDVEVPKALGDVFESVAGAIFLD 1019

FRHLSPGLAEVIQRFVTIQEENSHLISEEYYLVGEEAEDVEVPKALGDVFESVAGAIFLD

Sbjct 1889 FRHLSPGLAEVIQRFVTIQEENSHLISEEYYLVGEEAEDVEVPKALGDVFESVAGAIFLD 1948

Query 1020 SGMSLDTVWKVYYRMMKKEIEQFSTKVPKSPIRELLELEPETAKFSKPEKLADGRRVRVT 1079

SGMSLDTVWKVYYRMMKKEIEQFSTKVPKSPIRELLELEPETAKFSKPEKLADGRRVRVT

Sbjct 1949 SGMSLDTVWKVYYRMMKKEIEQFSTKVPKSPIRELLELEPETAKFSKPEKLADGRRVRVT 2008

Query 1080 VEVFGKGTFKGIGRNYRIAKCTAAKCALKQLKIGLLARKLTEHPSQSLLKVLRVCHDMKN 1139

VEVFGKGTFKGIGRNYRIAKCTAAKCALKQLKIGLLARKLTEHPSQSLLKVLRVCHD+KN

Sbjct 2009 VEVFGKGTFKGIGRNYRIAKCTAAKCALKQLKIGLLARKLTEHPSQSLLKVLRVCHDLKN 2068

Query 1140 KH 1141

KH

Sbjct 2069 KH 2070

Graphical representation


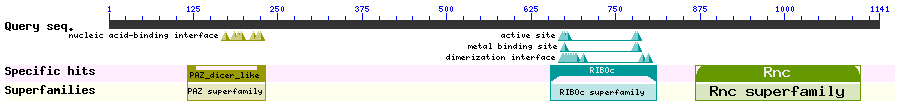


**Argonaute 1- isoform 1**

>TRINITY_DN26672_c0_g1_i1 length= 4207 nt

GTCAAATCCATTTTTTGTAAGTAAATTAAAAGATAACATCCATAAGAAGGAAATTAAAAATAACAATTCTTTTTGTTTAGTTCCGCAGATTAAAATTTTTAAGTACGACTAAGTGAGCAGACTAAATAACCACAGACCTGTTTTAACTTAAAGCTGACTTTAGTACATCGCCTGGAGACATTGACTACGCAACCATTTTCTATATTTATCCTCAAAAAACAAAATATTAAAGCTATCAACTAACAGCTGACTTGGAGACCTTGGCTTAAGAGAGAAATAAAGAGAGAAATAGAAAATAAATATATATAAAAAAAAACAAGCATCATAGAACATGTTACAACTTATCTACTAATTAAAATAGTTATAGTGTATTATCTCTCTTGACAGCCCTATTCCTATTTACATGCCTAGACTTGTGCACCAGCTAAAGAAGACTTACATATATACGGCAGCTTTCCTCAATTAGTTTTCTTCCAACTAGTGAAATTTTACTTAAGAAATATGAATCAGTGTTTTAATTTGACTCTTATGAAAAATATAACTATCACTGCTTTTTTTTGTAGGTACCTTAATCATAAAATAATTATCTTAATATTAAAAATCTTATTAAATAGGACGACCAACACAGCGATAAAATTCCTTCGAAAGAAGGTGAATGATTAAAGACACCACTGAAAAATAGCTGGCTGGTTCCTATAATCCCACATGGCAGGAAGTTCTTTCGCAATTTAGGTTATGCGAAGTACATTACTTTCTTCGTATCTGCATGAACCGTAATAGCTCGTGCCATTGCACCCGGTGTCCGGTCTTCACTACAGCCTGACTGGTGAGAACCCTCACCGCTGTCATGCTCTTTCTCAACAAGATGATACCTCGCCCTAAATGCAACAAGATGAGCGTAATAGGCAGGGGCAGGTATAGATACTGATCGTGTACACCTCACATAGGTATGGCACAACTGGTAAGTTAAGCATTGCAGTTCATCTGAATCGAAATGGTTGTCATCCCATAAAACATGATAATGACTCGGCCGACTAGTACCCTGAATACCTTGGTGGCTGCAAAGATAGAAGTCAAACTCAGTCGGGTGGGTTATCCCCACATCGACTGTTGTACCAGCAGGTATATTTCCAGACTTACCACTTTGCTCTTTTTTGTCAGCACAGAATAACCTTGTATGATGCCTCTTTTGCACTACAATAAATGTTATTCCAGGTTTATAATCTCCTTCGAGTTTAATACAAGCTTCCCTAATAGCTGTAAGTTCATGTTGTAAAACATGTAAGAATTGTCCTTCTGAAACACCATCTCTATACAGAATAATCCTATGAGGCTTGTACCCACCGGTACTTTTGTAAAACATTATCAGAAGCTCCCTGACCATGGAGGATAATTCTTGAATAATTTCTTGACGATGTTGTTGAACCCTAACAGTAGCAGCATATCGACTGGGATGTGCATCCATAGATCCAACGACGGCTGCTATCGAAGGTTTTTTGTTGTCTCCAGCAGGTGGGTGAGTAACATCGGCTCCAAGGAATATTACAGGTTCATTGAAAACCTTTGGCCTAATGCTGGGAACAAGAATGCTGTTAATGCCACCAAGCTTAACATTGATTTTGAGACAAAGATTGGAGAGTGTCTGTGGAGAAGTTTTATTTACATTTTTTGCCTGAACACATTGTGTTGCCATACCTAAGACTGTATCTCCAACCCTCTTCACTTCAGCATATACAGGTGTTTTCCCTGGAAGCACCACTACAACAAGCTGTAATGCCTGGAAGGAAGACTTCAAGTAACGGAACATGGGCTCCACTTGATCTGGTCCAGTAGCATATTTACAGAAGCATGGTTGGCCAATTATTGGCATTCCAGCATCATTACTAATTTTCTGCAATTGTTGGGTAAAATTCCTGAGAGCATCCTCTCTAACTGTCCTTTGAGGTGCGAAACAAGCAATGGCCCACACTCTTATTTCAACACCAGTGAAAAATTGCTTGCCCCGCATATCCCATACACCTTGATTAGGAAGGGCTTGCTGTTTTGCAGACAGCATGCCTTGGTATTCGAGCTGACTAGGCAAGTTAGGAGCTCGCCCACCATACTGAAGCTTCGGTGGCGGCAAAACCCTCCCTCGAACTTCCATCATATTATTGGAAATCGCCAGTCCGAACTCTTGAACATAGGCGTCATTATTGAAATCCGCTCTCCTCACGAGATTGTTGATCTCTCTTTCCCGATCAGGTGCTGACCTAGCAGTAGCTTTGATCATAGTTGAGGTCTGCATGTCAGTAAGCTTTTTAATGCATCTCTGACCAGCCACAATATTGCAAACCTCTAGGGGAAGATATGTATGCTTGTGCTCCTGCCCAACTTGAAGACAAGGTAAATGTGGATACCTTAATTTCATTTTGTATTTGTCTAAGAAATACTTAGCAACTGTGCATTCCACAGTTTGTCCGTTCTCTAATTGTAACGGGAACGACTGCATTTGAGCAGGTCTTCTTGTAACATTGCAGACTCTGTATTTCCTCCTCATTGTTCCACAATGTGTAATTTCAATTTTCAACCCTTTAATTTCTTTAGTAAACTTAACCCTCTGAGAATCTGTCAAGGGTTTTCGTTGTTCATTTATGTCCCTAATGTCTAACACTTCACACATAAACTCGATTACGGGCTGGGCTTTATAAAAAGCTGTGGCTGACACATCGATATTAAGCATCATCTTCCATTGTGAAGGTCTAACAGATTGATGAAAACCAAACCATACCTCACGACCACCGCCAAGTGGATGGTAGTAACCATCAGGACTAGAGAAGAATGATCTTCCTACTGGAGTATAAGTCATTGAAGGCAAATGCCTCATTACTACATCCAGTGCCATTATAGCATCATATGGAATCTGTCGAGTCCTTCCTTCCAATGCTTCTTCTAAGGCAAACAACGAAACTTGCGCCATCCATTTGATGGCCACTCTAAACACACGATCTTTTCCTTCACCTGGCAATGTTACCTCGAGTTCCATCCTATCATTGCCAATTGGAAGTGGATCTCTAGTGTAAAGATTACTTCTTCCATCAAAGACAGGTTTCAAGTTACCAAAGATTTTTGAATAGGCATGGACCATTGTTTCAATGATTTCCCTGTTGACCTTGCGAGGACACTTATCAGGTTGTATACTAATTTCATAATGATGTACGAAACCACGTGGCATAGTTATTTGGAAATGGTTCGCACGAAGCACTATAGGTCGACCTTCTCGGCCGAGATTAGGCCTTCGAGGACAGGTAAACATAGGTAAATCAGGAGGTTGGTGAGGAGGCTGAGGTGGAAGAAGAGCTATCGCTCCAGGGGGGACGCCAACTGGACCTGGTGGGGCCCCACCTCCCGCAGGAGCTCCGGGGGGCCCTACAGGAGGAGCTGGTTGTGCAAAGGATTTTCCAACCATCGAAGCTGTGTCCCTATGGATATGTGCCACTTCTTGGAATGGTACTGCCATTTCTTTATTTGCACTTAGTTACTCAGTTTTACACTTGATTATCAAGTTTTCCCTCATCATCATAGTTAATATAGTATGTTCTCCTAACTTTAGTAATTTTAAGTGTTTTATGAAGCACTGATATCATTATGCTTTGAATTGTATGTGAAGATTTCATCATATTATGATAGGAGTGTTAGAGTGGATTGTTTGACAATACCAAACACCCTTGCATCACAATTATCCTGTAATATCTACGGCAAACTAACTGCTATGTTGACAAAATGTACTGTATCCATTGAGATGATTCTAGCTGGGAACTACGAGGCAGATCGCAAGTTGAGTGTAAAATCGATAAGTTAACAGCACCATTCTAGGAATCTACGTTCTGTTTTTCATTCATTTCAGACTCGGCAATTCGTTTGAATGACAATTCGGAGTAAGTTGAAGGGATGCAGAATTATACATCTTGCAATTATATTAATCCAACAACTCTCGAACCGCACTGTTTACAAACTTTTATTTATCAATGACACTCGTCGATTGTAATAACGATAGGTGGTTGATTTCACAGATCGGAAATGCCAGTCTTTAGCATAAACAAAGATACCTGTCCAAAGTAAATTCAAGGTTAATATTGAATTTTTGAAATATTAAAACTATCCAACCCGGGTTGGATTGAAATTGCTGTTTGGGGGCGGAGCTTGGAGGATAGCACGTGGTACCGTG

Protein: RF -1: -3499 -> -734 (921 aa)

Comparison with Nezara viridula, PREDICTED: Argonaute-1-PC- Sequence ID: AVK59466.1

E=0.0; bits= 1924

Query 1 MAVPFQEVAHIHRDTASMVGKSFAQPAPPVGPPGAPAGGGAPPGPVGVPPGAIALLPPQP 60

MAVPFQEVAHIHRDTASMVGKSFAQPAPPVGPPGAPAGGGAPPGPVGVPPGAIAL+PPQP

Sbjct 1 MAVPFQEVAHIHRDTASMVGKSFAQPAPPVGPPGAPAGGGAPPGPVGVPPGAIALIPPQP 60

Query 61 PHQPPDLPMFTCPRRPNLGREGRPIVLRANHFQITMPRGFVHHYEISIQPDKCPRKVNRE 120

PHQPPDLPMFTCPRRPNLGREGRPIVLRANHFQITMPRGFVHHYEISIQPDKCPRKVNRE

Sbjct 61 PHQPPDLPMFTCPRRPNLGREGRPIVLRANHFQITMPRGFVHHYEISIQPDKCPRKVNRE 120

Query 121 IIETMVHAYSKIFGNLKPVFDGRSNLYTRDPLPIGNDRMELEVTLPGEGKDRVFRVAIKW 180

IIETMVHAYSKIFGNLKPVFDGRSNLYTRDPLPIGNDRMELEVTLPGEGKDRVFRVAIKW

Sbjct 121 IIETMVHAYSKIFGNLKPVFDGRSNLYTRDPLPIGNDRMELEVTLPGEGKDRVFRVAIKW 180

Query 181 MAQVSLFALEEALEGRTRQIPYDAIMALDVVMRHLPSMTYTPVGRSFFSSPDGYYHPLGG 240

MAQVSLFALEEALEGRTRQIPYDAIMALDVVMRHLPSMTYTPVGRSFFSSPDGYYHPLGG

Sbjct 181 MAQVSLFALEEALEGRTRQIPYDAIMALDVVMRHLPSMTYTPVGRSFFSSPDGYYHPLGG 240

Query 241 GREVWFGFHQSVRPSQWKMMLNIDVSATAFYKAQPVIEFMCEVLDIRDINEQRKPLTDSQ 300

GREVWFGFHQSVRPSQWKMMLNIDVSATAFYKAQPVIEFMCEVLDIRDINEQRKPLTDSQ

Sbjct 241 GREVWFGFHQSVRPSQWKMMLNIDVSATAFYKAQPVIEFMCEVLDIRDINEQRKPLTDSQ 300

Query 301 RVKFTKEIKGLKIEITHCGTMRRKYRVCNVTRRPAQMQSFPLQLENGQTVECTVAKYFLD 360

RVKFTKEIKGLKIEITHCGTMRRKYRVCNVTRRPAQMQSFPLQLENGQTVECTVAKYFLD

Sbjct 301 RVKFTKEIKGLKIEITHCGTMRRKYRVCNVTRRPAQMQSFPLQLENGQTVECTVAKYFLD 360

Query 361 KYKMKLRYPHLPCLQVGQEHKHTYLPLEVCNIVAGQRCIKKLTDMQTSTMIKATARSAPD 420

KYKMKLRYPHLPCLQVGQEHKHTYLPLEVCNIVAGQRCIKKLTDMQTSTMIKATARSAPD

Sbjct 361 KYKMKLRYPHLPCLQVGQEHKHTYLPLEVCNIVAGQRCIKKLTDMQTSTMIKATARSAPD 420

Query 421 REREINNLVRRADFNNDAYVQEFGLAISNNMMEVRGRVLPPPKLQYGGRAPNLPSQLEYQ 480

REREINNLVRRADFNNDAYVQEFGLAISNNMMEVRGRVLPPPKLQYGGRAPNLPSQLEYQ

Sbjct 421 REREINNLVRRADFNNDAYVQEFGLAISNNMMEVRGRVLPPPKLQYGGRAPNLPSQLEYQ 480

Query 481 GMLSAKQQALPNQGVWDMRGKQFFTGVEIRVWAIACFAPQRTVREDALRNFTQQLQKISN 540

GMLSAKQQALPNQGVWDMRGKQFFTGVEIRVWAIACFAPQRTVREDALRNFTQQLQKISN

Sbjct 481 GMLSAKQQALPNQGVWDMRGKQFFTGVEIRVWAIACFAPQRTVREDALRNFTQQLQKISN 540

Query 541 DAGMPIIGQPCFCKYATGPDQVEPMFRYLKSSFQALQLVVVVLPGKTPVYAEVKRVGDTV 600

DAGMPIIGQPCFCKYATGPDQVEPMFRYLKSSFQALQLVVVVLPGKTPVYAEVKRVGDTV

Sbjct 541 DAGMPIIGQPCFCKYATGPDQVEPMFRYLKSSFQALQLVVVVLPGKTPVYAEVKRVGDTV 600

Query 601 LGMATQCVQAKNVNKTSPQTLSNLCLKINVKLGGINSILVPSIRPKVFNEPVIFLGADVT 660

LGMATQCVQAKNVNKTSPQTLSNLCLKINVKLGGINSILVPSIRPKVFNEPVIFLGADVT

Sbjct 601 LGMATQCVQAKNVNKTSPQTLSNLCLKINVKLGGINSILVPSIRPKVFNEPVIFLGADVT 660

Query 661 HPPAGDNKKPSIAAVVGSMDAHPSRYAATVRVQQHRQEIIQELSSMVRELLIMFYKSTGG 720

HPPAGDNKKPSIAAVVGSMDAHPSRYAATVRVQQHRQEIIQELSSMVRELLIMFYKSTGG

Sbjct 661 HPPAGDNKKPSIAAVVGSMDAHPSRYAATVRVQQHRQEIIQELSSMVRELLIMFYKSTGG 720

Query 721 YKPHRIILYRDGVSEGQFLHVLQHELTAIREACIKLEGDYKPGITFIVVQKRHHTRLFCA 780

YKPHRIILYRDGVSEGQFLHVLQHELTAIREACIKLEGDYKPGITFIVVQKRHHTRLFCA

Sbjct 721 YKPHRIILYRDGVSEGQFLHVLQHELTAIREACIKLEGDYKPGITFIVVQKRHHTRLFCA 780

Query 781 DKKEQSGKSGNIPAGTTVDVGITHPTEFDFYLCSHQGIQGTSRPSHYHVLWDDNHFDSDE 840

DKKEQSGKSGNIPAGTTVDVGITHPTEFDFYLCSHQGIQGTSRPSHYHVLWDDNHFDSDE

Sbjct 781 DKKEQSGKSGNIPAGTTVDVGITHPTEFDFYLCSHQGIQGTSRPSHYHVLWDDNHFDSDE 840

Query 841 LQCLTYQLCHTYVRCTRSVSIPAPAYYAHLVAFRARYHLVEKEHDSGEGSHQSGCSEDRT 900

LQCLTYQLCHTYVRCTRSVSIPAPAYYAHLVAFRARYHLVEKEHDSGEGSHQSGCSEDRT

Sbjct 841 LQCLTYQLCHTYVRCTRSVSIPAPAYYAHLVAFRARYHLVEKEHDSGEGSHQSGCSEDRT 900

Query 901 PGAMARAITVHADTKKVMYFA 921

PGAMARAITVHADTKKVMYFA

Sbjct 901 PGAMARAITVHADTKKVMYFA 921

Graphical representation


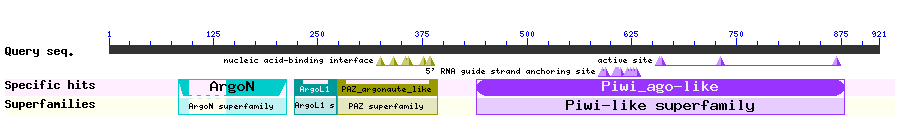


**Argonaute 1- isoform 3**

> TRINITY_DN26672_c0_g1_i3 length= 3987 nt

GTCAAATCCATTTTTTGTAAGTAAATTAAAAGATAACATCCATAAGAAGGAAATTAAAAATAACAATTCTTTTTGTTTAGTTCCGCAGATTAAAATTTTTAAGTACGACTAAGTGAGCAGACTAAATAACCACAGACCTGTTTTAACTTAAAGCTGACTTTAGTACATCGCCTGGAGACATTGACTACGCAACCATTTTCTATATTTATCCTCAAAAAACAAAATATTAAAGCTATCAACTAACAGCTGACTTGGAGACCTTGGCTTAAGAGAGAAATAAAGAGAGAAATAGAAAATAAATATATATAAAAAAAAACAAGCATCATAGAACATGTTACAACTTATCTACTAATTAAAATAGTTATAGTGTATTATCTCTCTTGACAGCCCTATTCCTATTTACATGCCTAGACTTGTGCACCAGCTAAAGAAGACTTACATATATACGGCAGCTTTCCTCAATTAGTTTTCTTCCAACTAGTGAAATTTTACTTAAGAAATATGAATCAGTGTTTTAATTTGACTCTTATGAAAAATATAACTATCACTGCTTTTTTTTGTAGGTACCTTAATCATAAAATAATTATCTTAATATTAAAAATCTTATTAAATAGGACGACCAACACAGCGATAAAATTCCTTCGAAAGAAGGTGAATGATTAAAGACACCACTGAAAAATAGCTGGCTGGTTCCTATAATCCCACATGGCAGGAAGTTCTTTCGCAATTTAGGTTATGCGAAGTACATTACTTTCTTCGTATCTGCATGAACCGTAATAGCTCGTGCCATTGCACCCGGTGTCCGGTCTTCACTACAGCCTGACTGGTGAGAACCCTCACCGCTGTCATGCTCTTTCTCAACAAGATGATACCTCGCCCTAAATGCAACAAGATGAGCGTAATAGGCAGGGGCAGGTATAGATACTGATCGTGTACACCTCACATAGGTATGGCACAACTGGTAAGTTAAGCATTGCAGTTCATCTGAATCGAAATGGTTGTCATCCCATAAAACATGATAATGACTCGGCCGACTAGTACCCTGAATACCTTGGTGGCTGCAAAGATAGAAGTCAAACTCAGTCGGGTGGGTTATCCCCACATCGACTGTTGTACCAGCAGGTATATTTCCAGACTTACCACTTTGCTCTTTTTTGTCAGCACAGAATAACCTTGTATGATGCCTCTTTTGCACTACAATAAATGTTATTCCAGGTTTATAATCTCCTTCGAGTTTAATACAAGCTTCCCTAATAGCTGTAAGTTCATGTTGTAAAACATGTAAGAATTGTCCTTCTGAAACACCATCTCTATACAGAATAATCCTATGAGGCTTGTACCCACCGGTACTTTTGTAAAACATTATCAGAAGCTCCCTGACCATGGAGGATAATTCTTGAATAATTTCTTGACGATGTTGTTGAACCCTAACAGTAGCAGCATATCGACTGGGATGTGCATCCATAGATCCAACGACGGCTGCTATCGAAGGTTTTTTGTTGTCTCCAGCAGGTGGGTGAGTAACATCGGCTCCAAGGAATATTACAGGTTCATTGAAAACCTTTGGCCTAATGCTGGGAACAAGAATGCTGTTAATGCCACCAAGCTTAACATTGATTTTGAGACAAAGATTGGAGAGTGTCTGTGGAGAAGTTTTATTTACATTTTTTGCCTGAACACATTGTGTTGCCATACCTAAGACTGTATCTCCAACCCTCTTCACTTCAGCATATACAGGTGTTTTCCCTGGAAGCACCACTACAACAAGCTGTAATGCCTGGAAGGAAGACTTCAAGTAACGGAACATGGGCTCCACTTGATCTGGTCCAGTAGCATATTTACAGAAGCATGGTTGGCCAATTATTGGCATTCCAGCATCATTACTAATTTTCTGCAATTGTTGGGTAAAATTCCTGAGAGCATCCTCTCTAACTGTCCTTTGAGGTGCGAAACAAGCAATGGCCCACACTCTTATTTCAACACCAGTGAAAAATTGCTTGCCCCGCATATCCCATACACCTTGATTAGGAAGGGCTTGCTGTTTTGCAGACAGCATGCCTTGGTATTCGAGCTGACTAGGCAAGTTAGGAGCTCGCCCACCATACTGAAGCTTCGGTGGCGGCAAAACCCTCCCTCGAACTTCCATCATATTATTGGAAATCGCCAGTCCGAACTCTTGAACATAGGCGTCATTATTGAAATCCGCTCTCCTCACGAGATTGTTGATCTCTCTTTCCCGATCAGGTGCTGACCTAGCAGTAGCTTTGATCATAGTTGAGGTCTGCATGTCAGTAAGCTTTTTAATGCATCTCTGACCAGCCACAATATTGCAAACCTCTAGGGGAAGATATGTATGCTTGTGCTCCTGCCCAACTTGAAGACAAGGTAAATGTGGATACCTTAATTTCATTTTGTATTTGTCTAAGAAATACTTAGCAACTGTGCATTCCACAGTTTGTCCGTTCTCTAATTGTAACGGGAACGACTGCATTTGAGCAGGTCTTCTTGTAACATTGCAGACTCTGTATTTCCTCCTCATTGTTCCACAATGTGTAATTTCAATTTTCAACCCTTTAATTTCTTTAGTAAACTTAACCCTCTGAGAATCTGTCAAGGGTTTTCGTTGTTCATTTATGTCCCTAATGTCTAACACTTCACACATAAACTCGATTACGGGCTGGGCTTTATAAAAAGCTGTGGCTGACACATCGATATTAAGCATCATCTTCCATTGTGAAGGTCTAACAGATTGATGAAAACCAAACCATACCTCACGACCACCGCCAAGTGGATGGTAGTAACCATCAGGACTAGAGAAGAATGATCTTCCTACTGGAGTATAAGTCATTGAAGGCAAATGCCTCATTACTACATCCAGTGCCATTATAGCATCATATGGAATCTGTCGAGTCCTTCCTTCCAATGCTTCTTCTAAGGCAAACAACGAAACTTGCGCCATCCATTTGATGGCCACTCTAAACACACGATCTTTTCCTTCACCTGGCAATGTTACCTCGAGTTCCATCCTATCATTGCCAATTGGAAGTGGATCTCTAGTGTAAAGATTACTTCTTCCATCAAAGACAGGTTTCAAGTTACCAAAGATTTTTGAATAGGCATGGACCATTGTTTCAATGATTTCCCTGTTGACCTTGCGAGGACACTTATCAGGTTGTATACTAATTTCATAATGATGTACGAAACCACGTGGCATAGTTATTTGGAAATGGTTCGCACGAAGCACTATAGGTCGACCTTCTCGGCCGAGATTAGGCCTTCGAGGACAGGTAAACATAGGTAAATCAGGAGGTTGGTGAGGAGGCTGAGGTGGAAGAAGAGCTATCGCTCCAGGGGGGACGCCAACTGGACCTGGTGGGGCCCCACCTCCCGCAGGAGCTCCGGGGGGCCCTACAGGAGGAGCTGGTTGTGCAAAGGATTTTCCAACCATCGAAGCTGTGTCCCTATGGATATGTGCCACTTCTTGGAATGGTACTGCCATTTCTTTATTTGCACTTAGTTACTCAGTTTTACACTTGATTATCAAGTTTTCCCTCATCATCATAGTTAATATAGTATGTTCTCCTAACTTTAGTAATTTTAAGTGTTTTATGAAGCACTGATATCATTATGCTTTGAATTGTATGTGAAGATTTCATCATATTATGATAGGAGTGTTAGAGTGGATTGTTTGACAATACCAAACACCCTGAAAAGTATAAAAGAAAAATGTTTAAAATACTTCAAGAAGTATCTCATATTCATTAAATTTTCTTATATTCTGCATTATGTATACATAACCGATTACCAAACTCTTCATTTCCTTATTTAGGAATAAAATTAACAACTGATATCGAAAAACTAAAATATTAGTTTTTAATTTTGATTCCATTATTGATATGGAAAATTTTAATTTTTATCTGAATAAAATATTTCACCATGGTAAGATAATCATATTATATGGCTTTTGTAAATAGAAATAAAAATAAA

Protein: RF -3: -3499 -> -734 (921 aa)

Comparison with Nezara viridula, PREDICTED: Argonaute-1-PC- Sequence ID: AVK59466.1

E=0.0; bits= 1923

Query 1 MAVPFQEVAHIHRDTASMVGKSFAQPAPPVGPPGAPAGGGAPPGPVGVPPGAIALLPPQP 60

MAVPFQEVAHIHRDTASMVGKSFAQPAPPVGPPGAPAGGGAPPGPVGVPPGAIAL+PPQP

Sbjct 1 MAVPFQEVAHIHRDTASMVGKSFAQPAPPVGPPGAPAGGGAPPGPVGVPPGAIALIPPQP 60

Query 61 PHQPPDLPMFTCPRRPNLGREGRPIVLRANHFQITMPRGFVHHYEISIQPDKCPRKVNRE 120

PHQPPDLPMFTCPRRPNLGREGRPIVLRANHFQITMPRGFVHHYEISIQPDKCPRKVNRE

Sbjct 61 PHQPPDLPMFTCPRRPNLGREGRPIVLRANHFQITMPRGFVHHYEISIQPDKCPRKVNRE 120

Query 121 IIETMVHAYSKIFGNLKPVFDGRSNLYTRDPLPIGNDRMELEVTLPGEGKDRVFRVAIKW 180

IIETMVHAYSKIFGNLKPVFDGRSNLYTRDPLPIGNDRMELEVTLPGEGKDRVFRVAIKW

Sbjct 121 IIETMVHAYSKIFGNLKPVFDGRSNLYTRDPLPIGNDRMELEVTLPGEGKDRVFRVAIKW 180

Query 181 MAQVSLFALEEALEGRTRQIPYDAIMALDVVMRHLPSMTYTPVGRSFFSSPDGYYHPLGG 240

MAQVSLFALEEALEGRTRQIPYDAIMALDVVMRHLPSMTYTPVGRSFFSSPDGYYHPLGG

Sbjct 181 MAQVSLFALEEALEGRTRQIPYDAIMALDVVMRHLPSMTYTPVGRSFFSSPDGYYHPLGG 240

Query 241 GREVWFGFHQSVRPSQWKMMLNIDVSATAFYKAQPVIEFMCEVLDIRDINEQRKPLTDSQ 300

GREVWFGFHQSVRPSQWKMMLNIDVSATAFYKAQPVIEFMCEVLDIRDINEQRKPLTDSQ

Sbjct 241 GREVWFGFHQSVRPSQWKMMLNIDVSATAFYKAQPVIEFMCEVLDIRDINEQRKPLTDSQ 300

Query 301 RVKFTKEIKGLKIEITHCGTMRRKYRVCNVTRRPAQMQSFPLQLENGQTVECTVAKYFLD 360

RVKFTKEIKGLKIEITHCGTMRRKYRVCNVTRRPAQMQSFPLQLENGQTVECTVAKYFLD

Sbjct 301 RVKFTKEIKGLKIEITHCGTMRRKYRVCNVTRRPAQMQSFPLQLENGQTVECTVAKYFLD 360

Query 361 KYKMKLRYPHLPCLQVGQEHKHTYLPLEVCNIVAGQRCIKKLTDMQTSTMIKATARSAPD 420

KYKMKLRYPHLPCLQVGQEHKHTYLPLEVCNIVAGQRCIKKLTDMQTSTMIKATARSAPD

Sbjct 361 KYKMKLRYPHLPCLQVGQEHKHTYLPLEVCNIVAGQRCIKKLTDMQTSTMIKATARSAPD 420

Query 421 REREINNLVRRADFNNDAYVQEFGLAISNNMMEVRGRVLPPPKLQYGGRAPNLPSQLEYQ 480

REREINNLVRRADFNNDAYVQEFGLAISNNMMEVRGRVLPPPKLQYGGRAPNLPSQLEYQ

Sbjct 421 REREINNLVRRADFNNDAYVQEFGLAISNNMMEVRGRVLPPPKLQYGGRAPNLPSQLEYQ 480

Query 481 GMLSAKQQALPNQGVWDMRGKQFFTGVEIRVWAIACFAPQRTVREDALRNFTQQLQKISN 540

GMLSAKQQALPNQGVWDMRGKQFFTGVEIRVWAIACFAPQRTVREDALRNFTQQLQKISN

Sbjct 481 GMLSAKQQALPNQGVWDMRGKQFFTGVEIRVWAIACFAPQRTVREDALRNFTQQLQKISN 540

Query 541 DAGMPIIGQPCFCKYATGPDQVEPMFRYLKSSFQALQLVVVVLPGKTPVYAEVKRVGDTV 600

DAGMPIIGQPCFCKYATGPDQVEPMFRYLKSSFQALQLVVVVLPGKTPVYAEVKRVGDTV

Sbjct 541 DAGMPIIGQPCFCKYATGPDQVEPMFRYLKSSFQALQLVVVVLPGKTPVYAEVKRVGDTV 600

Query 601 LGMATQCVQAKNVNKTSPQTLSNLCLKINVKLGGINSILVPSIRPKVFNEPVIFLGADVT 660

LGMATQCVQAKNVNKTSPQTLSNLCLKINVKLGGINSILVPSIRPKVFNEPVIFLGADVT

Sbjct 601 LGMATQCVQAKNVNKTSPQTLSNLCLKINVKLGGINSILVPSIRPKVFNEPVIFLGADVT 660

Query 661 HPPAGDNKKPSIAAVVGSMDAHPSRYAATVRVQQHRQEIIQELSSMVRELLIMFYKSTGG 720

HPPAGDNKKPSIAAVVGSMDAHPSRYAATVRVQQHRQEIIQELSSMVRELLIMFYKSTGG

Sbjct 661 HPPAGDNKKPSIAAVVGSMDAHPSRYAATVRVQQHRQEIIQELSSMVRELLIMFYKSTGG 720

Query 721 YKPHRIILYRDGVSEGQFLHVLQHELTAIREACIKLEGDYKPGITFIVVQKRHHTRLFCA 780

YKPHRIILYRDGVSEGQFLHVLQHELTAIREACIKLEGDYKPGITFIVVQKRHHTRLFCA

Sbjct 721 YKPHRIILYRDGVSEGQFLHVLQHELTAIREACIKLEGDYKPGITFIVVQKRHHTRLFCA 780

Query 781 DKKEQSGKSGNIPAGTTVDVGITHPTEFDFYLCSHQGIQGTSRPSHYHVLWDDNHFDSDE 840

DKKEQSGKSGNIPAGTTVDVGITHPTEFDFYLCSHQGIQGTSRPSHYHVLWDDNHFDSDE

Sbjct 781 DKKEQSGKSGNIPAGTTVDVGITHPTEFDFYLCSHQGIQGTSRPSHYHVLWDDNHFDSDE 840

Query 841 LQCLTYQLCHTYVRCTRSVSIPAPAYYAHLVAFRARYHLVEKEHDSGEGSHQSGCSEDRT 900

LQCLTYQLCHTYVRCTRSVSIPAPAYYAHLVAFRARYHLVEKEHDSGEGSHQSGCSEDRT

Sbjct 841 LQCLTYQLCHTYVRCTRSVSIPAPAYYAHLVAFRARYHLVEKEHDSGEGSHQSGCSEDRT 900

Query 901 PGAMARAITVHADTKKVMYFA 921

PGAMARAITVHADTKKVMYFA

Sbjct 901 PGAMARAITVHADTKKVMYFA 921

Graphical representation


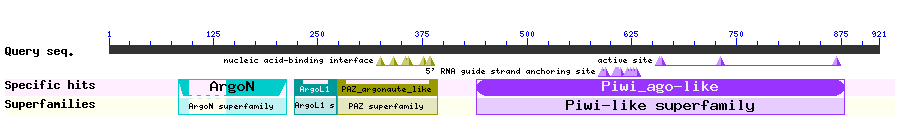


**Argonaute 1- isoform 4**

> TRINITY_DN26672_c0_g1_i4 length= 3896 nt

GTCAAATCCATTTTTTGTAAGTAAATTAAAAGATAACATCCATAAGAAGGAAATTAAAAATAACAATTCTTTTTGTTTAGTTCCGCAGATTAAAATTTTTAAGTACGACTAAGTGAGCAGACTAAATAACCACAGACCTGTTTTAACTTAAAGCTGACTTTAGTACATCGCCTGGAGACATTGACTACGCAACCATTTTCTATATTTATCCTCAAAAAACAAAATATTAAAGCTATCAACTAACAGCTGACTTGGAGACCTTGGCTTAAGAGAGAAATAAAGAGAGAAATAGAAAATAAATATATATAAAAAAAAACAAGCATCATAGAACATGTTACAACTTATCTACTAATTAAAATAGTTATAGTGTATTATCTCTCTTGACAGCCCTATTCCTATTTACATGCCTAGACTTGTGCACCAGCTAAAGAAGACTTACATATATACGGCAGCTTTCCTCAATTAGTTTTCTTCCAACTAGTGAAATTTTACTTAAGAAATATGAATCAGTGTTTTAATTTGACTCTTATGAAAAATATAACTATCACTGCTTTTTTTTGTAGGTACCTTAATCATAAAATAATTATCTTAATATTAAAAATCTTATTAAATAGGACGACCAACACAGCGATAAAATTCCTTCGAAAGAAGGTGAATGATTAAAGACACCACTGAAAAATAGCTGGCTGGTTCCTATAATCCCACATGGCAGGAAGTTCTTTCGCAATTTAGGTTATGCGAAGTACATTACTTTCTTCGTATCTGCATGAACCGTAATAGCTCGTGCCATTGCACCCGGTGTCCGGTCTTCACTACAGCCTGACTGGTGAGAACCCTCACCGCTGTCATGCTCTTTCTCAACAAGATGATACCTCGCCCTAAATGCAACAAGATGAGCGTAATAGGCAGGGGCAGGTATAGATACTGATCGTGTACACCTCACATAGGTATGGCACAACTGGTAAGTTAAGCATTGCAGTTCATCTGAATCGAAATGGTTGTCATCCCATAAAACATGATAATGACTCGGCCGACTAGTACCCTGAATACCTTGGTGGCTGCAAAGATAGAAGTCAAACTCAGTCGGGTGGGTTATCCCCACATCGACTGTTGTACCAGCAGGTATATTTCCAGACTTACCACTTTGCTCTTTTTTGTCAGCACAGAATAACCTTGTATGATGCCTCTTTTGCACTACAATAAATGTTATTCCAGGTTTATAATCTCCTTCGAGTTTAATACAAGCTTCCCTAATAGCTGTAAGTTCATGTTGTAAAACATGTAAGAATTGTCCTTCTGAAACACCATCTCTATACAGAATAATCCTATGAGGCTTGTACCCACCGGTACTTTTGTAAAACATTATCAGAAGCTCCCTGACCATGGAGGATAATTCTTGAATAATTTCTTGACGATGTTGTTGAACCCTAACAGTAGCAGCATATCGACTGGGATGTGCATCCATAGATCCAACGACGGCTGCTATCGAAGGTTTTTTGTTGTCTCCAGCAGGTGGGTGAGTAACATCGGCTCCAAGGAATATTACAGGTTCATTGAAAACCTTTGGCCTAATGCTGGGAACAAGAATGCTGTTAATGCCACCAAGCTTAACATTGATTTTGAGACAAAGATTGGAGAGTGTCTGTGGAGAAGTTTTATTTACATTTTTTGCCTGAACACATTGTGTTGCCATACCTAAGACTGTATCTCCAACCCTCTTCACTTCAGCATATACAGGTGTTTTCCCTGGAAGCACCACTACAACAAGCTGTAATGCCTGGAAGGAAGACTTCAAGTAACGGAACATGGGCTCCACTTGATCTGGTCCAGTAGCATATTTACAGAAGCATGGTTGGCCAATTATTGGCATTCCAGCATCATTACTAATTTTCTGCAATTGTTGGGTAAAATTCCTGAGAGCATCCTCTCTAACTGTCCTTTGAGGTGCGAAACAAGCAATGGCCCACACTCTTATTTCAACACCAGTGAAAAATTGCTTGCCCCGCATATCCCATACACCTTGATTAGGAAGGGCTTGCTGTTTTGCAGACAGCATGCCTTGGTATTCGAGCTGACTAGGCAAGTTAGGAGCTCGCCCACCATACTGAAGCTTCGGTGGCGGCAAAACCCTCCCTCGAACTTCCATCATATTATTGGAAATCGCCAGTCCGAACTCTTGAACATAGGCGTCATTATTGAAATCCGCTCTCCTCACGAGATTGTTGATCTCTCTTTCCCGATCAGGTGCTGACCTAGCAGTAGCTTTGATCATAGTTGAGGTCTGCATGTCAGTAAGCTTTTTAATGCATCTCTGACCAGCCACAATATTGCAAACCTCTAGGGGAAGATATGTATGCTTGTGCTCCTGCCCAACTTGAAGACAAGGTAAATGTGGATACCTTAATTTCATTTTGTATTTGTCTAAGAAATACTTAGCAACTGTGCATTCCACAGTTTGTCCGTTCTCTAATTGTAACGGGAACGACTGCATTTGAGCAGGTCTTCTTGTAACATTGCAGACTCTGTATTTCCTCCTCATTGTTCCACAATGTGTAATTTCAATTTTCAACCCTTTAATTTCTTTAGTAAACTTAACCCTCTGAGAATCTGTCAAGGGTTTTCGTTGTTCATTTATGTCCCTAATGTCTAACACTTCACACATAAACTCGATTACGGGCTGGGCTTTATAAAAAGCTGTGGCTGACACATCGATATTAAGCATCATCTTCCATTGTGAAGGTCTAACAGATTGATGAAAACCAAACCATACCTCACGACCACCGCCAAGTGGATGGTAGTAACCATCAGGACTAGAGAAGAATGATCTTCCTACTGGAGTATAAGTCATTGAAGGCAAATGCCTCATTACTACATCCAGTGCCATTATAGCATCATATGGAATCTGTCGAGTCCTTCCTTCCAATGCTTCTTCTAAGGCAAACAACGAAACTTGCGCCATCCATTTGATGGCCACTCTAAACACACGATCTTTTCCTTCACCTGGCAATGTTACCTCGAGTTCCATCCTATCATTGCCAATTGGAAGTGGATCTCTAGTGTAAAGATTACTTCTTCCATCAAAGACAGGTTTCAAGTTACCAAAGATTTTTGAATAGGCATGGACCATTGTTTCAATGATTTCCCTGTTGACCTTGCGAGGACACTTATCAGGTTGTATACTAATTTCATAATGATGTACGAAACCACGTGGCATAGTTATTTGGAAATGGTTCGCACGAAGCACTATAGGTCGACCTTCTCGGCCGAGATTAGGCCTTCGAGGACAGGTAAACATAGGTAAATCAGGAGGTTGGTGAGGAGGCTGAGGTGGAAGAAGAGCTATCGCTCCAGGGGGGACGCCAACTGGACCTGGTGGGGCCCCACCTCCCGCAGGAGCTCCGGGGGGCCCTACAGGAGGAGCTGGTTGTGCAAAGGATTTTCCAACCATCGAAGCTGTGTCCCTATGGATATGTGCCACTTCTTGGAATGGTACTGCCATTTCTTTATTTGCACTTAGTTACTCAGTTTTACACTTGATTATCAAGTTTTCCCTCATCATCATAGTTAATATAGTATGTTCTCCTAACTTTAGTAATTTTAAGTGTTTTATGAAGCACTGATATCATTATGCTTTGAATTGTATGTGAAGATTTCATCATATTATGATAGGAGTGTTAGAGTGGATTGTTTGACAATACCAAACACCCTTTATTTATCCATCTCAGTATTAAATTTGGTTATATTCAACAATAGAAAATTATGCTACACTATCTTTCAGTTCTTATTATTTATTTAAATATGATTAATGGAAGTTATTTCAGTATGCACACAATCTAATTGTATTAATTTGCTTTTTTAAGTGGCACCAAATCACTTCAGAGATAATATTGTTTGTT

Protein: RF -2: -3499 -> -734 (921 aa)

Comparison with Nezara viridula, PREDICTED: Argonaute-1-PC- Sequence ID: AVK59466.1

E=0.0; bits= 1924

Query 1 MAVPFQEVAHIHRDTASMVGKSFAQPAPPVGPPGAPAGGGAPPGPVGVPPGAIALLPPQP 60

MAVPFQEVAHIHRDTASMVGKSFAQPAPPVGPPGAPAGGGAPPGPVGVPPGAIAL+PPQP

Sbjct 1 MAVPFQEVAHIHRDTASMVGKSFAQPAPPVGPPGAPAGGGAPPGPVGVPPGAIALIPPQP 60

Query 61 PHQPPDLPMFTCPRRPNLGREGRPIVLRANHFQITMPRGFVHHYEISIQPDKCPRKVNRE 120

PHQPPDLPMFTCPRRPNLGREGRPIVLRANHFQITMPRGFVHHYEISIQPDKCPRKVNRE

Sbjct 61 PHQPPDLPMFTCPRRPNLGREGRPIVLRANHFQITMPRGFVHHYEISIQPDKCPRKVNRE 120

Query 121 IIETMVHAYSKIFGNLKPVFDGRSNLYTRDPLPIGNDRMELEVTLPGEGKDRVFRVAIKW 180

IIETMVHAYSKIFGNLKPVFDGRSNLYTRDPLPIGNDRMELEVTLPGEGKDRVFRVAIKW

Sbjct 121 IIETMVHAYSKIFGNLKPVFDGRSNLYTRDPLPIGNDRMELEVTLPGEGKDRVFRVAIKW 180

Query 181 MAQVSLFALEEALEGRTRQIPYDAIMALDVVMRHLPSMTYTPVGRSFFSSPDGYYHPLGG 240

MAQVSLFALEEALEGRTRQIPYDAIMALDVVMRHLPSMTYTPVGRSFFSSPDGYYHPLGG

Sbjct 181 MAQVSLFALEEALEGRTRQIPYDAIMALDVVMRHLPSMTYTPVGRSFFSSPDGYYHPLGG 240

Query 241 GREVWFGFHQSVRPSQWKMMLNIDVSATAFYKAQPVIEFMCEVLDIRDINEQRKPLTDSQ 300

GREVWFGFHQSVRPSQWKMMLNIDVSATAFYKAQPVIEFMCEVLDIRDINEQRKPLTDSQ

Sbjct 241 GREVWFGFHQSVRPSQWKMMLNIDVSATAFYKAQPVIEFMCEVLDIRDINEQRKPLTDSQ 300

Query 301 RVKFTKEIKGLKIEITHCGTMRRKYRVCNVTRRPAQMQSFPLQLENGQTVECTVAKYFLD 360

RVKFTKEIKGLKIEITHCGTMRRKYRVCNVTRRPAQMQSFPLQLENGQTVECTVAKYFLD

Sbjct 301 RVKFTKEIKGLKIEITHCGTMRRKYRVCNVTRRPAQMQSFPLQLENGQTVECTVAKYFLD 360

Query 361 KYKMKLRYPHLPCLQVGQEHKHTYLPLEVCNIVAGQRCIKKLTDMQTSTMIKATARSAPD 420

KYKMKLRYPHLPCLQVGQEHKHTYLPLEVCNIVAGQRCIKKLTDMQTSTMIKATARSAPD

Sbjct 361 KYKMKLRYPHLPCLQVGQEHKHTYLPLEVCNIVAGQRCIKKLTDMQTSTMIKATARSAPD 420

Query 421 REREINNLVRRADFNNDAYVQEFGLAISNNMMEVRGRVLPPPKLQYGGRAPNLPSQLEYQ 480

REREINNLVRRADFNNDAYVQEFGLAISNNMMEVRGRVLPPPKLQYGGRAPNLPSQLEYQ

Sbjct 421 REREINNLVRRADFNNDAYVQEFGLAISNNMMEVRGRVLPPPKLQYGGRAPNLPSQLEYQ 480

Query 481 GMLSAKQQALPNQGVWDMRGKQFFTGVEIRVWAIACFAPQRTVREDALRNFTQQLQKISN 540

GMLSAKQQALPNQGVWDMRGKQFFTGVEIRVWAIACFAPQRTVREDALRNFTQQLQKISN

Sbjct 481 GMLSAKQQALPNQGVWDMRGKQFFTGVEIRVWAIACFAPQRTVREDALRNFTQQLQKISN 540

Query 541 DAGMPIIGQPCFCKYATGPDQVEPMFRYLKSSFQALQLVVVVLPGKTPVYAEVKRVGDTV 600

DAGMPIIGQPCFCKYATGPDQVEPMFRYLKSSFQALQLVVVVLPGKTPVYAEVKRVGDTV

Sbjct 541 DAGMPIIGQPCFCKYATGPDQVEPMFRYLKSSFQALQLVVVVLPGKTPVYAEVKRVGDTV 600

Query 601 LGMATQCVQAKNVNKTSPQTLSNLCLKINVKLGGINSILVPSIRPKVFNEPVIFLGADVT 660

LGMATQCVQAKNVNKTSPQTLSNLCLKINVKLGGINSILVPSIRPKVFNEPVIFLGADVT

Sbjct 601 LGMATQCVQAKNVNKTSPQTLSNLCLKINVKLGGINSILVPSIRPKVFNEPVIFLGADVT 660

Query 661 HPPAGDNKKPSIAAVVGSMDAHPSRYAATVRVQQHRQEIIQELSSMVRELLIMFYKSTGG 720

HPPAGDNKKPSIAAVVGSMDAHPSRYAATVRVQQHRQEIIQELSSMVRELLIMFYKSTGG

Sbjct 661 HPPAGDNKKPSIAAVVGSMDAHPSRYAATVRVQQHRQEIIQELSSMVRELLIMFYKSTGG 720

Query 721 YKPHRIILYRDGVSEGQFLHVLQHELTAIREACIKLEGDYKPGITFIVVQKRHHTRLFCA 780

YKPHRIILYRDGVSEGQFLHVLQHELTAIREACIKLEGDYKPGITFIVVQKRHHTRLFCA

Sbjct 721 YKPHRIILYRDGVSEGQFLHVLQHELTAIREACIKLEGDYKPGITFIVVQKRHHTRLFCA 780

Query 781 DKKEQSGKSGNIPAGTTVDVGITHPTEFDFYLCSHQGIQGTSRPSHYHVLWDDNHFDSDE 840

DKKEQSGKSGNIPAGTTVDVGITHPTEFDFYLCSHQGIQGTSRPSHYHVLWDDNHFDSDE

Sbjct 781 DKKEQSGKSGNIPAGTTVDVGITHPTEFDFYLCSHQGIQGTSRPSHYHVLWDDNHFDSDE 840

Query 841 LQCLTYQLCHTYVRCTRSVSIPAPAYYAHLVAFRARYHLVEKEHDSGEGSHQSGCSEDRT 900

LQCLTYQLCHTYVRCTRSVSIPAPAYYAHLVAFRARYHLVEKEHDSGEGSHQSGCSEDRT

Sbjct 841 LQCLTYQLCHTYVRCTRSVSIPAPAYYAHLVAFRARYHLVEKEHDSGEGSHQSGCSEDRT 900

Query 901 PGAMARAITVHADTKKVMYFA 921

PGAMARAITVHADTKKVMYFA

Sbjct 901 PGAMARAITVHADTKKVMYFA 921

Graphical representation


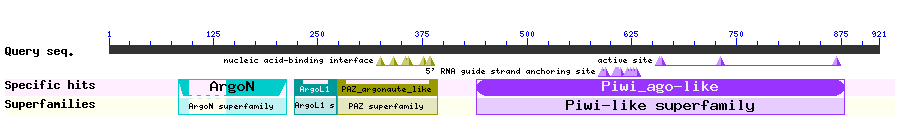


**Argonaute 1 – isoform 5**

>TRINITY_DN26672_c0_g1_i5 length= 3605 nt

GTCAAATCCATTTTTTGTAAGTAAATTAAAAGATAACATCCATAAGAAGGAAATTAAAAATAACAATTCTTTTTGTTTAGTTCCGCAGATTAAAATTTTTAAGTACGACTAAGTGAGCAGACTAAATAACCACAGACCTGTTTTAACTTAAAGCTGACTTTAGTACATCGCCTGGAGACATTGACTACGCAACCATTTTCTATATTTATCCTCAAAAAACAAAATATTAAAGCTATCAACTAACAGCTGACTTGGAGACCTTGGCTTAAGAGAGAAATAAAGAGAGAAATAGAAAATAAATATATATAAAAAAAAACAAGCATCATAGAACATGTTACAACTTATCTACTAATTAAAATAGTTATAGTGTATTATCTCTCTTGACAGCCCTATTCCTATTTACATGCCTAGACTTGTGCACCAGCTAAAGAAGACTTACATATATACGGCAGCTTTCCTCAATTAGTTTTCTTCCAACTAGTGAAATTTTACTTAAGAAATATGAATCAGTGTTTTAATTTGACTCTTATGAAAAATATAACTATCACTGCTTTTTTTTGTAGGTACCTTAATCATAAAATAATTATCTTAATATTAAAAATCTTATTAAATAGGACGACCAACACAGCGATAAAATTCCTTCGAAAGAAGGTGAATGATTAAAGACACCACTGAAAAATAGCTGGCTGGTTCCTATAATCCCACATGGCAGGAAGTTCTTTCGCAATTTAGGTTATGCGAAGTACATTACTTTCTTCGTATCTGCATGAACCGTAATAGCTCGTGCCATTGCACCCGGTGTCCGGTCTTCACTACAGCCTGACTGGTGAGAACCCTCACCGCTGTCATGCTCTTTCTCAACAAGATGATACCTCGCCCTAAATGCAACAAGATGAGCGTAATAGGCAGGGGCAGGTATAGATACTGATCGTGTACACCTCACATAGGTATGGCACAACTGGTAAGTTAAGCATTGCAGTTCATCTGAATCGAAATGGTTGTCATCCCATAAAACATGATAATGACTCGGCCGACTAGTACCCTGAATACCTTGGTGGCTGCAAAGATAGAAGTCAAACTCAGTCGGGTGGGTTATCCCCACATCGACTGTTGTACCAGCAGGTATATTTCCAGACTTACCACTTTGCTCTTTTTTGTCAGCACAGAATAACCTTGTATGATGCCTCTTTTGCACTACAATAAATGTTATTCCAGGTTTATAATCTCCTTCGAGTTTAATACAAGCTTCCCTAATAGCTGTAAGTTCATGTTGTAAAACATGTAAGAATTGTCCTTCTGAAACACCATCTCTATACAGAATAATCCTATGAGGCTTGTACCCACCGGTACTTTTGTAAAACATTATCAGAAGCTCCCTGACCATGGAGGATAATTCTTGAATAATTTCTTGACGATGTTGTTGAACCCTAACAGTAGCAGCATATCGACTGGGATGTGCATCCATAGATCCAACGACGGCTGCTATCGAAGGTTTTTTGTTGTCTCCAGCAGGTGGGTGAGTAACATCGGCTCCAAGGAATATTACAGGTTCATTGAAAACCTTTGGCCTAATGCTGGGAACAAGAATGCTGTTAATGCCACCAAGCTTAACATTGATTTTGAGACAAAGATTGGAGAGTGTCTGTGGAGAAGTTTTATTTACATTTTTTGCCTGAACACATTGTGTTGCCATACCTAAGACTGTATCTCCAACCCTCTTCACTTCAGCATATACAGGTGTTTTCCCTGGAAGCACCACTACAACAAGCTGTAATGCCTGGAAGGAAGACTTCAAGTAACGGAACATGGGCTCCACTTGATCTGGTCCAGTAGCATATTTACAGAAGCATGGTTGGCCAATTATTGGCATTCCAGCATCATTACTAATTTTCTGCAATTGTTGGGTAAAATTCCTGAGAGCATCCTCTCTAACTGTCCTTTGAGGTGCGAAACAAGCAATGGCCCACACTCTTATTTCAACACCAGTGAAAAATTGCTTGCCCCGCATATCCCATACACCTTGATTAGGAAGGGCTTGCTGTTTTGCAGACAGCATGCCTTGGTATTCGAGCTGACTAGGCAAGTTAGGAGCTCGCCCACCATACTGAAGCTTCGGTGGCGGCAAAACCCTCCCTCGAACTTCCATCATATTATTGGAAATCGCCAGTCCGAACTCTTGAACATAGGCGTCATTATTGAAATCCGCTCTCCTCACGAGATTGTTGATCTCTCTTTCCCGATCAGGTGCTGACCTAGCAGTAGCTTTGATCATAGTTGAGGTCTGCATGTCAGTAAGCTTTTTAATGCATCTCTGACCAGCCACAATATTGCAAACCTCTAGGGGAAGATATGTATGCTTGTGCTCCTGCCCAACTTGAAGACAAGGTAAATGTGGATACCTTAATTTCATTTTGTATTTGTCTAAGAAATACTTAGCAACTGTGCATTCCACAGTTTGTCCGTTCTCTAATTGTAACGGGAACGACTGCATTTGAGCAGGTCTTCTTGTAACATTGCAGACTCTGTATTTCCTCCTCATTGTTCCACAATGTGTAATTTCAATTTTCAACCCTTTAATTTCTTTAGTAAACTTAACCCTCTGAGAATCTGTCAAGGGTTTTCGTTGTTCATTTATGTCCCTAATGTCTAACACTTCACACATAAACTCGATTACGGGCTGGGCTTTATAAAAAGCTGTGGCTGACACATCGATATTAAGCATCATCTTCCATTGTGAAGGTCTAACAGATTGATGAAAACCAAACCATACCTCACGACCACCGCCAAGTGGATGGTAGTAACCATCAGGACTAGAGAAGAATGATCTTCCTACTGGAGTATAAGTCATTGAAGGCAAATGCCTCATTACTACATCCAGTGCCATTATAGCATCATATGGAATCTGTCGAGTCCTTCCTTCCAATGCTTCTTCTAAGGCAAACAACGAAACTTGCGCCATCCATTTGATGGCCACTCTAAACACACGATCTTTTCCTTCACCTGGCAATGTTACCTCGAGTTCCATCCTATCATTGCCAATTGGAAGTGGATCTCTAGTGTAAAGATTACTTCTTCCATCAAAGACAGGTTTCAAGTTACCAAAGATTTTTGAATAGGCATGGACCATTGTTTCAATGATTTCCCTGTTGACCTTGCGAGGACACTTATCAGGTTGTATACTAATTTCATAATGATGTACGAAACCACGTGGCATAGTTATTTGGAAATGGTTCGCACGAAGCACTATAGGTCGACCTTCTCGGCCGAGATTAGGCCTTCGAGGACAGGTAAACATAGGTAAATCAGGAGGTTGGTGAGGAGGCTGAGGTGGAAGAAGAGCTATCGCTCCAGGGGGGACGCCAACTGGACCTGGTGGGGCCCCACCTCCCGCAGGAGCTCCGGGGGGCCCTACAGGAGGAGGTTGTCCAACAGGATACATAATAATATTTCACTATATGTAGGAAGAAAACTATAGAGGAATTTTAAGCCTTAAAATATACTGTTCTTAAAGTTTATCAACATGGCTGCTTGCCACTGACGTCACTATCACAGATGTATAGCAATCGCCACTGTCTAGTGAATAAGGTTAATCTACCAGACCTAAT

Protein: RF -2: ORF – 3439 -> -734 (901 aa)

Comparison with Halyomorpha halys: REDICTED: protein argonaute-2 isoform X3 - Sequence ID: XP_014287705.1

E= 0.0; bits= 1877

Query 1 MYPVGQPPPVGPPGAPAGGGAPPGPVGVPPGAIALLPPQPPHQPPDLPMFTCPRRPNLGR 60

MYPVGQPPPVGPPGAPAGGGAPPGPVGVPPGAIALLPPQPPHQPPDLPMFTCPRRPNLGR

Sbjct 1 MYPVGQPPPVGPPGAPAGGGAPPGPVGVPPGAIALLPPQPPHQPPDLPMFTCPRRPNLGR 60

Query 61 EGRPIVLRANHFQITMPRGFVHHYEISIQPDKCPRKVNREIIETMVHAYSKIFGNLKPVF 120

EGRPIVLRANHFQITMPRGFVHHYEISIQPDKCPRKVNREIIETMVHAYSKIFGNLKPVF

Sbjct 61 EGRPIVLRANHFQITMPRGFVHHYEISIQPDKCPRKVNREIIETMVHAYSKIFGNLKPVF 120

Query 121 DGRSNLYTRDPLPIGNDRMELEVTLPGEGKDRVFRVAIKWMAQVSLFALEEALEGRTRQI 180

DGRSNLYTRDPLPIGNDRMELEVTLPGEGKDRVFRVAIKWMAQVSLFALEEALEGRTRQI

Sbjct 121 DGRSNLYTRDPLPIGNDRMELEVTLPGEGKDRVFRVAIKWMAQVSLFALEEALEGRTRQI 180

Query 181 PYDAIMALDVVMRHLPSMTYTPVGRSFFSSPDGYYHPLGGGREVWFGFHQSVRPSQWKMM 240

PYDAIMALDVVMRHLPSMTYTPVGRSFFSSPDGYYHPLGGGREVWFGFHQSVRPSQWKMM

Sbjct 181 PYDAIMALDVVMRHLPSMTYTPVGRSFFSSPDGYYHPLGGGREVWFGFHQSVRPSQWKMM 240

Query 241 LNIDVSATAFYKAQPVIEFMCEVLDIRDINEQRKPLTDSQRVKFTKEIKGLKIEITHCGT 300

LNIDVSATAFYKAQPVIEFMCEVLDIRDINEQRKPLTDSQRVKFTKEIKGLKIEITHCGT

Sbjct 241 LNIDVSATAFYKAQPVIEFMCEVLDIRDINEQRKPLTDSQRVKFTKEIKGLKIEITHCGT 300

Query 301 MRRKYRVCNVTRRPAQMQSFPLQLENGQTVECTVAKYFLDKYKMKLRYPHLPCLQVGQEH 360

MRRKYRVCNVTRRPAQMQSFPLQLENGQTVECTVAKYFLDKYKMKLRYPHLPCLQVGQEH

Sbjct 301 MRRKYRVCNVTRRPAQMQSFPLQLENGQTVECTVAKYFLDKYKMKLRYPHLPCLQVGQEH 360

Query 361 KHTYLPLEVCNIVAGQRCIKKLTDMQTSTMIKATARSAPDREREINNLVRRADFNNDAYV 420

KHTYLPLEVCNIVAGQRCIKKLTDMQTSTMIKATARSAPDREREINNLVRRADFNNDAYV

Sbjct 361 KHTYLPLEVCNIVAGQRCIKKLTDMQTSTMIKATARSAPDREREINNLVRRADFNNDAYV 420

Query 421 QEFGLAISNNMMEVRGRVLPPPKLQYGGRAPNLPSQL-EYQGMLSAKQQALPNQGVWDMR 479

QEFGLAISNNMMEVRGRVLPPPKLQYGGRAPNLPSQL EYQGMLSAKQQALPNQGVWDMR

Sbjct 421 QEFGLAISNNMMEVRGRVLPPPKLQYGGRAPNLPSQLQEYQGMLSAKQQALPNQGVWDMR 480

Query 480 GKQFFTGVEIRVWAIACFAPQRTVREDALRNFTQQLQKISNDAGMPIIGQPCFCKYATGP 539

GKQFFTGVEIRVWAIACFAPQRTVREDALRNFTQQLQKISNDAGMPIIGQPCFCKYATGP

Sbjct 481 GKQFFTGVEIRVWAIACFAPQRTVREDALRNFTQQLQKISNDAGMPIIGQPCFCKYATGP 540

Query 540 DQVEPMFRYLKSSFQALQLVVVVLPGKTPVYAEVKRVGDTVLGMATQCVQAKNVNKTSPQ 599

DQVEPMFRYLKSSFQALQLVVVVLPGKTPVYAEVKRVGDTVLGMATQCVQAKNVNKTSPQ

Sbjct 541 DQVEPMFRYLKSSFQALQLVVVVLPGKTPVYAEVKRVGDTVLGMATQCVQAKNVNKTSPQ 600

Query 600 TLSNLCLKINVKLGGINSILVPSIRPKVFNEPVIFLGADVTHPPAGDNKKPSIAAVVGSM 659

TLSNLCLKINVKLGGINSILVPSIRPKVFNEPVIFLGADVTHPPAGDNKKPSIAAVVGSM

Sbjct 601 TLSNLCLKINVKLGGINSILVPSIRPKVFNEPVIFLGADVTHPPAGDNKKPSIAAVVGSM 660

Query 660 DAHPSRYAATVRVQQHRQEIIQELSSMVRELLIMFYKSTGGYKPHRIILYRDGVSEGQFL 719

DAHPSRYAATVRVQQHRQEIIQELSSMVRELLIMFYKSTGGYKPHRIILYRDGVSEGQFL

Sbjct 661 DAHPSRYAATVRVQQHRQEIIQELSSMVRELLIMFYKSTGGYKPHRIILYRDGVSEGQFL 720

Query 720 HVLQHELTAIREACIKLEGDYKPGITFIVVQKRHHTRLFCADKKEQSGKSGNIPAGTTVD 779

HVLQHELTAIREACIKLEGDYKPGITFIVVQKRHHTRLFCADKKEQSGKSGNIPAGTTVD

Sbjct 721 HVLQHELTAIREACIKLEGDYKPGITFIVVQKRHHTRLFCADKKEQSGKSGNIPAGTTVD 780

Query 780 VGITHPTEFDFYLCSHQGIQGTSRPSHYHVLWDDNHFDSDELQCLTYQLCHTYVRCTRSV 839

VGITHPTEFDFYLCSHQGIQGTSRPSHYHVLWDDNHFDSDELQCLTYQLCHTYVRCTRSV

Sbjct 781 VGITHPTEFDFYLCSHQGIQGTSRPSHYHVLWDDNHFDSDELQCLTYQLCHTYVRCTRSV 840

Query 840 SIPAPAYYAHLVAFRARYHLVEKEHDSGEGSHQSGCSEDRTPGAMARAITVHADTKKVMY 899

SIPAPAYYAHLVAFRARYHLVEKEHDSGEGSHQSGCSEDRTPGAMARAITVHADTKKVMY

Sbjct 841 SIPAPAYYAHLVAFRARYHLVEKEHDSGEGSHQSGCSEDRTPGAMARAITVHADTKKVMY 900

Query 900 FA 901

FA

Sbjct 901 FA 902

Graphical representation


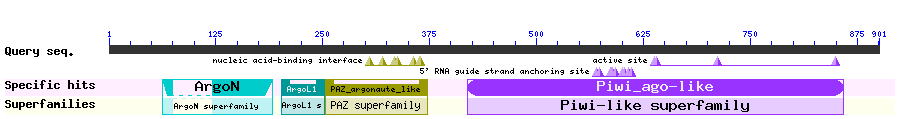


**Loquacious**

>TRINITY_DN26287_c0_g1_i1 length= 1864 nt

GTCGAATTTTTAAAGCTATGATATCACCAAAAAATAAAAATAAAATAAAAATCGCAGAAAAAAAGTTTAAAAGAAACTTATCCCATGTAAAAAATAATGGTGAGATGAATTAAAATAAAAATCCTTGTTACATTTACTTCAATTACCTTTGGCACATTGTAGCTGCTCCAAACAAAAAGGGTAACAGTACAGTTAATTTCATATTTAATTTAAGAAACTTTCAAAAACAAAATGAAAGGAAGAACAGAAAATACTAAATGTAAAAACATAAATACATAACTTTTATATTTTACTTATCTATAACAGTAAGTAACAAAAAAGGTTAAAAATTAAAAATAATTTCAGTTTTCAAGCAACTTCAACCAAGAAAACATTGGTTATCCTGCTTAACCATAAATATGTTTTACCAAGAAGCTAAGTGGTGATCAGAAAACATAAATGGTTATGTTTAAGAAATGGTTAAGAAAGGCATTGTAAAATAGGTTTACAACTAACAATGGGCCATAAATCATTCTGATGGATGAAGAAAACATATTTCATCTAGCTTTAATTGTAAAGATGATTTCCATTTTTGAGCTCATATCAATAAATTAATGGTACATAATTAAAATCAATTAAAAGTTATTAAAAACAAATTATCAGAGCAACAATATTTCTGATTCCTGTTGAGGAGGCTACCTTGCCAGAGGAAATGAGAACAAAGCAATAGAAACAGAGAAAGAAATGAGGTACACAGTTGCAGTTCTGAGCAGGTCTTCAGTCGCCCAATCCTATTTCTTGGTCATCAGTTTGAGATACTGTAGTGCATTGTAGGCCGCGCACGTTTGCGCCTCAGAGGAGCTCTGCCCGGTGCCATAACAAACTGCTACAGGCAGCGTCGAAAGCTGAACCAAGCTCTGACATTTTCCTGAGACAGATTTTTCTTCAACATCAACAAATGTTACTTCAAACTGTTGCTCAGCGGCTATTTCCTGTAGGAACTGGACTGCATTAAAATCTGAATCCTTTAAAGAAATAATCTGGAGCTCATCAAGCTTCGGCCCGACCGATGATTTGAGGTTCTTGTGGAATTGTGACACTTTGATGTTGTAAGGTGCCGTGGCAAGTGTTGGGATTTTGACCATCTTCAATGAATCAAACTCACTTTGAGGAACTCTCTGATTTATCTCATCTTCATCATCTAAGCCATAATGAATAGTGTTGCTTTCACAGGGTAGGTCTTTCAGTCTCATCCACATTTTATGTGCTGCTAGCCTCTTGGCAATTTTTTTCGATTTGCCAGTTCCTATTTCTTTATGCCGGAATACAAGGCAGGAAATGGTGAACTGTCTTTCATGGGGAAGGCCTTCTTCATTCTCTGTTTCGTAAGCAGGCGGAGGCCATCGCCTTGTCATGCATAACTCTTGCAGAAGACCAATAGGGTTGCCCATCATTCGCTCCTCAACTGCCCCAATGTTGGAGCCTAGGCTATCCGGATTTGAGGTCGGGCTAGAGATATCCATAGGTTCTGTATTTCCCGTGATCTTATCTAGAATCGCCTTCGCAGCAGCATGCTTTGCTTCTTTCTTAGATCTACCGGTGCCCATAGCAGCAAATCTACTAGATCAATAAACAGAATCTTGATGAATAAAAATGCATTAAATGTGAGAAAGTCTATCCAAACTTTTGGCCTGCAGTGTATCCACAAAAATTATTTTTCACTGGTTGACAGGGACTGCTTAGAGGCTATCCGGATTTGAGGTCGGGCTAGAGATATCCATAGGTTCTGTATTTCCCGTGATCTTATCTAGAATCGCCTTCGCAGCAGCATGCTTTGCTTCTTTCTTAGATCTACCGGTGCCCATAGCAGCAAATCTACTAGATC

Protein: RF -3: ORF: -1586 -> -771 (271 aa)

Comparison with Halyomorpha halys, REDICTED: RISC-loading complex subunit tarbp2-like isoform X1 - Sequence ID: XP_014274312.1

E=0.0; bits= 521

Query 1 MGTGRSKKEAKHAAAKAILDKITGNTEPMDISSPTS-NPDSLGSNIGAVEERMMGNPIGL 59

MGTGRSKKEAKHAAAKAILDKITGNTE +DISSPTS N +SLG NIG VEERMMGNPIGL

Sbjct 114 MGTGRSKKEAKHAAAKAILDKITGNTESLDISSPTSSNTESLGPNIGTVEERMMGNPIGL 173

Query 60 LQELCMTRRWPPPAYETENEEGLPHERQFTISCLVFRHKEIGTGKSKKIAKRLAAHKMWM 119

LQELCMTRRWPPPAYETENEEGLPHERQFTISCLVFRHKEIGTGKSKKIAKRLAAHKMWM

Sbjct 174 LQELCMTRRWPPPAYETENEEGLPHERQFTISCLVFRHKEIGTGKSKKIAKRLAAHKMWM 233

Query 120 RLKDLPCESNTIHYGLDDEDEINQRVPQSEFDSLKMVKIPTLATAPYNIKVSQFHKNLKS 179

RLKDLPCES TIHYGLDDEDEINQRVPQSEFDSLKMVKIPTLATAPYNIKVSQFHKNLKS

Sbjct 234 RLKDLPCESTTIHYGLDDEDEINQRVPQSEFDSLKMVKIPTLATAPYNIKVSQFHKNLKS 293

Query 180 SVGPKLDELQIISLKDSDFNAVQFLQEIAAEQQFEVTFVDVEEKSVSGKCQSLVQLSTLP 239

SVGPKLDELQIISLKDSDFNAVQFLQEIAAEQQFEVTFVDVEEKSV+GKCQSLVQLSTLP

Sbjct 294 SVGPKLDELQIISLKDSDFNAVQFLQEIAAEQQFEVTFVDVEEKSVTGKCQSLVQLSTLP 353

Query 240 VAVCYGTGQSSSEAQTCAAYNALQYLKLMTKK 271

VAVCYGTG SSSEAQTCAAYNALQYLKLMTKK

Sbjct 354 VAVCYGTGMSSSEAQTCAAYNALQYLKLMTKK 385

Graphical representation


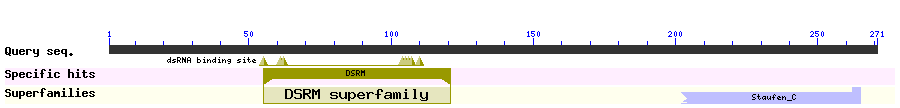


**Drosha (Ribonuclease 3)**

>TRINITY_DN27969_c7_g1_i2 length= 4363 nt

GCCCTTTTATCTGATTTATCTTATAATAAAATATTTTACGCAAAGTAAAAACAATAAATAGCTGAATATATATTAAAAACAAGAATTATAGCTAATCTTAGGTCTAATGATTTCAACTATAATAATATAATTTTATGTTTCAAAAACATGCTTATTCTGTTCATCTATTGAAAATGAGCTGTTATCATCTTCTGACGATGAATCTTCTTCTTTTTCCCTTACTTCTCCGTTTGATACACTGTTTTTATCCACTCCAGCTAATTGATCTTTCATAGATTTCAGTTCATTTGTGTAAAATAAATATTTGTTTGATAGTTCCTCATTAGCTTCATATCTTTGCACAGACCTATTTGAACTATTAGTACTTTGGTCTACACTGCTTCCATCTTCAGAAGAGCTGGTGGAAGATGTGCTGTAATAACGAACTTTTTTCTTTTTAAATTGCTGATCTGAACTAGATTCATCAGAACTAGACATTCTGTCGAGATTATCTTCATGAGATTTTTTATATTTGTATTTTTTAATTTTTCTGGAACTTTCTGAATCAGAATCTGATTTATCATCACTAGAAGAATATTTTCTGGATGTGGGTGATGATTCTTTGGAAGAAGAGGCTTTTGTGTTCTCCTTTTTAATTTTCTTATGCAAACTGTCATAATCATATTTTCTGTGCCTTTTTTCCTCATAATACTCTTTGTTTTGCTTCTTTTTTGATGCTGCAGAGTTCAGTGGTTCTTGCTTCATACTTTTAGCGATTACACGTTTTTGATGATCTAACTGAGGAAACAAACTCTGTGAGTGTTCCAAAGCTACTTTTGCTGCATTCATTTCAGCTTGTTGAATACTATGACCAGAAGCTTCAGCCAAACGTTTTCCTTTAAAATAAACAGCAACAGTGTACAAACGAGTGTTTGTTGGTCCCATGCATTGAATGACTTTATAAACTGGTATCTCTGGTTCACCTCCTTCCATTGTTCTCAAAGTAAGGCAACATTGTTGCAACTTTGATTTGGGGTCATTCCAATCTTGATGCATAATGAAATCATGAAGTCTTGGAAAGAAACAAACATTGCAGAAGGTCTGACAGTATAGTAAGCCTTTATCAACATAAAGAGCTCCAAGAAAAGCTTCTAAAAGATCAGCCCTGTCCTTTGTTTTTAGTTCAGCTTTTGGATTAGAATATACAGCATAAGCAGCCATACCAAGATCGTCACAAACCACTGCCTGGGTTCTATTGTTTACCAAAGAACTTCTCAGTAATGATAGATGACCTTCATGATGTTCAGGAAAATATTTATACAAATATTCTGAAGCAATTAACTGAAGAACAGTATCTCCAAGAAATTCTAACCTCTGATTGGACCCAAGAGTTAAGTTATTATAACCCATACTTCTGTCGGTAAAAGCACGGGCTAAAAGACGAATATGATTAAACTGAACACCAATAGAATCTTCAAATTTTGTAAGGTTTTGTAACATCTGGTAGGATTCAATCCATTTCCTATCACCAGCAGGTTCTTCTTCCTGCAAAGGATGAGGAGGATAATTCCTCCAAACATTTTGTAAAATTTCATTGTCTTCAAATAGAACTTCACCAAAAACTTTATCAGCTGCTTCTATACCACCATCAAGGAAAAGTGCACCCATTAAAGCCTCAAAACAGTTAGCCATTGCATGCCTCAACTCAAGATCATGACATAAATCTGAGCCATGAGCATATAGCATATATCCATCTAGACCTAATGTTTTTGCTAAAACTGCTAAGTGTTGATTTTGAACAATAGCCGCTCTATAAGTGGCAAGACCTCCTTCCTCAAGCCTAGGGAAAAGGTGAAACAAATGAATTGATGAAACGAATTCGACAACAGCATCCCCAAGGAATTCTAAACGTTCGTTGTGAGTAATATTAGATTCAGTTTCTCGCTTCTTACCAAATCTGGACATAATATTGATAAGTGTATTTATACCTCTCTTTCTTGTGTTCATATAATGAATTCGCCTATCTCCATATTCTGGCTGCCGAATTCCACAATTGGTAAGGGAATTTCGAGCATGATCAGGATTGGTTCCAAAATTCTCTCTGTAAGATGGATGAGTTAAAGCAAGCTGTAATAAGCTCCTATTTTTAAATTTATAATTTATTTTGTTTTCTAATACATCTAAAGAGCGATGGAATCTTAAATGAGATACTAAAACAGGAAGAAGCATGGCATGCTGGACTATATCACACATAATCCCTGTTCTGTAAAATCCTTCACTGGAAACAGCAATAGTAACATCTCGTTTCATTTTGCTTTGACTTCTCATCTCTTGAAGTTTATTTTCTTTTGCCTCCAATTTCCTTTTATCTTCATATGAGGATTTGGGCATATTGGCAAGAAGATGACGGAATTTCACATATTCTCGCCATGCTTTCTGATATTCTGGATTTCCAGCATAACTCAATTGCGGAGGACGAATTCCAAAATGAACTATTTCTGGGTATTTTGGATTACCTTCACTTGCATTACTTTGATCCTTATCAAGTTGGTCCACTCTGATGGAGCAAGGTTTTTTTCCAGGATAGGTAACCACCATTCCTTTAACCTCGTCAACAAGTTTTTGCCATTTGTAATTGGGTAAACTTATAATTTCTTTAAGCTCATCTTGTGGTATTAAAAGCTTTGAACTATTCAATAAGTAGCTCAATATTACATTCATTGATAGTAATTCTTTTCCATTTTCGGGAAGTTCACGTACGAACCGAGGCATAAAGTGGAACTGAGCACAACCATTAGAATCACCAGCTGCTTTAAAGTCTAGATCTACTAATTCCAAAATTTCTTTAAACAGAAACTCATGAAATAAGTCCAGCTCCTTTATAGTGAAATTTTGTGGTAGGTTTTCTTCAACATAAAGTATAGTGTATTCGATATTAAATCTAATAACTTTACATACAGGCAAATTTTCCAATGGTTTATGAGAAAACATGGAATAACCTTCAAATATGAATTCATGGTGATCATATTCAATTACAGTTGGTGTTTTAATTAAGAAATTGGTAGGAGGAGAAATAGTGATTCTGTAATGATACAATTTATCAGCATTATTTGTATTGACATCACATTTTTGTAAACTTAATTCACCAGCATAAATATTATGTCTAATACCAGATCGCTTGGCCTTAGCACTACATCGACACAAAGGGCCATCATTCATTTCTCCAGGATCATTGTACCATAATTCTGGGTGTAACCTATCAGGATGTGCTTTCTTTCTCTCTAATTCTTCAAGGAGTCCTTCATCATCCTCAGATTCATTTTCTGAGGAGGAATCTGAACTTGAATCATGATTTAAATTACAGCATTTATGCTTGTGTAACTTTTTCTTCCTTGGAGGGCATTGGTATGGAGGTTTCTCAGCCCTTGCTCTTTGACCTCTCTTTACCAATTCTTCTTCAAATTGACTACAGAGACTTAAAAGTTTTTCAGTAGCTTCCAACACAAGAGAATTGTCTTGTTTTTTAACATAATATAAATCAGCAGGTGCTGAACGTGTCCAAAACAATTTTTCTTTTTTTATAATATCTTCTGGATTCATATGACTAATTTCTTCTAATTTGGTTTTCATTTCTTTTGAAGTTGAACAGTTGTTTAATCTCCATTTATCAATAAATGATTGTTTTCTGGATTTAGAAGAATTACTTCTATCTCTCTCTGATGAATCAGAACTATCTCTTCTATAACTTCTAGATTTTCTGCTAATTACACTGCTTGAACTACTGTGTGCTCTTCTTCGTGGCTCTGGGCTGTAGGATCTATGTTTTGATCGATCTCTACTTGATCCTTTGTCAAAGCTACTCCTTGATCCATTTAAACATTTATAAATAAATTCATCTTGTAATTTAGCGCATTCATCCTTGCTTATGATTGTATCTGAAGCATTGGTTACACTATGACCATTTGAAATGTTATTTTGAGATTGACTACTCAGTACATCACTTCCCTGCTGAAACACTGGAGGTGGAGGATATGATGTATCATATGGTACAAATGCAGGAGTACTTGGATTGTAACAATTGTAAAATGCTGGAGGTGGCTGGGTATAATTCCCATCAGCATTATAAGAATAAGGCACTGTTGGTGGGTAATAGTAATACTGAGACATGATATACATATATCACATTAAACACTTATGAGATTTTGAAAACAGGATATCCTCTATGTAATGTTTAAATCACTATATACATGCTGTGGAATTCACTTCCACAAATCATAAAAATTGCGTAAGATTAATATATTACTACAAATTAAAACACTGAAGCGTAGTTTTATGAAAAACCATGTCGAACTGAAATTAAAAAATGTAAGAAACAGTATAAATT

Protein: RF -2: -4155 -> -133 (1340 aa)

Comparison with *Halyomorpha halys*, PREDICTED: ribonuclease 3 - Sequence ID: XP_014278529.1

E= 0.0; bits= 2366

Query 4 MSQYYYYPPTVPYSYNADGNYTQPPPAFYNCYNPSTPAFVPYDTSYPPPPVFQQGSDVLS 63

MSQYYYYP TVP+SYN + NY QPPP ++N YNPSTP +V YDTS PPPPVFQQGS++++

Sbjct 1 MSQYYYYPQTVPFSYNTESNYIQPPPPYFNSYNPSTPGYVSYDTSCPPPPVFQQGSELVT 60

Query 64 SQSQNNISNGHSVTNASDTIISKDECAKLQDEFIYKCLNGSRSSFDKGSSRDRSKHRSYS 123

+Q QNNI +G++VT +SDT +SKDECAKLQDEFIYKCLNGSRSSFDK RDRSKH+SYS

Sbjct 61 NQFQNNIVSGNNVTTSSDTNLSKDECAKLQDEFIYKCLNGSRSSFDKRPHRDRSKHKSYS 120

Query 124 PEPRRRAHSSSSSVISRKSRSYRRDSSDSSERDRSNSSKSRKQSFIDKWRLNNCSTSKEM 183

PEPRRR+HSSS S I RK R++RRDSSDSSERDR+ KSRK+SFIDKWRLNNCST+KEM

Sbjct 121 PEPRRRSHSSSHSAIIRKHRNHRRDSSDSSERDRNFHLKSRKKSFIDKWRLNNCSTTKEM 180

Query 184 KTKLEEISHMNPEDIIKKEKLFWTRSAPADLYYVKKQDNSLVLEATEKLLSLCSQFEEEL 243

K KLEEISHMNPEDIIKKEKLFWTRSAPADLYY+KKQ +S VLEAT+KLLSLC QFEEEL

Sbjct 181 KNKLEEISHMNPEDIIKKEKLFWTRSAPADLYYIKKQGSSSVLEATDKLLSLCCQFEEEL 240

Query 244 VKRGQRARAEKPPYQCPPRKKKLHKHKCCNLNHDSSSDSSSENESEDDEGLLEELERKKA 303

VKRGQRARAEKPPYQCPPRKKKLHKHKCCNLNHDSSSD+SSENESE+DEGLLEELERKKA

Sbjct 241 VKRGQRARAEKPPYQCPPRKKKLHKHKCCNLNHDSSSDTSSENESEEDEGLLEELERKKA 300

Query 304 HPDRLHPELWYNDPGEMNDGPLCRCSAKAKRSGIRHNIYAGELSLQKCDVNTNNADKLYH 363

HPDRLH ELWYNDPGEMNDGPLCRCSAKAKRSGIRHNIYAGELSL+KCD+NTNNADKLYH

Sbjct 301 HPDRLHAELWYNDPGEMNDGPLCRCSAKAKRSGIRHNIYAGELSLRKCDINTNNADKLYH 360

Query 364 YRITISPPTNFLIKTPTVIEYDHHEFIFEGYSMFSHKPLENLPVCKVIRFNIEYTILYVE 423

YRITISPPTNFLIKTPTVIEYDHHEFIFEGYSMFSHKPLENLPVCKVIRFNIEYTILYVE

Sbjct 361 YRITISPPTNFLIKTPTVIEYDHHEFIFEGYSMFSHKPLENLPVCKVIRFNIEYTILYVE 420

Query 424 ENLPQNFTIKELDLFHEFLFKEILELVDLDFKAAGDSNGCAQFHFMPRFVRELPENGKEL 483

ENLPQNFTI+ELDLFHEFLFKEILELVDLDFKAAGDSNGCAQFHFMPRFVRELPENGKEL

Sbjct 421 ENLPQNFTIRELDLFHEFLFKEILELVDLDFKAAGDSNGCAQFHFMPRFVRELPENGKEL 480

Query 484 LSMNVILSYLLNSSKLLIPQDELKEIISLPNYKWQKLVDEVKGMVVTYPGKKPCSIRVDQ 543

LSMNVILSYLLNSSKLLIP+DELKEIISLPNYKWQKLVDEVKGMVVTYPGKKPCSIRVDQ

Sbjct 481 LSMNVILSYLLNSSKLLIPKDELKEIISLPNYKWQKLVDEVKGMVVTYPGKKPCSIRVDQ 540

Query 544 LDKDQSNASEGNPKYPEIVHFGIRPPQLSYAGNPEYQKAWREYVKFRHLLANMPKSSYED 603

LDKDQSNAS+GNPKYPEIVHFGIRPPQLSYAGNPEYQKAWREYVKFRHLLANMPKSSYED

Sbjct 541 LDKDQSNASDGNPKYPEIVHFGIRPPQLSYAGNPEYQKAWREYVKFRHLLANMPKSSYED 600

Query 604 KRKLEAKENKLQEMRSQSKMKRDVTIAVSSEGFYRTGIMCDIVQHAMLLPVLVSHLRFHR 663

KRKLEAKENKLQEMRSQSKMKRDVTIAVSSEGFYRTGIMCDIVQHAMLLPVLVSHLRFHR

Sbjct 601 KRKLEAKENKLQEMRSQSKMKRDVTIAVSSEGFYRTGIMCDIVQHAMLLPVLVSHLRFHR 660

Query 664 SLDVLENKINYKFKNRSLLQLALTHPSYRENFGTNPDHARNSLTNCGIRQPEYGDRRIHY 723

SLDVLENKINYKFKNRSLLQLALTHPSYRENFGTNPDHARNSLTNCGIRQPEYGDRRIHY

Sbjct 661 SLDVLENKINYKFKNRSLLQLALTHPSYRENFGTNPDHARNSLTNCGIRQPEYGDRRIHY 720

Query 724 MNTRKRGINTLINIMSRFGKKRETESNITHNERLEFLGDAVVEFVSSIHLFHLFPRLEEG 783

MNTRKRGINTLINIMSRFGKKRETESNITHNERLEFLGDAVVEFVSSIHLFHLFPRLEEG

Sbjct 721 MNTRKRGINTLINIMSRFGKKRETESNITHNERLEFLGDAVVEFVSSIHLFHLFPRLEEG 780

Query 784 GLATYRAAIVQNQHLAVLAKTLGLDGYMLYAHGSDLCHDLELRHAMANCFEALMGALFLD 843

GLATYRAAIVQNQHLAVLAKTLGLDGYMLYAHGSDLCHDLELRHAMANCFEALMGALFLD

Sbjct 781 GLATYRAAIVQNQHLAVLAKTLGLDGYMLYAHGSDLCHDLELRHAMANCFEALMGALFLD 840

Query 844 GGIEAADKVFGEVLFEDNEILQNVWRNYPPHPLQEEEPAGDRKWIESYQMLQNLTKFEDS 903

GGIEAADKVFGEVLFEDNEILQNVWRNYPPHPLQEEEPAGDRKWIESYQMLQNLTKFEDS

Sbjct 841 GGIEAADKVFGEVLFEDNEILQNVWRNYPPHPLQEEEPAGDRKWIESYQMLQNLTKFEDS 900

Query 904 IGVQFNHIRLLARAFTDRSMGYNNLTLGSNQRLEFLGDTVLQLIASEYLYKYFPEHHEGH 963

IGV+FNHIRLLARAFTDRSMGYNNLTLGSNQRLEFLGDTVLQLIASEYLYKYFPEHHEGH

Sbjct 901 IGVKFNHIRLLARAFTDRSMGYNNLTLGSNQRLEFLGDTVLQLIASEYLYKYFPEHHEGH 960

Query 964 LSLLRSSLVNNRTQAVVCDDLGMAAYAVYSNPKAELKTKDRADLLEAFLGALYVDKGLLY 1023

LSLLRSSLVNNRTQAVVCDDLGMAAYAVYSNPKAELKTKDRADLLEAFLGALYVDKGLLY

Sbjct 961 LSLLRSSLVNNRTQAVVCDDLGMAAYAVYSNPKAELKTKDRADLLEAFLGALYVDKGLLY 1020

Query 1024 CQTFCNVCFFPRLHDFIMHQDWNDPKSKLQQCCLTLRTMEGGEPEIPVYKVIQCMGPTNT 1083

CQTFCNVCFFPRLHDFIMHQDWNDPKSKLQQCCLTLRTMEGGEPEIPVYKVIQCMGPTNT

Sbjct 1021 CQTFCNVCFFPRLHDFIMHQDWNDPKSKLQQCCLTLRTMEGGEPEIPVYKVIQCMGPTNT 1080

Query 1084 RLYTVAVYFKGKRLAEASGHSIQQAEMNAAKVALEHSQSLFPQLDHQKRVIAKSMKQEPL 1143

RLYTVAVYFKGKRLAEASGHSIQQAEMNAAKVALEHSQSLFPQLDHQKRVIAKSMKQEPL

Sbjct 1081 RLYTVAVYFKGKRLAEASGHSIQQAEMNAAKVALEHSQSLFPQLDHQKRVIAKSMKQEPL 1140

Query 1144 NSAASKKKQNKEYYEEKRHRKYDYDSLHKKIKKENTKASSSKESSPTSRKYSSSDDKSDS 1203

NS ASKKKQ+K+YYEEK ++KYDY + KKIKK NTKA SSKESSPTSR++SSS+DKSDS

Sbjct 1141 NSTASKKKQSKDYYEEKSYKKYDYSNSRKKIKKGNTKA-SSKESSPTSRRFSSSNDKSDS 1199

Query 1204 DSESSRKIKKYKYKKSHEDNLDRMSSSDESSSDQQFKKKKVRYYSTSSTSSSEDGSSVDQ 1263

DS+S RKIKKYK+KKSHE++LDR SSDE SD++ KKKK+RYYS+SSTSSSE SSVDQ

Sbjct 1200 DSDSHRKIKKYKHKKSHENSLDR--SSDEYYSDREIKKKKIRYYSSSSTSSSEGESSVDQ 1257

Query 1264 STNSSNRSVQRYEANEELSNKYLFYTNELKSMKDQLAGVDKNSVSNGEVREKEEDSSSED 1323

STNSSN+S++ YE++EEL+ KY FYTNELKS+KDQLA K+++ N +V +KE DS SED

Sbjct 1258 STNSSNKSMKGYESDEELTKKYSFYTNELKSLKDQLAQEGKSNLLN-QVDKKEVDSLSED 1316

Query 1324 DNSSFSI 1330

+NSSFS+

Sbjct 1317 ENSSFSV 1323

Graphical representation


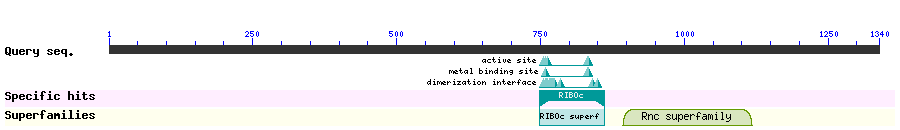


**Pasha (Partner of Drosha)**

>TRINITY_DN21663_c0_g2_i1 length=2499 nt

GCATTATAGATAAGAATAAATAAATATGTGTGTTTGAGAGGGAATGTGTATACATACACACATAAAAATAAATTTACAGGTTATGGGAGTACAAAGTTAAAAAATATTTATGCAGTAAACTGTCTTTTCTGATTCCTGCTAGGTAAATATTCTAATATAAACATAATCCATCACTAAATAAAGATTAAATTGTATATTTGATATCATATACATAAACCTTATACACATATAATCCAGATGGTCTTCAAGGTCCTTAATTTATGAATTATTGATATTAGCAAACATACAAGTATCGCTGTGTGTATATGAATTTGTATTAAAATATTATTTTTTATAAACTGTAATTTCTATTGATAATTAGCATTTTGAAATATAATACAGATTTAGAAATCTACTTTATTCAGTTCAGTTCCTGAACTAGGCAATGATGTTCCTGCAGGAGGTATAAACAAACCTATAGGCTTCACCATCATCTGCTGGTCATGAAGCTTGTTCATTTCACTCCTTAATTTGTTAATAATAGAATAATTGGGAGAATTTAAAGAAGCTTTACTCTGTAACATTGTAATCTCTTGTTCTTCTTGTTTTTTCTCTTTAACATTCCTAACAGAACGGTTACCATATAGCCTTAAAAGTGCACCCCAGCTTGTAATATGAGGGTGAAGATCCTGTAATATAGCCTGAGAAGCCCTTTGTTTTCCTTCTTTTTTGTTTTTGCAAATGATGCGTGCAGTGTGTTTTCCAACAGTCATAATAAATTCATTAGACTGCCTATGAAGAGGGTTAACTTTATAATCAATCGGCATTTCTTTGAGACCAAAATTTCTTTGTAAACATGTTAATAAAATAGAGTATGGAGAAGGTTCAGTTGTTTTTGCACAGAATTCTGCAACCCTTGGATCTTCTATCCTTATTTCATCAAAAATTGATAAACTGGTCTCTTCTTCATCTGGGAAATTAGTAATTCCACTCCTTTTTCCATTTTTATTATCACTTCTTATTTTTTCCCTCATTTCAGGAATTAATATTTCCAAAGAAGCCTTAGCAGCAGCAAGTTTTGCTTGTTTTTTACTTGTCCCTGTACCATAACCATACTTCATATCATTTATAATTACTGTAGCTGAGTAAGGAGTTGAAGGATTTTCCAATGCCTTGAATTCATAAGTTGGCTGTTTTTTAAGTGCATGCTGGACATATTCATGTAAAATGCAAATATAGCTTTTTCCATTTGGATTCATAATCCATTCTCCCCTAGGTTTTGCTTTAGGACCATCATCTGGATTTTGAATAGGGAATTTGATCAGCTTGGTTCCATCAGGAAAAGAGGGTCTGTGTTGTTCTTTTTCTATTTTTTTTGCTTTTGCATACTTACGTCTATCAGCCCATGAAACAAAATGTAACCTCTGTTCTTTTTTGAAACGGAATAGTTTTTTACAATACTGCTGTAATGTAAAGGCACCAAGGCTTTGAGAAAGTTTGTTTTCCTCGGTTGTTTCAATTTTGGCAGTGTGCAGATACTGTGGACCATCACTCTTTACATTCAAAGCATCCCCTTCTTCCTTTTTCCTTGTTTCTTCCTCATCAAGAGCACGGCGATATTGCAAACAAGGTATGGCACTCAGTGGCACATTATGCTTCCTAACACTCCCTTGCCCTAAGTAGTAAGGACGGCTAAAAGTGCATACTCTAGTTGTTTTATGTAAATAAATTGGCATTCCACTTTTATGAATGATTTTGATCCAACCTTCTGGTAACATTTCAAAATGGTTATAGCCTAGTTCTTCTAAAATTAATGTTTTATGTTCAGTAAATCTTATGCCATTTTCACCCTGGCTGTTACCATCAGATTCTAATTTTGTTTTTTTCGCTCCTTTTTCAAAACCTTCTTCCAGCATTGCTTCAATTTCATTATCACTAATATCAAAGTCTACTTCTGAATGATTATCAATATTTTCATCAATATCTGAACCTTCACATTCGTCAAGAACTTGAAATACTCTTAAATCTTCATTTTCGTAGTTTGCAGTTTCTTTAGAAAAGCTAACACTCATGGGTGATTTATTTTCTGGTAGATTGTGGTTCAAACATGGCTTTTTTACATTAGTATTGGAGTTACAGGACATGGGACTTGTAAATGATTTCCTTTTGCCCACTAATTCCATCTTTGACGTTGAAATTTCATCAGGATTCTCAGAACCTAATAGTTCGTAGTTCATCTTACATATACTCGTGTATAAGAGTTAAACTTTTTTTTATATTTATAAGTAATTAAACAACAGCTGATCTCTTTCCTATCTCAGATAGTGAACAAAAGTAAGTTAAATTTCAATAAACAACAAACAATCACCCGGAACTAAAAACTTAATTACCACTAACTCAATACTGTCTATAAAAACGTTAAGGATACATAAGAAAATCGAAATGCATTTCAAGCACCGCCTAAGATTAGGAGAGAAACGAACAATTACCTAAAGTGAAACCTTGACCAAAATGCAAAAG

Protein: RF -2: -2216 -> -384 (610 aa)

Comparison with *Halyomorpha halys,* PREDICTED: microprocessor complex subunit DGCR8 - Sequence ID: XP_014282581.1

E=0.0; bits= 1078

Query 14 ISTSKMELVGK--------RKSFTSPMSCNSNTNVKKPCLNHNLPENKSPMSVSFSKETA 65

+S SKME VG K FT SC+ V+ PCL+ PEN S F+KE

Sbjct 115 VSKSKMESVGTWKYTYQELNKQFTDSPSCSVKGTVENPCLDQKDPENVPSTSREFTKEEV 174

Query 66 NY-------------ENEDLRVFQVLDE--CEGSDIDENIDNHSEVDFDISDNEIEAMLE 110

+ ENEDLRVFQVLDE CEGSD+DE+ID SEV+FDISDNEIEAMLE

Sbjct 175 DIDISNMISNPYSFDENEDLRVFQVLDEWGCEGSDVDESIDQPSEVEFDISDNEIEAMLE 234

Query 111 EGFEKGAKKTKLESDGNSQGENGIRFTEHKTLILEELGYNHFEMLPEGWIKIIHKSGMPI 170

EGFEKG KKTK ESD NSQG+NGIR+TEHKTLILEELGYNHFEMLPEGWIKIIHKSGMPI

Sbjct 235 EGFEKGVKKTKAESDVNSQGDNGIRYTEHKTLILEELGYNHFEMLPEGWIKIIHKSGMPI 294

Query 171 YLHKTTRVCTFSRPYYLGQGSVRKHNVPLSAIPCLQYRRALDEEETRKKEEGDALNVKSD 230

YLHK TRVCTFSRPYYLGQGSVRKHNVPLSAIPCLQYRRALDEEE RKKEE D+ NVKSD

Sbjct 295 YLHKATRVCTFSRPYYLGQGSVRKHNVPLSAIPCLQYRRALDEEEIRKKEEKDSSNVKSD 354

Query 231 GPQYLHTAKIETTEENKLSQSLGAFTLQQYCKKLFRFKKEQRLHFVSWADRRKYAKAKKI 290

GPQYLHTAKIETTEENKLSQSLGAFTLQQYCKKLFRFKKEQRLHFVSWADRRKYAKAKKI

Sbjct 355 GPQYLHTAKIETTEENKLSQSLGAFTLQQYCKKLFRFKKEQRLHFVSWADRRKYAKAKKI 414

Query 291 EKEQHRPSFPDGTKLIKFPIQNPDDGPKAKPRGEWIMNPNGKSYICILHEYVQHALKKQP 350

EKEQHRPSFPDGTKLIKFPIQNPDDGPKAKPRGEWIMNPNGKSYICILHEYVQHALKKQP

Sbjct 415 EKEQHRPSFPDGTKLIKFPIQNPDDGPKAKPRGEWIMNPNGKSYICILHEYVQHALKKQP 474

Query 351 TYEFKALENPSTPYSATVIINDMKYGYGTGTSKKQAKLAAAKASLEILIPEMREKIRSDN 410

TYEFKALENPSTPYSATVIINDMKYGYGTGTSKKQAKLAAAKASLEILIPEMREKIRSDN

Sbjct 475 TYEFKALENPSTPYSATVIINDMKYGYGTGTSKKQAKLAAAKASLEILIPEMREKIRSDN 534

Query 411 KNGKRSGITNFPDEEETSLSIFDEIRIEDPRVAEFCAKTTEPSPYSILLTCLQRNFGLKE 470

KNGKRSGITNFPDEEETSLSIFDEIRIEDPRVAEFCAKTTEPSPYSILLTCLQRNFGLKE

Sbjct 535 KNGKRSGITNFPDEEETSLSIFDEIRIEDPRVAEFCAKTTEPSPYSILLTCLQRNFGLKE 594

Query 471 MPIDYKVNPLHRQSNEFIMTVGKHTARIICKNKKEGKQRASQAILQDLHPHITSWGALLR 530

MPIDYKVNPLHRQSNEFIMTVGKHTARIICKNKKEGKQRASQAILQDLHPHITSWGALLR

Sbjct 595 MPIDYKVNPLHRQSNEFIMTVGKHTARIICKNKKEGKQRASQAILQDLHPHITSWGALLR 654

Query 531 LYGNRSVRNVKEKKQEEQEITMLQSKASLNSPNYSIINKLRSEMNKLHDQQMMVKPIGLF 590

LYGNRSVRNVKEKKQEEQEITMLQSKASLNSPNYSIINKLRSEMNKLH+QQMMVKPIGLF

Sbjct 655 LYGNRSVRNVKEKKQEEQEITMLQSKASLNSPNYSIINKLRSEMNKLHEQQMMVKPIGLF 714

Query 591 IPPAGTSLPSSGTELNKVDF 610

IPPAGTSLPSSGTELNKVDF

Sbjct 715 IPPAGTSLPSSGTELNKVDF 734

Graphical representation


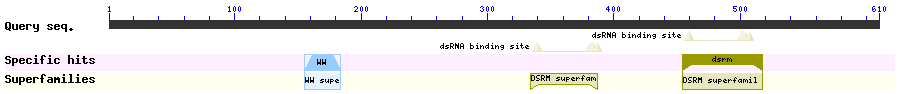


**Exportin-5**

>TRINITY_DN24852_c0_g1_i1 length= 4322 nt

CACAGGAAAACGAAAAACAAAGAAAAGGAAAAAAAAAATACAAATGCTTAAAAAACTAAAGAATAAGTTAATAATTTTGATCACATCGAATTGAGAAATTAAAATTAAATGATAGGATATGAAGTAGGAAAGTGACAGGATCACAACGCTGGAGACTAGAACTAGACGATTAGTAGAAGCTATACAAATGTGCTACGTTTTGAATAGCTTCACTAAACCATTCTCGTCGTTGTTAAATAGAAGATCTGAATTGAATTTCGGCTTTATACTGTCTATTTTGGGCAGATCTCTAATAGCAACTTTTTTCCTAAACAGCTGTCCGACGTTTCTTCCAATTAACGGGCTAGTAACTTTCTTAAACATTTCTTTTTTCGCTTTGTCGACTTTTGTTCCTTTTTGAACTTCCTTCAACATCCTGTCGTCTAGTTTTTGTAGATCAATCATATTAACATCTGGTATTTGCTTCATCACTTGAAGGATTGCTGGAGCTATCGGTCGCAAGATGCCATACATGAGGGTTCCCAGAACGAGCAGAGAACCCTGATTGGCATCGTGCTGACCATGACACTGGAGGCCAAACAAGATGGACGTAAGAGCATGGCAAGCAAGTTCTGCGGTCATCTTGCCTGCCGATACCAGGTGAAGCATGACCCCACCGAGGAGCGAAGTGCACTTAAGGCTGGCTGTACTGTCTCCCCAGTACAGAGCTCTCAATAAACAGAGTACAATAGGCTGGGAAGTCGGGTCAAAAGCGAGGACCCTCATACCCAGCTCGCTGATGATGTCCGACTGCGGCTTGACATCTTGGCCTTCCTCCTCCATACTGCCAGATCCGTTGCCGGTGTCCATTCCGCCGTACAATACCACCTTAAGAACATCTAAGTACTCCCTGGTAAGGGTTCTATTCAACATATCTTCTAGCATCTCCTGAGTGTCTGTATTCTCTTCGTGGTCGTCCGCATTACCATTTTCGCTTAATTCGGATAAATGTTGCCATCTAGCGTTCAGCCTGGCAAACATGTATGGTGTGAAATGAGCCAGGACAGGTGTTAAGACTGAATCGTAACAAGATGGTGGGCAACTAGTGACAAACGATTTCAAAAATACTCGAACAAATGGCCTCATCCTGTGGTCTGGTACAAACTCTAGATTGGAGAAGACCGTAGCTATGAGGGAGTGTGCCAAGTTGGGTACGTGGTACATATCATGGCTGAGTGCATGAAAGGCGTGGGCCAAGGCATGGCAGCAGTCCTCGTGAACATTGGAAATGAAATTCTGCATCCGTTGGAGTGGAGTTGTCTCGCCTTTGGATTCGCTGTTCATCACCTCACTGGTACTTCCAATCCCCAGCAAGTTCTGCCTTTCAAGCTCAAGCATCATATACGCTCCTCTGTAGCCCTCAGAGAGATTAGCAAGAGCAGCCGGAGATGATAAACCATTTATAACAAACGCTAGACTGAACAACCCAGGCAAGAGGGGAAGAACATGAGGAGTAGCGGGATTCCTGTAAATAGGGTTTCCTGCTTCTGTCGTACAAACGAGAAATCCTCCCCTGGCAGCGAGTTCTGGATCGCTGGGCCACATAACTCGCTTTACGACAGCAAGGATCACGTCCAAGCACCACATGATTTGTGACCTGTTTTGTCCGTTAATATCATCAGAACTAGGCTCCACGGGGGGCTTGTCTAACCCGACGTAAGACATGAACGCCGGCGTCGACATAAATGCAGTTGAAGCCATGTTATTCCAAGGACCGACGACTGGAGCCATCACTTCACCGATAAACCTACACTCTCGATCGTAGTCACATAGGTGGTTACTGATCAGAAGTAAAGCTTCCTGTAAGCACAAAGCTTCCATCGTCGAGAGCGTATGTGGTTTAGCCTGGAGGCTGACGACGATGCCGTGTATTCTCGAGAAAACTGGAAGTAGGAGAAGCGGGTATTTGTGCGCTATTTTGACCATGAGCGAGGCTGCATGCCGACGTAGGTTCTTCACTCCTCGAGATCTTTGATTTTTAGGCTCACCAGGGATAGTAAACACAAGGGTCGCAAAAATCTTGTCCAAAACTTTCGGTAAATAAATAGCTGTAGTTTCCGGAGGAGCCATACTGAGGAAAACGAAGAGTGCCGATATGCACGACAACATCGCTGAGAGGATAAGGGGATCAGTGGGCTCCAATTGTAAACATAGAACCAGGAGCTTCAATCCGTTATTGACATCGGGTCTTTCCTTCGCCATCACCAGTTTCCCAAGGACAGCATCGAGAGCCAGTGAAAGAGCTTCCCATTCCAGGTAAGCAATGGAAGTTAAAGTACATGGCTCCATATTGTTTTGCGTTACTGTCTTTTGGATCTGCACTTCCAACCACCTTTCAACGTAGGAATATGCAACCAATGGTGCAACTAAAGTAGCCTGTTTGAATGTATCAAGGAGCTCTACACGATGTTTATGAAAAAAGGCATTGTATTCATCTTCGGCATCAAAATCTTGTACAGCGTATGCCTCAGGAGTGTTCGGTTCTAGATTCGATTTCGATGAAGGGAAAGGTACCTTCATAATTTTAGGCCCAGCCGCTTCGACCCATTTTGGAACAAAACTGAGGAACACTTCATCTCTGCATATTTGATCGTGTTTCATCAAGGCGAGCCAAAGGGAATTCGCATAGCATACCAGGCTGAGGGAAGGATGACGAGTGAATGTAACGATGGCTTCTAAATAAATCTGGAAATTAGGTGGCCGAGACTGGCTATCTTTAGACCAAAGTGAGCAAAGCTGTGTACCCAGTCCTGTCAGAACACGTGTAAGTTTTTTTAAAAACTGATAATTCTTGTTCTCAGCTGATTGAAAAATGCATCTCATAGCGTCTTCACTAAATAATATCAGCAGAGGTCTCCTTTCATCGAGTTTACCTTTTCTTGATACAATCTGAAGCAAACATTCGGCAGATCCGTCTTGAAAGTTTTCATCATTTAATAGAACACAGAATATCTGAAGTAATTTACCATCTTCGGCCATGATGTGGCTCATCGAAACCCATTCAACGAAACCACTCAATGTTAATAATACAACCTGTACAACCCTAGCATGAGAGTTAGCCATTGCTACATTTCCTACTTCGAGACTTGATCGATATTGTTCATAATGGTTACCTATTAGTCCACGAAAAAACATGAACAGATCAGACATATTTGTCGTCAATGCCTGGTACAAATCTTTCCTACGTTGGTTCGATTCCAATGTCTGTAATACAGCAACATCTTCAACAAGCCTAAGTAAAACCAACAAAACCATTTCAGTCTGAATGTTCCCTTGTGAAGCACATTGATTCAGTTCCGCTAATAGAGTATTCCATTGTTGGGGCCATTCCCTTTTAATCATTTCTACAACGACACGAGACAATGCATCTCTCATATGAGCCTTATCTTCTAATGTTGTATCCATACCGCGTTCCACAAGTTTCATAGCGTTTTCCTTAATAAATAGTTTTTCGGCCTGAGTCATGTTATACCACCGGTACTTGATACAGTGCTCCATCAGTTGTAAACCGAAATGCCTAACGAAATGAGAATAATCAGCTCTTTGTGCTAAATACAAACCGCACTGAACACACAACGGGCTCTTTTCTTTGAACACTTCACAAGCATTGTAAGCTTCTAATCTTTCGGCCTGTGAAGCAAGTGGATTCATTGTCAATTCCACTGCAGCAGCCAACTGGGTAGCTACAGAACTGATTTCATTATCCATTTTCTAAGCTTTTGAGTAAACTTCAAAATTAAATGTAGTGACTAGTGCAGGAGGTGGAACTACAGATGTCTTTGTACTTTCATAGATTACACTTCTTGACAATAGTTTCAAAAGCATGTGAGAACCATGCCTTCACGTTACATATTTAATGAAATAACATTAAATAAGGCCTTCAATTTCCATGCAATGTTTAATAACGAAGTTTTGATGTATTTAGTAGAGAATGGACATGAAAAGCTGTTTGAAATAATGGTTTCCAATTAAAAACAATGAAGCTGCAGACGCATTGGTAATGTTTGATGTAATTTTTTTTTTCCTATTTGGCAAGTGATGAGGCTCAGAAATAAAGTTGAATAATGTTAAAGTAGTGTCGACAGATCCTAAGGACCGACGCTAACGAGATAAAAATTAAAACAAATTTAAGTCATCCTGAACGTATGTGCGGTCTAACCTTTCTAATTCGACTGGACCAATCTCAACCAACTCTCGATTTGTTTTGTTGGTATTCATGGAATTTCTTCATGCCGCGAAATTAAATATATTTTTAAAAAAGTATTCTCAAAATCTTGTCAAAT

Protein: RF -3: -3747 -> -193 (1184 aa)

Comparison with *Halyomorpha halys*, PREDICTED: exportin-5 - Sequence ID: XP_014280932.1

Range 1: E=0.0; bits= 2420

Query 1 MDNEISSVATQLAAAVELTMNPLASQAERLEAYNACEVFKEKSPLCVQCGLYLAQRADYS 60

MDNEISSVATQLAAAVELTMNPLASQAERLEAYNACEVFKEKSPLCVQCGLYLAQRADYS

Sbjct 1 MDNEISSVATQLAAAVELTMNPLASQAERLEAYNACEVFKEKSPLCVQCGLYLAQRADYS 60

Query 61 HFVRHFGLQLMEHCIKYRWYNMTQAEKLFIKENAMKLVERGMDTTLEDKAHMRDALSRVV 120

HFVRHFGLQLMEHCIKYRWYNMTQAEKLFIKENAMKLVE GMDTTLEDKAHMRDALSRVV

Sbjct 61 HFVRHFGLQLMEHCIKYRWYNMTQAEKLFIKENAMKLVESGMDTTLEDKAHMRDALSRVV 120

Query 121 VEMIKREWPQQWNTLLAELNQCASQGNIQTEMVLLVLLRLVEDVAVLQTLESNQRRKDLY 180

VEMIKREWPQQWNTLLAELNQCASQGNIQTEMVLLVLLRLVEDVAVLQTLESNQRRKDLY

Sbjct 121 VEMIKREWPQQWNTLLAELNQCASQGNIQTEMVLLVLLRLVEDVAVLQTLESNQRRKDLY 180

Query 181 QALTTNMSDLFMFFRGLIGNHYEQYRSSLEVGNVAMANSHARVVQVVLLTLSGFVEWVSM 240

QALTTNMSDLFMFFRGLIGNHYEQYRSSLE+GNVAMANSHARVVQVVLLTLSGFVEWVSM

Sbjct 181 QALTTNMSDLFMFFRGLIGNHYEQYRSSLELGNVAMANSHARVVQVVLLTLSGFVEWVSM 240

Query 241 SHIMAEDGKLLQIFCVLLNDENFQDGSAECLLQIVSRKGKLDERRPLLILFSEDAMRCIF 300

HIMAEDGKLLQIFCVLLNDENFQDGSAECLLQIVSRKGKLDERRPLLILFSEDAMRCIF

Sbjct 241 GHIMAEDGKLLQIFCVLLNDENFQDGSAECLLQIVSRKGKLDERRPLLILFSEDAMRCIF 300

Query 301 QSAENKNYQFLKKLTRVLTGLGTQLCSLWSKDSQSRPPNFQIYLEAIVTFTRHPSLSLVC 360

QSAENKNYQFLKKLTRVLTGLGTQLCSLWSKD QSRPPNFQIYLEAIVTFTRHPSLSLVC

Sbjct 301 QSAENKNYQFLKKLTRVLTGLGTQLCSLWSKDGQSRPPNFQIYLEAIVTFTRHPSLSLVC 360

Query 361 YANSLWLALMKHDQICRDEVFLSFVPKWVEAAGPKIMKVPFPSSKSNLEPNTPEAYAVQD 420

YANSLWLALMKHDQICRDEVFLSFVPKWVE AGPKIMKVPFPSSKSNLEPNTPE YAVQD

Sbjct 361 YANSLWLALMKHDQICRDEVFLSFVPKWVETAGPKIMKVPFPSSKSNLEPNTPEMYAVQD 420

Query 421 FDAEDEYNAFFHKHRVELLDTFKQATLVAPLVAYSYVERWLEVQIQKTVTQNNMEPCTLT 480

FDAEDEYNAFFHKHRVELLDTFKQATLVAPLVAYSYVERWLEVQIQKTVTQNN+EPCTLT

Sbjct 421 FDAEDEYNAFFHKHRVELLDTFKQATLVAPLVAYSYVERWLEVQIQKTVTQNNLEPCTLT 480

Query 481 SIAYLEWEALSLALDAVLGKLVMAKERPDVNNGLKLLVLCLQLEPTDPLILSAMLSCISA 540

S+AYLEWEALSLALDAVLGKLVMAKERPDVNNGLKLLVLCLQLEPTDPLILSAMLSCISA

Sbjct 481 SVAYLEWEALSLALDAVLGKLVMAKERPDVNNGLKLLVLCLQLEPTDPLILSAMLSCISA 540

Query 541 LFVFLSMAPPETTAIYLPKVLDKIFATLVFTIPGEPKNQRSRGVKNLRRHAASLMVKIAH 600

LFVFLSMAPPETTA+YLPKVLDKIFATLVFTIPGEPKNQRSRGVKNLRRHAASLMVKIA

Sbjct 541 LFVFLSMAPPETTAVYLPKVLDKIFATLVFTIPGEPKNQRSRGVKNLRRHAASLMVKIAQ 600

Query 601 KYPLLLLPVFSRIHGIVVSLQAKPHTLSTMEALCLQEALLLISNHLCDYDRECRFIGEVM 660

KYPLLLLPVFSRIHGIV+SLQAKPHTLSTMEALCLQEALLLISNHLCDYDRECRFIGEVM

Sbjct 601 KYPLLLLPVFSRIHGIVISLQAKPHTLSTMEALCLQEALLLISNHLCDYDRECRFIGEVM 660

Query 661 APVVGPWNNMASTAFMSTPAFMSYVGLDKPPVEPSSDDINGQNRSQIMWCLDVILAVVKR 720

+PV+GPWNNMASTAFMSTPAFMSYVGLDKPPVEPSSDDINGQNRSQIMWCLDVILAVVKR

Sbjct 661 SPVIGPWNNMASTAFMSTPAFMSYVGLDKPPVEPSSDDINGQNRSQIMWCLDVILAVVKR 720

Query 721 VMWPSDPELAARGGFLVCTTEAGNPIYRNPATPHVLPLLPGLFSLAFVINGLSSPAALAN 780

VMWPSDPELAARGGFLVCTTEAGNPIYRNPATPHVLPLLPGLFSLAFVINGLSSPAALAN

Sbjct 721 VMWPSDPELAARGGFLVCTTEAGNPIYRNPATPHVLPLLPGLFSLAFVINGLSSPAALAN 780

Query 781 LSEGYRGAYMMLELERQNLLGIGSTSEVMNSESKGETTPLQRMQNFISNVHEDCCHALAH 840

LSEGYRGAY MLELERQNLLGIGSTSEVMNSESKGETTPLQRMQNFISNVHEDCCHALAH

Sbjct 781 LSEGYRGAYTMLELERQNLLGIGSTSEVMNSESKGETTPLQRMQNFISNVHEDCCHALAH 840

Query 841 AFHALSHDMYHVPNLAHSLIATVFSNLEFVPDHRMRPFVRVFLKSFVTSCPPSCYDSVLT 900

AFHALSHDMYHVPNLAHSLIATVFSNLEFVPDHRMRPFVRVFLKSFVTSCPPSCYD+VLT

Sbjct 841 AFHALSHDMYHVPNLAHSLIATVFSNLEFVPDHRMRPFVRVFLKSFVTSCPPSCYDTVLT 900

Query 901 PVLAHFTPYMFARLNARWQHLSELSENGNADDHEENTDTQEMLEDMLNRTLTREYLDVLK 960

PVLAHFTPYMFARLNARWQHLSELSENGN DDHEENTDTQEMLEDMLNRTLTREYLDVLK

Sbjct 901 PVLAHFTPYMFARLNARWQHLSELSENGNTDDHEENTDTQEMLEDMLNRTLTREYLDVLK 960

Query 961 VVLYGGMDTGNGSGSMEEEGQDVKPQSDIISELGMRVLAFDPTSQPIVLCLLRALYWGDS 1020

VVLYGG +TGNGSGSMEEEGQ+VKPQSD+ISELGMRVLAFDPTSQPIVLCLLRALYWGDS

Sbjct 961 VVLYGGTETGNGSGSMEEEGQEVKPQSDVISELGMRVLAFDPTSQPIVLCLLRALYWGDS 1020

Query 1021 TASLKCTSLLGGVMLHLVSAGKMTAELACHALTSILFGLQCHGQHDANQGSLLVLGTLMY 1080

TASLKCTSLLGGVMLHLVSAGKMTA+LACHALTSILFGLQCHGQHDANQGSLLVLGTLMY

Sbjct 1021 TASLKCTSLLGGVMLHLVSAGKMTADLACHALTSILFGLQCHGQHDANQGSLLVLGTLMY 1080

Query 1081 GILRPIAPAILQVMKQIPDVNMIDLQKLDDRMLKEVQKGTKVDKAKKEMFKKVTSPLIGR 1140

GILRPIAP I+QVMKQIPDVNMIDLQKLDDRMLKE+QKGTKVDKAKKEMFKKVTSPLIGR

Sbjct 1081 GILRPIAPEIVQVMKQIPDVNMIDLQKLDDRMLKEIQKGTKVDKAKKEMFKKVTSPLIGR 1140

Query 1141 NVGQLFRKKVAIRDLPKIDSIKPKFNSDLLFNNDENGLVKLFKT 1184

NVGQLFRKKVAIRDLPKI+S+KPK NSDLLFNNDENGLV LFKT

Sbjct 1141 NVGQLFRKKVAIRDLPKIESLKPKLNSDLLFNNDENGLVTLFKT 1184

Graphical representation


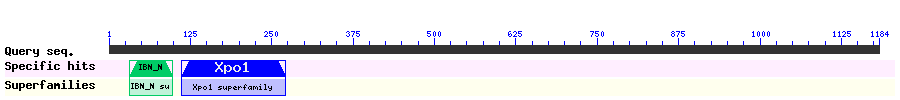


**Dicer-2 isoform 1**

>TRINITY_DN28568_c3_g1_i1 length= 5311 nt

GTACATTATAACGGTGAGTGCTGCTATATATTGATGTACACACTTAAAAGTGTTTATAATTTATATTTAAAAATTATAACTTCTTAAATAAATTTTTAAAATAAAATGACTTTCAACATCATCATTTTCATTTTGAAATTAAAAATGGAGATTATTTACAATAAAAAATATTAAAACTTTAAAAATATGTAATAAAACAAAAATTATTCACTATTGAATCATATTGGATCCAGATAATAATGCCTCAGAGCTACTTTAGCAGCAGCTAGTTTAGCTTGGTTTTTGTTCTCTCCTACTCCAACAAAGGCCTTCCTATCGTTCTTATTGGTAACAATGAGAGGCACCATGACCATGGTTTTGTTATCATTTAGAAATTGTGGTTTCTCAAAACATGGTGCTGGAACAGGAAATTTTTCATAAAGCAGTCGAATGCTGTTCTTAGGAATATTTCCAATGAATTCCTCTATTTCATTGTGCATTAACTTATAGAAAATTTTCCAAACAGTATTTAAAGATTTTCCACTATCTAAGTAAACAGCTGCAGCTAATGATTCAAACAAGTCACCAAGAACCTTCGGGACATCCACAATTTCAGCAGCTTGCACATCATTTTCACTCAGAAGGAACAATATCTCGGCACCAATTTTATGATTCCTTTTCTCTTGATGCTCAACAAATCTCTTTATGATATCATTTAGCTTACAGGCTTTGGTAAGCATGAATTTATGGAATCCATATCTTACAGTCAAGCAAGCGAATGTAACATTATTCACCAAGGCAGATCTGAGATCTGTTAATTCACCGGGTGAAATATTCTTGCATCTAGTATATATATAGGAAGTTATCAAAAAGTCTAAAATGGCATCACCCAGGAATTCCAATCGTTGATAACAATCAGTATAGTTCTGGCGGAAGGAACAATGAGTAAGAGCTTGTAAGAGATAACTTCTGTTTTTGAATCTGTAGCCCAAAATTGATTCTATGGCCTCGGGCATGATGAGAACTTCATTGACGTTACCACCATCAAATATTAGAGCAGTGGGTGCTGGTGCATATAATACTTTGTTAATATCAGACATATTTTCAGGAAGAATTTTCAGCCATTTACAGAGTTTAAAAGCTCCTTCAACACCACATGTCTCAAGATAAACTCCTATAAGGGCTTCTATACAATCAGCAATAACTTTATCAACTACGCAGTGCTGTCCAATTACTCCTAAGCTGCTATGGGTAGTTTCATCAGCTGGTGACGAATTACTGAACAACTCATGAATTTTTGACATTGTTTCTTCACTCAAAATAGCAGTATCTCTCTCTTTTTTTGTAAGATTTATTTGGAAGAGGACAGATGGCGGAATCTTAGCATCTTTAATAATTTGTTTAATGCTTTCCAAAAGTGTGAATCCTGGGACTTCCCAGTCATCAGGAATAAAGTCATTAACCTTCAAAATTTTTCCAAGATTTAAATACTTTCCACTATAATAGAGATTTCTATTGCCCACTATTTTCCCCTTGATGTTGCTTAGCTTGCCTTCGTCCTTGCTGTCAAATTGAATGAATAAAATTAAGGAAACTAAAAATTTCAAATAAGAGTCACCTAACGTTTCTAGCCTCTCATAATTCATGACATCATTAGAAGAACTAGCAGTAAGAGCCTTGACCATAGAACTCAGTTGAGGCCCCAATTGACAGTTCTTATTTAACAAAATTGGAATTTCAGGGGCATTAACTTTTGGTGGCATTATTATACTCCTATTGATTTGGACAGGAGTAACTTCCATTTTGGCAAATCTTTCATAGTGAATTACAGAAAGCAAATCTAGATTTCTCAAATTTCTTTCTATATCAATCGGTTCTTCCTCTTTATCCCATGTGCAATCTTTAGGTTGTAGCTCAACCATCTTATCCGCTAACATATTACTCACCTCAATAGTACTTCTAGCATTTGAACTTTCATCACCAGCTAAGGCTGTATCATCGACTTCTAAAGGTTGCCAATAAACACCTTTTGGAAGTTTTACAACTCCTAATCCTGTTTCTTCAGCAATTTTCTGACGAAGCTCTTCAGCATTCAACAGCATAGTAAGGCGATGCAATACTGATGGTAACAAAGTAACCTTCAACATATAAACTGCAGGGAACTCTAAAAGAGTGCACAGTTCAGGCACTAATGTCTCTTCAAAATCTTCTTCATCGCGACGTCTTTTCTTACCCAAGCGGTATTTCCCCCTTGGTAATAAACAGTTGTTTTTTCCCGAGATGGCTCTCACTTCAATCAATTTCTGATTTTTATTAACAATTGATTGATTGTAACGTTTAAAAAAATATTCAGAATATGAACTGTATTGCTCTGATGGAAAGGGTGACATGGGTGTTTGATCTTCACATACTCTTGTTACAACATATTTCAGCATGTACCCTTTTGGGCGATACCATGGTATGACTATCTTATTGAGGTATTTTTCTTGGGTCACGTCTATGCTCCCTCTAGAATCCAAGGGTACTGTTTTGACTGGAGGAATATGTCCATGATTATTTATAGTCTCCCAATCTATTGTAATGTCATCATTGTCAACAATAGTAGGAACTACATAATAGGAGTTTTCTTTGGTTCCATACTCTTTAACTAGAAATTCTTTCTTTAAAGTCCAAGCCTCTGTAAATATCAAAGAATGGAAAGCCACAATTTTATCAAAATTTTCACTGCTGAGAGTAATCCTCCGAGCATTTTGTTTTATATTAACTGCCACTTCACCAACATTCATGAATAATGGGAAATCACAGAGCTTAGGCCATTTCTTTGTAGTGAGCACGGAGAAATTTTTATTGGATTGTAATAAATTATAAAATGCTAATAACCTGGCATTATCAGGCTTTGGATAGTTTGGTGTTATTTTAATGACATGGAGGTAAACAGAACTATCTGCTCGAGGAAAACCACCACAGATCCACTTAGGAAACTGTTTATCATAAATCCTACGACATTTTTTTGTTCCAGCTTTAAAATAGGAACTTCCATCATCATTTTCCCAAAGAGGAAATAAAATCTTTTCTTCGACATCCTGAACATTATTTCCGATAGGGAGTAAATGATCATTAAGCTCACCCATCTCGTATAACATTTTACATGCATTCAATGCAACTGCTCCCTTGGCAGTTACAATGTTTTCACATGGCTGCCCCACAATAATCCCTTTAAGTGGAGAATTCACTGGCAACTGAAGATAGCAGATAATTTTTACTTTGCCATCTTCAAGAATTTCTTTCTTCCAAAAAAATACAGTAAGTCGGGTGAATTTATCAGAAGGCAAAGTAAAACAATACCTATTCAACAAAGAAACAGCAGATTCGACTGTCACTTTTGGTCCAGATGGCCCATTGATGCAGAAAGGTTCGACTTCTTTGGTGTAAAGAAACAACTCGGCTTCTGCTTCAGTCCCACCCCTTCGAAGTAGATCTTTGCCATAGCATAATTCCTGCTTTAGAGTTTCTTCAGATTCTTTGAAGGTATAATATTTTTTATGAAATGGATCAGCATTTGATTTTGGGGCAAATACAATGTATTTACTATCTCTGTGTCTTGCACGGCCTTTGGACTGAATATACGACCGAGTTGTTTTAGGATGATCAAATTTTATTACGTAATTGCAATTTTTCACATCAATACCTTCTTCGATCACATCAGAAGCAACTAGTATATTGGTCTCACCAATATTAAATTTTTTCAAGATCTTCTCATTCTTTTTCCTTTCAAGAATATATTCCCGAGTGATATGATAAGGGTTGCTATTTACACCAACAACAAAATCTGGCTTTATGTATTTATATTCTTCAATGGAAGCCGCTAAATCATCAAGAACATAGTACAAAACTTTAGCAGTTGTTTTTCTTTCAACAAACACCAAAGCTAAATTATCTTCTTTGAAATTCTTTAGTAGTTTGAACAATTCAATCATCTTTGGAGTTGAATATACAAATATTTTTCCAATTGTTTTTTTCATAATCAAATCAAGCTTCTTCCGAATTAGATGAAGCTGTATTATTATGTGACCTAAGAGTTTTATAAGTGCTAGGTCACTAGACGAAATTCTCATTCTTTCAAAGTGAACTATTCGTAACAATAGTGCATAAGAAGCAGGATAACCTCCTAATTCTTTGGTGATAATGGTTATTTCTTTGAGAATATTCTGGATTTCATTGATGGTATCTGATTTGTTGTTCATCAAGACAGCATCTGGAGGGTTGCTGGATGGCTTCGGAGAACTATCCAGTAGAACAATTGCATCCAGAATTGGGTAAATACTATGTAATGCTGAATACACAACTTTGTGACATTCATCCTCTTCATCAACATCATAATTAACTATTTCTTCTTTGGGATTAGTTGAAAATGCCAGAATCATTTCTTCATTTTCAGCTCTGGCTATTCTAGAATGCAATGTGATTTCCAAGTTATGGATTTCTCTTGATATACGATCTGGTTTACAATTAGAATTTAATAGCGTTGCAGTTAAACCTAGTACATGCGGTCTGTCCTCTTTAGGGATACCATTAAACTGATCCATTATCATTCTCATTGGATGTTTGTTAACAGCATGATGACATTCATCTATAACTATCAAATTTACTCGAGAAAATGAGATGTAATTGTGGAGAATCATATTATGAAATATAGCAGCAGTCATAACCAAAACCTGATTATTTTCAAGCTCCTCATGCCATTGGTTGATGTCCCAGTGATCCACTCCTTTGTCTCCAGTATATTCTCCAACTGTAAGCGGTGTTTGCTGTCTTATACTTTGAGCTTGTTGGGCTACTAATGCAACAGTATCAACAAGAAAAAAAGTTCTTTTCCCTCCTTCTGAATACAATAATTTTAGTTCATTTGATTTATGCTTAATAAGCATAACAGCGATGAATGTTTTGCCAGAGCCAGTTGGCAAAAAAATTATAGTGTTCTCCTTCATCACTCTTTCAAATAATTCAACTTGGTACTCCCTAGGTTTAAAATCTTCAATTTTTTTGTCCATTTTTAATACGTTCAGAAATTTGAAGAATATATGTCTTTTGGAGAAACAATAAACAGACAAACAAATATAAGTCTAAGACTAATGCCAGAGACACTCAAAACAAAACAAAGGTGGTGAGATGGGGTACAAACCCGGGCGCCAGCGTCTTATCACGATAAGGTTTGCATTGTAACTGACCCCCACTACCATCTCTTCGTCAAACCATCTATTATTTTTATGTAGAAAAACCACCTTAGTTACTGGAAGATTTTTTTTAATTTTTATTCTATTTTATGTAATAA

Protein: RF -3: -5030 -> -219 (1602 aa)

Comparison with *Halyomorpha halys*, PREDICTED: endoribonuclease Dicer isoform X1 - Sequence ID: XP_014275310.1

E=0.0; bits= 2795

Query 2 DKKIEDFKPREYQVELFERVMKENTIIFLPTGSGKTFIAVMLIKHKSNELKLLYSEGGKR 61

+K IEDFKPREYQVELFERVMK NTIIFLPTGSGKTFIAVM+IKHKSN+LKLLYSEGGKR

Sbjct 17 NKNIEDFKPREYQVELFERVMKHNTIIFLPTGSGKTFIAVMVIKHKSNDLKLLYSEGGKR 76

Query 62 TFFLVDTVALVAQQAQSIRQQTPLTVGEYTGDKGVDHWDINQWHEELENNQVLVMTAAIF 121

TFFLV+TVALVAQQAQSIRQQTPLTVGEY+ +KGVDHWDINQWHEELEN+QVLVMTAAIF

Sbjct 77 TFFLVNTVALVAQQAQSIRQQTPLTVGEYSAEKGVDHWDINQWHEELENHQVLVMTAAIF 136

Query 122 HNMILHNYISFSRVNLIVIDECHHAVNKHPMRMIMDQFNGIPKEDRPHVLGLTATLLNSN 181

H MILH+YI+ RVNL+VIDECHHAVNKHPM+++M++F G+P +DRPHVLGLTATLLNSN

Sbjct 137 HQMILHDYITLPRVNLLVIDECHHAVNKHPMKLVMEEFKGVPTKDRPHVLGLTATLLNSN 196

Query 182 CKPDRISREIHNLEITLHSRIARAENEEMILAFSTNPKEEIVNYDVDEEDECHKVVYSAL 241

CKP+RI EIHNLEIT+H++IARAENEEMI AFSTNPKEE+V Y+++E+DEC VV S++

Sbjct 197 CKPERIPTEIHNLEITMHAKIARAENEEMIQAFSTNPKEEVVIYNIEEDDECFAVVKSSI 256

Query 242 HSIYPILDAIVLLDSSPKPSSNPPDAVLMNNKSDTINEIQNILKEITIITKELGGYPASY 301

IYPIL IVL+DSSPKP S+PP+A L++NKSDTI EI NILKEI +IT+ELGGY ++Y

Sbjct 257 DEIYPILKLIVLVDSSPKPPSSPPNATLVDNKSDTIIEITNILKEIVLITRELGGYASTY 316

Query 302 ALLLRIVHFERMRISSSDLALIKLLGHIIIQLHLIRKKLDLIMKKTIGKIFVYSTPKMIE 361

LLLRIVHFERMRIS+SD AL+KLLGHIIIQLHLIRKKL+LIMK GKIF YST KMIE

Sbjct 317 VLLLRIVHFERMRISASDQALVKLLGHIIIQLHLIRKKLELIMKNRPGKIFHYSTSKMIE 376

Query 362 LFKLLKNFKEDNLALVFVERKTTAKVLYYVLDDLAASIEEYKYIKPDFVVGVNSNPYHIT 421

LFKLL+NF+E +LALVFVERKTTAKVLYYVLDDLAASIEEYKYIKPDF+VGVNSNPYHIT

Sbjct 377 LFKLLQNFEEKSLALVFVERKTTAKVLYYVLDDLAASIEEYKYIKPDFIVGVNSNPYHIT 436

Query 422 REYILERKKNEKILKKFNIGETNILVASDVIEEGIDVKNCNYVIKFDHPKTTRSYIQSKG 481

REYILERKKN KILKKFN+GE NILVASDVIEEGIDVKNCN+VIKFDHPKT RSYIQSKG

Sbjct 437 REYILERKKNHKILKKFNMGEINILVASDVIEEGIDVKNCNFVIKFDHPKTARSYIQSKG 496

Query 482 RARHRDSKYIVFAPKSNADPFHKKYYTFKESEETLKQELCYGKDLLRRGGTEAEAELFLY 541

RARHRDSKYIVFAP SN++ FH+ Y TFK+SEETLKQELCYG+D RRGGTEAEAELFLY

Sbjct 497 RARHRDSKYIVFAPSSNSNAFHRNYITFKQSEETLKQELCYGRDQSRRGGTEAEAELFLY 556

Query 542 TKEVEPFCINGPSGPKVTVESAVSLLNRYCFTLPSDKFTRLTVFFWKKEILEDGKVKIIC 601

+KEVEPF I+GP GPKVTVESA+SLLNRYCFTLP DKFTRLTVFFWKKE++ D K K +C

Sbjct 557 SKEVEPFFIDGPIGPKVTVESAISLLNRYCFTLPCDKFTRLTVFFWKKEVIVDNKPKTLC 616

Query 602 YLQLPVNSPLKGIIVGQPCENIVTAKGAVALNACKMLYEMGELNDHLLPIGNNVQDVEEK 661

+LQLP+N PLKGII GQPCEN+VTAKGAVALNACKMLY++GELN+HLLP+GN VQ+VEEK

Sbjct 617 FLQLPINCPLKGIIEGQPCENLVTAKGAVALNACKMLYKIGELNEHLLPVGNEVQNVEEK 676

Query 662 ILFPLWEND-DGSSYFKAGTKKCRRIYDKQFPKWICGGFPRADSSVYLHVIKITPNYPKP 720

IL PLWE++ D S K GTKK RRIYDKQFP+WICGGFP+ VY+HV+KI PNYPKP

Sbjct 677 ILCPLWEDEKDQKSLHKPGTKKSRRIYDKQFPRWICGGFPQVGQPVYIHVLKILPNYPKP 736

Query 721 DNARLLAFYNLLQSNKNFSVLTTKKWPKLCDFPLFMNVGEVAVNIKQNARRITLSSENFD 780

+N RLLAFYNLL SNKNF+VL++KKWPKLC+FPLFMNVGEV VNI+QNAR+ITL+ E +

Sbjct 737 ENDRLLAFYNLLLSNKNFAVLSSKKWPKLCNFPLFMNVGEVRVNIQQNARKITLNQEQYK 796

Query 781 KIVAFHSLIFTEAWTLKKEFLVKEYGTKENSYYVVPTIVDNDDITIDWETINNHGHIPPV 840

KI+AFHSL+FTEAW+LKKEFLV+EY KENSYYVVP IVDND ITIDWETI NHG IPPV

Sbjct 797 KILAFHSLLFTEAWSLKKEFLVREYENKENSYYVVPAIVDNDSITIDWETIKNHGEIPPV 856

Query 841 KTVPLDSRGSIDVTQEKYLNKIVIPWYRPKGYMLKYVVTRVCEDQTPMSPFPSEQYSSYS 900

K VPLDSRG I VT++KYLNKIVIPWYRPKG+MLKYVVTRVCE+QTPMSPFPSEQYSSYS

Sbjct 857 KAVPLDSRGDIIVTKDKYLNKIVIPWYRPKGFMLKYVVTRVCEEQTPMSPFPSEQYSSYS 916

Query 901 EYFFKRYNQSIVNKNQKLIEVRAISGKNNCLLPRGKYRLGKKRRRDEEDFEETLVPELCT 960

+YFFKRYNQS+VNKNQKLIEVRAISGKNNCLLPRGKYRLGKKRRRDEEDFEETLVPELCT

Sbjct 917 DYFFKRYNQSVVNKNQKLIEVRAISGKNNCLLPRGKYRLGKKRRRDEEDFEETLVPELCT 976

Query 961 LLEFPAVYMLKVTLLPSVLHRLTMLLNAEELRQKIAEETGLGVVKLPKGVYWQPLEVDDT 1020

LLEFPAVYMLK+TLLPS+LHR+ +LLNAEELRQKIA ETGLGVVKLP GVYW LEVDDT

Sbjct 977 LLEFPAVYMLKITLLPSILHRINILLNAEELRQKIAAETGLGVVKLPSGVYWSSLEVDDT 1036

Query 1021 ALAGDESSNARSTIEVSNMLADKMVELQPKDCTWDKEEEPIDIERNLRNLDLLSVIHYER 1080

ALAGDESSNARSTIEVSNMLADKMVELQPKDCTWDKEEEPIDIERNLRNLDLLSV+HYE+

Sbjct 1037 ALAGDESSNARSTIEVSNMLADKMVELQPKDCTWDKEEEPIDIERNLRNLDLLSVLHYEK 1096

Query 1081 FAKMEVTPVQINRS---------------IIMPPKVNAPEIPILLNKNCQLGPQLSSMVK 1125

FAK+E++ QI+RS I+ PPKVNAPEIPIL +KNC LGP+L S++K

Sbjct 1097 FAKIEISADQIDRSNGMVSERGSSVKKSNIMRPPKVNAPEIPILSDKNCVLGPELGSILK 1156

Query 1126 ALTASSSNDVMNYERLETLGDSYLKFLVSLILFIQFDSKDEGKLSNIKGKIVGNRNLYYS 1185

ALTASSSNDVMNYERLETLGDS+LKFLVSLILF+ F++KDEGKLS+IKGKI+GNRNLYYS

Sbjct 1157 ALTASSSNDVMNYERLETLGDSFLKFLVSLILFVHFENKDEGKLSSIKGKIIGNRNLYYS 1216

Query 1186 GKYLNLGKILKVNDFIPDDWEVPGFTLLESIKQIIKDAKIPPSVLFQINLTKKERDTAIL 1245

GKYL+LGKILKVNDFIPDDWEVPGFT+LESIK++I +AKIPPSVL+QI+LTKKERDTA+L

Sbjct 1217 GKYLSLGKILKVNDFIPDDWEVPGFTILESIKKVIINAKIPPSVLYQISLTKKERDTAVL 1276

Query 1246 SEETMSKIHELFSNSSPADETTHSSLGVIGQHCVVDKVIADCIEALIGVYLETCGVEGAF 1305

+EET SKIHELF NSSPADE+THSSLGVIGQ CVVDKVIAD +EALIGVYLE CG+EGAF

Sbjct 1277 NEETRSKIHELFCNSSPADESTHSSLGVIGQQCVVDKVIADAVEALIGVYLEACGIEGAF 1336

Query 1306 KLCKWLKILPENMSDINKVLYAPAPTALIFDGGNVNEVLIMPEAIESILGYRFKNRSYLL 1365

KLCKWLKILPENM DI+KVLY PAP+A I D G+VN+VL+MPE IESILGYRF+NRSYLL

Sbjct 1337 KLCKWLKILPENMCDIDKVLYEPAPSAQILDSGDVNDVLVMPEVIESILGYRFRNRSYLL 1396

Query 1366 QALTHCSFRQNYTDCYQRLEFLGDAILDFLITSYIYTRCKNISPGELTDLRSALVNNVTF 1425

QALTHCSFRQN+TDCYQRLEFLGDAILDFLITS+IY +CKNISPGELTDLRSALVNNVTF

Sbjct 1397 QALTHCSFRQNFTDCYQRLEFLGDAILDFLITSHIYNKCKNISPGELTDLRSALVNNVTF 1456

Query 1426 ACLTVRYGFHKFMLTKACKLNDIIKRFVEHQEKRNHKIGAEILFLLSENDVQAAEIVDVP 1485

ACLTVRYGFHKFMLTK CKL DIIKRFVEHQEKRNHKIGAEILFLLSENDV+AAEIVDVP

Sbjct 1457 ACLTVRYGFHKFMLTKTCKLTDIIKRFVEHQEKRNHKIGAEILFLLSENDVEAAEIVDVP 1516

Query 1486 KVLGDLFESLAAAVYLDSGKSLNTVWKIFYKLMHNEIEEFIGNIPKNSIRLLYEKFPVPA 1545

KVLGDLFESLAAAVYLDSGKSLNTVWKIFYKLMHNEIEEF+ NIPKNSIRLLYEKFP+PA

Sbjct 1517 KVLGDLFESLAAAVYLDSGKSLNTVWKIFYKLMHNEIEEFMRNIPKNSIRLLYEKFPIPA 1576

Query 1546 PCFEKPQFLNDNKTMVMVPLIVTNKNDRKAFVGVGENKNQAKLAAAKVALRHYYLDPI 1603

PCFEKP+FLN+N+T+VMVPLIVT KNDR F+GVGENKNQAKLAAAKVALRHYYLDP+

Sbjct 1577 PCFEKPRFLNENQTIVMVPLIVTAKNDRMRFIGVGENKNQAKLAAAKVALRHYYLDPM 1634

Graphical representation


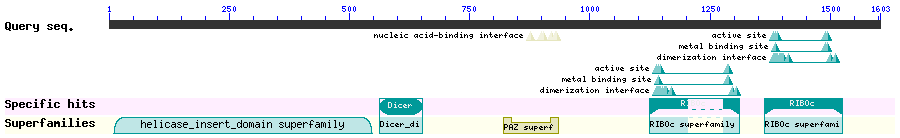


**Dicer-2 isoform 2**

> TRINITY_DN28568_c3_g1_i2 length= 2175 nt

GTACATTATAACGGTGAGTGCTGCTATATATTGATGTACACACTTAAAAGTGTTTATAATTTATATTTAAAAATTATAACTTCTTAAATAAATTTTTAAAATAAAATGACTTTCAACATCATCATTTTCATTTTGAAATTAAAAATGGAGATTATTTACAATAAAAAATATTAAAACTTTAAAAATATGTAATAAAACAAAAATTATTCACTATTGAATCATATTGGATCCAGATAATAATGCCTCAGAGCTACTTTAGCAGCAGCTAGTTTAGCTTGGTTTTTGTTCTCTCCTACTCCAACAAAGGCCTTCCTATCGTTCTTATTGGTAACAATGAGAGGCACCATGACCATGGTTTTGTTATCATTTAGAAATTGTGGTTTCTCAAAACATGGTGCTGGAACAGGAAATTTTTCATAAAGCAGTCGAATGCTGTTCTTAGGAATATTTCCAATGAATTCCTCTATTTCATTGTGCATTAACTTATAGAAAATTTTCCAAACAGTATTTAAAGATTTTCCACTATCTAAGTAAACAGCTGCAGCTAATGATTCAAACAAGTCACCAAGAACCTTCGGGACATCCACAATTTCAGCAGCTTGCACATCATTTTCACTCAGAAGGAACAATATCTCGGCACCAATTTTATGATTCCTTTTCTCTTGATGCTCAACAAATCTCTTTATGATATCATTTAGCTTACAGGCTTTGGTAAGCATGAATTTATGGAATCCATATCTTACAGTCAAGCAAGCGAATGTAACATTATTCACCAAGGCAGATCTGAGATCTGTTAATTCACCGGGTGAAATATTCTTGCATCTAGTATATATATAGGAAGTTATCAAAAAGTCTAAAATGGCATCACCCAGGAATTCCAATCGTTGATAACAATCAGTATAGTTCTGGCGGAAGGAACAATGAGTAAGAGCTTGTAAGAGATAACTTCTGTTTTTGAATCTGTAGCCCAAAATTGATTCTATGGCCTCGGGCATGATGAGAACTTCATTGACGTTACCACCATCAAATATTAGAGCAGTGGGTGCTGGTGCATATAATACTTTGTTAATATCAGACATATTTTCAGGAAGAATTTTCAGCCATTTACAGAGTTTAAAAGCTCCTTCAACACCACATGTCTCAAGATAAACTCCTATAAGGGCTTCTATACAATCAGCAATAACTTTATCAACTACGCAGTGCTGTCCAATTACTCCTAAGCTGCTATGGGTAGTTTCATCAGCTGGTGACGAATTACTGAACAACTCATGAATTTTTGACATTGTTTCTTCACTCAAAATAGCAGTATCTCTCTCTTTTTTTGTAAGATTTATTTGGAAGAGGACAGATGGCGGAATCTTAGCATCTTTAATAATTTGTTTAATGCTTTCCAAAAGTGTGAATCCTGGGACTTCCCAGTCATCAGGAATAAAGTCATTAACCTTCAAAATTTTTCCAAGATTTAAATACTTTCCACTATAATAGAGATTTCTATTGCCCACTATTTTCCCCTTGATGTTGCTTAGCTTGCCTTCGTCCTTGCTGTCAAATTGAATGAATAAAATTAAGGAAACTAAAAATTTCAAATAAGAGTCACCTAACGTTTCTAGCCTCTCATAATTCATGACATCATTAGAAGAACTAGCAGTAAGAGCCTGAAAAAGATGGATAAACAACTTTTATACACCATTATATATATTTTATCAGTTATAACAAGTAATATGTTAATTTGCACTTCCTTGGGATGACAATGACCTAATAGAACAATGATGTGAACATTAGTAGGTCATTAAAAAAGTCCTTTTACTTTATTTCAGATTATATAATTTAAACATAATTGAATTTACTTATATAATTGAATATATACCTCTTCAAGGTAAAGTTCATTGTTATAAATTACTTCCACCCATCTTTCTGCGAGTAATTGGATTTGATGACAGTACCACTCCCCGAGTGCCTGGGAAAGCCCACTGCTCGACCATCAAGAAAAAGCTCAAAATGTAAAACGACTTTAAAGTTCTACTAAACATAAAACATCACTTTTTTTTCAAACCAATTTAAATGCAACAAAGTGCAGCAATATTTTGATGGTTAACCCAGCAAAAATTTTTTCTTTGGCTGCCATAGAATGGCAAATTATCCCTTAATGCAAACTCAAGTCAAAAGGAATTAGAG

Protein: RF -2: -1625 -> -210 (468 aa)

Comparison with *Halyomorpha halys*, PREDICTED: endoribonuclease Dicer isoform X2 - Sequence ID: XP_014275311.1

E=0.0; bits= 852

Query 1 MNYERLETLGDSYLKFLVSLILFIQFDSKDEGKLSNIKGKIVGNRNLYYSGKYLNLGKIL 60

MNYERLETLGDS+LKFLVSLILF+ F++KDEGKLS+IKGKI+GNRNLYYSGKYL+LGKIL

Sbjct 1164 MNYERLETLGDSFLKFLVSLILFVHFENKDEGKLSSIKGKIIGNRNLYYSGKYLSLGKIL 1223

Query 61 KVNDFIPDDWEVPGFTLLESIKQIIKDAKIPPSVLFQINLTKKERDTAILSEETMSKIHE 120

KVNDFIPDDWEVPGFT+LESIK++I +AKIPPSVL+QI+LTKKERDTA+L+EET SKIHE

Sbjct 1224 KVNDFIPDDWEVPGFTILESIKKVIINAKIPPSVLYQISLTKKERDTAVLNEETRSKIHE 1283

Query 121 LFSNSSPADETTHSSLGVIGQHCVVDKVIADCIEALIGVYLETCGVEGAFKLCKWLKILP 180

LF NSSPADE+THSSLGVIGQ CVVDKVIAD +EALIGVYLE CG+EGAFKLCKWLKILP

Sbjct 1284 LFCNSSPADESTHSSLGVIGQQCVVDKVIADAVEALIGVYLEACGIEGAFKLCKWLKILP 1343

Query 181 ENMSDINKVLYAPAPTALIFDGGNVNEVLIMPEAIESILGYRFKNRSYLLQALTHCSFRQ 240

ENM DI+KVLY PAP+A I D G+VN+VL+MPE IESILGYRF+NRSYLLQALTHCSFRQ

Sbjct 1344 ENMCDIDKVLYEPAPSAQILDSGDVNDVLVMPEVIESILGYRFRNRSYLLQALTHCSFRQ 1403

Query 241 NYTDCYQRLEFLGDAILDFLITSYIYTRCKNISPGELTDLRSALVNNVTFACLTVRYGFH 300

N+TDCYQRLEFLGDAILDFLITS+IY +CKNISPGELTDLRSALVNNVTFACLTVRYGFH

Sbjct 1404 NFTDCYQRLEFLGDAILDFLITSHIYNKCKNISPGELTDLRSALVNNVTFACLTVRYGFH 1463

Query 301 KFMLTKACKLNDIIKRFVEHQEKRNHKIGAEILFLLSENDVQAAEIVDVPKVLGDLFESL 360

KFMLTK CKL DIIKRFVEHQEKRNHKIGAEILFLLSENDV+AAEIVDVPKVLGDLFESL

Sbjct 1464 KFMLTKTCKLTDIIKRFVEHQEKRNHKIGAEILFLLSENDVEAAEIVDVPKVLGDLFESL 1523

Query 361 AAAVYLDSGKSLNTVWKIFYKLMHNEIEEFIGNIPKNSIRLLYEKFPVPAPCFEKPQFLN 420

AAAVYLDSGKSLNTVWKIFYKLMHNEIEEF+ NIPKNSIRLLYEKFP+PAPCFEKP+FLN

Sbjct 1524 AAAVYLDSGKSLNTVWKIFYKLMHNEIEEFMRNIPKNSIRLLYEKFPIPAPCFEKPRFLN 1583

Query 421 DNKTMVMVPLIVTNKNDRKAFVGVGENKNQAKLAAAKVALRHYYLDPI 468

+N+T+VMVPLIVT KNDR F+GVGENKNQAKLAAAKVALRHYYLDP+

Sbjct 1584 ENQTIVMVPLIVTAKNDRMRFIGVGENKNQAKLAAAKVALRHYYLDPM 1631

Graphical representation


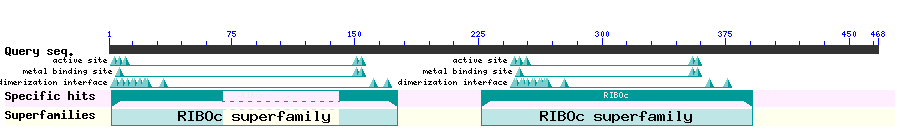


**Argonaute 2 isoform 1**

>TRINITY_DN26282_c4_g2_i1 length= 4196 nt

GTTTGATATCATAAATTAGAAATAATTACTAAAAAAAAGAAAAAAAAATCATTTATCTTTATTTTAAATTACATTGAAACGCTGTAACAATGCAATTCAGTTTTAACACTAAATAACGATTCATTAACATTGTCATGTATTTTTAAAAATAGCTTACTGTGATTCACAGACTGATGTGTCAGAGGACTTTGTATTTGATAACCTCTCATTTGGTTTTAATTTCATACAGTTGCGTCAATTTTATTCAATCTTGTCTGTTCTGGCATTACATTCCATGATTCAGGTTTTAGTTTATTTATTCTGGTGATCTTGTATTATATTCAATGATTAAAAGGTACTTTTGATCTAAATATTATTGGCCGTAGTGAAGATCATTTTATTGTACTCCAGTTTTTGTGAAACTTGAATATATCATCAAATGGGCAAGAAAGGAAAGAAAGGTGGAGGAAGAGGAGGAGGAGGAGGAGGCGGTGGACAACCTGGAGAAGCAGCTCCAGGCCCATCTAATCAACCTCAGCAACAGCAACAGCATCAGGAACAGCAAAGATTTCATGATGATCCTCAAGATTTCCCTGCACTTGGTGGTGGAGGAAGGAGAGGTGGAAGGGGAGGAGGCGGAGGTGGGCGGGGAGGAGGTGGAGGTGGAGGTAGACCTCAACCTGGAGAAGCGCCACCAGAACAATCGCAAAGGCAGCAGGAGCATTCTCAGCCACAAGATATCCCACAGCCAGGAGGAGGAGGAAGGAGAGGTGGAAGGGGTGGAGGTGGACAGGGAGGCGGTGGAGGTAGACCTCAACCTGGAGAAGCGCCACCAGAACAATCGCAAAGGCAGCAGGAGCATTCTCAGCCACGAGATGCTCCTCAAGATATCCCACAGCCAGGAGGAGGAAGAGGGCGTGGACGTGGTAGACCTCAAGCTGTTGAAGCACACCCAGGCACGTCTTATCAACCTGAACCAGCTCAAAGGCAACATCAACAGCAACAGGATTTTCCACGACCTGGAGGAGAAGGAAGAGGGCGTGGACGCGGTAGACCTCAACCTGTTGAAGCACAACCAGGTGCATATTTTCATCCTGAACAGCCACAGAGGCAACAGCAGCAACAACATCAACAACCGAGGTTTGAGGATGTTCCTCAGGATTTTTCAAGACCTGGAGGAGAAGGTGACTCAAGAAGGAGGGGAGGAAAGCAACAACAGAGATCTGGACCTCAGGAAGCTTCACTACCGACTCAACCAATGCCTTCCCAGCAGTTTCCACAGGGACAAAGGATGCAACCTCCAGGAATACCAGTAGGCGATGCTAGACCAGTGTCTCAGCAACAGCAGGCCCGCCTCCCTTTGCAAACTGGTCCCAGAGGGCAACCTACTGCATGGAGGCAGAGAGGACCATCTCCACAACAAATGTCTCAACCACAATCTAGAGAAACAACTCCAACTTCTCCGCCACTAGTATCTCCTGTAGCCAGTGTGCTTCCTATTAGACCTCAATCCTCTGACCAGCAACAAACTAGACCACAGCCTTTTCAACCACCGGCAGAACAACAAGCGGTGGCACCTCAGCCAAAATCTCCCCCAGCCGGCAGTGCTTTAGCAAAAAAAATGGAATCTTTATCTGTTGTATCTTCATTAATACCACCCAAAAGAGCAGGTCGTCCTCCAGACCCGAAAAAAGGAAGAGAAATTCTTGTTGAAACTAATCACCTTAACTTAAACATTAAGAATAAGGGTATGGTGATTCATCACTATGATGTCTCTATTAATCCTGAAAAACCTTTTCGTAATTATAGAGCTGCTGTGGAAGCGGTGCGACGGAAGTGTTTTGCCCATCGATTCCCTGCCTTTGATGGAAAAAAGAACCTGTACAGTTATCCTGAGTTGCCTCTTAGAACATCGGAACTTGTTGAAACTGTGTCAATTTATGATAATGAAAGAGATCAAGAAAAAGAAATGACGGTAACCATCAAGTATGCTACTCAAGTTAATGTCTCAATGATATGGGATTATCTGAGGTGTGGCACTTCAACTAACGCACCACAGGAAGCTATTCAAGCACTTGATGTTGTCTTAAGGCAACCAGCTGCCAACAGATTTGTAACAGTCGGAAGATCATTCTTCACCCCTCCACCTGGTAGAGCGATTGATCTTGGTTATGGCCTTGACCTTTGGTATGGATTTTTCCAGTCTGCTATTATTGGTTGGAAACCATTTCTTAACATTGATGTGGCCCATAAGGGTTTCCCGGCTGCTGACAATTGTGTTGATGCCTTAAAAAAATTTATTAGTGGGAGAGTTGATGATCCTAACTTAAGGATTCAGCACTATGAAATGGATTCCTTTGTTTCATACATAAAAGATTTAAAAGTTGCATATGAAATTCCATCAAGAGGAATTAAAAGAACGTATAAAGTAAACAATATTTCAACTTGCCCTAGAGACAACAGATTTGAAGTTGTTGACAGAGAGACAAATAATAGATCAAATACAACTGTAGAAAATTATTTTGGCACTCAGTATAATTATAGATTGAGATACCCTCACTTACCATGTCTTTCCGTTGGTTCACGTGAAAAGCCTCTTTCTCTTCCATTGGAGTTATGTAGGATCATTGAAGGTCAAGTAACTATGAAGAAAATGAATGATGCTCAGACTCGCACTATGGTGAGAGAGGCAGCTGTGGATACAGATAGAAGGAAAGATAAAATTAAAAGTTCTATTGCTAAGATAAACTTCAATGCTGACCCATGTTTAAAAGAATTCGGGCTCTCAGTGGATAGTGACTTTACTAGAGTCAAGGCCCGAATCATGAATGCCCCTACAGTGAAATATTTAGAAAACCCAGTTACTGTTAGACAAGGTGTCTGGAGAAGCGAAAGGTTTATAAAAGGGGCTGAGTTGAGCCTGTGGAGGATTGTTAATACTAATCAACGAGGTATTAGAGAACAAGATTTAAGAAACCTTGCAAATGGTTTAATAAATCATGGAAGAGATTGCGGCATGAATATTAGCAATAATTATTTGATTGCTACCCTGCTTACTCCTATTGATCTTGAACAATACTTTAAAAAATGCCTGCAAGAAAAAGTAAAACTTGTTATTGTTGTCTTACCCGATAGAGGAATAATGACATACGCATCAATTAAGAAAGTGGCTGAACTTCAAGTTGGTATTCTTACTCAGTGCCTCAAGTCAATTACAGTGCAAAAAAGACTAAATGCTGCTACATTTATTAACATACTGCAAAAGGTTAATGCAAAATTGAATGGTGTCAACCATCACATCACTGACAGTTATTGGCCAAGAATTTTCCAAAGACCTGTCATTGTTGTGGGAGCTGATGTGACACATCCTGCCCCAGACCAGATAAATGTCCCATCAATTGCTGCAGTGGCCGCTTCTCAAGACCCTCGAGCCTTTAGGTATAATATGATATGGAAGCTCCAACCACCACGGGAAGAAGTCATCAGAGATCTTCAAAGTATTATGAAAGATCAGTTACTGACATTCTATAGAAGCACCCGCTATAAACCTGAAGCTATCCTCTTTTATAGGGATGGTGTTTCAGAAGGACAATTCAAAACAATCTTAAATCAAGAGTTGATGGCCATAAGGAAGGCTTGTTCAAGTCTCAGTGTTGATTACAAACCTCCCATAACGTTTATCGTTGTACAGAAACGACATCACACCAGATTCTTTCCTCAGCAGAAGGATGCCGATGGTAGGAATAAGAATGTACCTGCTGGTACCGTGGTTGATACCGATATCACTCATCCTACTGAATTAGACTTCTATTTAGTTAGTCACGCCAGTATACAGGGAACTGCACGTCCTACTAAGTATCATTTGTTATGGGATGACTCTAGTTTATCTGAACAGAGCTTAGAAGAGATAACTTATTATCTTTGTCACCTGTTTACCAGATGCACACGCTCAGTTTCGTACCCAGCACCTACTTATTATGCTCATCTTGCTGCCTTCCGTGCTAGAGCATATACAGATACAGAGAGGTTACAGTTGGACCGTCTTCCAGAAGAGCAGATTAGAAGGAAGGTGCAGGATTCAGTTGGTCTAGAAAACCCAATGTTCTTCGTATAATAGAAAAAATATTAACCTTATACTATCTTTTCATGATATAACTTCATATTATAAAAAAAAAGTTTTTTTTTTTTTTTAATGCTTTATACACTTTTAAACGCTG

Protein: RF 2: 419 -> 4093 (1224 aa)

Comparison with Nezara viridula, PREDICTED: Argonaute 2-PC- Sequence ID: AVK59468.1

E=0.0; bits= 1516;

Query 304 ARLPLQTGPRGQPTAWRQRGPSPQQMSQPQSRETTPT-------SPPLVSPVASVLPIRP 356

AR PLQTGPRGQ T W QRGP PQQ+SQPQSRETTPT VSPV S P+R

Sbjct 121 ARFPLQTGPRGQ-TPWAQRGPPPQQVSQPQSRETTPTHQTQLPPQQQPVSPVTSAPPVRQ 179

Query 357 QSSDQQQTRPQPFQPPAEQQAVAPQPKSPPAGSALAKKMESLSV-VSSLIPPKRAGRPPD 415

Q SDQQ +RP + Q A +P+SP GSALA+KM ++ + SS IPPKR RP D

Sbjct 180 QPSDQQPSRPP--SSQPQVQQQAAKPRSPAEGSALAEKMRNIDIKSSSYIPPKRGDRPSD 237

Query 416 PKKGREILVETNHLNLNIKNKGMVIHHYDVSINPEKPFRNYRAAVEAVRRKCFAHRFPAF 475

PKKGREI+VETNHLNLNIKNK MVIHHYDVSINPEKP+RNYR AVEAVR++CF +RFPAF

Sbjct 238 PKKGREIVVETNHLNLNIKNKNMVIHHYDVSINPEKPYRNYRQAVEAVRQRCFKNRFPAF 297

Query 476 DGKKNLYSYPELPLRTSELVETVSIYDNERDQEKEMTVTIKYATQVNVSMIWDYLRCGTS 535

DGKKNLYSYPELPL+ +ELVETV+IYDNERDQEKEMTVTIKYATQVNVSMIWDYLR GTS

Sbjct 298 DGKKNLYSYPELPLQKTELVETVTIYDNERDQEKEMTVTIKYATQVNVSMIWDYLRAGTS 357

Query 536 TNAPQEAIQALDVVLRQPAANRFVTVGRSFFTPPPGRAIDLGYGLDLWYGFFQSAIIGWK 595

TN PQEAIQALD+VLRQPAANRFVTVGRSFF+PPPGR IDLGYGLDLWYGFFQSAIIGWK

Sbjct 358 TNGPQEAIQALDIVLRQPAANRFVTVGRSFFSPPPGRVIDLGYGLDLWYGFFQSAIIGWK 417

Query 596 PFLNIDVAHKGFPAADNCVDALKKFISGRVDDPNLRIQHYEMDSFVSYIKDLKVAYEIPS 655

PFLNIDVAHKGFPAADNCV AL KF+ G + DP +++ YE +SFV+YIKDLKV YEIPS

Sbjct 418 PFLNIDVAHKGFPAADNCVVALGKFVQGDLGDPRFQMRPYERESFVAYIKDLKVVYEIPS 477

Query 656 RGIKRTYKVNNISTCPRDNRFEVVDRETNNRSNTTVENYFGTQYNYRLRYPHLPCLSVGS 715

+ +KRTYKV NI+TCPRDNRFE+ D+ETNN+S TTVE YF +QYN RL+YPHLPCL+VGS

Sbjct 478 KSVKRTYKVTNITTCPRDNRFEITDKETNNKSETTVERYFVSQYNIRLKYPHLPCLTVGS 537

Query 716 REKPLSLPLELCRIIEGQVTMKKMNDAQTRTMVREAAVDTDRRKDKIKSSIAKINFNADP 775

REKPL+LP+ELC I+ GQVTMKKMNDAQTRTMV+EAAVDT+RRK+KI SI +INFN+DP

Sbjct 538 REKPLALPMELCTIVAGQVTMKKMNDAQTRTMVKEAAVDTERRKEKIVKSIRQINFNSDP 597

Query 776 CLKEFGLSVDSDFTRVKARIMNAPTVKYLENPVTVRQGVWRSERFIKGAELSLWRIVNTN 835

CLKEFGLSVD FT+VKARIMNAPT+ Y E PV VRQGVWR+E+FI+GA+L WRIV TN

Sbjct 598 CLKEFGLSVDDQFTKVKARIMNAPTLDYRERPVPVRQGVWRNEKFIQGADLVQWRIVTTN 657

Query 836 QRGIREQDLRNLANGLINHGRDCGMNISNNYLIATLLTPIDLEQYFKKCLQEKVKLVIVV 895

Q IRE DLR LA+GLINHG +CGMNISNNY I + TP +LEQYF +CL+E+VKLVIVV

Sbjct 658 QM-IREPDLRKLASGLINHGMECGMNISNNYKIVSKNTPRELEQYFSQCLKEEVKLVIVV 716

Query 896 LPDRGIMTYASIKKVAELQVGILTQCLKSITVQKRLNAATFINILQKVNAKLNGVNHHIT 955

LPDRG MTYASIKK+AE+QVGILTQCLK++TVQ+RLNAATF+NILQKVNAKLNG+NHHIT

Sbjct 717 LPDRG-MTYASIKKIAEIQVGILTQCLKTVTVQRRLNAATFVNILQKVNAKLNGINHHIT 775

Query 956 DSYWPRIFQRPVIVVGADVTHPAPDQINVPSIAAVAASQDPRAFRYNMIWKLQPPREEVI 1015

SYWP+ FQ+PVIVVGADVTHPAPDQINVPSIAAVAAS DP+AFRYNMIWKLQPPREEVI

Sbjct 776 HSYWPKFFQKPVIVVGADVTHPAPDQINVPSIAAVAASHDPKAFRYNMIWKLQPPREEVI 835

Query 1016 RDLQSIMKDQLLTFYRSTRYKPEAILFYRDGVSEGQFKTILNQELMAIRKACSSLSVDYK 1075

RDL+SIMK+QLL FYRSTRYKP+AILFYRDGVSEGQFK ILNQEL AIR+ACSSLS DY

Sbjct 836 RDLESIMKEQLLAFYRSTRYKPQAILFYRDGVSEGQFKMILNQELQAIRRACSSLSSDYT 895

Query 1076 PPITFIVVQKRHHTRFFPQQKDADGRNKNVPAGTVVDTDITHPTELDFYLVSHASIQGTA 1135

PPITFIVVQKRHHTRFFP++KDADGRN NVPAGTVVDT+ITHPTELDFYLVSHASIQGTA

Sbjct 896 PPITFIVVQKRHHTRFFPEKKDADGRNMNVPAGTVVDTEITHPTELDFYLVSHASIQGTA 955

Query 1136 RPTKYHLLWDDSSLSEQSLEEITYYLCHLFTRCTRSVSYPAPTYYAHLAAFRARAYTDTE 1195

RPTKYHLLWDDS+LSEQ+LEEITYYLCHLFTRCTRSVSYPAPTYYAHLAAFRARAYTD +

Sbjct 956 RPTKYHLLWDDSNLSEQNLEEITYYLCHLFTRCTRSVSYPAPTYYAHLAAFRARAYTDAD 1015

Query 1196 RLQLDRLPEEQIRRKVQDSVGLENPMFFV 1224

RLQL++L EEQ+RR V+DSV NPMFFV

Sbjct 1016 RLQLNQLQEEQVRRTVKDSVCKNNPMFFV 1044

Graphical representation


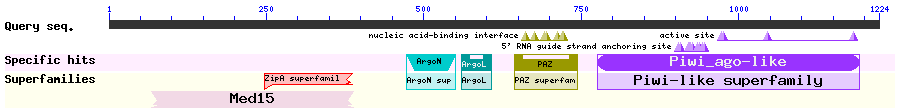


**Argounaute 2 isoform 2**

> TRINITY_DN26282_c4_g2_i2 length= 4271 nt

GTTTGATATCATAAATTAGAAATAATTACTAAAAAAAAGAAAAAAAAATCATTTATCTTTATTTTAAATTACATTGAAACGCTGTAACAATGCAATTCAGTTTTAACACTAAATAACGATTCATTAACATTGTCATGTATTTTTAAAAATAGCTTACTGTGATTCACAGACTGATGTGTCAGAGGACTTTGTATTTGATAACCTCTCATTTGGTTTTAATTTCATACAGTTGCGTCAATTTTATTCAATCTTGTCTGTTCTGGCATTACATTCCATGATTCAGGTTTTAGTTTATTTATTCTGGTGATCTTGTATTATATTCAATGATTAAAAGGTACTTTTGATCTAAATATTATTGGCCGTAGTGAAGATCATTTTATTGTACTCCAGTTTTTGTGAAACTTGAATATATCATCAAATGGGCAAGAAAGGAAAGAAAGGTGGAGGAAGAGGAGGAGGAGGAGGAGGCGGTGGACAACCTGGAGAAGCAGCTCCAGGCCCATCTAATCAACCTCAGCAACAGCAACAGCATCAGGAACAGCAAAGATTTCATGATGATCCTCAAGATTTCCCTGCACTTGGTGGTGGAGGAAGGAGAGGTGGAAGGGGAGGAGGCGGAGGTGGGCGGGGAGGAGGTGGAGGTGGAGGTAGACCTCAACCTGGAGAAGCGCCACCAGAACAATCGCAAAGGCAGCAGGAGCATTCTCAGCCACAAGATATCCCACAGCCAGGAGGAGGAGGAAGGAGAGGTGGAAGGGGTGGAGGTGGACAGGGAGGCGGTGGAGGTAGACCTCAACCTGGAGAAGCGCCACCAGAACAATCGCAAAGGCAGCAGGAGCATTCTCAGCCACGAGATGCTCCTCAAGATATCCCACAGCCAGGAGGAGGAAGAGGGCGTGGACGTGGTAGACCTCAAGCTGTTGAAGCACACCCAGGCACGTCTTATCAACCTGAACCAGCTCAAAGGCAACATCAACAGCAACAGGATTTTCCACGACCTGGAGGAGAAGGAAGAGGGCGTGGACGCGGTAGACCTCAACCTGTTGAAGCACAACCAGGTGCATATTTTCATCCTGAACAGCCACAGAGGCAACAGCAGCAACAACATCAACAACCGAGGTTTGAGGATGTTCCTCAGGATTTTTCAAGACCTGGAGGAGAAGGTGACTCAAGAAGGAGGGGAGGAAAGCAACAACAGAGATCTGGACCTCAGGAAGCTTCACTACCGACTCAACCAATGCCTTCCCAGCAGTTTCCACAGGGACAAAGGATGCAACCTCCAGGAATACCAGTAGGCGATGCTAGACCAGTGTCTCAGCAACAGCAGGCCCGCCTCCCTTTGCAAACTGGTCCCAGAGGGCAACCTACTGCATGGAGGCAGAGAGGACCATCTCCACAACAAATGTCTCAACCACAATCTAGAGAAACAACTCCAACTTCTCCGCCACTAGTATCTCCTGTAGCCAGTGTGCTTCCTATTAGACCTCAATCCTCTGACCAGCAACAAACTAGACCACAGCCTTTTCAACCACCGGCAGAACAACAAGCGGTGGCACCTCAGCCAAAATCTCCCCCAGCCGGCAGTGCTTTAGCAAAAAAAATGGAATCTTTATCTGTTGTATCTTCATTAATACCACCCAAAAGAGCAGGTCGTCCTCCAGACCCGAAAAAAGGAAGAGAAATTCTTGTTGAAACTAATCACCTTAACTTAAACATTAAGAATAAGGGTATGGTGATTCATCACTATGATGTCTCTATTAATCCTGAAAAACCTTTTCGTAATTATAGAGCTGCTGTGGAAGCGGTGCGACGGAAGTGTTTTGCCCATCGATTCCCTGCCTTTGATGGAAAAAAGAACCTGTACAGTTATCCTGAGTTGCCTCTTAGAACATCGGAACTTGTTGAAACTGTGTCAATTTATGATAATGAAAGAGATCAAGAAAAAGAAATGACGGTAACCATCAAGTATGCTACTCAAGTTAATGTCTCAATGATATGGGATTATCTGAGGTGTGGCACTTCAACTAACGCACCACAGGAAGCTATTCAAGCACTTGATGTTGTCTTAAGGCAACCAGCTGCCAACAGATTTGTAACAGTCGGAAGATCATTCTTCACCCCTCCACCTGGTAGAGCGATTGATCTTGGTTATGGCCTTGACCTTTGGTATGGATTTTTCCAGTCTGCTATTATTGGTTGGAAACCATTTCTTAACATTGATGTGGCCCATAAGGGTTTCCCGGCTGCTGACAATTGTGTTGATGCCTTAAAAAAATTTATTAGTGGGAGAGTTGATGATCCTAACTTAAGGATTCAGCACTATGAAATGGATTCCTTTGTTTCATACATAAAAGATTTAAAAGTTGCATATGAAATTCCATCAAGAGGAATTAAAAGAACGTATAAAGTAAACAATATTTCAACTTGCCCTAGAGACAACAGAAGAAATATTCTTTCTTTACAAGGTTCTAAAATTGTTTGAAAAGACTAAAGATTGAAACACTCTGTGAAATGCAGATTTGAAGTTGTTGACAGAGAGACAAATAATAGATCAAATACAACTGTAGAAAATTATTTTGGCACTCAGTATAATTATAGATTGAGATACCCTCACTTACCATGTCTTTCCGTTGGTTCACGTGAAAAGCCTCTTTCTCTTCCATTGGAGTTATGTAGGATCATTGAAGGTCAAGTAACTATGAAGAAAATGAATGATGCTCAGACTCGCACTATGGTGAGAGAGGCAGCTGTGGATACAGATAGAAGGAAAGATAAAATTAAAAGTTCTATTGCTAAGATAAACTTCAATGCTGACCCATGTTTAAAAGAATTCGGGCTCTCAGTGGATAGTGACTTTACTAGAGTCAAGGCCCGAATCATGAATGCCCCTACAGTGAAATATTTAGAAAACCCAGTTACTGTTAGACAAGGTGTCTGGAGAAGCGAAAGGTTTATAAAAGGGGCTGAGTTGAGCCTGTGGAGGATTGTTAATACTAATCAACGAGGTATTAGAGAACAAGATTTAAGAAACCTTGCAAATGGTTTAATAAATCATGGAAGAGATTGCGGCATGAATATTAGCAATAATTATTTGATTGCTACCCTGCTTACTCCTATTGATCTTGAACAATACTTTAAAAAATGCCTGCAAGAAAAAGTAAAACTTGTTATTGTTGTCTTACCCGATAGAGGAATAATGACATACGCATCAATTAAGAAAGTGGCTGAACTTCAAGTTGGTATTCTTACTCAGTGCCTCAAGTCAATTACAGTGCAAAAAAGACTAAATGCTGCTACATTTATTAACATACTGCAAAAGGTTAATGCAAAATTGAATGGTGTCAACCATCACATCACTGACAGTTATTGGCCAAGAATTTTCCAAAGACCTGTCATTGTTGTGGGAGCTGATGTGACACATCCTGCCCCAGACCAGATAAATGTCCCATCAATTGCTGCAGTGGCCGCTTCTCAAGACCCTCGAGCCTTTAGGTATAATATGATATGGAAGCTCCAACCACCACGGGAAGAAGTCATCAGAGATCTTCAAAGTATTATGAAAGATCAGTTACTGACATTCTATAGAAGCACCCGCTATAAACCTGAAGCTATCCTCTTTTATAGGGATGGTGTTTCAGAAGGACAATTCAAAACAATCTTAAATCAAGAGTTGATGGCCATAAGGAAGGCTTGTTCAAGTCTCAGTGTTGATTACAAACCTCCCATAACGTTTATCGTTGTACAGAAACGACATCACACCAGATTCTTTCCTCAGCAGAAGGATGCCGATGGTAGGAATAAGAATGTACCTGCTGGTACCGTGGTTGATACCGATATCACTCATCCTACTGAATTAGACTTCTATTTAGTTAGTCACGCCAGTATACAGGGAACTGCACGTCCTACTAAGTATCATTTGTTATGGGATGACTCTAGTTTATCTGAACAGAGCTTAGAAGAGATAACTTATTATCTTTGTCACCTGTTTACCAGATGCACACGCTCAGTTTCGTACCCAGCACCTACTTATTATGCTCATCTTGCTGCCTTCCGTGCTAGAGCATATACAGATACAGAGAGGTTACAGTTGGACCGTCTTCCAGAAGAGCAGATTAGAAGGAAGGTGCAGGATTCAGTTGGTCTAGAAAACCCAATGTTCTTCGTATAATAGAAAAAATATTAACCTTATACTATCTTTTCATGATATAACTTCATATTATAAAAAAAAAGTTTTTTTTTTTTTTTAATGCTTTATACACTTTTAAACGCTG

Protein: RF 2: 419 -> 4093 (1224 aa)

Comparison with *Nezara viridula*, PREDICTED: Argonaute 2-PC- Sequence ID: AVK59468.1

E=0.0; bits= 565;

Query 304 ARLPLQTGPRGQPTAWRQRGPSPQQMSQPQSRETTPT-------SPPLVSPVASVLPIRP 356

AR PLQTGPRGQ T W QRGP PQQ+SQPQSRETTPT VSPV S P+R

Sbjct 121 ARFPLQTGPRGQ-TPWAQRGPPPQQVSQPQSRETTPTHQTQLPPQQQPVSPVTSAPPVRQ 179

Query 357 QSSDQQQTRPQPFQPPAEQQAVAPQPKSPPAGSALAKKMESLSV-VSSLIPPKRAGRPPD 415

Q SDQQ +RP + Q A +P+SP GSALA+KM ++ + SS IPPKR RP D

Sbjct 180 QPSDQQPSRPP--SSQPQVQQQAAKPRSPAEGSALAEKMRNIDIKSSSYIPPKRGDRPSD 237

Query 416 PKKGREILVETNHLNLNIKNKGMVIHHYDVSINPEKPFRNYRAAVEAVRRKCFAHRFPAF 475

PKKGREI+VETNHLNLNIKNK MVIHHYDVSINPEKP+RNYR AVEAVR++CF +RFPAF

Sbjct 238 PKKGREIVVETNHLNLNIKNKNMVIHHYDVSINPEKPYRNYRQAVEAVRQRCFKNRFPAF 297

Query 476 DGKKNLYSYPELPLRTSELVETVSIYDNERDQEKEMTVTIKYATQVNVSMIWDYLRCGTS 535

DGKKNLYSYPELPL+ +ELVETV+IYDNERDQEKEMTVTIKYATQVNVSMIWDYLR GTS

Sbjct 298 DGKKNLYSYPELPLQKTELVETVTIYDNERDQEKEMTVTIKYATQVNVSMIWDYLRAGTS 357

Query 536 TNAPQEAIQALDVVLRQPAANRFVTVGRSFFTPPPGRAIDLGYGLDLWYGFFQSAIIGWK 595

TN PQEAIQALD+VLRQPAANRFVTVGRSFF+PPPGR IDLGYGLDLWYGFFQSAIIGWK

Sbjct 358 TNGPQEAIQALDIVLRQPAANRFVTVGRSFFSPPPGRVIDLGYGLDLWYGFFQSAIIGWK 417

Query 596 PFLNIDVAHKGFPAADNCVDALKKFISGRVDDPNLRIQHYEMDSFVSYIKDLKVAYEIPS 655

PFLNIDVAHKGFPAADNCV AL KF+ G + DP +++ YE +SFV+YIKDLKV YEIPS

Sbjct 418 PFLNIDVAHKGFPAADNCVVALGKFVQGDLGDPRFQMRPYERESFVAYIKDLKVVYEIPS 477

Query 656 RGIKRTYKVNNISTCPRDNRFEVVDRETNNRSNTTVENYFGTQYNYRLRYPHLPCLSVGS 715

+ +KRTYKV NI+TCPRDNRFE+ D+ETNN+S TTVE YF +QYN RL+YPHLPCL+VGS

Sbjct 478 KSVKRTYKVTNITTCPRDNRFEITDKETNNKSETTVERYFVSQYNIRLKYPHLPCLTVGS 537

Query 716 REKPLSLPLELCRIIEGQVTMKKMNDAQTRTMVREAAVDTDRRKDKIKSSIAKINFNADP 775

REKPL+LP+ELC I+ GQVTMKKMNDAQTRTMV+EAAVDT+RRK+KI SI +INFN+DP

Sbjct 538 REKPLALPMELCTIVAGQVTMKKMNDAQTRTMVKEAAVDTERRKEKIVKSIRQINFNSDP 597

Query 776 CLKEFGLSVDSDFTRVKARIMNAPTVKYLENPVTVRQGVWRSERFIKGAELSLWRIVNTN 835

CLKEFGLSVD FT+VKARIMNAPT+ Y E PV VRQGVWR+E+FI+GA+L WRIV TN

Sbjct 598 CLKEFGLSVDDQFTKVKARIMNAPTLDYRERPVPVRQGVWRNEKFIQGADLVQWRIVTTN 657

Query 836 QRGIREQDLRNLANGLINHGRDCGMNISNNYLIATLLTPIDLEQYFKKCLQEKVKLVIVV 895

Q IRE DLR LA+GLINHG +CGMNISNNY I + TP +LEQYF +CL+E+VKLVIVV

Sbjct 658 QM-IREPDLRKLASGLINHGMECGMNISNNYKIVSKNTPRELEQYFSQCLKEEVKLVIVV 716

Query 896 LPDRGIMTYASIKKVAELQVGILTQCLKSITVQKRLNAATFINILQKVNAKLNGVNHHIT 955

LPDRG MTYASIKK+AE+QVGILTQCLK++TVQ+RLNAATF+NILQKVNAKLNG+NHHIT

Sbjct 717 LPDRG-MTYASIKKIAEIQVGILTQCLKTVTVQRRLNAATFVNILQKVNAKLNGINHHIT 775

Query 956 DSYWPRIFQRPVIVVGADVTHPAPDQINVPSIAAVAASQDPRAFRYNMIWKLQPPREEVI 1015

SYWP+ FQ+PVIVVGADVTHPAPDQINVPSIAAVAAS DP+AFRYNMIWKLQPPREEVI

Sbjct 776 HSYWPKFFQKPVIVVGADVTHPAPDQINVPSIAAVAASHDPKAFRYNMIWKLQPPREEVI 835

Query 1016 RDLQSIMKDQLLTFYRSTRYKPEAILFYRDGVSEGQFKTILNQELMAIRKACSSLSVDYK 1075

RDL+SIMK+QLL FYRSTRYKP+AILFYRDGVSEGQFK ILNQEL AIR+ACSSLS DY

Sbjct 836 RDLESIMKEQLLAFYRSTRYKPQAILFYRDGVSEGQFKMILNQELQAIRRACSSLSSDYT 895

Query 1076 PPITFIVVQKRHHTRFFPQQKDADGRNKNVPAGTVVDTDITHPTELDFYLVSHASIQGTA 1135

PPITFIVVQKRHHTRFFP++KDADGRN NVPAGTVVDT+ITHPTELDFYLVSHASIQGTA

Sbjct 896 PPITFIVVQKRHHTRFFPEKKDADGRNMNVPAGTVVDTEITHPTELDFYLVSHASIQGTA 955

Query 1136 RPTKYHLLWDDSSLSEQSLEEITYYLCHLFTRCTRSVSYPAPTYYAHLAAFRARAYTDTE 1195

RPTKYHLLWDDS+LSEQ+LEEITYYLCHLFTRCTRSVSYPAPTYYAHLAAFRARAYTD +

Sbjct 956 RPTKYHLLWDDSNLSEQNLEEITYYLCHLFTRCTRSVSYPAPTYYAHLAAFRARAYTDAD 1015

Query 1196 RLQLDRLPEEQIRRKVQDSVGLENPMFFV 1224

RLQL++L EEQ+RR V+DSV NPMFFV

Sbjct 1016 RLQLNQLQEEQVRRTVKDSVCKNNPMFFV 1044

Graphical representation


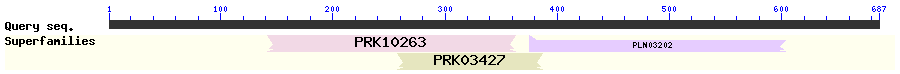


**R2D2**

>TRINITY_DN21334_c0_g1_i1 length= 1278 nt

AGATAAACATACCTTCCATCATGGCTATACCTTTTGGAAGGTTATAACAAGCCAACTATGATCAATAGTATTTCTAACTTCAAATATAGTGTGAATTAGCACTTGCTTTCTTAAAATTAGTGCGGAATAGATTTTAGGTGTTAAATTGAAGTGACAAGCATTGTGATTATGATTTTTTATTATTTATTTAACCCGCTCAATAGAGATTTTTTTTTTAGTTTTTAATTGCTATGGATGGAAAAACACCTGTAACGTTACTTCATGAGTATTTAATCAAAGGTGGAGAAGTTCCTGATTACAAATTGGTATATAATGGAGTAGGAACACATGATCCTTTATTCCAATATGAAGTCAGTGCTAAAGGTATGTCTGCTGTAGGGAAAGGCAAATCAAAAAAAGAAGCCAAACATGATGCAGCAAGGTCCTTACTTCTTAAGTTGAAAGATGAACATGCTTTGGAAGAGGCGGTAGAAGTTGTTTCACCTTATGAACATTCTCTTAAAGAAAATGCTGTTGGCCAACTTCAGGATTTCTGTTCACAGCACAACACAGCTTTCCCAAAATATGATTTAATAAGAGATGAAGGTTTAGCACATGCGAAAATTTTTGGTATACGCTGTAGGGTTTCTTCTTTTTTCACTGAAGCTGAAGCCCGAACAAAGAAACAAGCCAAGCAGCAAGCTTCACATTTAATGCTGTTGAAGCTGGAAAAATGTCTTAGTGAGGGGAACTTTATTGCAATGCCTGATACAGAGAAACAAGATTCCAGTGGCTTAGTTGAAAAGGCTTGTGATGTGACCAAGGAAGCATATCAAAAAGTTGTGCAAGAAAGGACGAATGATAAAGACAAAATATCATGCTATAGACTAGGAACTTCAATGAGTGATTTTTCTAATCAGTTTTTAGACAATGTTTTACCTTTATCTGAGACTCTTAAGTCAATTGCTGATAAAGATGATTCTTTCTTTGAAACCCTTCAAGATCCAGAAGAATTGCTTTCCACAATATTAACACAATTAGATTACCATTACAATTATGAATTTTTAGAAACTAGGACGGAATTAGAGTATTTATGTAGTTTACAGATTAGAGAATTGAATTGTGCTAACTTTATAGGGTTTTCAACAACAAATACTATTGCTAAAAAAAATGCTAACATCCAAGCGCTTCAGTTTTTAGCAACTATGTCTAAAATGTGAAAACAAATTTTGGTAACTACTATATTAAATTCATTATTTTAATTTAAGAGGAGTTTCATCCATTTAATTTAATTTAACA

Protein: RF -3: -231 -> -1199 (322 aa)

Comparison *Halyomorpha halys,* PREDICTED: Interferon-inducible double-stranded RNA-dependent protein kinase activator A-like isoform X1 - Sequence ID: XP_014288218.1

E= 0.0; bits= 559

Query 304 ARLPLQTGPRGQPTAWRQRGPSPQQMSQPQSRETTPT-------SPPLVSPVASVLPIRP 356

AR PLQTGPRGQ T W QRGP PQQ+SQPQSRETTPT VSPV S P+R

Sbjct 121 ARFPLQTGPRGQ-TPWAQRGPPPQQVSQPQSRETTPTHQTQLPPQQQPVSPVTSAPPVRQ 179

Query 357 QSSDQQQTRPQPFQPPAEQQAVAPQPKSPPAGSALAKKMESLSV-VSSLIPPKRAGRPPD 415

Q SDQQ +RP + Q A +P+SP GSALA+KM ++ + SS IPPKR RP D

Sbjct 180 QPSDQQPSRPP--SSQPQVQQQAAKPRSPAEGSALAEKMRNIDIKSSSYIPPKRGDRPSD 237

Query 416 PKKGREILVETNHLNLNIKNKGMVIHHYDVSINPEKPFRNYRAAVEAVRRKCFAHRFPAF 475

PKKGREI+VETNHLNLNIKNK MVIHHYDVSINPEKP+RNYR AVEAVR++CF +RFPAF

Sbjct 238 PKKGREIVVETNHLNLNIKNKNMVIHHYDVSINPEKPYRNYRQAVEAVRQRCFKNRFPAF 297

Query 476 DGKKNLYSYPELPLRTSELVETVSIYDNERDQEKEMTVTIKYATQVNVSMIWDYLRCGTS 535

DGKKNLYSYPELPL+ +ELVETV+IYDNERDQEKEMTVTIKYATQVNVSMIWDYLR GTS

Sbjct 298 DGKKNLYSYPELPLQKTELVETVTIYDNERDQEKEMTVTIKYATQVNVSMIWDYLRAGTS 357

Query 536 TNAPQEAIQALDVVLRQPAANRFVTVGRSFFTPPPGRAIDLGYGLDLWYGFFQSAIIGWK 595

TN PQEAIQALD+VLRQPAANRFVTVGRSFF+PPPGR IDLGYGLDLWYGFFQSAIIGWK

Sbjct 358 TNGPQEAIQALDIVLRQPAANRFVTVGRSFFSPPPGRVIDLGYGLDLWYGFFQSAIIGWK 417

Query 596 PFLNIDVAHKGFPAADNCVDALKKFISGRVDDPNLRIQHYEMDSFVSYIKDLKVAYEIPS 655

PFLNIDVAHKGFPAADNCV AL KF+ G + DP +++ YE +SFV+YIKDLKV YEIPS

Sbjct 418 PFLNIDVAHKGFPAADNCVVALGKFVQGDLGDPRFQMRPYERESFVAYIKDLKVVYEIPS 477

Query 656 RGIKRTYKVNNISTCPRDNRRNI 678

+ +KRTYKV NI+TCPRDNR I

Sbjct 478 KSVKRTYKVTNITTCPRDNRFEI 500

Graphical representation


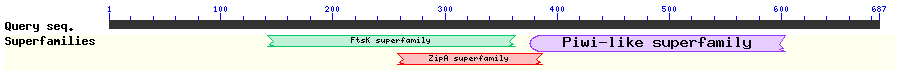


**Argonaute 3**

>TRINITY_DN27163_c0_g1_i1 length= 2854 nt

CTTTGTCTGACAATGATTCTGATGCTTCTTTGTGAACATTTTCACCAATTAAATAAGCAAGTCGGTGAGCATATTGACAAGGTGCAGGGACTCGGATCGTTCCTGGCCAGTTATAATACAGATGAGTAAGCATATATGTGAATCGCTGAATTTGATCAGGAGACATAGTTGTTGTATTCCTCACGACTATGTAGTGAGTTGGCGAAACAGTTCCCTGATTAACGTGTTGAGATACCAAAAAGAAATCATGAAGGTAACGACGAGTAATCGTATGATCCATAACAGTTCCTGGCAACGGATTAGAAAAGCTCTTATCACCACGCTCAACTCCAAATAGCCTAGTATTAATCCTTTTCTGCACAACTACAAAAAGGATCTTAGGCTTGTATTCGGGTGAGATTCTCTGACAGGCAGTCATTATTTGAGGCAGTTCATAATCTTCACAAAGCCGCAGTTGACCGTCAGATACTCCATCTCTAAATATTGTAATTTGCTCAGGAAAATTTCCATTAACCTCTCTATATTTTTGCAACGAGGAAATCATACTGACTTGAAGGCCATCTACAAATTCGAGACCAGTAGCCTGACTGTAAATTTTGGAATACCAACGAGTCAGTGGTTGGTTCAATGAGCTGACAAGGGCACCAACGCTGTTCGATTTTTTTTTGGGATCGTGATAGGTGTCCAGTCCACAAACCATAGTGAATTTCAAAGGTACATTAACTGCCCACAATGTTCCACCAAGTTTACAATTCATCTGTAGAGCAATTTTTAAGGTAATAGCTCTTGAATTCCCAGCTCTTCTTACCGTCCTTGACAGTATTACCTGTGAAGGAATGGGTTGTTCAACGCAGCACAGCTTCTTAACAGCGCTGTACTTGTCGGTTCTGGCCAACGGAAAGATGATGACGACTATCTGAATGTTATCCCTGATCGACGACCTTAAAGACCTGATGTAATTGTCAGTCCTGTCATCTGGAATCGTAACAATGTTCGGCTTCATAATTTTGATACCCATTTGAGGTCCCATCTTTATCATCTGTTCTGTAAAGGATGTAACCATATCCAAATCTCTTCTGGTACAGACCACCGTCCAAGTGACAAAATTAATAGCAGATATTGCTTTGTTTTTATTGGAAGCAGCAGACCAGCTAGCATCTCGACTGCCAGGAACCGTAACTCCACCACCGAATATTATGTCAACAGGGGGAATATTACGACCCGGAAGAACCATATAAGCATCATCCAGTGAAATCCCCCAGTCAGAGAGAACTTTGGTAGCTTCGGCGGAACCTTTAATATTATTTGTGAACTGGTTAATTGCGTAGTGCCTTTGGTTGGGCGAGATACTGATGCACTGAGTGATATCTTTCATAACCCTAGCATCAGCCCTCATCGTATCAGTTATTCCTGTTAGGAAACAGAGTTCAGGGACTAAAGAAACTAATTGCCTTTCTTCCTTTCCTCTAACTCTTGTTTTTACTCTGCTTTTCAGAAGCGGTTGGTTATAATCTGTAATGTTTATGTTATAGTGAATCCTGTAGTAGTCGACGTATTTAATTTTTTCACCATTACTTTTTTCGAAGGAATCATTAGGGCATTCATCGAATGACAAATCATCAATTCTATAAACCTTGTTGTTATACCTTGTAAGTACAGACTGACCTAATATTAACTGGATAAATTCGTCTTTCCAGTGCTGGTGATTTGAATTTTGGAGATCATCCAACAGTTGAAGAACAGTTTGATTTCTCAGAACTCTGTGGGAGGCATCACAACAGAGCATGATGCCTCCTTCATATTCATCAATAGCAGTTATGTAGCCCGGCCATACTTCCAACTTATGCAATGGAATAGGCATAGCTGATTTTGGATCATAAAAATTTTTTCCATGAAGCGCCAAGCCTAGAATAACCATAATCCTTCTAAATAGAATGTTGTAGAGGTGCACAGCTTCGGGATCGCCAAATTTCCTTTGTTTCGTAAATGTAATTTTGACCGTAACAACACTATTATCGTGCGATACAGTTGTTTTTATCGTGGTAACTCTATCAGGTAATTTAAATGGCAAGTAAAGAACCATTCCATCAAATAATCTGGTCTCTCCGATTACATTCTTGACAGTATTCAATATCGAAAATTTTAGACTCCTCGATTCTATCGGGGGATCATATTGAACATCGTACTGAAAAAGACCTTTACCTTTCTCCACTTTGAGCCTGACGTAGTTTACGCCAATGGATACTTCCCTTCCATATGTTCCCTTCATTATTACAGGTTCCTTTTCTGCTGAAGACGTGGATTCGGCCAAGGAAGTTCTAGACAACTGTCTAGAGATTTTTTCAACTGCAGGGCTGTCTTCCCTAGTAGCCAATGAGGCTAACAAAGCTCTTCCTCTTCCAGATTTCGGAGGACCTCCCATATCCTCCTTCACTTCTTCACATTTCCTCAATTCACTAGCTATCAAGGCACTTCTGCCTCTTGGTTTTTTTATTTCACCAAATGATGTTTGAGCTACCGTATCCTGCCCATAAAATTTTGGATTGTGTTCCTGCGCGAATGGTGAAAATGAAGAGGATGACGGACGAGATGATTCAGGTTGAGGGAACGATTGAGCATGAGGTAATGACTGCGGCTGGGATTCAAGAAGCTTTCTTAAAGCTTCTCCTCTCCCAAGACGTCTACCATCCATTTTATTAGATAAATCAATTTATGCAGTAAAGGTAGACTATATGATGACGATGACAACCAAAAATTTATCCAAAACAATGATAAATAATTAAAAGTGAAAGAAAAGAACGAATATCTGGGCGAAACGACAAGCTTAAACAAAGACCTACACTTTTTGGTCAAG

Protein: RF -1: -2692 -> >2 (896 aa)

Comparison with *Halyomorpha halys*, PREDICTED: protein argonaute-3

E= 0.0; bits= 1595

Query 1 MDGRRLGRGEALRKLLESQPQSLPHAQSFPQPE-SSRPSSSSFSPFAQEHNPKFYGQDTV 59

MDGRRLGRGEALRKLLESQP L HAQS+ Q E SS SSSS S AQE +P Y QD

Sbjct 1 MDGRRLGRGEALRKLLESQPH-LSHAQSYSQHEPSSHRSSSSLSSAAQEQDPYTYRQDVA 59

Query 60 AQTSFGE-IKKPRGRSALI-ASELRKCEEVKEDMGGPPKSGRGRALLASLATREDSPAVE 117

A TS + +KKP GRSAL+ E+RK EE+KE++ P SGRGRALLASL RE SP+VE

Sbjct 60 AHTSESQGVKKPMGRSALLLGGEMRKSEELKEEI--PKPSGRGRALLASLVAREGSPSVE 117

Query 118 KISRQLSRTSLAESTSSAEKEPVIMKGTYGREVSIGVNYVRLKVEKGKGLFQYDVQYDPP 177

KI+R LSRTSLA ST EKEPV+MKG++GRE+S+GVNYV+LKVEKGKGLFQYDVQYDPP

Sbjct 118 KITRHLSRTSLAGSTVE-EKEPVVMKGSFGREISVGVNYVKLKVEKGKGLFQYDVQYDPP 176

Query 178 IESRSLKFSILNTVKNVIGETRLFDGMVLYLPFKLPDRVTTIKTTVSHDNSVVTVKITFT 237

+ESRSLKF ILN+VK+VIG+T+LFDGMVLYLPFKL +VT +KTTV+HDNSVVTVKITFT

Sbjct 177 VESRSLKFGILNSVKDVIGDTKLFDGMVLYLPFKLNQKVTILKTTVAHDNSVVTVKITFT 236

Query 238 KQRKFGDPEAVHLYNILFRRIMVILGLALHGKNFYDPKSAMPIPLHKLEVWPGYITAIDE 297

K+RK GDPEA+HLYN+LFRRIMVILGLALHGKNF+DPKSAMPIPLHKLEVWPGYITAIDE

Sbjct 237 KERKLGDPEAIHLYNVLFRRIMVILGLALHGKNFFDPKSAMPIPLHKLEVWPGYITAIDE 296

Query 298 YEGGIMLCCDASHRVLRNQTVLQLLDDLQNSNHQHWKDEFIQLILGQSVLTRYNNKVYRI 357

YEGGIMLCCDASHRVLRNQTVLQL+DDLQNSN +HW+DEF+QLILGQSVLT+YNNKVYRI

Sbjct 297 YEGGIMLCCDASHRVLRNQTVLQLMDDLQNSNQKHWRDEFVQLILGQSVLTKYNNKVYRI 356

Query 358 DDLSFDECPNDSFEKSNGEKIKYVDYYRIHYNINITDYNQPLLKSRVKTRVRGKEERQLV 417

DD+SF+ECPND FEKSNGEK++YVDYYR+ YN+ +TD NQPLLKSRVK RVRGKEE+QLV

Sbjct 357 DDVSFEECPNDCFEKSNGEKVRYVDYYRLQYNLRLTDSNQPLLKSRVKMRVRGKEEKQLV 416

Query 418 SLVPELCFLTGITDTMRADARVMKDITQCISISPNQRHYAINQFTNNIKGSAEATKVLSD 477

SLVPELCFLTGITDTMRADARVMKDITQCISISPNQRHYAINQFTNNI+ S EATKVLSD

Sbjct 417 SLVPELCFLTGITDTMRADARVMKDITQCISISPNQRHYAINQFTNNIRASPEATKVLSD 476

Query 478 WGISLDDAYMVLPGRNIPPVDIIFGGGVTVPGSRDASWSAASNKNKAISAINFVTWTVVC 537

WGISLDDAYMVLPGRNIPP+D++FGGGVT+PGSR+A+WS ASNKNKAIS +NFVTW+V+C

Sbjct 477 WGISLDDAYMVLPGRNIPPIDVMFGGGVTIPGSREANWSGASNKNKAISVVNFVTWSVIC 536

Query 538 TRRDLDMVTSFTEQMIKMGPQMGIKIMKPNIVTIPDDRTDNYIRSLRSSIRDNIQIVVII 597

TRRD+DMVTSF EQMIK+GPQMGIKI KP+IV+IPDDRTDNYIRSLRSSI++NIQIVV+I

Sbjct 537 TRRDMDMVTSFIEQMIKIGPQMGIKINKPDIVSIPDDRTDNYIRSLRSSIKNNIQIVVVI 596

Query 598 FPLARTDKYSAVKKLCCVEQPIPSQVILSRTVRRAGNSRAITLKIALQMNCKLGGTLWAV 657

FPLARTDKYSAVKKLCCVE+PIPSQVIL+RTVRRAG S+AITLKIALQMNCKLGGTLWAV

Sbjct 597 FPLARTDKYSAVKKLCCVEEPIPSQVILARTVRRAGTSKAITLKIALQMNCKLGGTLWAV 656

Query 658 NVPLKFTMVCGLDTYHDPKKKSNSVGALVSSLNQPLTRWYSKIYSQATGLEFVDGLQVSM 717

NVPLK+TMVCGLDTYHDPK++++SVGALVSSLNQPLTRWYSKIYSQ+TGLEFV GLQVSM

Sbjct 657 NVPLKYTMVCGLDTYHDPKRRADSVGALVSSLNQPLTRWYSKIYSQSTGLEFVAGLQVSM 716

Query 718 ISSLQKYREVNGNFPEQITIFRDGVSDGQLRLCEDYELPQIMTACQRISPEYKPKILFVV 777

I+SLQKYREVNG++PEQITIFRDGVSDGQLRLCEDYELPQIM ACQRISPEY PKILFVV

Sbjct 717 IASLQKYREVNGSYPEQITIFRDGVSDGQLRLCEDYELPQIMNACQRISPEYMPKILFVV 776

Query 778 VQKRINTRLFGVERGDKSFSNPLPGTVMDHTITRRYLHDFFLVSQHVNQGTVSPTHYIVV 837

VQKRINTRLFGVER DKSFSNP+PGTVMDHTITRRYLHDFFLVSQHV+QGTVSPTHYIVV

Sbjct 777 VQKRINTRLFGVER-DKSFSNPMPGTVMDHTITRRYLHDFFLVSQHVSQGTVSPTHYIVV 835

Query 838 RNTTTMSPDQIQRFTYMLTHLYYNWPGTIRVPAPCQYAHRLAYLIGENVHKEASESLSDK 897

RNTT+MSPDQIQRFTYMLTHLYYNWPGTIRVPAPCQYAHRLAYLIGEN+HKEA+ESLSDK

Sbjct 836 RNTTSMSPDQIQRFTYMLTHLYYNWPGTIRVPAPCQYAHRLAYLIGENIHKEAAESLSDK 895

Graphical representation


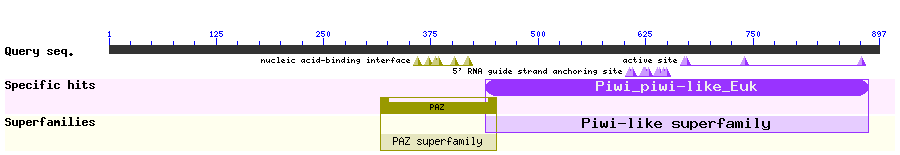


**Aubergine**

>TRINITY_DN680_c0_g1_i1 length= 2588 nt

GTTGTTCCTGTTTTATATTGTTTTAATTTTGGAAATATGGAAGAAGGGAGAGCTCGAGGCAGAGCCAGGGGTAGGGCAAGACAGGATCCTAAGAGAGTGGAGATGACTCAAAGTGTGATTGAAGCTAGAGGAGCGAGGGCCAAACCACAGCAGGCCGTTCCTACAGTTGCTTCTGTCTCAGGAGACATGAAGCAGTTATCGGTAACTCCTAGAAGACCTGGAGGAAGAAAAATGATTGATAAATTGGATTATGTTTCAATAAGATCTAGGCCTAAAGAACTTGTCACCAAACAAGGGTCCACTGGCCAAAAGGTCAAACTTCAAGCCAATTATTTTAAATTAGAAACTCACCCTGATTGGGCCTTATATCAATATAGAGTTGATTTTTCACCTGAAGAAGAGAGAACTGCTGTGAAAAAAGCATTACTTAAACCTCACAAAGATATTTTTAATTGTGCATATTTGTTTGATGGTACAGTGTTGTATGTTTGTAATAGGTTGAAAACTGACCCAATTGAACTTTTCTCACTCCGGGATACTGACCGGAAGAAGATAAGGATTACTATAAAATTTGTTGGTGATGTTGTTATGGGAGATTACCAATATCTTCAAGTTTTTAATATAATTATGCGCAAGTGTCTTGATAATCTTAAATTACAAATGGTGGGCCGTAATTTCTTTGATGCCAAAGCCAGAATTGAAATACGTGAGTACAGGATGGAGCTTTGGCCTGGTTATCTAACATCGATCAGACAATGTGAAGATCATATTCTAATGAATGCTGAAATAACGTATAAGGTAATGCGTTGTGAGACTGTTCTTGATTTAATTGTTAAATGTAGAAACTCCGAAGATTGGGAGAAAACCTTTGAGAATGCAATCATAGGTACTATTGTTTTAACTGATTACAATAATCGCACATATCGTATTGATGATGTGAACTTTAATTGTACCCCTATGTCTAAGTTTAAAATTAAGAATGGTGAATCATCCACTTATTGTGAATACTATAAGACTCGTTATGGTATTAAAATCAGGGAACCGAATCAACCCATGTTAGTTTCTAAAGCGAAACCGAGGGAAGTTCGAGCTGGAATGACTGAAATAATTTATTTGGTTCCAGAGCTGTGTCGCTTGACAGGTCTTACTGATGATATGAGAACAAACTTTCAACTAATGAGGGCTCTTGCTGAACATACTAGAGTTACACCAAAGCTTAGAATTGATAAATTATTAAAATTCAATCACAGATTAAAAGAAAGTAAAGAAATCACCAAAGATTTAGATTCATGGAACATGAAGCTTGCTGAGTCTTTAGTAACATTCGATGGACGAGTCCTTCCTATGGAGAAGATCTACTTTGGGGAAAATAGAGCTGTGACTGCTGGGAACGATGCGGACTGGACCAGAAGCATGAGAGATCACTCCATGTTGACCAGTGGAAATTTCAAATCTTGGTCCATTGTTTACTTAGGAAGAACAAAGAGTGAAGTTAATAGCTTCATTCACACTCTTTCTAAGGCAGCTGAGAGTTTAAATTTTCGAGTCCCTACACCAAGAATGATTGAAGTCATAAATGACAGGTGTGGTAGCTTTGTTGAAGCATTAGATTCTGTGATTTCCATGCACAACCCTCAATTAATACTTTGTATTGTTCCAAACAGTAGAGCTGATAGATATGCAGCCATTAAGAAGAAATGTTGTGTAGATAGAGCAGTCCCCACCCAAGTTGTAGTTGCAAAACATTTCAATTCTAAAAATCTAATGGCTATTTCTACAAAAATTGCAATTCAGATTAATTGCAAGCTTGGTGGTATCCCTTGGACCATTGCAAATCCACTGAAAGGTTTAATGGTGGTAGGATATGATGTCTGTCATGATGCTTCTCGAAAAGACATGTCATATGGAGCAATGGTGGCTTCTTTGAATCCATCTCTTTCAAGATACTATTCCTCTGTAACTCCCCACTCTCATGGGGAGGAGCTATCTAATGACCTGGCCATGAATATTTTCAAAGCTATTATTCGGTATAAGGAAAATAATGAAGGAAACATACCTTCAATGATAATACTCTATCGAGACGGTGTTGGGGAGGGTCAAATACCATTTGTTTATAATCACGAAGTAAGAATTGTCAAAGAACGACTTTCTGAGATCTACACAGATAAACCTCCTAAGTTAGGGTTTGTAATTGTTACTAAGAGACTTAATACCAGGTTATTCCTCAATGGCAGTAATGCTACCCCTGGTACGGTTGCTGATGATTGCATCACTTCTCCTGATCGCTACGACTTCTTCCTTGTATCACAATCTGTACGCCAAGGTACTGTAAGCCCTACCTCATATAATGTTATAGATGATTCCACTGGTCTAGATGCAGATAAAATGCAACGGCTTGCTTACAAAATGACTCACCTGTATTACAATTGGAGTGGTACTGTAAGAGTTCCAGCTCAGTGCCAGTATGCTCATAAGTTGGCTTTTCTTGTTAGCCAGTGTTTACATAGAGCCCCAAATCCTGATCTTGATGATTTGTTGTATTTTTTGTAATTTGTTCAAGGTAAATTTGGAACTAATACTATGATGTG

Protein: RF -1: -37 -> -2550 (837 aa)

Comparison with *Halyomorpha halys*, PREDICTED: protein aubergine-like - Sequence ID: XP_014270559.1

E= 0.0; bits= 1676

Query 1 MEEGRARGRARGRARQDPKRVEMTQSVIEARGARAKPQQAVPTVASVSGDMKQLSVTPRR 60

MEEGRARGRARGR+RQDPK+ E+T +VIEARG R+KPQ A PTVA+V GDMKQL+VTPRR

Sbjct 1 MEEGRARGRARGRSRQDPKKTEVTHNVIEARGVRSKPQHASPTVATVVGDMKQLAVTPRR 60

Query 61 PGGRKMIDKLDYVSIRSRPKELVTKQGSTGQKVKLQANYFKLETHPDWALYQYRVDFSPE 120

PGGRKM+DKLD+VSIRSRPKEL+TKQG+TGQKVKLQANYFKLETHPDWALYQYRVDFSPE

Sbjct 61 PGGRKMMDKLDFVSIRSRPKELLTKQGTTGQKVKLQANYFKLETHPDWALYQYRVDFSPE 120

Query 121 EERTAVKKALLKPHKDIFNCAYLFDGTVLYVCNRLKTDPIELFSLRDTDRKKIRITIKFV 180

EERT+VKKALLKPHKDIFNCAYLFDGTVLYVCNRLKTDPIELFSLRDTDRKKIRITIKFV

Sbjct 121 EERTSVKKALLKPHKDIFNCAYLFDGTVLYVCNRLKTDPIELFSLRDTDRKKIRITIKFV 180

Query 181 GDVVMGDYQYLQVFNIIMRKCLDNLKLQMVGRNFFDAKARIEIREYRMELWPGYLTSIRQ 240

GDVVMGDYQYLQVFNIIMRKCLDNLKLQMVGRNFFDA+ARIEIREYRMELWPGYLTSIRQ

Sbjct 181 GDVVMGDYQYLQVFNIIMRKCLDNLKLQMVGRNFFDARARIEIREYRMELWPGYLTSIRQ 240

Query 241 CEDHILMNAEITYKVMRCETVLDLIVKCRNSEDWEKTFENAIIGTIVLTDYNNRTYRIDD 300

CEDHILMNAEITYKVMRCETVLDLIVKCR+SEDWEKTFENAIIGTI+LTDYNNRTYRIDD

Sbjct 241 CEDHILMNAEITYKVMRCETVLDLIVKCRSSEDWEKTFENAIIGTIILTDYNNRTYRIDD 300

Query 301 VNFNCTPMSKFKIKNGESSTYCEYYKTRYGIKIREPNQPMLVSKAKPREVRAGMTEIIYL 360

VNFNCTPMSKFKIKNGESSTYCEYYKTRYGIKI+EPNQPMLVSKAKPREVRAGMTEIIYL

Sbjct 301 VNFNCTPMSKFKIKNGESSTYCEYYKTRYGIKIKEPNQPMLVSKAKPREVRAGMTEIIYL 360

Query 361 VPELCRLTGLTDDMRTNFQLMRALAEHTRVTPKLRIDKLLKFNHRLKESKEITKDLDSWN 420

VPELCRLTGLTDDMRTNFQLMRALAEHTRVTPKLRI+KLLKFNHRLKESK+ITKDLDSWN

Sbjct 361 VPELCRLTGLTDDMRTNFQLMRALAEHTRVTPKLRIEKLLKFNHRLKESKDITKDLDSWN 420

Query 421 MKLAESLVTFDGRVLPMEKIYFGENRAVTAGNDADWTRSMRDHSMLTSGNFKSWSIVYLG 480

MKLAESL+TFDGRVLPMEKIYFGENR VTAGNDADWTRSMRDHSML+SGNFKSWSIVYLG

Sbjct 421 MKLAESLITFDGRVLPMEKIYFGENRVVTAGNDADWTRSMRDHSMLSSGNFKSWSIVYLG 480

Query 481 RTKSEVNSFIHTLSKAAESLNFRVPTPRMIEVINDRCGSFVEALDSVISMHNPQLILCIV 540

RTKSEVNSFIHTL+KAAESLNFRVPTPR+ EV+NDRCGSFVEALDSVISMHNPQLILCIV

Sbjct 481 RTKSEVNSFIHTLAKAAESLNFRVPTPRLTEVVNDRCGSFVEALDSVISMHNPQLILCIV 540

Query 541 PNSRADRYAAIKKKCCVDRAVPTQVVVAKHFNSKNLMAISTKIAIQINCKLGGIPWTIAN 600

PNSRADRYAAIKKKCCVDRAVPTQVVVAKHFNSKNLMAISTKIAIQINCKLGGIPWTIAN

Sbjct 541 PNSRADRYAAIKKKCCVDRAVPTQVVVAKHFNSKNLMAISTKIAIQINCKLGGIPWTIAN 600

Query 601 PLKGLMVVGYDVCHDASRKDMSYGAMVASLNPSLSRYYSSVTPHSHGEELSNDLAMNIFK 660

PLKGLMVVGYDVCHDASRKDMSYGAMVASLNPSLSRYYSSVTPHSHGEELSNDLAMNIFK

Sbjct 601 PLKGLMVVGYDVCHDASRKDMSYGAMVASLNPSLSRYYSSVTPHSHGEELSNDLAMNIFK 660

Query 661 AIIRYKENNEGNIPSMIILYRDGVGEGQIPFVYNHEVRIVKERLSEIYTDKPPKLGFVIV 720

AIIRYKENNEGNIPSMIILYRDGVGEGQIPFVYNHEVRIVKERLSEIY DKPPKLGFVIV

Sbjct 661 AIIRYKENNEGNIPSMIILYRDGVGEGQIPFVYNHEVRIVKERLSEIYKDKPPKLGFVIV 720

Query 721 TKRLNTRLFLNGSNATPGTVADDCITSPDRYDFFLVSQSVRQGTVSPTSYNVIDDSTGLD 780

TKRLNTRLFLNGSNATPGTVADDCITSPDRYDFFLVSQSVRQGTVSPTSYNVIDDSTGLD

Sbjct 721 TKRLNTRLFLNGSNATPGTVADDCITSPDRYDFFLVSQSVRQGTVSPTSYNVIDDSTGLD 780

Query 781 ADKMQRLAYKMTHLYYNWSGTVRVPAQCQYAHKLAFLVSQCLHRAPNPDLDDLLYFL 837

ADKMQRLAYKMTHLYYNWSGTVRVPAQCQYAHKLAFLVSQCLHRAPNPDLDDLLYFL

Sbjct 781 ADKMQRLAYKMTHLYYNWSGTVRVPAQCQYAHKLAFLVSQCLHRAPNPDLDDLLYFL 837

Graphical representation


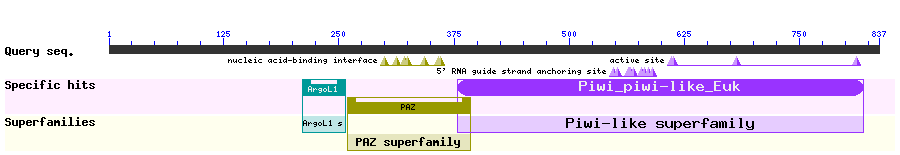


**Piwi**

>TRINITY_DN26875_c9_g1_i2 length= 3175 nt

CCCAAAACATTTTCATAACTGATTTTTAAAACCAATTAAACAGAAAGCAAAATAGAAAAAAAGGTACATATGAATCTTACAAACTATTCTTCCTTACTCATTGGAAGGAATAAAAAAAATTAAAGGAAATATAGTAAATGACTAAGTTCTGCACTAGGAGGTATGTGGATCACCTGCCCAACTAAAAATGCTAGCTTATGAGCATATTGGACCTGGCATGGAACCCTCACGGTAGAAGTGCAATTATAATAGAGATGGCACATTTTGTAGGTCATCCTCTGAATATGATCAGGTTGCAGTCTACAATCATCTACAATTACATTGTAGTTGGTAGGAGTCACAGTACCCTGTCGTACTGATTGTGATACCAAGAAAAAATCATACTTCTCAGGTTGGGTAATGACATCATCAACTATTGTACCAGGGGGAGGGTTGAAGTAGTCTGTTTCACCTTTCCTAACTAAAATCCTTGTCTGAATACGCTTAGATACAATGACAACTGTCAATTGTGGAGTGTTATGAGGAAACATTTGAGAAAGCCTCGACTTAATCATCCGGATTTCATGATCCAAAACATATCTAATATTTGATTCACCAACGCCATCTCTATACATTACTATTTTGGGAGGTAAATAATTGTTGATTTCAATAAATCGCAAAACTGCATGGCCTATGTGGGTAGCAATATCATTTGATAATTCCTCTCCTAAACTGTGTCTGCTGACTGAAGAGAAAAATTTTGAGAAATGGTAATCAAGAGAGGCTACCATCGCACCTACAGATTTCCCTTTCATTCTGGTATCATGGCACACATCAAATCCGACTATCATTGTAGCAAATGTTATATTTTTTTTGGTACCATCTGTCAGGAATACGTTATCCTTAAATGGGATTGGGGTGAACCATGGGGTCCCACCCAGTTTACAGTTCATCTGTATAACAATTTTAGTGCAGACTGATAGCAAACTTTTATGAGTTAATGTTTTAGTAGCTATCACTTGAGAAGCAACTGGCCTGTCACAGTAACACTTCTTTTTAATGGCAGCATACAGGTCAGACCTAGTTGGATTTATGACACACATAACCAGCTGGATTCCACCAGAATTCATACATTTTTCAATTACTCCAAGATAGTTGGCAATCTTATCAGAATCAATAATTATCACTTCAGGTTTTCTCATTTCAAAGCTTAAACTCCTTGCTGCTTTAATTAGTTCAGCAACAAAATGGTCAAGAGGGTTACCATTAAACCTTCTATCTTGACATGCATGTTTCGAAGCTATTAATATCCATTTTTCTAGTCTAACAGATCTCAACATGCCAACATTACGCAAAGTACGAGACCAATCTCCATCTTGCGACTGTATTTTAGCCTCGTTGGCCACATACAAAATCTCACCATTAAAAACTCTTCCTGGGACAAGTTCCAAGCTTGGAGCAAATTTGAGGTTCCACTTAGTAAGGTGTTCTCTAACCTCTTCTTTTCCATGCAATTTCCTCATAAATTGATAGTAGCTTTCAGCACGCTTAGAGGGTGCTACACGAGTATGATCGGCAACTGCCTTCATTAAATTGAAATTGCTCCTCATTTCATCAGAAAGACCTGTCATTTGGCAAAGTTCAGGAATTAGTACAATGTTCTGATCCCTTCCTCCTCTTATATCTCTGGCTTTCGGTCTTGATATAAGCAAAGGCTGCCTACGGTTGTGTATTACAAGTCCATATTTCTTAGAATAGTATTCTATATAAGATATATCAGAATTATCCTTCCTAAATGTATCCATCGGTGTCATATCAAATGCCACATCATCAACTCGATAAGGCTTGTTGTTATAATATGTAAAGACCACAGAACCTAATATCAAGTCCAAAAAGGATTTCTGCCAATTTGGACCATATCTCTCACGACAACCTTCAAGGTATTCTAAAACACTATCGGTACGTAACACTTTATGTGAAACATCGATTCCCATCATAATGTGATTCTCAAATTGGAGAATCGAAGTAGTAAAACCCGGCCATATTTCAAGTTTAAAATTTGGCATTTTCAAACTCTTACTAGGATCAAAGTATTCACGCCCAATAAGATTAAACTTCATAGCAGACATACAGTTACGAAGAATAATATTATACACTTGAATATATTGAAAATCGCCAACAGCTATATCATTGGTAAATCTGATAGCTACATTAATTACTGAACCATCACGATCACTTTTAATTGTCAGATTGAAGGGATCTTTATGAAATCTCTGGTTGGTGAACATCATCGATCCATCAAAAATAAATCCCTGTTTAATTTCACTCCTAACAGAGTGTATTATCCCCCTACGAACTCGTGTTCTTTCTTCATCAGGAGAAAAATCAACTCTGTGTTGATACATAGTCCAATCAGTTGTTTTGACTAATCTGAAATGATTAGTCACTAATTCGACCTTCTTTCCTAAAGTTCCACATTTAGCACTCAAATTTTCAGGCCTTGTTCTAACTGGAGGTAACACTTCCTTCCGACCTCTCACAGCACCTCTACGCAAAGCAGTTCCAGGTTTAGGTTCTCCTACAGATATTCCTCTAAACGCTCCAGATACTGCATCTATATCGGGATCAAGTCCACATGCTCCCTCCTCTTGTGGCTCTTGGTGCTGTAGAACTTTTGGCGGTTGTACTTGTTTAGGAAGCTGCCCCCTAGCTCGAGCTGAAATTCTCGGCTGCACTGGCTGCTGTTGCAGAGGTGATGCAGAGGGTAACTGATATTGATTTGATGGACCCTGTGAGGGTTGTTGAGATGAAGCAGGTTGAGACCAAGCAGACCGTTGAGGCGGAGCAGCTTGTTGTGGTGGAGCAGCTTGTTGTGATGGAGCAGCTTGTTGGGGCAGAGCAGCTTGTTGGGGCTGAGTAGCTTGTTGACGTACCTGTGGGGAAACCTGTTGTTGAAGCTGCTGCTGAGAAGCTGCAGGAACCCCACGGGCTCTTCCCCAGGCTCTACCCCTAGCCCTACCCTTAGGTTGTTGCCCTTCTGACATTTTTTACAATTTCAGACCTCCTCAATCGTTTGTAGCAAGACTGAATCAGATCACGAAAAATAAATTACTGGATAAGGAAAATAACAATCATTAAAATAAAAACAAATGAATTCAGACCAGACAACATGTAAGACTTCTGACCTCGTGAGGAG

Protein: RF -3: -3023 -> -120 (967 aa)

Comparison with *Halyomorpha halys*, PREDICTED: protein aubergine-like isoform X3 - Sequence ID: XP_014275927.1

E= 0.0; bits= 1172

Query 83 PSQGPSNQYQLPSASPLQQQPVQPRISARARGQLPKQVQP-------------------- 122

P S+ Q PS P QQQ R + RARG +PKQVQP

Sbjct 122 PLSPTSSSEQFPSL-PQQQQKQPSRSAGRARGIVPKQVQPGASGVQQMLQQQQPPQSQSQ 180

Query 123 PKVLQHQEPQEEGACGLDPDIDAVSGAFRGISVGEPKPGTALRRGAVRGRKEVL-PPVRT 181

P QE EEGAC +DP+I +VSGAF+G+ VGE KPG LRRG +RGR V+ PP +T

Sbjct 181 PTPQPQQERHEEGACAIDPEIKSVSGAFQGLKVGESKPGN-LRRGMMRGRLTVVTPPPKT 239

Query 182 RPENLSAKCGTLGKKVELVTNHFRLVKTTDWTMYQHRVDFSPDEERTRVRRGIIHSVRSE 241

RP+NLS K G G V+LVTN++RL K+T+W++YQ+RVD P+EERT+VR+G+I +

Sbjct 240 RPDNLSVKLGVSGTPVKLVTNYYRLTKSTNWSLYQYRVDI-PNEERTKVRKGLISCHKDR 298

Query 242 IKQGFIFDGSMMFTNQRFHKDPFNLTIK--SDRDGSV-INVAIRFTNDIAVGDFQYIQVY 298

I ++FDG+M+F Q+ H D N I+ S R V I + IRFTN++ VGD+QYIQ++

Sbjct 299 IGDAYLFDGTMLFVIQKLHPDR-NAVIELCSKRHDDVKITMTIRFTNEMLVGDYQYIQLF 357

Query 299 NIILRNCMSAMKFNLIGREYFDPSKSLKMPNFKLEIWPGFTTSILQFENHIMMGIDVSHK 358

NIILRNCM MK+ L+GREYFD KS+KMPN+ LEIWPG++TSILQFENHIMMGIDVSHK

Sbjct 358 NIILRNCMDDMKYQLVGREYFDARKSIKMPNYHLEIWPGYSTSILQFENHIMMGIDVSHK 417

Query 359 VLRTDSVLEYLEGCRERYGPNWQKSFLDLILGSVVFTYYNNKPYRVDDVAFDMTPMDTFR 418

VLR+D+V E+L CRER+G +++K F D +LG+VVFT+YNNKPYRVDDVAFD TP+ TF

Sbjct 418 VLRSDNVWEFLRACRERHGTDYEKHFQDTMLGAVVFTFYNNKPYRVDDVAFDETPISTFT 477

Query 419 -KDNSDISYIEYYSKKYGLVIHNRRQPLLISRPKARDIRGGRDQNIVLIPELCQMTGLSD 477

K + SY +YY KYG+ I + QPLL+SRPK RDIR GR NI+LIPELCQMTG++D

Sbjct 478 TKKGQNTSYFDYYHTKYGVEIKDLNQPLLVSRPKPRDIRAGRSNNIILIPELCQMTGIND 537

Query 478 EMRSNFNLMKAVADHTRVAPSKRAESYYQFMRKLHGKEEVREHLTKWNLKFAPSLELVPG 537

EMR+NF LMKAVA HTRV P KR +SYY FM+ L E+ +E L WN++F+ LE V G

Sbjct 538 EMRNNFTLMKAVAAHTRVEPEKRIQSYYNFMKDLGSCEKAQEKLEVWNVQFSQELETVQG 597

Query 538 RVFNGEILYVANEAKIQSQDGDWSRTLRNVGMLRSVRLEKWILIASKHACQDRRFNGNPL 597

R+ + E L+V I+ + GDWSR LR MLR V+L+ WI+IASK A D+R GNPL

Sbjct 598 RILHPEDLHVGGNKMIRPESGDWSRPLRAASMLRIVKLKNWIVIASKQATYDKRVRGNPL 657

Query 598 DHFVAELIKAARSLSFEMRKPEVIIIDSDKIANYLGVIEKCMNSGGIQLVMCVINPTRSD 657

D+F+++L KAA +L F+ +PEVIIID DKI+NYLG I+K M++ +QLVMC+IN +R+D

Sbjct 658 DNFLSDLYKAAGTLKFKFDEPEVIIIDQDKISNYLGAIDKAMSASKLQLVMCIINYSRND 717

Query 658 LYAAIKKKCYCDRPVASQVIATKTLTHKSLLSVCTKIVIQMNCKLGGTPWFTPIPFKDNV 717

LYAAIKKKC CDRP+ SQVIATKTL HK+LLSVCTKI IQ+NCKLGG+PWFTP+PFK+N+

Sbjct 718 LYAAIKKKCLCDRPIPSQVIATKTLGHKNLLSVCTKIAIQINCKLGGSPWFTPVPFKENI 777

Query 718 FLTDGTK-KNITFATMIVGFDVCHDTRMKGKSVGAMVASLDYHFSKFFSSVSRHSLGEEL 776

DGT +N+ A MIVGFDVCHDTR+KG+SVGAMVASLDY FS+F+SSVSRH+LGEEL

Sbjct 778 VRNDGTVLENMIHAIMIVGFDVCHDTRLKGQSVGAMVASLDYMFSQFYSSVSRHNLGEEL 837

Query 777 SNDIATHIGHAVLRFIEINNYLPPKIVMYRDGVGESNIRYVLDHEIRMIKSRLSQMFPHN 836

SNDIA HI AV+RF E+N YLPPKIV++RDGVGESNI YV +HEI+ IK RL +MFP+

Sbjct 838 SNDIANHISCAVMRFQELNQYLPPKIVVFRDGVGESNINYVKEHEIKRIKERLLEMFPNR 897

Query 837 TPQLTVVIVSKRIQTRILVRKGETDYFNPPPGTIVDDVITQPEKYDFFLVSQSVRQGTVT 896

TP+LTV+IVSKRIQ R + + Y NP PGT+VDDVITQPEKYDFFLVSQSVRQGTVT

Sbjct 898 TPRLTVIIVSKRIQARFFMEDRKGKYLNPLPGTVVDDVITQPEKYDFFLVSQSVRQGTVT 957

Query 897 PTNYNVIVDDCRLQPDHIQRMTYKMCHLYYNCTSTVRVPCQVQYAHKLAFLVGQVIHIPP 956

PTNYNVI D+C ++PDH+QR+ YKMCHLYYNCTSTVRVPCQVQYAHKLAFLVGQVIHIPP

Sbjct 958 PTNYNVIFDECNMKPDHVQRLAYKMCHLYYNCTSTVRVPCQVQYAHKLAFLVGQVIHIPP 1017

Query 957 SAELSHLLYFL 967

+ L HLLYFL

Sbjct 1018 NPVLDHLLYFL 1028

Graphical representation


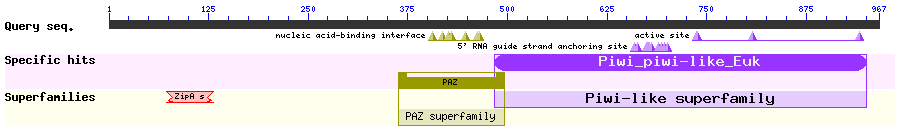


**Zucchini**

>TRINITY_DN27351_c2_g5_i2 length= 1198 nt

GGTTGCATCAGCTTTAAATGCTGTTTCCGTGTCATGCAGCCGGTGCTGTCTCTTCTTGCTGCTTCTGCATGGTTTATAACTGTGATATTTTCAGTGTTTTCTTTGTCAGTATCCTCCCTGTTATCGGCATCTTTATAAAAAGTCGCAATAATTTGGTCATCAGTAAGCATACCATAGCCTCCGTCGTTGTCAGCTTCTAAACAAAAAACTAAATTTTCCTGAACTTATCCAGGTTACCCAAAGAACCAACATAACGGGGTCCAACTAAACGAGGCTGTACTGTATTTTTTAAAAATTAGGAAACAAAATTTATAAAAATAGTTTTATTAATCTAATAAAAATGTATAAATACAAAATATTTAACATGTACATCTAACATTTTTTAAACGTTAGTCATTTGGTGCTGTGGTGCTTGAGCTTTTATGATTCCATCCAAGGGAGAATAATTGCGAGGTGAAAAATCTTCCCAAAGAGCATTGAAAGCATCCCCAAAGGAATTGACCAGCTCTGGTTGCGTTGTTATCATAACATTTTCCCAATTACAGAACGCTCCAGTCATTGTGAAATTCATACTGCCATTAATCAGTACCTCATCATCAACAATAACAAACTTGTGGTGCATAATGAATGGAGACATTCTCTGTCTTGTTGGAATATCTGCTGCCCTGAAAGTGTTTATCAACGATTGAGCTGAAAAAGACATGTCACTATCAGCTATAACTCGCACTTTGATCCCTCGCTGGTGTGCCTTAATAATACAATCTCCAAACAGTTTAGCGGTGATTATATAGATACAAACATCCAGACTCGTTTTAGCATTATTAATGAATCCCAGAATATAACTCAAACGTCCATAATTGCAGTCCAAATTGCCACAATCAAATTCCCTCTTCATGTGACCATCACACTGTTCACTGCTTACACCAAAGGTCAACAATCGGTTTTTATTTAATATGTCGACCTGGTTCTCTTTCTTTCTCTTAATTATTTTTTTCCACATGAATTGACTCAATTTGTAACTGATGATGCTTAAAACAGATACAGTACCGAATGCAGAAATTCCCAAGACCCAAAGTCTACCTTTAGTGTAACTCATTTGGATTAAAATATATGAGAAACATTGACTTCAACTAACTAAATCCATAATCAGTAATTCGTTAGGTCTTTTGCTCAAGTCAAAATAAGGCGGAAGAAGGTT

Protein: RF -1: -1096 -> -383 (237 aa)

Comparison with *Halyomorpha halys*, PREDICTED: mitochondrial cardiolipin hydrolase - Sequence ID: XP_014288409.1

E= 1e-152; bits= 432

Query 1 MSYTKGRLWVLGISAFGTVSVLSIISYKLSQFMWKKIIKRKKENQVDILNKNRLLTFGVS 60

MS TKG LW+LG+S FGTV V+ I +YK +Q+M K+I K KK+NQ+DILNKNRLLTFGVS

Sbjct 1 MSDTKGSLWLLGLSTFGTVCVIGIFNYKFNQWMRKQINKIKKDNQLDILNKNRLLTFGVS 60

Query 61 SEQCDGHMKREFDCGNLDCNYGRLSYILGFINNAKTSLDVCIYIITAKLFGDCIIKAHQR 120

SEQCDGHMKREF+CGNLDCNYGRLSYILGFINN + SLDVCIYIITAKLFGDCIIKAHQR

Sbjct 61 SEQCDGHMKREFNCGNLDCNYGRLSYILGFINNCEKSLDVCIYIITAKLFGDCIIKAHQR 120

Query 121 GIKVRVIADSDMSFSAQSLINTFRAADIPTRQRMSPFIMHHKFVIVDDEVLINGSMNFTM 180

G+KVRVIADSDMSFSAQSLINTFRAADIPTRQRMSPFIMHHKFVIVD+EVLINGSMNFTM

Sbjct 121 GVKVRVIADSDMSFSAQSLINTFRAADIPTRQRMSPFIMHHKFVIVDNEVLINGSMNFTM 180

Query 181 TGAFCNWENVMITTQPELVNSFGDAFNALWEDFSPRNYSPLDGIIKAQAPQHQMTNV 237

TGAFCNWENVMIT+QP+LVNSF DAFN+LWEDFSP NYSPLDGII+AQAPQHQ+ NV

Sbjct 181 TGAFCNWENVMITSQPQLVNSFEDAFNSLWEDFSPHNYSPLDGIIRAQAPQHQVANV 237

Graphical representation


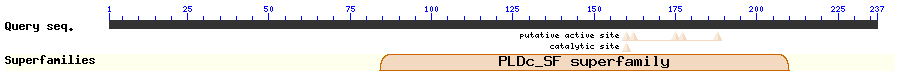


**Data S3:** Sequences of *E. heros* RISC-associated auxiliary factors.

**Tudor SN**

> TRINITY_DN27987_c0_g1_i5 length= 4170 nt

CTCCTCATAACTTTGATCATAGCCAATTACTTTCAGTTGTATTTATTAGTTAAGACTGATTATCTATTTGTTTTTCTATTACTTATTTTAAATGTTAATCAGTTGATTGTGCGGAAGAAGATGATGCAAAGTATTGCTTCAGTCAAAATGATGTTTCTAAAGAATTTCCTGTCAATCATTATCTAGCTGGCTTATGGTACTTGAAAATAGCGATCAGTTGTTCTGATCTTGATTCTACTATTGGCTTTTCACAACCAAAACGTAAAAGAAAACCTTTCAAAGCAATGTTATGTAATGAATGTGGTGACAACACATCTGATTTTCATTGCAAGCAGTGTGAAGCACCTTACTGTCAACAATGTTTCAAACTGGTCCATTCATCTGCAAAAGCATTCAGAAATCATGTCAGGATGTCTTTATTGGATTCCTATTTATGCCTCGATGCTGATTTGTCTATGATGTGCTTGACCCATAGCAAATACAAACTTGAATTTTTTTGTCTTCCTTGTCATACTGCAGTTTGCTCCCATTGCTTGGTTGAATCGCACAATGGTCATGTAGTTGATAAGATTGAAAATGTGGATATTAATTTTGAAGAAGAATTCATTCAGCGTTATATGGACGTATCGAATATTGTTGCAAGAATGAAGTCTTCTAAAAAGACAGCTGAGAATATTTTACAGCATGATAAAGATATTTGTGAGATAAAAAAGACTGATGGAGAAATCACTGATCATTTCATACTACTCCATGGTAAACTTTCATTGATTGAGCACAAACTTCGTAAAAAATTATCATCAGCAAACACTAGGAAACATTTAGAGGAGATTCTTAAACAAGTGACTGGATACATCGAGGATGCAGAGACAATTTTGGCGTCTACAAGATCTTTATTTACTGATGGATCAGCTGTTGGTCCCAAATGTTTAGCAGTCATAGACAATTTGAAGTCTCGTCAACAACTACCATGCTATTTAATTGAAAATGCATTTTGGGAAGAGCCTCGATTTTGTGCTGACAAAGAAATTATTAAGACTTTGGAAAACCATTGCCATTTGGAATTGGTTGAAGAACCTAAGTACCAATTGGTCACAAAAGAAAATATACCTTCTGGCTATGCGATATCCCCGACTAAGGATCTTTCAAGAGATGAAGTGCTGAAGTTACTTAAAAAACCATACCTGATTGTTGATTCATCATCTGTTTCACATTCGGATGGGACACTAAGCCCAGCACTTTCTCAAACGAGTGAAAAATCATTCTCATCGCAGACTCGTGATCATTCGTCACAATCTCTTTTTTATGCAGATGAAATCGCTGTTGGGTGTACGCTCAGTGTTCGTATTAGTCATCTTATTTCTCCCTCGCACTTCTATGTTCAGAGAGAGATTGCTCAGAAGAAACTACTTGAATTTCAAGCATCAATCAGGCCTTCACAAGTTCAACTTTGTAAACCACCATATAATCCAGACAAAGGTAAACTTTATTTGACACTTTATGAGGCTGACAGCAAGTGGTACAGAGCTAGAATCACTAAAGTAATCGATAATAAGAAATTTGAAGTATTTTATGTTGATTTTGGAAATTCTGAAATTGTTGACAAGTCAAAAATTAGAACTATTCCTAAATCATCTTTGAACATGCCTTGTTTGGCTTATCGATGTCAACTATCAGATTGTGTACCTAGATATGGAGATGACTGGGATGCACGTGCCATTTCACTAATGACTGAAATAATGGATGATGATTTTGTGAGTATAACAGTTGTCGACAAAACAAGCATCTCTTATTTAGTAGATCTGCATAAAGTCACTGAAAATTCTTTCATTGATCTTCGGCAGTCATTGGTATTTCATGAATTAGCTTCCATGACAAAAATGATTAGTCAAACAACAGATATTAGTGTGCAACAGAGTCTGTCAACACCTCCTCAGCATCATTATACTGATGGGAGTGTCTTCGAAGTTCATGTTTCGAATGTTGAATCTCCTCATAGCTTTTATGTTCAAGATTTAACAAATTCTAGAAAACTCGCGCAATTGGCAGAAGACTTACAAAGAGCCTATAATATTAAAAATGTACAGAAAAATGCTATTTATGATCCGAAAAAAGGCATGCTTGTTGCTGCTCGTTATAGTAAAGATTTAAAGTGGTATCGAGCCAAAATTATCGACTATCTTGAAGGAAGGAAAGTTGTAGTATTTTTTATTGATTTTGGAAACGAAGAGACATTAGTTTGTGATAATATTAAAATACTGTCACAAAGATTTTGCAGTTTCCCTGCACAGGCTCTTAAATGTTCACTCACAGATGTGTACCCAGCTGATGGAATTGATTTTTGGACAGAAGAAGTTTGTACAAAATTCCACAAACTGGTCTTTGATAAACAATTTAAATCAATAGTTGATGGCGTAGGGAATGGAGAACTTAACCTTGTTCTCTTTGAAGTTTCTAAGGAAATGGATGTGTGTATCAATGCGAAGATTGTAAAAGAAGGTTTAGCGATGAGCACAGGACCCAACTCTACTATTGTCGAGTTGCCTAAAATTTCTGCCAATCAAATAGATCCGACTTTTCAGATTATGGCCGTAGAAAAGAAAAAAGTCAAAAAGAAAACCGAAAGGTCATGTAGTAAATCTGATGGCGATTCTCAAGCTATGGACCACTTCATTAAGATGCCCGATGGTGTTAGGACTAAAGTAGTCATTAAAAATATTATCAATCCTGGAGAATTTTACATTATGCCTTACTGTTTTAAAGATAGAATCAATAAGTTAAAATTTAGTCTCCAGGAGTTTTATGAAAACAAAGCTACTGTTTGTCAAAAGGAATGGGCTGTTGATGATAGGTGTGTCGTCAATTTTGAAAATAGCTGGCATAGGGCTGTAATATCAGAACTATTAGTGAATGCTCAAGTGAAGATTAATCTAGTCGATGAAGGATGTGAAAAAATAGTACATATTAAAAATATCGATGTAATGGAAAAAATATTCACGGAAATTCCAGACGGTGTTGTAAAATGTCATCTTGGAGGGCTAGAACCTGCACTTCATCAGTGGTCAGCACTTTCTATTTCTGAATTTGAAGAATTTGTGAATAACAGAAAAGATGGCCTCTATATCAGCCAATTAGGAAAAATAAAAGGTGATTCCCTACCAGTGGAACTCTTCTCCAGAAGACCTCTAGATGTCGGCCCGACTGAACCTATGAAAGATGACTGGAAAAGTCTGAATCATTACCTTCGATTTACTGGTTTCGCCAAGGCAGATGGAATGATCAAATGGGATAGTGATGGAAAAATTTTAAGTATGTCTCAAGACAGTAATAACAGTTATTCATCAATTTTAGATGAATTGATTAGAGATTCATTTGGTTTAAATGAGGAAAATGTTGAAGAAAACACAAGTTGTGCTGAAATCGTTGTTGATGATTATGGAACAGTGGTTAGTAGCTGGCTGCCTCCAGAGCCGTTGAAGAACTTGGAATTCATCGCAGCTCCTACTTATGTCGATGAAAATTGTATCGTTTATCTTCACAATTTTCATGAGAGTGAGCAATTGTTGGAAGAAATTAGTACTGCCCTAGGAACAAAATATGATAATAGTGTACCTCATTTACATGATTCAATGATAGAGGAGGGTGATATATGTGTTGCTAAGTATCACCTCGACAATAAGTGGTACAGGGCTGTTCTTTTAGAGAAATGTTTAGAAACTAGTCAATATAAAATACAATTTGTCGATTATGGAAACATTGAGATGTGTAAATTGACAGAATTGAGGAAAATTCCTGTTGCCCAGCATATACCTATACAGTGTTATAAATGTTGCTTTCATAAACTCAAGCCCATTGACCCTTCAGGTGTTTGGAGACCGAGACACATCGAAATAATTCAATACTTACTTGTAGACAGGCACTGTCAGATAAAACTAGTTAAAATCCCTGATTCTGATTTATTTGGGATTGAAAAACTTATACTACCAGATGGTATTAGTTTTGTCGGTGAAATGGTAAAAGAAGGTTTTGCCATGTACAAAAATAAGTTAGAAGTTGATCCAAATTATATAAGTTCAGATGATATAGGTGATGATAATACTGAGGAACTAAATATAAAATCAGAATCAGAGAGTGTTTGTAGTGTCATTGAAGTAAAACCTGATTTGGAAAGGA

Protein: RF -3: -285 -> >4169 (1294 aa)

Comparison with *Halyomorpha halys*, PREDICTED: tudor domain-containing protein 1-like isoform X2 - Sequence ID: XP_014284230.1

E= 0.0; bits= 2031

Query 1 MLCNECGDNTSDFHCKQCEAPYCQQCFKLVHSSAKAFRNHVRMSLLDSYLCLDADLSMMC 60

MLCNECGDNSD+HCKQCEAPYCQQCFKLVH+SAKAFRNHVRMSLLDSYLCLDADLSMMC

Sbjct 26 MLCNECGDNPSDYHCKQCEAPYCQQCFKLVHASAKAFRNHVRMSLLDSYLCLDADLSMMC 85

Query 61 LTHSKYKLEFFCLPCHTAVCSHCLVESHNGHVVDKIENVDINFEEEFIQRYMDVSNIVAR 120

L H+K+KLEFFC+PCHTA CSHCLVE HNGH VDKIENV+ +FEEEFIQRYMDVSNIV R

Sbjct 86 LNHTKHKLEFFCIPCHTAACSHCLVELHNGHAVDKIENVEFDFEEEFIQRYMDVSNIVTR 145

Query 121 MKSSKKTAENILQHDKDICEIKKTDGEITDHFILLHGKLSLIEHKLRKKLSSANTRKHLE 180

MKSSKK AENIL ++KDI EIKKT+ EIT++F+LLHGKLSLIEH LRKKLSS NTR++LE

Sbjct 146 MKSSKKLAENILHNEKDISEIKKTEEEITEYFLLLHGKLSLIEHNLRKKLSSVNTRRNLE 205

Query 181 EILKQVTGYIEDAETILASTRSLFTDGSAVGPKCLAVIDNLKSRQQLPCYLIENAFWEEP 240

EILKQVTGYIEDAETILASTR+LF+DGSAVGPKCLAVIDNLKSRQQLPCYLIE+A W EP

Sbjct 206 EILKQVTGYIEDAETILASTRTLFSDGSAVGPKCLAVIDNLKSRQQLPCYLIESASWVEP 265

Query 241 RFCADKEIIKTLENHCHLELVEEPKYQLVTKENIPSGYAISPTKDLSRDEVLKLLKKPYL 300

RF ADK+ IKTLENHC L+LVEEP+YQLV KE +P+ Y +SPTKDL+RDEVLKLL+KPYL

Sbjct 266 RFSADKDFIKTLENHCDLKLVEEPRYQLVPKEKVPTEYTVSPTKDLTRDEVLKLLRKPYL 325

Query 301 IVDSSSVSHSDGTLSPALSQ--TSEKSFSSQTRDHSSQSLFYADEIAVGCTLSVRISHLI 358

IVDSS S S+GT SP+ SEKS+SSQTRDHSS +L Y DEIA+GC+L VRISHLI

Sbjct 326 IVDSSPPSPSEGTPSPSSPPSLASEKSYSSQTRDHSSHALLYPDEIALGCSLRVRISHLI 385

Query 359 SPSHFYVQREIAQKKLLEFQASIRPSQVQLCKPPYNPDKGKLYLTLYEADSKWYRARITK 418

SPSHFYVQRE AQKKLLEFQAS+R SQ+Q CKPPYN +KGK+YLTLY ADSKWYRARIT

Sbjct 386 SPSHFYVQRENAQKKLLEFQASLRTSQLQQCKPPYNLEKGKVYLTLYGADSKWYRARITN 445

Query 419 VIDNKKFEVFYVDFGNSEIVDKSKIRTIPKSSLNMPCLAYRCQLSDCVPRYGDDWDARAI 478

+ID KFEVFYVD+GNSEIVDKS+IRTIPKSSLNM CLAYRCQLSDCVPRYGDDWDA AI

Sbjct 446 IIDPMKFEVFYVDYGNSEIVDKSRIRTIPKSSLNMSCLAYRCQLSDCVPRYGDDWDAEAI 505

Query 479 SLMTEIMDDDFVSITVVDKTSISYLVDLHKVTENSFIDLRQSLVFHELASMTKMISQTTD 538

SLMTEIMD+DFV+ITV+DKT+++YLVDLHKVTENSFI+LRQSLVFHELASMTKM+SQTTD

Sbjct 506 SLMTEIMDEDFVTITVLDKTNVAYLVDLHKVTENSFINLRQSLVFHELASMTKMLSQTTD 565

Query 539 ISVQQSLSTPPQHHYTDGSVFEVHVSNVESPHSFYVQDLTNSRKLAQLAEDLQRAYNIKN 598

I+VQQ+L+TP QH YT+GSVFE H+S+VESPH+FY+QD+TN RKLAQ+ EDLQR YNIKN

Sbjct 566 INVQQTLTTPSQHKYTEGSVFEGHISHVESPHAFYIQDITNCRKLAQMTEDLQRTYNIKN 625

Query 599 VQKNAIYDPKKGMLVAARYSKDLKWYRAKIIDYLEGRKVVVFFIDFGNEETLVCDNIKIL 658

Q NAIYDPKKGMLVAA YSKD KWYR KI+DYLEGRKV +FFIDFGNEETL+CDNIK+L

Sbjct 626 KQNNAIYDPKKGMLVAACYSKDSKWYRGKIVDYLEGRKVKIFFIDFGNEETLICDNIKLL 685

Query 659 SQRFCSFPAQALKCSLTDVYPADGIDFWTEEVCTKFHKLVFDKQFKSIVDGVGNGELNLV 718

SQRFCS PAQALKC+L+DVYP +G D+W EE+CTKFH+L+FDKQFK +VD + NGEL +V

Sbjct 686 SQRFCSLPAQALKCTLSDVYPLNGKDWWDEEICTKFHELIFDKQFKLLVDSIANGELAVV 745

Query 719 LFEVSKEMDVCINAKIVKEGLAMSTGPNSTIVELPKISANQIDPTFQIMAVEKKKVKKKT 778

L++VSKE+D CINAKIVKEGLAMSTGPNS +VE PKIS IDPTFQIMAVEKK+V KK

Sbjct 746 LYQVSKELDTCINAKIVKEGLAMSTGPNSVLVEFPKISGEPIDPTFQIMAVEKKRVNKKI 805

Query 779 ERSCSKSDGDSQ-AMDHFIKMPDG-VRTKVVIKNIINPGEFYIMPYCFKDRINKLKFSLQ 836

+ +K D +S + D F+ MP+G VRTKV++K I NPGEFY+MP CF++R KLKF LQ

Sbjct 806 VKP-NKLDSESPLSDDFFVMMPEGVVRTKVIVKTIHNPGEFYVMPTCFRERTTKLKFDLQ 864

Query 837 EFYENKA-TVCQKEWAVDDRCVVNFENSWHRAVISELLVNA-QVKINLVDEGCEKIVHIK 894

EFY N+ QKEW +DDRCVVN + WHRA+ISELL N +K+NL+DEGCEKIV K

Sbjct 865 EFYGNRRLNHVQKEWNIDDRCVVNCDGIWHRAIISELLPNDFLIKVNLIDEGCEKIVDCK 924

Query 895 NIDVMEKIFTEIPDGVVKCHLGGLEPALHQWSALSISEFEEFVNNRKDGLYISQLGKIKG 954

NI++M+ IF EIPDGVVKCHLGG+EP L+ WSALS+SEFEEFVNNRK+ L+ISQLGKIK

Sbjct 925 NIEIMDNIFAEIPDGVVKCHLGGIEPTLNTWSALSVSEFEEFVNNRKESLFISQLGKIKN 984

Query 955 DSLPVELFSRRPLDVGPTEPMKDDWKSLNHYLRFTGFAKADGMIKWDSDGKILSMSQDSN 1014

+SLPVELFSR PL+VGPTEPMKDDWKS+NHYLRF G AKADGMI+WDSDGKIL+M +D

Sbjct 985 ESLPVELFSRIPLEVGPTEPMKDDWKSVNHYLRFIGLAKADGMIEWDSDGKILNMPED-- 1042

Query 1015 NSYSSILDELIRDSFGLNEENVEENTSCAEIVVDDYGTVVSSWLPPEPLKNLEFIAAPTY 1074

N SILD + +DS L EENV EN+ A I V+ G ++ SWLPPEPL NLEFIAAPTY

Sbjct 1043 NDCLSILDRMFKDSMNLFEENV-ENSYMANI-VEHGGLMLKSWLPPEPLTNLEFIAAPTY 1100

Query 1075 VDENCIVYLHNFHESEQLLEEISTALGTKYDNSVPHLHDSMIEEGDICVAKYHLDNKWYR 1134

VDEN IVYLH+FH+SE++L+EIS+ALGTKYDNSVP LHDS IEEGDICVAKYHLDNKWYR

Sbjct 1101 VDENGIVYLHDFHKSEKILQEISSALGTKYDNSVPRLHDSTIEEGDICVAKYHLDNKWYR 1160

Query 1135 AVLLEKCLETSQYKIQFVDYGNIEMCKLTELRKIPVAQHIPIQCYKCCFHKLKPIDPSGV 1194

AVLL KCLETS+Y IQFVDYGN+E CKL ELRKIPVAQHIPIQ Y+CCFH +KP+DPSG+

Sbjct 1161 AVLLGKCLETSEYTIQFVDYGNVETCKLGELRKIPVAQHIPIQSYRCCFHTVKPMDPSGM 1220

Query 1195 WRPRHIEIIQYLLVDRHCQIKLVKIPDSDLFGIEKLILPDGISFVGEMVKEGFAMYKNKL 1254

W+ R +EIIQY +VD++CQIKLVKIP SDLFGIE+L LP+G +V EMV++G A+Y+N

Sbjct 1221 WKQRDVEIIQYYIVDKNCQIKLVKIPGSDLFGIEQLKLPEGCDYVAEMVRDGLAIYRNFF 1280

Query 1255 EVDPNYISSDDIGDDNTEELNIKSESESVCSVIEVKPDLE 1294

+ + SD+IGD+N E +N KSESE+VC IE+KP +E

Sbjct 1281 KNTKDV--SDEIGDNNDEVVNTKSESETVCDTIELKPVME 1318

Graphical representation


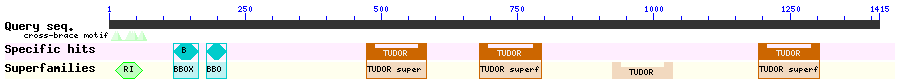


**Translin**

>TRINITY_DN23719_c0_g1_i1 length= 1008 nt

GTTTATTATACATAACAAAATGTAAAAATAATACAAATGTACATATGAATATTCACAATATGACACAATTACTTTGTAAAAAAAGGAAATTTGACAACTTAATTCACCTACAGTACATTTAAGAAGTCTCTTGAACATTAGTTCTTTGGCAAGAATCCAAATTTGATTTGTTTTCAGAAGGCTTTAGACCTCTAATAGACAGATCATATACAACCTCTTCTATTTTCTTCAAGTCATATTTAAAACAATCATAATGTTTTCTTAGAGAGTCATTTTTGAAGTTGAGTAAACGAAACCCAGTATTCAGCTCATTGACGAACTGTGATATGGCAACAGGGCGTTCATAGTCCCCACAAGCAGCACTATTTACTGCCAACCTAGACAGTTCTGTAACCAACTGAAGAAGGCCATGCAAGTAATCTTCCAAGTCTAGGTGAAAGCCTTCTGATTGGTCGATTTTTAAAGAGAGTTCAGAGGCAACCGATTCTCTGGATGCTAGTTTGCCCGACTGTAGGAATTCAATTAAAGCAATGCAAAATACAGCTTTTTGAGTAATACCTCGCCAAATTTCATGATATTTATAATATTCTTTAAGTGGGATTCCTGAACTCAATTTCTGGTAAACTTTGGCACATGATTCAACAAGTTCTTTAGCTCTACTAAGGACTTTTGGAATATCTTTAATTCCAGATTCTAGATGAATCCCTTGAAGGATTGTCATCATTTGCCTTATTTGTAGCTCTAATTCGCGGGTTGTTTCCTGTATTACCTCACGTCGAGCCTGTTCTTCCTCTACATATTTTTGAAATTTCTCAAAAAGGTTCAGCATATCCTCAGAATTAGTAGACATGATTAAAAACCGCACGGACGTTGTTATCACACTAACAAACTGTTAAAAACAATCAGTAAACAAGCCTGAGAAGGTTTCAGCGGGAATAGCAATCACAGAAACAGCTGTGCTCTTTTAGTTTAATTACTGATAACAATGGAGCGCTGACAACGTAGGTG

Protein: RF -3: -850 -> -119 (243 aa)

Comparison with *Halyomorpha halys*, PREDICTED: translin - Sequence ID: XP_014290495.1

E= 1e-154; bits= 434

Query 1 MSTNSEDMLNLFEKFQKYVEEEQARREVIQETTRELELQIRQMMTILQGIHLESGIKDIP 60

M+T++EDML+LFEKFQKYVEEEQARREVIQE+ R+LE IRQMMTI+QGIH ESGIKD P

Sbjct 1 MTTSTEDMLSLFEKFQKYVEEEQARREVIQESARDLEQHIRQMMTIIQGIHQESGIKDSP 60

Query 61 KVLSRAKELVESCAKVYQKLSSGIPLKEYYKYHEIWRGITQKAVFCIALIEFLQSGKLAS 120

KV+SRAK+LV C +VYQKLSSGIP KEYYKYHEIWRG TQKAVFCIALIEFLQSGKLAS

Sbjct 61 KVISRAKDLVGPCGQVYQKLSSGIPPKEYYKYHEIWRGTTQKAVFCIALIEFLQSGKLAS 120

Query 121 RESVASELSLKIDQSEGFHLDLEDYLHGLLQLVTELSRLAVNSAACGDYERPVAISQFVN 180

RE VA+EL+LKIDQ+EGFHLDLEDYLHGLLQLV+ELSRLAVNSAACGDY+RPVAISQFVN

Sbjct 121 REQVAAELTLKIDQAEGFHLDLEDYLHGLLQLVSELSRLAVNSAACGDYDRPVAISQFVN 180

Query 181 ELNTGFRLLNFKNDSLRKHYDCFKYDLKKIEEVVYDLSIRGLKPSENKSNLDSCQRTNVQ 240

ELN GFRLLNFKNDSLRKHYDCFKYDLKKIEEVVYDLSIRGLK SENKS++D C +N+

Sbjct 181 ELNAGFRLLNFKNDSLRKHYDCFKYDLKKIEEVVYDLSIRGLKSSENKSSIDVCPTSNIP 240

Query 241 ETS 243

E S

Sbjct 241 ENS 243

Graphical representation


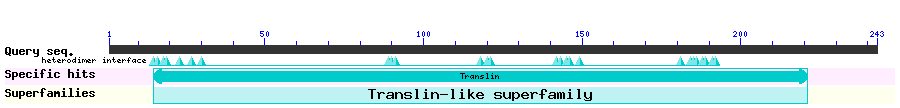


**Similar to Translin associate fator X (TRAX)**

>TRINITY_DN25936_c1_g1_i2 length= 1262 nt

CCTAACAATTACATTGTTTACTGTTTACAAATTTATTTGACATTATTTTACCATATTACTTCTATTTGTTCTTGACATCTTTGTTAATATTTATAAACATCATTTACTTACTATGGGATATGAAGAGAATAAAAGAAAGTATCAAAAACCACATCAAGAGAAAAAACCGATTCCACAGAAAGAGAAAACTCCAGTTATTGAAGCATTCGAAAAATATGCATTAGAATTGAATTCAAAACAGGATAAATCTGAACGACTTGTCAAACTTAGTAGAGATGTAACAATTTCAAGTAAACGTGCTATATTTGCACTCCATGCCCTGATGAGATCGGCAAATTATGAAAAAGATTTGGATAAAATTACTGAAACTTTGAAAACAGTAAGGGAAAGTCTTTTAAAAAATATAGCATTTGAACTCGATGGTGATGACCCTTACCAGTTTTGCAGAAATTATACTGCAGGTCTTCAAGAATATATTGAAGCTGTAACATTTTATCACTTCTTAACCAAAGGCGGACTTTTTAATGTTGAAGTGATTAAAGATGAATTAACGTTTGAAAAAGAAGTTATGGATCAAGGAGAGCCACCTAAAGAAAAAGTACCGCTTCTTGTTCCTCTGTTTGACTACATTCTTGGAGTACAGGACCTAACCGGTGAAATTATGCGGCACTGCATCAGTTGCTTCAGCGCTCGTAATTTAGAAGAGGGCAACAAAGATTGTAATTTTGTCAAAAATCTCTATACAGGAATGCTATTGTTAAATTCATACAGAATGACTGGTATGCAAGGGAGGGATTTTATGAAAAAAATGTCTGTTACCCTCCAATCATTAAAAAAAATGGAAATGGCCTGCTATGCTGCTCATATTCGGGGATCAGAAGTCCTAGATTTTGGATTTAATACAGATGAATTTGAACCTTATACTTGGTGATTAGTTAATAACATAGGATAGTACCTAATCTATTATATTCTCAAGATAAAGTGTTGCATGTTTTTATATGTAATTACATTCATTAATTTTCTATATCGGAAATTGCTATTTCATAGAATTTACTGATTAGTTTAATTTTGATCGGTACTATTCCTCAAAATATGTTATTTGTAATGTAAAAAATGCAAATCCAGGTTTCCACATGTTGAATTCTATAAAAAAGATAGTTCCCTACCCCAATCGAACATATTTTTAGTATATTTGTGACAAATATGTTAATATGACTTTTTCACTGGTTCCTTTAACTTCTTTAGTATAACCCAAGTATGTG

Protein: RF -2: -113 -> -931 (272 aa)

Comparison with *Halyomorpha halys*, PREDICTED: translin-associated protein X isoform X1 - Sequence ID: XP_014289754.1

E= 3e-162; bits= 456

Query 24 EKTPVIEAFEKYALELNSKQDKSERLVKLSRDVTISSKRAIFALHALMRSANYEKDLDKI 83

+KTPV EAFEKYA+ELNSKQDKSER+VKLSRDVTI+SKRAIF+LHALMRS NYE+DL KI

Sbjct 24 DKTPVTEAFEKYAMELNSKQDKSERIVKLSRDVTIASKRAIFSLHALMRSGNYEEDLAKI 83

Query 84 TETLKTVRESLLKNIAFELDGDDPYQFCRNYTAGLQEYIEAVTFYHFLTKGGLFNVEVIK 143

TETL+TVRE+LLKNIA EL GDDPYQFCRNYTAGLQEY+EA+TFYHF+TKGGLFN+EVI+

Sbjct 84 TETLETVRENLLKNIALELAGDDPYQFCRNYTAGLQEYVEAITFYHFITKGGLFNIEVIQ 143

Query 144 DELTFEKEVMDQGEPPKEKVPLLVPLFDYILGVQDLTGEIMRHCISCFSARNLEEGNKDC 203

DELTF+K+VM+QGEPP+EKVPLLVP FDYILG+QDLTGEIMRHCI+CFSARNLE GNKDC

Sbjct 144 DELTFQKKVMEQGEPPQEKVPLLVPFFDYILGIQDLTGEIMRHCINCFSARNLEAGNKDC 203

Query 204 NFVKNLYTGMLLLNSYRMT---GMQGRDFMKKMSVTLQSLKKMEMACYAAHIRGSEVLDF 260

NFVKNLYTG+LLLN+YR GMQGRDF KKMSVTLQSLKKMEMACYAAHIRGSE LDF

Sbjct 204 NFVKNLYTGLLLLNTYRNAMSHGMQGRDFTKKMSVTLQSLKKMEMACYAAHIRGSEALDF 263

Query 261 GFNTDEFEPYTW 272

GFNTDEFE Y +

Sbjct 264 GFNTDEFESYNF 275

Graphical representation


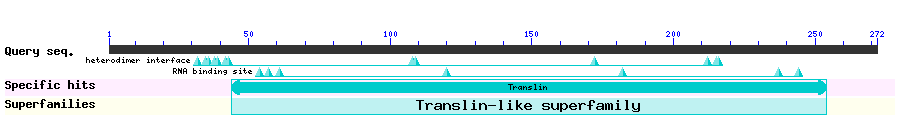


**Armitage**

>TRINITY_DN18678_c0_g2_i1 length= 1885 nt

GGCCGCAGATGAAAGGAAGTATGAGGGAGTCATTCATCGAGTGACCCAAACAGAGCTGTGGCTCCAGTTCGCCAAAGATTTTCACGATTCCTATGGAGAAGGGGTTAAATATTCTGTATCTTTTGTGACATCAAGAGCTTTGCGTAGAATGCATCAAGCTATTAACCTTGCAGCAAAACATCTTGGTTATGGTTGGCTATTTCCGACAGGAGTAGTGCCTAGGTTGCCTCAGGTTGTCGTTGAAGAGGAAATCGAAGAAAATGACTCTACAAAAACGACAAAAAAAGAACGGGGTTCCAAGAAAGTTAATAGTTTACCTGCGGTTCAAACTCCTTCAAGCCTAGCATACAGTCCTGATCCCAGAAAAGTGTCTATTGCCGGACAAGCCAATCTTAGTATTGATCATAAATTTCAGGCTTCATTATTGAAACCCGGATACCGCGGTAGAGGTAGAAGGTACAATAACCGGGATATCCAATGGAACAGGCAAGGTAGTGGTTATGGTATGCACTGCACCATCGACAATGAGGTCCGTGTGAATATTGGTTTAGAGAGAATAAAGAAAATAAGATGGTTCAACAAAGGTCTAAATAGACAGCAAAAAGAAGCTGTGAAGAACGTACTCTTAGGAGAAGCCAGGCCTTTGCCTTATGTTATATTTGGGCCACCAGGCACTGGCAAGACTGTCACAGTCGTTGAAACTATTTTGCAATTACATGCTCTCATTCCAGAAAGTAGATTGCTTGTTGCGACTCCCTCTAATTCTGCTGCGGATCTCATTACCGAACGATTATTGGATGCTGGTGATTTAGAACAAGGAGATCTATTGAGGATGGTCGGTTATCATTATTTAGAACAAGGAAGAATTGCTGCATCCATTGTGCCGTACGCAGCAGTTCCAGATGTTAAAGCTATTAACGTTGCTGGTCTTTCTGGTGCTTCCCATGAAGGAGTGCAGATGTGCGGGAGAGAACTACTCGGTCAACACCGAGTGACTGTAGGAACTCTTGGCTGCCTTGGTCTATTATATAATATGGGCTTTCCTAGAGGCCACTTCACCCATGTCATAGTTGATGAAGCAGGTCAGGCTACCGAACCTGAGTTGCTTATTCCTATGGTTTTTTTGCACATGGAATATGGGCAGGTGGTTTTAGCTGGTGATCCTCTACAGCTTGGACCAGTTGTCACTTCACGTCTTGCGTCAAGATGTGGTCTACAGGATTCTCTTCTTGCTAGATTTCTTAACCGATTTCCATACACAAGAGATCCTAATGGTTTTCCAGACAGTTCAGGCTATGATCCACGCTTGGTAACTAAACTTGTGAACAACTACAGATCACTTCCTACTATACTGGAACTACCCAGCATGCTTTTCTATGATAATGATCTTATCCCTAATGTATCTGAGGATTCGAGTGAAGAAGCAGCACTATTGAGAGCCCTGGCCCCATTACTACCTTGTCGCATATTTGGCGGACGTGCGCCTCCTCTTCTGTTTCATGGTGTCCGTGGCACTAATTGTCAAGAAACTGAATCACACTCATGGTATAACCCTCAAGAAGTGTTTCAAGCCTTTGTTTACTTGAATTTATTGTATAAAGCCGGACTTCGCCCTGATCAAGTTGGAATCATTACTCCATATCAACTACAGTCTAATAAAATCAGGTTTATGTTGGAGCGGATAAATATAGAACCTCCAAAAGTTGGTTCTGTTGAAGAATTTCAAGGGCAAGAAAAGATGGCAATCATCGTTAGCGTTGTAAGAAGTAGCCCAGATTTGATCAGTTATGACATGCAAAGAGCACTCGGTTTTGTAGCTAATGCTAGAAGATTGAACGTAGCATTATCGCGAGCCAGGGCAATCTTGATTATCTTAGGAAATCCT

Protein: RF -2: -149 -> >1885 (578 aa)

Comparison with *Halyomorpha halys*, PREDICTED: probable RNA helicase armi - Sequence ID: XP_014289817.1

E= 0.0; bits= 1110

Query 1 MHQAINLAAKHLGYGWLFPTGVVPRLPQVVVEEEIEENDSTKTTKKERGSKKVNSLPAVQ 60

MHQAINLAAKHLG+GWLFPTGV+PRLPQVVVEEEIEE+ STKT +K+RGSKKVNSLPA++

Sbjct 604 MHQAINLAAKHLGFGWLFPTGVIPRLPQVVVEEEIEESGSTKTVRKDRGSKKVNSLPALE 663

Query 61 TPSSLAYSPDPRKVSIAGQANLSIDHKFQASLLKPGYRGRGRRYNNRDIQWNRQGSGYGM 120

+ L YSPDPRK+SIAG+ NLSIDHKFQASLLK G RGR RRYNNRDIQWNRQGSGYGM

Sbjct 664 S-KDLLYSPDPRKISIAGRTNLSIDHKFQASLLKAGQRGRARRYNNRDIQWNRQGSGYGM 722

Query 121 HCTIDNEVRVNIGLERIKKIRWFNKGLNRQQKEAVKNVLLGEARPLPYVIFGPPGTGKTV 180

HCTIDNEVRVNIGLERIKKIRWFNKGLNRQQKEAVKNVLLGEARPLPYVIFGPPGTGKTV

Sbjct 723 HCTIDNEVRVNIGLERIKKIRWFNKGLNRQQKEAVKNVLLGEARPLPYVIFGPPGTGKTV 782

Query 181 TVVETILQLHALIPESRLLVATPSNSAADLITERLLDAGDLEQGDLLRMVGYHYLEQGRI 240

TVVETILQLHALIPESRLLVATPSNSAADLITERLLDAGDLEQGDLLRMVGYHYLEQGRI

Sbjct 783 TVVETILQLHALIPESRLLVATPSNSAADLITERLLDAGDLEQGDLLRMVGYHYLEQGRI 842

Query 241 AASIVPYAAVPDVKAINVAGLSGASHEGVQMCGRELLGQHRVTVGTLGCLGLLYNMGFPR 300

AA+IVPYAAVPDVKAINVAGLSG+SHEGVQMCGRELLGQHRVTVGTLGCLGLLYNMGFPR

Sbjct 843 AAAIVPYAAVPDVKAINVAGLSGSSHEGVQMCGRELLGQHRVTVGTLGCLGLLYNMGFPR 902

Query 301 GHFTHVIVDEAGQATEPELLIPMVFLHMEYGQVVLAGDPLQLGPVVTSRLASRCGLQDSL 360

GHFTHVIVDEAGQATEPELLIPMVFLHMEYGQVVLAGDPLQLGPVVTSRLASRCGLQDSL

Sbjct 903 GHFTHVIVDEAGQATEPELLIPMVFLHMEYGQVVLAGDPLQLGPVVTSRLASRCGLQDSL 962

Query 361 LARFLNRFPYTRDPNGFPDSSGYDPRLVTKLVNNYRSLPTILELPSMLFYDNDLIPNVSE 420

LARFLNRFPYTRDP+GFPDSSGYDPRLVTKLVNNYRSLPTILELPSMLFYDNDLIPNVSE

Sbjct 963 LARFLNRFPYTRDPHGFPDSSGYDPRLVTKLVNNYRSLPTILELPSMLFYDNDLIPNVSE 1022

Query 421 DSSEEAALLRALAPLLPCRIFGGRAPPLLFHGVRGTNCQETESHSWYNPQEVFQAFVYLN 480

DSSEEA LLRALAPLLPCRIFG RAPPLLFHGVRGTNCQETESHSWYNPQEVFQAFVYLN

Sbjct 1023 DSSEEAGLLRALAPLLPCRIFGTRAPPLLFHGVRGTNCQETESHSWYNPQEVFQAFVYLN 1082

Query 481 LLYKAGLRPDQVGIITPYQLQSNKIRFMLERINIEPPKVGSVEEFQGQEKMAIIVSVVRS 540

LLYKAGLRPDQVGIITPYQLQSNKIRFMLERINIEPPKVGSVEEFQGQEKMAIIVSVVRS

Sbjct 1083 LLYKAGLRPDQVGIITPYQLQSNKIRFMLERINIEPPKVGSVEEFQGQEKMAIIVSVVRS 1142

Query 541 SPDLISYDMQRALGFVANARRLNVALSRARAILIILGNP 579

SPDLISYDMQRALGFVANARRLNVALSRARAILIILGNP

Sbjct 1143 SPDLISYDMQRALGFVANARRLNVALSRARAILIILGNP 1181

Graphical representation


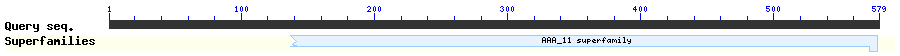


**Homeless (Spindle E)**

>TRINITY_DN27544_c0_g1_i2 length= 4674 nt

CTGTAACCTCGCCTCAGCGTGTGTCAATCTTTGCTAAGGTTACTTAGACTATGTTTCGCTCTGTCAGGATCAGTCTTAGTCTATTAATCTCAGATTGTTGACTTGGTAGACTACTGAAGAAGTTTGGTCAAGGATTCTTGGCCAGTACATTGAAAACATAATGGATTTTGCGACTGTCCTTCAGAGGGCCAGAGATGGCCTTCCTATTCGAAGGATTCAAATCGAAGGTGGTCAGACTGAGGGTCGTATCGCTCCCGACATCAAAAAACCAAAAGCTAAAGGAAGAACCATTATGTCTGGACTACAATATGCTGAACATAGTAGGGCTCAAGATAGGCTAGAACAATTGAAGTTGGATGAAGAATTGAAGGATGAAAAGAATATGTATGGCTGTGAAGAACTTACTTCTGTTGGTACATTACCGAATCTTAATGATTTGAGACCTGAGAATTTAACCAACGTTTACCAAAGTTATAACTTCAAGACAAAGTTAGGTGTAAATCTACTTATTCATGACTATAAGGAAGAGATTTTAGGGCATGTTGACACTAATCAAGTTGTGATCATAAAGGGAGTTACTGGCTGTGGTAAAACCACTCAGGTACCTCAGTTCATCCTTGATCAACATGAAGAAATGAATATTCATTGTAATATTGTTGTCACTCAGCCGAGGCGTATTGCAGCTATTTCAGTAGCAAAGAGAGTCTCCATTGAGCGAGGGTGGCCATTAGGTTCCATTGTTGGATATCAGATCGCTTTGGAACGAAACTGTTCTCAAGATACCCGCCTTATGTATTGCACTACTGGTGTTCTTCTTCAGCAGCTTGTAAAAAAACAATCCCTGTCTGATTACACTCACATCATTGTTGATGAGGTTCATGAGAGAGACAATGAAACTGATTTCCTCCTCATTATATTAAAAAAATTGTTGAGAAGTGCGCAGTGCAGAACGAAGGTTATTCTGATGTCAGCAACGATGAATGTAAATCAATTTTCAGAATACTTTTCCAGAACTGTAAATGGGGTTACCATTGACCCACCCATTGTAGAATTGGCTCATATGGCCAAATTCCCAGTTCAATATTACTACCTGGACAGCCTTGTTAACAGGTTACCAGGTATAAAAGTACCTGAAATTATTATGAGCAATCCTTCAATTTCAACTTATTCTTATGACTTGGCTAGAGCTCTTATAGAGGTTTTCCGTGTGATTGATGAACATGAGCATCAAGATAATAATTTTATTGGTTCTGTTCTTATATTTTTACCTGGTATTGCAGAAATCGAAACAATGTTTGCTCGACTCCAAAATTACACAGGGGCTGTTGAAAAGTGGTGGCTTTGCCCCCTCCATTCATCGATCACCTACGACGAACAAATGAAGGCCTTCCAGCCTGCTCCTAAGGGCCACCGCAAGATAATTCTTGCTACAAACATTGCTGAAAGTTCCATTACTGTACCTGACATTAAGTATGTAATAGACTTCTGCCTCGCAAAGCAACAGGTTTTGGAACCTGAAACGAGCTATTCTTGCCTACAGCTGACCTGGATCTCTAAATCACAGGGAACCCAGAGAGGTGGTAGGGTTGGAAGGGTCATGCCCGGCCGAGTTTACCGCCTCATACCAAAGGATGAATATATGGAGTTGCCCGAAGACATAACTCCAGAAATAATGAGATGCCCATTGGACCAGTTGGTCTTGAAAGCAAAGCAGTTGAGGATGGGCGATCCAGCTACACTGCTAGGTCTTGCTATAGACCCTCCTGACCTGTCGAACATACATAAGACCATTCTTCATTTAAAAGAGGCAGGTGCACTTCTCTTAACCGCTAATGGAGAATACAAAGATAATGATGGTGACTTAACTTTCATCGGTCAGATTATGGCATCGCTTCCACTGGACATCCATCTTTCCAAGCTTATAATTCTTGGTCACATGTTTTCTTGTCTGTCAGATGCCATAATAATGGCCAGTGCAATGTCCGTGAAGAGCGTCTTTAGCACACCTTTCAGGCAGCAACTGGAGGCCTATAACTCAAAATTAACTTGGGCAGATTCCTCATGTAGTGATCCTATCGCATATCTCCATGCTTATTCGCTTTGGAAATTCAAATCAAAAATGGGTTACTTCAAGAGATCAGGTGGTGAAAGTGAGCTAGATTGGTGTCGAAAATGCTTCATTCAAGGGAATCTTATCAAAGAGGTTTCTAGATTAGAAAATGAAATAGTACAGCGTTTGAGGGCGTTAGGGCTTGAAGAAATGAAAGGAGACACAAGCGTGTCATGGACTGCTGCAGAGAAGCCTATTATTCTTAAGCTCATGATTGCTGGAGCATTTTATCCCAATTACTTCATTCCTTTTGGCTCCGACGAGAAGGATGCTGTTAAACTGCTCGGCGGACGAGATCCACACAACACTGTGTACCTGACAGGATTACCCAATAACCAGCCAGGCCCTCTTTACACACATGCCATACGAAACCATTTTCTCCATTGCGGCTCAAATATAGAAGTTTCATTTGACGGAAGCAGCAAAGTTTATTTAACATTCGGAAGCAGTATCCCAGTGAACCAAGACCCCGAGAAACCCGGTTTGATGCCAGGAAAAATCAGTATGGCTGTTTATAGGAGTATTAAACTTCGCCAGCTTCAAATCCCGATCACTGTACCAGTTTTATTACCTCATGAAGCTCGAAAAAGGGCCCAAGAGGTTTTTGGTGAAAGATTGAAACCAAGCCTGTTCAATGTTAACAAAAGGAAAGCAGTAATGCCCATTAAGAAGACCAATATGCCCTCTCTCGGGACTAGCATTCTTCCTGTCGTTATCACTCATGTTGAAACTCCTTCCAGGTTTTGGGTAAATATTAATGAACCCATCAACACAAACAGGATTTATTGGATTCACGCTTCTCTTAACAAAATGACTGAACCCCTACCATTATATAGCGGTTCCTACGAAGATGGCTCTCCTTGTATTGCCCCTTATAAAGATAAGAATGGTCCTTCCGAGTACTACAGAGCTCGCATCATCAGTAGTATTGAGATAGATCCTAGAGGAACAGTTACAAGGGTTCAGGTTTTCTTCATCGATTACGGTAATCAAGAATCTATTTTCACAAAGGAACTGAGAGACTATCCTGAATCTTTAACTAATGTAAAGGAGGAACCAGACCTAGCGCTTGAATCAAGCATGGCCGAGATTGGACCATCCTATAATAGAAACCCAAAAGGTGATTGGTCACAGGAGATCTGCAGGGAATTCCCTAAATTCTTCAAAAAGAGTACAGGTATAGCCAAAATTTTTTCAGTTGTCAACAACGTAATGGCAGTAACATTGTTTTCATCAGAGTTACTTGATAAAAACCCGAGGACTGATTTTCCTTTTGAAATGTCCTTTAATTACTATCTAATAAAGAGTGGTTTCGCAGAACCTATAGACGAGCCTTATCTATCAAGAGAAAACCATATGTTGAGAGAAACTTCGGAAAGGTCATCTGTAAACAGTCTTGATTTATTGAATGCTGACCTGTACAATGATAATTTGTCTGATATTAATTTTACTCCTCCTAAACCTGAAGAGTGCCGAAGTAAGATAACTCTGAAAGGTCCTCGATCACCGTTGGAAATGAACCTTTATTCTCTTCCTAAGAAGTGTCAAGGAAAAGAGACGATCATTGAATGGAATTCTGTTAATTCTGTTCTCTTGGACAATTATCCTATGGACCCACATAGCAGGTTGGTCATTGCAGCTTCTGTGACGCAGAATGCTGGAACGAATCGATTAACATTGAGAAACACAACAATTATGCCTAACATCCATGGGCTAAGTTCTTTGGTCTGCTTGCTCTTTGCTCCTAGGATAGAACTGAGGGCAGACAATGAAAGACGCCAATTGACCGGTGCGCTCTGCGGACTCGGCTTTAAACCTGAAACTGGAGAAGTTTTTTACCCCGAAAATGATCTTGAAGTGCTTTTTGATACTAAGATTACTCTTATGGATTTAGAATTGGTTAATAAGTTGAGATTTTGGATGGACTATATCATGGGCGGGGGAGAGGGCCCTCACAGTGAGCTGTCAAGGCCAGGCATTATTAAGTCACAGGAGAGGATAAAAACATACATCTTAGACCTGCTCTTCAAGAAACGCCCCTGTATTACCCCTCAACCCGTCTCACATCCGTACGAGTGGGACCAGTTAGAACCTGATGAGATCCTCGATCCTCAAGGTAACGACACCAGCCTCTATCCTCTCATCTGGGGTGTATCCCTCGTTGGAGAAGGTGCTAGGGACAATGCTATTCTGTCAAGACTGGAAACTCTTAACATGATTGCTGAGGGCAAAGAGATTTTCAAGACTGCTGTTCAATGTGAGTTGTGCCAAGTGTACAACGAAACATTACAAGAACTGAGGTTGCATCTTACGACTACTTTGCACACAATGAAACTAGCCGATTTCAAGGAGAGCATGAGAGCTGAAAGAAGGAAATAACGTGATAACTCATAGGTTGTCTTCTTGAGTCACCATTTTAAATTTAATTTGATTAATCTCTTTTAGATTTAGTTTCCCTTCTGAATTTTACTTTTTGTTAAAAGTTTTATACATTTTATCATGGCTTTTTGTTCATAAAAGTTTCTTTGAATATTTTATTTTTTTTAAAGTAAAAAGTTTTGGTT

Protein: -2: -161 -> -4489 (1442 aa)

Comparison with *Halyomorpha halys*, PREDICTED: probable ATP-dependent RNA helicase spindle-E - Sequence ID: XP_014286769.1

E= 0.0; bits= 2707

Query 1 MDFATVLQRARDGLPIRRIQIEGGQTEGRIAPDIKKPKAKGRTIMSGLQYAEHSRAQDRL 60

MDF TVLQRAR+GLPIRRIQIEGGQTEGR A + +KPKAKGRTIMSGL YAE SRA DRL

Sbjct 1 MDFETVLQRAREGLPIRRIQIEGGQTEGRTAMETRKPKAKGRTIMSGLHYAEESRAIDRL 60

Query 61 EQLKLDEELKDEKNMYGCEELTSVGTLPNLNDLRPENLTNVYQSYNFKTKLGVNLLIHDY 120

EQLKLDEEL KNM+GCEELTSVGTLPNLNDL+P+NLTNVYQSY FKTKL NLLIH+Y

Sbjct 61 EQLKLDEELTSNKNMFGCEELTSVGTLPNLNDLKPDNLTNVYQSYCFKTKLSTNLLIHEY 120

Query 121 KEEILGHVDTNQVVIIKGVTGCGKTTQVPQFILDQHEEMNIHCNIVVTQPRRIAAISVAK 180

KEEILGH+DTNQVVI+KGVTGCGKTTQVPQFILDQHE +NIHCNI VTQPRRIAAISVAK

Sbjct 121 KEEILGHIDTNQVVIVKGVTGCGKTTQVPQFILDQHEGINIHCNIAVTQPRRIAAISVAK 180

Query 181 RVSIERGWPLGSIVGYQIALERNCSQDTRLMYCTTGVLLQQLVKKQSLSDYTHIIVDEVH 240

RVS ERGWPLGS+VGYQIALERNCSQDTRLMYCTTGVLLQQLVKKQSLSDYTHIIVDEVH

Sbjct 181 RVSNERGWPLGSVVGYQIALERNCSQDTRLMYCTTGVLLQQLVKKQSLSDYTHIIVDEVH 240

Query 241 ERDNETDFLLIILKKLLRSAQCRTKVILMSATMNVNQFSEYFSRTVNGVTIDPPIVELAH 300

ERDNETDFLLIILKKLLRSAQCRTKVILMSATMNV QFS+YFSRTVNGV+I+PP+VELAH

Sbjct 241 ERDNETDFLLIILKKLLRSAQCRTKVILMSATMNVTQFSDYFSRTVNGVSIEPPVVELAH 300

Query 301 MAKFPVQYYYLDSLVNRLPGIKVPEIIMSNPSISTYSYDLARALIEVFRVIDEHEHQDNN 360

++KFPVQYYYLDSL NRLPGIK+PEI ++NPSISTYSYDLARALIEVFRVIDEHEH DNN

Sbjct 301 ISKFPVQYYYLDSLTNRLPGIKIPEINLNNPSISTYSYDLARALIEVFRVIDEHEHHDNN 360

Query 361 FIGSVLIFLPGIAEIETMFARLQNYTGAVEKWWLCPLHSSITYDEQMKAFQPAPKGHRKI 420

FIGSVL+FLPGIAEIETM+ARLQ+Y G+ E WWLCPLHSSITYDEQMKAFQPAP+GHRK+

Sbjct 361 FIGSVLVFLPGIAEIETMYARLQSYAGSAEMWWLCPLHSSITYDEQMKAFQPAPRGHRKV 420

Query 421 ILATNIAESSITVPDIKYVIDFCLAKQQVLEPETSYSCLQLTWISKSQGTQRGGRVGRVM 480

ILATNIAESSITVPDIKYVIDFCLAKQQVLEPET YSCLQLTWI+KSQGTQRGGRVGRVM

Sbjct 421 ILATNIAESSITVPDIKYVIDFCLAKQQVLEPETGYSCLQLTWITKSQGTQRGGRVGRVM 480

Query 481 PGRVYRLIPKDEYMELPEDITPEIMRCPLDQLVLKAKQLRMGDPATLLGLAIDPPDLSNI 540

PGRVYRLIPKD YMEL EDITPEIMRCPLDQLVLKAKQLRMG+PATLLGLAIDPPDLSNI

Sbjct 481 PGRVYRLIPKDYYMELSEDITPEIMRCPLDQLVLKAKQLRMGNPATLLGLAIDPPDLSNI 540

Query 541 HKTILHLKEAGALLLTANGEYKDNDGDLTFIGQIMASLPLDIHLSKLIILGHMFSCLSDA 600

HKTILHLKEAGALL+TANG+YKDNDGDLTFIGQIMASLPLDIHLSKLIILGHMFSCLSDA

Sbjct 541 HKTILHLKEAGALLMTANGQYKDNDGDLTFIGQIMASLPLDIHLSKLIILGHMFSCLSDA 600

Query 601 IIMASAMSVKSVFSTPFRQQLEAYNSKLTWADSSCSDPIAYLHAYSLWKFKSKMGYFKRS 660

IIMASAMSVKS+FSTPFRQQL+AYNSKLTWADSSCSDPIAYLHAYSLWKFKSKMG+FKRS

Sbjct 601 IIMASAMSVKSIFSTPFRQQLDAYNSKLTWADSSCSDPIAYLHAYSLWKFKSKMGFFKRS 660

Query 661 GGESELDWCRKCFIQGNLIKEVSRLENEIVQRLRALGLEEMKGDTSVSWTAAEKPIILKL 720

GGESE+DWCRK FIQGNLI+E SRLENEIVQRLR LG+EEMKG+TSVSWTAAEKP+ILKL

Sbjct 661 GGESEIDWCRKVFIQGNLIREASRLENEIVQRLRGLGVEEMKGETSVSWTAAEKPVILKL 720

Query 721 MIAGAFYPNYFIPFGSDEKDAVKLLGGRDPHNTVYLTGLPNNQPGPLYTHAIRNHFLHCG 780

+IAGAFYPNYFIPFGSDEKDAVKLLGGRDP +TVYLTGLPNNQPGPLYTHAIRNHFLHCG

Sbjct 721 IIAGAFYPNYFIPFGSDEKDAVKLLGGRDPLSTVYLTGLPNNQPGPLYTHAIRNHFLHCG 780

Query 781 SNIEVSFDGSSKVYLTFGSSIPVNQDPEKPGLMPGKISMAVYRSIKLRQLQIPITVPVLL 840

SNIEVSFDGSSK+YL+FGSS+P N++PEK LMPGKISMAVYRSIKLRQLQ+PITVPVLL

Sbjct 781 SNIEVSFDGSSKIYLSFGSSVPTNREPEKADLMPGKISMAVYRSIKLRQLQVPITVPVLL 840

Query 841 PHEARKRAQEVFGERLKPSLFNVNKRKAVMPIKKTNMPSLGTSILPVVITHVETPSRFWV 900

PHEARKRAQEVFG+RL PSLFNVNKRKAVMPIKKTNMPSLGTSILP+V THVE+PS+FWV

Sbjct 841 PHEARKRAQEVFGDRLTPSLFNVNKRKAVMPIKKTNMPSLGTSILPIVFTHVESPSKFWV 900

Query 901 NINEPINTNRIYWIHASLNKMTEPLPLYSGSYEDGSPCIAPYKDKNGPSEYYRARIISSI 960

N+NEP+N+NRIYWIH+SLN+++EPLPLY+G Y+DGSPCIAP+ DKNGPSEYYRARIISSI

Sbjct 901 NVNEPVNSNRIYWIHSSLNRISEPLPLYTGPYDDGSPCIAPFNDKNGPSEYYRARIISSI 960

Query 961 EIDPRGTVTRVQVFFIDYGNQESIFTKELRDYPESLTNVKEEPDLALESSMAEIGPSYNR 1020

++D G VTRVQV FIDYGNQESI TKELRDYP+SLTNVKEEPDLALE+S+AE+GPS+ +

Sbjct 961 DVDQSGAVTRVQVIFIDYGNQESICTKELRDYPKSLTNVKEEPDLALEASLAEVGPSFTK 1020

Query 1021 NPKGDWSQEICREFPKFFKKSTGIAKIFSVVNNVMAVTLFSSELLDKNPRTDFPFEMSFN 1080

NP+G WS E C+EF +FFK +TGIAKIFSVVNNVMAVTLFSS+LL K PFEMSFN

Sbjct 1021 NPRGGWSPESCKEFSRFFKNNTGIAKIFSVVNNVMAVTLFSSDLLSKKSEAHIPFEMSFN 1080

Query 1081 YYLIKSGFAEPIDEPYLSRENHMLRETSERSSVNS--LDLLNADLYNDNLSDINFTPPKP 1138

YYLIK+GFAEPIDEPYLSRENHMLRE SERSS + L++LNADLYNDNLSDINF PP+P

Sbjct 1081 YYLIKNGFAEPIDEPYLSRENHMLREASERSSSTNHCLEVLNADLYNDNLSDINFEPPRP 1140

Query 1139 EECRSKITLKGPRSPLEMNLYSLPKKCQGKETIIEWNSVNSVLLDNYPMDPHSRLVIAAS 1198

EECRSK+TLKGPRSPLEMNLYSLPKKCQGKETIIEWNSVNSVLLD YPMDPHSRLVIAAS

Sbjct 1141 EECRSKVTLKGPRSPLEMNLYSLPKKCQGKETIIEWNSVNSVLLDTYPMDPHSRLVIAAS 1200

Query 1199 VTQNAGTNRLTLRNTTIMPNIHGLSSLVCLLFAPRIELRADNERRQLTGALCGLGFKPET 1258

VTQNAG+NRLTLRNTTIMPNIHGLSSLVCL+FAPR+ELRAD+ERRQLTGALCGLGF+PET

Sbjct 1201 VTQNAGSNRLTLRNTTIMPNIHGLSSLVCLIFAPRVELRADSERRQLTGALCGLGFEPET 1260

Query 1259 GEVFYPENDLEVLFDTKITLMDLELVNKLRFWMDYIMGGGEGPHSELSRPGIIKSQERIK 1318

G FYPENDLEVLFDTKITLMDLE+VNKLRFWMDYIMGGGEGPHSELSRPGIIKSQERIK

Sbjct 1261 GAAFYPENDLEVLFDTKITLMDLEVVNKLRFWMDYIMGGGEGPHSELSRPGIIKSQERIK 1320

Query 1319 TYILDLLFKKRPCITPQPVSHPYEWDQLEPDEILDPQGNDTSLYPLIWGVSLVGEGARDN 1378

+YI DLLFKKRPC+TPQPVS+P+EWDQLEPDE+LDPQG DTSLYPLIWG+SLVGEGARDN

Sbjct 1321 SYITDLLFKKRPCVTPQPVSYPHEWDQLEPDELLDPQGCDTSLYPLIWGISLVGEGARDN 1380

Query 1379 AILSRLETLNMIAEGKEIFKTAVQCELCQVYNETLQELRLHLTTTLHTMKLADFKESMRA 1438

AILSRLETLNMIAEGKEIFKTAVQCELCQVYNETLQELRLHLTTTLHT+KLADFKESM+A

Sbjct 1381 AILSRLETLNMIAEGKEIFKTAVQCELCQVYNETLQELRLHLTTTLHTIKLADFKESMKA 1440

Query 1439 ERRK 1442

ERRK

Sbjct 1441 ERRK 1444

Graphical representation


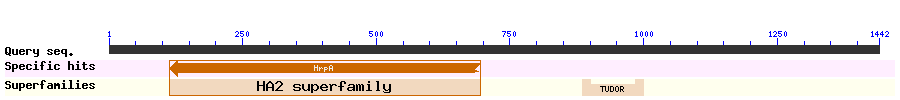


**Maelstrom**

>TRINITY_DN28490_c2_g2_i1 length= 1816 nt

ATATCTGAAACTATTAATAGCATTGATAGTCCTGAAAACATCCGGAAGGGAATCGGTGAAAATTTATAAATGAATTTGCTTTTATTCCCGTTAAAGTAGTTTTTGTTGTTTTATAATTTTATAATTCTTACAGTTGGCAGTAATGGTAAGCAATCGGATGGAGTGTTAGTATGCCACAGAAAAAAAAACAAAAAGCTGGGAAAAATGGGTTTTATTATTTTATGTTAGAAGTCCAAAAACAAGAAGCTCATAAAGGCAATAAGTATTCTTTGCCTGAAATTTCTGAAATTGCAAATCCATTGTGGTCTGCTATGTCACCAGAAGAACGTAAACCATATAATGACAAAGCACTAATGAACGTTTCCAAAAGTAATGAAGACTTAACAAAAAAATATACTTCTTTGGGAATTAGTTATGCTGAACTAGATGCCGAACAAAGAGAGTTGGATGAAGCCCGAAATATGATGATTGCTACAATTAAAAATACAATTGCGAATCTTGACACCAGAAGTTCTCTGAAGACTTATACATTCTTTGTATGCCATGCCAACTATTTCTACAAGACTGATCATCATGTTTATTATCCTGCCGAAATAGCTTTTGCTGCCTTTAATCTGGAGCTTGGTGTTCTTGCCACCCTACATTTCTTTGTGGACCCTGGAAAGATTCCTCTTGGTTATAAATATGAAGCTGCAGATTGGTCAGCTAGAACTCATGGAATTCCAGTAAATGATCCTAGTTGGAATGAAGGGGTCAAAGATCCTCTAGAAATGTTCAGAAGGGTTAAGGATTTCCTTAAGAGTTTTGCTGGGGACTCTGAAGTACCTCCTATCTACACAATGTTTGATAATTTAAATGCCAACAGTCGTCATTCTGTCGTAAAGTCTGCTTTGAATTTGATGTGTGATGCTGCAAATGAAGATTCCTCTCTATTTCGTGTTTATTGTTTACCTCATTTATTCTTTGAGATTAGAAATAAATGTTCTGCAGGAATACCAAGTGTGGCCATAATGAAAAGTGAATTAGATAAGGATGTTTTTTCATATGGCAGGGATATAGGATGTTACTATCATGAAGAAAAAGATCTTTCTATGAATTGTTCACTTTCCATAGTAACAAGATGGGTATTTACCATTTGTGATCATTGTTGCAAACATCTTTGTATAAAGCTCATCCTAGGGAGACATGTTCCAAAAGATACAGACATTTCAGAAAGTGCTCGATTTATAGAAGATAAATATTCTCCAAATAAGCTTGATCATAGTGATTCCTATAGTAAACCTTTGACCATCATTGACCATGGACGGTTAAAAGAACAAAGAGCAGAACAAAAAAGAGTTCAGGATAAACACTTGCGTGAAAGTGAAAAAATTAGGCTTCCGAAATCTGTTTACAGCAACAAGATTGGTGCCAGATTCAGTCAAGATTCAGGTGCTAATAGTTCTCAAAGCTCTTTGGAAATGAAAGGAACAAAATCAGAAGCTAATGAAAATGATTGGTGCATTGTTAAAGGGAGAAGCTTAGGACGTGGACGTGGTTTTATACCAGAAGAACCTTCAACTGAAGAGCCTAACTGGCCAGTTTCTGGAAGGGGAAGAGCTTTTCAATAGTAATTTCTTGGAGAAATTTTAATATAGACAAAAAACTTTGTAATTTTAATATGGACGAAAAAGATTGTTATTTTTAATATTTTTTTATGTAACAGTTTCCTATACTGTAAATAAATTTTTGTGTGTGTATGTATATATACAAACTATATATATATATATATTTTTATATTTATTTCAATTATGATAGAAAATCAAAAGAATTGAAG

Protein: RF 3: 171 -> 1610 (479 aa)

Comparison with *Halyomorpha halys,* PREDICTED: protein maelstrom homolog isoform X1 - Sequence ID: XP_014290039.1

E= 0.0; bits= 694

Query 48 MSPEERKPYNDKALMNVSKSNEDLTKKYTSLGISYAELDAEQRELDEARNMMIATIKNTI 107

M+PEERKPYNDKAL +VSK +ED TKK+TSLGISYAELDAEQREL EA N+M+ATIKNT+

Sbjct 18 MTPEERKPYNDKALTSVSKDHEDNTKKFTSLGISYAELDAEQRELAEAHNLMLATIKNTV 77

Query 108 ANLDTRSSLKTYTFFVCHANYFYKTDHHVYYPAEIAFAAFNLELGVLATLHFFVDPGKIP 167

ANLD SSLK++ FFVC+ NYFYK DHH+YYPAE+A AAF+LE GVL T+HFFVDPGKIP

Sbjct 78 ANLDINSSLKSHKFFVCYVNYFYKNDHHIYYPAEVALAAFSLESGVLGTIHFFVDPGKIP 137

Query 168 LGYKYEAADWSARTHGIPVNDPSWNEGVKDPLEMFRRVKDFLKSFAGDSEVPPIYTMFDN 227

LGYK+EAADWS RTHGIPVNDPSWNEG+KD EMF++VKDFLKSF+ +EVPPIYTMFD+

Sbjct 138 LGYKFEAADWSTRTHGIPVNDPSWNEGIKDHFEMFKKVKDFLKSFSDSNEVPPIYTMFDS 197

Query 228 LNANSRHSVVKSALNLMCDAANEDSSLFRVYCLPHLFFEIRNKCSAGIPSVAIMKSELDK 287

LNANSR S+VKSALNLMCDAANED LFRVYCLPHLFFEIRNKCS IPSVAIMKSELDK

Sbjct 198 LNANSRLSIVKSALNLMCDAANEDPDLFRVYCLPHLFFEIRNKCSKEIPSVAIMKSELDK 257

Query 288 DVFSYGRDIGCYYHEEKDLSMNCSLSIVTRWVFTICDHCCKHLCIKLILGRHVPKDTDIS 347

DVFSY RD+GCYYHEEKDLSM CSLS+VTRWVFTICDHCCKHL IKLILGRHVPKDTD+S

Sbjct 258 DVFSYARDLGCYYHEEKDLSMYCSLSVVTRWVFTICDHCCKHLGIKLILGRHVPKDTDLS 317

Query 348 ESARFIEDKYSPNKLDHSDSYSKPLTIIDHGRLKEQRAEQKRVQDKHLRESEKIRLPKSV 407

ES RF++DKYSPNKLDH S+SKPLTIIDHGRLKEQRAEQKR QD+HLRESEKIRLPKS

Sbjct 318 ESTRFMDDKYSPNKLDHCGSHSKPLTIIDHGRLKEQRAEQKRAQDRHLRESEKIRLPKSS 377

Query 408 YSNKIGARFSQDSGANSSQSSLEMKGTKSEANENDWCIVKGRSLGRGRGFIPEEPSTEEP 467

YSNKIGARFS D GA+SS+SS+E K SE W V+G+ GRGRGF+ +EPS + P

Sbjct 378 YSNKIGARFSNDLGASSSRSSVEKKEESSENE---WRTVRGKGFGRGRGFLVQEPSAKAP 434

Query 468 --NWPVSGRGRAF 478

WPV+GRGRAF

Sbjct 435 VSAWPVTGRGRAF 447

Graphical representation


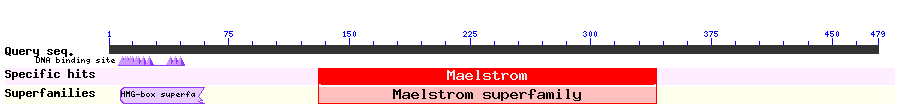


**HEN1**

>TRINITY_DN25538_c0_g1_i1 length= 2992 nt

GACAAATATGGTAACATCAGTCTTAATTATATTATTTCCAATCATCCAGTATTGGCACATGGGAGATGTTAATTTTTTAAGCACTTATAAGTATGAAAGCATTGCTTAACTATGAAAATATTTACAATACACATTTTCACAAATGATATGTTGAATAATAAAACATAATGTATAAATTAAATTTCTTAATATTGACAATCTGTGTTAATCTTCGGGTACAGTTGGTTCGCCACCAGCCCTTGCATCACCTGGATAAAAATCAACTTGTCCTTCCCTGTCTACAACTAAATCACTACTATCATTATCCCTATCTGGGCCTGCATCACCACTACCATCGTCATCCCACATTTCCATGTTCAAGAGCCACGAGGGAAATTGATTCTGATTATGGGCACTACCATCTTCTACCTGAAGGCTGCCAAATGAAGATGGAGATGGTAAGTCAAAAAGGTAAACAGTATCATCATTTGATGACAATTCCTCAGGTACAATGGTATCATTGGATACATCTAAAGCTGAGAGGAAAAGTCCATCATCGTCCGATTCACGCTCATCTGCAGCTTCAACTTCTTCACCACCAACTACTAAGTCCTCAAAAATCAACTCCTCTCCTAGTAACTGACCTCCTTCCATATTGTTGCCTTCACCATCTCTGTTATTATTGGCTACATCTCCATTTTCTACATTGTCAGCAAACACAATTGGAGGGTTACCATGTACAGGCTCGATCCTTTCATCGGCAGAAGAATCTTCATCTTCTAGACTAGATTCCGTTTCAGTAATGATTTCATCAACCTGTTCAGGAGTTAAATCGAGATCCATCTCTTGTGTTGAATTAGGATAGCCACTATCGACTGCTTTACTATGACTACCAAATGTTTCTGTAAGACTAAGAATACTTGATCTCTTACATGTTTCAAGATCCATATCTACTACTGAAGTATCACAGTCAATAATTTTCTTATCTATATTTAAACTATACCTAAAACTTTTGTCTTTACTTTTGTACACATTTACATTTTCCAAAGATTGGCTCCGAGGAACAAGTGATGATGACTGTTCACAATGAACTTGTTTATGACCTTCAACTAAAGTGTCAGCTACTTCTTTACTAACAGGCACCTCTGAACTAGATGATGGAATATCTATATTTTGTTTTATTTCATTTTCGTTAATTTCTTCATTTATTATGTCTTTTTGACCTTCTTCTATAACATTAAGTGAAAATGGCTGATCTTTATTTTGGTGAAAGGCATCAGGTATATTATCTTGAGTTTCAACAGCACCATTACATGAAGATGAAGATGGATGCTGTTCTGTATCAGAAATATTATCTTTTACAGCATTAGTTTCACTGCAGGCATCATTCATATTTTTCTTACTTTTCTCCCCTGGTGAATTCGAGTTTTCCACAAGTGTATCACTTGATTTAGAAGTGTTAGCAGATGTCGAACTTGAATACTGAAGAGTACTATCAACATTTAAATAATCGACTTGATTTTCTCCAACTGGAGTAACAGATTCAGCTCTAAGTTCCTCTTCATCAGATGACAACAAATTGAGATGATCTGGCTCATCTGCTACATTAATGGCAGCATAATTATGTTCCTCTCTTACGTTATCTTCTCTAGACCTGTTTAGTAAATAACCAAATTCATCTGCAAAATCTTCGAGATCATCAATATGGTCACAGTTCGAGTTTGACTCAGGTATCATTTCATTTACATTTTCTAAAACATAGCCCCAATCATCATCATTTGCCACAAAATCATTCAATAAATCATTATTCTCAATTATCCAACTTTCTTCTTCATCACCAATGTCATCAACGATCCCCTCATATGAAGGAAAAGACTCTTCTTCATCTGGTTGACAAGGCACAACTAGTGTAAGTTGATTATTTTTAAGTTCAACATTTTTATTAGATTTTTCCAGAAAAATTTTTAATCTTTCTTCTGTACATGAAACAGTAATGTAATTAATAATGTCCACTAGAGGTATTTCAAATTGATCCCCAATTAAATACTCATCATTTTTTGAATAATGGTCAATAAATTGATAAACCTCAAACTCCAAAGTGTTATTATACTTTTCTTCTTGTGTTTCATAAGGAAATTCTACTTCGTGAACAGTATTATAGTATACCAATCTTCAAACTGTCGACGACTCCATTCAAATTTATGGTCATAATGCCTAAATGTATTTGGTTCGAGGTTTCTAAAGAGCACATTGAAGTCTGCATTAGGTGTAGTAAATATTGCAACATCTGGTTGAACAACACCAAATATGTTATAAGGTAGATCATCCAACTCAGGAGGAAATAAATGTTCAATTAGTTCAATAGCAACTACAGCATTCACATATTTCAGCCTTACATCAGGCAGTGTAGCACTTCCCTTAAAAATACTCACTTTCAGAGGAGTACTCCTTCTTCTTAAATAATCAGCATGCAATGGTGAAACTCTACATATACTTTTTTTAAGTACAGTTTCATCAATGTCAACTCCAATATATTCCTCAACAGTTTCAAGTCGTTTGATATGAGTGAAGAATGCTATTTCGGAGCATCCTATGTCAACAATCTTTTTTATGTCATAGATTAATTGTAATTTAACCAAAATGCTATAAACAGCATGGTATCTTTGAATAAAGGCTGGAGGTAGAAATCTAATTCCCACATCATCAATTATACCTATTCCTTCTAATTCTATACTATCAAGCAGTTCTATTGGGCCAGGTGTGCTTAGATTATCATTGGAGGGGGTACTTTGAAATATCCTTCTTTGGACAACTTTAAAAAGATAATTAATTAAATATAAAAAGGAAACAATCATTATAGGTTGTATTAACTATTAACTGGTTCTCATGTTATTCATCTCAAAGCATTATAGCGAACTATTAATCTCTAAGCCATAAAACGTAGATAAAATTGTTTGTAAACAAACTAAAAGATTGTAGTTGTTCCCAGCTTTACCGGTTATCCCAACAGCTG

Protein: RF -2: -1716 -> -205 (503 aa)

Comparison with *Halyomorpha halys*, PREDICTED: uncharacterized protein LOC106685926 - Sequence ID: XP_014284423.1

E= 0.0; bits= 656

Query 1 MIPESNSNCDHIDDLEDFADEFGYLLNRSREDNVREEHNYAAINVADEPDHL---NLLSS 57

M P+S N D +DD+ D DE +L D N +A+NV +P +LL S

Sbjct 425 MKPQSKPNYDPMDDVNDLVDELDNVLRNPENDVTESGQNNSAMNVMCDPKQSGVNDLLLS 484

Query 58 DEEELRAESVTPVGENQVDYLNVDSTLQYSSST-SANTSKSSDTLVENSNSPGEKSKKNM 116

DEE+LRA+SVTPVGENQV YLN+DS LQ+S ST S NTSKSSDTLV+++ SP K K

Sbjct 485 DEEDLRADSVTPVGENQVQYLNIDSILQHSGSTGSGNTSKSSDTLVDHTLSPMNKIKNCT 544

Query 117 NDACSETNAV---KDNISDTEQHPSSSSCNGAVETQDNIPDAFHQNKDQPFSLNVIE-EG 172

ND SE NA+ K++ SD E++P SS+ N AVE NI + FH+N D+ FS +V++ E

Sbjct 545 NDDYSEKNALSPLKNSFSDKEEYPCSSTINDAVEISLNISNPFHEN-DKQFSCDVLQSES 603

Query 173 QKDIINEEINENEIKQNIDIPSSSSEVPV--SKEVADTLVE---GHKQVHCEQSSSLVPR 227

QK EINEN+ K N++ S SS +PV S V DT+VE K+VH +QS+S+V R

Sbjct 604 QK-----EINENKTK-NLE-NSLSSAIPVGESNRVTDTVVEEVSNSKEVHYDQSTSIVTR 656

Query 228 SQSLENVNVYKSKDKSFRYSLNIDKKIIDCDTSVVDMDLETCKRSSILSLTETFGSHSKA 287

SQSLEN+N+YK K+K FRYSLNID K++DCDT+VVDMDLETCK SSI SLTET+GSHSKA

Sbjct 657 SQSLENINLYKCKEKCFRYSLNIDNKLLDCDTAVVDMDLETCKGSSIPSLTETYGSHSKA 716

Query 288 VDSGYPNSTQEMDLDLTPEQVDEIITETESSLEDEDSSADERIEPVHGNPPIVFADNVEN 347

VDSGYPNSTQ+MDLDLTPEQVDEIITETESSLEDEDSSADER+EPVHGNPPIVFADNVEN

Sbjct 717 VDSGYPNSTQDMDLDLTPEQVDEIITETESSLEDEDSSADERVEPVHGNPPIVFADNVEN 776

Query 348 GDVANNNRDGEGNNMEGGQLLGEELIFEDLVVGGEEVEAADERESDDDGLFLSALDVSND 407

GDVANNNRDGEGNNMEGGQ+L EELIFE LV+GGEEVEAADE ESDDDGLFLSALDVSND

Sbjct 777 GDVANNNRDGEGNNMEGGQVLPEELIFEGLVIGGEEVEAADEHESDDDGLFLSALDVSND 836

Query 408 TIVPEELSSNDDTVYLFDLPSPSSFGSLQVED-GSAHNQNQFPSWLLNMEMWDDD-GSGD 465

TIVPEE+SSN+DTVYLFDLPSPSSFGSLQVE+ S HNQNQFPSWLLNME+W+DD GSGD

Sbjct 837 TIVPEEMSSNEDTVYLFDLPSPSSFGSLQVEEPTSFHNQNQFPSWLLNMEVWEDDSGSGD 896

Query 466 AGPDRDNDSSDLVVDREGQVDFYPGDARAGGEPTVPED 503

AGPDRD DSSDLVVDREGQVDFYPGDARAGGEPT+P+D

Sbjct 897 AGPDRDEDSSDLVVDREGQVDFYPGDARAGGEPTLPDD 934

Graphical representation

**No putative conserved domains have been detected**

**PRP16, mut6 homolog**

>TRINITY_DN28417_c1_g3_i5 length= 4725 nt

TTTTTTTATATTAAGAGTGTGTATTTTAAAAGGTAATATTTACAATAGTTAATATTAAATAAAAGTATTTACAAAAATAATAATAAGTAAAATAAAGATGTCTGATTGTTCTTCATAGTAGTTTGATACGGAGACCTACAGACCAAGCCTAAGTGGAGTCCTAAGAGGTGTACTTCCAGGAGCTCCAGGTTCTGCTACCTGAGAGCTCCTTTTGGACCAACGACCTCGTTCTTCTTCTGCCTGTCTTTCTCTCTCTTTCATTTGTTTCTGGGCAGCCTCCATTTCAGCTTCCATACCCTCCATGGCCTCTCGCCTCTTAGATGAGCCAGAACGTCCAGTTTCTTTGATGGAGAAGAACATTGGACCTAGTTCAGCAAGCCAAACACCGTCTACTGCAGTAACACACTGCATATATTCCTTGGAAGTCATCACAAGCTCATGGTAGACAACATAGTCAGGAGTGAAACCCATCCCAAATAGAGCTGATGTAGGATGAAGATGGCAGGGCATTCCTGTTCTTAAATGAACATACTCCCCTATTCCTTTGAGGCGAGCCCCTTGATGGAAATAGGCTGAACAAATGCATTTTCTGACTATGTCCCAATCAGTACCACATGATACTACTTCCATTTTCTGTTGGTCTAGGATGTCTTTTAGCTGCTGACGAACCTCCCGTACTTTTCTCATGGCTTTAGCATGAATAAAGTGGTCATTGCACCAGGTTGAAGAGTATTTGTTCTGCTTCCATTGATTATAGACATTTAAATAGGTTAAATGATCAGATTCTGGAACTTGAAATTTTTCTCTAACACTGTCTGCTTCATCTTCTCTCCCTTTGGGTCGATAAAAAATTGTTGGTACAGACAACATTGAAACAATAATAAGAATTTCAGCAGTGCACCCCATTTGGTTGGATACAATCAACATTTGACACTGTGGAGGATCTAAAGGAAATTCAGCCATTTGTCTGCCGAGTGGTGTCAATGATCCCGTGTGGTCCAGAGCTCCAAGGATCCACAGTTGGTAGAGAGAATTAAGAATGTTGTCCTGGGGAGGCGGGTCCATAAAATGGAACTGTAATAGATCTTGAACACCCAATGACTTAAGCAATAGAACGGTGTTAGCCAAATTAGTCCTTTGAATCTCTGGAACAGTTGAAGTTAAAAGTTCATCCTTATACTGTCGCTCTGTATATAGCCTGTAAGCATGCCCGGGTCCTGTCCTACCGGCTCTTCCAGACCGCTGGTTTGCGTTAGCCTGAGAGATGGGATATATTTGTAAGGCATCCATACCTATCCTAGGATTGTAAACTTTCAGCTTGCAGTAACCAGAATCCACAACATACATAATACCGTCAACTGTTAAGGATGTTTCCGCAATGTTTGTAGCTACAACACATTTGCGCACTCCATCAGGTGATGCTTGAAAGATTTTAGCCTGTAGGTCAGACGGCAGTTGGGAGTAGATGGGCAGAATAGAGAGAGGAGGTGCGGCATCTATCTCAGCTAGCCTCTCTCCTAACACTTCACAAGTAACTTCAATGTCTTCCTGACCAGGCATGAATATTAGAATGTCACCTTTTGTTGGCTGAAGGTGTATCTGCAATGCTTGCTTGACAGAAGCCTCAACATAGTCCTCGACGGCATTTCTAGCAAACAGTACCTCAACAGGGAATGTCCTTCCAGGGATTGTGTAGACCGGGACATGTCCAAAAAACATGGAAAACTTTGAAGAGTCCATGGTGGCTGAAGTGACTATTAACTTCAGGTCTTGCCTTCGAGCTACCACCTGTCGCAGAAGTCCAAAGAGAACATCTGTGTTGAGAGACCTTTCATGGGCTTCATCCATAATAATTGCAGAATAGTTGTCTAAATCAGGCTCTCTTAGCGACTCTCTTAACAGGATACCATCTGTCATATATTTAATAACAGTGTCCTCCGAAGTACAATCTTCAAATCGAATAGCATATCCCACAGTCTTCCCTAATGGTGAGGCCATCTCATCAGACACTCTCTTAGCCACAGACATTGCAGCAACCCTCCTCGGTTGGGTGCAGCCTATCATGCCTGACTTGGAGTAGCCATCCTCATGTAGGTACTGGGTCAACTGAGTTGTCTTACCAGAACCTGTTTCACCAACAACAACTACAACGCTGTTTTCACGAATTATATTCAGTAGCTCTTGCCTAACAGCAAAAACAGGAAGATATTGCCTCTGCTGTTGAATAGTCTTCTTTTTAGCAAATTCACTGGAGGCTGGGGCCATATCTTTCATATGCTCAGCAAATTTTTGACTAGTCTTATAGTCAGCCTCAACTTCACTATCTATTTTGTCTTCTTCCTTATCTTTCTTCTTTGGAACCCCCATGATGTTACCTATTGTTGTTCCTGACAGTTCCCAATGCTTTTTCTGTGCTCTTTTTCTCTCTTTCTGTTCTCTATATGCTCTAACCAGGGCTGATCCCTTCCTTGAGACCAATGCCATATCTGAAGTTGGATCCTTGACTGGAATCACTGGTTCTGGTTGCTTGGTAAATACAATACGACCATCCAAGAATGGAGGTACTATGTTGTGCACTAAGAGATGGACTCTGGCTTCCGCTTCCTCATCAAAATCCTCATCAAGATCAAGATTTTGAACCACTCCAGAGGTAAGCATCCTGTTTCTTTCCCACAGCTCATTATCTTTGTTAATCTGACGCTGCTGGGCTGACATCCGTTTCTTTTTTGAACGTTCTAATTGACGTTCTCGCTTAGTTATGTACTCTTGGCTGACAGAGCCGAAAGGGTCTCTACCCTCATCTATACCCTGATCAAGTCCATACCATTCTCTGTCCAACCTCTTTTGCTCCTCTTCCCAAAGTAGGCGATCTTTCTCCGAACCCCATGGAACATCATCCTCTTTTCCAGCTAAAGGTGTAGCTCCTGTTCGTGATCTTTCTGGTGCCCAACTGTTGAATCTATGTGCTGGGGTAGGTCTTCTCATGGACCAGTCATCTTCCCTAGGCTCTGTCTTCGGTGTGGGGTGTTCCCATGAAGACTTTGCAGCGGGTGTAGAAACTTCATCTTCTTCCCAACCAGTTCTAGATGGAGTATCTTTAACAGAAAACATAGGTGTCAGTGGTTCATCTTTGAACCTTGGAGATTCTGACCAAGACCTCTGAGACCTGTTATAGCTGTGTTCGCTTCTGCGGTCATTTCGATCTTCATTCCGTCTATCACTTCTCCTGTCACGATCTCTGTTTCCTCTACTACGATCTCTATCTCTATCTCTATCCCTGTGTCTATATCTTTCCCTATCTTTCTCTCTTCTGTAGTCTTTTCTGTAGGTGTCATCATTTTCATTATGTTCTTTTATATCTGCTGTAACATGAATTCCTCTTTTCTTTTCTTCTTTCCTTCGCCTTTCATCCATTCTTTCTTTGGCCTCTCTAGTAACACCTCCTGTATGAGTGGGCGTTTCGTCATTTTTTTCTCTGTAATGCCTGTCGCTGTGTTTATCAGGTTTCACAAAACTAGTTTTATTTTCTTCTGATTCTTTTACTTTCTTTGATGTCTCTTTTTTTTCATCATTTTCTCTTTCTTTCCTACGAAGTGCTGCTAGCCTGTCCAAACCCAGCAATGAAGTTTGTGGGACTTTAAACTGGAATGCCCCTTTAGGTTCTGGTTTCTTTTTAATTATAAGACCACCTTGTTTTTCTTCAACTCCTTCAAGTCTATAAACACCCTCATCATCTTCAACTTCCATGACAAAATATACTAAGAGATAATTGTTTTTTCTTCTAACACGTATCAAATATATATTTCAGCTGCAACAAATCATACAAATAGATGTAAACAACAAAATATTTTGAAAACTTCACCATACACGAAGGTTAAAAACGGTTACAAAATGTAAGATAGAAAGGAAATTACAGAGAGGTACTGATTTGAAATATGGCTAGGACTGTAAGCCGCAAACGGTGAAGACTATACGTGCGTGTTGTTCAGTATTGAAAGAAAATATCACAAGTGTAGAGATGAAATGAATTTAAAGTAAACTTTCCAGTGGCTATGTGGGACCTAGAGCACTGTGATCCAAAAAAGTGTACAGGTCGCAAACTTGCTAGAAAAGGTATGATCAAAACTCTGAGGCTTCAACAAAGATTTAATGGAATAGTTCTTTCTCCTATGGGAATTAAGTGTATCTCTCCTTTGGATAAGGACATAGTTGAAAGATTTGGTGCTGGTGTTATTGACTGTTCTTGGGCAAAATTAGAAGAAACCCCTTTTGAGCAGATGAAAACTTCAAATCCTAGGATTTTGCCATTTTTGGTTGCTGCAAATCCAATTAATTATGGCAAGCCAATAAAATTGTCTTGTGTTGAGGCACTTGCAGCACTTTTCTATATAACAGGTTTTCAAGATGTAGCTGAACATTATTTATCCCAATTCACATGGGGAGATAGTTTTATAAATTTGAATCAAGAACTGTTGGACAGGTATGCAGCCTGTAAAGATAGTGAAAAGGTTGTTGAAGCCCAAAATGCTTATCTTGCTGAATGCGAAGAAGAGAGGAATGAAAGGCATTTGATTCCTGATTTTGAAAGTTCTTCTGAAAGTTCTGAAGAAGACAATGAGCCAACATGATTGATTGCTTGAGTTTTTTCAAGAGTTGTTGATTTGTTATACTCGAAAGAAGAGTTGTAATTTTAAGGATTGTAAATATATTTAATTTTGATTGAGA

Protein: RF -1: -3747 -> -136 (1203 aa)

Comparison with *Halyomorpha halys*, PREDICTED: pre-mRNA-splicing factor ATP-dependent RNA helicase PRP16 - Sequence ID: XP_014279344.1

E= 0.0; bits= 2423

Query 1 MEVEDDEGVYRLEGVEEKQGGLIIKKKPEPKGAFQFKVPQTSLLGLDRLAALRRKEREND 60

ME EDDEGVYRLEGVE+KQGGLIIKKKPEP+G+FQFKVPQTSLLGLDRLAALRRKEREND

Sbjct 1 MEAEDDEGVYRLEGVEQKQGGLIIKKKPEPQGSFQFKVPQTSLLGLDRLAALRRKEREND 60

Query 61 EKKETSKKVKESEENKTSFVKPDKHSDRHYREKNDETPTHTGGVTREAKERMDERRRKEE 120

EKKE SKK+KE +ENK SFVKPDKH DRHYREK DETPTHTGGVTREAKERM+ERRRKEE

Sbjct 61 EKKEASKKLKEEQENKASFVKPDKHGDRHYREKYDETPTHTGGVTREAKERMEERRRKEE 120

Query 121 KKRGIHVTADIKEHNENDDTYRKDYRREKD------RERYRHRDRDRDRDRSRGNRDRDR 174

KKRGIHVT+DIKEHNEN+D+Y KD+RR++D R R R RDRDRDR+R RG+RDRDR

Sbjct 121 KKRGIHVTSDIKEHNENNDSYIKDFRRDRDKGNDRHRNRQRERDRDRDRERRRGDRDRDR 180

Query 175 RSDRRNEDRNDRRSEHSYNRSQRSWSESPRFKDEPLTPMFSVKDTPSRTGWEEDEVSTPA 234

RS+RRN+DRNDRRS+ SYNRSQRSWSESPRFKDEPLTPMFSVKDTPSRTGWEEDEVSTPA

Sbjct 181 RSERRNDDRNDRRSDRSYNRSQRSWSESPRFKDEPLTPMFSVKDTPSRTGWEEDEVSTPA 240

Query 235 AKSSWEHPTPKTEPREDDWSMRRPTPAHRFNSWAPERSRTGATPLAGKEDDVPWGSEKDR 294

AKSSWEHPTPKTEPREDDWSMRRPTP HRFNSWAPERSRTGATPLAGKE++VPWGSEKDR

Sbjct 241 AKSSWEHPTPKTEPREDDWSMRRPTPGHRFNSWAPERSRTGATPLAGKEEEVPWGSEKDR 300

Query 295 LLWEEEQKRLDREWYGLDQGIDEGRDPFGSVSQEYITKRERQLERSKKKRMSAQQRQINK 354

LLWEEEQKRLDREWYGLDQGI+EGRDPFGSVSQEYITKRERQLERSKKKRMSAQQRQINK

Sbjct 301 LLWEEEQKRLDREWYGLDQGIEEGRDPFGSVSQEYITKRERQLERSKKKRMSAQQRQINK 360

Query 355 DNELWERNRMLTSGVVQNLDLDEDFDEEAEARVHLLVHNIVPPFLDGRIVFTKQPEPVIP 414

DNELWERNRMLTSGVVQNLDLDEDFDEEAEARVHLLVHNIVPPFLDGRIVFTKQPEPVIP

Sbjct 361 DNELWERNRMLTSGVVQNLDLDEDFDEEAEARVHLLVHNIVPPFLDGRIVFTKQPEPVIP 420

Query 415 VKDPTSDMALVSRKGSALVRAYREQKERKRAQKKHWELSGTTIGNIMGVPKKKDKEEDKI 474

VKDPTSDMALVSRKGSALVRAYREQKERKRAQKKHWELSGTTIGNIMGVPKKKDKE+DKI

Sbjct 421 VKDPTSDMALVSRKGSALVRAYREQKERKRAQKKHWELSGTTIGNIMGVPKKKDKEDDKI 480

Query 475 DSEVEADYKTSQKFAEHMKDMAPASSEFAKKKTIQQQRQYLPVFAVRQELLNIIRENSVV 534

D + EADYKTSQKFAEHMKDMAPASSEFA+KKTIQQQRQYLPVFAVR+ELLNIIRENSVV

Sbjct 481 DGDAEADYKTSQKFAEHMKDMAPASSEFARKKTIQQQRQYLPVFAVREELLNIIRENSVV 540

Query 535 VVVGETGSGKTTQLTQYLHEDGYSKSGMIGCTQPRRVAAMSVAKRVSDEMASPLGKTVGY 594

VVVGETGSGKTTQLTQYLHEDGYSKSGMIGCTQPRRVAAMSVAKRVSDEMASPLGKTVGY

Sbjct 541 VVVGETGSGKTTQLTQYLHEDGYSKSGMIGCTQPRRVAAMSVAKRVSDEMASPLGKTVGY 600

Query 595 AIRFEDCTSEDTVIKYMTDGILLRESLREPDLDNYSAIIMDEAHERSLNTDVLFGLLRQV 654

AIRFEDCTSEDTVIKYMTDGILLRESLREPDLDNYSAIIMDEAHERSLNTDVLFGLLRQV

Sbjct 601 AIRFEDCTSEDTVIKYMTDGILLRESLREPDLDNYSAIIMDEAHERSLNTDVLFGLLRQV 660

Query 655 VARRQDLKLIVTSATMDSSKFSMFFGHVPVYTIPGRTFPVEVLFARNAVEDYVEASVKQA 714

VARRQDLKLIVTSATMDSSKFSMFFGHVPVYTIPGRTFPVEVLFARNAVEDYVEASVKQA

Sbjct 661 VARRQDLKLIVTSATMDSSKFSMFFGHVPVYTIPGRTFPVEVLFARNAVEDYVEASVKQA 720

Query 715 LQIHLQPTKGDILIFMPGQEDIEVTCEVLGERLAEIDAAPPLSILPIYSQLPSDLQAKIF 774

LQIHLQPTKGDILIFMPGQEDIEVTCEVLGERLAEIDAAPPLSILPIYSQLPSDLQAKIF

Sbjct 721 LQIHLQPTKGDILIFMPGQEDIEVTCEVLGERLAEIDAAPPLSILPIYSQLPSDLQAKIF 780

Query 775 QASPDGVRKCVVATNIAETSLTVDGIMYVVDSGYCKLKVYNPRIGMDALQIYPISQANAN 834

QASPDGVRKCVVATNIAETSLTVDGIMYVVDSGYCKLKVYNPRIGMDALQIYPISQANAN

Sbjct 781 QASPDGVRKCVVATNIAETSLTVDGIMYVVDSGYCKLKVYNPRIGMDALQIYPISQANAN 840

Query 835 QRSGRAGRTGPGHAYRLYTERQYKDELLTSTVPEIQRTNLANTVLLLKSLGVQDLLQFHF 894

QRSGRAGRTGPGHAYRLYTERQYKDELLTSTVPEIQRTNLANTVLLLKSLGVQDLLQFHF

Sbjct 841 QRSGRAGRTGPGHAYRLYTERQYKDELLTSTVPEIQRTNLANTVLLLKSLGVQDLLQFHF 900

Query 895 MDPPPQDNILNSLYQLWILGALDHTGSLTPLGRQMAEFPLDPPQCQMLIVSNQMGCTAEI 954

MDPPPQDNILNSLYQLWILGALDHTGSLTPLGRQMAEFPLDPPQCQMLIVSN+MGCTAEI

Sbjct 901 MDPPPQDNILNSLYQLWILGALDHTGSLTPLGRQMAEFPLDPPQCQMLIVSNEMGCTAEI 960

Query 955 LIIVSMLSVPTIFYRPKGREDEADSVREKFQVPESDHLTYLNVYNQWKQNKYSSTWCNDH 1014

LIIVSMLSVPTIFYRPKGREDEADSVREKFQVPESDHLTYLNVYNQWKQNKYSSTWCNDH

Sbjct 961 LIIVSMLSVPTIFYRPKGREDEADSVREKFQVPESDHLTYLNVYNQWKQNKYSSTWCNDH 1020

Query 1015 FIHAKAMRKVREVRQQLKDILDQQKMEVVSCGTDWDIVRKCICSAYFHQGARLKGIGEYV 1074

FIHAKAMRKVREVRQQLKDILDQQKMEVVSCGTDWDIVRKCICSAYFHQGARLKGIGEYV

Sbjct 1021 FIHAKAMRKVREVRQQLKDILDQQKMEVVSCGTDWDIVRKCICSAYFHQGARLKGIGEYV 1080

Query 1075 HLRTGMPCHLHPTSALFGMGFTPDYVVYHELVMTSKEYMQCVTAVDGVWLAELGPMFFSI 1134

HLRTGMPCHLHPTSALFGMGFTPDYVVYHELVMTSKEYMQCVTAVDGVWLAELGPMFFSI

Sbjct 1081 HLRTGMPCHLHPTSALFGMGFTPDYVVYHELVMTSKEYMQCVTAVDGVWLAELGPMFFSI 1140

Query 1135 KETGRSGSSKRREAMEGMEAEMEAAQKQMKERERQAEEERGRWSKRSSQVAEPGAPGSTP 1194

KETGRSGSSKRREAMEGMEAEME AQKQMKERERQAEEERGRWSKRSSQVAEPGAPGSTP

Sbjct 1141 KETGRSGSSKRREAMEGMEAEMEIAQKQMKERERQAEEERGRWSKRSSQVAEPGAPGSTP 1200

Query 1195 LRTPLRLGL 1203

LRTPLRLGL

Sbjct 1201 LRTPLRLGL 1209

Graphical representation


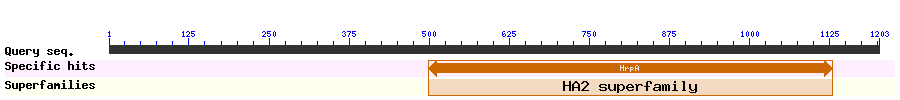


**Clip 1**

>TRINITY_DN28382_c0_g1_i10 length= 6180 nt

TCTGTATTGACAATGATAATCAAGAAAGCAGTAACTAAATAACTTGAAAACATATTATAAATATGTAGTTCGCATTGCTTTAAAACTAATAGTATGTTACTCGGTCAAGCTTTAAGAAATATAATTTTATAATTATATAATTTTTGTATTAATACTGAAATGAACATTTACCCTCACTATTCACGAAATGGTAACGACAAATCTCTACACCTATTTACTTTTAAAAAAATATAATAATGAAATTTCAAAGAAAAAACAAAATAAGAGAAAAATGATAATTGTGTGTGGTCATTATAACACAATAAAAGAGTATCAATTTATTGTGTTATAATAACTGCACACCCCGATTATCTTCTTCCCTATACAATTACGGTCAACCAAATAAAGGTTTCACCAGTAAAAATTAAATTAGTTCATTGTTAATCTGATCCAAGTTGAGGACCAATTTTTTGTTTCGGCGAAACTAGGCCTAAAAAATTTAAATAAATAAAAAATAAACGAAAAATATTTTAAAATTATTAACTTTTCTTAAAGGATACTCTTACATCATAATATATTTAGTCTCAATCCATTAAATCACACAACGCGTAAAGAAATTAAAATAAAAGATTACAATAAACCATAGGAATTATCACAGTCATTAAAAAACCTTTGCATCAATAAATCTAATCATAAAAAATTGACTACAGCCACTGCTTTAAAAATATATATGAATATAAACTCTATATGTTTTAAAGGTTGTTACGCTTATGTTATTGGTGCGTCATCATCTTGCAGAGATGTCACAAATACTGTATTATAGCGCCTCACACGGAATTGGTACAATTTTTCAATTAATTTGAGGCGTTTAATTAAAAAGAAAATAGCACCAGCTAGAGCTAATATCATACAAGGTACAACAATCCAGAGAGCAATAGAGCTTCCTCCACTAACAACTGGATGAGTATCAACAGAGTCACTTGTAGATGGAACAAAAATACGGACACATTTTCCATTTTCAGATTTAAACAATTCTTTGCATTTACAAACACCTACATTATCCTTTTCTTGAAGGCATTGGGAGTTTTCCCCACAATTATTATTAAAAACATCATCCAGCTTGCATGATTCTTATTAAATTAATACTTATGTAAACTACACAATAACGAAGTTAATGAATTTGTAGATTAGAAGTCGGTATCCATGTAGGTAACATCTGCCATATTCGTTGGGTTTATCCAGATGAACTATTGTATTTGGTATTAACAGTTTACTTTCTGAGAATGTTAAACAATTTACGTGTGTTTTGTCAATTATTTTAATAATAACTCAAAGTGTTTTCATAACCATACGTAGTAAAATACGGCTTGAATTTATAAAAATAAACTACCTTCTCTTGTCTACCTTTTAATTATTTATTGCTTCAATTGTAAAAGCTAGAATATCTCTCCATTTATGTGGCTACATGAAATCTTTGATGTATTTTCTATTGCTTGATGAAATAGAGACATGTTTTCTTCAATTAGAAGTGAATCTATAGTTTTTGTATCACCAGAGACATCACTACCTAGCTATTCCAACAAGAATGTATTGTGTTATTTTGAAATAGTGTAGTGTCCTTTTAAAAATTTCTTATATATTGTTTTAACGAATTCGAAAGTATTCATGCAGATGTGATAGAACGAACATTGATGGAACAAAATGTCAGCAAAAGATATGGATGGTTTTATGGGTCTTCTGTCCACAACAGATACAAAACAAAAGCTCGTTATTGGGCAGGATCTTTTGAACTATTTGCAGAATGGATCATCCATAGAATGTTCTGATATTGGCATGGTAGTTGACAGCCTTCTGCCATGGATACAAAGCAGCAATTTCAAGGTGTCGCAAATGGGGCTGGACCTTATGACAGAACTAGTACGAAGGATGGAAGGCTATTTCAAACCCTATATTCCAACTGTTATACCTCCTGTTGTCGACCGACTAGGTGATAGTAAGGAATTAGTTCGTGATCGCTGTCAGCTTCTATTGAGTACATTGATGGAAGTGGGCGCAATCACTCCTCAACATCTTTGGGATCGATTGGCTCCTGCATTCGCTCATAAGAATTCAAACGTTAGAGATGAAATTATGAAATGTCTGACTACAACGCTAAACCAGCATGGGGCAAATACTGTCTGTGTTTCCCGTTTGGTGCCATCATTAGTTAAGCTGTTATCTGATCCCAACGCCGCCGTAAGGGACAACGCACTCGGCACATTAGCCAATGTTTACAGGCATGTTGGAGAACGATTTAGGGCAGACCTAATCAAGAAACATCAGTTACCTCCTACCAAAGCTCCAAACCTAATGGCAAAACTGGACGAAATCAAAGCAGCTGGAGATTTCTTCCCTACTGCACATCTTCCTACTAGTTTGCTGGATGAAGATGAGACTGATAGGAGCGCGCCATCGTCAGCTACAAAACGATCCGCCAGTGTTACTCGGAAAAATATCACCTCCTCCGCTTCAAAGCCTACTTCAGTGCCAGCCCCCTCTTCTTGTACTGCTACTCCGAGTTCAGCATCGAAATTCACCCGGACTGGTTCCTTGAGGAAACCGAGTGCAGGAACGGGTCCCGCTGGTGCAGTCGACGAAGATGGTTTCATTAAGGCGTTCCACGATGTACCGAATGTTCAAATATTTAGTGTTCGAGATTTGGAGGATACACTTAATAAGGTTCGAACCATCATAGCAGATCCGAACCAAGAATGGAATAAGCGTGTGGATGCAATAAAGAAGATACGCTCACTCCTGATAGCGGGTGCTAGCGGTTACGAAGAATTTTATAATCATTTACGATTACTCGATATTCCATTCCAAATATCAGTCAAAGAATTAAGGTCTCAGGTGGTGAGGGAAGCATGTGTTACTTTAGCGTTTATGTCTCAACACCTCACAAACAGGTTTGAACATTTTGCTGAAAATCTTGTACCAGTCTTGATGAATTTGATACAAAATTCAGCTAAGGTGATTGCTAGCGCTGGCCAAGTATGCATCGGCTTCATCGTCCGCTATACTCATTCTAATCGATTAGTACCACACATTTGCTCAGGATTGTCATCAAAATCTCGTGAGATAAGGAGAGCAGCTTGTAAGTTCCTTCATATAATTCTTTCTACCTGGTCTGGTCACATTCTTACGAGGCACTCCGCCTTACTACAAAGTGCTATAGCCTCGGCAATCGCAGATGCTGATCAAGAAGTTCGTGGAACTGCTAGGAAAGCTTACTGGGCATTTAAAGATCAGTTTCCGCATCTTGCTGAAAGTCTCCTAGAGAACTTAGATGCTACTTATAAACGAAGCCTCCACTCCGACATGTCCAATTCAAGTTCTTCTAATTCTCTTCATCATCAGCCAACCTCCACCACAAGACGACAAGAAGCAGCTACACCTCTTTCTAAGAGGATTCCTGTAGGATCTCCGAGAACAGCCTCGCCACGTTCTAATTCAGCTATAGATCTCCAAGCAGCTCAGCGAGCAAAAGCCAGAGCACAATACGCAGCTTTAGCTAGACAAAAAGTCGGATCGAACACTAGTCTTCCTCGTCCCAAAAAAACAGGTACCGAATCTGGACCAAGTTCTTTAGCATTTTCTCCTGAACATTCGAGGCAAGGAAGGAGTAGAACGACAGGAATCAGCCAGTCACAGCCTTCAAGCAGGTCAGGATCACCCTCATCACGGTTGAACTACGCAACTTATTCATCTTGTTTGGGAGAGCGTTCCAGGAGGTCCCCGTCGTATACTACACGCTCAACTCAAGGCTCTCGAGAAACTTCACCTTCTAGATACAACCCGCTTCCTCCACTAGGGAAACTGAGGGGTGTTCGCCCTCCTCTTTCAAGGCCTGTGATGGCGCATAAAATTTTACAGCAGTCCCTTGAGGCTGAATCAGCCTTAGCAGATGCTTTAGGCTACACCGATGGACGAGGTGATACCAGTGGTAGCTGTGTTACATCCCCTCGTAGGTTGAGATTAGACGATCATTCTGATGATTCTGAAACATCCAGTGTTTGTTCCGAACGATCTTTCGAATCTCATCGGAGACCTTCTGATTCCTACTCATGGAGTGGTTCGCAACAAAGACTCTACCGAGATATGTGGGAACCAGCCATTAAGGATATAAATGAAATTATGGAAGCGTGCGAGTCGACACATTGGTCCGATAGGAAGGATGGTTTAGTATCTCTATCTGCCTATCTTCAAGCTGGTAATACTTTATCGCCTCATCAGCTCAATAGAATCACTGAAAATTTTACAAAAATGCTCTCTGATACACATACTAAGGTTTTCTCGCTTTTTCTTGATACTGTATCCGACCTTGTTTCAACACACTCTGCTGATCTCCATCCATGGCTTTATATTTTATTAACTAGGCTTTTTAATAAACTCGGAGGAGATCTGTTATCTTCCATACAGACTAAGATTCAGAAGACTCTAGGAGTTGTTAAGTCAAGTTTTGGACCGGAACCACTTTTACATTGGGCACTTAGATTTCTGGTAGATCCAACCCAAACTCCGAACACACGAGTGAAATTGGCTGTATTAGGTTTTATTGCTAAACTAGCACCTGCTGCTGATCCATCTTCTGCTTTCCCGCCTGCTTCTCCTTCTTCATCAGGACAATCAAAAGATGTAACAACGACTGCTCTGACTAAAATGATAGGTTGGACCATGGGAGAAGGAATCAAACAAGGAGGAGAACTGAGGCGTGGAGCACAAGAAGCCATTTTAGCGCTTTTTAATTTGAACACTCCGCAAGTAACCTTAAGGTTATCCCAGCTACCCAAAGAGTATCAGGAAGCTGCCGGAAGCCTGATTCGTATGAGACGTGGCAGTGGAGGTGGAGGTGACTCGTTGAGCCCTGATCAGGTTTATCGCTCTCTTCGTCGCACTACAGCTGAAATACAAAGTTACTCTTACGATACCAATGGAGGAAACAAAGTTGCGGATACGGCTTCACATGACTCGGGCATCAGTCAAATGTCGGATAAACATGACGACATTCTTTGGGGGATGAGTTCTCTTTCGTTATCTTCACCTACAAGATCCGGCAAAGACTACAATGGCCTAGAAACTACTGATTCACCAACCTCTCTGGGTAACGGTTTTTCCAAAGAGGGCGGAAGTAGTGAAGCTGGAGATGCCTTGGGTAAACTACTAGATACTATAGAAAACAGCAGTGGAGCAGAAAGGAAAGCTGCTTTCGACCAGCTTGCTACGCTTATCAGAGAATCGGATCCTATCACATTAAATGCTCATTTTAGAAAGATCCTCAAGGGTGTCCTCGGAAAATCTGGCCTTTTAGGAGAAGCAGATTCGAGATTAGGTGGACTCAGCGCGCTGTCGGAGGTGATGAAGAGGAAATGTCTTTGGTCCTCTCTCCAAACATATGTCGAGCTCATTCTTTTACGTGTGATAGATGCATATTCGGACGGGAATAAAGAAGTATCCAAACTCGCCGAGCAGGCCGCACTCCTCATAATTTCTGCATTAGATCCCGCAGATGTAATTAGGGTATTAGTACCATTAATGAGGGCAGAAGAATTTCCTAAAAATCTAATGGCCATTAAAACTCTGACAAAACTTGTTGAATTAGAAACAAAAGAAGTTATAATGCCATTTCTTCCAGACATTATGCCGGGACTTGTTCAGGCCTATGCTAACGAGGAGAGTTTAGTTCGAAAATCAACCGTATTTTGTATGGTCGCTCTTCATAACAAACTCGGAGGAGAGACGCTTAAGCCATATCTCGCATCTCTTAACGCAAGCAAATTAAAGCTTTTACATTTGTATATAAATAGGACTCAAACAGATCAGTCGCCGAGTTCTCCCAAGCCTGCTGCAGCCGTATAATGTGCCATCCCAATTATTTCGCTGATATATATTTTTATATTCATTGTAATTGCTTTGTAAAAAACCAAAATGGAACAAAAAAAAAAAATTAGAGAAGATACATTTAGTAATACTTATAACATAATAACACTTCTCATTACGTGTAGTCAGTTCTCAGTTGTACAGCTTATTATTATGCACATATTTAATTTGTATTTATATCTACTTTTTATTAATAAAAGTCTATTGTAACTACTGTCGACATGTATTTAATTAATTTTGTTTAAGTGAATTTAATATGAACAAAGTTGCATACATTATTTATTG

Protein: RF -1: -1681 -> -5874 (1397 aa)

Comparasion with *Halyomorpha halys*, PREDICTED: CLIP-associating protein - Sequence ID: XP_014275582.1

E: 0.0; bits= 2731

Query 1 MSAKDMDGFMGLLSTTDTKQKLVIGQDLLNYLQNGSSIECSDIGMVVDSLLPWIQSSNFK 60

MSAKDMDGFMGLLSTTDTKQKL+IGQDLLNYLQNGSSIECSDIGMVVDSLLPWIQSSNFK

Sbjct 1 MSAKDMDGFMGLLSTTDTKQKLIIGQDLLNYLQNGSSIECSDIGMVVDSLLPWIQSSNFK 60

Query 61 VSQMGLDLMTELVRRMEGYFKPYIPTVIPPVVDRLGDSKELVRDRCQLLLSTLMEVGAIT 120

VSQMGLDLMTELVRRMEGYFKPYIPTVIPPVVDRLGDSKELVRDRCQLLLSTLMEVGAIT

Sbjct 61 VSQMGLDLMTELVRRMEGYFKPYIPTVIPPVVDRLGDSKELVRDRCQLLLSTLMEVGAIT 120

Query 121 PQHLWDRLAPAFAHKNSNVRDEIMKCLTTTLNQHGANTVCVSRLVPSLVKLLSDPNAAVR 180

PQHLWDRLAPAFAHKNSNVRDEIMKCLTTTLNQHGANTVCVSRLVPSLVKLLSDPNAAVR

Sbjct 121 PQHLWDRLAPAFAHKNSNVRDEIMKCLTTTLNQHGANTVCVSRLVPSLVKLLSDPNAAVR 180

Query 181 DNALGTLANVYRHVGERFRADLIKKHQLPPTKAPNLMAKLDEIKAAGDFFPTAHLPTSLL 240

DNA GTLANVYRHVGER RADL KKHQLPPTKAPNLMAKLDEIKAAGDFFPTAH TS +

Sbjct 181 DNASGTLANVYRHVGERLRADLTKKHQLPPTKAPNLMAKLDEIKAAGDFFPTAHHATSEI 240

Query 241 DEDETDRSAPSSATKRSASVTRKNITSSASKPTS-VPAPSSCTATPSS----ASKFTRTG 295

P SATKRSASVTRKN++SSASKPTS +PAPSSCTATPSS ASKF+RTG

Sbjct 241 KC--LILFQPPSATKRSASVTRKNVSSSASKPTSGMPAPSSCTATPSSVPNSASKFSRTG 298

Query 296 SLRKPSA-GTGPAGAVDEDGFIKAFHDVPNVQIFSVRDLEDTLNKVRTIIADPNQEWNKR 354

SLRKPSA G+GPAGAVDEDGFIKAFHDVPNVQIFSVRDL+DTLNKVR +IADPNQEWNKR

Sbjct 299 SLRKPSAAGSGPAGAVDEDGFIKAFHDVPNVQIFSVRDLDDTLNKVRAVIADPNQEWNKR 358

Query 355 VDAIKKIRSLLIAGASGYEEFYNHLRLLDIPFQISVKELRSQVVREACVTLAFMSQHLTN 414

VDAIKKIRSLLIAGAS YEEFYNHLRLLD+PFQISVKELRSQVVREACVTLAFMSQHLTN

Sbjct 359 VDAIKKIRSLLIAGASSYEEFYNHLRLLDVPFQISVKELRSQVVREACVTLAFMSQHLTN 418

Query 415 RFEHFAENLVPVLMNLIQNSAKVIASAGQVCIGFIVRYTHSNRLVPHICSGLSSKSREIR 474

RFEHFAENLVPVL+NLIQNSAKVIASAGQVCIGFIVRYTHS+RLVPHICSGLSSKSREIR

Sbjct 419 RFEHFAENLVPVLINLIQNSAKVIASAGQVCIGFIVRYTHSSRLVPHICSGLSSKSREIR 478

Query 475 RAACKFLHIILSTWSGHILTRHSALLQSAIASAIADADQEVRGTARKAYWAFKDQFPHLA 534

RAACKFLHIILSTWSGHILT+HSALLQSAIASAIADADQEVRGTARKAYWAFKDQFP+LA

Sbjct 479 RAACKFLHIILSTWSGHILTKHSALLQSAIASAIADADQEVRGTARKAYWAFKDQFPNLA 538

Query 535 ESLLENLDATYKRSLHSDMSNSSSSNSLHHQPTSTTRR---------------------- 572

ESLL+NLDA YKRSLHSDMSNSSSSNSLHHQPT+ TRR

Sbjct 539 ESLLDNLDANYKRSLHSDMSNSSSSNSLHHQPTTATRRLGSTENLTNGRRSSSSSITTSR 598

Query 573 ---QEAATPLSKRIPVGSPRTASPRSNSAIDLQAAQRAKARAQYAALARQKVGSNTSLPR 629

EA TPLSKRIP+GSPRTASPRSNSAIDLQAAQRAKARAQYAALARQKVGSNTSLPR

Sbjct 599 RIESEAVTPLSKRIPIGSPRTASPRSNSAIDLQAAQRAKARAQYAALARQKVGSNTSLPR 658

Query 630 PKKTGTESGPSSLAFSPEHSRQGRSRTTGISQSQPSSRSGSPSSRLNYATYSSCLGERSR 689

PKKTGTESGPSSLAFSPEH+RQGRSRTTGISQSQPSSRSGSPSSRLNYATYSSCLGERSR

Sbjct 659 PKKTGTESGPSSLAFSPEHTRQGRSRTTGISQSQPSSRSGSPSSRLNYATYSSCLGERSR 718

Query 690 RSPSYTTRSTQGSRETSPSRYNPLPPLGKLRGVRPPLSRPVMAHKILQQSLEAESALADA 749

RSPSYTTRSTQGSRETSPSRYNPLPPLGKLRGVRPPLSRPVMAHKILQQSLEAESALADA

Sbjct 719 RSPSYTTRSTQGSRETSPSRYNPLPPLGKLRGVRPPLSRPVMAHKILQQSLEAESALADA 778

Query 750 LGYTDGRGDTSGSCVTSPRRLRLDDHSDDSETSSVCSERSFESHRRPSDSYSWSGSQQRL 809

LGYTDGRGDTSGSCVTSPRRLRLDDHSDDSETSSVCSERSFESHRRPSDSYSWSGSQQRL

Sbjct 779 LGYTDGRGDTSGSCVTSPRRLRLDDHSDDSETSSVCSERSFESHRRPSDSYSWSGSQQRL 838

Query 810 YRDMWEPAIKDINEIMEACESTHWSDRKDGLVSLSAYLQAGNTLSPHQLNRITENFTKML 869

YRDMWEPAIKDINEIMEACESTHWSDRKDGLVSLSAYLQAGNTLSPHQLNRITENF M

Sbjct 839 YRDMWEPAIKDINEIMEACESTHWSDRKDGLVSLSAYLQAGNTLSPHQLNRITENFKSMF 898

Query 870 SDTHTKVFSLFLDTVSDLVSTHSADLHPWLYILLTRLFNKLGGDLLSSIQTKIQKTLGVV 929

+VFSLFLDTVSDLVSTHSADLHPWLYILLTRLFNKLGGDLLSSIQTKIQKTLGVV

Sbjct 899 ILFFFQVFSLFLDTVSDLVSTHSADLHPWLYILLTRLFNKLGGDLLSSIQTKIQKTLGVV 958

Query 930 KSSFGPEPLLHWALRFLVDPTQTPNTRVKLAVLGFIAKLAPAADPSSAFPPASPSSSGQS 989

KSSFGPEPLLHWALRFLVDPTQTPNTRVKLAVLGFIAKLAPAADPSSAFPPASPS+SGQS

Sbjct 959 KSSFGPEPLLHWALRFLVDPTQTPNTRVKLAVLGFIAKLAPAADPSSAFPPASPSTSGQS 1018

Query 990 KDVTTTALTKMIGWTMGEGIKQGGELRRGAQEAILALFNLNTPQVTLRLSQLPKEYQEAA 1049

KDVTTTALTKMIGWTMGEGIKQGGELRRGAQEAILALFNLNTPQVTLRLSQLPKEYQEAA

Sbjct 1019 KDVTTTALTKMIGWTMGEGIKQGGELRRGAQEAILALFNLNTPQVTLRLSQLPKEYQEAA 1078

Query 1050 GSLIRMRRGSGGGGDSLSPDQVYRSLRRTTAEIQSYSYDTNGGNKVADTASHDSGISQMS 1109

GSLIRMRRGSGGGGDSLSPDQVYRSLRRTTAEIQSYSYDTNGGNKVADTASHDSGISQMS

Sbjct 1079 GSLIRMRRGSGGGGDSLSPDQVYRSLRRTTAEIQSYSYDTNGGNKVADTASHDSGISQMS 1138

Query 1110 DKHDDILWGMSSLSLSSPTRSGKDYNGLETTDSPTSLGNGFSKEGGSSEAGDALGKLLDT 1169

DKHDDILWGMSSLSLSSPTRSGKDYNGLETTDSPTSLGNGFSKEGGSSEAGDAL KLLDT

Sbjct 1139 DKHDDILWGMSSLSLSSPTRSGKDYNGLETTDSPTSLGNGFSKEGGSSEAGDALSKLLDT 1198

Query 1170 IENSSGAERKAAFDQLATLIRESDPITLNAHFRKILKGVLGKSGLLGEADSRLGGLSALS 1229

IENSSGAERKAAFDQLATLIRESDPITLN HFRKILKGVLGKSGLLGEADSRLGGL+ALS

Sbjct 1199 IENSSGAERKAAFDQLATLIRESDPITLNTHFRKILKGVLGKSGLLGEADSRLGGLTALS 1258

Query 1230 EVMKRKCLWSSLQTYVELILLRVIDAYSDGNKEVSKLAEQAALLIISALDPADVIRVLVP 1289

EVMKRKCLWSSLQTYVELILLRVIDAYSDGNKEVSKLAEQAALLIISALDPADVIRVLVP

Sbjct 1259 EVMKRKCLWSSLQTYVELILLRVIDAYSDGNKEVSKLAEQAALLIISALDPADVIRVLVP 1318

Query 1290 LMRAEEFPKNLMAIKTLTKLVELETKEVIMPFLPDIMPGLVQAYANEESLVRKSTVFCMV 1349

LMRAEEFPKNLMAIKTLTKLVE+ETKEVI+PFLPDIMPGLVQAYANEESLVRKSTVFCMV

Sbjct 1319 LMRAEEFPKNLMAIKTLTKLVEMETKEVILPFLPDIMPGLVQAYANEESLVRKSTVFCMV 1378

Query 1350 ALHNKLGGETLKPYLASLNASKLKLLHLYINRTQTDQSPSSPKPAAAV 1397

ALHNKLGGETLKPYLASLNASKLKLLHLYINRTQTDQSPSSPKPAAAV

Sbjct 1379 ALHNKLGGETLKPYLASLNASKLKLLHLYINRTQTDQSPSSPKPAAAV 1426

Graphical representation


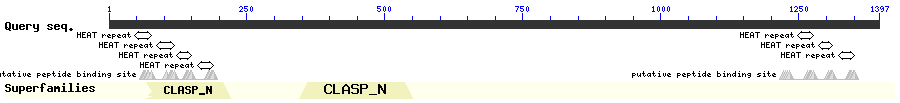


**Elp-1**

>TRINITY_DN28718_c0_g2_i2 length= 3876 nt

ATTATACCATTTAACAAGGAATATAACAAAATCTTTCCTTCTTATCATTACCTTTGCAATGATAGATTCAATTTCAGCATATACATTTATTACCTACTTAATATTTACATTAATTATTAGGCTCGAAAAAATATTTTTTATAAAACTTCTAAAATTTGAATCGAGTATCGCTATGACTCAAGTACATTAAATTTCCAATCTTGATTTATTTTGACTTCTGGTGGATACTTGAATTTTTCGTCTAAAGCAGATGTTTCATTTGTTCCAACTTCCTTTGGCCAAATAACGACTTTTAGTTTATCAATAAGGGACAGCCATTCATTCAGTTCTGTCTGAAGTTTTCCAGCTACTCCATCTTCCCTGAATACCAGCAATGTAGAACATGCTGAGTGAACTTCGCCAACATATTTTGGCGTCGAAGAAATAATACTACTTAATGTTGCAATAAGAGCTTCTTCTTCTCTTGGATTACCTTCTTTTAAGCTCCACATTTTTCGACTCATCTTTTTTGTGTTCTTGCTTGATCTCGATTGGAAAGATTTAGTGCGAACTGATGAAGTGCTGCTTGTATCAGAAGCTGAATCATTCATGTCATCAAAAATATATGCAGTTCGGTTTTCTTTCTCAATCCTAACTTTTACTAACCTCTCTACATATTTTCTTGTTTTATCAGTTTGAGTATGAATATCGTCCACTAGGTTAATAGCAGTATCGATTAATGAATCTCTAATAAGGCCATCAATCAAATCAGTCTTCTTAAACTTCTTAGCAGTATAGATGGCTTCTTTCCAACACCTCGCATTGATATACATTTCAACAGCTCTATCCGTATCTAACAACCATTCAGTATACAGTATTGCTGCTTCTGAATATTTGTGCGCAGATATTAACATATTGCAAACATTTTCAGCAATTTTCTTCTGTTCTTCTTCACTAAAATCTGAATCCATAACGATGACCATGCACTGCCTCCAGTTGCCAGATCTAGTAAACGATTGAAGAGCTTTTTGTTTCTCATCACTGCGGAGGTACATTATACCAGATTCCTCATAAAATCTTTCCCTGAATAGATATTCAGCATACTTAGAAGCAATGATTGAAGATCTCTTTGGATCATCACTAAATAATTTTAATGCGGTTGAATACAATGAATTTTCTTCTATAAATTTTAAACACTCTTCTTCATGGTCGTCGCAACATTTACTGAGGTGCTTCAAAGCAGATTCGAATCTCTTCAATTTTTTATCTATCACAAATCTCATGTAATTGGATTCCATGGATTTTAATGAATTCAAATATGGGATATACTCTTTGGGGTCTTTCTGCGATTTTCCAGCAATTTTCAAGGCAGCATTCAAATCGTAAACACCCAAAGCTTCCATATACAATTTGTTAGAATCAACAATAAACATTAAATGTTGCATTGCGACTTCATTGTTGGCAAGCTGAATCGCTTTACTCATATCGCCTATTTTAACTAAAGCACTGAGTATTGGAAAAATATATTTCTTCTTTAAATCCTCGATCGACATGACTGATAACAGAGCTTCGCAGATCGATTTGACTTTTGAACCATTCAGAAAGAAGGTATCCTTATTTTTGTAGTACTGCTTATATAAATTTTCTGTAACGTTTTCACCAGAAAGTTCGGTGATAAGAAGAGTGATCCATTGAGGGTCGATTTGGTTAACGAAATTATTAACTTCGTCCAAGAAAAGTTTCTGATTATGATCAATAATAAGATTGAGATCGATCCTCTGCTTTCTCATTAGAGAAAATGCAGATTTATACTCCTTTGAATCGATCAGTGTGCCGAGAGTTAGGATAGTGAGAGCTCGTGGCTGTATAGTTTCTAGATTCCCTCTCGGTGCCTGCACAACTACTGAATTATCGAATGTTGTAACGAGTCTGGAACCTCTTTCTAACTTTCTGTCGGATACTTCTACACACTCTGAATCATAGCACTGTAAAATCACTAATTTGTGCGAAGACGTTGACACTAATACATAAGGATCTTTCAAAACGAATGAGGTAACTTTGCAGGTTATAGAAGGCAAGTTGATAGGAGTGTTGTTAAAGTATAATCTCGATAATTCTGATAAGCAGTAAAGACCAGAAGAATGAACTTTGATGTCGACACAAGGTTCGATCAAGGATAAAGACTCAGTAATGATTAGAGACTCAATATTGTAGTTGAATAACTTTCCAGTAGTCGTTTGAATAACAAGTTTGTCTGATCTTCTGTTGCATGTTATAATATTTCCTGATAATTTTGAAACATTTCTCAATTCCAGACTGCCTTCAGAGAACAAGAATTGATGAATGTGGGTTTCACGTTTAACTGAAGCACAACAAATTAAAGTGCCTGTGTTAACCCAATCCCAATGAGATAATGAAACCATCTCCTCGGTGAAATTATACTTCTTAACCAAATCAGATAAAATCACAGGATTTGAGATTGGGTCCTTAAAAAACGTAATATCACCATTAAACAGAATTGCACATAGCATATAAGTCTGCTCCCCATCAATTGGAGCAAACATCACATGATTTACTGCATCTGGACACTTCAGTTTGTATGAAGACATCGGAGGAGGTATAACTGCTTCTTTGAATGACGTAAGCAAAATAGAATCATTATCAATAACACTAACATATGATTTATCTTCACATTCTGAATTGTAACTATTACTAACAGCAAACGTCCATTCTATTATCTGTAAAACTCCTCCTTCACATAGGGCATAAATTCTATTTGGTCGTTCTTTATCCCACCAAACATAACGAGCTGATGATACAAATTTGAACTTCTGTTTTATATACCAGTGATAATTTCCTGTGACCCATAACATTACTTCTTCACCAAGATTTTGTTGATTTTCACAAACCGTGCAAAGGATGGTTGATCCTTCATTCCAAGAGATGTGTTTGACTTTCATATCAGCTGGTAAAGTGAAGTCTCCATGTTTCAGACCATTCTTCTCCAGGAAAGAAACAACTTTTTTGTTTGGTAACTGCTGAGGAAGGGCTATCAAATTCCCACTTGGTCTCCAGCAGATAGGTTCATTCAAACCAGTTATATCTTCACTGGTGCTGAGTAATACTCCATCCATATTAAAAATCTTAACCCTCCTTAAGTTCATTTCAGAGCACCAATAACTAGCAGCGAATAGACTACTGTCCCCTCTCCAAGTTACACGAATCGAGTGATTGTCTTCAATAAACGACGGGTCGATTGTAGAAGGTTTGGACTTGGCTGCATTCTTCCCTTCAGATCCATGAAACTGTGTTTCCTTTTTACCCCAACCGACACTGATCAATTCATTTTCTCCACGAAAAGAATCATTTAGATCAATCTCAGCAAGTGTGAAGAATTCTGAATTCAATAAAATCAAAGAACCGCTAGCAGTTATTATTGCCGCAAGTTCTTCATCAGGGCTCCACTGAATATCACTAACGTTGCATTCTAATTCATGACTAATACTACAATGTTCAACTCCTTTATTGTCTAATATAAGAGAGTATATCACCCCATTTTCGAAACAAACATTAACTGAATTATACAAATCTCTATACCACATGCCGATTGGTTTTGGAACATTTTCATCTGGGAATAAACTTTCTAAATCATAATATTTTGATGTTTCTTCCGAGATCAAATCAACAGAATACAAAATATTCTTTGATATCACAAACACATTTTTAAGTTTCGCAGATAAATCATTGTCATGGGATGTTTGTCCGCAAACTATAAGAGTAGAGTCTTCGAACACATCACAATGTATTTTTAAGTTGAGTAAAAGCTTTAAATTCTTCATAGTTGTAAATATCAACAATAAAACGTGATAGAAGGTTTTACTCAGGTTTCATCTAGGC

Protein: RF -2: -3818 -> -171 (1215 aa)

Comparison with *Halyomorpha halys,* PREDICTED: elongator complex protein 1 - Sequence ID: XP_014290480.1

E= 0.0; bits= 2045

Query 12 IHCDVFEDSTLIVCGQTSHDNDLSAKLKNVFVISKNILYSVDLISEETSKYYDLESLFPD 71

+HCDV D++LIV GQTS DNDLS KLKNVFVISKN LYSVDLIS SKYYDL SLF D

Sbjct 12 VHCDVLADTSLIVGGQTSIDNDLSVKLKNVFVISKNTLYSVDLISGIASKYYDLASLFSD 71

Query 72 ENVPKPIGMWYRDLYNSVNVCFENGVIYSLILDNKGVEHCSISHELECNVSDIQWSPDEE 131

++VPK + MWYRDLYN++ +CFENG I+SLILDN+ VEH SI H+LEC DIQWSPDEE

Sbjct 72 KDVPKAMSMWYRDLYNTIYICFENGEIFSLILDNEKVEHSSIIHDLECKPLDIQWSPDEE 131

Query 132 LAAIITASGSLILLNSEFFTLAEIDLNDSFRGENELISVGWGKKETQFHGSEGKNAAKSK 191

AA++ + SLILLNSEFF LAEIDLNDS GENELISVGWGKKETQFHGSEGKNAAKSK

Sbjct 132 HAAVVCGNDSLILLNSEFFILAEIDLNDSLPGENELISVGWGKKETQFHGSEGKNAAKSK 191

Query 192 PSTIDPSFIEDNHSIRVTWRGDSSLFAASYWCSEMNLRRVKIFNMDGVLLSTSEDITGLN 251

PS+IDPSFIEDNHS+RVTWRGD +LFAASYWCS+ ++RRVKIFNMDGVLLSTSEDI GLN

Sbjct 192 PSSIDPSFIEDNHSLRVTWRGDGNLFAASYWCSKTSMRRVKIFNMDGVLLSTSEDIPGLN 251

Query 252 EPICWRPSGNLIALPQQLPNKKVVSFLEKNGLKHGDFTLPADMKVKHISWNEGSTILCTV 311

EPICWRPSGNLIALPQQLPNKKVVSFLEKNGLKHGDFTLP+D+KVKHISWNEGSTILCT+

Sbjct 252 EPICWRPSGNLIALPQQLPNKKVVSFLEKNGLKHGDFTLPSDVKVKHISWNEGSTILCTI 311

Query 312 CENQQNLGEEVMLWVTGNYHWYIKQKFKFVSSARYVWWDKERPNRIYALCEGGVLQIIEW 371

CEN + LGEEVMLWVTGNYHWYIKQKFKFVS R VWWDKERPNR+YALCEGG+LQI+EW

Sbjct 312 CENNEYLGEEVMLWVTGNYHWYIKQKFKFVSQTRNVWWDKERPNRMYALCEGGILQILEW 371

Query 372 TFAVSNSYNSECEDKSYVSVIDNDSILLTSFKEAVIPPPMSSYKLKCPDAVNHVMFAPID 431

TF V+NSY+S CEDKS+VSVIDNDSILLTSFKEAVIPPPMSSYKL CP AVNHVMFAP D

Sbjct 372 TFVVNNSYHSHCEDKSFVSVIDNDSILLTSFKEAVIPPPMSSYKLICPSAVNHVMFAPFD 431

Query 432 GEQT--YMLCAILFNGDITFFKDPISNPVILSDLVKKYNFTEEMVSLSHWDWVNTGTLIC 489

+Q +MLCA+LFNGD+ FFKDP+S+P ILSDLV+KYNF+EE++SLSHWDWV++ T I

Sbjct 432 KDQPSLHMLCAVLFNGDVAFFKDPLSSPTILSDLVQKYNFSEEIISLSHWDWVSSDTFIF 491

Query 490 CASVKRETHIHQFLFSEGSLELRNVSKLSGNIITCNRRSDKLVIQTTTGKLFNYNIESLI 549

C+SV RETHIHQF S+ LEL+NV+KL GNIITCNR +KLVIQTT GKLFNYN +SL+

Sbjct 492 CSSVNRETHIHQFSISKDGLELKNVTKLLGNIITCNRSGNKLVIQTTDGKLFNYNTKSLV 551

Query 550 ITESLSLIEPCVDIKVHSSGLYCLSELSRLYFNNTPINLPSITCKVTSFVLKDPYVLVST 609

+TES SLIEPC DIKVH SGL+CLSELSRLY NN PI+ PSITCKVTSF+LKDPY+LV+T

Sbjct 552 VTESFSLIEPCYDIKVHPSGLFCLSELSRLYLNNVPIDFPSITCKVTSFILKDPYLLVTT 611

Query 610 SSHKLVILQCYDSECVEVSDRKLERGSRLVTTFDNSVVVQAPRGNLETIQPRALTILTLG 669

SSHKLVILQC DSE VE+SDRKLERG RLVTTFDNSVV+QAPRGNLETIQPRALTILTLG

Sbjct 612 SSHKLVILQCCDSENVEISDRKLERGCRLVTTFDNSVVLQAPRGNLETIQPRALTILTLG 671

Query 670 TLIDSKEYKSAFSLMRKQRIDLNLIIDHNQKLFLDEVNNFVNQIDPQWITLLITELSGEN 729

+LIDSK++KSA SLMRKQRIDLNLIIDHNQKLFLDEVNNFVNQIDPQWITLLITELSGE+

Sbjct 672 SLIDSKQFKSALSLMRKQRIDLNLIIDHNQKLFLDEVNNFVNQIDPQWITLLITELSGED 731

Query 730 VTENLYKQYYKNKDTFFLNGSKVKSICEALLSVMSIEDLKKKYIFPILSALVKIGDMSKA 789

VT NLYKQYYK KD+ FLNGSKVKS+CEALLSVMSIEDLKKKY+FPILSALVK+G+MSKA

Sbjct 732 VTANLYKQYYKKKDSSFLNGSKVKSVCEALLSVMSIEDLKKKYMFPILSALVKLGEMSKA 791

Query 790 IQLANNEVAMQHLMFIVDSNKLYMEALGVYDLNAALKIAGKSQKDPKEYIPYLNSLKSME 849

IQLA+NEVAMQHLMFIVDSNKLYMEALGVY+LNAALKIA KSQKDPKEYIPYLNSLKSME

Sbjct 792 IQLASNEVAMQHLMFIVDSNKLYMEALGVYNLNAALKIAEKSQKDPKEYIPYLNSLKSME 851

Query 850 SNYMRFVIDKKLKRFESALKHLSKCCDDHEEECLKFIEENSLYSTALKLFSDDPKRSSII 909

NYMRFVIDKKLKR+ESAL H+SKCCD+HEEECLKFIE+NSLY ALK+F +D KRSSII

Sbjct 852 FNYMRFVIDKKLKRYESALTHISKCCDEHEEECLKFIEDNSLYPGALKIFKNDLKRSSII 911

Query 910 ASKYAEYLFRERFYEESGIMYLRSDEKQKALQSFTRSGNWRQCMVIVMDSDFSEEEQKKI 969

ASKYA YLF++R YEESGIMYLRSD+ +KALQSFTR+GNWRQCM+IVMDS+FS EEQ+K

Sbjct 912 ASKYANYLFKQRHYEESGIMYLRSDDTEKALQSFTRAGNWRQCMIIVMDSNFSNEEQRKY 971

Query 970 AENVCNMLISAHKYSEAAILYTEWLLDTDRAVEMYINARCWKEAIYTAKKFKKTDLIDGL 1029

AE+VCNMLISAHKYSEAAILYTEWLLDTD+AVEMY+ ARCWKEAIYTAKKF K+ LID +

Sbjct 972 AESVCNMLISAHKYSEAAILYTEWLLDTDKAVEMYVQARCWKEAIYTAKKFNKSSLIDTI 1031

Query 1030 IRDSLIDTAINLVDDIHTQTDKTRKYVERLVKVRIEKENRTAYIFDDMNDSASDTSSTSS 1089

++D++I+TA L +DIH+Q+DKT+KYVERL VRIEKENR YIFDDM+DSASDTSSTSS

Sbjct 1032 VKDAVIETANILAEDIHSQSDKTKKYVERLKNVRIEKENRATYIFDDMSDSASDTSSTSS 1091

Query 1090 VRTKSFQSRSSKNTKKMSRKMWSLKEGNPREEEALIATLSSIISSTPKYVGEVHSACSTL 1149

VRTKSFQSRSSKNTKKMSRKMWSLKEGNPREEEAL+ATLSS ISST KYVGEV+SACSTL

Sbjct 1092 VRTKSFQSRSSKNTKKMSRKMWSLKEGNPREEEALVATLSSSISSTEKYVGEVNSACSTL 1151

Query 1150 LVFREDGVAGKLQTELNEWLSLIDKLKVVIWPKEVGTNETSALDEKFKYPPEVKINQDWK 1209

LVFRED +A KLQ+ L EWLS+IDK K VIWPKE GTNE S LDEKFKYPPEV I QDWK

Sbjct 1152 LVFREDQLAAKLQSALAEWLSIIDKSKNVIWPKEAGTNEASVLDEKFKYPPEVNIKQDWK 1211

Query 1210 FNVLES 1215

FN L S

Sbjct 1212 FNFLMS 1217

Graphical representation


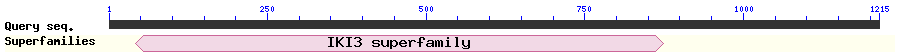


**GLD-1 homolog**

>TRINITY_DN27129_c0_g1_i23 length= 2518 nt

CTCCTGTGACCAATATTTTAGCAGAATAGTAGTTTTGCTAATGAAGGAGAACAACCATAAGGAAACAGCAAACACTACTCAATTCTCCTCTACAATCGGCACATAGCATTTGCTCTCAATATCATGCAGTCTCCTAATATAGTCAACTTCTTTATAATACTCATGGCAAGTGTTGCATACTACAATTATCCCTCCATCGATCCTAACCTCCGAGCTATAAATGTCCCGACACAAGATCTCTCTCAAGAATATGACTTTATCATTGTCGGCGCTGGAACTGCAGGCTGTGTACTAGCAAATAGGCTAACAGAAATCGCCAACTGGACTGTACTATTGTTAGAAGTTGGTGGTGAAGAGACCATCATAAGCGATACACCGCTATTGGCTGGGACTTTATGGAATTCACCCTTAAATTGGAATTATACAACAACGAGACAACCGAGGGCATGTCTGGCAACCGATGGGATTTGTCCTTGGCCAAGAGGAAGAGTAATAGGAGGAAGCAGTACACTCAATTTTATGGTATACGTTAGGGGCAATAGAAGAGATTATGATGGATGGGCTGCCCTCGGAAACACAGGTTGGTCTTACGATGAAGTTCTACCTTACTTCCGGCTATCAGAGGACAACACGAATCCAATATATGCAAATGACACAACTTACCATTCGACTGGAGGTTATCAGACTGTCTCAGATTTAAGTTATGAAACACCTCTAGTCAGTGGATATCTAGCAGGAGGAGAAGAGATTGGTTTTCAGGTTCGAGACTTAAATGCAGAATATCAAACTGGCTTCATGCCAGTTCAAGGAACGCTAAGAAATGGTTCGAGATGTTCAACAGGGAAAGCATTTTTAAGACCAGTTAGGAACCGACCTAACCTTAATGTTGCAGAAGGGTCATTCGTGACTAAAATAAATTTCATTGACAACAAAGCTGTTGGTCTAACATTCATAAGAAACAACCAAGAAATAAATGTGACAGCTAGAAAGGAAGTTATAATATCAGCAGGTTCAATTAATTCACCTCAGCTATTGATGCTTTCAGGCATTGGTCCTGCGGATGAATTAAATAAATTTGGAATACCAGTGATCAGCAATTTAAGTGTTGGGTACAATCTTCAAGATCACGTTGGTGCCCCACTTTTTTATCAAACCAATCCTCCAGTGGCGATTACTGCCTCATCTTATGAGAATATAGAAGCGATACAGGAATATTCTCAACCAAATGCTTCAGGTCCACTGACATCGCCAGTTGGTATTGAAACAATAGCTTTCCTCAATTCTACTTTTACAAATTCATCCATCGATTATCCAGACATTGAAATACACTTCACTTCTTTTTTCAATACTTTAGAAAACAATAACAGTATTTGGTTCGGTGTAGGGCTGGTAATCCATCCGCAGAGCAGGGGTAGAATAACATTACAATCAAACGACCCTAGTCAACATCCATTGATAAATCCCAATTATTTAAGTGAGCCACAGGACTTGCAAACATTAGTGCAAGCTCTAAAATATGTGTCAGCTGTTGCCAATACAACAGCGATGCAAAAGTATAATAACGTTTTTCAAGATGAATTATTCACCCTTTGTAATAATTATACTATTTTCACTGATGAATTTTACACCTGTGTCATAAAAACTTATACTACGACTATTTTTCATCCTATCGGAACTTGTAAGATGGGTCCAAGCAGTGATACAGAAGCTGTTGTAAATCCCAATCTTCAAGTCAATGGAGTCGAGAATTTGCGAGTCATTGATGCTTCTATCATGCCATTTGTAACAGGAGGTAATACAAATGCTCCGATAATAATGATAGCTGAGCGTGGTGCCGACATTATCAAAGCTTATTATGGTCAACCAACGCTGATCCCTCAAAATTAATCTTGAATAACTGCAACGGTATTATGGGTACCCTGCATATCTTAGAGGAGGTGGAGAATATTATAACAGATTATTATAAGCATGCTTCAAATTTGTATACTTTTGATATGTTAAATATTATTTACTAGAATAATGCATAGAATTTTTAAAAAAAGAAAAAAAAATTAATAAACATCTTGTTTAAGATTATTTCTTTAATATTTTAAAAGTCACTTTACACTCACAAAAAATAATTTGAGTGAGGCCAATAGAAATGATTGCCATAATATTGTTTTTTTAAATTTTATTTTGTTTTTTCTTTTGCAAACCAAGCAAAAATTTTACTCTCCTGTCAGGTAACAGGCAGCCGGAGTCAAATCTAAATGCACGAAACAACAGAAAAATTCTTCGTAACTTTCTAAATAAAAGATAATAAACATTTAAATTAAAGTAAGTACTGAGTATCACATCTGCGTGAATGGAAGAAGTAAATGAACTAGAATAAAAAAAAAAAATTATATAAATTATCAGTTCTGAATTTTTAGGTACCTTTGTGCTTTTTTTTTTTTTTTTTTTTTTTAAATCGCCTTAATTTTTCATTTTTTTAAAATTTAAGGATTAAGGGCCAAAAATTCAGGAAAAGGGAAAAAGG

Protein: RF -1: -124 -> -1887 (587 aa)

Comparison with *Halyomorpha halys* PREDICTED: glucose dehydrogenase [FAD, quinone]-like isoform X1 - Sequence ID: XP_014290348.1

E= 0.0; bits= 1073

Query 1 MQSPNIVNFFIILMASVAYYNYPSIDPNLRAINVPTQDLSQEYDFIIVGAGTAGCVLANR 60

MQ PNIVNFFIILMASVAYYNYPSIDPNLRAINVPT +LSQ+YDFIIVGAG+AGCVLANR

Sbjct 1 MQPPNIVNFFIILMASVAYYNYPSIDPNLRAINVPTPELSQQYDFIIVGAGSAGCVLANR 60

Query 61 LTEIANWTVLLLEVGGEETIISDTPLLAGTLWNSPLNWNYTTTRQPRACLATDGICPWPR 120

LTEI+NWTVLLLEVGGEETIISDTPL+A TLWNS LNWNYTTTRQ RACL TDGICPWPR

Sbjct 61 LTEISNWTVLLLEVGGEETIISDTPLMAVTLWNSSLNWNYTTTRQARACLVTDGICPWPR 120

Query 121 GRVIGGSSTLNFMVYVRGNRRDYDGWAALGNTGWSYDEVLPYFRLSEDNTNPIYANDTTY 180

GRVIGGSSTLNFM YVRGNRRDYDGWAALGN GWSY+EVLPYF LSEDN NP+YA DT Y

Sbjct 121 GRVIGGSSTLNFMAYVRGNRRDYDGWAALGNPGWSYEEVLPYFLLSEDNRNPLYAMDTIY 180

Query 181 HSTGGYQTVSDLSYETPLVSGYLAGGEEIGFQVRDLNAEYQTGFMPVQGTLRNGSRCSTG 240

HSTGGYQTVSDLSY+T LVSGYLA GEEIGFQ+RD+NAEYQTGFMPVQGTLRNGSRCSTG

Sbjct 181 HSTGGYQTVSDLSYQTSLVSGYLAAGEEIGFQIRDINAEYQTGFMPVQGTLRNGSRCSTG 240

Query 241 KAFLRPVRNRPNLNVAEGSFVTKINFIDNKAVGLTFIRNNQEINVTARKEVIISAGSINS 300

KAFLRPVRNR NL VAEGSFVTKINF++NKAVG+TF+RNNQ+INVTA+KEVIISAGSINS

Sbjct 241 KAFLRPVRNRTNLYVAEGSFVTKINFMNNKAVGVTFVRNNQQINVTAKKEVIISAGSINS 300

Query 301 PQLLMLSGIGPADELNKFGIPVISNLSVGYNLQDHVGAPLFYQTNPPVAITASSYENIEA 360

QLLMLSG+GPA+ELN+FGIPVI NLSVGYNLQDHVGAPLFY+TNP VAITASSYENIEA

Sbjct 301 AQLLMLSGVGPANELNRFGIPVIKNLSVGYNLQDHVGAPLFYKTNPSVAITASSYENIEA 360

Query 361 IQEYSQPNASGPLTSPVGIETIAFLNSTFTNSSIDYPDIEIHFTSFFNTLENNNSIWFGV 420

I EYSQPNA+GPLTSPVGIETIAFLNST NSSIDYPDIEIHFTS+ + LENN+SIWFGV

Sbjct 361 ILEYSQPNATGPLTSPVGIETIAFLNSTLANSSIDYPDIEIHFTSYVSYLENNDSIWFGV 420

Query 421 GLVIHPQSRGRITLQSNDPSQHPLINPNYLSEPQDLQTLVQALKYVSAVANTTAMQKYNN 480

GLVIHPQS GRITLQS DP QHPLI+PNY SEPQDLQTL+++LKYVS +AN+TAMQKYN+

Sbjct 421 GLVIHPQSSGRITLQSTDPYQHPLIDPNYFSEPQDLQTLMESLKYVSLIANSTAMQKYNS 480

Query 481 VFQDELFTLCNNYTIFTDEFYTCVIKTYTTTIFHPIGTCKMGPSSDTEAVVNPNLQVNGV 540

VFQD+ FTLCNNYT ++DEFY CVIKTYTTTIFHP+GTCKMGPS+DTEAVVNPNLQV+G+

Sbjct 481 VFQDQFFTLCNNYTTYSDEFYNCVIKTYTTTIFHPVGTCKMGPSTDTEAVVNPNLQVHGI 540

Query 541 ENLRVIDASIMPFVTGGNTNAPIIMIAERGADIIKAYYGQPTLIPQ 586

ENLRVIDASIMPFVTGGNTNAP+IMIAE GADIIKAYY QPT IPQ

Sbjct 541 ENLRVIDASIMPFVTGGNTNAPVIMIAEHGADIIKAYYNQPTQIPQ 586

Graphical representation


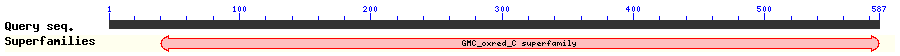


**ACO-1 homolog**

>TRINITY_DN28221_c0_g1_i1 length= 2961 nt

CTGTGATAGTGAGGTGTTACCTTCTCCAGTCCTTATCAAGCTTTCTCCCAGTCGAAATTGTTCTTTAGCGGTCTTGTATTTTATTCTACATTTTTCTACCATGTCAGGTCCGAACCCATATAATAAAATTCTAAAAAACTTGGATGTTGCTGGTGTGTCCTACAAATACTATAATCTTCCTGAATTAGGACCTCAATATGCGAAACTTCCATTCTCAATTAGAGTACTTCTGGAATCGGCAGTCAGAAACTGCGATAACTTTGAAGTTAAGCAAACTGATGTGGAGAACATTTTAAATTGGGAAAAAACTGGAAAAGATAGTAGCTCAGTAGAAGTGGCTTTCAAACCAGCTAGGGTTTTGCTACAGGATCTGACTGGTGTGCCAGCCGTTGTTGACTTCGCTGCTATGCGTGATGCTGTTAAAGCACTTGGTGGCAATCCAGATAAAATTAATCCCATTTGTCCTTCTGATCTTGTTATAGATCATTCTGTACAGGTCGACTTTGCCCGAGTTGATAATGCCCTACAGAAAAATGAAGAAATTGAATTTGAAAGAAATAAGGAGAGATTCACATTTCTTAAGTGGGGAGCTAAAGCTTTCCGAAACATGCTCATCGTTCCTCCCGGTTCAGGGATTGTCCATCAGGTGAATCTTGAATACTTAGCGAGAGTAGTTTTCACTGATGAGTTGCTTTACCCTGACAGTGTTGTTGGTACGGACTCTCATACAACTATGATTAATGGTCTCGGTGTCGTCGGATGGGGAGTGGGAGGTATCGAAGCAGAAGCTGTTATGCTTGGTCAAGCTATAAGTATGTTGATCCCTCAGGTCATTGGATATAAATTGGTCGGAACACTTAATCAGTATGCCACATCAACTGATCTGGTCCTTACTATCACCAAGAATTTGAGACAGTTGGGAGTTGTAGGCAAGTTTGTCGAATTCTTCGGACCAGGAGTTGCTGAATTATCTATTGCCGATAGAGCTACCATCTCCAATATGTGCCCAGAATATGGTGCCACCGTCGGCTTTTTCCCAGTGGATGAGAACAGCCTCTACTATCTGTCTCAGACTAATCGAGATCCAGCTAAAATAGAAGCCATCAGAAATTATCTGAAAACTGTTGGAATGCTGAGAGATTACGCGAATGCTGCACAGGACCCAGTCTATTCCGAGGTTGTGGAACTCGACCTGTCTACAGTGGTCAGTTGTGTTTCTGGACCAAAGAGGCCACAGGATCGGGCTTCAGTAACTGAAGTTAAATCATTATTTACACAGGCTCTGACCAATAAGGTGGGTTTCCAAGGATTCGGATTGAAGCCATCAGCTGTGTCAGCGAAAGGTAGCTTCTCCTTCGAGGGGAAGGAGTACACCCTCAGCCACGGCTCTGTCGTCATCGCCGCCATTACTTCTTGCACCAACACCTCCAACCCTTCCGTCATGTTGGGAGCTGGTCTGTTGGCTAAGAAAGCAGTTGAAGCCGGCCTCTCTGTCTTACCTTATGTAAAAACATCTCTGTCTCCTGGTTCAGGAGTTGTGACCCATTACCTTCGTGAGTCAGGTGTTATACCATACCTCGACAAGCTCGGCTTCTCCATTGTCGGCTATGGCTGTATGACCTGTATTGGAAATTCTGGTCCTTTACCAGAAGCTATGATCGAAGCTATTGAAAAGAATGAACTTGTGTGCTGCGGTGTGCTGAGTGGAAACAGAAACTTTGAAGGGCGTATCCACCCAAACACTAGAGCTAACTATTTGGCCTCACCGCTCCTTGTTGTAGCCTACGCCATCGCTGGTAGAATTGATATAGATTTTGAAACTGAACCTCTTGGAAAAAGACCTGACGGAAGTGATGTTTTCTTGAGGGATGTCTGGCCTCTTCGCTCAGAAATCCAAGAAGTGGAAAAAAAGACAGTAATACCCGCTATGTTCAGAGATGTTTACGCAAGAATAGAAAACGGTTCCAACAGTTGGAGGGCTCTGAATGCTCCCGATGACAAGCTTTACCCATGGGATCCTTCGTCTACTTACATCAGGAGGCCTCCATTTTTTGATGGAATGACCCGAGATTTACCAAAGATTAAGAAGATTGAAAATGCTAAAGTGCTACTTTTCCTTGGCGATTCAGTAACTACAGATCACATCTCACCAGCTGGTAGTATCGCCAGGAACAGCCCAGCTGCACGCTACCTTGCTTCTAAAGGACTCACACCTCGTGAATTCAACTCATATGGCTCAAGAAGAGGAAACGATGAAGTAATGGCTCGTGGAACCTTTGCAAACATAAGACTTGTGAATAAATTCCTTAATCGTGCTGCTCCGAGGACAGTGCACATTCCATCAGAGGAAGAGTTGGATGTGTTCGATGCAGCTGAAAAGTATAAGAGTGAAGGAGTACCACTCATTGCAATCGTTGGAAAAGATTACGGTTCTGGCTCTTCTAGGGATTGGGCTGCTAAGGGGCCATTGCTTCTTGGGATCAAAGCTGTTATTGCTGAATCATATGAGCGTATCCATCGCTCCAACTTAGTCGGAATGGGAATAGTTCCTCTACAATTCCTGGAAGGACAGTCAGCTGACTCTCTTGGTCTAACGGGGAAAGAATCATACACCATCAACTTGCCCGCTGATCCTAGGCCACACCAGAAAATCACTGTTCAGACGGATAAAGGAAAGAGCTTTGAGGTCATCGTGAGGTTCGATACTGAGGTCGACCTCCTATACTTTAAACATGGCGGAATCCTCAACTGCATGGTCAGAAGAATGCTCGGTTAATTTTTTTTTTTTTTTTTTAAATTCAGTTTGTTGTAACTTGTATGATATTTTTCATGACAATAAAATTTGTTGGAAAAAGAATATGTAGTACATATTATGGTAATATGGTCATGGTCTGATGTTTTTGTATTGTATAATACATAGATATATATTTTTATATTTTTGTGATCTGAATATAAAGGGAG

Protein: RF -2: -101 -> -2776 (891 aa)

Comparison with *Halyomorpha halys*, PREDICTED: cytoplasmic aconitate hydratase-like - Sequence ID: XP_014275296.1

E: 0.0; bits= 1660

Query 3 GPNPYNKILKNLDVAGVSYKYYNLPELGPQYAKLPFSIRVLLESAVRNCDNFEVKQTDVE 62

GPNPY+KILK LD +GVSY YYNLPELGPQY KLPFSIRVLLESAVRNCDNFEVK+ DV+

Sbjct 5 GPNPYDKILKKLD-SGVSYNYYNLPELGPQYEKLPFSIRVLLESAVRNCDNFEVKEADVQ 63

Query 63 NILNWEKTGKDSSSVEVAFKPARVLLQDLTGVPAVVDFAAMRDAVKALGGNPDKINPICP 122

NILNWEKTGKDSSSVEV FKPARVLLQDLTGVPAVVDFAAMRDAVK+LGGNPDKINPICP

Sbjct 64 NILNWEKTGKDSSSVEVYFKPARVLLQDLTGVPAVVDFAAMRDAVKSLGGNPDKINPICP 123

Query 123 SDLVIDHSVQVDFARVDNALQKNEEIEFERNKERFTFLKWGAKAFRNMLIVPPGSGIVHQ 182

SDLVIDHSVQVDFARVDNALQKNEEIEFERNKERFTFLKWGAKAFRNMLIVPPGSGIVHQ

Sbjct 124 SDLVIDHSVQVDFARVDNALQKNEEIEFERNKERFTFLKWGAKAFRNMLIVPPGSGIVHQ 183

Query 183 VNLEYLARVVFTDELLYPDSVVGTDSHTTMINGLGVVGWGVGGIEAEAVMLGQAISMLIP 242

VNLEYLARVVFTD+LLYPDSVVGTDSHTTMINGLGVVGWGVGGIEAEAVMLGQAISMLIP

Sbjct 184 VNLEYLARVVFTDKLLYPDSVVGTDSHTTMINGLGVVGWGVGGIEAEAVMLGQAISMLIP 243

Query 243 QVIGYKLVGTLNQYATSTDLVLTITKNLRQLGVVGKFVEFFGPGVAELSIADRATISNMC 302

QVIGYKLVG LNQYATSTDLVLTITK+LRQLGVVGKFVEFFGPGV ELSIADRATISNMC

Sbjct 244 QVIGYKLVGKLNQYATSTDLVLTITKHLRQLGVVGKFVEFFGPGVVELSIADRATISNMC 303

Query 303 PEYGATVGFFPVDENSLYYLSQTNRDPAKIEAIRNYLKTVGMLRDYANAAQDPVYSEVVE 362

PEYGAT+GFFPVDENSLYYLSQTNRD AKI+AIR YLK VGMLRDYANAAQDPV+S+VVE

Sbjct 304 PEYGATIGFFPVDENSLYYLSQTNRDEAKIDAIRKYLKAVGMLRDYANAAQDPVFSQVVE 363

Query 363 LDLSTVVSCVSGPKRPQDRASVTEVKSLFTQALTNKVGFQGFGLKPSAVSAKGSFSFEGK 422

LDL+TVVS +SGPKRPQDRA+VTEVKS F ALT+KVGF G+GLK AV+A GSFS+EGK

Sbjct 364 LDLATVVSSISGPKRPQDRAAVTEVKSQFLSALTHKVGFNGYGLKSEAVNATGSFSYEGK 423

Query 423 EYTLSHGSVVIAAITSCTNTSNPSVMLGAGLLAKKAVEAGLSVLPYVKTSLSPGSGVVTH 482

EYTL HGSVVIAAITSCTNTSNPSVMLGAGLLAKKAVEAGLSVLPY+KTSLSPGSGVVTH

Sbjct 424 EYTLKHGSVVIAAITSCTNTSNPSVMLGAGLLAKKAVEAGLSVLPYIKTSLSPGSGVVTH 483

Query 483 YLRESGVIPYLDKLGFSIVGYGCMTCIGNSGPLPEAMIEAIEKNELVCCGVLSGNRNFEG 542

YL+ESGVIPYL+KLGFSIVGYGCMTCIGNSGPLP+ MIEAIEKNELVCCGVLSGNRNFEG

Sbjct 484 YLQESGVIPYLEKLGFSIVGYGCMTCIGNSGPLPDNMIEAIEKNELVCCGVLSGNRNFEG 543

Query 543 RIHPNTRANYLASPLLVVAYAIAGRIDIDFETEPLGKRPDGSDVFLRDVWPLRSEIQEVE 602

RIHPNTRANYLASPLLVVAYAIAGRIDIDFETEPLGK+ DGS+VFLRD+WPLRSEIQEVE

Sbjct 544 RIHPNTRANYLASPLLVVAYAIAGRIDIDFETEPLGKKADGSNVFLRDIWPLRSEIQEVE 603

Query 603 KKTVIPAMFRDVYARIENGSNSWRALNAPDDKLYPWDPSSTYIRRPPFFDGMTRDLPKIK 662

KKTVIPAMFRDVYARIENGSN+WR+LNAP D+LYPWD STYI++PPFFDGMTRDLP+IK

Sbjct 604 KKTVIPAMFRDVYARIENGSNNWRSLNAPSDQLYPWDSKSTYIKKPPFFDGMTRDLPEIK 663

Query 663 KIENAKVLLFLGDSVTTDHISPAGSIARNSPAARYLASKGLTPREFNSYGSRRGNDEVMA 722

KI NAKVLLFLGDSVTTDHISPAGSI+R SPAARYLASKGLTPREFNSYG+RRGNDE+MA

Sbjct 664 KITNAKVLLFLGDSVTTDHISPAGSISRKSPAARYLASKGLTPREFNSYGARRGNDEIMA 723

Query 723 RGTFANIRLVNKFLNRAAPRTVHIPSEEELDVFDAAEKYKSEGVPLIAIVGKDYGSGSSR 782

RGTFANIRLVNKFLN A P+T+H PS EE+DVFDAAE+YKSEGVPLIAIVGKDYGSGSSR

Sbjct 724 RGTFANIRLVNKFLNHAGPQTLHFPSGEEMDVFDAAERYKSEGVPLIAIVGKDYGSGSSR 783

Query 783 DWAAKGPLLLGIKAVIAESYERIHRSNLVGMGIVPLQFLEGQSADSLGLTGKESYTINLP 842

DWAAKGPLLLGIKAVIAESYERIHRSNLVGMGIVPLQFLEGQ+ADSLGLTGKE+YTINLP

Sbjct 784 DWAAKGPLLLGIKAVIAESYERIHRSNLVGMGIVPLQFLEGQTADSLGLTGKENYTINLP 843

Query 843 ADPRPHQKITVQTDKGKSFEVIVRFDTEVDLLYFKHGGILNCMVRRML 890

ADPRPHQKI VQT GKSFEVIVRFDTEVD+LYFKHGGILNCMVRRML

Sbjct 844 ADPRPHQKINVQTCNGKSFEVIVRFDTEVDILYFKHGGILNCMVRRML 891

Graphical representation


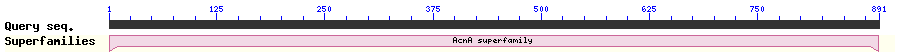


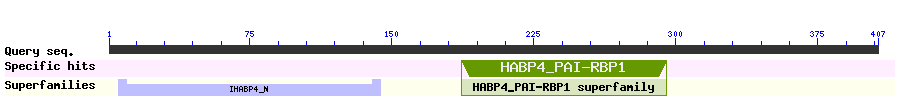


**Vasa intronic gene (VIG)**

> TRINITY_DN19858_c0_g1_i2 length= 1493 nt

CGCTGATGGTTAACCTCGTGGGATTTTTAGATTGTCGCCATTTTGCTTTGTAGCACATTTCAAATCCAGTGGACGAAGTTTTCAAACTCTGTCTGTTTTCCGAATTTAAGCATGTCTACTACACAGTACGGAATCGGTGTCACTAAGAACCGATTCGAGTTATTCGACATTGATGATGAGGATCCATTAGAAGTTTTGAAATCACGTGAACTGGAAAGGGAGAATCGTAAGAAATCCAAGCTGTGTGAAAAAGAAAATAAAGGGAAGGAACTCGTACCAAAACCAAAGGTTAGCATTCAAAGGAAAGGAATTAAGGAAACTCAGAATCTAAAACCTCTTGAAGGACAAAAACCGAAAGAGGAAGTGAAAGGTAGACCTGCTAGAATTGAGAGACCAGAACGTAAATTTGCTGGAACAGATGCTAGAGAAGTTCAAAATAACAGGCGTAATCGTGTAGAGGACCGTGCATCAAATGAATTTCAGCCAAGGGAAGAACGAGGTGGTGAAAGGTTCGACAGACGTGAGTATCGATCAGATAGGACAACTAGTGGATTTTATGGAGAAGGTGGTGATGGAAGAGGTCGAGGTAGGAGTGGACCCCAACGTGGCAATTTTATTCGTGGAGGTAGAGGAGGAAGAGGTAGCCAGCGCCCAACATTTGATGTTAGAGGAAAGAGAGAATATGATCGACAGTCAGGCTCTGATAAAACTGGTGTGAAACATGTAGACAAGAGGGAAGGTGCTGGCGCACACAACTGGGGGAATCTTCGAGACGATATTGTTGATATTCAAAATGCCCCTGTTACTGATGAAACTACCTGGACTGTTGAGAAAACTGAAGAACCTGCTGTGGAAACCAATGGAGCAATAGTTGAAACCGAAGAAGTTGTGCAAACAACTGCTGAAGAAGAATTAAAAGAGTTAACTTTAGATGAATGGAAGGCTTTAAAAGCTCCCCGGCAAAAACCTACCTACAATATTCGAAAGGCTGGTGAAGGAGAAGACCCTACTCAATGGAAAAAGATGTATGCTTTGCAGAAAAAGAAGGATGGAGAGGAAGAGGAGGAAGATGACGAATTCGAATATGAAAGTTTTGACTATCCCCAAAGAGTTGGACGCCAAAAGCATGTTTTGGATATTGACATACATTTTAAAGATACTCGGGGTGGAGGTAGAGGTCGTGGTGGAAGAGGTATGGGTCGTGGAGGCCCCCGTATGGGTCTGCGAGGAACAAATGGACCACCAGCTGAAAAAGTTCATATTGCCCGTGATCCTGTTATTCCAAAAATTGCTCCAAAAGTTGATGATGAGCATGATTTTCCTTCCTTGGGTTAAATGACACTAGTCTGGAATGAATAATTGTTAAATTCAATTAAAACTCCTTCTGAATTTAGACTGACATTTATTTATAGGAAAAAAAAAAAAAAAAAAAAACCTTTCCTATTTCCATTTCAGGAGTCTAGGTTACTTTTTTTTTCTAAATTGTATGTGTG

Protein: RF 1: 112 -> 1335 (407 aa)

Comparison with *Halyomorpha halys*, PREDICTED: plasminogen activator inhibitor 1 RNA-binding protein-like isoform X2 - Sequence ID: XP_014292052.1

E= 0.0; bits= 644

Query 1 MSTTQYGIGVTKNRFELFDIDDEDPLEVLKSRELERENRKKSKLCEKENKGKELVPKPKV 60

MSTTQYGIGVTKNRFELFDIDDEDPLEVLK RE ERE RKK+KL EKENKGKEL PKPKV

Sbjct 1 MSTTQYGIGVTKNRFELFDIDDEDPLEVLKLREQEREARKKTKLSEKENKGKELAPKPKV 60

Query 61 SIQRKGIKETQNLKPLEGQKPKE-EVKGRPARIERPERKFAGTDAREVQNNRRNRVEDRA 119

SIQRKGIKETQNLKPLEGQKPKE EVKGRPARIERPERKF GTD REVQNNRRNRVEDRA

Sbjct 61 SIQRKGIKETQNLKPLEGQKPKEAEVKGRPARIERPERKFTGTDPREVQNNRRNRVEDRA 120

Query 120 SNEFQPREERGGERFDRREYRSDRTTSGFYGEGGDGRGRGRSGPQRGNFIRGGRGGRGSQ 179

S +FQPREERGGERFDRREYRSDRTTSGFYGEGGDGRGRGRSGPQRGNFIRGGRGGRGSQ

Sbjct 121 STDFQPREERGGERFDRREYRSDRTTSGFYGEGGDGRGRGRSGPQRGNFIRGGRGGRGSQ 180

Query 180 RPTFDVRGKREYDRQSGSDKTGVKHVDKREGAGAHNWGNLRDDIVDIQNAPVTDETTWTV 239

RPTFDVRGKREYDRQSGSDKTGVKHVDKREGAGAHNWGNLRDDIVDIQNAPV DETTWTV

Sbjct 181 RPTFDVRGKREYDRQSGSDKTGVKHVDKREGAGAHNWGNLRDDIVDIQNAPVPDETTWTV 240

Query 240 EKTEEPAVETNGAIVETEEVVQTTAEEELKELTLDEWKALKAPRQKPTYNIRKAGEGEDP 299

EKTEEPAVETNGAIVETEEV+ TAEEELKELTLDEWKALKAPRQKPTYNIRKAGEGEDP

Sbjct 241 EKTEEPAVETNGAIVETEEVLPNTAEEELKELTLDEWKALKAPRQKPTYNIRKAGEGEDP 300

Query 300 TQWKKMYALQKKKDGEEEEEDDEFEYESFDYPQRVGRQKHVLDIDIHFKDTRGGGRGRGG 359

TQWKKMYALQKKKDGEEEE+DDEFEYES++YPQRVGRQKHVLDIDIHFKDTRGGGRGRGG

Sbjct 301 TQWKKMYALQKKKDGEEEEDDDEFEYESYEYPQRVGRQKHVLDIDIHFKDTRGGGRGRGG 360

Query 360 RGMGRGGPRMGLRGTNGPPAEKVHIARDPVIPKIAPKVDDEHDFPSLG 407

RGMGRGGPRMGLRGTNGPPAEKVHIARDPVIPKIAPKVDDEHDFPSLG

Sbjct 361 RGMGRGGPRMGLRGTNGPPAEKVHIARDPVIPKIAPKVDDEHDFPSLG 408

Graphical representation


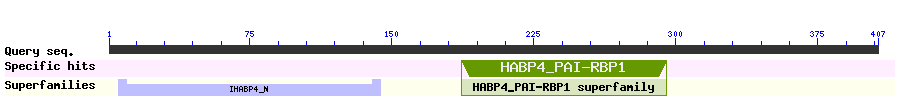


**Staufen**

>TRINITY_DN28984_c5_g1_i18 length= 1899 nt

CACTCTTTCCCTACACGACGCTCTTCCGATCTCTAAATCTTACTTTGTGCTGTTGCTGCGAAGGCTGTGACTGGGATTGCTGTGGTTGACTTTGAGGAAGCGGGTGACCACTCCTGCTCAACGTCGGGCTTAAGTAGCCAAGTTGTTGAAGGAGACCTTCGGCGGCATTTCGCTTCGCTACTTTTTTGTTCGGTCCCGTTCCAATATATGAATGTTTTCCAACAGTCACTTCCATAATAAACTCTCGTCTCCTCGGAAGACCACGTTCTTCCCGTAGAGTATAAACCGGTTCACGTTCTTTTTTAGCCTGTTGAATTTGGATCAAACGTGAAATAGGATTCATTTCATCACTATTATCACGTTCGTCGTATTTGGTCTCCTGAGAAACTGGAGTTTCCTTAATGATATTCCTAGATTTCTTCTTCCCCGTAGTTGCTTTACGTTTTGCACGAGCAAGTGCTGACGCACAACCAGGAGCTGTGACTGTAGTTGTTGGAGGTAGAGTCCTTAGTTGTTCCAGCATCTTCTCGGCAGCTCGTTTTTTCGAAATCTTCTTTCCATTCCCTTCACCAGTCGTTTCAAACTTATCGCCTACGCAACACCTCGTTACAAAAGTTTTCATATGTGGTTGACCTTTTTCAGAGATGACTTCAAAAAGAACAGGAAGATTTCTCTTTAATGCTATCTCATGAACTAGAGATATCGGTGACTTAAGTTCTGACTCCTGCTCGGTCAGTCCACCACAAATAGTAGAATTGGCTGGGCAAGTGTCATCAACGGGGAGGCTGTGCAGCAACTGAAGTGCTTTAGAAGCAGCATCGTGTCGTGCAGCTTGAGCTGAGTTCCCTTCACCAGTGAAATCACGCTCTCCTACCTTCACCACAACTTTGTAAAGAGGTGCTTTGTTCCCAGCACCATAAAACCTTGGATCAACGCGATTAAAACCACCTCTGAAATTAGGTTTTCCTCTTCCGTAAGGGAATGAGAATTGGTCATTGTAGGGCTGGGTGTACATTCCCCTGTGGGAATGGTGGTGGTAGCTACTGTACTGATGGAACTGAGGAGATTCAACGAGGGTATAAACAGTCGGCTCCCCCCTCTTCATTGCCAAAGCATTCAGTTCTACTGTAGGGGTGATGTTACTTTTGGAACCTCTAGATTGCCTAGAAGATTTAGGTGGTGGATGCTTGTAAGTAGTACTCTTCAGAGCGATACCAGCAGCCGCATGCTGCGCCTTTTTGATGCTGGCACCTTCTGCAGTATACTCCTCGTTACCGAGCTTCAATGTAACAGTAAACTTCTTTTTGTGCGGGGGACCATCTTCACTGGTTAGGCGATACTGATGTTCAATCTTATTGTACCTTGCCAACTCATTGACAAGGCACATCGGAGTCTTTTCTTTACCCAAGACAGATTGGCTGCCATCAGACGTGGCAGGTTCATCTTGACTTGATTGTCCGCCGGCAGAAGACCCTTCATTGTCAGTACCTACCGGCACTGGTGCGCTTCCTGCCATTGTTAACTGGCCTGTAACACATCCATTTTCATCAAATAAATCCTTGAACTCATCAATATCAATCCATTTTTTATAAACAGTTCCTTCAATAACTGTGCATAGTAGTATCAGGATAAGAAGAAATCCTGCATGTACTCCACCTACTGGACATAAGAGAAAAAATGTTGAAGTACAGGTTCCGGCTTGGGGGAGTCTGGTACGTTAAAGAAGACCGGATTAAACATTTTATTCTGAGGACACTTTCTTTATCTCTTATCTCCAGTTTATCCGATCTCTTTCGCTTCTTACTCTATTGTTTTTCGAATAAAGTTGTTCAAGACAAAACTGACAAAAACTTAATTATTCCAAGTGTAAATCAAAACTTGAAAATGAAATTAAAATAG

Protein: RF -3: -1516 -> -11 (501 aa)

Comparison with *Halyomorpha halys*, PREDICTED: double-stranded RNA-binding protein Staufen homolog 2 isoform X5 - Sequence ID: XP_014282526.1

E= 0.0; bits= 956

Query 1 MAGSAPVPVGTDNEGSSAGGQSSQDEPATSDGSQSVLGKEKTPMCLVNELARYNKIEHQY 60

MAGSAPVPVGTDNEGSSAGGQS+QDEPATSDGSQSVLGKEKTPMCLVNELARYNKIEHQY

Sbjct 1 MAGSAPVPVGTDNEGSSAGGQSTQDEPATSDGSQSVLGKEKTPMCLVNELARYNKIEHQY 60

Query 61 RLTSEDGPPHKKKFTVTLKLGNEEYTAEGASIKKAQHAAAGIALKSTTYKHPPPKSSRQS 120

RLTSEDGPPHKKKFTVTLKLGNEEYTAEGASIKKAQHAAAGIALKSTTYKHPPPKSSRQS

Sbjct 61 RLTSEDGPPHKKKFTVTLKLGNEEYTAEGASIKKAQHAAAGIALKSTTYKHPPPKSSRQS 120

Query 121 RGSKSNITPTVELNALAMKRGEPTVYTLVESPQFHQYSSYHHHSHRGMYTQPYNDQFSFP 180

RGSKSNITPTVELNALAMKRGEPTVYTLVESPQFHQY+ ++HSHRGMYTQPYND F+FP

Sbjct 121 RGSKSNITPTVELNALAMKRGEPTVYTLVESPQFHQYT--YNHSHRGMYTQPYNDHFTFP 178

Query 181 YGRGKPNFRGGFNRVDPRFYGAGNKAPLYKVVVKVGERDFTGEGNSAQAARHDAASKALQ 240

YGRGKPNFRGGFNRVDPRFYG GNKAPLYKVVVKVGER+F+GEGNSAQAARHDAASKALQ

Sbjct 179 YGRGKPNFRGGFNRVDPRFYGPGNKAPLYKVVVKVGEREFSGEGNSAQAARHDAASKALQ 238

Query 241 LLHSLPVDDTCPANSTICGGLTEQESELKSPISLVHEIALKRNLPVLFEVISEKGQPHMK 300

LLHSLP+DDTCPANSTICGGLTEQE+ELKSPISLVHEIALKRNLPVLFEVISEKGQPHMK

Sbjct 239 LLHSLPLDDTCPANSTICGGLTEQETELKSPISLVHEIALKRNLPVLFEVISEKGQPHMK 298

Query 301 TFVTRCCVGDKFETTGEGNGKKISKKRAAEKMLEQLRTLPPTTTVTAPGCASALARAKRK 360

TFVTRCCVGDKFETTGEGNGKKISKKRAAEKMLEQLRTLPPTTTVTAPGCASALARAKRK

Sbjct 299 TFVTRCCVGDKFETTGEGNGKKISKKRAAEKMLEQLRTLPPTTTVTAPGCASALARAKRK 358

Query 361 ATTGKKKSRNIIKETPVSQETKYDERDNSDEMNPISRLIQIQQAKKEREPVYTLREERGL 420

ATTGKKKSRNIIKETP+SQETKYDERDNSDEMNPISRLIQIQQAKKEREPVYTLREERGL

Sbjct 359 ATTGKKKSRNIIKETPISQETKYDERDNSDEMNPISRLIQIQQAKKEREPVYTLREERGL 418

Query 421 PRRREFIMEVTVGKHSYIGTGPNKKVAKRNAAEGLLQQLGYLSPTLSRSGHPLPQSQPQQ 480

PRRREFIMEVTVGKHSYIG+GPNKKVAKRNAAEGLLQQLGY SPTLSRSGH LPQSQ QQ

Sbjct 419 PRRREFIMEVTVGKHSYIGSGPNKKVAKRNAAEGLLQQLGYASPTLSRSGHQLPQSQSQQ 478

Query 481 SQSQPSQQQHKVRFRDRK 498

SQSQPSQQQHKVRF D K

Sbjct 479 SQSQPSQQQHKVRFSDEK 496

Graphical representation


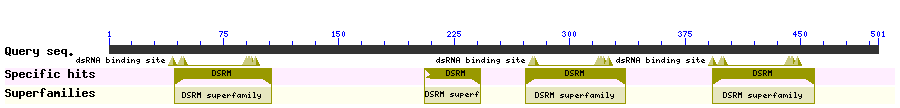


**RNA helicase Belle**

>TRINITY_DN29259_c2_g3_i3 length= 3421 nt

TATAAATAAAATTTAAATTAAAAAAAAAAAAAAAAAAAAAAAAAAAAAAAAAATAAAAAAAAAAAAATTAGGAAAAACCAATTCAGTTTAACTTGAGATTGAGTATCTGATGTTCAATCCCTTCGATACAAGCACTCCTTGAGCAAAACTAATAATAAATAATTCTGAATGGTAGATGCATTTATTAGCTCTTCATTTACTCAAAAGGAAAATATTTGTACACAATAAATCAATTAACTTTTCCCCAAGTTAGATCATGGGCACTAACTTATGATAGTTGTCATTTATATTTACAAAAATTATTTGTCATCCCTTAAAAAAATTACATTTAACTCAACATTTTTTTTCTCAATTTACTTTCTTAAACTGAAGACTGGAGTGCCGTTGCCGTGTCCAGGTGTGGCACATATCTCTGTGATATATATACATATAAATAAAGAGGTTTATGCATCGGATAGCCTTAAAAGCTGTGCTAAAGACCTTATTCGATCAAATCTTAAGGGGTCCTTAACTGGAGAATCTTTGGTTTATACCCTTCTCTCCAAAGCAATCCGATCGTAGTTGGCCTTCACAATAAATGATACTATACAAGGCCTACCAATATGTATATATACAATCACATAATGACTTTATTTAATATATTCAGTAAGAATAAAATCACAGTGTTAATGCCCCTAACTTATTAAAAATACACATAATGAATTTTTAATATTCTTTAGGCGTATAAACATTGAGTTGTATGGGATCATATATAATATTTACACATCTATATATGTACATACAACATAATATAATCAGTTGTGTATACGCCCATATGTGCATAGTTGTAGTTTGTGGCTAGGCTGAAGAAGAGCGGGGGCGAGATCGATTGGCTGAGAGAGGCAGGGAGTATGGTGAGAGAATTATTGCCCCCACCAATCGTGGTCGGAGGAGCTGTTGTTCTGGTTGGATGAATATGAACCACCATAGCCTGCGTTGCCGCTACCCCCTCCGTAGTAACTATTAGAACTAGAGTATCCACCATAGCCAGATGATCCTCCTCCAGATCTTGAAGGGTTCCTTGAAGAGCCACCACCACCGCCGCCACTGCTTCCTCCCTGTTGACGGTAGTCCCGTGCTCCAAATCCACTGGAGAATCCTTTGCCGCCTCGGCGAGATGAAAGAGGCTGCTTAGTTTCTGCAGCCATTCTTTGCATCCATGAAGGCATGGTCTGCTTAGTTTCTTGTATGAGATCAAACAGATCTCTAACTAAATTCCTATTTTTATCATTGAAGAAGGATGTAGCCAAACCTAAGTTGCCCATACGGCCAGTACGTCCAATTCTGTGAACGTATTCTTCTACATCATTTGGTAAATCAAAATTAATCACATGTTTTACATGAGGAATATCCAAACCCCTAGCCGCTACAGCAGTCGCAACAAGAACTGGTGTTTGGCCTGTTCGGAAGTTTCTCAGAGCTTCTTCTCTTTCCTTCTGCGTCCTATCACCATGGATGGAAGTTACAGGGTAGCCAACACTGTAGAGAAAATCTTCAAGCGAGTCGGCACCTTTCTTTGTTTCAACAAATACCAATGTCAGCGAACCTTCTTCAGCATTTTTCGAAGCATTAAGAAGGTCCAACAGAAATCCTCTTTTGTCATGTTCTTCAACCCAGATGATTTGTTGGGTAATATTTTCTGAGGTAGATCCGACACGGCCAATGGCCAAAAATATATAGTCATTCAAAAAATCTAATGCCAATATCTGAATTTCTTTTGGGAAAGTAGCAGAAAACATCAGAGTTTGTCTGTCGCCAGTAGGAGGCATAGAATGCTTTTCAACGATTCTTCTGATTTGGGGTTCAAAACCCATATCTAACATTCTATCAGCTTCGTCAAGTACCAAATACCTGCAGTTTTCTAAGCCTATTCTTCCTCTAGCAAGCATATCAACTAAACGCCCTGGGGTAGCAACTAACAAATGACATCCACGGTCAAGATCTCTCATCTGCTCAGCAACATGAGCACCCCCATACACAACACATGGTCGAAGACGAGACCTATACGCAAATTTACGAGCTTCATCATAAATCTGGGTAGCAAGTTCTCTCGTAGGTGCAAGAACAAGACCCATAGGAAACTGCTTACGTTTTCCTGGGAACTTGGGATTCTGTGGTCCCCTTTCAAGAATTTGATTAAGGATAGGAATCAGGAAAGCGGCTGTTTTACCACTGCCAGTCTGGGCGCAAGCCATCAGATCCCTTTTAGCCATAATGATAGGTATGGCATTTTTCTGTACTGGGGTTGGCTTATCATAGCGAGCAAGCCGTATATTATTCGATATTATCTCATTCATCTGCAAATCGTCAAATGTTTGGATTTGGGGTGGTACTTGGATACCAGTAGCTTCAACTGGGATATCCTCATACTTATTAAAGTTAATACCAGTATTGGCATTGCCAAATAGTTCAAATTCAAGTCGCTCATCTCTGGGGAGCAGCGTGGTCCATCCTGCAGAAGAACCTTGGGGCTTCTCTTGCCATCTATCATTGCCAGTATTCCCTGAAGTGGGAGTGTTCCCGCCTCTTCTACTGCCCCAGTCATCACGATCACGTTCCCTATCACGTTCCCTGCTGCTTCGGAATGAAGAGCCTCCCCTATCACCCCAAGATCTGTCTCCATTCTGTGAGTCATAATCGCCATTTCTCCTACTTCCTCCTCGATAACTGTCGCTTCTGCCACCTCCACCTCCACCTCCACCGCCACCGCCAGCACTGCCGCCTCCACCACTACGAAATCCACCACCGCTGCCTCCTCTACTTGGAAAGTCACGACTGTCGTTGTAAGAAGAATAGTTAGAAGAGGGTGGTTGTTCTCCGCTGCGAGATTCACCTCCAGCCTGTTTGTTACGGAGATGGGGAGGAATGTAGGGGCCGGTTTCTTTACGGCTACCCTCCAAGTCCAGACCAGCAAATCGCTGCTCTAGACCTGATCCATTTTGGTTGGGTACATTACTCATATTACTATAAATCAGTACTTTAAAAGTAAGCCCTTTCAAGTGCTTTTCCAATAAAAGTAAAACACAAAGAGAACTGGAATGATGTACACGGGATTATACAGAGGACATGTACACTAAGCATCGATAAGTCGATTTTACCGGAAGCTTTCGATTATGTCAACAACCCCTCCAAGGCCAATAAATAATTCTTACTTAAAATTTGTTCCTTTCTAGAATATTGTTCTTATAAGAAAACAGTAATTTGTTGCAAAGCTATATGTAAATATCTTTATTCAAATGTTTGATATCTATAAAGTTGGATTTGGTGAACTTGTCTTGATTCCATTATTGATGTAATTTGTTTAAGAATTAAAAATGAACTTTATTTTGTAGCAAATTTTTTTTTTTTTGTTTGAGAAAAGGTTGATAATCATATATTTCGG

Protein: RF -3: -2999 -> -903 (698 aa)

Comparison with *Halyomorpha halys*, PREDICTED: ATP-dependent RNA helicase bel isoform X2 - Sequence ID: XP_014279436.1

E= 0.0; bits= 1377

Query 1 MSNVPNQNGSGLEQRFAGLDLEGSRKETGPYIPPHLRNKQAGGESRSGEQPPSSNYSSYN 60

MSNVPNQNGSGLEQRFAGLDLEGSRKETGPYIPPHLRNKQAG ESR GEQPPSSN+SSYN

Sbjct 1 MSNVPNQNGSGLEQRFAGLDLEGSRKETGPYIPPHLRNKQAG-ESR-GEQPPSSNFSSYN 58

Query 61 DSRDFPSRGGSGGGFRSGGGGSAG----GGGGGGGGGGRSDSYRGGSRRNGDYDSQNGDR 116

DSRD+PSRGGSGGGFRSGGGG G GGGGGGGGGRSDSYRGGSRRNGDYDSQNGDR

Sbjct 59 DSRDYPSRGGSGGGFRSGGGGGGGSSGSAGGGGGGGGGRSDSYRGGSRRNGDYDSQNGDR 118

Query 117 SWGDRGGSSFRSSRERDRERDRDDWGSRRGGNTPTSGNTGNDRWQEKPQGSSAGWTTLLP 176

SWGDRGGSSFRSSRERDRERDRDDWGSRRGGNTPTSGNTGNDRWQEKPQGSSAGWTTLLP

Sbjct 119 SWGDRGGSSFRSSRERDRERDRDDWGSRRGGNTPTSGNTGNDRWQEKPQGSSAGWTTLLP 178

Query 177 RDERLEFELFGNANTGINFNKYEDIPVEATGIQVPPQIQTFDDLQMNEIISNNIRLARYD 236

RDERLEFELFGNANTGINFNKYEDIPVEATG+QVPPQIQTFDDLQMNEIISNNIRLARYD

Sbjct 179 RDERLEFELFGNANTGINFNKYEDIPVEATGVQVPPQIQTFDDLQMNEIISNNIRLARYD 238

Query 237 KPTPVQKNAIPIIMAKRDLMACAQTGSGKTAAFLIPILNQILERGPQNPKFPGKRKQFPM 296

KPTPVQKNAIPIIMAKRDLMACAQTGSGKTAAFLIPILNQILERGPQN +FPGKRKQFPM

Sbjct 239 KPTPVQKNAIPIIMAKRDLMACAQTGSGKTAAFLIPILNQILERGPQNTRFPGKRKQFPM 298

Query 297 GLVLAPTRELATQIYDEARKFAYRSRLRPCVVYGGAHVAEQMRDLDRGCHLLVATPGRLV 356

GLVLAPTRELATQIYDE+RKFAYRSRLRPCVVYGGAHVAEQMRDL+RGCHLLVATPGRLV

Sbjct 299 GLVLAPTRELATQIYDESRKFAYRSRLRPCVVYGGAHVAEQMRDLERGCHLLVATPGRLV 358

Query 357 DMLARGRIGLENCRYLVLDEADRMLDMGFEPQIRRIVEKHSMPPTGDRQTLMFSATFPKE 416

DMLARGRIGLENCRYLVLDEADRMLDMGFEPQIRRIVEK+SMPPTG+RQTLMFSATFPKE

Sbjct 359 DMLARGRIGLENCRYLVLDEADRMLDMGFEPQIRRIVEKNSMPPTGERQTLMFSATFPKE 418

Query 417 IQILALDFLNDYIFLAIGRVGSTSENITQQIIWVEEHDKRGFLLDLLNASKNAEEGSLTL 476

IQILALDFLNDYIFLAIGRVGSTSENITQQIIWVEEHDKRGFLLDLLNASKNAEEGSLTL

Sbjct 419 IQILALDFLNDYIFLAIGRVGSTSENITQQIIWVEEHDKRGFLLDLLNASKNAEEGSLTL 478

Query 477 VFVETKKGADSLEDFLYSVGYPVTSIHGDRTQKEREEALRNFRTGQTPVLVATAVAARGL 536

VFVETKKGADSLEDFLY VGYPVTSIHGDRTQKEREEALRNFRTGQTPVLVATAVAARGL

Sbjct 479 VFVETKKGADSLEDFLYGVGYPVTSIHGDRTQKEREEALRNFRTGQTPVLVATAVAARGL 538

Query 537 DIPHVKHVINFDLPNDVEEYVHRIGRTGRMGNLGLATSFFNDKNRNLVRDLFDLIQETKQ 596

DIPHVKHVINFDLPNDVEEYVHRIGRTGRMGNLGLATSFFNDKNRNLVRDLFDLIQETKQ

Sbjct 539 DIPHVKHVINFDLPNDVEEYVHRIGRTGRMGNLGLATSFFNDKNRNLVRDLFDLIQETKQ 598

Query 597 TMPSWMQRMAAETKQPLSSRRGGKGFSSGFGARDYRQQGGSSGGGGGGS-SRNPSRSGGG 655

TMPSWMQRMAAETKQPLSSRRGGKGFSSGFGARDYRQQGGSSGGGGGG SRNPSRSGGG

Sbjct 599 TMPSWMQRMAAETKQPLSSRRGGKGFSSGFGARDYRQQGGSSGGGGGGGPSRNPSRSGGG 658

Query 656 SSGYGGYSSSNSYYGGGSGNAGYGGSYSSNQNNSSSDHDWWGQ 698

SSGYGGYSSSNSYYGGGSGNAGYGGSYSSNQNNSSSDHDWWGQ

Sbjct 659 SSGYGGYSSSNSYYGGGSGNAGYGGSYSSNQNNSSSDHDWWGQ 701

Graphical representation


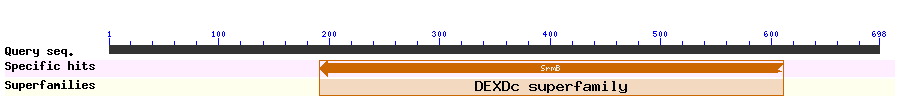


**Protein arginine methyltransferase 7 (PRMT)**

>TRINITY_DN28449_c4_g3_i2 length= 1682 nt

GAAAATTTATAGAAGATAAGTGGTTGAAGTCTCATTTCGAAAGTTTAGCTTGCTGTATTCATTGGCTTTGTTTGTCAATGAATAGTCTTGTTTATTTTAACTTTGACATCTTTACTAATCTGTTTTAGTTAGCCTCCACTATAATAAGTTATGAGTTACTCATTTTGGAACGTGTAATCGCTTGTTTATAGTTATGAACGTATTTTCTTTTCCTTGACCCTTCTTGAACTTTGTGAAATGTTCATCAATTAAAATAATAATGGCATTTCGAAATAATCGGAAATTAATTAAAACCGTATTAATTGAAAGAAGACACTTTTTTTCAAATATTTCTGTTAGTAAACGTGATGAAAGTTGTTTTAGTTTATTGAAGTCTAGGATAAAATTTAATGGCCCTCTCACAGTATATGATTACATGAAAGAAGTTTTGACCAATCCTGTTAGTGGTTATTATGTTACTAAAAATGTTATTGGAAAACAGGGAGACTTCATCACTTCTCCTGAAATTTCTCAACTTTTAGGTGAAATGGTTGCTTTGTGGACTTTAAATGAATGGAACAAGTTAGGCTCTCCAAAACCTCTTCAGTTAGTTGAATTAGGACCAGGAAGAGGGTCTATGATGCACGATATTTTAAGGGTTTACAAAAAATTAAATCTTGATAATGATATTAGTGTTCATTTTGTGGAAGTCAGTGATGAACTCAGTACAGTTCAAGCAAATAAACTTTGTACTTCTATTTTAAAACATGACAATAAAACTTGTTATCAAGAAGGAAAAACAGAAAATAATGTTCCTATTTATTGGCATAAGGCGATTCAGGATGTTCCTAAAAAGTTTTCTTGTATTTTAGCTCATGAGTTTTTTGATGCATTACCTGTTCATATTCTACAGAAAACAGTAAATGGTTGGCGGGAAGTCTTGATAGATCTTACTGAATGTGACACGAAACTTCGCTATGTTATTTCTTCTGGCCCAACACCTGCTTGTGTATTTAGTAAATACTCCAACGGTCGAGATTATTTTGAAATCAGTCCGCAGTCTGGTCTTTTGCTTGAACATGTTGCCTGTTTATTAGAAGAACATGGTGGGTTTGCCTTGATTATAGACTATGGCCATACTGGAGAAAAAAAAGATACATTTAGGGGTTTCAGGAACCATCAATTAATTAATCCATTAGAAGCTCCGGGTACCTCTGATTTGACAGCAGATGTAGATTTTGCATTTCTTAAGGATGTTACTAAAGATAAATTAATTTCCTTTGGCCCTGTTTCCCAAAGGTCTTTCCTGAAACAATTACACATAGATGTTCGCTTACAGATAATTTTGAAGAGCTGTAAGAGTGAGAAAGAAAAAGAAGACATCTTGTCTGGTTACCACATGATCATGGATTCTGATAAGATGGGAGAATGTTTTAAAGTAATGTCATTATTTCCTGCAGTTCTGAAAGAATACTTGAATAATTTTCCAGTTGTAGGCTTTATAAATAAATAACTGCTTATTAACTTTTTATACTGAAGGAGATACAATCAATTATTTAAGTATTTATTTGGCATAACTCCTATTATATTTAATATTTGTAGCTACATGTTTCGGAAACAATGTTCCATCATTAGGCTTCTCTTAACCCTTTTAATGCGGAAATGCAAATGGTTTTTTTTGTCTCCCGCGTTTTTATTTACCC

Protein: RF +2: +260 > +1492 (410 aa)

Comparison with *Halyomorpha halys*, PREDICTED: protein arginine methyltransferase NDUFAF7, mitochondrial - Sequence ID: XP_014292128.1

E= 0.0; bits= 726

Query 1 MAFRNNRKLIKTVLIERRHFFSNISVSKRDESCFSLLKSRIKFNGPLTVYDYMKEVLTNP 60

M + RK+IKTVL ER+ FFS+I ++K DE+CF+LLKSRI F GPLTVYDYMKEVLTNP

Sbjct 2 MFLQGLRKIIKTVLTERKIFFSSIPLNKSDETCFNLLKSRINFAGPLTVYDYMKEVLTNP 61

Query 61 VSGYYVTKNVIGKQGDFITSPEISQLLGEMVALWTLNEWNKLGSPKPLQLVELGPGRGSM 120

VSGYYVTKNVIGKQGDFITSPEISQLLGEMVALWTLNEW KLGSPKP QLVELGPG+GSM

Sbjct 62 VSGYYVTKNVIGKQGDFITSPEISQLLGEMVALWTLNEWTKLGSPKPFQLVELGPGKGSM 121

Query 121 MHDILRVYKKLNLDNDISVHFVEVSDELSTVQANKLCTSILKHDNKTCYQEGKTENNVPI 180

MHDILRV K+L LD I+VHFVEVSDELS Q +KLCTS+LKHDNK+ YQEGKTENNVPI

Sbjct 122 MHDILRVCKQLKLDEHINVHFVEVSDELSKAQGDKLCTSVLKHDNKSYYQEGKTENNVPI 181

Query 181 YWHKAIQDVPKKFSCILAHEFFDALPVHILQKTVNGWREVLIDLTECDTKLRYVISSGPT 240

YWHKAIQDVPK F+CILAHEFFDALPVHIL++TV+GWREVLIDLTEC +KLRYVIS PT

Sbjct 182 YWHKAIQDVPKNFTCILAHEFFDALPVHILKQTVDGWREVLIDLTECCSKLRYVISPAPT 241

Query 241 PACVFSKYSNGRDYFEISPQSGLLLEHVACLLEEHGGFALIIDYGHTGEKKDTFRGFRNH 300

PACVFSKYSNGRDYFEISPQSGL+LEH+AC LEEHGGF LIIDYGH GEKKDTFRGFRNH

Sbjct 242 PACVFSKYSNGRDYFEISPQSGLILEHIACTLEEHGGFVLIIDYGHDGEKKDTFRGFRNH 301

Query 301 QLINPLEAPGTSDLTADVDFAFLKDVTKDKLISFGPVSQRSFLKQLHIDVRLQIILKSCK 360

QL++PL +PGTSDLTADVDFAFLK+VTKDKL+SFGPV QRSFLK+LHIDVRLQ++LK+CK

Sbjct 302 QLVDPLVSPGTSDLTADVDFAFLKNVTKDKLLSFGPVPQRSFLKELHIDVRLQVLLKNCK 361

Query 361 SEKEKEDILSGYHMIMDSDKMGECFKVMSLFPAVLKEYLNNFPVVGFINK 410

+EKEK DILSGYHMIMDSDKMGECFKVMSLFP+VLKE N++PVVGF+NK

Sbjct 362 NEKEKNDILSGYHMIMDSDKMGECFKVMSLFPSVLKELFNDYPVVGFVNK 411

Graphical representation


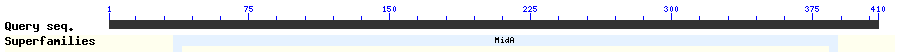


**Gawky**

>TRINITY_DN28905_c2_g1_i2 length= 4952 nt

ATCATGACAAAAGTAAATCATTGAAATCTTTTCCAAAAAAAAAAAAATTAATAAACAATAATAATTAAACATTACCACAATGATTGTTAAAGTAACCCAATGAAAGGAATTTACCGTTTTGCGTGGGGGAGAGGGAGTGGGCTCAGTGCCTAGCACAGCGGAGCAGTGTAAGGGCCGATGTCCCACGAGATGTGCGACAACTCCGATTTGGCTTCCTCGTTCGGCCATTGCCTGCTTTAAATATTTCTGCCACAATGAGAAAAGCCCAAGATAATTAATTAGTCTATAAGACTTTGGTTTTTACATATTGCCTGCCAAAGACGTACTGAGAGCCAATGTTTCGAAACAATTCTAGTTCAAATGAGATTTCTTCTAAAACTAATGCCTTTGTACAAAATAAAGACGAGGAGGACAAATCTGAGAGCTTGTTAAGAGGTATGGCGCAGCCTCCCAAGCCTACGAGTCCTACTCATCAAGTGCCTGAGAAAAGGGACGTAATGGTGGTAGATATTGGGGTGAGAGAAGATGATGGCCCCGTCCTGACTGTGATAACCAACCATCCGTCCCAAGCGCCCGCCAAGATTTTCTCATCAGAAATTGGTGAAAGTGAATCTGACGGTTCTTCCAAAATGCCACTAGCAGAGACACAAGGAACGGGTGCTCTTTGCTTAGATAGCATTAAGTCTATTAGTGTTAATGAATCATTTAGTGTTAAGGATAAATTTATTTGCCCGAGCAAGAGTTCAATTCTGCCGCCAAACATTCCAAATACCAACGATGATACCGATCAAGATGTTAAAAGCTTTAAAATCTGTGATTATTATACTCGGTGGGGAATACCACGAAACTTGAAACTGTTAGGAGGAGGAGAGAGTTCACTTACTACAGGGACTACCGGTTGGGGATCCCCACCTTCAAATCAAGGTGGTAGTACTGGTTGGAACAGTGCTAATACTACTAGTGGAAGTAATAGTTCTTCAGGACAAGGACAAGCAGGTACTGGGCAAAGCCCAGCTCCTGCCTCTGCTGGACAAACTTGGGGTAGTTCCCAAAATAATACCAACAACAGCAATAGTAATAGTAACAACAATAATGGATCTCGCAGTTCTGTTAGCCAGCAAGGTGGAGGTAGCACACAACAGCAACCAGGAGGGGGGCCACCTAGTCAGTCCACGGCTCCACCTGTAGCAACTGTGTCTACATCAACTGTTACAACTGCTCCAGCCTCTTCAGCAACAAATACGTCCAATATTAACACTGCTACAACATCAGCTTCTCAACAAAATGGTTCAGCTAGTGGCAACCAAGTGGTAGGAAGTGGTTCTACCTGGGCAACTGCTGTTGGTAAAGGGCTTCCTCCGACAAGCACAGCTTCAACTCCAACTTCAAGTGGAAGCACATCTACTAAGCAACAAATGGAACAGCTAAACACAATGAGAGAAGCCCTTTACAGTCAAGATGGATGGGGTGGTCAAAATGTTAACCAAGATAGCAATTGGGATATACCAGGTTCCCCAGAACCAGGCACAAAAGATAGCAACAATGCAGCTCCTGTTCCTCTTTGGAAACTGCCTATCAATAATGGTACTGATCTTTGGGAGGCTAATCTGAGAAATGGCGGTGTTCCTCCTCCTGTAAGCCAACAGAGTCAGAAAACACCTTGGGTTCACACTCCAAGCACCAACATTGGTGGAACATGGGGTGAAGATGATGAAGGTGATGCTTCTAATGTGTGGACTGGTGTTCCTCAAGCACAGACTGGATGCGGTCCTCAATGGCCAGCTCAACCACCTCCTATTTGGCCTGCTACTAAGAAAGAAGGAGATTGGGGAGGGCCTAACTGGAATGATCAACGTGACACAAGAGATCTTCGCCACAGTGATATGAGACAAATGATGGATGCTAGAGATCATATGAGACCAACTTCTATTGATCACAGATCAATGGGAGGCAATGATGTTATAATGCGAGGTGACCCACGCGGAATCAGCGGTAGGCTTAATGGAGTAACGAGTGAGGCCATGTGGCCTGGTCCAGGTCCTCATCACCATATACCCCATCATCAAGGAAAATTGCCTTCTCAACCTAATCAACCAGTTAATCAATGGAGCAGCTCTGGACCCCCAATGAAGGACATGACTGGTCTTGGTGGTAAATCAACTGGTTGGGAGGAGCCTTCACCTCCAGCTCAACGGAGGAATATGCCTAATTATGATGATGGAACATCACTTTGGGGCCCACAGCATCCCAGACCTACCATCCAAGGTCAAAATAAAGTTTCTCATTGGAAAGAAATGCCGGCTCCTGGAATAGGGCGAGGTGGTTTACAGTGTCCCCCAGGCCGTGCTAACCCTACAATGAAACCAGATCAACCTTTATGGCCTCATCATCCCAGAAATGAACGGGGATGGGAAGGAGGAATGGATAGTGGACCCTGGGGAGATGAAAAACCAACTCCTGCTGCTGCACCTTGGATGGACCAAGGTCTAGCTCCTTCATCATGGCAAGGTGGACCAAAACATAAACCAGCATGGGATGGATCTGATTTAGATCCCACTTCTTGGGTTCACTCAAAACAGCCCTCTAAGTCCGTTTCAAAGGAATTTATTTGGACAAGCAAGCAGTTTCGTATTTTGTCTGAAATGGGTTTCAAGAAAGAAGATATAGAAAGTGCATTAAGAAGTTCCGGAATGAGCCTTGAAGATGCATTAGATCAGCTTAACACAAATAGGGGACTGAGTGCTGGAGGTGGTAGTGAGAGGTGGCCACGGCATGGTGACTTAGATTCAGAGCATGCTGCAATTATGAATACATTTCCTTCTCCTCAGCAAACAATCTGTCTTGCTCCATTTCCACAGGGTGGAGGTGGTGGTGGAAGTGGAAGTGGACCAGGAGGTGGACCTACCTTAGCGACTATAACACCAGCTGTAATGCAGAAACTTCTTGCACAACAACCACCACAGCAACAACCTTTTGCCCAGCAATCTTCAAGAACACAACAAACCCAACAACCATCAGCTCAGCAGCTTAGAATGCTGGTCCAGCAAATTCAAATGGCTGTTCAAACTGGTTACCTCAGCCCCCAGATTTTAAATCAACCATTAGCTCCACAAACCCTTATTTTATTGAACCAACTACTACAACAGATAAAAAATCTTCAACAGCTTATGCAACACCACACAGTAATGCAAGTAAATCCTCTTGGAAAACCAAGCTCAAATCACTTGTTACAATTATCTGTGCAGATTACAAAGACCAAGCAACAGATTACAAATCTTCAGAATCAGATTGCTGCTCAGCAAGCTGTGTATGTTAAGCATCAACAGCATACACCACCTACTTCTGAGTTTTTCAAGAGTTCATTACATGAACCAATTTCTGCACTTCATCCTAATTTTTCTGATCTTTCTCTTAAAGATCCCCCGACCAGTGGAACTAGCCAGCAATCACGATTAAATCAGTGGAAGTTACCTGCCCTGGAAAAAGACTCAGATATTGGGACAGGTGAATTTTCTAGAGCTCCAGGTACAACAGCTAAGTCAGCTCAAGGCTCTTCTTCACCTAATACAAATTTATTACTTGGGCAGGCTGATGGTACTTGGTCTTCTGTAAATCGTGAATCTAGTTGGCCTGATTCATCCGGTGATGATGCTTCTGGCAAGGATTGGCCAAATTCCAGTCAACCTCCATCTCAAGCATTCTCTGATCTTGTTCCTGAGTTTGAACCAGGAAAGCCTTGGAAGGGAAACCCACTAAAAAGCATCGAGGATGATCCAAGCCTTACACCTGGTTCGGTTGTGAGGTCTCCTCTTTCTCTGCCTTCAATAAAGGATACACATATATTATCAACTAGTACTGGTGCTGGCAAAGCTTCACCTACTACCAGTTCTTCTTTAGATATTATCCCATCTCTTGGCTTGTCATCATCTACTTGGAGCTTTAATCCACCACCTTCTTCATCTAACACTAGTGTGAAGCTGAATTCTAGTGGAGCTGCTGGAGGTGGTAGTGGGTCAACATCAAATAATGGTGGAGGCAAAAATAGTACTTCAACTTGGGAAACTAATTCGTCTGAATTGTGGGCTCCCAAAAGAGGGCCTCCTCCAGGTTTACCAGCTAAACCTAGTGGTGGTTCAAGTGGTGGACAGGCTGCAAATGGTTGGGGACCTTTGTCTAGTAGTGGCCGTTGGTCAACTGGGCAAGGTTGGCCTGGACCGAATCAGGCGGCTGCAACTCAGCCAGGTTCTACTTGGTTGTTATTGCGAAATCTTACTCCTCAGATTGACGGTTCAACTTTAAAAACTCTATGTTTACAACATGGGCCATTATCAAATTTCCATCTCTACCTTAACCATGGCATCGCTCTTGCTAAATATGCATCTCGGGAAGAAGCCAATAAGGCCCAAGGTGCTTTAAACAATTGTGTTCTTGGTAACACTACAATATTTGCTGAGAGTCCCAGTGAGACCGATGTGTTGTCATTACTTAATCATCTTGGTGGACAAGGAGGGACCGCCAGTGGCAGCTCAGGATGGCGTGGTAAGGAAGCTTGGGGCAATTCCCAGCTTTGGGGAGCCACTGGAGCAAGCTCAGCTGCTGCTTCTTTGTGGGCAGGAGATAGTGATCAGCATCGTAACACTCCATCCTCAATAAATTCTTATTTGCCAGGTGACCTTCTTGGTGGTGAGTCTATTTAGGCAAATCTTCATTCTTCTCTCAAACCTTCACCAAATTCTTCTCGATCTATAAATACGTCAATCAAAACTATTGAACAAAAAAAATACAAAAAAAACAAAAAAAAACAAGTACTTTGATCTCAGAAACACCACATGACCTTTTTATTATAAATATATATGATATGAAGTATATGCAATTAATTATTTGTACCAGGAACGTATATCTTATTATTATTATTATCATTATTATTATTATTATTATTATTATCATTATCA

Protein: RF -3: -336 -> -4697 (1453 aa)

Comparison with *Halyomorpha halys*, PREDICTED: protein Gawky isoform X1 - Sequence ID: XP_014288686.1

E= 0.0; bits= 2803

Query 1 MFRNNSSSNEISSKT-NAFVQNKDEEDKSESLLRGMAQPPKPTSPTHQVPEKRDVMVVDI 59

MFRNNSSSNEISSKT NAFVQNKDEEDKSESLLRGMAQPPKP SPT QVPEKRDVMVVDI

Sbjct 1 MFRNNSSSNEISSKTTNAFVQNKDEEDKSESLLRGMAQPPKPISPTLQVPEKRDVMVVDI 60

Query 60 GVREDDGPVLTVITNHPSQAPAKIFSSEIGESESDGSSKMPLAETQGTGALCLDSIKSIS 119

GVRE+DGPVLTVITNHPSQAPAKI SSEI E ESDGSSKMPLAET GTGALCLDSIKSIS

Sbjct 61 GVREEDGPVLTVITNHPSQAPAKISSSEIDECESDGSSKMPLAETHGTGALCLDSIKSIS 120

Query 120 VNESFSVKDKFICPSKSSILPPNIPNTNDDTDQDVKSFKICDYYTRWGIPRNLKLLGGGE 179

VN+SFSVKDKFI PSKSSILPPNIP TNDDTDQDVKSFKICDYYTRWGIPRN KLLGGGE

Sbjct 121 VNKSFSVKDKFIYPSKSSILPPNIPKTNDDTDQDVKSFKICDYYTRWGIPRNFKLLGGGE 180

Query 180 SSLTTGTTGWGSPPSNQGGSTGWNSANTTSGSNSSSGQGQAGTGQSPAPASAGQTWGSSQ 239

SSLTTGTTGWGSPPSNQGGS+GWNSA+TTSG+NSSSGQGQAG GQSPAPASAGQTWGSSQ

Sbjct 181 SSLTTGTTGWGSPPSNQGGSSGWNSASTTSGNNSSSGQGQAGAGQSPAPASAGQTWGSSQ 240

Query 240 NNTNNSNSNSNNNNGSRSSVSQQGGGSTQQQPGGGPPSQSTAPPVATVSTSTVTTAPASS 299

NNTNNSNS+SNNNNGSR+SVSQQGGGSTQQQPGGGPPSQSTAPPVATVST TVTTAPASS

Sbjct 241 NNTNNSNSSSNNNNGSRNSVSQQGGGSTQQQPGGGPPSQSTAPPVATVSTPTVTTAPASS 300

Query 300 ATNTSNINTATTSASQQNGSASGNQVVGSGSTWATAVGKGLPPTSTASTPTSSGSTSTKQ 359

ATNTSNINTATTS+SQQNGS +GNQVVGSGSTWATAVGKGLPPTSTA+TPTSSGSTSTKQ

Sbjct 301 ATNTSNINTATTSSSQQNGSTTGNQVVGSGSTWATAVGKGLPPTSTATTPTSSGSTSTKQ 360

Query 360 QMEQLNTMREALYSQDGWGGQNVNQDSNWDIPGSPEPGTKDSNNAAPVPLWKLPINNGTD 419

QMEQLNTMREALYSQDGWGGQNVNQDSNWDIPGSPEPGTKDSNNAAPVPLWKLPINNGTD

Sbjct 361 QMEQLNTMREALYSQDGWGGQNVNQDSNWDIPGSPEPGTKDSNNAAPVPLWKLPINNGTD 420

Query 420 LWEANLRNGGVPPPVSQQSQKTPWVHTPSTNIGGTWGEDDEGDASNVWTGVPQAQTGCGP 479

LWEANLRNGGVPPPVSQQSQKTPWVHTPSTNIGGTWGEDDEGDASNVWTGVPQAQTGCGP

Sbjct 421 LWEANLRNGGVPPPVSQQSQKTPWVHTPSTNIGGTWGEDDEGDASNVWTGVPQAQTGCGP 480

Query 480 QWPAQPPPIWPATKKEGDWGGPNWNDQRDTRDLRHS-DMRQMMDARDHMRPTSIDHRSMG 538

QWPAQPPPIWPATKKEGDWGGPNWNDQRDTRDLRHS DMRQMMDARDHMRP SIDHRSMG

Sbjct 481 QWPAQPPPIWPATKKEGDWGGPNWNDQRDTRDLRHSSDMRQMMDARDHMRP-SIDHRSMG 539

Query 539 GNDVIMRGDPRGISGRLNGVTSEAMWPGPGPHHHIPHHQGKLPSQPNQPVNQWSSSGPPM 598

GNDVIMRGDPRGISGRLNGVTSEAMWPGPGPHHHIPHHQGKLPSQPNQPVNQWSS+GPPM

Sbjct 540 GNDVIMRGDPRGISGRLNGVTSEAMWPGPGPHHHIPHHQGKLPSQPNQPVNQWSSTGPPM 599

Query 599 KDMTGLGGKSTGWEEPSPPAQRRNMPNYDDGTSLWGPQHPRPTIQGQNKVSHWKEMPAPG 658

KDMTGLGGKSTGWEEPSPPAQRRNMPNYDDGTSLWGPQHPRPTIQGQNKVSHWKEMP PG

Sbjct 600 KDMTGLGGKSTGWEEPSPPAQRRNMPNYDDGTSLWGPQHPRPTIQGQNKVSHWKEMPTPG 659

Query 659 IGRGGLQCPPGRANPTMKPDQPLWPHHPRNERGWEGGMDSGPWGDEKPTPAAAPWMDQGL 718

IGRGGLQCPPGRANPTMKP+QPLWPHHPRNERGWEGGMDSGPWGDEKPTP AAPWMDQGL

Sbjct 660 IGRGGLQCPPGRANPTMKPEQPLWPHHPRNERGWEGGMDSGPWGDEKPTPTAAPWMDQGL 719

Query 719 APSSWQGGPKHKPAWDGSDLDPTSWVHSKQPSKSVSKEFIWTSKQFRILSEMGFKKEDIE 778

APSSWQGGPKHKPAWDGSDLDPTSWVHSKQPSKSVSKEFIWTSKQFRILSEMGFKKEDIE

Sbjct 720 APSSWQGGPKHKPAWDGSDLDPTSWVHSKQPSKSVSKEFIWTSKQFRILSEMGFKKEDIE 779

Query 779 SALRSSGMSLEDALDQLNTNRGLSAGGGSERWPRHGDLDSEHAAIMNTFPSPQQTICLAP 838

SALRSSGMSLEDALDQLNTNRGLS GGG ERWPRHGDLD EHAAIMN FPSPQQTICLAP

Sbjct 780 SALRSSGMSLEDALDQLNTNRGLSGGGGGERWPRHGDLDPEHAAIMNAFPSPQQTICLAP 839

Query 839 FPQGGGGGGSGSGPGGGPTLATITPAVMQKLLAQQPPQQQPFAQQSSRTQQTQQPSAQQL 898

+PQGGGGGGSGSGPGGGPTLATITPAVMQKLLAQQPPQQQPFAQQSSRTQQTQQPSAQQL

Sbjct 840 YPQGGGGGGSGSGPGGGPTLATITPAVMQKLLAQQPPQQQPFAQQSSRTQQTQQPSAQQL 899

Query 899 RMLVQQIQMAVQTGYLSPQILNQPLAPQTLILLNQLLQQIKNLQQLMQHHTVMQVNPLGK 958

RMLVQQIQMAVQTGYLSPQILNQPLAPQTLILLNQLLQQIKNLQQLMQHHTVMQVNPLGK

Sbjct 900 RMLVQQIQMAVQTGYLSPQILNQPLAPQTLILLNQLLQQIKNLQQLMQHHTVMQVNPLGK 959

Query 959 PSSNHLLQLSVQITKTKQQITNLQNQIAAQQAVYVKHQQHTPPTSEFFKSSLHEPISALH 1018

PSSNHLLQLSVQITKTKQQITNLQNQIAAQQAVYVKHQQHTPPT+EFFKSSLHEPIS LH

Sbjct 960 PSSNHLLQLSVQITKTKQQITNLQNQIAAQQAVYVKHQQHTPPTTEFFKSSLHEPISGLH 1019

Query 1019 PNFSDLSLKDPPTSGTSQQSRLNQWKLPALEKDSDIGTGEFSRAPGTTAKSAQGSSSPNT 1078

PNFSDLSLKDPPTSGTSQQSRLNQWKLPALEKDSDIG+GEFSRAPGTTAKSAQGSSSPNT

Sbjct 1020 PNFSDLSLKDPPTSGTSQQSRLNQWKLPALEKDSDIGSGEFSRAPGTTAKSAQGSSSPNT 1079

Query 1079 NLLLGQADGTWSSVNRESSWPDSSGDDASGKDWPNSSQPPSQAFSDLVPEFEPGKPWKGN 1138

NLLLGQADGTWSSVNRESSWPDS+GDDASGKDWPNSSQPPSQAFSDLVPEFEPGKPWKGN

Sbjct 1080 NLLLGQADGTWSSVNRESSWPDSAGDDASGKDWPNSSQPPSQAFSDLVPEFEPGKPWKGN 1139

Query 1139 PLKSIEDDPSLTPGSVVRSPLSLPSIKDTHILSTSTGAGKASPTTSSSLDIIPSLGLSSS 1198

PLKSIEDDPSLTPGSVVRSPLSLPSIKDTHILSTSTGAGKASPTTSSSLDIIPSLGLSSS

Sbjct 1140 PLKSIEDDPSLTPGSVVRSPLSLPSIKDTHILSTSTGAGKASPTTSSSLDIIPSLGLSSS 1199

Query 1199 TWSFNPPPSSSNTSVKLNSSGAAGGGSGSTSNNGGGKNSTSTWETNSSELWAPKRGPPPG 1258

TWSFNPPPSSSNTSVKLNSSGA GGGSGSTSNNGGGKNS STWETNSSELWAPKRGPPPG

Sbjct 1200 TWSFNPPPSSSNTSVKLNSSGAGGGGSGSTSNNGGGKNSASTWETNSSELWAPKRGPPPG 1259

Query 1259 LPAKPSGGSSGGQAANGWGPLSSSGRWSTGQGWPGPNQAAATQPGSTWLLLRNLTPQIDG 1318

LPAKP+GGSSGGQA NGWGPLSSSGRWSTGQGWPGPNQAAATQPGSTWLLLRNLTPQIDG

Sbjct 1260 LPAKPTGGSSGGQATNGWGPLSSSGRWSTGQGWPGPNQAAATQPGSTWLLLRNLTPQIDG 1319

Query 1319 STLKTLCLQHGPLSNFHLYLNHGIALAKYASREEANKAQGALNNCVLGNTTIFAESPSET 1378

STLKTLCLQHGPLSNFHLYLNHGIALAKYASREEANKAQGALNNCVLGNTTIFAESPSET

Sbjct 1320 STLKTLCLQHGPLSNFHLYLNHGIALAKYASREEANKAQGALNNCVLGNTTIFAESPSET 1379

Query 1379 DVLSLLNHLGGQGGTASGSSGWRGKEAWGNSQLWGATGASSAAASLWAGDSDQHRNTPSS 1438

DVLSLL HLGGQG ASGSS WRGKEAWGNSQLWGATGASS AASLWAGDSDQHRNTPSS

Sbjct 1380 DVLSLLQHLGGQGSAASGSSAWRGKEAWGNSQLWGATGASSTAASLWAGDSDQHRNTPSS 1439

Query 1439 INSYLPGDLLGGESI 1453

INSYLPGDLLGGESI

Sbjct 1440 INSYLPGDLLGGESI 1454

Graphical representation


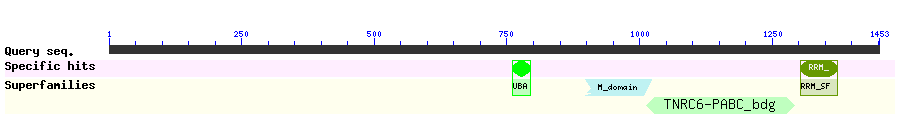


**Data S4:** Sequences of *E. heros* Nucleases.

**Exoribonuclease 1 (Eri1)**

>TRINITY_DN29099_c1_g1_i3 length= 5604 nt

CTGAAGTTTAAAATAGAACACTTTGATATAATTATTACAATGATAATTTTTCTAAAACAGGTATATATCATATGATATGAGCTAAATAATTTTATGTACAAATATGTAATATGGAAGGCAGCTGTAATACTGCCTATGTTTCCTTCATAATGTACAGTTATAAATATATATCATATTTATATAAATCTATATTAATTAATTTGAAGATTATGCACAAATAGCTGACTATTTGCTATCTGGTAATGGGACTGAAAAGTTTGCAGCAATTCTACTTTTTACTTTTGCTGGTCTCCGAGGAACTTGGTTAGGCTTGATAGATTTTTGATTTCCTTCATTACCAGGAGACTGAAGGGTCTCTTCGTTTTTAACTGCTTTTGATCCATAACCTCTTGATTCTTCCTGTGGAGAGTTTGGAATCACTGGACTCAGAACTTTTTGGGGAGACCTTACTTGTTCCTGAGGTCTGCCAGGGAAATTCCTCATTGCAGGTCCAAACTGCATTTCAGAAATAGCCCCTCCTGTTCTACTAGAGCACCATTGCATCGGAGGAGTAGGAAGCTGCTGGTTTATTCCTATTCTGGAAGTGGATTGTTGTGGTTGTGGTTGTGGCTGCGGCTGCGGCTGCAACATCATAGGCTGAGGTGGTCGATGAATATTTCTGTAGCCTAATGGACTTCTCACAATTTCTTCTCTCTTTAATATTTGTTTAATTGCTTTACAACAAGCATTTTCAGCAGCCTCTTCTTGATTTCTACAAGAATCTCCGATAACTTTTCCAATTTCATTTGAAAACGTTAAAATGCAAGTTATTTCTCCAGAAGATGGTAAGTGCTTGTAATTATACTTAGGCAACTGAATATTTTTGTTAAGACATAAATTCTTTAACTCTAAATCACAATTAATATGATTGCAAGTCTTAGGTCTCATTATACTCCCTTGATTATAAATAAAATTATTGGTATTAAGAGGAGCCATATTTGCTGCTCTTCGACTGCTGTTATTGGCGTGCTGGATCAATTGGGGTTGAAAGTCAGGAGTAAAATGTTGATTGACTGGAGCTACTGGAGTTTCATTCAACTTCAGAAACTTACGAAGAGCTATAGTTTGCTCAACTACAACAGTTTCAGTGACAGGGGATACTGGTGTAACCTTTGGGGAAGGAACACCAGGTCTTAAAGATTTTTCTTCAGGTACTTTCTGATTCTGAAGGAAATTCCACATAGCTTTAAATTCCAAATCGGCTCCTAATGGCCTATTATGATGGGGAACATGGTTGGTAGGAAATGGCGCCTGTTTGGCAGGATTAAAATTCTGAATAAACTGCTTTACAGATTCATTACCTTGAGGAGTAGTACTTGGTATATTCATTTCTCTGTTCCTTGGGATTTGATAGTACTTGTTTTCATTACGTGGGCTACTATTATAATTCATACTTCTCCATGTTTCTCCAGCATTAGTTTGATTCACATTGGCCATTTTAGGAGCAGTAGAAACATTTACTTTTCCTGACTTTCCTGCCCCATAAGATAGGTTAATAAATGCCTGGCTAGGAAGCCTGTAAATCCTAGCTTCTTCGCAAGATCGATGCGAGCCGCCAATCACAGGACTATCGAATAAGACATCATACAAAACATTGACATTGCTGGCAGGTTTGTCAATACCTACAACAGTCCCCATTAATCCCAGTGGTATACAGTGGTTTTCTCGAACGGAAACAATCCTATCGAGCATGTCCACAGAAGTATTTGGATCAGGAGGTAACTTGCTAAGTCTTGGCTTTGGAATGTATATACTCGAAGGATTGATTATAACGTCAATAGTTTTGTCAGATTTCATAGAGCAGTAATCAATGACACGTTTTTCGATTTCTTTAATAGCTTCAGGGGTCAGAATTTGGGAACCTGACGGCCTTCTCTCGGCACTACTATGGGGCTGCGCTTTAAGCCAACTTTTAACTTCATTTACTTTTTCATCCGTGTTTTCTGGAAAGATATCCTTATTCAGAAACATGTCGCCATCACTTTTCATTGTTTGAAGGTTTTCAAACAATTCAGGAAATTTACCCATATATGCATCTAGAAGTGCTATAGTTTTTTCAGAATAAAACCAAACATTGCCTTCTCTTCTTGTATAGCCAATGAGCTCGTCTCCTTGTTTGGTAAATTTTAGCCCAAGACCGACATTGATCCTTTCATCCTTATCTGCGTTCTTAATAAAAATAGATCCAGTCAAACGGGAAAAAAGATGGCTACCAATACCAAGCCTCTGAGCAGCTATACTGCCTGGGTAATATTTGAGTTCTGTTTTATGTGATAGTTCAAGGATTCCAGATAGATCCAAATTCGATTCTACTTGGATTGTAACTTGCACCATTTTGTCATTTAGAACAGATTTCACTTTTCCAAACATCCCGTAGTGTAATGGTTCCAGTAGAAATACTTGTTTCCCAATCGAAAAGACTTTATTCAGATCCAGTATTTTTTCCAATTCTTCATCATCTTCGATTAATTTTGAATTATCAAGGTCCAGATACGTCTGATAAGGAAATGGGTAACTTTGTTCACTCCACATGTAAGTCTTTTTTCCCTGGGTCTTAGAAGAATCACTCTCGTCGAAATGTATTCTAACATCTTCCACAGTTTTAACGTAAATTATAAGATCCAGTTTACCCAGATCTATTCCCAGGTTGTTGAAATGTCTTTTTAAAACATCCTCTGCATCGTATTTATGTCTTTTATTTTCACATTTAACAGGTGGTTTATTATGCTCAATTCTGTACTGGAACTGTGGCGATGAAATAGCAGTAGCCATTGCCTTCTGAATATGTGGCCAACCGACGTAAATTACTTTGTTGCGCAATTTTTCAGCTATTGAATACAATACTTCTTCTTCACTCTCAATTTCTATTGGATCAATATTTAAAATCATGTTTTCTCCTCTACTGGGATGCTCAAAAACTTTAACCTTAGCCATTTTTAATTCAACCTTAAAAGAAATACCACGCAGTTGTGGAAATCCATATCGTACTCCTGAAAGAGCCCCAGGGAGAATACCTTTGACAACTTTCTTCGGATCTACAGCGATGTCTGTTCTTGAAAGAGGAGTCATTAGAGCATGATTATTTTTAATTGTTGGAAAGTACTCAGGAGCTTCACAAAGTCCTAAATTATCTGAAGTATATTCATAAACTTCCATAGGCTGTACGGAATTTCTTCTCACTTCTTCATCAGTCAGGTCTTTATAGTGAGGTTGCATGGCATCTAGAAGACTTTTCTCCTCGATGAATGGAATCAAAACGACTGCTTCCCAATCATTCAATTTATCATTTAAATCGGTTTCAAATTCAGGAGGGTAGAACTGTATTATAGGCGATTCAGGGGAATCCATGAGGTCTTGAAATGGTTCAGGGACAATACTTTTGCTAGCAGAAGGCAACACTGCAAGAAGCTGCTGGAATGGCTGAAATGGAGCTGATAAATCAAATTTAAAATCGAAATTGGTGAATCCTTTGATATCCGATACATAGGGGGCATAGTGATGAGGATAGTACCACGACCAAGACGGGCACCCTGTATAATAGTAAGACAGATTCCACTGTATCGCTCGAACGTAGCATTCGGCTTGGCTCTTCAATACCTCACTGTCAACATTTTCATAGTTCAATTTTTCCATATAGAAGTTTGCCTTATAATGTGTGAACTCATCCTTTATAAGTTGTTCTTCGTCAGAACTATCAAAAGCGAGATCATCGTCACCTTCGAACATTTTGTTAGAATCTTCTACAAGTTTTATTAAATCATCATCAAATTCAGGTAAGCCTTCATCGGTTTCATCAGAAAGAAGATTGTTTCCCCTCTTACTTTCTAAATACTTAATATCAGCTTGTATATCCCTAAAGTTTTCTAGCTCTATTTCAGCTAGTTTCGTCAAATATTTTTCAAATCTTTCTAAATTTAAAGTCCCATAATCATTTATGTACCCTCCTAAAGAAGGAAGAATCTCCATATAAGCTTGGTACAATTTATGAAGAGCATTGTTAGCAATGTGAAATTTGGGAAGATTAGGAATGAAATCATTTCCAACTAAAAAGCCCATCAATACCCAATCATCGATGATCCGCTCAATATCATATTCAAATGACAATTTATCCTTTAATGACCGGAATTCCAAATCTAAATATCCTCGCATCATAGATAGATGAAGTAAAAAAAATTTAGTTTCTTCGACATTTTTAGGCTTTTTAGCATTTCGTGAAAATTTAACCTCTTCTCTCAAAAGTGCAAAGTGTGGTTCATGGGTACACAGCCCTAGCATAATCAGATCAGCATCCAAGCCGTGAAGGCAATGCCTCGTATTCGGATCCCAATCACTTTGAGATCGTAAGTACCTAATATAATCCATAATCTTATGCTCTCCTTCACCTGGAACATTATGACCAGATAAAATAATTTTTGGCCTTTGCCATGTCTCATCAGTCGAGATCTTGTGTTTTATAAAATATTGTAGCTGCTTGTGTAATCTATCCATGAAAACAGTTCCGGGGGTTATACAGTTAGAATCAAATCTTTCACTGTCTGGTAGCGGTTCACCCTTATCGACAGCTCTTTTAAGGGCTAATTCAGCATCTTTTGCTGTTCTAAATCTTCGACTTCTCTGCTGATTCATTTTAGCTCTAGGAGCAACACCATCAATAGCAATAAAAAAAAGTTTTTTAGGTTGAATCAGCCTGAACAAAACCTCAAGATAATAAAATATATCTTTAAAAATTTCTTCTTCAGTGATCCTAAAATGAACACTGTCATCAGGATGCGAACAATTGTGGATGATCCCATTAGCATCAATGTAAAAATTGTCAAATTCTGGAATCTCATATTCTCTCACTACTTCACAAAGGCATGGATAACGTTCACTAATAAACCTGAAAAACTTTGGTACACCCATTTTCACTTCTTGTTGTCAATTCTATGTCTTTTCAAAACATAAGCCCACTTCAAAACAATATCTCTAACAGTGTTGCATTGAGTTCTTGATGTAGTAACGAGGTAACTTGGACAGAAATTCGCTGAATCGTCATTCTAATCAATCCTAACTTTTATCGTCCGCATCACTCTCATAGAAACAAGTAATCTTTTATCTCCCCTTCCATCTCTTCGCATTTTCCTTATTACATTCATTTTTAAAAATAATCTTTGTACCATATAAATGATTTAATAGGTAAGGATGTAATCTAAAAATAAGCGCATTTAAAAGCAACATTTTCACTGAGAGCAAATAGTACTTTATCTAGTCTAGCTACTGCAAATAAAGAATATTTGTTATTTATAATCATATTGCTTTTGTAATTTTTTTTAAATTCCTATTTGATCAACATCAGCGTATTTTCAAATGTTCTACGTTTGAATTATATTAATGCCACTATTCGTAAATCTTATTCTCAAAACGGACAAACAGCATGGATCTTGGAACCTCTAAACCCATCACTCGAATTAAGGTTTTTTCTTAAAGAAAGTTTCATTGTGCATTATTATTGTGAGAAAACAGTAAATAAGAATCAGATTCATCTTGTTTCATCTACTTATAATTTTTTTTACATATTATTTACAA

Protein: RF -1: -4941 -> -226 (1571 aa)

Comparison with *Halyomorpha halys*, PREDICTED: 5'-3' exoribonuclease 1 - Sequence ID: XP_014290344.1

E: 0.0; bits= 2701

Query 1 MGVPKFFRFISERYPCLCEVVREYEIPEFDNFYIDANGIIHNCSHPDDSVHFRITEEEIF 60

MGVPKFFRFISERYPCLCEVV+EYEIPEFDNFYIDANGIIHNCSHPDDSVHFRI+EEEIF

Sbjct 1 MGVPKFFRFISERYPCLCEVVKEYEIPEFDNFYIDANGIIHNCSHPDDSVHFRISEEEIF 60

Query 61 KDIFYYLEVLFRLIQPKKLFFIAIDGVAPRAKMNQQRSRRFRTAKDAELALKRAVDKGEP 120

KDIFYYLEVLFRLIQPKKLFFIAIDGVAPRAKMNQQRSRRFRTAKDAELALKRAVDKGE

Sbjct 61 KDIFYYLEVLFRLIQPKKLFFIAIDGVAPRAKMNQQRSRRFRTAKDAELALKRAVDKGET 120

Query 121 LPDSERFDSNCITPGTVFMDRLHKQLQYFIKHKISTDETWQRPKIILSGHNVPGEGEHKI 180

LPD++RFDSNCITPGTVFMD+LHKQLQYFIKHKISTD TWQ+PKIILSGHNVPGEGEHKI

Sbjct 121 LPDTDRFDSNCITPGTVFMDKLHKQLQYFIKHKISTDATWQKPKIILSGHNVPGEGEHKI 180

Query 181 MDYIRYLRSQSDWDPNTRHCLHGLDADLIMLGLCTHEPHFALLREEVKFSRNAKKPKNVE 240

MDYIRYLRSQSDWDPNTRHCLHGLDADLIMLGLCTHEPHF+LLREEVKFSR AKKPKNVE

Sbjct 181 MDYIRYLRSQSDWDPNTRHCLHGLDADLIMLGLCTHEPHFSLLREEVKFSRTAKKPKNVE 240

Query 241 ETKFFLLHLSMMRGYLDLEFRSLKDKLSFEYDIERIIDDWVLMGFLVGNDFIPNLPKFHI 300

ETKFFLLHLSMMRGYLDLEFRSLKDKLSFEYDIERIIDDWVLMGFLVGNDFIPNLPKFHI

Sbjct 241 ETKFFLLHLSMMRGYLDLEFRSLKDKLSFEYDIERIIDDWVLMGFLVGNDFIPNLPKFHI 300

Query 301 ANNALHKLYQAYMEILPSLGGYINDYGTLNLERFEKYLTKLAEIELENFRDIQADIKYLE 360

ANNALHKLYQAYMEILP+LGGYINDYGTLNLERFEKYLTKLAEIEL+NFRDIQADIKYLE

Sbjct 301 ANNALHKLYQAYMEILPTLGGYINDYGTLNLERFEKYLTKLAEIELDNFRDIQADIKYLE 360

Query 361 SKRGNNLLSDETDEGLPEFDDDLIKLVEDSNKMFEGDDDLAFDSSDEEQLIKDEFTHYKA 420

SK G LLSDETDE + +FDD+L++LVEDSNKMFEG+DD+AFDSSD+EQLI+DEF H+KA

Sbjct 361 SKSGKKLLSDETDEAISKFDDELMRLVEDSNKMFEGEDDMAFDSSDDEQLIQDEFRHHKA 420

Query 421 NFYMEKLNYENVDSEVLKSQAECYVRAIQWNLSYYYTGCPSWSWYYPHHYAPYVSDIKGF 480

FYMEKLNYENVDSEVL SQAECYVRAIQWNL YYYTGCPSWSW+YPHHYAPYVSDIKGF

Sbjct 421 RFYMEKLNYENVDSEVLMSQAECYVRAIQWNLFYYYTGCPSWSWFYPHHYAPYVSDIKGF 480

Query 481 TNFDFKFDLSAPFQPFQQLLAVLPSASKSIVPEPFQDLMDSPESPIIQFYPPEFETDLND 540

T+FDFKFDLSAPFQPFQQLLAVLPSASK+IVP PFQDLM SP+SPIIQFYPP+FETDLND

Sbjct 481 TDFDFKFDLSAPFQPFQQLLAVLPSASKNIVPGPFQDLMASPDSPIIQFYPPDFETDLND 540

Query 541 KLNDWEAVVLIPFIEEKSLLDAMQPHYKDLTDEEVRRNSVQPMEVYEYTSDNLGLCEAPE 600

K+NDWEAVVLIPFIEEKSLLDAMQP+YKDLT++E RRNSVQPMEVYEYTSDNLG CEAPE

Sbjct 541 KMNDWEAVVLIPFIEEKSLLDAMQPYYKDLTEDENRRNSVQPMEVYEYTSDNLGRCEAPE 600

Query 601 YFPTIKNNHALMTPLSRTDIAVDPKKVVKGILPGALSGVRYGFPQLRGISFKVELKMAKV 660

+FP I+NNHA +T LSR+DIA+DP+KVVKGILPGALSG RYGFPQLRGISFKVELK+AKV

Sbjct 601 HFPPIENNHAQVTTLSRSDIAIDPRKVVKGILPGALSGARYGFPQLRGISFKVELKLAKV 660

Query 661 KVFEHPSRGENMILNIDPIEIESEEEVLYSIAEKLRNKVIYVGWPHIQKAMATAISSPQF 720

KVFEHPSRGENMIL ID IE ES EE+LYS+AEKLRNK+IYVGWPHIQK ATAISS Q

Sbjct 661 KVFEHPSRGENMILKIDRIEFES-EEILYSMAEKLRNKIIYVGWPHIQKGKATAISSEQC 719

Query 721 QYRIEHNKPPVKCENK-RHKYDAEDVLKRHFNNLGIDLGKLDLIIYVKTVEDVRIHFDES 779

Q+RIEHNKPP K ++K RHK DA D+ +R+ NLGIDLGK+D+IIYVKTV D+R+ FDE+

Sbjct 720 QFRIEHNKPPFKYDDKNRHKLDAADLQQRYLLNLGIDLGKVDIIIYVKTVVDIRVQFDEN 779

Query 780 DSSKTQGKKTYMWSEQSYPFPYQTYLDLDNSKLIEDDEELEKILDLNKVFSIGKQVFLLE 839

DSS+TQGK+TYMWSE YPFPYQTYLDL+N LI++DEELE++ DLN++F IGKQVFL++

Sbjct 780 DSSQTQGKRTYMWSETHYPFPYQTYLDLNNFTLIDNDEELEQMKDLNEIFPIGKQVFLMD 839

Query 840 PLHYGMFGKVKSVLNDKMVQVTIQVESNLDLSGILELSHKTELKYYPGSIAAQRLGIGSH 899

L+YGMFGKVKS+ ++K V+V I VE NLDLSGILELSHKT+L+YYPGSIAAQRLGIGSH

Sbjct 840 LLNYGMFGKVKSIDDNKRVEVIINVEPNLDLSGILELSHKTDLRYYPGSIAAQRLGIGSH 899

Query 900 LFSRLTGSIFIKNADKDERINVGLGLKFTKQGDELIGYTRREGNVWFYSEKTIALLDAYM 959

LFSRLTGSIFIKNAD+DERINVGLGLKFTKQG+ELIGYTRREGN+W+YSEKTIALLD YM

Sbjct 900 LFSRLTGSIFIKNADRDERINVGLGLKFTKQGEELIGYTRREGNIWYYSEKTIALLDEYM 959

Query 960 GKFPELFENLQTMKSDGDMFLNKDIFPENTDEKVNEVKSWLKAQPHSSAERRPSGSQILT 1019

KFPELFE L + KSDGDM+LNKDIFP+NTDEK++E+++WLKAQ HSSAERRPSGS +LT

Sbjct 960 DKFPELFEKLHSTKSDGDMYLNKDIFPDNTDEKMSEIRTWLKAQSHSSAERRPSGSLLLT 1019

Query 1020 PEAIKEIEKRVIDYCSMKSDKTIDVIINPSSIYIPKPRLSKLPPDPNTSVDMLDRIVSVR 1079

EA+KEIEKRVI+ C+ +++KT+ V NPSSIYIPKPRLSKLPPDPNT+V+MLDR++ VR

Sbjct 1020 SEAMKEIEKRVIELCANRANKTLAV--NPSSIYIPKPRLSKLPPDPNTTVNMLDRVIFVR 1077

Query 1080 ENHCIPLGLMGTVVGIDKPASNVNVLYDVLFDSPVIGGSHRSCEEARIYRLPSQAFINLS 1139

ENHCIPLGLMGTVVG+DKPA++ N LYD+LFD PVIGGS RSC++ RIYRLP+Q+FIN+S

Sbjct 1078 ENHCIPLGLMGTVVGVDKPANSGNALYDILFDCPVIGGSCRSCDQPRIYRLPNQSFINIS 1137

Query 1140 YGAGKSGKVNVSTAPKMANVNQTNAGETWRSMNYNSSPRNENKYYQIPRNREMNIPSTTP 1199

YGA KSGKVNVS KMANVNQTN GE+WR NYN+SPRNENK+YQIPRNRE++ P TP

Sbjct 1138 YGARKSGKVNVSAPSKMANVNQTNVGESWRGSNYNNSPRNENKFYQIPRNRELSQPG-TP 1196

Query 1200 QGNESVKQFIQNFNPAKQAPFPTNHVPHHNRPLGADLEFKAMWNFLQNQKVPEEKSLRPG 1259

QGN+SVKQF+QNF+PAKQ FP NHVPH NR LGAD EF+AMWNFLQNQKVPEEKSLRPG

Sbjct 1197 QGNDSVKQFLQNFHPAKQGAFPANHVPHPNRSLGADSEFQAMWNFLQNQKVPEEKSLRPG 1256

Query 1260 VPSPKVTPVSPVTETVVVEQTIALRKFLKLNETPVAPVNQHFTPDFQPQLIQHANNSSRR 1319

VP + PV+ VVEQTIALRKFLKLNETP+ PVNQHFTP+FQPQLIQHANNSSRR

Sbjct 1257 VPPQQKV---PVSPVEVVEQTIALRKFLKLNETPLPPVNQHFTPEFQPQLIQHANNSSRR 1313

Query 1320 AANMAPLNTNNFIYNQGSIMRPKTCNHINCDLELKNLCLNKNIQLPKYNYKHLPSSGEIT 1379

A NMAPLNTNNF++NQGS+MRPKTC+HINCDLEL+NLC+NKNIQ+P+YNYKHL SSGEIT

Sbjct 1314 AGNMAPLNTNNFLFNQGSVMRPKTCSHINCDLELRNLCVNKNIQIPRYNYKHLASSGEIT 1373

Query 1380 CILTFSNEIGKVIGDSCRNQEEAAENACCKAIKQILKREEIVRSPLGYRNIHRPPQPMML 1439

CI+TFSN+IGK +GDSCRNQEEAAENA CKAIK ILKREEIVRSPLGYRN+HRP QPM +

Sbjct 1374 CIITFSNDIGKAVGDSCRNQEEAAENASCKAIKLILKREEIVRSPLGYRNVHRPAQPMAM 1433

Query 1440 QPQPQPQPQPQQSTSRIGINQQLPTPPMQWCSSRTGGAISEMQFGPAMRNFPGRPQEQVR 1499

QPQQSTSRIG+NQQLP PP+QWCSSRT I + QF P MRN Q+Q+R

Sbjct 1434 --------QPQQSTSRIGMNQQLPIPPVQWCSSRT-NMIPDTQFTPPMRN----KQDQIR 1480

Query 1500 SPQKVLSPVIPNSPQEESRGYGSKAVKNEETLQSPGNEGNQKSIKPNQVPRR-PAKVKSR 1558

SPQKVLSPV+PNSPQEESRGYG K VKNEET EGNQK KPNQ RR AK KSR

Sbjct 1481 SPQKVLSPVVPNSPQEESRGYGQKTVKNEETHPLSAEEGNQKP-KPNQSHRRSAAKAKSR 1539

Query 1559 IAANFSVPLPDSK 1571

IAANFSVPLPDSK

Sbjct 1540 IAANFSVPLPDSK 1552

Graphical representation


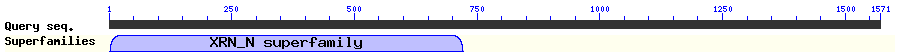


**DNA/RNA non-specific endonuclease isoform 1**

>TRINITY_DN25069_c2_g1_i1 length= 2225 nt

ATGATATTTCAGAAAAAAATCTGGATGATTGATCATTCTACTAAATAAATACAGACATGTTGACATAGTATGGTGAATAGAATACCAAGAAAATGAACATTGAAAAAAATAAAATAAAATAAAAAAACGTGTTTCTTTTCACAAGCTTTTAGGATATAATTATTGAACATACATCAATTAATACAATATGGTTTTAAATTTTTAGCATTTCTGGTTGTACCTATACACCTCTCAAAGTTAGTGAAGAAGAAACCAAACAGGCATCAAATGATCTGAAGGAATATGAAAAAAAGTACGAGCAGCCTAAAAAGTCTAGTGATCATTCAGTGCTCACAAGTGTATGATATCCACTCTACCAGTGGGTTATGAAATGTACTTTCAAGAAAAAGTGGAATGCATATCGAATAATGAAATAATTAAAAAAGAGGGATCAAAGTAATAGTGAATTCGAGTGAGTATGGAGCTTATCAAGGTCAGATAGGTATCTGGTTTGGGCCAAACCAGAAATATATGGAACATTAAGATTTTAGTAAATATATGACTTGTGAAATAATCTGGAGTATTGAGAATTTCACAATGGCTTTGGATTGTAAGGATTGGATACGGGAAGAATAGCAAAATTTACTGGGGCCCCAAAGATCCACTCAGTTTAACTCCGTACGTCAATATATGTTAGATTATATCAAAGTAGATGAGTCTAAGTGCATTTTGATGCTGGATTTTATGTAATATTGAATTTTGTAATACAACTTTTGTTATCATAATTTTATTTTATTTTGTAATATTATATCCAATCAGGAGATGTGATGTAACTAGCCTTTTGGAAATACATTTAGTTTCGGTAGAATTGGTCTAAAAAATGATGACATAAATTTTGGTATTATGTATATTTACAAATCTACCAATCGACAAAGCAGGACATTTACTTTCCCTTGAGGACAGACTTGCAGGTGAGATCAGGCATTTCAGGCACGGCCTTCTTGAGGTCCTGGTACTGGCAGCAGTAGACGTAGCCCTTGGCCAAGTCGTTCTGGAGGACCGGCCATCCGGTTTGAGCGCAGATGTCGTTGCAGATGGGCTTGGGCTTGCTGGTCAGGAAGGGGTTGTTGGTGGCGACGAAGGCGATGCAAGAGTCGTCTTGGGTGTTCCTCACGACCTTCCAGAAGTGTTCGGGTACTGGAAGTCTGGAGTCCTGGAGTTTGATCTCCTTCTGGCCTCTGGCTGCTGGAAGGGTGAGGACTCCTTCGGTTCCTGTGACGACTTGGAGGTCAGCTCCGAGATTCTTAGCTATTTTCCTGGTGTTGTCTTCAACCTTGAGCCAGTTTCCGGCGTTGATGGACTGCCACTGAGGAGCGACGTTGACGTAGAAGTAGGTTGCGAACTGGTGCGCGCTGAAGAGGAAGTCAGCGTCTGGCGCCAGGTGCCCTCTGGCCAGGAAGAAGGTCTTCTTGGAATCGAAGTACTTGTTAGCGTTGGCTGCTCCCAGTTCGCGCTCTAAAACTGCTTGTTGGTTTTTCTGGGCATAGGCGTGCTCTGGGTTGAATCCCTTGAAGAAAATGCTGTCCCCCCTGGAGAAGAGCGGCCGTGCGGTAGTTCTGTAAACCTTGCCTCCCATGATGGCCCCGTGGATCGTGTGGCTGGAGTAGAACGTATGGCTGTTAGCAACGTCGTGACAGGACTCGATCAATGGAATCCATTTTTCCATATCGAAGCCGATCTCTACGACGATGCCCTTTCCATTGGCGCACTTCCTCTGGGTCGGCCTGGAAGTGGCGCTGGCCCTGGAGCTGCAGTCGAGGTCCAAGGAGGAGTAGACCGAGCCGTCGATGGAGAGCTTGTTCCCCGCCTGGCAGGAGGCTGAGGACTGCTCTGTGTTAGTGGCAGCGATGTGGTTCTTCTTACCGGGGCAGGATATTACAAGTTGCTCTCCTTCCCTCAGTGCGATGATTCCCCTCTTTCCCTCCATTTCAGGTAAGACAAGGTCCAAAGAACCAGATGGAGTTTTCTGAAGAAAGAGAGGTTCATTTTTCTTGGGCAAGTCGGTATTCAAGTCCAAAATACAAGCACCCCCGACAGGGTCAACCCTCGAGACCACCCTTGCCTCTGCAGACAGGAGACAGGCCGCCAACGATAGGAGAACAAGTCCGATCATCTTGAAGGAACTAGTTCATCCTGGATCCGTACCCACTTTTTATAGTTTAGCGGAACAGTTGAGTCAATAGAAG

Protein: RF -2: -2152 -> -923 (409 aa)

Comparison with *Halyomorpha halys*, PREDICTED: uncharacterized protein LOC106684787 – Sequence ID: XP_024218583.1

E: 6e-172; bits= 490

Query 145 EIGFDMEKWIPLIESCHDVANSHTFYSSHTIHGAIMGGKVYRTTARPLFSRGDSIFFKGF 204

E+G + WIP+IESCHDV NS+TFYS+HT+HGAIMGGKVYRTTARPLFSRG SIFFKGF

Sbjct 101 EVG---DSWIPMIESCHDVENSNTFYSTHTVHGAIMGGKVYRTTARPLFSRGLSIFFKGF 157

Query 205 NPEHAYAQKNQQAVLERELGAANANKYFDSKKTFFLARGHLAPDADFLFSAHQFATYFYV 264

NPE AY QKNQQAVL R+LG ANANKYFDSKKTF+LARGHLAPDADFLFSAHQF TYFYV

Sbjct 158 NPETAYTQKNQQAVLARDLGTANANKYFDSKKTFYLARGHLAPDADFLFSAHQFLTYFYV 217

Query 265 NVAPQWQSINAGNWLKVEDNTRKIAKNLGADLQVVTGTEGVLTLPAARGQKEIKLQDSRL 324

NVAPQWQSINAGNWL+VEDNTRKIAK+LGADLQ+VTGTEG+LTLP+ +G+KEI+LQ S+L

Sbjct 218 NVAPQWQSINAGNWLRVEDNTRKIAKSLGADLQIVTGTEGILTLPSTKGEKEIRLQSSKL 277

Query 325 PVPEHFWKVVRNTQDDSCIAFVATNNPFLTSKPKPICNDICAQTGWPVLQNDLAKGYVYC 384

PVPEHFWKV+RNTQDDSCIAFV+TNNPFLTS PK +C D+C+Q GWPVLQNDL+KGYVYC

Sbjct 278 PVPEHFWKVLRNTQDDSCIAFVSTNNPFLTSAPKTLCQDVCSQNGWPVLQNDLSKGYVYC 337

Query 385 CQYQDLKKAVPEMPDLTCKSVLKGK 409

C+YQD+KKA+PEMP+LTCKSVLKGK

Sbjct 338 CRYQDIKKAIPEMPNLTCKSVLKGK 362

Graphical representation


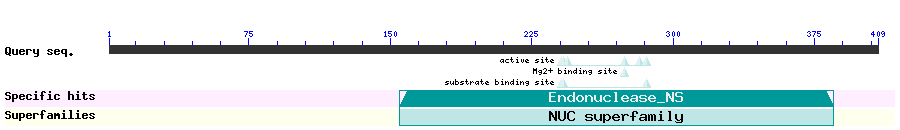


**DNA/RNA non-specific endonuclease isoform 3**

>TRINITY_DN25069_c2_g1_i3 length= 1530 nt

CTTCCAAAGTCAATCATTTCAAAATGTGTGGATGCGGATGAAATAGGAGTTTTTATTAGCTTTTGCTTGATTGGTGAGTGCATTTTACCGATTGGTATGTTTTACCTTTATGACATCATCTACTCCGCAACCTCTACTACTCAACCTGGTGCATTCTATTTTGCAAGTGCAGCTACTATTTTAGTTTGTGTGATATTTTACAGCATTTCTGGTTGTACCTATACACCTCTCAAAGTTAGTGAAGAAGAAACCAAACAGGCATCAAATGATCTGAAGGAATATGAAAAAAAGTACGAGCAGCCTAAAAAGTCTAGTGATCATTCAGTGCTCACAAGTGTATGATATCCACTCTACCAGTGGGTTATGAAATGTACTTTCAAGAAAAAGTGGAATGCATATCGAATAATGAAATAATTAAAAAAGAGGGATCAAAGTAATAGTGAATTCGAGTGAGTATGGAGCTTATCAAGGTCAGATAGGTATCTGGTTTGGGCCAAATCAACAACACATGGGATATTTAGATCTTTATGGATATGTTTGTTTGTGATATAAGAATAATGAATTTTTAAATAATTAGTTAATACATTGTATTTTTTTAGAGTTAAGTGGACCAAAATATCATCCCGTTCTCCTGGAAATCCTTCATAAAAACTCTCATTTCTTTTTTATTCTGTCATTGTTCAAACAATCAAAAGTGACCTTTGTTTCCATATTATTAACCAGCTGATGCCAGAAATAAGTCGCTCTTACTAAAATAAACTCAGTACCAACCCAAACCCATTTGTTATAAACATTGTTTTCCTAAATTATCTCGATAAACTTTGTTAATGATAATAATGCAAAGATATTATACAAATAGTACGCTATCTATTCTATATCTTAACTATTCTAATTAGCTATCCCCACTATGCATTGTGTACACCACACAATTAAATGCAATCTCTTCTGCATTCCTGGCATCCTTTTAGAACATAAGTAAATACATACAAATCAAAATTATATCTAAATCTTTCAGTGCATGAAAAGAACTAAAAGTGTTTATTCAGCCTATGTGCTACATCATGAAAATTATCATCATGATTGAAAGGTTTGGCAAGAAAATTTACATGATAAGATAACCAAGCCATGTTTGAATTATAAGGAACAAGATGGCTGATGAAGCAGCCCTTATGCTATCTTATCCGTATGGCTTTCTATAATAAATAAAGGGAAATAAGCAAAAAAGAAAAAATTAAAAAATAATGGCGAACAAGGTGGGATGAAAGTCCACCTACAAAATAGAAAAAATAAAAAATAAATAAAATACCAACTGTAGAGTCCTGATTGAGAATAGGTCATACATGAATGACTAATGAGTTCATGATAGCAAAAGGGAATGAAATGTTTAATTACAACTGCCTCAAAATAAAATATTTACTTGAACAGTGTTCTTCATTTACTAGAATATTATTACAGAAGGAAATTCAAACAGATAATGCAGTAGTTAATGGAAACAGTGAAACAACATGTCAAACAGCTCTAAATTAATTG

Protein: RF 1: 97 -> 342 (81 aa)

Comparison with *Halyomorpha halys*, PREDICTED: uncharacterized protein LOC106691872– Sequence ID: XP_014293261.1

E: 2e-18; bits= 83.6

Query 1 MFYLYDIIYSATSTTQPGAFYFASAATILVCVIFYSISGCTYTPLKVSEEETKQASNDLK 60

+FYLYD IY+ATSTT PGAF+ ASA V +IFYSI+ C YTPL V++EE K+ S+DL+

Sbjct 389 IFYLYDYIYTATSTTLPGAFFLASAVIAAVSLIFYSITACIYTPLNVNDEEVKKISSDLE 448

Query 61 EYEKKYEQPKK 71

+ EK+ + +K

Sbjct 449 KCEKESQLQEK 459

Graphical representation

**No putative conserved domains have been detected**

**DNA/RNA non-specific endonuclease isoform 4**

>TRINITY_DN25069_c2_g1_i4 length= 2151nt

CTTCCAAAGTCAATCATTTCAAAATGTGTGGATGCGGATGAAATAGGAGTTTTTATTAGCTTTTGCTTGATTGGTGAGTGCATTTTACCGATTGGTATGTTTTACCTTTATGACATCATCTACTCCGCAACCTCTACTACTCAACCTGGTGCATTCTATTTTGCAAGTGCAGCTACTATTTTAGTTTGTGTGATATTTTACAGCATTTCTGGTTGTACCTATACACCTCTCAAAGTTAGTGAAGAAGAAACCAAACAGGCATCAAATGATCTGAAGGAATATGAAAAAAAGTACGAGCAGCCTAAAAAGTCTAGTGATCATTCAGTGCTCACAAGTGTATGATATCCACTCTACCAGTGGGTTATGAAATGTACTTTCAAGAAAAAGTGGAATGCATATCGAATAATGAAATAATTAAAAAAGAGGGATCAAAGTAATAGTGAATTCGAGTGAGTATGGAGCTTATCAAGGTCAGATAGGTATCTGGTTTGGGCCAAACCAGAAATATATGGAACATTAAGATTTTAGTAAATATATGACTTGTGAAATAATCTGGAGTATTGAGAATTTCACAATGGCTTTGGATTGTAAGGATTGGATACGGGAAGAATAGCAAAATTTACTGGGGCCCCAAAGATCCACTCAGTTTAACTCCGTACGTCAATATATGTTAGATTATATCAAAGTAGATGAGTCTAAGTGCATTTTGATGCTGGATTTTATGTAATATTGAATTTTGTAATACAACTTTTGTTATCATAATTTTATTTTATTTTGTAATATTATATCCAATCAGGAGATGTGATGTAACTAGCCTTTTGGAAATACATTTAGTTTCGGTAGAATTGGTCTAAAAAATGATGACATAAATTTTGGTATTATGTATATTTACAAATCTACCAATCGACAAAGCAGGACATTTACTTTCCCTTGAGGACAGACTTGCAGGTGAGATCAGGCATTTCAGGCACGGCCTTCTTGAGGTCCTGGTACTGGCAGCAGTAGACGTAGCCCTTGGCCAAGTCGTTCTGGAGGACCGGCCATCCGGTTTGAGCGCAGATGTCGTTGCAGATGGGCTTGGGCTTGCTGGTCAGGAAGGGGTTGTTGGTGGCGACGAAGGCGATGCAAGAGTCGTCTTGGGTGTTCCTCACGACCTTCCAGAAGTGTTCGGGTACTGGAAGTCTGGAGTCCTGGAGTTTGATCTCCTTCTGGCCTCTGGCTGCTGGAAGGGTGAGGACTCCTTCGGTTCCTGTGACGACTTGGAGGTCAGCTCCGAGATTCTTAGCTATTTTCCTGGTGTTGTCTTCAACCTTGAGCCAGTTTCCGGCGTTGATGGACTGCCACTGAGGAGCGACGTTGACGTAGAAGTAGGTTGCGAACTGGTGCGCGCTGAAGAGGAAGTCAGCGTCTGGCGCCAGGTGCCCTCTGGCCAGGAAGAAGGTCTTCTTGGAATCGAAGTACTTGTTAGCGTTGGCTGCTCCCAGTTCGCGCTCTAAAACTGCTTGTTGGTTTTTCTGGGCATAGGCGTGCTCTGGGTTGAATCCCTTGAAGAAAATGCTGTCCCCCCTGGAGAAGAGCGGCCGTGCGGTAGTTCTGTAAACCTTGCCTCCCATGATGGCCCCGTGGATCGTGTGGCTGGAGTAGAACGTATGGCTGTTAGCAACGTCGTGACAGGACTCGATCAATGGAATCCATTTTTCCATATCGAAGCCGATCTCTACGACGATGCCCTTTCCATTGGCGCACTTCCTCTGGGTCGGCCTGGAAGTGGCGCTGGCCCTGGAGCTGCAGTCGAGGTCCAAGGAGGAGTAGACCGAGCCGTCGATGGAGAGCTTGTTCCCCGCCTGGCAGGAGGCTGAGGACTGCTCTGTGTTAGTGGCAGCGATGTGGTTCTTCTTACCGGGGCAGGATATTACAAGTTGCTGAATGATGTGATGGCATTATTTTCTCCTAATAATAACCAGAAACCTTTATCCTGACAAGACTTATAAATTCATCAAGTCATCCACTGCTAGCTTCAAAATCGTTTTATCCGCTGATTTTAATATAGAATAGTTAAGCTTTTTCGTTTATTTTTTTTCGAATTAGTGTTTACCACCGCCGTTCTTCAATGAGCCATAAGCGATTTTCGTGAGAGGACCGGTCTATAAT

Protein: RF -2: -1703 -> -921 (260 aa)

Comparison with *Halyomorpha halys*, PREDICTED: uncharacterized protein LOC106684787 – Sequence ID: XP_024218583.1

E: 8e-173; bits= 486

Query 2 EKWIPLIESCHDVANSHTFYSSHTIHGAIMGGKVYRTTARPLFSRGDSIFFKGFNPEHAY 61

+ WIP+IESCHDV NS+TFYS+HT+HGAIMGGKVYRTTARPLFSRG SIFFKGFNPE AY

Sbjct 104 DSWIPMIESCHDVENSNTFYSTHTVHGAIMGGKVYRTTARPLFSRGLSIFFKGFNPETAY 163

Query 62 AQKNQQAVLERELGAANANKYFDSKKTFFLARGHLAPDADFLFSAHQFATYFYVNVAPQW 121

QKNQQAVL R+LG ANANKYFDSKKTF+LARGHLAPDADFLFSAHQF TYFYVNVAPQW

Sbjct 164 TQKNQQAVLARDLGTANANKYFDSKKTFYLARGHLAPDADFLFSAHQFLTYFYVNVAPQW 223

Query 122 QSINAGNWLKVEDNTRKIAKNLGADLQVVTGTEGVLTLPAARGQKEIKLQDSRLPVPEHF 181

QSINAGNWL+VEDNTRKIAK+LGADLQ+VTGTEG+LTLP+ +G+KEI+LQ S+LPVPEHF

Sbjct 224 QSINAGNWLRVEDNTRKIAKSLGADLQIVTGTEGILTLPSTKGEKEIRLQSSKLPVPEHF 283

Query 182 WKVVRNTQDDSCIAFVATNNPFLTSKPKPICNDICAQTGWPVLQNDLAKGYVYCCQYQDL 241

WKV+RNTQDDSCIAFV+TNNPFLTS PK +C D+C+Q GWPVLQNDL+KGYVYCC+YQD+

Sbjct 284 WKVLRNTQDDSCIAFVSTNNPFLTSAPKTLCQDVCSQNGWPVLQNDLSKGYVYCCRYQDI 343

Query 242 KKAVPEMPDLTCKSVLKGK 260

KKA+PEMP+LTCKSVLKGK

Sbjct 344 KKAIPEMPNLTCKSVLKGK 362

Graphical representation


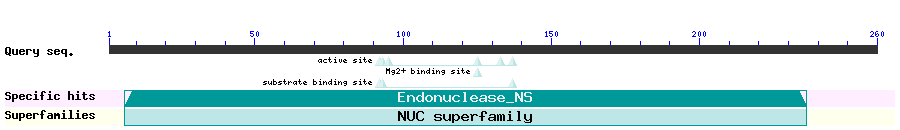


**DNA/RNA non-specific endonuclease isoform 6**

>TRINITY_DN25069_c2_g1_i6 length= 2370 nt

CTTCCAAAGTCAATCATTTCAAAATGTGTGGATGCGGATGAAATAGGAGTTTTTATTAGCTTTTGCTTGATTGGTGAGTGCATTTTACCGATTGGTATGTTTTACCTTTATGACATCATCTACTCCGCAACCTCTACTACTCAACCTGGTGCATTCTATTTTGCAAGTGCAGCTACTATTTTAGTTTGTGTGATATTTTACAGCATTTCTGGTTGTACCTATACACCTCTCAAAGTTAGTGAAGAAGAAACCAAACAGGCATCAAATGATCTGAAGGAATATGAAAAAAAGTACGAGCAGCCTAAAAAGTCTAGTGATCATTCAGTGCTCACAAGTGTATGATATCCACTCTACCAGTGGGTTATGAAATGTACTTTCAAGAAAAAGTGGAATGCATATCGAATAATGAAATAATTAAAAAAGAGGGATCAAAGTAATAGTGAATTCGAGTGAGTATGGAGCTTATCAAGGTCAGATAGGTATCTGGTTTGGGCCAAACCAGAAATATATGGAACATTAAGATTTTAGTAAATATATGACTTGTGAAATAATCTGGAGTATTGAGAATTTCACAATGGCTTTGGATTGTAAGGATTGGATACGGGAAGAATAGCAAAATTTACTGGGGCCCCAAAGATCCACTCAGTTTAACTCCGTACGTCAATATATGTTAGATTATATCAAAGTAGATGAGTCTAAGTGCATTTTGATGCTGGATTTTATGTAATATTGAATTTTGTAATACAACTTTTGTTATCATAATTTTATTTTATTTTGTAATATTATATCCAATCAGGAGATGTGATGTAACTAGCCTTTTGGAAATACATTTAGTTTCGGTAGAATTGGTCTAAAAAATGATGACATAAATTTTGGTATTATGTATATTTACAAATCTACCAATCGACAAAGCAGGACATTTACTTTCCCTTGAGGACAGACTTGCAGGTGAGATCAGGCATTTCAGGCACGGCCTTCTTGAGGTCCTGGTACTGGCAGCAGTAGACGTAGCCCTTGGCCAAGTCGTTCTGGAGGACCGGCCATCCGGTTTGAGCGCAGATGTCGTTGCAGATGGGCTTGGGCTTGCTGGTCAGGAAGGGGTTGTTGGTGGCGACGAAGGCGATGCAAGAGTCGTCTTGGGTGTTCCTCACGACCTTCCAGAAGTGTTCGGGTACTGGAAGTCTGGAGTCCTGGAGTTTGATCTCCTTCTGGCCTCTGGCTGCTGGAAGGGTGAGGACTCCTTCGGTTCCTGTGACGACTTGGAGGTCAGCTCCGAGATTCTTAGCTATTTTCCTGGTGTTGTCTTCAACCTTGAGCCAGTTTCCGGCGTTGATGGACTGCCACTGAGGAGCGACGTTGACGTAGAAGTAGGTTGCGAACTGGTGCGCGCTGAAGAGGAAGTCAGCGTCTGGCGCCAGGTGCCCTCTGGCCAGGAAGAAGGTCTTCTTGGAATCGAAGTACTTGTTAGCGTTGGCTGCTCCCAGTTCGCGCTCTAAAACTGCTTGTTGGTTTTTCTGGGCATAGGCGTGCTCTGGGTTGAATCCCTTGAAGAAAATGCTGTCCCCCCTGGAGAAGAGCGGCCGTGCGGTAGTTCTGTAAACCTTGCCTCCCATGATGGCCCCGTGGATCGTGTGGCTGGAGTAGAACGTATGGCTGTTAGCAACGTCGTGACAGGACTCGATCAATGGAATCCATTTTTCCATATCGAAGCCGATCTCTACGACGATGCCCTTTCCATTGGCGCACTTCCTCTGGGTCGGCCTGGAAGTGGCGCTGGCCCTGGAGCTGCAGTCGAGGTCCAAGGAGGAGTAGACCGAGCCGTCGATGGAGAGCTTGTTCCCCGCCTGGCAGGAGGCTGAGGACTGCTCTGTGTTAGTGGCAGCGATGTGGTTCTTCTTACCGGGGCAGGATATTACAAGTTGCTCTCCTTCCCTCAGTGCGATGATTCCCCTCTTTCCCTCCATTTCAGGTAAGACAAGGTCCAAAGAACCAGATGGAGTTTTCTGAAGAAAGAGAGGTTCATTTTTCTTGGGCAAGTCGGTATTCAAGTCCAAAATACAAGCACCCCCTGTATCTATTTTCCGGTTGCGATTATAATCGTTTTTCGGCAAATCCACCGCCTCCTGATCATCGGGCCAAAATATCCCATCAAAAAACCAGCCCAGAGACCTGCCGTCGACCCGACAGAGAGAGAAGAGAAGGAACGCGAAGTGGAGAGTGCCCATACCGACTGACTCCGCTAGCACAAGCTAACTGTTAACTGTACTCCAAACCTCTCGGCATCATCTCTTCTAAATCTTACTTCCACAAGGAACTTCCCATACCATATCCCAGGACCTCAGGGGCAATTCCGTCAACTAGTAATCACAG

Protein: RF -2: -2225 -> -921 (434 aa)

Comparison with *Halyomorpha halys*, PREDICTED: uncharacterized protein LOC106684787 – Sequence ID: XP_024218583.1

E: 4e-170; bits= 486

Query 170 EIGFDMEKWIPLIESCHDVANSHTFYSSHTIHGAIMGGKVYRTTARPLFSRGDSIFFKGF 229

E+G + WIP+IESCHDV NS+TFYS+HT+HGAIMGGKVYRTTARPLFSRG SIFFKGF

Sbjct 101 EVG---DSWIPMIESCHDVENSNTFYSTHTVHGAIMGGKVYRTTARPLFSRGLSIFFKGF 157

Query 230 NPEHAYAQKNQQAVLERELGAANANKYFDSKKTFFLARGHLAPDADFLFSAHQFATYFYV 289

NPE AY QKNQQAVL R+LG ANANKYFDSKKTF+LARGHLAPDADFLFSAHQF TYFYV

Sbjct 158 NPETAYTQKNQQAVLARDLGTANANKYFDSKKTFYLARGHLAPDADFLFSAHQFLTYFYV 217

Query 290 NVAPQWQSINAGNWLKVEDNTRKIAKNLGADLQVVTGTEGVLTLPAARGQKEIKLQDSRL 349

NVAPQWQSINAGNWL+VEDNTRKIAK+LGADLQ+VTGTEG+LTLP+ +G+KEI+LQ S+L

Sbjct 218 NVAPQWQSINAGNWLRVEDNTRKIAKSLGADLQIVTGTEGILTLPSTKGEKEIRLQSSKL 277

Query 350 PVPEHFWKVVRNTQDDSCIAFVATNNPFLTSKPKPICNDICAQTGWPVLQNDLAKGYVYC 409

PVPEHFWKV+RNTQDDSCIAFV+TNNPFLTS PK +C D+C+Q GWPVLQNDL+KGYVYC

Sbjct 278 PVPEHFWKVLRNTQDDSCIAFVSTNNPFLTSAPKTLCQDVCSQNGWPVLQNDLSKGYVYC 337

Query 410 CQYQDLKKAVPEMPDLTCKSVLKGK 434

C+YQD+KKA+PEMP+LTCKSVLKGK

Sbjct 338 CRYQDIKKAIPEMPNLTCKSVLKGK 362

Graphical representation


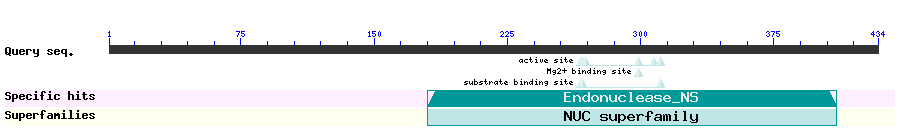


**DNA/RNA non-specific endonuclease isoform 7**

>TRINITY_DN25069_c2_g1_i7 length= 2223 nt

CTTCCAAAGTCAATCATTTCAAAATGTGTGGATGCGGATGAAATAGGAGTTTTTATTAGCTTTTGCTTGATTGGTGAGTGCATTTTACCGATTGGTATGTTTTACCTTTATGACATCATCTACTCCGCAACCTCTACTACTCAACCTGGTGCATTCTATTTTGCAAGTGCAGCTACTATTTTAGTTTGTGTGATATTTTACAGCATTTCTGGTTGTACCTATACACCTCTCAAAGTTAGTGAAGAAGAAACCAAACAGGCATCAAATGATCTGAAGGAATATGAAAAAAAGTACGAGCAGCCTAAAAAGTCTAGTGATCATTCAGTGCTCACAAGTGTATGATATCCACTCTACCAGTGGGTTATGAAATGTACTTTCAAGAAAAAGTGGAATGCATATCGAATAATGAAATAATTAAAAAAGAGGGATCAAAGTAATAGTGAATTCGAGTGAGTATGGAGCTTATCAAGGTCAGATAGGTATCTGGTTTGGGCCAAACCAGAAATATATGGAACATTAAGATTTTAGTAAATATATGACTTGTGAAATAATCTGGAGTATTGAGAATTTCACAATGGCTTTGGATTGTAAGGATTGGATACGGGAAGAATAGCAAAATTTACTGGGGCCCCAAAGATCCACTCAGTTTAACTCCGTACGTCAATATATGTTAGATTATATCAAAGTAGATGAGTCTAAGTGCATTTTGATGCTGGATTTTATGTAATATTGAATTTTGTAATACAACTTTTGTTATCATAATTTTATTTTATTTTGTAATATTATATCCAATCAGGAGATGTGATGTAACTAGCCTTTTGGAAATACATTTAGTTTCGGTAGAATTGGTCTAAAAAATGATGACATAAATTTTGGTATTATGTATATTTACAAATCTACCAATCGACAAAGCAGGACATTTACTTTCCCTTGAGGACAGACTTGCAGGTGAGATCAGGCATTTCAGGCACGGCCTTCTTGAGGTCCTGGTACTGGCAGCAGTAGACGTAGCCCTTGGCCAAGTCGTTCTGGAGGACCGGCCATCCGGTTTGAGCGCAGATGTCGTTGCAGATGGGCTTGGGCTTGCTGGTCAGGAAGGGGTTGTTGGTGGCGACGAAGGCGATGCAAGAGTCGTCTTGGGTGTTCCTCACGACCTTCCAGAAGTGTTCGGGTACTGGAAGTCTGGAGTCCTGGAGTTTGATCTCCTTCTGGCCTCTGGCTGCTGGAAGGGTGAGGACTCCTTCGGTTCCTGTGACGACTTGGAGGTCAGCTCCGAGATTCTTAGCTATTTTCCTGGTGTTGTCTTCAACCTTGAGCCAGTTTCCGGCGTTGATGGACTGCCACTGAGGAGCGACGTTGACGTAGAAGTAGGTTGCGAACTGGTGCGCGCTGAAGAGGAAGTCAGCGTCTGGCGCCAGGTGCCCTCTGGCCAGGAAGAAGGTCTTCTTGGAATCGAAGTACTTGTTAGCGTTGGCTGCTCCCAGTTCGCGCTCTAAAACTGCTTGTTGGTTTTTCTGGGCATAGGCGTGCTCTGGGTTGAATCCCTTGAAGAAAATGCTGTCCCCCCTGGAGAAGAGCGGCCGTGCGGTAGTTCTGTAAACCTTGCCTCCCATGATGGCCCCGTGGATCGTGTGGCTGGAGTAGAACGTATGGCTGTTAGCAACGTCGTGACAGGACTCGATCAATGGAATCCATTTTTCCATATCGAAGCCGATCTCTACGACGATGCCCTTTCCATTGGCGCACTTCCTCTGGGTCGGCCTGGAAGTGGCGCTGGCCCTGGAGCTGCAGTCGAGGTCCAAGGAGGAGTAGACCGAGCCGTCGATGGAGAGCTTGTTCCCCGCCTGGCAGGAGGCTGAGGACTGCTCTGTGTTAGTGGCAGCGATGTGGTTCTTCTTACCGGGGCAGGATATTACAAGTTGCTCTCCTTCCCTCAGTGCGATGATTCCCCTCTTTCCCTCCATTTCAGGTAAGACAAGGTCCAAAGAACCAGATGGAGTTTTCTGAAGAAAGAGAGGTTCATTTTTCTTGGGCAAGTCGGTATTCAAGTCCAAAATACAAGCACCCCCGACAGGGTCAACCCTCGAGACCACCCTTGCCTCTGCAGACAGGAGACAGGCCGCCAACGATAGGAGAACAAGTCCGATCATCTTGAAGGAACTAGTTCATCCTGGATCCGTACCCACTTTTTATAGTTTAGCGGAACAGTTGAGTCAATAGAAG

Protein: RF -2: -2150 -> -921 (409 aa)

Range 1:

Comparison with *Halyomorpha halys*, PREDICTED: uncharacterized protein LOC106684787 – Sequence ID: XP_024218583.1

E: 6e-172; bits= 490

Query 145 EIGFDMEKWIPLIESCHDVANSHTFYSSHTIHGAIMGGKVYRTTARPLFSRGDSIFFKGF 204

E+G + WIP+IESCHDV NS+TFYS+HT+HGAIMGGKVYRTTARPLFSRG SIFFKGF

Sbjct 101 EVG---DSWIPMIESCHDVENSNTFYSTHTVHGAIMGGKVYRTTARPLFSRGLSIFFKGF 157

Query 205 NPEHAYAQKNQQAVLERELGAANANKYFDSKKTFFLARGHLAPDADFLFSAHQFATYFYV 264

NPE AY QKNQQAVL R+LG ANANKYFDSKKTF+LARGHLAPDADFLFSAHQF TYFYV

Sbjct 158 NPETAYTQKNQQAVLARDLGTANANKYFDSKKTFYLARGHLAPDADFLFSAHQFLTYFYV 217

Query 265 NVAPQWQSINAGNWLKVEDNTRKIAKNLGADLQVVTGTEGVLTLPAARGQKEIKLQDSRL 324

NVAPQWQSINAGNWL+VEDNTRKIAK+LGADLQ+VTGTEG+LTLP+ +G+KEI+LQ S+L

Sbjct 218 NVAPQWQSINAGNWLRVEDNTRKIAKSLGADLQIVTGTEGILTLPSTKGEKEIRLQSSKL 277

Query 325 PVPEHFWKVVRNTQDDSCIAFVATNNPFLTSKPKPICNDICAQTGWPVLQNDLAKGYVYC 384

PVPEHFWKV+RNTQDDSCIAFV+TNNPFLTS PK +C D+C+Q GWPVLQNDL+KGYVYC

Sbjct 278 PVPEHFWKVLRNTQDDSCIAFVSTNNPFLTSAPKTLCQDVCSQNGWPVLQNDLSKGYVYC 337

Query 385 CQYQDLKKAVPEMPDLTCKSVLKGK 409

C+YQD+KKA+PEMP+LTCKSVLKGK

Sbjct 338 CRYQDIKKAIPEMPNLTCKSVLKGK 362

Range 2:

Comparison with *Halyomorpha halys*, PREDICTED: uncharacterized protein LOC106684787 – Sequence ID: XP_024218583.1

E: 3e-19; bits= 94.7

Query 145 EIGFDMEKWIPLIESCHDVANSHTFYSSHTIHGAIMGGKVYRTTARPLFSRGDSIFFKGF 204

E+G + WIP+IESCHDV NS+TFYS+HT+HGAIMGGKVYRTTARPLFSRG SIFFKGF

Sbjct 101 EVG---DSWIPMIESCHDVENSNTFYSTHTVHGAIMGGKVYRTTARPLFSRGLSIFFKGF 157

Query 205 NPEHAYAQKNQQAVLERELGAANANKYFDSKKTFFLARGHLAPDADFLFSAHQFATYFYV 264

NPE AY QKNQQAVL R+LG ANANKYFDSKKTF+LARGHLAPDADFLFSAHQF TYFYV

Sbjct 158 NPETAYTQKNQQAVLARDLGTANANKYFDSKKTFYLARGHLAPDADFLFSAHQFLTYFYV 217

Query 265 NVAPQWQSINAGNWLKVEDNTRKIAKNLGADLQVVTGTEGVLTLPAARGQKEIKLQDSRL 324

NVAPQWQSINAGNWL+VEDNTRKIAK+LGADLQ+VTGTEG+LTLP+ +G+KEI+LQ S+L

Sbjct 218 NVAPQWQSINAGNWLRVEDNTRKIAKSLGADLQIVTGTEGILTLPSTKGEKEIRLQSSKL 277

Query 325 PVPEHFWKVVRNTQDDSCIAFVATNNPFLTSKPKPICNDICAQTGWPVLQNDLAKGYVYC 384

PVPEHFWKV+RNTQDDSCIAFV+TNNPFLTS PK +C D+C+Q GWPVLQNDL+KGYVYC

Sbjct 278 PVPEHFWKVLRNTQDDSCIAFVSTNNPFLTSAPKTLCQDVCSQNGWPVLQNDLSKGYVYC 337

Query 385 CQYQDLKKAVPEMPDLTCKSVLKGK 409

C+YQD+KKA+PEMP+LTCKSVLKGK

Sbjct 338 CRYQDIKKAIPEMPNLTCKSVLKGK 362

Graphical representation


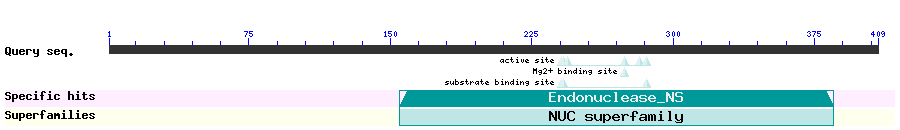


**DNA/RNA non-specific endonuclease isoform 9**

>TRINITY_DN25069_c2_g1_i9 length= 2372 nt

ATGATATTTCAGAAAAAAATCTGGATGATTGATCATTCTACTAAATAAATACAGACATGTTGACATAGTATGGTGAATAGAATACCAAGAAAATGAACATTGAAAAAAATAAAATAAAATAAAAAAACGTGTTTCTTTTCACAAGCTTTTAGGATATAATTATTGAACATACATCAATTAATACAATATGGTTTTAAATTTTTAGCATTTCTGGTTGTACCTATACACCTCTCAAAGTTAGTGAAGAAGAAACCAAACAGGCATCAAATGATCTGAAGGAATATGAAAAAAAGTACGAGCAGCCTAAAAAGTCTAGTGATCATTCAGTGCTCACAAGTGTATGATATCCACTCTACCAGTGGGTTATGAAATGTACTTTCAAGAAAAAGTGGAATGCATATCGAATAATGAAATAATTAAAAAAGAGGGATCAAAGTAATAGTGAATTCGAGTGAGTATGGAGCTTATCAAGGTCAGATAGGTATCTGGTTTGGGCCAAACCAGAAATATATGGAACATTAAGATTTTAGTAAATATATGACTTGTGAAATAATCTGGAGTATTGAGAATTTCACAATGGCTTTGGATTGTAAGGATTGGATACGGGAAGAATAGCAAAATTTACTGGGGCCCCAAAGATCCACTCAGTTTAACTCCGTACGTCAATATATGTTAGATTATATCAAAGTAGATGAGTCTAAGTGCATTTTGATGCTGGATTTTATGTAATATTGAATTTTGTAATACAACTTTTGTTATCATAATTTTATTTTATTTTGTAATATTATATCCAATCAGGAGATGTGATGTAACTAGCCTTTTGGAAATACATTTAGTTTCGGTAGAATTGGTCTAAAAAATGATGACATAAATTTTGGTATTATGTATATTTACAAATCTACCAATCGACAAAGCAGGACATTTACTTTCCCTTGAGGACAGACTTGCAGGTGAGATCAGGCATTTCAGGCACGGCCTTCTTGAGGTCCTGGTACTGGCAGCAGTAGACGTAGCCCTTGGCCAAGTCGTTCTGGAGGACCGGCCATCCGGTTTGAGCGCAGATGTCGTTGCAGATGGGCTTGGGCTTGCTGGTCAGGAAGGGGTTGTTGGTGGCGACGAAGGCGATGCAAGAGTCGTCTTGGGTGTTCCTCACGACCTTCCAGAAGTGTTCGGGTACTGGAAGTCTGGAGTCCTGGAGTTTGATCTCCTTCTGGCCTCTGGCTGCTGGAAGGGTGAGGACTCCTTCGGTTCCTGTGACGACTTGGAGGTCAGCTCCGAGATTCTTAGCTATTTTCCTGGTGTTGTCTTCAACCTTGAGCCAGTTTCCGGCGTTGATGGACTGCCACTGAGGAGCGACGTTGACGTAGAAGTAGGTTGCGAACTGGTGCGCGCTGAAGAGGAAGTCAGCGTCTGGCGCCAGGTGCCCTCTGGCCAGGAAGAAGGTCTTCTTGGAATCGAAGTACTTGTTAGCGTTGGCTGCTCCCAGTTCGCGCTCTAAAACTGCTTGTTGGTTTTTCTGGGCATAGGCGTGCTCTGGGTTGAATCCCTTGAAGAAAATGCTGTCCCCCCTGGAGAAGAGCGGCCGTGCGGTAGTTCTGTAAACCTTGCCTCCCATGATGGCCCCGTGGATCGTGTGGCTGGAGTAGAACGTATGGCTGTTAGCAACGTCGTGACAGGACTCGATCAATGGAATCCATTTTTCCATATCGAAGCCGATCTCTACGACGATGCCCTTTCCATTGGCGCACTTCCTCTGGGTCGGCCTGGAAGTGGCGCTGGCCCTGGAGCTGCAGTCGAGGTCCAAGGAGGAGTAGACCGAGCCGTCGATGGAGAGCTTGTTCCCCGCCTGGCAGGAGGCTGAGGACTGCTCTGTGTTAGTGGCAGCGATGTGGTTCTTCTTACCGGGGCAGGATATTACAAGTTGCTCTCCTTCCCTCAGTGCGATGATTCCCCTCTTTCCCTCCATTTCAGGTAAGACAAGGTCCAAAGAACCAGATGGAGTTTTCTGAAGAAAGAGAGGTTCATTTTTCTTGGGCAAGTCGGTATTCAAGTCCAAAATACAAGCACCCCCTGTATCTATTTTCCGGTTGCGATTATAATCGTTTTTCGGCAAATCCACCGCCTCCTGATCATCGGGCCAAAATATCCCATCAAAAAACCAGCCCAGAGACCTGCCGTCGACCCGACAGAGAGAGAAGAGAAGGAACGCGAAGTGGAGAGTGCCCATACCGACTGACTCCGCTAGCACAAGCTAACTGTTAACTGTACTCCAAACCTCTCGGCATCATCTCTTCTAAATCTTACTTCCACAAGGAACTTCCCATACCATATCCCAGGACCTCAGGGGCAATTCCGTCAACTAGTAATCACAG

Protein: RF -2: -2227 -> -923 (434 aa)

Range 1:

Comparison with *Halyomorpha halys*, PREDICTED: uncharacterized protein LOC106684787 – Sequence ID: XP_024218583.1

E: 5e-170; bits= 486

Query 170 EIGFDMEKWIPLIESCHDVANSHTFYSSHTIHGAIMGGKVYRTTARPLFSRGDSIFFKGF 229

E+G + WIP+IESCHDV NS+TFYS+HT+HGAIMGGKVYRTTARPLFSRG SIFFKGF

Sbjct 101 EVG---DSWIPMIESCHDVENSNTFYSTHTVHGAIMGGKVYRTTARPLFSRGLSIFFKGF 157

Query 230 NPEHAYAQKNQQAVLERELGAANANKYFDSKKTFFLARGHLAPDADFLFSAHQFATYFYV 289

NPE AY QKNQQAVL R+LG ANANKYFDSKKTF+LARGHLAPDADFLFSAHQF TYFYV

Sbjct 158 NPETAYTQKNQQAVLARDLGTANANKYFDSKKTFYLARGHLAPDADFLFSAHQFLTYFYV 217

Query 290 NVAPQWQSINAGNWLKVEDNTRKIAKNLGADLQVVTGTEGVLTLPAARGQKEIKLQDSRL 349

NVAPQWQSINAGNWL+VEDNTRKIAK+LGADLQ+VTGTEG+LTLP+ +G+KEI+LQ S+L

Sbjct 218 NVAPQWQSINAGNWLRVEDNTRKIAKSLGADLQIVTGTEGILTLPSTKGEKEIRLQSSKL 277

Query 350 PVPEHFWKVVRNTQDDSCIAFVATNNPFLTSKPKPICNDICAQTGWPVLQNDLAKGYVYC 409

PVPEHFWKV+RNTQDDSCIAFV+TNNPFLTS PK +C D+C+Q GWPVLQNDL+KGYVYC

Sbjct 278 PVPEHFWKVLRNTQDDSCIAFVSTNNPFLTSAPKTLCQDVCSQNGWPVLQNDLSKGYVYC 337

Query 410 CQYQDLKKAVPEMPDLTCKSVLKGK 434

C+YQD+KKA+PEMP+LTCKSVLKGK

Sbjct 338 CRYQDIKKAIPEMPNLTCKSVLKGK 362

Range 2:

E: 3e-29; bits= 123

Comparison with *Halyomorpha halys*, PREDICTED: uncharacterized protein LOC106684787 – Sequence ID: XP_024218583.1

Query 1 MGTLH-FAFLLF-SLCRVDGRSLGWFFDGIFWPDDQEAVDLPKNDYNRNRKIDTGGACIL 58

M LH +F LF +L RVDGRSLG F D FW D + D Y++ R I GGACIL

Sbjct 1 MRVLHVISFALFCALSRVDGRSLGIFED--FWSDGGDPGDSVGYGYDKQRHIAIGGACIL 58

Query 59 DLNTDLPKKNEPLFLQKTPSGSLDLVLPEMEGKRGIIALREGE 101

DLNTDLPKKNEPLFLQ++ SG LDLVLPE+EG RG+IALREGE

Sbjct 59 DLNTDLPKKNEPLFLQRSSSGDLDLVLPELEGNRGVIALREGE 101

Graphical representation


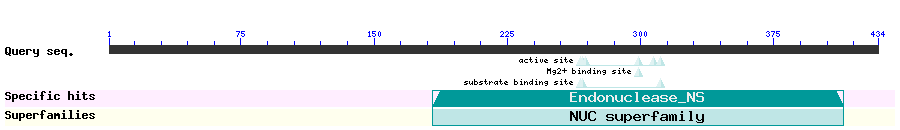


**DNA/RNA non-specific endonuclease isoform 10**

>TRINITY_DN25069_c2_g1_i10 length= 2225 nt

ATGATATTTCAGAAAAAAATCTGGATGATTGATCATTCTACTAAATAAATACAGACATGTTGACATAGTATGGTGAATAGAATACCAAGAAAATGAACATTGAAAAAAATAAAATAAAATAAAAAAACGTGTTTCTTTTCACAAGCTTTTAGGATATAATTATTGAACATACATCAATTAATACAATATGGTTTTAAATTTTTAGCATTTCTGGTTGTACCTATACACCTCTCAAAGTTAGTGAAGAAGAAACCAAACAGGCATCAAATGATCTGAAGGAATATGAAAAAAAGTACGAGCAGCCTAAAAAGTCTAGTGATCATTCAGTGCTCACAAGTGTATGATATCCACTCTACCAGTGGGTTATGAAATGTACTTTCAAGAAAAAGTGGAATGCATATCGAATAATGAAATAATTAAAAAAGAGGGATCAAAGTAATAGTGAATTCGAGTGAGTATGGAGCTTATCAAGGTCAGATAGGTATCTGGTTTGGGCCAAACCAGAAATATATGGAACATTAAGATTTTAGTAAATATATGACTTGTGAAATAATCTGGAGTATTGAGAATTTCACAATGGCTTTGGATTGTAAGGATTGGATACGGGAAGAATAGCAAAATTTACTGGGGCCCCAAAGATCCACTCAGTTTAACTCCGTACGTCAATATATGTTAGATTATATCAAAGTAGATGAGTCTAAGTGCATTTTGATGCTGGATTTTATGTAATATTGAATTTTGTAATACAACTTTTGTTATCATAATTTTATTTTATTTTGTAATATTATATCCAATCAGGAGATGTGATGTAACTAGCCTTTTGGAAATACATTTAGTTTCGGTAGAATTGGTCTAAAAAATGATGACATAAATTTTGGTATTATGTATATTTACAAATCTACCAATCGACAAAGCAGGACATTTACTTTCCCTTGAGGACAGACTTGCAGGTGAGATCAGGCATTTCAGGCACGGCCTTCTTGAGGTCCTGGTACTGGCAGCAGTAGACGTAGCCCTTGGCCAAGTCGTTCTGGAGGACCGGCCATCCGGTTTGAGCGCAGATGTCGTTGCAGATGGGCTTGGGCTTGCTGGTCAGGAAGGGGTTGTTGGTGGCGACGAAGGCGATGCAAGAGTCGTCTTGGGTGTTCCTCACGACCTTCCAGAAGTGTTCGGGTACTGGAAGTCTGGAGTCCTGGAGTTTGATCTCCTTCTGGCCTCTGGCTGCTGGAAGGGTGAGGACTCCTTCGGTTCCTGTGACGACTTGGAGGTCAGCTCCGAGATTCTTAGCTATTTTCCTGGTGTTGTCTTCAACCTTGAGCCAGTTTCCGGCGTTGATGGACTGCCACTGAGGAGCGACGTTGACGTAGAAGTAGGTTGCGAACTGGTGCGCGCTGAAGAGGAAGTCAGCGTCTGGCGCCAGGTGCCCTCTGGCCAGGAAGAAGGTCTTCTTGGAATCGAAGTACTTGTTAGCGTTGGCTGCTCCCAGTTCGCGCTCTAAAACTGCTTGTTGGTTTTTCTGGGCATAGGCGTGCTCTGGGTTGAATCCCTTGAAGAAAATGCTGTCCCCCCTGGAGAAGAGCGGCCGTGCGGTAGTTCTGTAAACCTTGCCTCCCATGATGGCCCCGTGGATCGTGTGGCTGGAGTAGAACGTATGGCTGTTAGCAACGTCGTGACAGGACTCGATCAATGGAATCCATTTTTCCATATCGAAGCCGATCTCTACGACGATGCCCTTTCCATTGGCGCACTTCCTCTGGGTCGGCCTGGAAGTGGCGCTGGCCCTGGAGCTGCAGTCGAGGTCCAAGGAGGAGTAGACCGAGCCGTCGATGGAGAGCTTGTTCCCCGCCTGGCAGGAGGCTGAGGACTGCTCTGTGTTAGTGGCAGCGATGTGGTTCTTCTTACCGGGGCAGGATATTACAAGTTGCTCTCCTTCCCTCAGTGCGATGATTCCCCTCTTTCCCTCCATTTCAGGTAAGACAAGGTCCAAAGAACCAGATGGAGTTTTCTGAAGAAAGAGAGGTTCATTTTTCTTGGGCAAGTCGGTATTCAAGTCCAAAATACAAGCACCCCCGACAGGGTCAACCCTCGAGACCACCCTTGCCTCTGCAGACAGGAGACAGGCCGCCAACGATAGGAGAACAAGTCCGATCATCTTGAAGGAACTAGTTCATCCTGGATCCGTACCCACTTTTTATAGTTTAGCGGAACAGTTGAGTCAATAGAAG

Protein: RF -2: -2152 -> -923 (409 aa)

Range 1:

Comparison with *Halyomorpha halys*, PREDICTED: uncharacterized protein LOC106684787 – Sequence ID: XP_024218583.1

E: 7e-172; bits= 490

Query 145 EIGFDMEKWIPLIESCHDVANSHTFYSSHTIHGAIMGGKVYRTTARPLFSRGDSIFFKGF 204

E+G + WIP+IESCHDV NS+TFYS+HT+HGAIMGGKVYRTTARPLFSRG SIFFKGF

Sbjct 101 EVG---DSWIPMIESCHDVENSNTFYSTHTVHGAIMGGKVYRTTARPLFSRGLSIFFKGF 157

Query 205 NPEHAYAQKNQQAVLERELGAANANKYFDSKKTFFLARGHLAPDADFLFSAHQFATYFYV 264

NPE AY QKNQQAVL R+LG ANANKYFDSKKTF+LARGHLAPDADFLFSAHQF TYFYV

Sbjct 158 NPETAYTQKNQQAVLARDLGTANANKYFDSKKTFYLARGHLAPDADFLFSAHQFLTYFYV 217

Query 265 NVAPQWQSINAGNWLKVEDNTRKIAKNLGADLQVVTGTEGVLTLPAARGQKEIKLQDSRL 324

NVAPQWQSINAGNWL+VEDNTRKIAK+LGADLQ+VTGTEG+LTLP+ +G+KEI+LQ S+L

Sbjct 218 NVAPQWQSINAGNWLRVEDNTRKIAKSLGADLQIVTGTEGILTLPSTKGEKEIRLQSSKL 277

Query 325 PVPEHFWKVVRNTQDDSCIAFVATNNPFLTSKPKPICNDICAQTGWPVLQNDLAKGYVYC 384

PVPEHFWKV+RNTQDDSCIAFV+TNNPFLTS PK +C D+C+Q GWPVLQNDL+KGYVYC

Sbjct 278 PVPEHFWKVLRNTQDDSCIAFVSTNNPFLTSAPKTLCQDVCSQNGWPVLQNDLSKGYVYC 337

Query 385 CQYQDLKKAVPEMPDLTCKSVLKGK 409

C+YQD+KKA+PEMP+LTCKSVLKGK

Sbjct 338 CRYQDIKKAIPEMPNLTCKSVLKGK 362

Range 2:

E: 3e-19; bits= 94.7

Query 27 VGGACILDLNTDLPKKNEPLFLQKTPSGSLDLVLPEMEGKRGIIALREGE 76

+GGACILDLNTDLPKKNEPLFLQ++ SG LDLVLPE+EG RG+IALREGE

Sbjct 52 IGGACILDLNTDLPKKNEPLFLQRSSSGDLDLVLPELEGNRGVIALREGE 101

Graphical representation


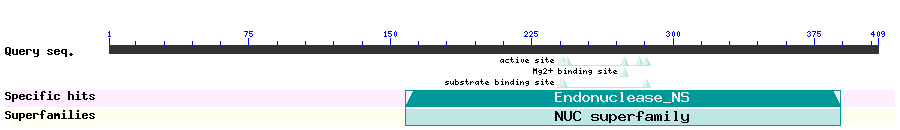


**Small RNA degrading nuclease 1 – SDN1**

>TRINITY_DN22912_c0_g1_i1 length= 2469 nt

GTTAAATAAAGTTTATTTAAATGCTTAGTAAAACAAATATTTCATTTTCCTTTCTTCTCCTAGTCAAATATGTTTTTCAACAGTATGATTGGATTTACCATTCTTGTCATAACGGACTAGGTGAGAGAATAATGATGTGGCACTAATATTCTCAAAGTGCTGTGTGTCTGGACGAAAACCCTTTCGGAAATTGTGCATGGCATCATGATCCTGATGATCTCTCTCTCTTAATTGTGGCCAAATGAGTCGCTTAGAATCAGCAACAGATATTCGTGCCATGCCAGCAGCTTCCCTTGATTCCAGGAGAATCCTAGCTGAATTGGATTCTTCTTTAGATCTTGGGCCTCCCAACACTGCATTTCCCCAACTGAGTGAGTGGTACAGTTTAGCTTGCACTAATTTCATAGAGGCCAGTGCATCCTCAATTGGACTGTGCCCAAGAATATCATCAGTTTGAATTCTTAAAGATAAGAATTCTTCAACGAGGATTCTGAGCTTTGTCTTTCTTTGTCGGATGCCAGACATGTTGAAGATAACTGATGTATCAATGACATATGGATGCATCATCTTCATCGCAGTCAAATCCATGTTCAATGATTGACCTATAAGAATAAGATCAGGAGGACATATGGCACGAATATCTGACTGCACGTCTTCTAAACGAGTTTTCACTGACTTCATCATAGAAGGAGTGATACCAGAAAATTTTGTAAGGTAGTTTTTAATAGGGTTATATGGCTTTACAAGTGTTTCATAAACAATATTGCAATCTTCATCAACAATGCAGATCCGAGTCAATTCACTACCAACAGTAGTAAGACACATTTCACAATCCAAAGCCCACATAGGTGAGTCGTCTGATACTTCTGTATAACCATCACGTGTGAAAACAAAGTCTGAATACCTTCTTCCTAGTTCTCCACTTAGAGGAAGAGGGTAATTTTCCTCAATCATATCCCATGGGCTGAGAACAAGCTGTTTCCTCGAAAACTTATCTTTCGGTCTTATAGACATTGTCTCTTCTCTCTTAATCACAGGACAATCTTCAACAACTTTTTGGCTTTTATTGGTGATTGGGAAAATAGTTTTGAATGTATTAAAGATGTCACCAGAACTGTAAGCTTCTTTTAAGGATCCATACTCAGAAGCAAGTTTTTGTCTTTGGCTGTTTGTCAAAGAAACAAGTGCAAGTTCACTGATCAAATCACCACCATACTGTAACGGAGAAAGGACTTCTAATTGCTTCTCAAATTGTGAAAGGATTTTTAAATCTGCTTCCTTTTCCAGATACTCTTTTAGACCAGCACCCTCAATTATCATAAAAATACGCCGCTCCAATTTCTGGTACTTTTTACAGTTTATCCACCTACTGGGACCTTTTGTCCCCATATGAGCAAAAAGTGAGGTAATGATAAAGTGCTGGAAGTCATCAAAGGGTAACGGCACTCGTTGAGAATCTGGCAAACTTAGTGAAGCAGCTTCACCAACTTCATTCAACTCCAAAAAAGGTCTTTCATGTCTTATCTTTTTTCTCATTTTAAGCAGCTTATTCAACTCAGACAATTGTTCTTCACTAAGTACACTTCGTTCCTGTTTAACATGGACTTCATTTTTGTCACTTCCGGCTTGGCCATTGAGAGGGAAGAGAAAATCACACTTCATTCGCTTAGGAATTGGTTCCTCAAAGTTTTTCTGAAATTTTTTACTATGAAAATCTTTCTGAGGTATGCCACCAGAAGTTGAATTTATGCTTGACTTCTTACGATCTTGATCATTTAAACTACTTATTTCCAACAATGCTTTCAATTTCTTCTTTTTATTTTCTTTCCTTCTAAGTTTTCTAAGATTACGTTTGTTATGCTCCAACATACTGAAAAACCATTAATTTAGATCTTTTTAATTTTTTTTTAGGGTTATAAAACTTTAACTGAAAACAGATACTAGCGTTAGCAATAAAGTGCTACTTTCTTGGCAGAAAATAGCTAACATCATTAAAACTGTTTAAAGATGTAACTATCCGTCAAAAACATATAAAAATTATAGGTGTTACAATTCATCTATCACGTGGATAGATAACCCCCAACACCAAAACATTACCTCCCGCTAACTATGTTGTTAAAGTTTTGCCAACGAGTTAATCCACTAACTCAGAAAATACTTAACAATTTTGTTCTCATGTGAAATTAACTTCGTCAAAAGTGTAAAATGAAAACTTTTGCAATATTATTTTTGGCATTCATGCCCAAAGTAAAATTATTCTACCAAATCGCAAGCAATATCCTAATTTATGTTTTGTACAGTATAAAACAATGACTCTCAAAATAATAACCGCAACTAAATATTCCTGCCCAAGACGGTACCTTTCTTAAAACAGCTGTATTATTTTCTTCTAAAGATCTCGAAACATTAAATTAAAACCTTTCTCCTCTCAGATGTTAAATGGTAATACTTTTACCAAACGAATATTAT

Protein: RF -1: -1869 -> -64 (601 aa)

Comparison with *Halyomorpha halys,* PREDICTED: uncharacterized exonuclease C637.09 isoform X1 - Sequence ID: XP_ [XP_014279339.1](https://www.ncbi.nlm.nih.gov/protein/XP_014279339.1?report=genbank&log$=protalign&blast_rank=1&RID=N8SG7HT3014)

E: 0.0; bits= 895

Query 1 MLEHNKRNLRKLRRKENKKKKLKALLEISSLNDQDRKKSSINSTSGGIPQKDFHSKKFQK 60

MLEHNKRNL+KL+RKENKKKKLKALLEISSLNDQDRKKSSINS S G+P KD SKKFQK

Sbjct 7 MLEHNKRNLKKLQRKENKKKKLKALLEISSLNDQDRKKSSINSASSGLPVKDPSSKKFQK 66

Query 61 NFEEPIPKRMKCDFLFPLNGQAGSDKNEVHVKQERSVLSEEQLSELNKLLKMRKKIRHER 120

NFEEP PKRMK DFLFPLNG AG+D V ++Q+RS+L+EEQ+SELNKLLKMRK+IRHER

Sbjct 67 NFEEPTPKRMKRDFLFPLNGIAGNDDENVQIRQDRSLLTEEQISELNKLLKMRKRIRHER 126

Query 121 PFLELNEVGEAASLSLPDSQRVPLPFDDFQHFIITSLFAHMGTKGPSRWINCKKYQKLER 180

PFLELNEVGEAASLSLP SQRVPLPFDDFQHFI+TSLFAHM TKGPSRWINCKKYQKLER

Sbjct 127 PFLELNEVGEAASLSLPGSQRVPLPFDDFQHFILTSLFAHMSTKGPSRWINCKKYQKLER 186

Query 181 RIFMIIEGAGLKEYLEKEADLKILSQFEKQLEVLSPLQYGGDLISELALVSLTNSQRQKL 240

RIFMIIEGAGLKEYLEKEA+LK LSQFEK+LEVL+PLQYGGDL+ ELALVSLTNSQ+QKL

Sbjct 187 RIFMIIEGAGLKEYLEKEAELKTLSQFEKKLEVLTPLQYGGDLVRELALVSLTNSQKQKL 246

Query 241 ASEYGSLKEAYSSGDIFNTFKTIFPITNKSQKVV-----EDCPVIKREETMSIRPKDKFS 295

SEYGSLKEAY SGD+F TFKTIFPIT K K ED E TM R KDKFS

Sbjct 247 VSEYGSLKEAYKSGDVFKTFKTIFPITAKCNKAAESIRKEDIQANNTESTMYKRQKDKFS 306

Query 296 RKQLVLSPWDMIEENYPLPLSGELGRRYSDFVFTRDGYTEVSDDSPMWALDCEMCLTTVG 355

RK L+LSPW+M EE++PL L E +R++DFVFTRD Y+EVSD+SPMWA+DCEMC T G

Sbjct 307 RKLLLLSPWEMAEEDFPLLLDEERQQRFADFVFTRDSYSEVSDNSPMWAVDCEMCSTAYG 366

Query 356 SELTRICIVDEDCNIVYETLVKPYNPIKNYLTKFSGITPSMMKSVKTRLEDVQSDIRAIC 415

ELTRICIVDE+ NIVYETLVKPYNPI NYLT+ SGITPSM+K+V TRLEDVQ+DIRAIC

Sbjct 367 HELTRICIVDENSNIVYETLVKPYNPIINYLTQHSGITPSMLKNVNTRLEDVQADIRAIC 426

Query 416 PPDLILIGQSLNMDLTAMKMMHPYVIDTSVIFNMSGIRQRKTKLRILVEEFLSLRIQTDD 475

PPD+ILIGQSL+ DL AM+MMHPYVIDTSVIFN+SG++ RKTKL++LVEEFLSLRIQT+D

Sbjct 427 PPDVILIGQSLSNDLKAMQMMHPYVIDTSVIFNLSGVKYRKTKLKVLVEEFLSLRIQTED 486

Query 476 ILGHSPIEDALASMKLVQAKLYHSLSWGNAVLGGPRSKEESNSARILLESREAAGMARIS 535

LGH PIEDA+A+MKLVQ KL H+ +WGNAVL GPRSKEES SAR+LLESREAAGM R+S

Sbjct 487 KLGHCPIEDAVAAMKLVQEKLRHTPTWGNAVLAGPRSKEESRSARVLLESREAAGMGRLS 546

Query 536 VADSKRLIWPQL--RERDHQDHDAMHNFRKGFRPDTQHFENISATSLFSHLVRYDKNG 591

+ +S+R+ W QL ++RD Q+ D ++NF D Q+ +N +TSLF+HLV ++ +

Sbjct 547 LTNSERITWTQLMNKKRDDQNDDGVYNFHP--YSDNQYEDN--STSLFAHLVSHETDA 600

Graphical representation


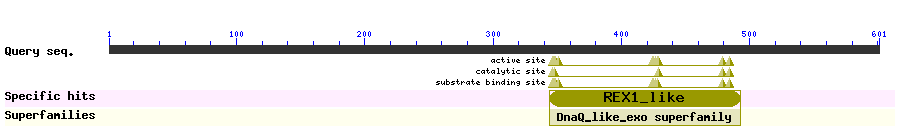


**Nibbler**

>TRINITY_DN28702_c0_g1_i2 length= 3271 nt

ATAACAATTTAGTAAATCAAAATTTTAAAATTCTATTAAAATATGTTTGATATTTTAAATAATCTTATTGTCTTAAAAAGTTATATAACTTTCCTTAAAATTTAATAAATATATATAGATATATATTCTTTTCATTTCATTCTTCGCCAATAATCTGAATGTCAATATCGAAAGGAACACAGATGAGGGTGAATCACAAAAAATGCGGGAAGGGAAGAAATGTTATAAGACGATATTGGTGGTTAGGCTTTATTCTCTCAATGGTGCGCGTGTCAACGGAAGCCGCTATTTGCTCTCGGTCTGCCAGAATACTCGTCCACAATCCTCACACATGCAGATCAAATCAGCCAATTTAATTTGTTCTTCAATGACGAGGTCCAACCTAATTGGAACTCCAGTCGTAGTGGTTGGATGAGAATTCATTCCGTTCTCCACGGGCCCAGTGCCTGCAATCATGTCAGATCTGTAGTAATCATCTTCTTCATCTGAGTCTGTTAACTCGTTGTCAGAGTAACCAGCCGGTTGGCAGTTCGTAGTCTTTGTGTTGTCTTTCTTAGCAAACCAATCCTCAACAGTATTCTTGTCTATCATCACAACATGGTCACTGTTACATTCCTGGCACCTGGTGAAAATATCCTCAGGTTTTACGATAATGTACAGCTGCCTGATGACTTCCCTGTACTGATCTGGGGAGGCACACGACCGCACCAAGTAGCATTTGTCCTTCGATAACTGCTCAGAAATTGTTTTAAAAGGCCAAGAAGAAGACAGTACAATCCTGTTTTCCAATTCAGCGATTTCGGCAATGTCATGTAGCTCAGCTACAACAGTATCTACGCCGTGCCTTCGCAATACGCACGCCAGCATAGCCAGGCTTGGCTCACATACTAGCTTCATCATCTTTGCTTCGGTTGCTGTCGTTCTATACCTCGTTGCTTCTTTATTTTTTTTATTTGATTTTGACTTCTTAGCCTTCCCTTGTTCAGTTTTAGTCATCATATCTTGAACGATATCATCAATAGGTATTTTCCTTTCAGAGCACAGCCTTTTTATAACATCGTAAACTTCAATTAGGCAATAAGCATCCAGCGCAGCATATACCTTTTGGGTCGGCCTTAGTGGACGAGTCTCCCAATTAGAAAATTGTTCTCTCTTGTTGAGCGGCTTTCCAATGCAAAGAGCAACAAGTTGGCTTAAACTACAACTAGAACAGCTCTCATTGGCTTCATAGGGAAACTCGAAATTGTAATCCTTCACAAGTCTATTCCAGAGCGAGGAAAGATCCAAAAAACCGAGCCCAGACATCCGAAATGCGAAATCGATAGGAAAACACTGAGCCAACATAGATGAATCAGCACCCATAGCAAATCCTATTTTGAGGATATCGTGGTTGTTGAGAAATACATGAGCGAACTTCCCCCACAGTTGATGGGAATTGGCTAAAGCGAGTACATCGATGAGAAACACTTTGTCTTCCTTTGCCAGCTGAAGTAGGGCGAGCTCTCCGGAAGGAGCTACCATCGTCGGCTTCCATTCCAAATCGATACCGATTGTAGTAGCTCCATCTATATAAGAATCAATTAGAGACGAAAAGGCTGCTTCCGTATCGATAATAAATATTGAAGAAAGCTGCAGAGGAAACTTGTGATACTGATCGTTCTGGTCTTGTTCGTCTTCTACTTGTTGATTTGAAAGTGGTAAATCATCTTCCCTCCAGCCATTTTCATCAAGAGATCTGACGCTCGGAGGCATATCGGAACGAGGAACAGAGAAGAGGCGAGCACATATCAAGGCTTCAGTTGGGTCTTCTTCTTCATCGAGCCTATTCACTAAGTGGATCATAACCTCTCTTCTCGAACCACAAGCATCTTTTACGAGCTCTCGGAATGCCTCCTTGCTTAAACTTCGGTCATAGTATCTCTTCCGGATGAGAAAGTTGACAGTGCCCATGCCGTTTCTGAGGGTGAGGTTGGGCGTAGTGTCGGGAGGGATATTGTGTATCGAGGCGAATCTCTTCAGCAGCTTACACAGAGGTTTCGTTTGAGATAGCTTGGGTAAAGAAGCATGCTTGACGTTTTCTATCTTCAAGTCTATTATTAGCTGATCGATGAAATTCATAATACTCCTCTGTGACAAAATGTTATCCAAGAATTTAATCAATTCTAAATGATGAGAACGGTTCTTCTTGAGAAAGTCGTCGACAACTGTAAGCTTATCTTGAAAAATGAGCGGAACAATAAAATCAAAAATACCAAATTCAGATTCTAAATCCAACATCATAGACCATTCGCATACTTCTTTGTACATATGTAGCTGAAGATTGTCATTCAGAGATTTCACAAATAAATGCTTGTTTTCTTTAAGTTGATAAACTTCAGTAATATTCTTGCTTATAATCCTATGTCTTTGTCTCGAGGCTACTTGAAATGCTTCCCGTTGTACTTCTTCAGTGAGGCAGTGTTTATAATTATCTCTTCTAATACTAATCCAATTGGCGAATTGTTCAATAATAGTAAATGAAAGACCATTGTGTCGAGACAGGCTATAGTCGGGTGACTGCCTAATTAAAATTAGAGCACTTTCATATGGATTTTCACTCGATATAAAGTGATGCTGAAGCATTCGATCCAATGCATCACATCTTTTACACTGATTCCAAACTTTTGCCAAAGTACTCCTGAAGTCTGCAGGACATCCTACAGGCCCATCCATTGTATTAAATCAAACCACTATTACACCATCATATTAGTAAAATGCTGTGGAGAATTCATTTTTCCAATTGCCATTGCTTGATAACAGTTATGTTATCAATTTTAATAAATGAACAGGGCTTGAAGTTTTCATTCTTTGCGTGAGGATGCATTTCAATGTCTCTTGTTTCAATGTAAGTATTTATCAACAACAAAACACGCACCTATGCCTAGCCACCATTTCAAAGTTTATTTACGTATGTTTTAGTTTTGTCAAAGCAACAAATTTTTAACTTGTAGACGCATGTCCAGTAGAGACAGGTTTGCCATTGACAGGTGAATAAAATTAACATATTTTTTACCATACAGAAAAGAAAACAGTATTTACTATTTTTCCAATACTTCGCAAACTTCTAAAAAAGATACACGACCAATTGATACAAGGATTTCTGACAATTGCTTTCAAATGGTTCACAACTTATAAATGCAAAAATAGCATTGATTACTCCATGAAGATATGAAATAATCATACATTAAATTATAAGAAATAAGTGGGAAGGAATGACGAAACTTAATTTAAT

Protein: RF -2: -2712 -> -286 (808 aa)

Comparison with *Halyomorpha halys,* PREDICTED: exonuclease mut-7 homolog - Sequence ID: [XP_024216394.1](https://www.ncbi.nlm.nih.gov/protein/XP_024216394.1?report=genbank&log$=protalign&blast_rank=1&RID=N8U0362D015)

E= 0.0; bits= 1402

Query 1 MDGPVGCPADFRSTLAKVWNQCKRCDALDRMLQHHFISSENPYESALILIRQSPDYSLSR 60

MDG GC ADF STLAKVWNQCKRCDALDRMLQHHF S++NPYES L+LIRQSPDYSLSR

Sbjct 1 MDGIAGCSADFSSTLAKVWNQCKRCDALDRMLQHHFDSNKNPYESVLVLIRQSPDYSLSR 60

Query 61 HNGLSFTIIEQFANWISIRRDNYKHCLTEEVQREAFQVASRQRHRIISKNITEVYQLKEN 120

NGLSFTII+QFA+WIS RR NYKHCLTEEV+REAFQVASRQR++ ISKNI +VY LK+

Sbjct 61 PNGLSFTIIKQFASWISYRRANYKHCLTEEVKREAFQVASRQRNKTISKNIIDVYLLKDY 120

Query 121 KHLFVKSLNDNLQLHMYKEVCEWSMMLDLESEFGIFDFIVPLIFQDKLTVVDDFLKKNRS 180

K LFVKSLNDNLQLHMYKEVCEWSMMLDLESEFGI DFIVPLIFQDKLTVVD+FLKKNR+

Sbjct 121 KQLFVKSLNDNLQLHMYKEVCEWSMMLDLESEFGISDFIVPLIFQDKLTVVDEFLKKNRT 180

Query 181 HHLELIKFLDNILSQRSIMNFIDQLIIDLKIENVKHASLPKLSQTKPLCKLLKRFASIHN 240

HHLELIKFLDNILSQRSI NFID LIIDLKIEN+KHASLPKLSQTKPLCKLLKRFASIHN

Sbjct 181 HHLELIKFLDNILSQRSISNFIDHLIIDLKIENIKHASLPKLSQTKPLCKLLKRFASIHN 240

Query 241 IPPDTTPNLTLRNGMGTVNFLIRKRYYDRSLSKEAFRELVKDACGSRREVMIHLVNRLDE 300

IPPD+ PNLTLRNG+GTVNFLIRKRYYDRSLSKEAFRELVKDACGSRREVMIHLVNRLDE

Sbjct 241 IPPDSMPNLTLRNGIGTVNFLIRKRYYDRSLSKEAFRELVKDACGSRREVMIHLVNRLDE 300

Query 301 EEDPTEALICARLFSVPRSDMPPSVRSLDENGWREDDLPLSNQQVEDEQDQNDQYHKFPL 360

E DPTEALICARLFSVP SDMPPSVR+LDENGWRED+L +S++ VE+ D +D+YHKFPL

Sbjct 301 EGDPTEALICARLFSVPSSDMPPSVRALDENGWREDNLAISDELVEERLDHSDEYHKFPL 360

Query 361 QLSSIFIIDTEAAFSSLIDSYIDGATTIGIDLEWKPTMVAPSGELALLQLAKEDKVFLID 420

QLSSIFIIDTEAAFSSLIDS IDGATTIGIDLEWKPTMVAP+GELALLQLAKEDKVFLID

Sbjct 361 QLSSIFIIDTEAAFSSLIDSSIDGATTIGIDLEWKPTMVAPTGELALLQLAKEDKVFLID 420

Query 421 VLALANSHQLWGKFAHVFLNNHDILKIGFAMGADSSMLAQCFPIDFAFRMSGLGFLDLSS 480

VL+LANSH LWG+FA+ FLNNHDILKIGFAMGADS+MLAQCFPIDFAFRMSGLGFLDLSS

Sbjct 421 VLSLANSHHLWGQFANAFLNNHDILKIGFAMGADSTMLAQCFPIDFAFRMSGLGFLDLSS 480

Query 481 LWNRLVKDYNFEFPYEANESCSSCSLSQLVALCIGKPLNKREQFSNWETRPLRPTQKVYA 540

LWN+LVKDYNFEFPYEANE+CSSCSLSQLVALCIGKPLNKREQFSNWETRPLR TQ+VYA

Sbjct 481 LWNKLVKDYNFEFPYEANETCSSCSLSQLVALCIGKPLNKREQFSNWETRPLRLTQRVYA 540

Query 541 ALDAYCLIEVYDVIKRLCSERKIPIDDIVQDMMTKTEQGKAKKSKSNKKNKEATRYRTTA 600

ALDAYCLIEVY VIKRLC+ER IP+DDI+QDMMT+TEQGKAKKSK +KK+KEA ++R+

Sbjct 541 ALDAYCLIEVYQVIKRLCAERNIPLDDIIQDMMTRTEQGKAKKSKKDKKSKEAMKHRSVP 600

Query 601 TEAKMMKLVCEPSLAMLACVLRRHGVDTVVAELHDIAEIAELENRIVLSSSWPFKTISEQ 660

TEAK MKLVCEPSLAMLA +LRRHGVDT++AELHDIA+IA ENRI LSSSWPFKTISEQ

Sbjct 601 TEAKKMKLVCEPSLAMLATILRRHGVDTILAELHDIADIAVSENRIALSSSWPFKTISEQ 660

Query 661 LSKDKCYLVRSCASPDQYREVIRQLYIIVKPEDIFTRCQECNSDHVVMIDKNTVEDWFAK 720

L KDKCYLVRSCAS DQYREV+RQLYIIVKPEDI +RCQ+CNSDHV MI+K TVEDWF +

Sbjct 661 LPKDKCYLVRSCASSDQYREVLRQLYIIVKPEDILSRCQDCNSDHVTMINKKTVEDWFLQ 720

Query 721 KDNTKTTNCQPAGYSDNELTDSDEEDDYYRSDMIAGT-GPVENGMNSHPTTTTGVPIRLD 779

K +K NC PAG+SDNELTDS EEDDY+ D T +ENG +PTT TGVPIRLD

Sbjct 721 KGKSKAENCVPAGFSDNELTDS-EEDDYFTLDTSDCTPNAIENGSKFYPTTMTGVPIRLD 779

Query 780 LVIEEQIKLADLICMCEDCGRVFWQTESK 808

LV EEQIKLA LI +CE CGRVFWQ E+K

Sbjct 780 LVAEEQIKLASLISLCEKCGRVFWQAETK 808

Graphical representation


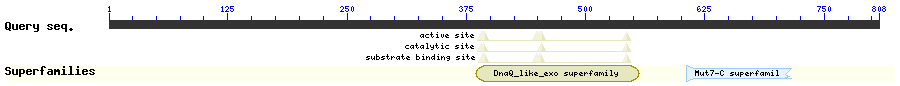


**Data S5:** Sequences of *E. heros* Antiviral.

**Ars2**

>TRINITY_DN25188_c0_g1_i1 length= 2434 nt

ATACGTTTCAAAACTGCATACTCTGCTTTAGGAGGCTGTGAACGAGCCACAGTTTACAGTCAACTATTCTTCGGAGGTGGATTGTTTGTAGTATTAGGACTTTTATTTTACTGATTGTTAATAGAAAGTTGGTTTCAATATGGGAGATAGTGAAGATGAGTATGATAAAAAGAGACGAGATAAATTTCAAGGTGAAAGGAGTGATTCATATAGGACTGATAAACGCAAAGACGATGACTGGGGAAGACCTAGGATGAGAAATGATTATCGAGACAGGTACCAGTACCAAGGAGATTTGCCACCTACTAAACGGATGAGATATGACTCGGATGACATTAGGAGAATGAGGTATAATGATCCTGGGTATGGCCCATACAACAGTTGGGGTCCTGAACCACCATATCCTGGAAATTCAAGGCTAGGTGAAATGGATACCCAACCTCCAATCATGACTTTCAAAGCTTTTCTTCAATCTCAAGATGACAACATAACTGATGAAGAAGCTATAGCTAAATATGCTGAGTATAAACTTGAATTTAGGCGCCAACAGCTGAATGAATTTTTCGTCGCTCATAAAGAAGAAGAATGGTTCAAACTAAAATACCATCCTGAAGAATCTCTTAAAAGAAAAGAAGAACTTAATAGTGCACTAAAGAGAAGGTGTGATGTTTTTCTTGAAATGTTGGAATCAAAACGCATGGATGATATAAGAGTTGATACCGAACAAGGAGATGAAGTTGTCAAACTTTTAGATTCTGTTGTAATTCGTCTAGAAGGAGGAACTGACCTTGATTTAACAATTCTCGACCAACAGGAAGGGCAGAAAGATGAGCTAAAAGAAAAGGAGAAGAGTGATGAGAAACCTAAAGACAACAATAAGTCAGATGAAGAAGAAGTTAAAAAAAAAGATAATGAAGAGAATAAAGATGAAATTGTAATTGATGAAGATGATGACCGTAAAGAGGAAGAAGAAAAGAATGAAGTTCATGAATTATCTGAAGATGAAAGACCACCAGGAGTTGATCCTGAAAGTGATATTGAAAAAGAAGATGTGCCTGCAGAGAAAGAATCGCCATCTGAAAAAGAGATGCAACATGAAGAGAAAGAGTCATCACCAGAAAAAGAAAAAAAGGAAGAAGTAAAGAAACCAAAAGATGACCAAGATTCTGACAAGGATCACGATGAAGTTGTTCCATTAACACCTAGAGCACTGCACAAAACTTCATCTATTTTTCTTCGAACCATTTCTCCCAGAGTTACCAAGGCTGAAATTGAATCGATTTGTAAGAAGTATCCAGGATTTCTTAGAGTATCCCTTTCTGAACCTCAAGCAGAACAGCATTGGTATAGAAGGGGATGGGTGACTTTTAGGCGTGATGTTAATATTAAAGAAATCTGTTGGAATTTAAATAATATAAGGGTGAAAGAGTGTGAAATGGGGGCTATAGTAAATCGGGATTTATCACGCAGAGTAAGATCAGTGAGTGGACTGTCAGGACATAAACAGGTTGTCTTGGCTGACTTGAAGTTAGCTGCAAAAATCATACAGGAATTGGATTCAAGGGCTGGTTTCTGCAATCCTGATCACAGCGAAACTTTTGGGTTGGAATCAAGAAATGCTGTCTTTAAAGGTATTACAGAGTATTTGGTTGAAGAAGCCCCAGCTGAGGAAGAAGAACTTTTGGGTCAAGGTATCGTTGCTGACGAATCCAAGTCTCGCATAGCAGGTGCTATAAAGATTCTGGATAAACTGCTTCTTTATTTGAGGATTGTCCATTCTGTGGATTACTATAACACTTCACAATATACATCAGAAGACGAAATGCCCAACAGATGTGGCATAATGCATTTAAGAGGATTACCTTCAAGTACTGAGGTTTCTCCACAAGAAATACAAGAATATATCGACGGTTATAAAACAAAACTTGAGCCCTTATATACTCCAGTACCTACTGTTTCTGAACAAGAACTAACATCATTAGGTGCTAAGGATAGAGACACTGAAGTAGAGAAATTTATACAAGCTAATACTCAGGAATTAGCAAAAGACAAGTGGTTATGTCCTTTATCAGGAAAAAAATTCAAAGGACCAGAGTTTGTGAGGAAACATATTTATAACAAATTTGCTCAAGAACTTGAAGAAGTAAAAAAGGAGGTTGATTATTTTAATAATTACCTTCGTGATCCAAAACGGCCACAGTTAGCAGAACATCCTGGAAATAGGGGTGGGAAAAAGGAACCTGAATCTCCTTACCATTACCAGTATGGAGGTGGTTTCAAACGTGGATTTGGCCATTTTGGTGGACATGGTGGTTTCAACCGTGGACGAGGAGGTTTTGGAAGGGGAAGGGGTATGGACTACAGGCCTATTATTACTTATAGGGACTTGGATGCCCCATGTGAGCCAGATGAAATAATATAAATGCAGTCTTAT

Protein: RF -2: -140 -> -2422 (760 aa)

Comparison with *Halyomorpha halys,* PREDICTED: serrate RNA effector molecule homolog isoform X1 - Sequence ID: XP_014277995.1

E: 0.0; bits= 1523

Query 1 MGDSEDEYDKKRRDKFQGERSDSYRTDKRKDDDWGRPRMRNDYRDRYQYQGDLPPTKRMR 60

MGDSEDEYDKKRRDKFQGERSDSYRTDKRKDDDWGRPRMRNDYRDRYQYQGDLPPTKRMR

Sbjct 1 MGDSEDEYDKKRRDKFQGERSDSYRTDKRKDDDWGRPRMRNDYRDRYQYQGDLPPTKRMR 60

Query 61 YDSDDIRRMRYNDPGYGPYNSWGPEPPYPGNSRLGEMDTQPPIMTFKAFLQSQDDNITDE 120

YDSDDIRRMRYNDPGYGPYNSWGPEPPYPGNSRLGEMDTQPPIMTFKAFLQSQDDNITDE

Sbjct 61 YDSDDIRRMRYNDPGYGPYNSWGPEPPYPGNSRLGEMDTQPPIMTFKAFLQSQDDNITDE 120

Query 121 EAIAKYAEYKLEFRRQQLNEFFVAHKEEEWFKLKYHPEESLKRKEELNSALKRRCDVFLE 180

EAIAKYAEYKLEFRRQQLNEFFVAHKEEEWFKLKYHPEESLKRKEELNSALK+RCDVFLE

Sbjct 121 EAIAKYAEYKLEFRRQQLNEFFVAHKEEEWFKLKYHPEESLKRKEELNSALKKRCDVFLE 180

Query 181 MLESKRMDDIRVDTEQGDEVVKLLDSVVIRLEGGTDLDLTILDQQEGQKDELKEKEKSDE 240

MLES RMDDIRVDTEQGDEVVKLLDSVVIRLEGGTDLDLTILDQQEGQKDE KEK+KSD+

Sbjct 181 MLESHRMDDIRVDTEQGDEVVKLLDSVVIRLEGGTDLDLTILDQQEGQKDEQKEKDKSDD 240

Query 241 KPKDNNKSDEEEVKKKDNEENKDEIVIDEDDDRKEEEEKNEVHELSEDERPPGVDPESDI 300

KPK+NNKSDEEEVK+K+NEENKDEIVIDEDD+RKEEEEKNEVHELSEDERPPGVDPESDI

Sbjct 241 KPKENNKSDEEEVKQKENEENKDEIVIDEDDERKEEEEKNEVHELSEDERPPGVDPESDI 300

Query 301 EKEDVPAEKESPSEKEMQHEEKESSPEKEKKEEVKKPKDDQDSDKDHDEVVPLTPRALHK 360

EKEDVPAEKESPSEKE Q EEKESSPEKEKKEEVKK KDDQDSDKDHDEVVPLTPRALHK

Sbjct 301 EKEDVPAEKESPSEKETQPEEKESSPEKEKKEEVKKSKDDQDSDKDHDEVVPLTPRALHK 360

Query 361 TSSIFLRTISPRVTKAEIESICKKYPGFLRVSLSEPQAEQHWYRRGWVTFRRDVNIKEIC 420

TSSIFLRTISPRVTKAEIESICKKYPGFLRVSLSEPQAEQHWYRRGWVTFRRDVNIKEIC

Sbjct 361 TSSIFLRTISPRVTKAEIESICKKYPGFLRVSLSEPQAEQHWYRRGWVTFRRDVNIKEIC 420

Query 421 WNLNNIRVKECEMGAIVNRDLSRRVRSVSGLSGHKQVVLADLKLAAKIIQELDSRAGFCN 480

WNLNNIRVKECEMGAIVNRDLSRRVRSVSGLSGHKQVVL DLKLAAKIIQELDSRAGFCN

Sbjct 421 WNLNNIRVKECEMGAIVNRDLSRRVRSVSGLSGHKQVVLGDLKLAAKIIQELDSRAGFCN 480

Query 481 PDHSETFGLESRNAVFKGITEYLVEEAPAEEEELLGQGIVADESKSRIAGAIKILDKLLL 540

PD SETFGLE+RNAVFKGITEYLVEEAPAEEEELLGQGIVADESKSRIAGAIKILDKLLL

Sbjct 481 PDQSETFGLETRNAVFKGITEYLVEEAPAEEEELLGQGIVADESKSRIAGAIKILDKLLL 540

Query 541 YLRIVHSVDYYNTSQYTSEDEMPNRCGIMHLRGLPSSTEVSPQEIQEYIDGYKTKLEPLY 600

YLRIVHSVDYYNTSQYTSEDEMPNRCGIMHLRGLPSSTEVSPQEIQEYIDGYKTKLEPLY

Sbjct 541 YLRIVHSVDYYNTSQYTSEDEMPNRCGIMHLRGLPSSTEVSPQEIQEYIDGYKTKLEPLY 600

Query 601 TPVPTVSEQELTSLGAKDRDTEVEKFIQANTQELAKDKWLCPLSGKKFKGPEFVRKHIYN 660

TPV TV+EQELTSLGAKDRDTEVEKFIQANTQELAKDKWLCPLSGKKFKGPEFVRKHIYN

Sbjct 601 TPVQTVTEQELTSLGAKDRDTEVEKFIQANTQELAKDKWLCPLSGKKFKGPEFVRKHIYN 660

Query 661 KFAQELEEVKKEVDYFNNYLRDPKRPQLAEHPGNRGGKKEPESPYHYQYGGGFKRGFGHF 720

KF+QELEEVKKEVDYFNNYLRDPKRPQLAEHPGNRGGKKEPESPYHYQYGGGFKRGFGHF

Sbjct 661 KFSQELEEVKKEVDYFNNYLRDPKRPQLAEHPGNRGGKKEPESPYHYQYGGGFKRGFGHF 720

Query 721 GGHGGFNRGRGGFGRGRGMDYRPIITYRDLDAPCEPDEII 760

GGHGGFNRGRGGFGRGRGMDYRPIITYRDLDAPCEPDEII

Sbjct 721 GGHGGFNRGRGGFGRGRGMDYRPIITYRDLDAPCEPDEII 760

Graphical representation


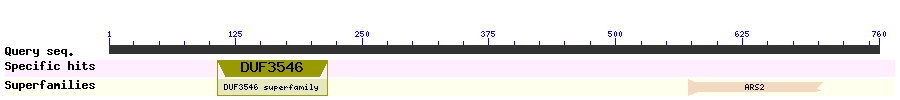


**NinaC**

>TRINITY_DN16459_c0_g1_i1 length= 939 nt

TTCCCCAAATACCATCCTTCAAGTTTCAAGCGAATCATTAACAGGCGGCAGTTATCTCTTGTTTGTTCAACTTCCTCATCAAAGTCAAAAGCCAAAAATTGATATCGGTCCAAACATTGTGCGAAGGTAATCCTATGAGAATAGCCCTGCTGTCTAGCTTTTGCTGTATCGATTACTGCTAAAGCCCTTAGCTGTTGTCTGATGATACCTTGTTGGAAACCTCCTGGAGTACCCTGAAGATCTGTCCTGATACATCGAACAAAATGGGAACTACCTGATGCTAATGATTTCAAAATCTCAAGAGATGTTGATTTAAATATGGCTGCAGCAGTTCTTATACCCCTTGTTTGAGAAAACTCCCCTTTTGAAGCTGTATTGTAAGGCCTTGCAATTCTGTTTTGATCAGCTAGTAATGCTGATCCCCAACTTTTCTTCTTAAGAACACCAGTACTGGTTATAATATTATCTGTGTTAATTGTGAGATTTCCAGTCCGGTTCAATTTGTTCGTAAAGAGCTGTTTAATATTATTGTTTACTGAACATCTTAGAATTTCAGTTAATTCTGGTGGCAGGAAATCTCGATTTTTTGTACACATGGTTGATGTTTGATATCGTACTTTTCCTGTATAATGAGCTACGAAAAATTCTTCTTCTCCACATGAGAGTATACGGGACCCTTCACTTTTTTTGTCTAGAGCATCTATTATGTATTCTGAATCCATATTGAGTTTGTTTGCTTCATCTAAAATATGCATAAGGCCATTTGGTTTATTAAATAATTCATCCATTGTCTCCCTGTTATTGTAAAATTGAAACTTTTTTACTTGAATTTCTTCCTCCTCTTCTTCTTCAATTTCAGATATAAATATTTTTTGGTTGTAATAATACTGAAGTTGTTCATTCAATGAGTTTACAAATAGTTGTTCTAGCCAGATCGGA

Protein: RF -2: -788 -> >3 (261 aa)

Comparison with *Halyomorpha halys*, PREDICTED: neither inactivation nor afterpotential protein C - Sequence ID: XP_014281724.1

E= 0.0; bits= 525

Query 1 MDELFNKPNGLMHILDEANKLNMDSEYIIDALDKKSEGSRILSCGEEEFFVAHYTGKVRY 60

MDELFNKPNGLMHILDEANKLN+DSE+IIDALDKKSEGSRILSCGEEEFFVAHYTGKVRY

Sbjct 776 MDELFNKPNGLMHILDEANKLNVDSEHIIDALDKKSEGSRILSCGEEEFFVAHYTGKVRY 835

Query 61 QTSTMCTKNRDFLPPELTEILRCSVNNNIKQLFTNKLNRTGNLTINTDNIITSTGVLKKK 120

QT+ MCTKNRDFLPPELTEILRCS NNNIKQLFTNKLNRTGNLTINTD +ITSTGV+KKK

Sbjct 836 QTTAMCTKNRDFLPPELTEILRCSANNNIKQLFTNKLNRTGNLTINTDTVITSTGVIKKK 895

Query 121 SWGSALLADQNRIARPYNTASKGEFSQTRGIRTAAAIFKSTSLEILKSLASGSSHFVRCI 180

SWGSALLADQNR ARPYNTASKGEFSQTRGIRTAAAIFKSTSLEILKSLASGSS+FVRCI

Sbjct 896 SWGSALLADQNRTARPYNTASKGEFSQTRGIRTAAAIFKSTSLEILKSLASGSSYFVRCI 955

Query 181 RTDLQGTPGGFQQGIIRQQLRALAVIDTAKARQQGYSHRITFAQCLDRYQFLAFDFDEEV 240

RTDLQ TPGGFQQGIIRQQLRAL+VIDTAKARQ GYSHRITFAQCLDRYQFLAFDFDEEV

Sbjct 956 RTDLQSTPGGFQQGIIRQQLRALSVIDTAKARQLGYSHRITFAQCLDRYQFLAFDFDEEV 1015

Query 241 EQTRDNCRLLMIRLKLEGWYLG 262

E+TRDNCRLLMIRLKLEGWYLG

Sbjct 1016 EKTRDNCRLLMIRLKLEGWYLG 1037

Graphical representation


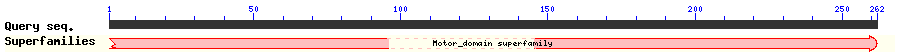


**Beta 1,4-mannosyltranferase (Egghead)**

>TRINITY_DN19795_c0_g1_i1 length= 2462 nt

GGAGATTTTTTCAAGGAAATAATGAATAATTCACAATAAAAATAAATAAATAAAGCTTATTTTAACTAAGAATCCTACATGATTACTATACGTAACACAAATATAACAGTATAATGAAATATAATACTATACTGTTCAGTCTTCCTATATCATTTACACTTGTTGTGCAATAACAACTAATTAAGTAGAGGAGTACATTTTTAATGAGTTAAGAATTATAAATACAAATTAAAAAAAAATAGCAACTTAATGAGGTTTATTAAGAAAACAATACAATAAATGGACATTACACAGCCAACTTTATCCTTTCTGTTCTTCCTTATAATACGGACAAGTAAATTTCATAATCACTGACTAAGGAACAGCTAACACAGGTCTATACACACATGCACTCATACATTAATGAACAAGCAAGTAAACTGAAATTATGACAATATTACAATAAAATTAATTAATCACTATTACAGAATTAGATAAGAAAATAAAAAATTATGCTCATGATGTTCTTATATTGCAGTGTAATTATAAATCACAATGTTTATATATAAAGAATGTTCAGATACCCTTCATCAAATATACCCAATATTATAACACCAGGTTTAATTCATATTAGAAAAAATAAAACACTATGACCACATTTATTTAATATTGACATCACAAAGTTTACTTTAGACTTTGTACTTAGATAAATGTAAACATTTATTATCATAGTATGAAATGAATGATGACTATCATTAATGTGAGGGGTAAAACAGACCTTTTTAAATAATAAGAACAAGGACTTTACGGAATACATGACTCTATTTAAATAAATATCAAAATTACTCTACCTAAGCATTGATAACTTCTGAATCTAACAACTAAACAGTCAATGGCGGTCGATTATCTTTATTTACAACATAAAATTTATGCTTTTTTCCTAATAAACCCCAAATCACTGCAACATTTTCAATAACAACATTGACTGGAATCGTAGCTAATGCACCAAAAATACAAAGACTAAACTTGATGAAACCAAAACGATATAATGAAAATGATTTAATAACACCAAAAATATACATATAGATGCTTACAGCACCAATGAAAGCACATAAGAAATCTATAAAAGGAGGACAAGGAATTGGACACAGTGAAGCTAATAATATATTTGATGTTGAAAGTGGCATTGTAACCCATGAATAACATGAAATACTGAGAAATAATTTATTTTTCAAAGGAATTTTTTTCGAACGTACAACTAGAAGGATTCCTTGAAGCCAACGTTTTCTTTGTTGTAAGAAGTCACCAAGAGTAAAAGGCGACTTTTCCCACATTTCTCCTTCAATGAAATTGAATGTATATCCCATTTTATATGCTTTCATAGCAAAAAAACAGTCTTCAGCAACAGAACCATCCAGGCCATTATCAAATGTCACATCACGTTCTGCTTTCACCTGAGTGACGACATATGATCCTTTCCAGCTAAGAAGGGGTTTGTGAAACATTAGAAATTGAAAGCGAAGCTTTCCCATATCATCTGCAACCCTAAAAGTGTCAGCAAGAGTAGTGATCCAGTTGACAACTTCTTCATTTGCATATGTTATCAGGCCTTGCCCAAATGAATGTTTTCCATCGATGACAAAATTCAAAATACCTCTAATAGAATTTTCTGTCAAGAGAGTTTCTTCATCAAGATGTACAATCCAATCAGAATCAGATAAAGTATTAACACCTTCTTCTAAACAATACTGAAGGGCACGAGCTTTAAATAGTGCACCAGATTTAGGTTTATAATTTCCTGGCACAACAGTTTCTATAACCCGCCTATCTTTCATTAGATTTAAAGGTTTATCAGTCACTACTTCAATTACAAAGTGTTCTAAACCGGCACTCAAACACTTGGACAAATTACGGCTAACATTGTTCTTCACTAACTGTGGATAATCACCACGAGTAACTATCCTTACACAGATAAATGGGGCTAAAAGTGGACTACCTTTCAAAATAACATTGTCAGGGAAAGCATTATAAAATGTAAGCCCAGCGAAATTCAGCAGGATCTGCGGCAAAGGTAAAAATGCCAAAAATCTAAGAAAATATAAAGTGATCGTTATCAAAAATCCATATTTCGCCCATGGATCTATAACTGTTTCATTACTTGATAACTTAAGTCCTCCAGTTAATAGTTCAAAAATAATGATAACTGAAAACAATAATGCACAGTGAAGAGCATGCTTAGTTTTGCTGTTTAACATATCTGACACTTTTGTTATCTCCCAACGATCAAATGCATTACTATTCCTAACAATTTGATTGGTAAAAATGTGTAATAAAATCACTAAAAATGATAATAAACTTAGTGAATAACATTTATACAATCATTTTAAGTAGTCCGTTATTTTTGAACACCAAAGAAAATTCACGAACAACTTCAAAATGGCAACACCCAAATATGACTTAGGAGCATGACTGCTTAATTAACT

Protein: RF -1: -2234 -> -861 (457 aa)

Comparison with *Halyomorpha halys*, PREDICTED: beta-1,4-mannosyltransferase egh - Sequence ID: XP_014283435.1

E: 0.0; bits= 914

Query 1 MLNSKTKHALHCALLFSVIIIFELLTGGLKLSSNETVIDPWAKYGFLITITLYFLRFLAF 60

MLNSKTKHALHCALLFSVIIIFELLTGGLKLSS+ETVIDPW KYGF+ITI LY LRFLAF

Sbjct 1 MLNSKTKHALHCALLFSVIIIFELLTGGLKLSSDETVIDPWVKYGFVITIVLYALRFLAF 60

Query 61 LPLPQILLNFAGLTFYNAFPDNVILKGSPLLAPFICVRIVTRGDYPQLVKNNVSRNLSKC 120

LPLPQILLNFAGLTFYNAFPDNVILKGSPLLAPFICVRIVTRGDYPQLVKNNVSRNLSKC

Sbjct 61 LPLPQILLNFAGLTFYNAFPDNVILKGSPLLAPFICVRIVTRGDYPQLVKNNVSRNLSKC 120

Query 121 LSAGLEHFVIEVVTDKPLNLMKDRRVIETVVPGNYKPKSGALFKARALQYCLEEGVNTLS 180

LSAGLEHFVIEVVTDKPL+LMKDRRVIETVVPGNYK KSGALFKARALQYCLEEGVNTLS

Sbjct 121 LSAGLEHFVIEVVTDKPLHLMKDRRVIETVVPGNYKAKSGALFKARALQYCLEEGVNTLS 180

Query 181 DSDWIVHLDEETLLTENSIRGILNFVIDGKHSFGQGLITYANEEVVNWITTLADTFRVAD 240

DSDWIVHLDEETLLTENSIRGILNFVIDGKHSFGQGLITYANEEVVNWITTLADTFRVAD

Sbjct 181 DSDWIVHLDEETLLTENSIRGILNFVIDGKHSFGQGLITYANEEVVNWITTLADTFRVAD 240

Query 241 DMGKLRFQFLMFHKPLLSWKGSYVVTQVKAERDVTFDNGLDGSVAEDCFFAMKAYKMGYT 300

DMGKLRFQFLMFHKPLLSWKGSYVVTQVKAERDVTFDNGLDGSVAEDCFFAMKAYKMGYT

Sbjct 241 DMGKLRFQFLMFHKPLLSWKGSYVVTQVKAERDVTFDNGLDGSVAEDCFFAMKAYKMGYT 300

Query 301 FNFIEGEMWEKSPFTLGDFLQQRKRWLQGILLVVRSKKIPLKNKLFLSISCYSWVTMPLS 360

FNFIEGEMWEKSPFTLGDFLQQRKRWLQGILLVVRSKKIPLKNK+FLSISCYSWVTMPLS

Sbjct 301 FNFIEGEMWEKSPFTLGDFLQQRKRWLQGILLVVRSKKIPLKNKIFLSISCYSWVTMPLS 360

Query 361 TSNILLASLCPIPCPPFIDFLCAFIGAVSIYMYIFGVIKSFSLYRFGFIKFSLCIFGALA 420

TSNILLASLCPIPCPP IDFLCAFIGAVSIYMYIFGVIKSFSLYRFG IKFSLCIFGALA

Sbjct 361 TSNILLASLCPIPCPPLIDFLCAFIGAVSIYMYIFGVIKSFSLYRFGVIKFSLCIFGALA 420

Query 421 TIPVNVVIENVAVIWGLLGKKHKFYVVNKDNRPPLTV 457

TIP+NV+IEN AVIWGLLGKKHKFYVVNKDNRPPLTV

Sbjct 421 TIPINVIIENAAVIWGLLGKKHKFYVVNKDNRPPLTV 457

Graphical representation


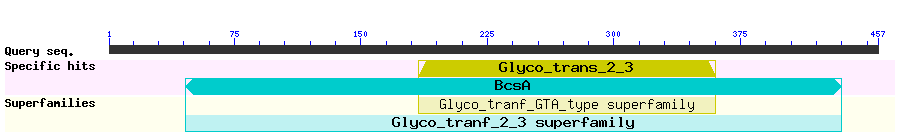


**CG4572**

>TRINITY_DN16339_c0_g1_i1 length= 1543nt

TTTTTACTCAATGATAAAACATATTTATTTCTTTAAACATGTTATTAACCAAAGTAACATGTAACAATATGAGTCAAGAAGAAATTCAGAATGGTTTATTAAACACAAAACGTGTAATTAAATCAAGGGCCCATTTCGGTTGGTCTCCAGGCACCATATGACCAGCATTTCTGACCAGCACTTCGGTAAAACCTTTTACAGATTTTGAATATCCAGCAAGTTCATTTCCCACCATCCATTTTTTTCTTGGAACTGTTTTATATATATTGGCTCCACTCCACTTTAATCGTTGCAAAAAATTTATTGTAAGTGGATAGGGAACAATTATATCCAACTGGCCATTGTAAAACAATACTTTATATTTCTCTACTAATTTTTCTACCAAAGGCTTAACTGATTGCATTATATCTTGTTTTAAATACTGTTCTACTTTTGTGTCAGTGTGAAAAGTCAGATTTCCTACATGTATGCTTCTTCTCATTATATCTTTTTGAACATATACTGCCATGTCTCCATATGGACTGTCATCTTGATTATGCAAATAGTTAAAATAAAACTCAAATCCTGTCTGATTGTAAAAAAAACTTTTATAAGGAGTTAAATCACCGTTTAGTAAAGCATCAAAACCTTCAAAGGCTTTTAAATAATTCTTTGCTTGAATATTTTTAATGATCTCATCTTGCTTCCTTTGAAACGCCATCTTCGTGTTACCGTCTATTAAACCAATCTGGTATAGGTAATCTCCATAGTTCATCATATTTTCTGGGTCTGATAGACCGTTGCCAATAGAAATTCCCTTCAAATTTATTTTTTGAATATTTTTTTCATTGCTAGTGTGAATTTTATAAGCAACTGCAGGGACATATTTTCCGGCATATGATTCACCAGCTACAAAGAAATCATTTTTTTGAAGTTCTGGAAATAATTTAAAAAATTGTATAAGAGCTGAGTATAGATCATTTCCAACACCATCTTCATTTTTTACATATCCATTATCATTTTCCGTAAAACTAAATCCTGTGCCAACAGGGTTATCAATGTAAAGGACATTAAGTATCTGTGACCAATAATATTTTCTAGATTTTAGACCTCTTTCTTTCTTAACGTAGAAGGGACCATTTTCATTAAATAATCCATATAATGACGAAGCACCAGGCCCTCCTTGTAGCCAAAGCACAACTGGGGCAGAAGTTGAATTATTTTCTGCAGGAAAATACCAAAAAAACATGTTGGAATTGTACTGCTTATTAACTGTTAAAAAACCAGCATAGCTTTTAATGTTAGATTTTACTGGTTTGACAGATGCCTGCAATCGCCCTTCCGCGATGGACCCATTTTCAATATACGGAGTTAAAAACAAAGGGTCTCCAACATTATCACCAGCTATATGAGGAAAATTTCTATAACTATAAAGTCGAAAACCACTGATCAAACTAAAAAGTATGTACATACACAATAAGGAAAAATAAGAATTCATCATGACACTGTTTTCACTTCAAACGAAATTTAAACTGAAAATCAGAAGCCAAAAGTATTACGCAAG

Protein: RF -1: -1480 -> -86 (464 aa)

Comparison with *Halyomorpha halys*, PREDICTED: venom serine carboxypeptidase-like - Sequence ID: XP_014280828.1

E: 0.0; bits= 857

Query 5 YFSLLCMYILFSLISGFRLYSYRNFPHIAGDNVGDPLFLTPYIENGSIAEGRLQASVKPV 64

Y L+C+Y + SGF + YRNFP+I GDNVG+PLFLTP+IENGSIAEG+ ASVKPV

Sbjct 14 YLVLICVYYCVNFTSGFGM-KYRNFPYITGDNVGEPLFLTPFIENGSIAEGQSAASVKPV 72

Query 65 KSNIKSYAGFLTVNKQYNSNMFFWYFPAENNSTSAPVVLWLQGGPGASSLYGLFNENGPF 124

K+N+KSYAGF TVNKQYNSNMFFWYFPAENNST+APVVLWLQGGPGASSLYGLFNENGPF

Sbjct 73 KANVKSYAGFFTVNKQYNSNMFFWYFPAENNSTTAPVVLWLQGGPGASSLYGLFNENGPF 132

Query 125 YVKKERGLKSRKYYWSQILNVLYIDNPVGTGFSFTENDNGYVKNEDGVGNDLYSALIQFF 184

YVKKERGLKSRKYYWSQILNV+YIDNPVGTGFSFT+NDNGYVKNEDGVGNDLYSAL QFF

Sbjct 133 YVKKERGLKSRKYYWSQILNVIYIDNPVGTGFSFTDNDNGYVKNEDGVGNDLYSALTQFF 192

Query 185 KLFPELQKNDFFVAGESYAGKYVPAVAYKIHTSNEKNIQKINLKGISIGNGLSDPENMMN 244

KLFPEL+KNDFFVAGESYAGKYVPA+AYKIHTSNE+N+ KINLKGISIGNGLSDPENM+N

Sbjct 193 KLFPELRKNDFFVAGESYAGKYVPAIAYKIHTSNEQNLPKINLKGISIGNGLSDPENMLN 252

Query 245 YGDYLYQIGLIDGNTKMAFQRKQDEIIKNIQAKNYLKAFEGFDALLNGDLTPYKSFFYNQ 304

YGDYLYQIGLID +T+MAFQRKQD+I+KNIQAKNYLKAFEGFDALLNGDLTPYKSFFYNQ

Sbjct 253 YGDYLYQIGLIDSSTRMAFQRKQDDIVKNIQAKNYLKAFEGFDALLNGDLTPYKSFFYNQ 312

Query 305 TGFEFYFNYLHNQDDSPYGDMAVYVQKDIMRRSIHVGNLTFHTDTKVEQYLKQDIMQSVK 364

TGF FYFNYLHN+DDSPYGDM YVQKD+MRRSIHVGNLTFHTD+KVEQYLKQD+MQSVK

Sbjct 313 TGFSFYFNYLHNEDDSPYGDMGEYVQKDVMRRSIHVGNLTFHTDSKVEQYLKQDVMQSVK 372

Query 365 PLVEKLVEKYKVLFYNGQLDIIVPYPLTINFLQRLKWSGANIYKTVPRKKWMVGNELAGY 424

P +EKLVEKYKVLFYNGQLDIIVPYPLTINFLQRLKWSGANIYKTVPRKKWMVGNELAGY

Sbjct 373 PWIEKLVEKYKVLFYNGQLDIIVPYPLTINFLQRLKWSGANIYKTVPRKKWMVGNELAGY 432

Query 425 SKSVKGFTEVLVRNAGHMVPGDQPKWALDLITRFVFNKPF 464

SK+VKGFTEVLVRNAGHMVPGDQPKWALDLITRFV+NKPF

Sbjct 433 SKTVKGFTEVLVRNAGHMVPGDQPKWALDLITRFVYNKPF 472

Graphical representation


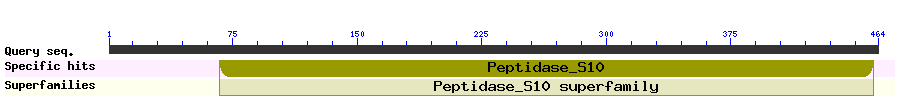


**Data S6:** Sequences of *E. heros* Intracellular transport.

**Vacuolar H+ ATPase subunit A (vha68)**

> TRINITY_DN27975_c3_g2_i3 length= 4431 nt

AAAAGAATGATTCTAGAAGGCAATGGATTAAGACAAATAAATAAAATACTATTAAAAAAACAGTCCTTTTTTAATTAATTAAAATTGCGATCATATCAATAGTTAATTCCAAAACTAACATAAGTAAAATGATTTAATGTGGAGAGAATATGGCACAACCTGTCTGCATGTGTTAATGACATCTCAGATGTATATGAAAAACATTATTTTTATTGTTACATCTCCTTACTTTGTACAGGAAATCATACCAAATTTAATAATTGAATAAAATTTTATTAGCAGAGGCATACACGCTCATATGCATACACGCTGTCAGGCACACTATACACACAAAAAAATATACATAAACATTTAATCTGTACAATTTCATTTATAAACATGATCATATATTGTCTTCATCAGGTTTCGACCAATGCCAATGTAAATTTTATCGCTCAAAGGATTTCCAGTCATTAACTAAGATTTAACAGGGTGAAAGAGTAAACAATCACTGTTTCGAAAAGTGTCGAGAGTATGTGGACACCAAGCAAACAAAAACAAATTATAAATTACAAGTTATAGAAACACAAAAGTCGAGATAACTTTATTAACGTTCACTATTCGGTAAATATTAATAACCATCATCGTGAAGATTTTTGATTGGTATAATATTAAATGCAACATCAACATCACTTATCAGAGTTCTTTTTATCATGATGGTGGTGGTGGTGTGATGAGTTCCTCTCGTTAAGATGTTTGTCATTGTGGTGATTATGGTCGTGCGATTTCTTATCATGGTGATGATTAAACTTTTCATTGTGATGGTGCCTTTCATTATGGGAGATTATTCCATTAGTGAGAGCTGGTGTTCTGAGGTTCTTCTGCAGGACATGAGCATCAGCCGGTTCAATTCTTTTGTAATAATGTTTCTTAGTTTCATGAATTTCAAAACCAAATTTTTTGTAAAAATCTATCGCACCAGTGTTATTGACTTGAACATGCCTGGTCATTAATAAACTATCAAGAGTATTGTTGAAAAAGCTGACAAGATACATTATCAAAAGCAGCACTGATACAGAAGTCTAAAATAAACATTATCATAATTTCCATCCTTCTCAACATGCCTCAAAACATGTTCAACCATAACAGTGCCAACCCCTAATCTGCGGTATGGTGAAAGACATCCAAGGGTCATAATATAAAGTCTACGTGAGTTTTCTGAAGTATCAACACGACAACAAACTGCTCCGACAACAATATCGTTGTAGTATGCTAACTTAGCAAGTTCTCCAACTTCCAATACATCTTTATAAAACTTTTCATTGTAAGAAACTGGGAAAACAACAGTGTTTAGTGTTTTCAACTGTTTCACATTGTGAGGGGTAACGTCTCCAAGTTCAATTTTCGCTCGTGGCGGTTTTTCTACATCACTTATATTTATATCACTGGACATGGTAAAATAACGCTATTCGTCAGCGTATGGTATGATAAGCCGAGCGTATAGACCCGAGGTAAAAAAAATCATTAATTCTAAAACAAGTCCATCAAGTGAAACACTTTCAAGCAGTTTTCTGAAAGGTTCTGTATGAGGACGGGAACACGGAGAGGGAGAACTTAACTATCAGCTATAAGATCACGTGCTCCAATTATAATTATTCAAATGATTTATAAAAAGTAGAATTTGTTAAATTACATAATTTTTTTTTGTCTCTCTTATAATGCAGCTAAATGTTTCTAAATAGACAATTCAGAGAATTGTATGTTTAATACTAGAGCCCAAGGTTTGGTGCTGATATAAGTTGCAGCTTAGATACAGCTGATAGTTCTTGTACTCAGCTGTAGGTGGGTGAGGTTGAAGTGATATACATTTACGTGACTAGGAGGAAAATTTTGCAAAATGGCCTTGCCCAGGATAAAAGATGAAGACCAGGAGTCGAAGTTTGGATATGTGTTTGGTGTATCTGGTCCTGTCGTCACAGCTGAAAAAATGTCAGGATCAGCTATGTACGAGTTAGTAAGAGTTGGTTATTTTGAACTGGTTGGTGAAATTATCCGTCTTGAAGGTGATATGGCAACCATTCAGGTATATGAAGAAACTTCTGGAGTTACAGTTGGTGACCCTGTCTTAAGAACAGGTAAACCTTTATCTGTCGAATTGGGTCCTGGTATTCTTGGAAGTATCTTTGATGGTATCCAGCGACCCTTGAAAGATATTAACGAAATCTCAAACAGTATTTACATCCCAAAGGGTGTTAACATCCCAGCATTGTCAAGAAGTGCAGCTTGGGAATTCACACCAACTAATATAAAGGTTGGAAGCCATATAACTGGAGGTGATCTTTATGGAGTTGTCCATGAGAACACCCTCGTCAAACATAAAATGATCCTGCCTCCGAGAGCTAAGGGAACTGTCACCTTCCTTGCTGCTCCTGGTAATTACACAGTCGATGATGTTGTACTTGAAACGGAGTTTGATGGTGAGAAAACCAAGTTCACTATGCTGCAAGTTTGGCCTGTGAGGCAGCCCCGACCAGTCACGGAGAAACTCCCCGCTAACTACCCCTTATTGACTGGTCAGCGTGTATTGGATGCACTCTTCCCATGTGTCCAAGGAGGTACTACTGCCATCCCAGGTGCTTTCGGTTGCGGTAAAACTGTCATTTCTCAAGCTTTGTCAAAGTATTCCAACTCAGATGTCATTATCTACGTCGGCTGTGGAGAGAGAGGAAATGAAATGTCTGAGGTACTGAGAGATTTCCCTGAGTTGTCAGTTGAGATCGATGGTGTTACGGAATCCATCATGAAGAGAACAGCACTAGTAGCTAATACCTCTAACATGCCTGTAGCTGCCCGAGAAGCTTCTATCTATACTGGTATAACATTGTCTGAATACTTCAGAGATATGGGTTACAATGTTTCTATGATGGCTGACTCGACTTCTAGATGGGCTGAGGCTTTGAGAGAAATCTCTGGTCGGTTGGCTGAAATGCCTGCTGACAGTGGTTACCCTGCTTACTTGGGAGCTCGATTGGCTTCATTCTATGAACGTGCTGGCAGAGTCAAATGCCTTGGTAATCCAGAGAGGGAGGGATCTGTTTCAATTGTAGGAGCTGTATCACCTCCTGGTGGTGACTTCTCAGATCCTGTAACATCTGCCACTCTTGGTATTGTTCAAGTGTTCTGGGGATTGGACAAGAAGCTGGCTCAAAGAAAGCACTTTCCGTCCATCAACTGGCTTATTTCTTACAGCAAGTACATGAGAGCTTTAGACGATTTTTATGACAAGAACTTCCCTGAGTTTGTTCCACTTAGGACAAAAGTTAAAGAAATCCTTCAAGAAGAAGAAGATCTTTCAGAAATTGTACAACTGGTAGGTAAAGCCTCCCTTGCTGAATCTGATAAGATCACTCTTGAAATCGCCAAGCTACTCAAGGATGACTTCCTTCAACAAAACAGTTACTCACCTTATGATCGTTTCTGTCCATTCTACAAGACCGTCGGTATGTTGAAAAACATGATTTCCTTCTACGACCTGGCCCGACATGCAGTCGAATCTACAGCTCAAAGTGAAAAGAAGATTACATGGGCTGTAATCAAAGAAAGCATGGGCAATATTCTCTACCAGTTGTCGTCGATGAAGTTTAAGGACCCTGTAAAAGATGGTGAAGCTAAAATCAGATCAGACTTTGAGCAGATGCAAGAAGACATACAGCAAGCGTTTAGGAACCTCGAGGACTAACAACAGTCAGCTGTTAGCAATCATGTACTGTTGTTTGTTGTTCTCGACTTGTATTTTATCTCTCCCTACTGTTTATATATACATTTGTGTCCTCACCTCTTCGGAATGTCATTAATTCTTCATTTCTATGTATATATATTTACATATATAATAACAAAATGTATAATTATAAATTTGAAAAAGTGTATCATAATTCTTTAAACAATGACATTAAATGAAAAAAAAAAAAATTAAAAAAATATGAAAAGCAGAAATAGATTTAGATAACCACATTTAAACTTAAGATGGGGTCTTCGGGAACTAGTTTTCGGTCATTAGATGTTTTAATGATATTTTCAGATTTATTTAAGAGATTTATTAGGTGAAATTATTTAGGACCTAGATAGTTCAAATGACACTTCGGAGTGAACAGCAGTACTAGGGAATTTATCTATCCCAAGAATTTTAATGTAATTTACTTTTATTTGTTTAATTTTTGTTATCGATTGTTGTATAAGATTTTTTTTTTTTTGTTATATCTCTTCTCAATCATTCCGTTTGTTGTATGTAAACAAAAAAAAAGATATAAAATGTTAAAAATATAAAAATAATGAAATTAAGCAGTTATATCACGAGGTTTTAAAAAAAATAAATATATATAGTACTTATTTGAAGAGGATTTTTTTTTAAATTTTATCATGGTACTAAAAATTGTATCTAATATTCGAAGCACA

Protein: RF 2: 1877 -> 3718 (613 aa)

Comparison with *Halyomorpha halys*, PREDICTED: V-type proton ATPase catalytic subunit A - Sequence ID: XP_014272529.1

E: 0.0; bits= 1250

Query 1 MALPRIKDEDQESKFGYVFGVSGPVVTAEKMSGSAMYELVRVGYFELVGEIIRLEGDMAT 60

MALPRIKDEDQESKFGYVFGVSGPVVTAEKMSGSAMYELVRVGYFELVGEIIRLEGDMAT

Sbjct 1 MALPRIKDEDQESKFGYVFGVSGPVVTAEKMSGSAMYELVRVGYFELVGEIIRLEGDMAT 60

Query 61 IQVYEETSGVTVGDPVLRTGKPLSVELGPGILGSIFDGIQRPLKDINEISNSIYIPKGVN 120

IQVYEETSGVTVGDPVLRTGKPLSVELGPGILGSIFDGIQRPLKDINEISNSIYIPKGVN

Sbjct 61 IQVYEETSGVTVGDPVLRTGKPLSVELGPGILGSIFDGIQRPLKDINEISNSIYIPKGVN 120

Query 121 IPALSRSAAWEFTPTNIKVGSHITGGDLYGVVHENTLVKHKMILPPRAKGTVTFLAAPGN 180

IPALSRSAAWEF PTNIKVGSHITGGDLYGVVHENTLVKHKMILPPRAKGTVT+LAAPGN

Sbjct 121 IPALSRSAAWEFQPTNIKVGSHITGGDLYGVVHENTLVKHKMILPPRAKGTVTYLAAPGN 180

Query 181 YTVDDVVLETEFDGEKTKFTMLQVWPVRQPRPVTEKLPANYPLLTGQRVLDALFPCVQGG 240

YTVDDVVLETEFDGEKTKFTMLQVWPVRQPRPVTEKLPANYPLLTGQRVLDALFPCVQGG

Sbjct 181 YTVDDVVLETEFDGEKTKFTMLQVWPVRQPRPVTEKLPANYPLLTGQRVLDALFPCVQGG 240

Query 241 TTAIPGAFGCGKTVISQALSKYSNSDVIIYVGCGERGNEMSEVLRDFPELSVEIDGVTES 300

TTAIPGAFGCGKTVISQALSKYSNSDVIIYVGCGERGNEMSEVLRDFPELSVEIDGVTES

Sbjct 241 TTAIPGAFGCGKTVISQALSKYSNSDVIIYVGCGERGNEMSEVLRDFPELSVEIDGVTES 300

Query 301 IMKRTALVANTSNMPVAAREASIYTGITLSEYFRDMGYNVSMMADSTSRWAEALREISGR 360

IMKRTALVANTSNMPVAAREASIYTGITLSEYFRDMGYNVSMMADSTSRWAEALREISGR

Sbjct 301 IMKRTALVANTSNMPVAAREASIYTGITLSEYFRDMGYNVSMMADSTSRWAEALREISGR 360

Query 361 LAEMPADSGYPAYLGARLASFYERAGRVKCLGNPEREGSVSIVGAVSPPGGDFSDPVTSA 420

LAEMPADSGYPAYLGARLASFYERAGRVKCLGNPEREGSVSIVGAVSPPGGDFSDPVTSA

Sbjct 361 LAEMPADSGYPAYLGARLASFYERAGRVKCLGNPEREGSVSIVGAVSPPGGDFSDPVTSA 420

Query 421 TLGIVQVFWGLDKKLAQRKHFPSINWLISYSKYMRALDDFYDKNFPEFVPLRTKVKEILQ 480

TLGIVQVFWGLDKKLAQRKHFPSINWLISYSKYMRALDDFYDKNFPEFVPLRTKVKEILQ

Sbjct 421 TLGIVQVFWGLDKKLAQRKHFPSINWLISYSKYMRALDDFYDKNFPEFVPLRTKVKEILQ 480

Query 481 EEEDLSEIVQLVGKASLAESDKITLEIAKLLKDDFLQQNSYSPYDRFCPFYKTVGMLKNM 540

EEEDLSEIVQLVGKASLAESDKITLEIAKLLKDDFLQQNSYSPYDRFCPFYKTVGMLKNM

Sbjct 481 EEEDLSEIVQLVGKASLAESDKITLEIAKLLKDDFLQQNSYSPYDRFCPFYKTVGMLKNM 540

Query 541 ISFYDLARHAVESTAQSEKKITWAVIKESMGNILYQLSSMKFKDPVKDGEAKIRSDFEQM 600

I+FYDLARHAVESTAQSEKKIT+AVIKESMGNILYQ+SSMKFKDPVKDGE+KIR+DFEQ+

Sbjct 541 ITFYDLARHAVESTAQSEKKITFAVIKESMGNILYQMSSMKFKDPVKDGESKIRADFEQL 600

Query 601 QEDIQQAFRNLED 613

QEDIQQAFRNLED

Sbjct 601 QEDIQQAFRNLED 613

Graphical representation


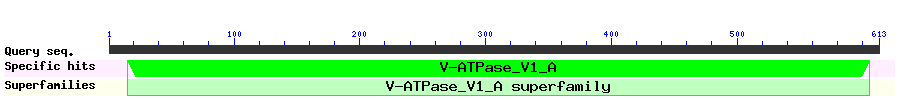


**Vacuolar H+ ATPase subunit C (vha16)**

>TRINITY_DN27944_c2_g4_i1 length= 939 nt

CTAGAGTAAAATTTTACAAAAAAACAAAACAAAACAAAAAAAAAAGCATTCTTGAGGCTCTACAATGGGAAAAAAGAACATAAAACTGAAAGAAAAAATTGAGGTGCAAAAAATTCGTAGGTAGAAAAAAAAATAAAAATAAAAAGTGCAATCATTTTCCAATCACTTCAATGCAACAAATTGATTCAACAGCTCAGAACATTAATATTTTCAATTTGAATGTAATAAAATAAAACAGGATATTTTGAAAGGAGGAATCCAAATTAGAAATCATGATTGGAAAGTGATTAACTATATTGTCTACAAGAATATACTGGTCAAAAAAGGGTAGCTGTTGAGCCCAAAAAGAATACCAAATGCATGATTTATTTTGTGTACAAATAGATTGCCACAATAAGTCCATAGAGACCTAATACTTCAGCAAAAATTAAAATAAGAATCATTCCAACAAACAATCGTGGCTGTTGAGCAGTTCCTCTAACACCTGCATCACCTACAATGCCAATGGCAAAGCCAGCAGCAAGACCACTAAACCCTACTGCTAAACCAGCTCCCAGATGCATAAAACCCTTGTATAGTGAATATTTGGCAGGAGGATCAAGAGCTCCTGCAATCAGTACAGCAACAACCAAGCCGTAAATAGCAATAATACCAGCCATGACAACAGGAATAATTGATTTCATGATCAACTCGGGTCGCATAACAGACATGGCTGCGATACCCGTTCCTGATTTTGCAGTTCCATAGGCTGCACCAAGAGCACTAAAAATCATAGCTGAAGCAGCTCCCATAACTCCAAAAAATGGTCCGTATATTGGGTTATCGCTAGACATTTTCGGTTGGATAATTCCTGAGCAGGAGCAAGTACCTCAGATGACAGAAATAGAAATGGACCACAAGCTGTCTTTACACCCCTAGACTTGCTGAGATCGGAAGAGC

Protein: RF -2: -833 -> -366 (155 aa)

Comparison with *Halyomorpha halys*, PREDICTED: V-type proton ATPase 16 kDa proteolipid subunit - Sequence ID: XP_014275063.1

E: 4e-100; bits= 289

Query 2 SSDNPIYGPFFGVMGAASAMIFSALGAAYGTAKSGTGIAAMSVMRPELIMKSIIPVVMAG 61

S+DNPIYGPFFGVMGAASAMIFSALGAAYGTAKSGTGIAAMSVMRPELIMKSIIPVVMAG

Sbjct 3 STDNPIYGPFFGVMGAASAMIFSALGAAYGTAKSGTGIAAMSVMRPELIMKSIIPVVMAG 62

Query 62 IIAIYGLVVAVLIAGALDPPAKYSLYKGFMHLGAGLAVGFSGLAAGFAIGIVGDAGVRGT 121

IIAIYGLVVAVLIAGALDPPAKYSLYKGFMHLGAGLAVGFSGLAAGFAIGIVGDAGVRGT

Sbjct 63 IIAIYGLVVAVLIAGALDPPAKYSLYKGFMHLGAGLAVGFSGLAAGFAIGIVGDAGVRGT 122

Query 122 AQQPRLFVGMILILIFAEVLGLYGLIVAIYLYTK 155

AQQPRLFVGMILILIFAEVLGLYGLIVAIYLYTK

Sbjct 123 AQQPRLFVGMILILIFAEVLGLYGLIVAIYLYTK 156

Graphical representation


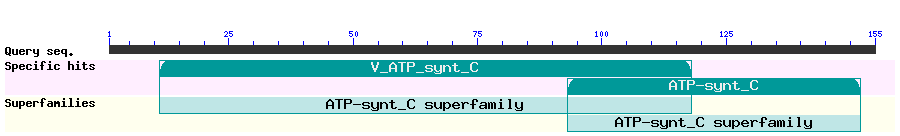


**Small Rab GTPases**

>TRINITY_DN26388_c3_g5_i1 length= 1923 nt

TCGGAATATATTGTGGAACGCCATTTTCTCACTCGAACCCAGTGACTTTGTGTTTATTATTTTTCCAAACAAGCTTGAACATGACATCAAGAAAGAAAGTTCTTTTGAAAGTTATCATTCTCGGTGATTCTGGAGTTGGAAAAACTTCACTCATGAACCAATATGTCAATAAAAAATTTTCTAACCAGTATAAGGCTACGATCGGTGCAGATTTTCTTACAAAAGAAGTATTAGTTGATGATCGGGTTGTGACAATGCAGATATGGGACACAGCAGGCCAGGAAAGGTTTCAGTCATTAGGTGTTGCGTTTTACCGAGGTGCAGATTGTTGTGTTCTTGTATTTGATGTATCTGCACCAACAACTTTTAAGTCTCTTGACTCATGGCGTGATGAATTTCTCATACAAGCATCTCCTAGAGATCCAGAGAACTTTCCATTTGTTCTGCTTGGAAACAAAGTTGATCTAGAAAATAGAGCGGTTTCTGCGAAAAGAGCGCAGCACTGGTGTCAGTCAAAGAACAATATTCCTTACTATGAAACAAGTGCAAAAGAGGCTATAAATGTCGAGCAAGCATTTTTAACTATTGCTAAAAATGCCCTCGCCCAAGAATCAGAGGCCAACCTGTATAATGAGTTCCCCGACCAAATTAAGCTGACTGGAGACCCAAAAGTACAAGTCCCAGATCAATGCGCCTGTTAGTTGAAGAACGTTTTCTGTGATTTCATCTTATGTGCTTTTGATGGAGCACATTGCATCTACTCAGTTCCGCATACCGGTTTTAATTGGGGGGGTCTCAGGGTAACAAATTGAATGATACGTTAATATATTTATATATATATGTTTGCATATATATAAACTGAAATATACATTATTTATATATAGACATATAAATTATAGAATTAATAATACATAAACTTATCAAATGCTAAATAATGTATTTACTGTTCTTTTACAGTAAATATAATTTTTTATCTATGTAATATTAGATTTATTATGTCAGTTGTTTTATTTTGTAAATACATGATTATGTGATACAAATTAAATGAAGTGCACCTTCAAACTGTATAGTCATGACATAATACTTAATTTGAGATTGTAAATTATGTTTTTGTATTTAAACCAGTTCAACGTTTGTTGTCGGTCATTAATATTATTGTTATTATTATTTTTTTTTTAATGCACTTCTTATCAATCTATATACTTTTTAATTAAACCCTAGCACTGGCAGTACTCTCATAAGTTTCAATTTCAGTTTGACTTTATTTATTTAGTTTATTTCTCTAATATATATAGTTTATAATGTATGATATGTATGTATATTAGATGAATTATATTGCATTTTATGAGGTTTTATTGAATGTTTGTTTACTTTATATTGAACTTACGTTTTCCAAGTTAATTTTTGTAAATTAGAATAATAGGTATCGGCAGTCTATAAACTTTTAAAATTCTTGTTTTCCATATTTTAGATCAGAACTATAACTTGTATTTGAAATTTTTTGATGTACTATACATCAAGTAAAATTGCTATTGAACTTGAAATTAAATATTTGTTTTAAAGTTTTATTGGTTTTTGTTTTGCTGGTTATATATAAGTTGTGATAAATATTATGTTGTCAATCAGAGTAGTATAAAATTATGCTGTTTGTATAGTATGACATGTTAAATATAGATTATATGCAATTCTAATAAAATGTACTAAATTCTATGAATGTATAGCTCCTTCAAGGTGGTTTTTGTAATGCATATGAAGAAGAGTGCTTTGTGTATAGCAGTTCAGTTCACTTTGTCATCGAATAAATAGATTGAATATTATTACATTTATATTCTCCTGCTTTTTAAAGTTTATTTTAAACATCATAATTGTGTTATTATTTAATTCAATTTACCCTTATTTAAAAAGTTAGGGCTATTAAAATAG

Protein: RF -3: -81 -> -701 (201 aa)

Comparison with *Halyomorpha halys*, PREDICTED: ras-related protein Rab-7a - Sequence ID: XP_014286452.1

E: 3e-152; bits= 425

Query 1 MTSRKKVLLKVIILGDSGVGKTSLMNQYVNKKFSNQYKATIGADFLTKEVLVDDRVVTMQ 60

MTSRKKVLLKVIILGDSGVGKTSLMNQYVNKKFSNQYKATIGADFLTKEVLVDDRVVTMQ

Sbjct 1 MTSRKKVLLKVIILGDSGVGKTSLMNQYVNKKFSNQYKATIGADFLTKEVLVDDRVVTMQ 60

Query 61 IWDTAGQERFQSLGVAFYRGADCCVLVFDVSAPTTFKSLDSWRDEFLIQASPRDPENFPF 120

IWDTAGQERFQSLGVAFYRGADCCVLVFDVSAPTTFKSLDSWRDEFLIQASPRDPENFPF

Sbjct 61 IWDTAGQERFQSLGVAFYRGADCCVLVFDVSAPTTFKSLDSWRDEFLIQASPRDPENFPF 120

Query 121 VLLGNKVDLENRAVSAKRAQHWCQSKNNIPYYETSAKEAINVEQAFLTIAKNALAQESEA 180

VLLGNKVDLENRAVSAKRAQHWCQSKNNIPYYETSAKEAINVEQAFLTIAKNALAQESEA

Sbjct 121 VLLGNKVDLENRAVSAKRAQHWCQSKNNIPYYETSAKEAINVEQAFLTIAKNALAQESEA 180

Query 181 NLYNEFPDQIKLTGDPKVQVPDQCAC 206

NLYNEFPDQIKLTGDPKV PDQCAC

Sbjct 181 NLYNEFPDQIKLTGDPKVPAPDQCAC 206

Graphical representation


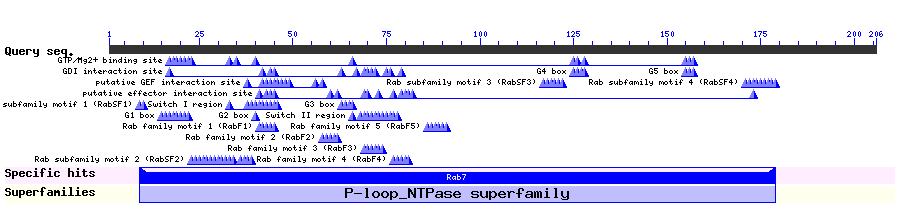


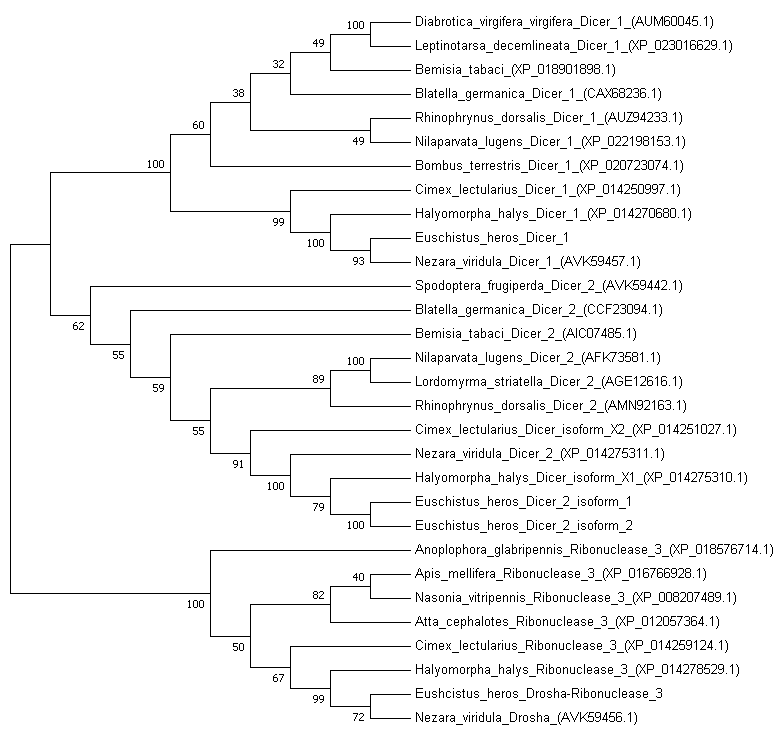


**Fig. S1 -** Phylogenetic tree of *Euschistus heros* Dicer 1 (DCR-1), Dicer 2 (DCR-2) and Drosha with the DCRs of other insect species. Proteins sequences were aligned using the MUSCLE and tested using the Neighbor-Joining Three. Numbers at each branch node represent the values calculated by bootstrap analysis (1,000 replications).


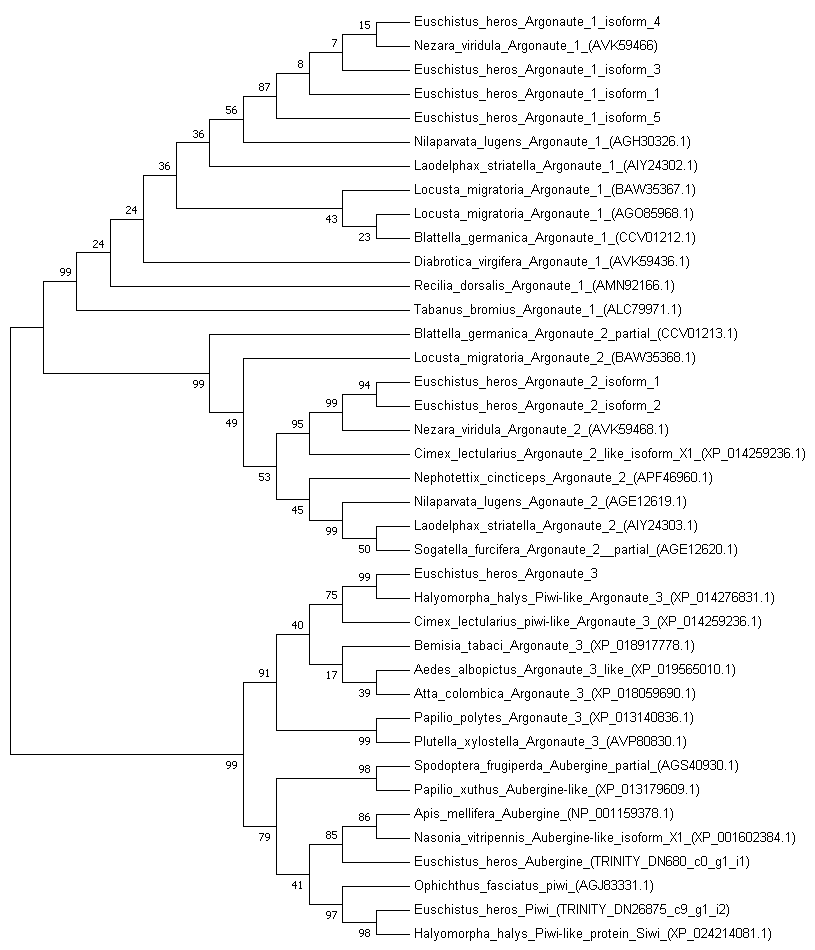


**Fig. S2 -** Phylogenetic tree of *Euschistus heros* Argonaute 1 (AGO-1), Argonaute 2 (AGO-2), Argonaute 3 (AGO-3), Aubergine (AUB) and Piwi with the AGOs of other insect species. Proteins sequences were aligned using the MUSCLE and tested using the Neighbor-Joining Three. Numbers at each branch node represent the values calculated by bootstrap analysis (1,000 replications).


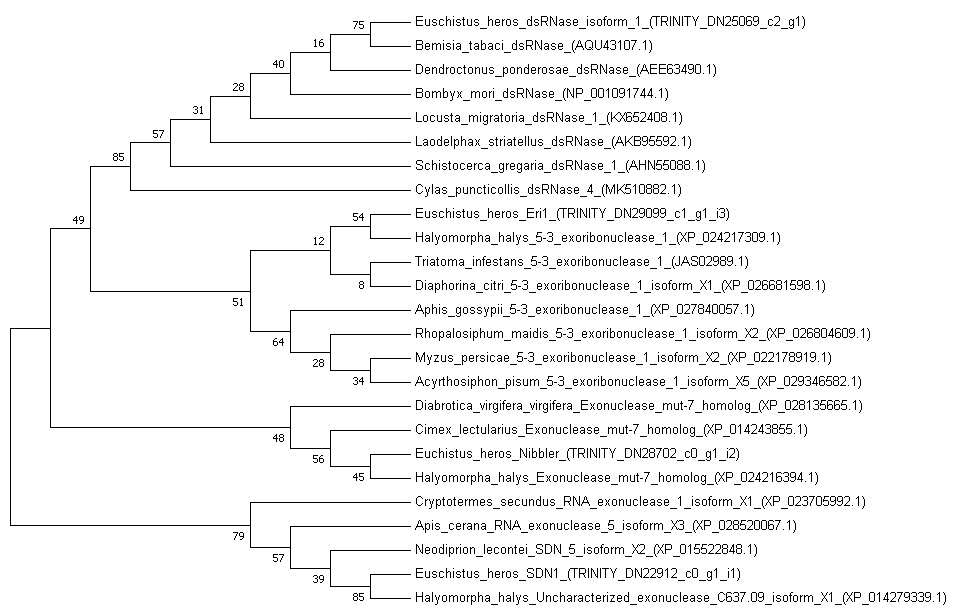
**Fig. S3 -** Phylogenetic tree of *Euschistus heros* nucleases, Eri-1, Nibbler, SDN1, and dsRNase with the nucleases of other insect species. Proteins sequences were aligned using the MUSCLE and tested using the Neighbor-Joining Three. Numbers at each branch node represent the values calculated by bootstrap analysis (1,000 replications).


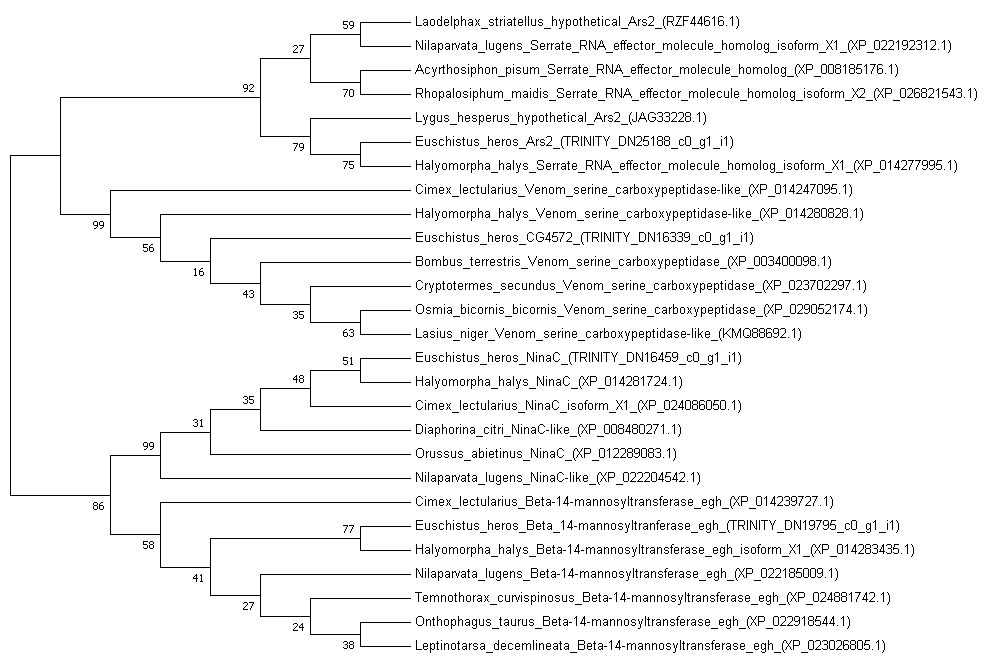


**Fig. S4 -** Phylogenetic tree of *Euschistus heros* antiviral RNAi proteins, Ars2, ninaC, egh, and CG4572 with the antiviral RNAi proteins of other insect species. Proteins sequences were aligned using the MUSCLE and tested using the Neighbor-Joining Three. Numbers at each branch node represent the values calculated by bootstrap analysis (1,000 replications).

A)

120 min

30 min

60 min

10 min

1 min

0 min


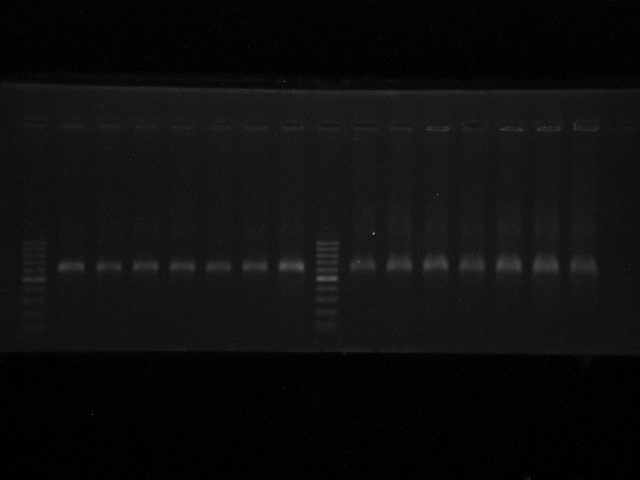


B)

0 min

120 min

1 min

10 min

30 min

60 min


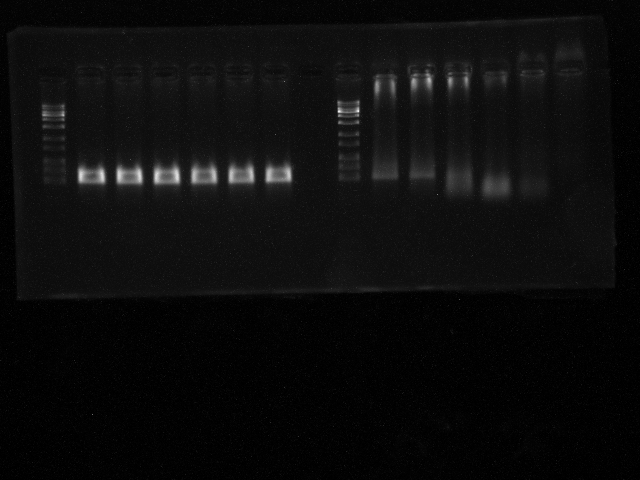


**Fig. S5 -** *Ex vivo* dsRNA degradation assay of different dsRNA formulations: (A) dsRNA-V-ATPase-A with water, (B) dsRNA-V-ATPase-A with hemolymph. The hemolymph of *E. heros* was extracted and incubated with 200 ng/µl of dsRNA-V-ATPase-A for different periods and run in 1 % agarose gel. The red arrow indicates the size of ~600 base pair.
